# Analysis of networks of host proteins in the early time points following HIV transduction

**DOI:** 10.1186/s12859-019-2990-3

**Published:** 2019-07-17

**Authors:** Éva Csősz, Ferenc Tóth, Mohamed Mahdi, George Tsaprailis, Miklós Emri, József Tőzsér

**Affiliations:** 1 Proteomics Core Facility, Department of Biochemistry and Molecular Biology, Faculty of Medicine, University of Debrecen, Egyetem ter 1., Debrecen, 4032 Hungary; 2 Laboratory of Retroviral Biochemistry, Department of Biochemistry and Molecular Biology, Faculty of Medicine, University of Debrecen, Egyetem ter 1., Debrecen, 4032 Hungary; 3 Arizona Research Labs, University of Arizona, PO Box 210066, Administration Building, Room 601, Tucson, AZ 85721-0066 USA; 4 The Scripps Research Institute, 132 Scripps Way, Jupiter, FL 33458 USA; 5 Department of Medical Imaging, Division of Nuclear Medicine and Translational Imaging, Faculty of Medicine, University of Debrecen, Nagyerdei krt. 98., Debrecen, 4032 Hungary

**Keywords:** Weighted network, Quantitative proteomics, Host response, HIV-1

## Abstract

**Background:**

Utilization of quantitative proteomics data on the network level is still a challenge in proteomics data analysis. Currently existing models use sophisticated, sometimes hard to implement analysis techniques. Our aim was to generate a relatively simple strategy for quantitative proteomics data analysis in order to utilize as much of the data generated in a proteomics experiment as possible.

**Results:**

In this study, we applied label-free proteomics, and generated a network model utilizing both qualitative, and quantitative data, in order to examine the early host response to Human Immunodeficiency Virus type 1 (HIV-1). A weighted network model was generated based on the amount of proteins measured by mass spectrometry, and analysis of weighted networks and functional sub-networks revealed upregulation of proteins involved in translation, transcription, and DNA condensation in the early phase of the viral life-cycle.

**Conclusion:**

A relatively simple strategy for network analysis was created and applied to examine the effect of HIV-1 on host cellular proteome. We believe that our model may prove beneficial in creating algorithms, allowing for both quantitative and qualitative studies of proteome change in various biological and pathological processes by quantitative mass spectrometry.

**Electronic supplementary material:**

The online version of this article (10.1186/s12859-019-2990-3) contains supplementary material, which is available to authorized users.

## Background

Utilization of state-of the art proteomics methods can generate thousands of data points, and extensive information on proteins present in the sample can be obtained. High-resolution shotgun proteomics can provide both qualitative and quantitative information about proteins, and can be applied in an unbiased way to study the complete proteome [1, 2]. Despite the high amount of data available, it is sometimes difficult to acquire relevant biological information, in which case sophisticated analytical methods and capable software are needed [3].

Network analysis is widely used in biological data analysis for examination of transcriptomic, proteomic or metabolomic datasets [4–6], and for analyzing interactions between various molecules [7, 8]. In the cellular environment, most of the proteins exert their biological function as part of a complex, or in the form of interactions with other proteins, therefore, application of protein-protein interaction (PPI) analysis methods is advantageous [9]. PPI networks can provide a new layer of information, allowing for the utilization of currently available data, in addition to possibly unravelling hidden biological phenomena [10, 11].

New concepts on network analysis are emerging helping the understanding of biological complexity [12], however, in most cases, only the presence or absence of the protein is considered, the available quantitative data can hardly be incorporated into the network analyses.

The replication cycle of human immunodeficiency virus-1 (HIV-1) is a complex, multi-step, and highly regulated process. The cycle typically begins with viral attachment to cell surface receptors, and ending with the production of infectious virions. Due to the multiple processes involved, the replication cycle has been classically divided into two distinct phases; the early and late phase. The early phase encompasses cell binding, fusion, internalization, uncoating, reverse transcription, as well as integration of the viral cDNA into the host genome. On the other hand, transcription of viral genome, export of viral RNA, assembly of virions at the plasma membrane, as well as budding and maturation of the released virions are parts of the late phase of the replication cycle [13, 14]. While late phase events are relatively well characterized, the precise mechanism and regulation of early phase steps remain poorly understood.

Genomics and proteomics studies were carried out to investigate how HIV-1 hijacks the host cellular machinery, avoiding being sensed by host immune responses. siRNA screens were implemented to study the cellular genes and proteins required for HIV-1 infection [9, 15, 16], HIV-1 protein – host protein protein-protein interaction networks were generated, and the data were deposited in HIV-1 Human Interaction Database [17].

In case of HIV infection, the network-based examinations have identified perturbed host cellular systems; such as the proteasome and transcriptional regulation, and have revealed that HIV-1 preferably interacts with highly connected and central cellular proteins [18–20].

In this study, we have generated the protein expression profiles of cells during early HIV-1 infection using protein mass spectrometry, and integrated the acquired data with knowledge-based protein-protein interaction network to understand how cellular network is perturbed by HIV.

## Results

Our aim was to analyze the proteomic landscape of the early stage of HIV-1 based lentiviral vector transduction. 293 T cells were infected with VSV-G pseudotyped HIV-1 vector, and 0, 4 and 12 h post-infection, cell lysates were harvested. Label-free proteomics was applied to examine protein-level changes. Duplicate samples for three time points were collected (0, 4, and 12 h post-transduction) in case of virus transduced samples and in case of control, mock transduced samples. The collected 6 virus treated and 6 control samples were analyzed in duplicates, allowing for the measurement of two technical and two biological replicates for each time point.

The mass spectrometry proteomics data have been deposited into the ProteomeXchange Consortium [21] via the PRIDE partner repository with the dataset identifier PXD010436 and 10.6019/PXD010436.

Identified proteins (Additional file 1) were manually curated, and in the case of non-human or non-viral identifications, the sequences were verified. In many instances, they were mistakenly designated as non-human proteins, in which case it was corrected. In few instances, the non-human proteins could not be matched to any of the human or viral proteins, and consequently, these sequences were omitted from further analyses. The data for Rhodobacter capsulatus cytochrome c, bovine pancreatic trypsin inhibitor, bovine serum albumin and pig trypsin were kept to serve as reference for quantitative analyses, but were not used for further computations. The relative amount of proteins was computed based on spectral counting and in case of each protein the mean of the results of the four analyses corresponding to each condition was calculated (Additional file 2).

### Statistical analysis

Firstly, a qualitative analysis was carried out to detect newly expressed or down-regulated proteins in the first 4 or 12 h after HIV-1 pseudovirion transduction. Only those proteins were considered for statistical analysis which could be quantified in at least 2 out of 4 replicates, and were not quantified in other conditions. HIST1H1E, HNRNPL, PRRC2A and TRIM28 were quantified only at H04, and there were no proteins quantified solely in H12 time point (Additional files 1, 2). HIST1H1E interacts with linker DNA between nucleosomes, and functions in DNA condensation, HNRNPL and TRIM28 play a role in translation and transcription, while PRRC2A plays a role in inflammatory processes.

Some of the proteins were quantified in all time points except H12. These include ALYREF, CCDC86, CSDA, COX5A, HN1, MYL6, PPIF, SEPT2, SRSF6, TCOF1, and TPM3 (Additional file 1). These proteins participate in RNA binding (ALYREF, CCDC86, SRSF6, TCOF1), DNA binding (CSDA), protein folding (PPIF), energy generation (COX5A), signalization (HN1) and cytoskeleton assembly (MYL6, SEPT2, TPM3).

**Fig. 1:**
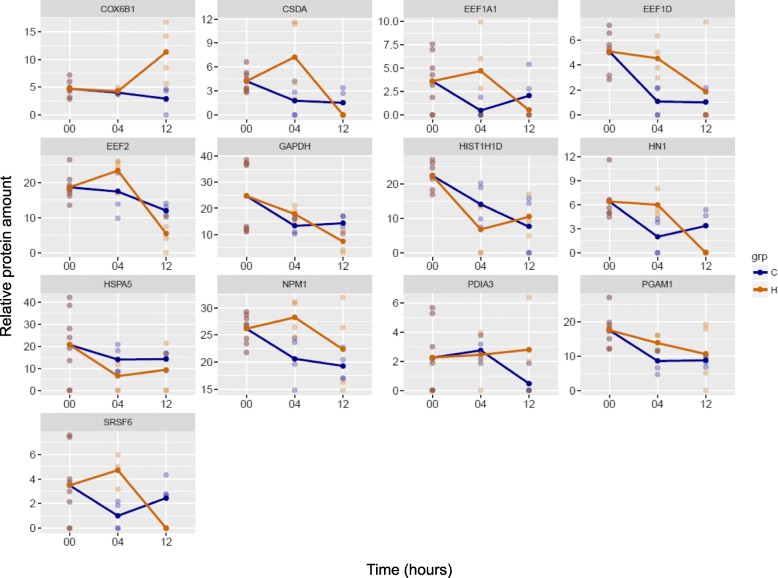
Relative protein amounts showing statistically significant changes in HIV-1 treated samples compared to controls. The x axis shows the time-points of sample collection in hours, and the y axis shows the relative protein amounts. Blue color refers to control (C), and yellow color to the HIV-1 treated sample (H)

In order to examine changes in the amount of proteins, statistical analysis was carried out (Additional file 3). The amount of CSDA, EEF1A1, EEF1D, HN1, NPM1, PGAM1 and SRSF6 increased significantly, while that of HIST1H1D and HSPA5 significantly decreased in H04 (Fig. 1). It is interesting to note that after peaking in H04, CSDA, HN1 and SRSF6 were not quantified in H12. In H12, compared to C12, the amount of COX6B1 and PDIA3 increased, while that of EEF2 and GAPDH decreased significantly (Fig. 1). When the function of proteins showing statistically significant changes was examined, we observed an increase in the amount of proteins implicated in RNA binding in H04, and an overall decrease in their amount in H12.

### Network analysis

**Fig. 2:**
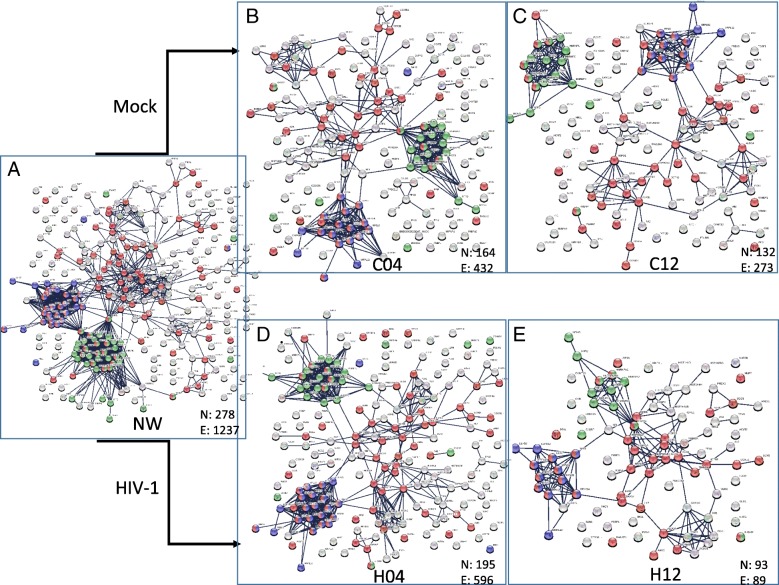
Protein-protein interaction network of the proteins quantified in each condition. The PPI networks were generated by STRING (confidence 0.95) using the list of quantified proteins presented in Additional file 2 in case of each condition. The number of nodes (N) and the number of edges (E) according to STRING in case of each network is indicated. **a**. PPI network of proteins in the 0 h time point (NW), **b**. PPI network of proteins in the 4 h time point in control, mock-transduced cells (C04). **c** PPI network of proteins in the 12 h time point in control, mock-transduced cells (C12). **d**. PPI network of proteins in the 4 h time point in HIV vector-transduced cells (H04). **e**. PPI network of proteins in the 12 h time point in HIV vector- transduced cells (H12). Red dots represent proteins belonging to transport GO term, blue dots indicate proteins having a role in translation, while green dots indicate proteins with a role in RNA splicing according to the functional enrichment analysis provided by STRING

To broaden our insight, and to better understand the possible functional associations of protein changes upon HIV-1 pseudovirion transduction, we have searched for the available protein-protein interactions of the quantified proteins in our datasets. For evaluation of the interactions, the STRING database was used, which contains information on known and predicted, direct physical, and indirect functional protein-protein interactions [22]. Only interactions which were of high confidence (interaction score in STRING database > 0.95) were used. Initially, five binary interaction networks were generated: NW0 combined proteins from mock- and virion-treated cell lysates collected at 0 time-point, C04 and C12 networks contained proteins from the mock-treated cells collected 4 h and 12 h post-infection, respectively, and the H04 and H12 networks contained proteins from the HIV-1 transduced cells collected at 4 and 12 h time-points, respectively (Fig. 2). The number of nodes and the number of edges of the networks show a decreasing trend over time, with a marked shrinkage in H12.

**Fig. 3:**
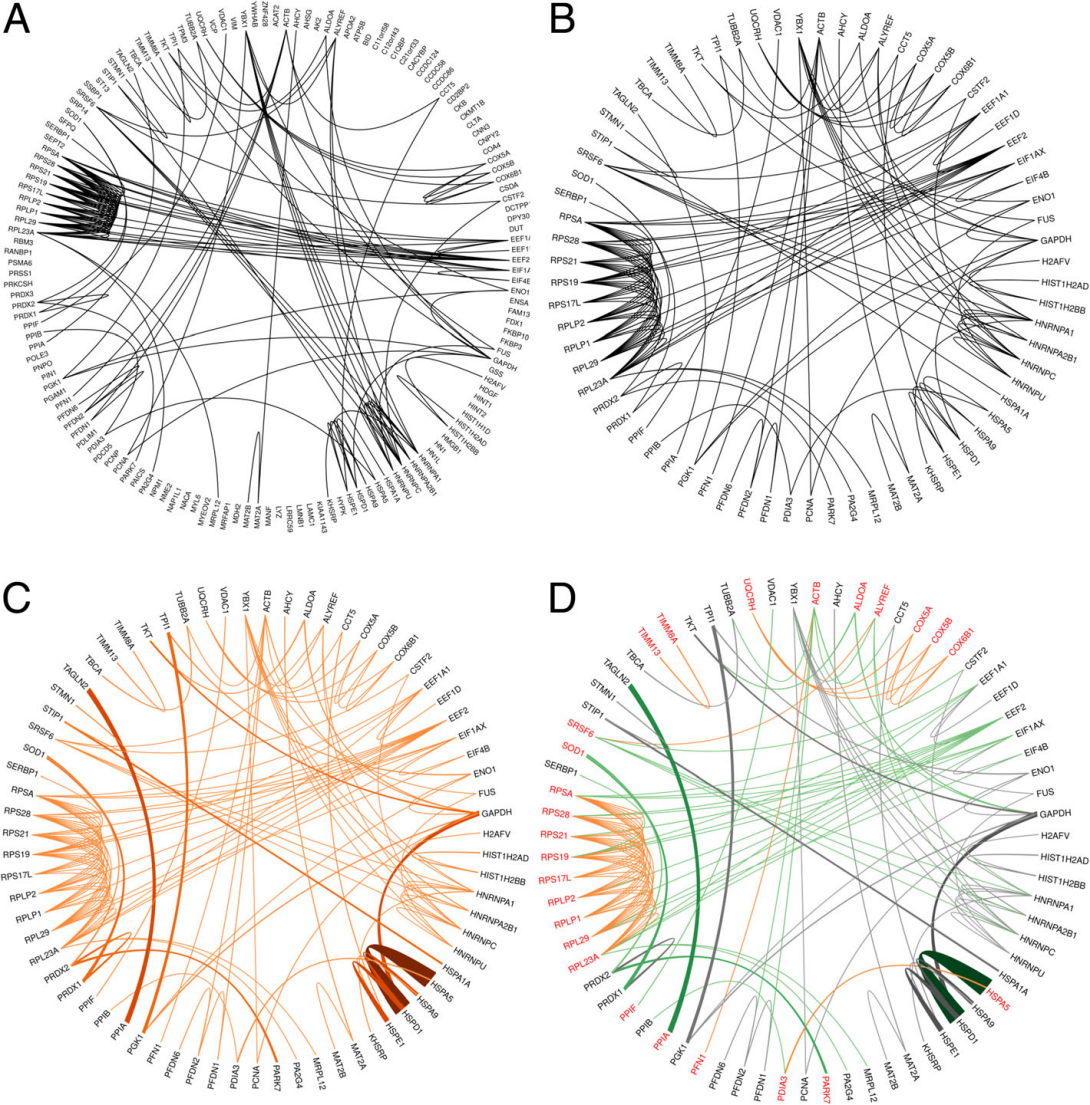
Network generation pipeline. Representative network drawn by circlize, showing data for sample1 and interactions generated by STRING. **a**. Binary network containing all the identified proteins arranged in alphabetical order on the external ring of the circular plot. Thin black curves show the possible interactions generated by STRING. **b**. Binary network containing only the proteins with interactions. The isolated proteins (i.e. without any connection) were eliminated. Thin black curves show the possible interactions generated by STRING. **c**. Weighted network containing the interacting proteins. Orange lines represent interactions, the higher the intensity of the color and thickness of the line the higher the interaction strength. **d**. Weighted network with functional feature. A randomly selected GO function (GO:0044765) is used to illustrate the functional network. Red proteins are part of the functional sub-network, while black proteins are not, being considered as non-functional proteins. The weighted interactions are color-coded according to the protein-pair classification: functional – functional interactions are orange, non-functional – non-functional interactions are gray, and functional – non-functional interactions are green. The interaction strength is represented by the intensity of the color and thickness of the line: the higher the intensity of the color and the thickness of the line, the higher the interaction strength

**Fig. 4:**
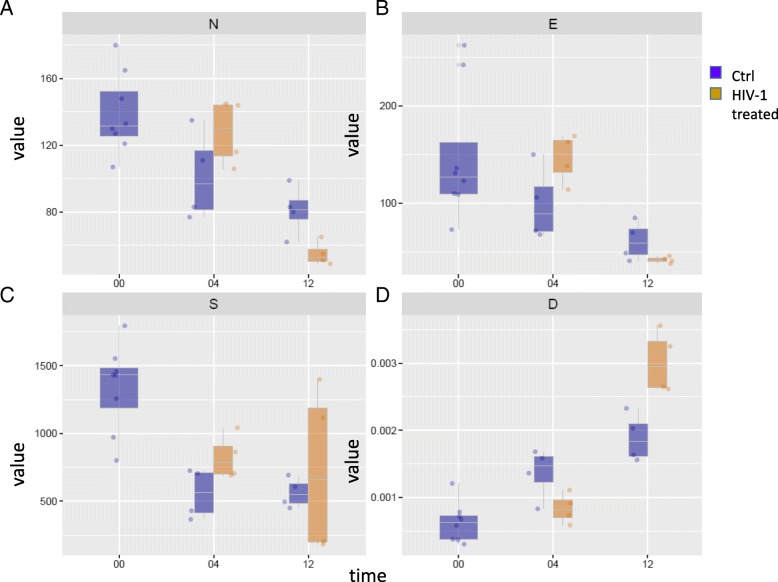
Network parameters. **a**. Number of nodes (N), **b**. number of edges (E), **c**. network strength (S), **d**. strength or edge density (D) in case of networks observed in the examined conditions. The y axis show the mean value characteristic for each parameter, and the x axis indicates the time points. Blue color refers to control, while the yellow color to the HIV-1 treated conditions

These binary networks provide information solely on the possibility of interaction between two proteins (Fig. 2, Fig. 3a, b), hence, in order to gain more realistic information, protein amounts measured by spectral counting were implemented into the network using a simple statistical model. In this way, binary edges were transformed into estimated protein pair’s interaction intensities in the sample, which is proportional to the amounts of proteins participating in the interaction, and inversely proportional to the number of interactions (Fig. 3c). The weighted networks were examined, and the number of nodes (N), edges (E), network strength (S), edge density (D) and functional and non-functional edge ratio (R) were calculated (Fig. 4).

The number of nodes decreased significantly in H12, indicating network shrinkage in H12, observed in the binary network (Fig. 4a). The number of edges and network strength did not change in a statistically significant manner (Fig. 4b, c), however, edge density decreased significantly in H04 while increasing significantly in H12 (Fig. 4d). These changes indicate the presence of a less interactive network in H04, and a smaller; yet more active, PPI network in H12 (Fig. 4a, d).

**Fig. 5:**
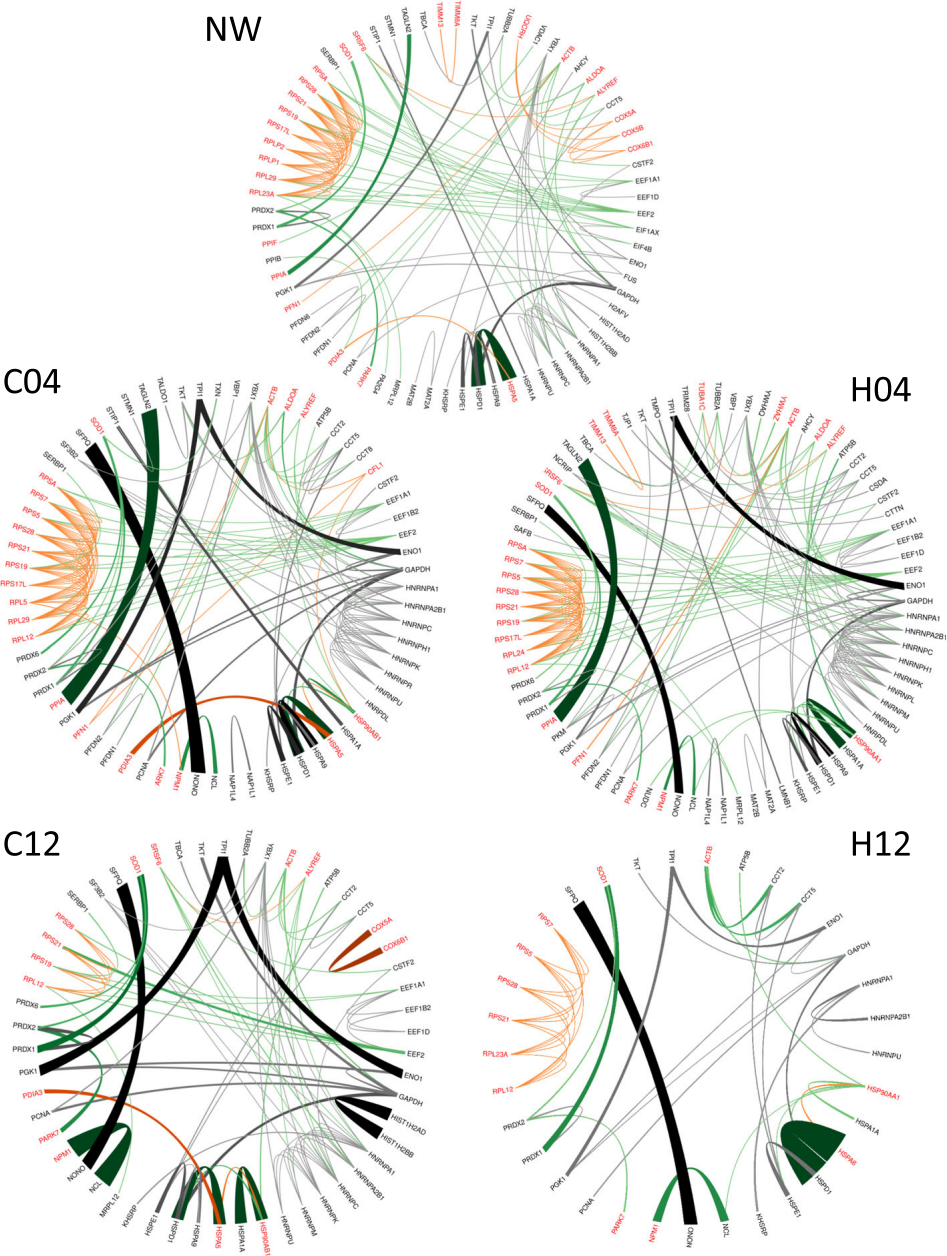
Network changes visualized in case of a representative functional sub-network. The representative figure shows the changes in weighted networks in case of proteins belonging to randomly selected GO:0044765 GO term. NW represents the weighted PPI network of interacting proteins in the 0 h time point, C04 and C12 correspond to PPI networks of interacting proteins in the 4 h and 12 h time point, respectively, in control, mock-transduced cells. H04 and H12 represent the weighted PPI network of interacting proteins in the 4 h and 12 h time point, respectively, in HIV vector-transduced cells. Red proteins are part of the functional sub-network, while black proteins are non-functional proteins. The weighted interactions are color-coded according to the protein-pair classification: functional – functional interactions are orange, non-functional – non-functional interactions are gray, and functional – non-functional interactions are green. The interaction strength is represented by the intensity of the color and thickness of the line: the higher the intensity of the color and the thickness of the line, the higher the interaction strength

Next, we were eager to analyze the functionality of the networks, and hence, we generated functional sub-networks of proteins belonging to GO terms. All the Molecular Function, Biological Process and Cellular Component GO terms listed as enriched by STRING in C04, H04, C12 and H12; where at least 10 protein per GO function in any of the networks were present, were considered. To visualize network changes, the GO.0044765 term was chosen randomly (Fig. 3d), and the change of this sub-network was visualized in all time points (Fig. 5). Proteins present in a given GO term listed as enriched by STRING were considered as being part of the functional sub-network (f), whereas proteins not being part of the specific GO term, were considered as non-functional (n) proteins. Three types of interactions were analyzed: i) interactions between proteins belonging to functional sub-networks (f), ii) interactions between proteins not belonging to functional sub-network (n), and iii) interactions between functional and non-functional proteins (c – cross) (Fig. 3d). In order to better understand the changes, a statistical approach was applied, and the following network parameters were calculated: in case of each functional (f) network of proteins belonging to a specific GO term, the Nf, Ef, Sf, Df, and Rf, while for non-functional (n) proteins the Nn, En, Sn, Dn, and Rn network parameters were calculated. In case of interactions between the functional and non-functional proteins (c) the Ec, Sc, Dc, and Rc network parameters were calculated (Additional file 4).

According to our hypothesis, those GO functions or functional sub-networks might be responsible for the changes induced by HIV-1, where the parameters in the functional network change significantly, whereas in the non-functional network, no statistically significant changes are shown. At the same time, those GO functions where the parameters in the functional network do not change in a statistically significant manner, yet do so in the non-functional sub-networks, are thought to not explain the changes related to HIV-1 transduction.

After statistical analysis and FDR correction of the results (Additional file 5), in case of some GO terms, statistically significant differences were observed. No significant difference in edge and strength values were observed in any of the functional sub-networks (Ef and Sf), and the number of nodes was significantly reduced in H12 only in the case of 5 functional sub-networks (Additional file 6). Considering edge density (D) and ratio (R), only those GO terms were further considered where (i) the significant difference was present only in the functional sub-network (Df and Rf, respectively) and (ii) where the significant difference was present both in the functional sub-network (Df and Rf) and in the cross network (Dc and Rc) (Additional file 6). According to our hypothesis, proteins belonging to the GO terms listed in Table 1 and Table 2, are responsible for the changes of cellular proteome observed in the H04 and H12 networks in response to HIV-1 transduction. In H04 sample, an increase in the node number (proteins present in the network) was observed, however, this increase was not significant. In the same time, a global decrease in interactivity; represented by the number of edges, was noticed. Proteins which might be responsible for this reduced interactivity belong to the RNA processing-related functions (splicing, RNA synthesis, RNA catabolism, translation, transcription), regulation of cell death, regulation of cellular response to stress, viral life cycle (viral gene expression, viral transcription, viral life cycle) and protein localization, and some very general GO terms; such as protein binding, cellular macromolecular biosynthetic process, purine nucleotide binding, organic substance transport, etc. (Table 1). In spite of the reduced global interactivity, some functional sub-networks; such as viral process, protein kinase binding, multi-organism process, de novo protein folding and protein complex subunit organization, show significantly increased interactivity (Table 1).

**Table 1:** List of GO terms with significantly different changes in the functional sub-network in H04

GO code	GO term	Significantly changed parameter	Direction of change in H04
GO.0000184	nuclear-transcribed mRNA catabolic process, nonsense-mediated decay	Df	decrease
GO.0000228	nuclear chromosome	Df	decrease
GO.0000375	RNA splicing, via transesterification reactions	Df	decrease
GO.0000398	mRNA splicing, via spliceosome	Df	decrease
GO.0000956	nuclear-transcribed mRNA catabolic process	Df	decrease
GO.0003735	structural constituent of ribosome	Df	decrease
GO.0005198	structural molecule activity	Df	decrease
GO.0005515	protein binding	Df	decrease
GO.0005524	ATP binding	Df, Dc	decrease
GO.0005622	intracellular	Df	decrease
GO.0005737	cytoplasm	Df	decrease
GO.0005739	mitochondrion	Df	decrease
GO.0005840	ribosome	Df	decrease
GO.0006351	transcription, DNA-templated	Df, Dc	decrease
GO.0006366	transcription from RNA polymerase II promoter	Df, Dc	decrease
GO.0006367	transcription initiation from RNA polymerase II promoter	Df	decrease
GO.0006396	termination of RNA polymerase II transcription	Df	decrease
GO.0006397	mRNA processing	Df	decrease
GO.0006401	RNA catabolic process	Df	decrease
GO.0006402	mRNA catabolic process	Df	decrease
GO.0006412	translation	Df	decrease
GO.0006413	translational initiation	Df, Dc	decrease
GO.0006414	translational elongation	Df, Dc	decrease
GO.0006415	translational termination	Df	decrease
GO.0006417	regulation of translation	Df, Dc	decrease
GO.0006518	peptide metabolic process	Df	decrease
GO.0006605	protein targeting	Df	decrease
GO.0006614	SRP-dependent cotranslational protein targeting to membrane	Df	decrease
GO.0006732	coenzyme metabolic process	Df, Dc	decrease
GO.0006886	intracellular protein transport	Df	decrease
GO.0008104	protein localization	Rf	decrease
GO.0008152	metabolic process	Df	decrease
GO.0008380	RNA splicing	Df	decrease
GO.0009056	catabolic process	Df	decrease
GO.0009892	negative regulation of metabolic process	Df	decrease
GO.0009987	cellular process	Df	decrease
GO.0010468	regulation of gene expression	Df	decrease
GO.0010556	regulation of macromolecule biosynthetic process	Df	decrease
GO.0010608	posttranscriptional regulation of gene expression	Df, Dc	decrease
GO.0010941	regulation of cell death	Df	decrease
GO.0015031	protein transport	Df	decrease
GO.0016032	viral process	Df	increase
GO.0016043	cellular component organization	Df	decrease
GO.0016070	RNA metabolic process	Df, Dc	decrease
GO.0016071	mRNA metabolic process	Df	decrease
GO.0016482	cytoplasmic transport	Df	decrease
GO.0016604	nuclear body	Df, Dc	decrease
GO.0017076	purine nucleotide binding	Df, Dc	decrease
GO.0018130	heterocycle biosynthetic process	Df, Dc	decrease
GO.0019058	viral life cycle	Df	decrease
GO.0019080	viral gene expression	Df	decrease
GO.0019083	viral transcription	Df	decrease
GO.0019222	regulation of metabolic process	Df	decrease
GO.0019438	aromatic compound biosynthetic process	Df, Dc	decrease
GO.0019538	aromatic compound biosynthetic process	Df	decrease
GO.0019899	enzyme binding	Df	decrease
GO.0019900	kinase binding	Df, Dc	increase
GO.0019901	protein kinase binding	Df	increase
GO.0022607	cellular component assembly	Df	decrease
GO.0022626	cytosolic ribosome	Df	decrease
GO.0030554	adenyl nucleotide binding	Df, Dc	decrease
GO.0031625	ubiquitin protein ligase binding	Df, Dc	decrease
GO.0031974	membrane-enclosed lumen	Df	decrease
GO.0032550	purine ribonucleoside binding	Df, Dc	decrease
GO.0032553	ribonucleotide binding	Df, Dc	decrease
GO.0032555	purine ribonucleotide binding	Df, Dc	decrease
GO.0032774	RNA biosynthetic process	Df, Dc	decrease
GO.0034248	regulation of cellular amide metabolic process	Df	decrease
GO.0034613	cellular protein localization	Rf	decrease
GO.0034622	cellular macromolecular complex assembly	Df	decrease
GO.0034645	cellular macromolecule biosynthetic process	Df	decrease
GO.0034654	nucleobase-containing compound biosynthetic process	Df, Dc	decrease
GO.0034655	nucleobase-containing compound catabolic process	Df	decrease
GO.0035639	purine ribonucleoside triphosphate binding	Df, Dc	decrease
GO.0036094	small molecule binding	Df, Dc	decrease
GO.0042981	regulation of apoptotic process	Df	decrease
GO.0043065	positive regulation of apoptotic process	Df	decrease
GO.0043066	negative regulation of apoptotic process	Df, Dc	decrease
GO.0043226	organelle	Df	decrease
GO.0043227	membrane-bounded organelle	Df, Dc	decrease
GO.0043229	intracellular organelle	Df	decrease
GO.0043231	intracellular membrane-bounded organelle	Df	decrease
GO.0043233	organelle lumen	Df	decrease
GO.0044085	cellular component biogenesis	Df	decrease
GO.0044237	cellular metabolic process	Df	decrease
GO.0044238	primary metabolic process	Df	decrease
GO.0044248	cellular catabolic process	Df	decrease
GO.0044249	cellular biosynthetic process	Df	decrease
GO.0044260	cellular macromolecule metabolic process	Df	decrease
GO.0044265	cellular macromolecule catabolic process	Df	decrease
GO.0044267	cellular protein metabolic process	Df	decrease
GO.0044271	cellular nitrogen compound biosynthetic process	Df, Dc	decrease
GO.0044391	ribosomal subunit	Df	decrease
GO.0044422	organelle part	Df	decrease
GO.0044424	intracellular part	Df	decrease
GO.0044444	cytoplasmic part	Df	decrease
GO.0044446	intracellular organelle part	Df	decrease
GO.0044451	nucleoplasm part	Df, Dc	decrease
GO.0044454	nuclear chromosome part	Df	decrease
GO.0044802	single-organism membrane organization	Df, Dc	decrease
GO.0045184	establishment of protein localization	Df	decrease
GO.0046907	intracellular transport	Df, Dc	decrease
GO.0051084	de novo posttranslational protein folding	Df	increase
GO.0051704	multi-organism process	Df, Dc	increase
GO.0060255	regulation of macromolecule metabolic process	Df	decrease
GO.0060548	negative regulation of cell death	Df, Dc	decrease
GO.0065003	macromolecular complex assembly	Df	decrease
GO.0070013	intracellular organelle lumen	Df	decrease
GO.0070972	protein localization to endoplasmic reticulum	Rf	decrease
GO.0071013	catalytic step 2 spliceosome	Df	decrease
GO.0071702	organic substance transport	Df, Dc	decrease
GO.0071704	organic substance metabolic process	Df	decrease
GO.0071705	nitrogen compound transport	Df, Dc	decrease
GO.0071822	protein complex subunit organization	Df	increase
GO.0071840	cellular component organization or biogenesis	Df	decrease
GO.0072594	establishment of protein localization to organelle	Df	decrease
GO.0080090	regulation of primary metabolic process	Df	decrease
GO.0080135	regulation of cellular response to stress	Df, Dc	decrease
GO.0090304	nucleic acid metabolic process	Df, Dc	decrease
GO.0097367	carbohydrate derivative binding	Df, Dc	decrease
GO.1901362	organic cyclic compound biosynthetic process	Df, Dc	decrease
GO.1901566	organonitrogen compound biosynthetic process	Df	decrease
GO.1901575	organic substance catabolic process	Df	decrease
GO.1901576	organic substance biosynthetic process	Df	decrease
GO.1902580	single-organism cellular localization	Df	decrease

In case of each GO term where statistically significant changes were observed in network parameters in HIV-1 treated cells at 4 h time-point the GO identifier, the changed parameter and the direction of change is indicated

**Table 2:** List of GO terms with significantly different changes in the functional sub-networks in H12

GO code	GO term	Significantly changed parameter	Direction of change in H12
GO.0005759	mitochondrial matrix	Nf	decrease
GO.0003674	molecular function	Df, Dc	increase
GO.0003723	RNA binding	Rf, Rc	increase
GO.0005488	binding	Df, Dc	increase
GO.0005615	extracellular space	Rf, Rc	increase
GO.0005634	nucleus	Df, Dc	increase
GO.0005654	nucleoplasm	Df, Dc	increase
GO.0005739	mitochondrion	Df	increase
GO.0005743	mitochondrial inner membrane	Df, Dc	increase
GO.0005759	mitochondrial matrix	Df	increase
GO.0005856	cytoskeleton	Df, Dc	increase
GO.0006401	RNA catabolic process	Df, Dc	increase
GO.0006402	mRNA catabolic process	Df, Dc	increase
GO.0009058	biosynthetic process	Rf, Rc	decrease
GO.0009987	cellular process	Df, Dc	increase
GO.0010467	gene expression	Rf, Rc	increase
GO.0016032	viral process	Rf	increase
GO.0016604	nuclear body	Nf	decrease
GO.0017076	purine nucleotide binding	Df, Dc	increase
GO.0019058	viral life cycle	Df, Dc	increase
GO.0022607	cellular component assembly	Df	increase
GO.0030054	cell junction	Df	increase
GO.0031966	mitochondrial membrane	Df, Dc	increase
GO.0031981	nuclear lumen	Df, Dc	increase
GO.0043066	negative regulation of apoptotic process	Df	increase
GO.0043209	myelin sheath	Df	increase
GO.0043232	intracellular non-membrane-bounded organelle	Df	increase
GO.0043933	macromolecular complex subunit organization	Rf, Rc	increase
GO.0044237	cellular metabolic process	Df	increase
GO.0044265	mitochondrial nucleoid	Df, Dc	increase
GO.0044428	nuclear part	Df, Dc	increase
GO.0044430	cytoskeletal part	Df, Dc	increase
GO.0044451	nucleoplasm part	Nf	decrease
GO.0044712	single-organism catabolic process	Nf	decrease
GO.0044765	single-organism transport	Df, Dc	increase
GO.0044822	poly(A) RNA binding	Rf	increase
GO.0051084	de novo posttranslational protein folding	Rf, Rc	increase
GO.0051704	multi-organism process	Rf, Rc	increase
GO.0060548	negative regulation of cell death	Df	increase
GO.0071013	catalytic step 2 spliceosome	Df	increase
GO.0071705	nitrogen compound transport	Nf	decrease
GO.0071822	protein complex subunit organization	Rf	increase
GO.1901575	organic substance catabolic process	Df	increase

In case of each GO term where statistically significant changes were observed in network parameters in HIV-1 treated cells at 12 h time-point the GO identifier, the changed parameter and the direction of change is indicated

In H12, a statistically significant reduction of the node numbers and shrinkage of the network; along with a significant increase in interactivity, was observed (Fig. 4). The proteins responsible for the increased interactivity (increased Df and Rf values) belong to RNA binding, RNA catabolic process, viral life cycle, viral process, negative regulation of cell death, de novo posttranslational protein folding, protein complex subunit organization, and cellular metabolic process, etc. (Table 2). The cell junction and the myelin sheet GO terms also appear in H12, however, when proteins belonging to these GO terms were examined, it was found that they are part of more general GO terms from the list; such as intracellular non-membrane-bounded organelle or nucleus, extracellular space, etc. In case of biosynthetic process functional sub-network (GO.0009058), a decrease in the Rf was observed.

## Discussion

Genome-wide RNA interference-based screens were carried out to evaluate more than 20,000 human gene products to determine their alteration in HIV infection [23, 24]. A previous study showed an overall downregulation of cellular genes encoding for nuclear proteins, and genes involved in DNA replication and protein synthesis in the early stages of the early phase of viral infection [25], in a pattern that was confirmed by our analysis (Table 1). Upregulation of cellular genes was only found to occur at a later time point, peaking at 22 h post-infection, additionally, analysis on T cells showed that the most profound changes in cellular proteome appear 24 h after infection, at time points related to the late phase of infection [26].

It was found that up to 300 host cellular genes were involved in the life cycle of HIV-1, and while the identity of the genes was divergent among different studies, they were found more or less to belong to similar pathways [27, 28]. Network analysis is widely used in the examination of protein-protein interactions, providing information regarding protein changes on a different level, giving a more ample view of the alterations and perturbations of the biological systems as a result of a particular treatment. During analysis of PPIs, the presence or absence of a protein is evaluated, and the interactions, in light of existing evidence (ex. experimental data, literature search, computational methods), are displayed [29, 30]. STRING is a widely used, constantly updated, and expanding database of PPIs [22], used for the examination of verified, or potential interactions among proteins of interests. These networks are rich in information on protein clusters and functions based on Gene Ontology (GO), however, enrichment of GO terms does not handle protein amounts, therefore, reflecting theoretical, rather than actual parameters. Meanwhile, the use of highly accurate mass spectrometry techniques provide analytical data that is wealthy in quantity as well as quality. There were few attempts made to introduce the quantitative data into the network analysis [31, 32]. In order to implement quantitative data into the PPI networks, instead of the widely used binary networks, a weighted network often utilized in information science [33] was used in this study. Taking into account the protein amount reflected by the normalized total spectra, instead of the probabilistic assumption [32], we choose a simple statistical model. In our model, the protein pair’s interaction is proportional to their amount in the sample, and inversely proportional to the number of possible interactions listed in the PPI network generated by STRING for proteins present in the sample. After including the interaction density values as network edge weights; calculated by our method, we could determine a sort of weighed network parameters for the statistical investigation of network alterations.

In our study, we aimed at characterizing the cellular proteome changes in the early stage of HIV-1 infection, within the 0–12 h time interval. Generation of weighted networks, and analysis of functional sub-networks revealed that the dynamics of protein level changes in sub-networks is different in HIV-1 transduced samples 12 h post-infection. Expectedly, in the very early stages of infection, proteins involved in translation, transcription and DNA condensation were upregulated, notably HIST1H1E, HNRNPL, PRRC2A and TRIM28. Some other proteins; such as ALYREF, CCDC86, CSDA, COX5A, HN1, MYL6, PPIF, SEPT2, SRSF6, TCOF1, and TPM3, prominently associated with RNA binding, cytoskeleton assembly, and signaling were quantified in all time points except H12.

Examining the binary networks, two protein clusters could be observed. One comprising proteins having a role in translation and ribosome biogenesis, and the other containing proteins from the hnRNP family with a role in RNA splicing (Fig. 2). The functional sub-network containing the ribosome component proteins did not show a statistically significant change, and with this, we can demonstrate on protein level the same findings observed by Kleinman et.al. at gene level, who could not observe statistically significant difference in case of genes having a role in ribosome biogenesis at 12 h time point [34]. Regarding the other cluster containing mainly hnRNP proteins, we could not observe a statistically significant change in network parameters among the different time points. However, literature data show that host RNA splicing is altered upon HIV-1 infection, and the level of class A/B and H of hnRNP proteins changes; initially decreased 6–12 days post infection, thereafter increased [35]. At the same time, it was shown that some proteins of this cluster; such as HNRNPH1, HNRNPU and SRSF6, are so called HIV-1 dependency factors [36] and are required by HIV-1. These data are derived from later time-points, as most of the experiments do not examine such early events at 4 h or 12 h post infection.

Considering the results of the analyses, based on the weighted networks, we could identify increased cellular metabolic processes comprising increased RNA binding and catabolism, cellular component assembly, along with increased viral process and inhibition of apoptosis (increased negative regulation of apoptotic process). RNA binding was shown to be increased upon RNA virus infection; Garcia-Moreno et al. observed an increased activity of RNA-binding proteins upon sindbis virus (SINV) infection at 18 h time point [37]. At the same time, they observed an increased binding of RNA binding proteins to viral RNAs. This implies a massive downregulation of the host mRNAs 18 h post infection, involving mainly the housekeeping genes [37]. In case of HIV-1 infection, global siRNA studies indicate that a statistically significant portion of the host factors participate in mRNA transport [18].

Cells infected with HIV-1 usually die by apoptosis, hence prevention of apoptosis might help maintain the viral reservoir in the host [18, 38]. It was shown that a fraction of infected immune cells survive, highlighting the importance of escaping from apoptosis in the development of viral reservoirs [38]. A mixed pattern of upregulation and downregulation of genes involved in antiviral defense and cell death signaling were observed by Mohammadi et al. at early time points [24]. Inhibition of apoptosis increases the virus production in HIV-1 infected cells [39], and modulation of this system might be a good possibility for a therapeutic intervention [40].

Based on our data on the weighted networks, HSPA8 shows an increased interactivity in H12 datasets (Fig. 5a). HSPA8 and other members of the Hsp70 family play a key role during viral infection either as receptors for the virus, as chaperons aiding the protein folding, or as transporters between organelles [18, 41, 42].

Hijacking of the host system by HIV-1 is a complex phenomenon with early and late events. In the early phases of the viral infection, the virus utilizes cellular RNA and protein production machinery for its replication. It was observed that by 15 h post infection, all viral transcripts were produced by the cells, and 18 h after infection, the virus budding commences [24]. Chang et al.;. using next generation sequencing, observed a considerable viral mRNA level in infected cells 12 h post-infection [43]. In this sense, examining the host response 48 h [15, 44] or 6 days post-infection [45] cannot provide us with information on the very early events. Observations made by Kleinmann et al. analyzing the dataset generated by Chang et al., show that at 12 h post infection, the gene expression profiles are similar to the mock samples, and clear distinctions could only be made after 24 h, highlighting the necessity of more sensitive methods for the examination of early events of HIV-1 infection.

It is challenging to properly compare our results to those presented in the scientific literature, since the commonly used starting time point examined is 48 h post infection, in case of HIV-1. However, considering the findings presented by different groups; either on HIV-1 or other RNA virus infections, our findings are in good agreement with previous studies analyzing transcriptomic and proteomic changes upon virus infection in these very early time points. The use of non-primary HIV-1 cell targets; such as HEK, and pseudotyped virions, and the application of data-dependent sampling [46], may indeed limit interpretation of the results. The utilization of other cell types and data acquisition methods with higher reproducibility; such as parallel reaction monitoring [2] or data independent acquisition [47], might give more accurate input data. In spite of the above limitations, we believe that this model of proteomic data evaluation serves as a good starting point for further development of algorithms implementing not only qualitative, but also quantitative data generated in a given proteomic experiment, and that such a combination will undoubtedly aid in the understanding and deciphering of complex biological phenomena.

## Conclusion

A weighted network model facilitating the use of both qualitative and quantitative data, acquired in a label-free proteomics experiment was generated and applied to examine the early host response to HIV-1. Upregulation of proteins involved in translation, transcription and DNA condensation in the early phase of the viral life-cycle could be observed, highlighting the utility of our weighted PPI network data analysis approach. More studies are required to further demonstrate the utility of this new data**-**driven weighted network based analysis, and it should be noted that the current model has a serious limitation. The strength of different protein-protein interactions in the edge weight calculation; due to the lack of information, is not yet included. However, the applied weight-model can easily be extended to use this type of information as soon as any public database becomes available. We hope that this approach can open new ways for creating algorithms, allowing for both quantitative and qualitative studies of proteome change in various biological and pathological processes by quantitative mass spectrometry.

## Methods

### Production of viral particles

Viral particles were produced with some modifications of a previously utilized protocol [48]. Briefly, recombinant viruses were produced by transient transfection of 293 T cells (ATCC® CRL­3216™) using pWOX-CMV-GFP (transfer vector plasmid), pMDLg/pRRE (packaging plasmid), pRSV.rev (Rev-coding plasmid), and pMD. G (VSV-G envelope protein-coding plasmid). Vectors were a kind gift from D. Trono (University of Geneva Medical School, Geneva, Switzerland) [49], and were subsequently modified by our research group [48]. Salmon sperm DNA (Sigma-Aldrich) was also added. Media containing virus particles was concentrated by Ultracel-100 K Amicon Ultra Centrifugal Filter (Millipore), and stored in − 70 °C. Quantity of pseudovirions produced was assessed by measurement of reverse transcriptase (RT) activity using a colorimetric kit (Sigma-Aldrich, Roche).

### Transduction and sample collection

293 T cells in T-25 cell culture flasks were either mock-treated or transduced at 50% confluency with 5 ng RT equivalent of the HIV-based pseudovirions, in the presence of 4 μg/ml polybrene (Sigma-Aldrich), in 1 ml total volume, and incubated at 37 C°. After 0, 4, and 12 h, cells were trypsinized for 10 min, then washed tree times with ice-cold PBS to remove non-fused pseudovirion particles. The final pellet was suspended in 4 ml lysis buffer (150 mM sodium chloride, 1.0% Triton X-100, 0.5% sodium deoxycholate, 0.1% sodium dodecyl sulfate (SDS), and 50 mM Tris) pH 8.0, supplemented with cOmplete protease inhibitor cocktail (Sigma-Aldrich), incubated for 30 min at room temperature, centrifuged, and the supernatant was mixed with 24 ml cold (− 20 C°) acetone and stored at − 20 C° overnight.

### Mass spectrometry analysis

The cleared cell lysates were acetone-precipitated with six volumes of cold acetone overnight. The precipitates were re-dissolved in 25 mM ammonium bicarbonate (Sigma-Aldrich) and digested in-solution with trypsin [50]. The tryptic fragments were used for replicate LC-MS/MS analyses at University of Arizona in Tucson, AZ, USA.

500 ng per 5 μL injected protein lysate spiked with 300 fmol of Rhodobacter capsulatus cytochrome c T33 V mutant, was analyzed using a LTQ Orbitrap Velos mass spectrometer (Thermo Fisher Scientific) equipped with an Advion nanomate ESI source (Advion), after Omix (Agilent Technologies) C18 sample clean-up according to the manufacturer’s instructions. Peptides were eluted from a C18 precolumn (100-μm × 2 cm, Thermo Fisher Scientific) onto an analytical column (75-μm × 10 cm, C18, Thermo Fisher Scientific) using a 165 min gradient of solvent A (water, 0.1% formic acid) and solvent B (acetonitrile, 0.1% formic acid). The flow rate was 500 nl/minute. Data-dependent analysis (DDA) was performed by the Xcalibur v 2.1.0 software [51] using a survey mass scan at 60,000 resolution in the Orbitrap analyzer scanning mass/charge 350–1600, followed by collision-induced dissociation tandem mass spectrometry (MS/MS) at 35 normalized collision energy of the 14 most intense ions in the linear ion trap analyzer. Precursor ions were selected by the monoisotopic precursor selection setting with selection or rejection of ions held to a +/− 10 ppm window. Singly charged ions were excluded from MS/MS. Dynamic exclusion was set to place any selected m/z on an exclusion list for 45 s after a single MS/MS. Tandem mass spectra were searched against the UniprotKB/Swiss-Prot release available on December 12, 2014 without species restriction. At the time of the search, this database contained 459,734 entries. All MS/MS spectra were searched using Thermo Proteome Discoverer 1.3 (Thermo Fisher Scientific) considering fully tryptic peptides with up to 2 missed cleavage sites. Variable modifications considered during the search included methionine oxidation (15.995 Da), and cysteine carbamidomethylation (57.021 Da). The parent ion mass tolerance was 10 ppm, while the fragment tolerance was 0.8 Da. Proteins were identified at 99% confidence with XCorr score cut-offs [52] as determined by a reversed database search. The protein and peptide identification results were validated with Scaffold v4.4.6. (Proteome Software Inc.) [1]. Peptide identifications were accepted if they had greater than 89% probability to achieve an FDR less than 0.1% by the Scaffold Local FDR algorithm. Protein identifications were accepted if they had greater than 99% probability and contained at least 2 identified peptides. Protein probabilities were assigned by the ProteinProphet algorithm [53]. Proteins that contained similar peptides and could not be differentiated based on MS/MS analysis alone were grouped to satisfy the principles of parsimony. Proteins sharing significant peptide evidence were grouped into clusters.

Protein quantification was done based on spectral counting; the quantitative values were generated by the Scaffold program based on the normalized total spectra. In case of protein clusters, each peptide was used only once for quantification for the first human protein in the cluster, as listed by Scaffold. All quantitative data were used for statistical analyses; none of the data points were removed.

### Statistical analysis of proteomics data

For both statistical and network analysis, we used in-house developed R-software based on STRING [54–57], circlize (https://jokergoo.github.io/circlize_book/book/), MASS [55], lsmeans [56], matrixStats [57], reshape2 [58] and ggplot2 [59] packages. Assuming that data from technical repetitions are often characterized by Poisson distribution [60], and the large variances of biological replicas can be modelled by negative binomial distribution [61], we used modified general linear models to describe group-level differences in measured protein data in the 4 and 12 h time points. For each protein; after fitting negative binomial generalized linear model [55], we performed a post-hoc analysis [62] to characterize time-dependent mean differences by z score, and corrected *p* values for multiple comparisons.

### Network analysis

Gene names of the identified human proteins were subjected to STRING database [22] and five PPI networks were generated. The NW0 combined proteins from mock- and HIV-1 plasmid-treated cell lysates collected at 0 time-point, the C04 and C12 networks contained proteins from the mock cells collected 4 and 12 h post-infection, respectively, while the H04 and H12 networks contained proteins from the HIV-1 treated cells collected at 4 and 12 h time-points, respectively. Very high confidence interactions (interaction score > 0.95) in between the query proteins were used for the generation of each binary network. In these networks, the nodes were the proteins and the edges indicated the interactions between proteins as they were present in STRING. For network generation, the SRING R-package and the STRING database was applied, and the 0.95 combined score value to generate the binary networks B_t,s_ (B_0_, B_4h,C_, B_4h,H_, B_12h,C_, B_12h,H_) corresponding to the protein sets. In these networks, the binary edges indicated only the possibility of the interactions, taking no notice of the quantity.

To estimate the real interaction density, binary networks (B_t,s_) generated by STRING were further modified, and the amount of proteins measured by spectral counting was used to add w_ij_ weights to the edges. In this way, the existence of edges provides information on the existence of interaction, and the strength of protein pair’s interactions were estimated by this edge-weight model:

1 $$ {w}_{ij}=\frac{n_i}{k_i}\frac{n_j}{k_j} $$

where w_ij_ represents the interaction density between protein P_i_ and P_j_; n_i_, n_j_ means the quantity while k_i_, k_j_ denote the degree (the number of edges) of P_i_ and P_j_ in the given B_t,s_ binary network.

In this calculation, we used the measured data (n_i_, n_j_), which enabled us to alter the theoretical binary PPI network into a realistic, sample related interaction network, in which the weights of the edges are in direct proportion to the quantities and in inverse proportion to all interaction possibilities of the connected proteins in the given sample.

Because we can consider the n_i_ as the number of molecules of the protein P_i,_ the n_i_/k_i_ ratio represents the number of P_i_ molecules involved in one interaction of P_i_, and thus, the interaction density between P_i_ and P_j_ can be described by the product of n_i_/k_i_ and n_j_/k_j_. It should be mentioned that the used edge-weight model in the absence of a strong interactor protein may overestimate the effect of other weak interactor proteins, also, interaction strength data cannot be achieved in a classical quantitative proteomics experiment, and currently are unavailable in publicly accessible databases.

### Functional subnetwork construction

In order to investigate the PPI networks of the proteins belonging to GO (*geneontology.org/*) terms, we marked in each W_t,s_ the nodes by a function flag, which indicated whether or not the protein belongs to a given f-function; in our case, to a GO term. The so-called functional enrichment according to GO terms was done by STRING, using default settings and the Molecular Function, Biological Process and Cellular Component GO terms listed as enriched by STRING in C04, H04, C12 and H12, where at least 10 protein per GO function in any of the networks was present, were considered. This procedure defined a sort of W^f^_t,s_ functional networks, and divided them into two disjunctive sub-networks (F ^f^_t,s_ functional, belonging to the GO term and NF ^f^_t,s_ non-functional not being part of the respective GO term), containing the functional and the non-functional nodes, respectively. Because of this separation, the edges (i.e. the interactions) were also classified into three classes: functional edges between the functional nodes, non-functional edges between non-functional nodes and cross-edges in between functional and non-functional nodes, depending on the f-markers of the connected proteins.

### Examination of the global characteristics of the evaluated PPI networks

Any undirected weighted PPI network W(N,E) consists of two sets: N nodes and E edges. Each of the links (interactions) is defined by a couple of nodes (proteins) P_i_ and P_j_, and its value is w_ij_. Since the direction of interaction cannot be ordered, the connectivity matrix became symmetric: w_ij_ = w_ji_.

#### Number of nodes (N) and edges (E)

N, Nf and Nn denotes the number of nodes (i.e. proteins) in the whole network and the functional and non-functional sub-networks, respectively, with the following relation:

2 $$ \mathrm{N}=\mathrm{Nf}+\mathrm{Nn} $$

E denotes the number of edges (i.e. interactions) in the whole network. Ef and En are the number of edges within the functional and the non-functional sub-networks, respectively. The number of cross-edges (Ec) shows the connected proteins between the functional and the non-functional sub-networks. The edge numbers follow the next relation:

3 $$ \mathrm{E}=\mathrm{Ef}+\mathrm{En}+\mathrm{Ec} $$

#### Network strength and averaged node strength (S)

We defined the network strength S as the total sum of the weights of edges:

4 $$ S=\frac{1}{2}\ {\sum}_{i,j=1}^N{w}_{i,j} $$

In the functional networks we can calculate strength of whole network (S), and the functional (Sf) and non-functional sub-networks (Sn), as well. The sum of cross connection edges can be calculate as follows:

5 $$ \mathrm{Sc}=\mathrm{S}\hbox{-} \mathrm{Sf}\hbox{-} \mathrm{Sn} $$

#### Edge-weight density or strength density (D)

the edge-weight density measures how the weighted network is saturated by strong edges:

6 $$ D=\frac{S}{w_{max}\frac{N\left(N-1\right)}{2}} $$

In the functional networks we can measure the edge-weight density of the whole network (D) and the functional (Df) and non-functional sub-networks (Dn), as well.

Edge-weight ratio (R): using the network strength we can define the edge-weight ratio parameter for the two sub-networks:

7 $$ Rf=\frac{Sf}{S} $$

and the non-functional relative edge-weight density:

8 $$ Rn=\frac{Sn}{S} $$

Since the distribution of network parameters was not Gaussian or negative binomial, we used Wilcoxson tests [63] to characterize the group-related differences at the 4 and 12 h time points. The evaluated *p*-values were corrected for multiple comparisons by false discovery rate methods [64].

## Additional files

Additional file 1: List of identified proteins. (XLSX 1147 kb)

Additional file 2: List of quantified proteins. The gene name according to UniProt in case of quantified proteins is given, and for each protein, the mean amount of four replicates is presented for each time point except NW, where the mean amount of 8 replicates is given. (XLSX 23 kb)

Additional file 3: Statistical analysis of protein quantities. The gene name according to UniProt, the *p* value and z score for the 4 h and 12 h time points are listed in case of each protein. The lists are presented in ascending order of the *p* values. (XLSX 24 kb)

Additional file 4: Network parameters calculated for functional sub-networks. The y axis show the mean value characteristic for each parameter, and the x axis indicates the time points. Blue color refers to the control, while the yellow color to the HIV-1 treated conditions. N refers to the number of nodes, E to the number of edges, S show network strength, D represents the edge density and R the edge ratio. The f refers to the functional sub-network, the n to the non-functional subnetwork containing the proteins not present in the functional sub-network, while the c refers to the interactions between the functional and the non-functional sub-networks. (PDF 9676 kb)

Additional file 5: Statistical analysis of network parameters. The FDR-corrected p value and z score for the 4 h and 12 h time points, respectively, in case of each network parameter calculated (N, Nf, Nn, E, Ef, En, Ec, S, Sf, Sn, Sc, D, Df, Dn, Dc, Rf, Rn, Rc) for each GO function presented in Additional file 4. (XLSX 287 kb)

Additional file 6: List of GO terms with network parameters that were significantly changed in the functional sub-networks. (XLSX 16 kb)

## Data Availability

The mass spectrometry datasets generated during the current study were deposited to the ProteomeXchange database and are available via the PRIDE repository with the dataset identifier PXD010436 and 10.6019/PXD010436. All data analyzed during this study are included in this published article [and its supplementary information files].

## Abbreviations

cDNA: Complementary DNA
D: Saturation of the PPI interaction density in the sample
Dc: Saturation of the PPI interaction density between the functional and non-functional proteins in the sample
Df: Saturation of the PPI interaction density of the functional proteins in the sample
DMEM: Dulbecco’s modified Eagle’s medium
Dn: Saturation of the PPI interaction density of the nonfunctional proteins in the sample
DNA: Deoxyribonucleic acid
E: Number of interactions in the network generated from STRING with combined score of 0.95
Ec: Number of interactions between the functional and non-functional proteins
Ef: Number of interactions between the functional proteins
En: Number of interactions between the non-functional proteins
FBS: Fetal bovine serum
GO: Gene Ontology
HEK: Human embryonic kidney cells
HIV: Human immunodeficiency virus
MIPS: Monoisotopic precursor selection
N: Number of proteins in the network
Nf: Number of proteins in the functional sub-network
Nn: Number of proteins in the non-functional sub-network
PBS: Phosphate buffered saline
PPI: Protein-protein interaction
ppm: Parts per million
R: Relative PPI density, edge-weight ratio
Rc: Relative cross-functional PPI density, edge-weight ratio between the functional and non-functional PPI networks
Rf: Relative functional PPI density, edge-weight ratio between in the functional PPI network
Rn: Relative non-functional PPI density, edge-weight ratio in the non-functional PPI network
RNA: Ribonucleic acid
RT: Reverse transcriptase
S: PPI density of the sample
Sc: PPI density of the cross-functional proteins in the sample
Sf: PPI density of the functional proteins in the sample
siRNA: Small interfering RNA
Sn: PPI density of the non-functional proteins in the sample
VSV: Vesicular stomatitis virus
