# Supplementary material for: Analysis of networks of host proteins in the early time points following HIV transduction
Source: BMC Bioinformatics. 2019 Jul 17;20:398. doi: 10.1186/s12859-019-2990-3 (PMC6637640; doi:10.1186/s12859-019-2990-3)
Supplement: Supplementary file 4 — Network parameters calculated for functional sub-networks. The y axis show the mean value characteristic for each parameter, and the x axis indicates the time points. Blue color refers to the control, while the yellow color to the HIV-1 treated conditions. N refers to the number of nodes, E to the number of edges, S show network strength, D represents the edge density and R the edge ratio. The f refers to the functional sub-network, the n to the non-functional subnetwork containing the proteins not present in the functional sub-network, while the c refers to the interactions between the functional and the non-functional sub-networks. (PDF 9676 kb) [file 12859_2019_2990_MOESM4_ESM.pdf]

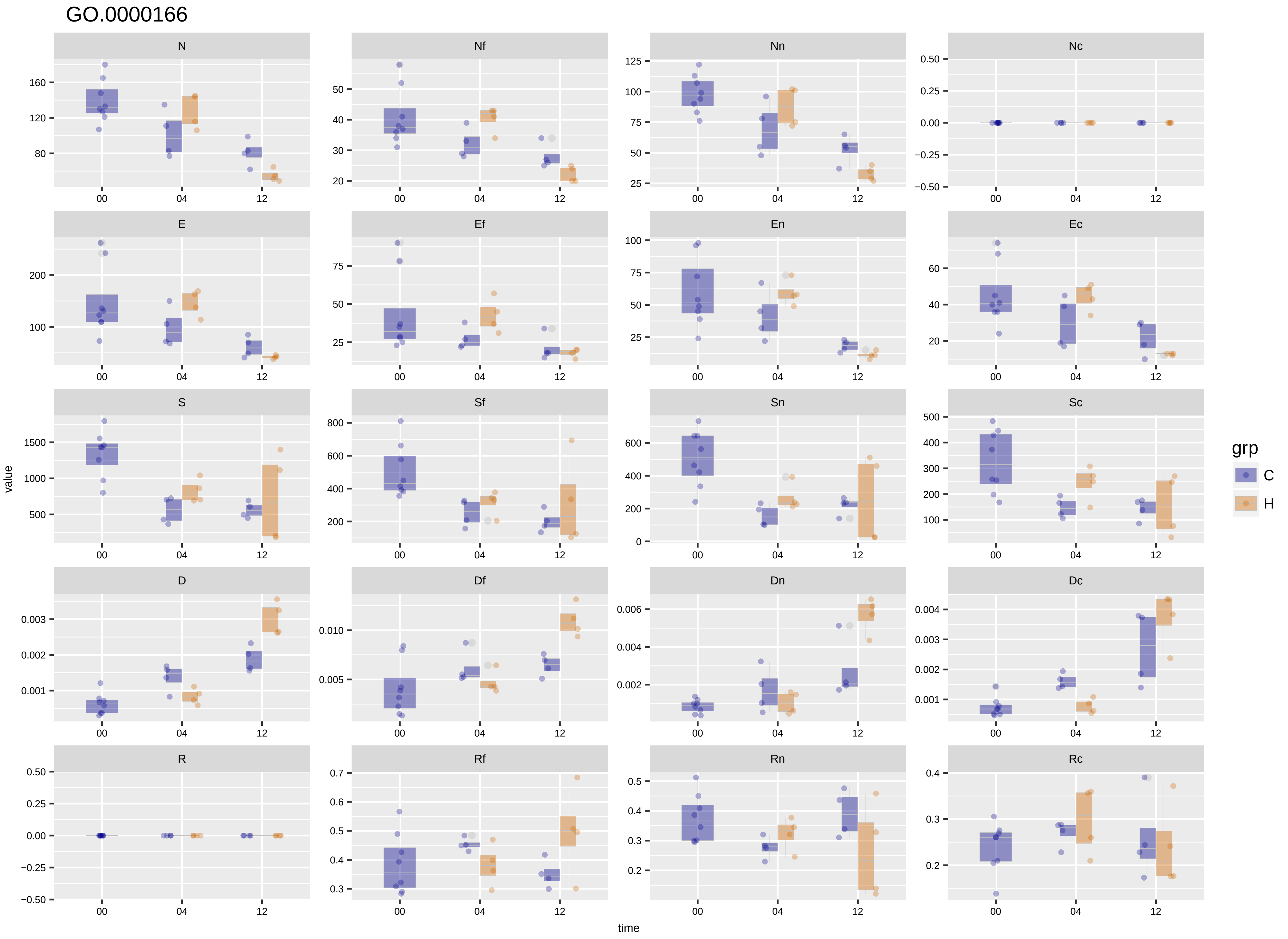

GO.0000166
N
Nf
Nn
Nc
E
Ef
En
Ec
S
Sf
Sn
Sc
D
Df
Dn
Dc
R
Rf
Rn
Rc
value
time
grp
C
H
00
04
12

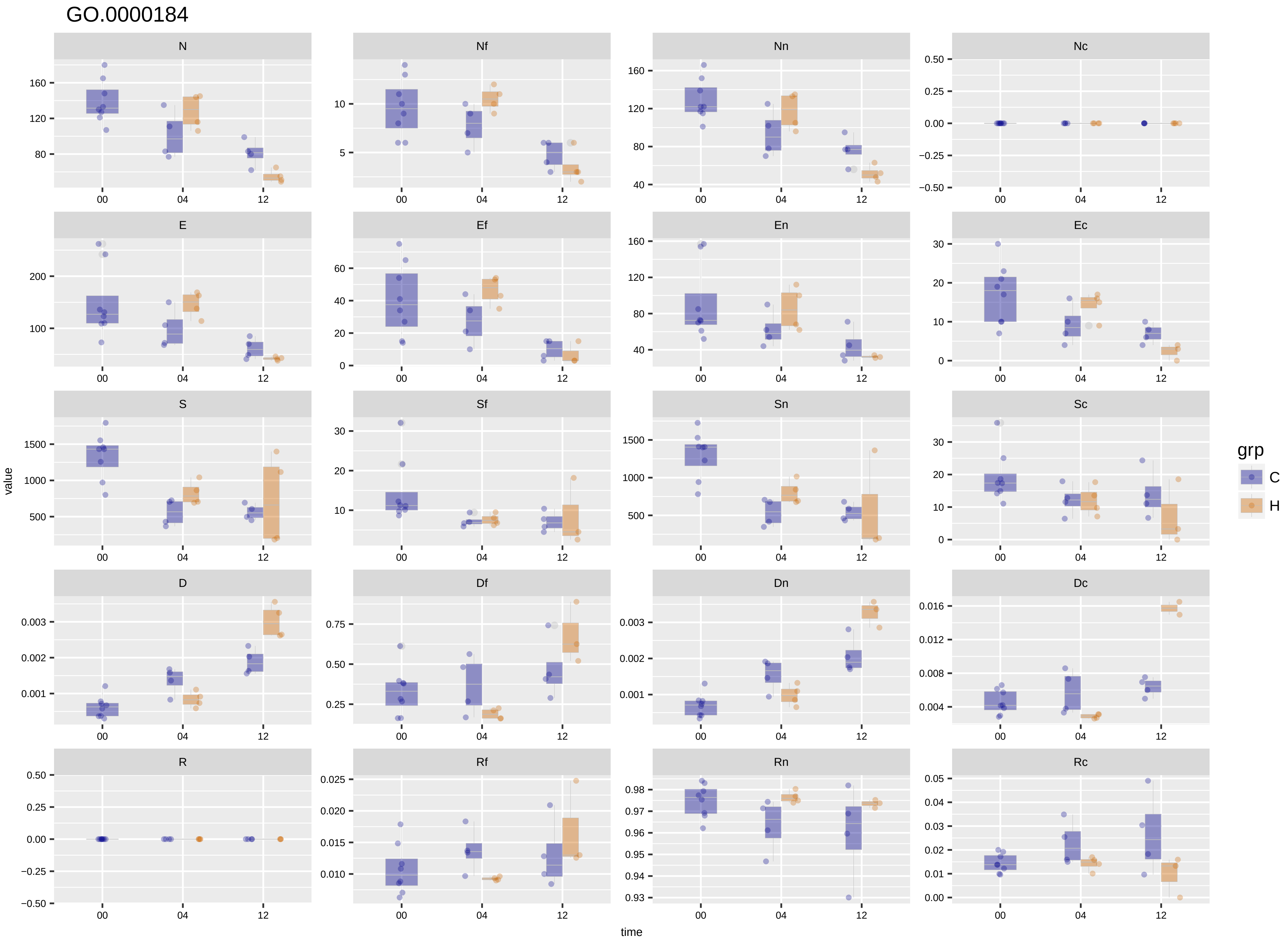

GO.0000184
N
Nf
Nn
Nc
E
Ef
En
Ec
S
Sf
Sn
Sc
D
Df
Dn
Dc
R
Rf
Rn
Rc
00
04
12
value
time
grp
C
H

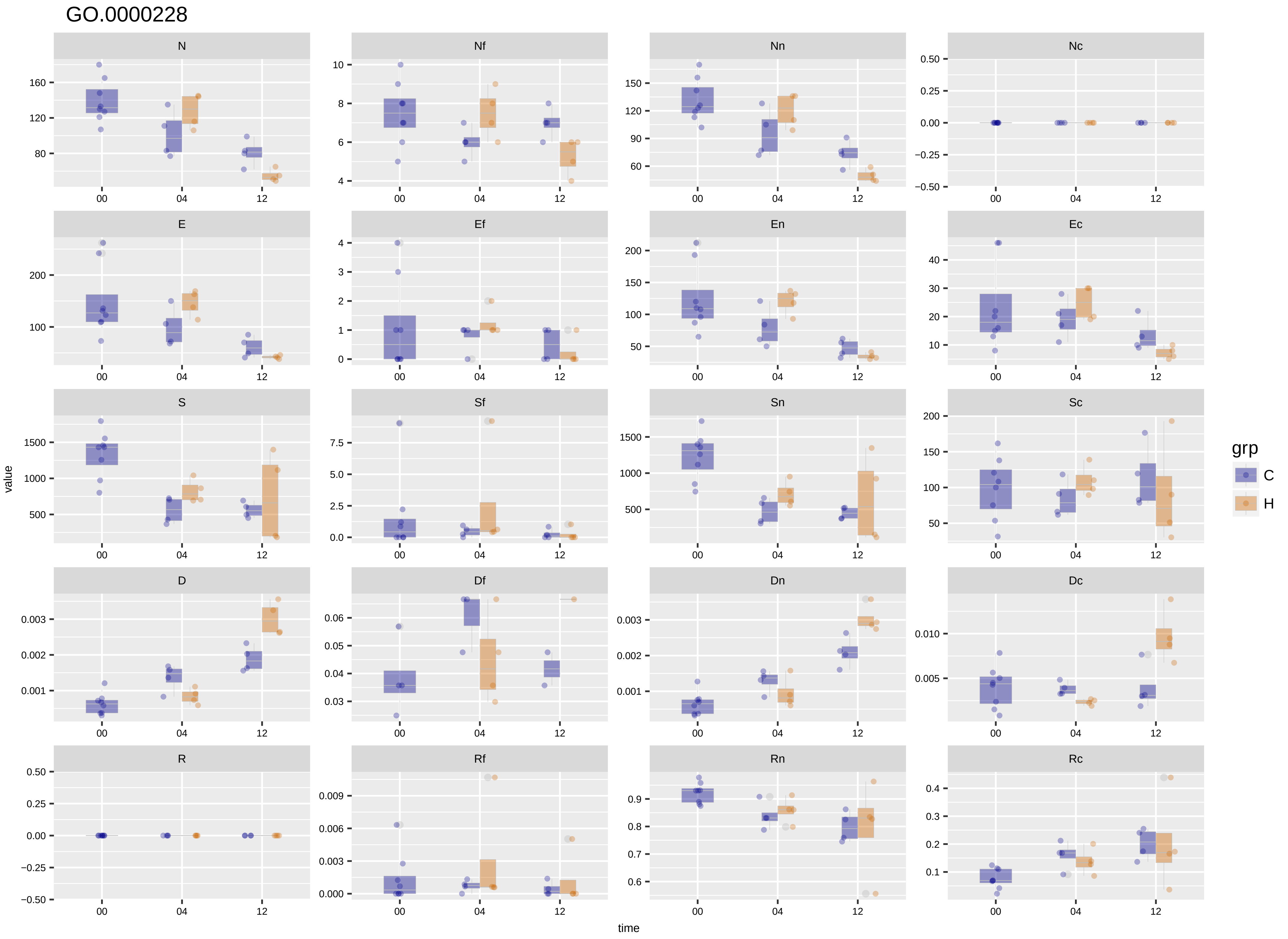
GO.0000228
N
Nf
Nn
Nc
E
Ef
En
Ec
S
Sf
Sn
Sc
D
Df
Dn
Dc
R
Rf
Rn
Rc
value
time
grp
C
H

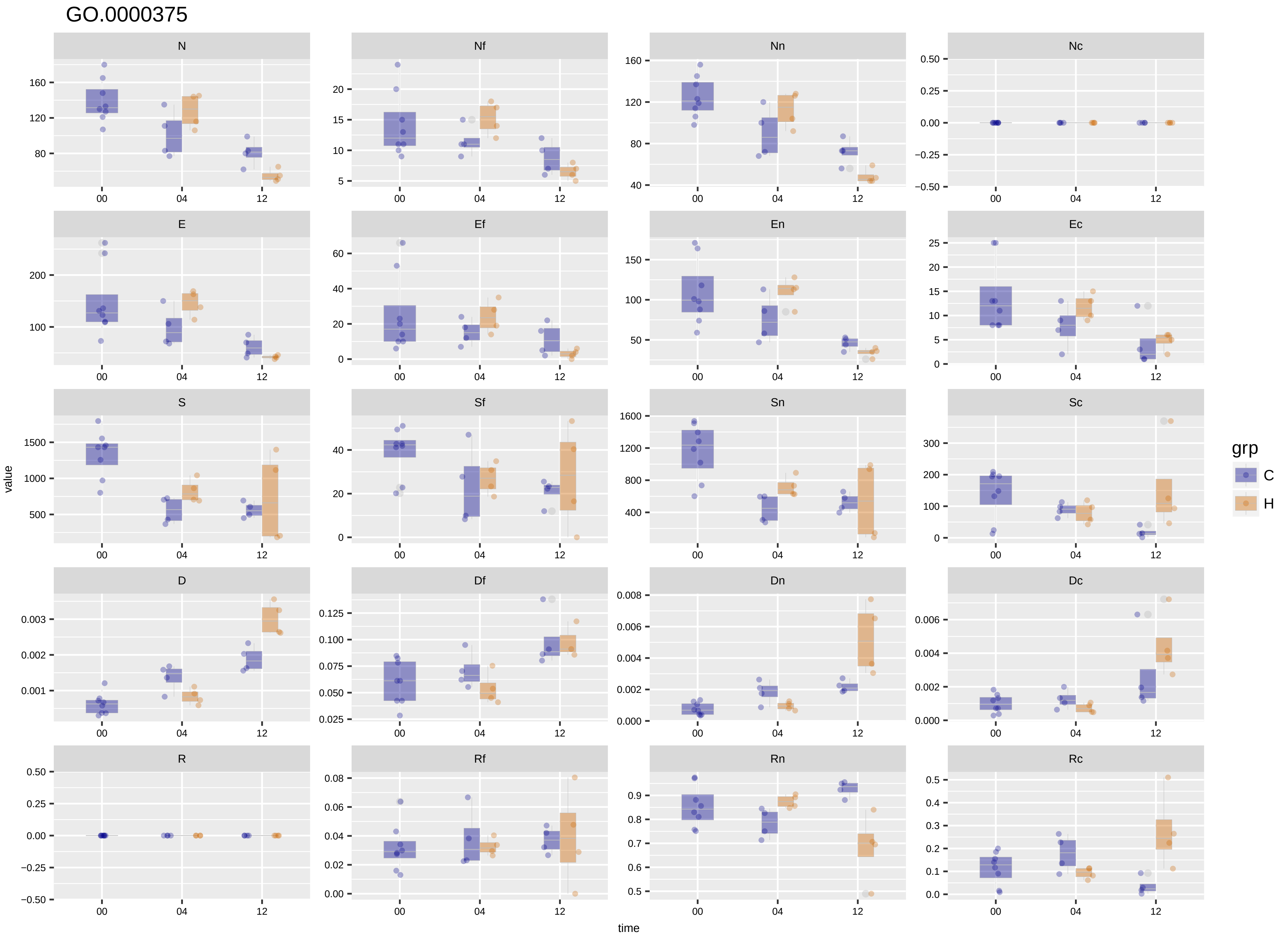
GO.0000375
N
Nf
Nn
Nc
E
Ef
En
Ec
S
Sf
Sn
Sc
D
Df
Dn
Dc
R
Rf
Rn
Rc
value
time
grp
C
H

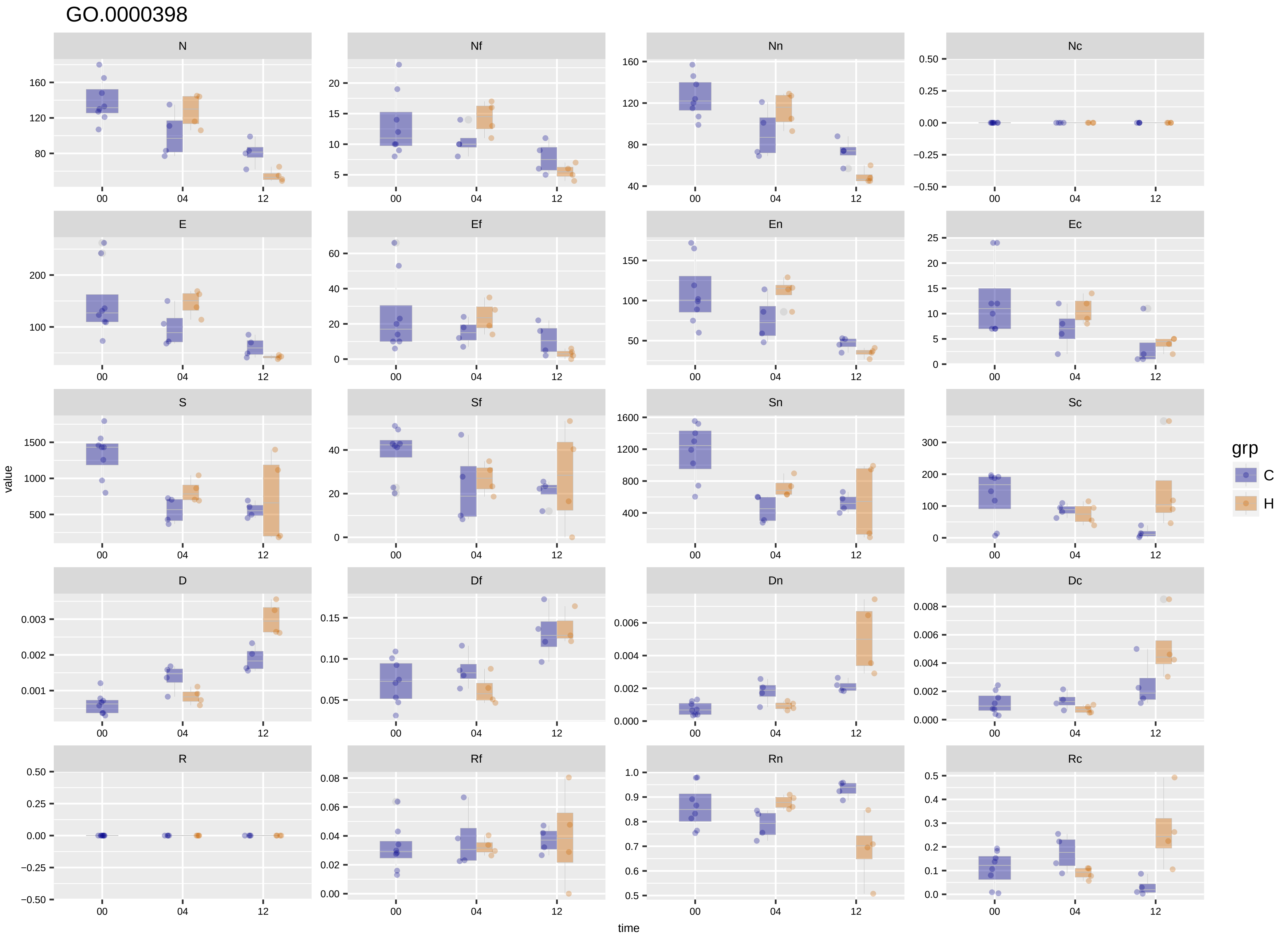
GO.0000398
N
Nf
Nn
Nc
E
Ef
En
Ec
S
Sf
Sn
Sc
D
Df
Dn
Dc
R
Rf
Rn
Rc
00
04
12
value
time
grp
C
H

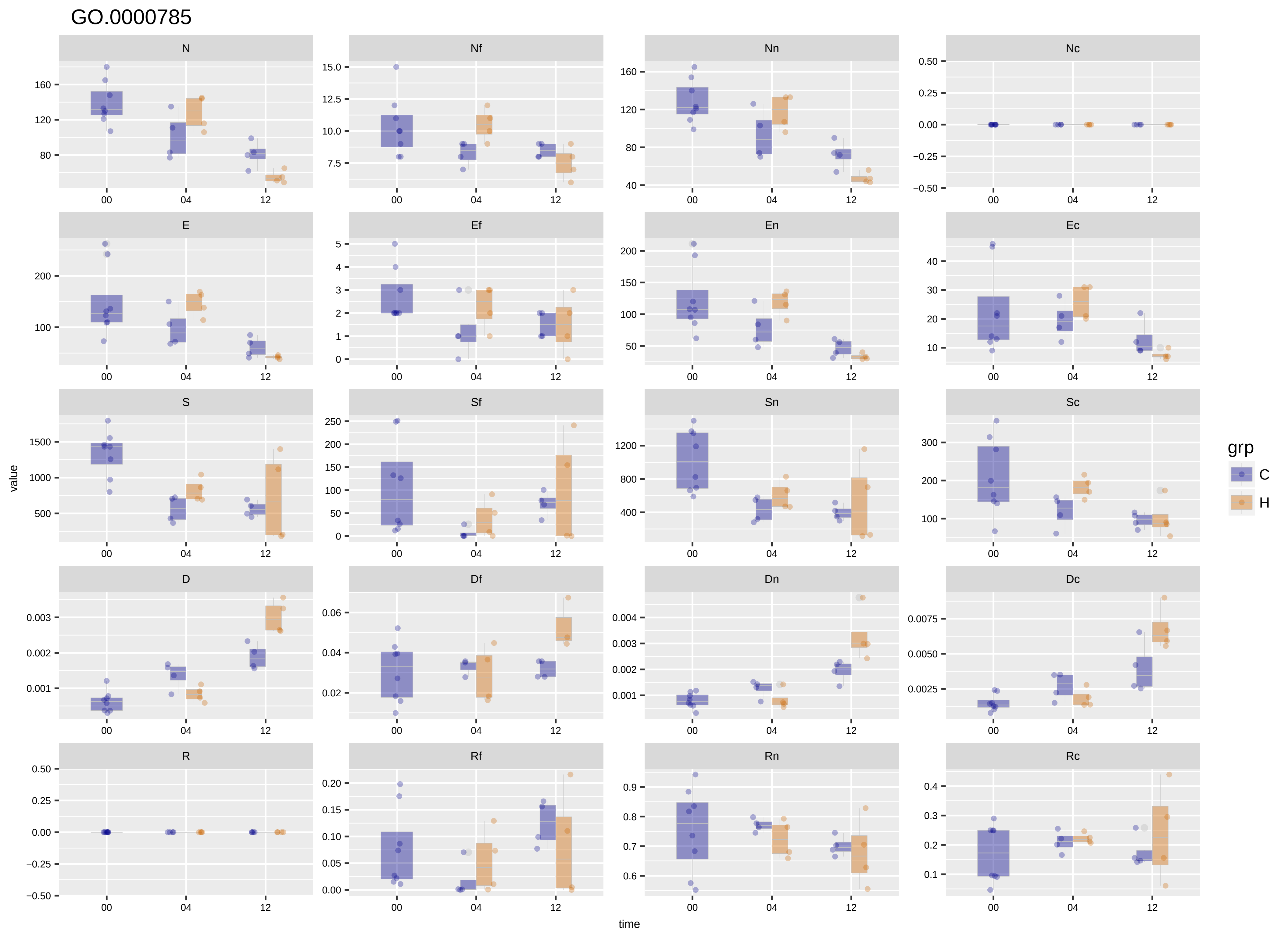
GO.0000785
N
Nf
Nn
Nc
E
Ef
En
Ec
S
Sf
Sn
Sc
D
Df
Dn
Dc
R
Rf
Rn
Rc
00
04
12
value
time
grp
C
H

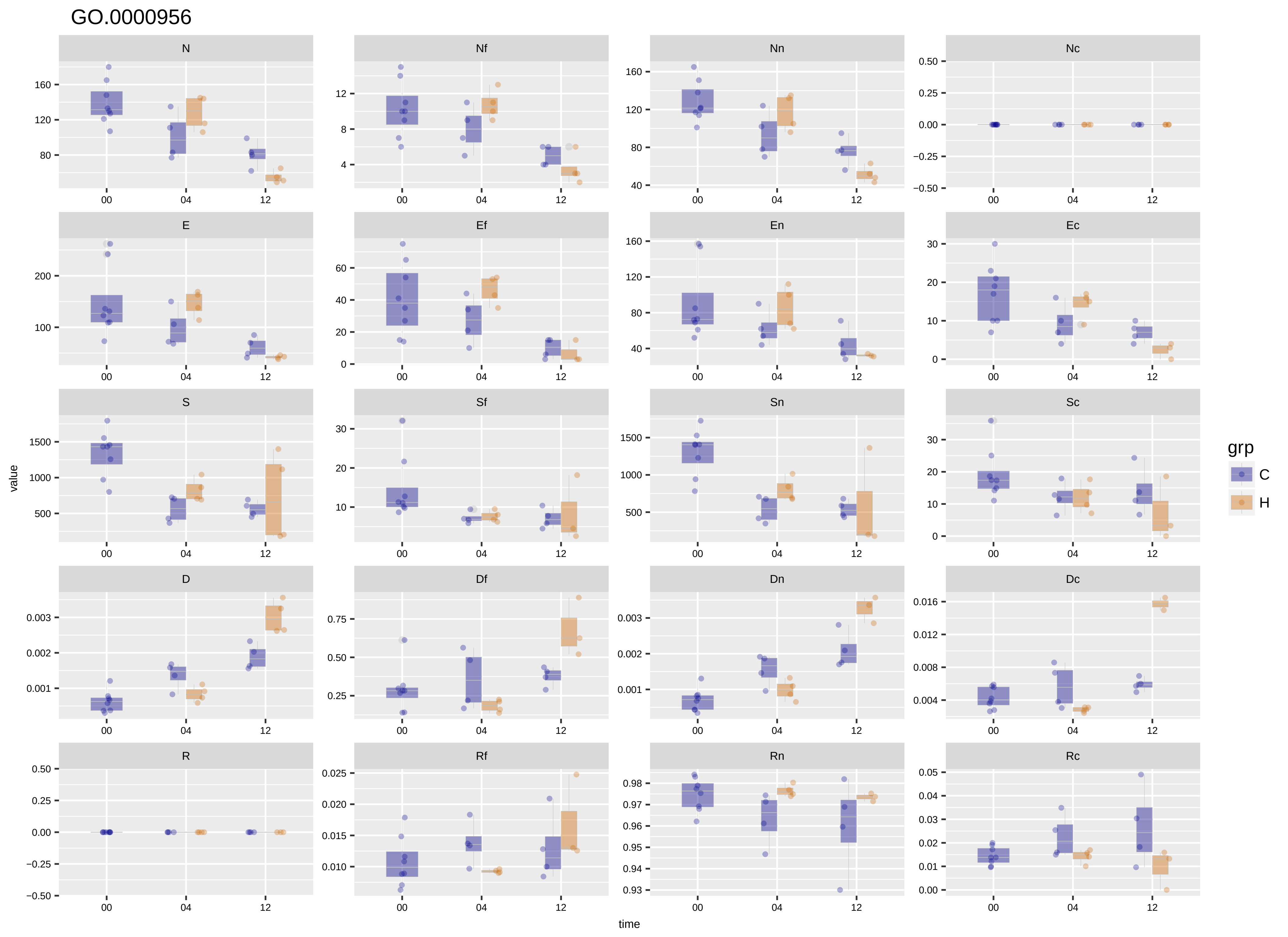
GO.0000956
N
Nf
Nn
Nc
E
Ef
En
Ec
S
Sf
Sn
Sc
D
Df
Dn
Dc
R
Rf
Rn
Rc
value
time
00
04
12
grp
C
H

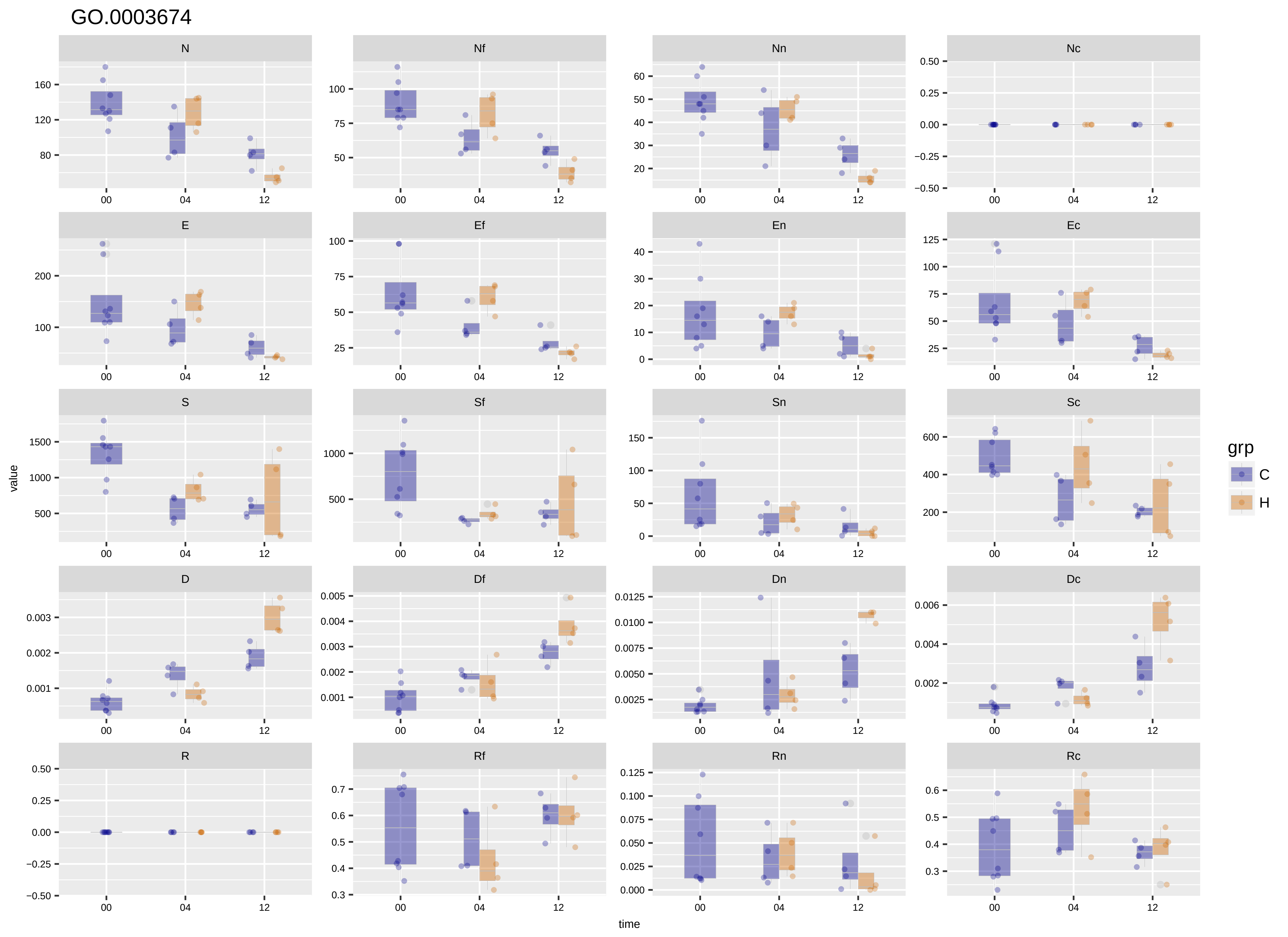

GO.0003674
N
Nf
Nn
Nc
E
Ef
En
Ec
S
Sf
Sn
Sc
D
Df
Dn
Dc
R
Rf
Rn
Rc
value
time
grp
C
H

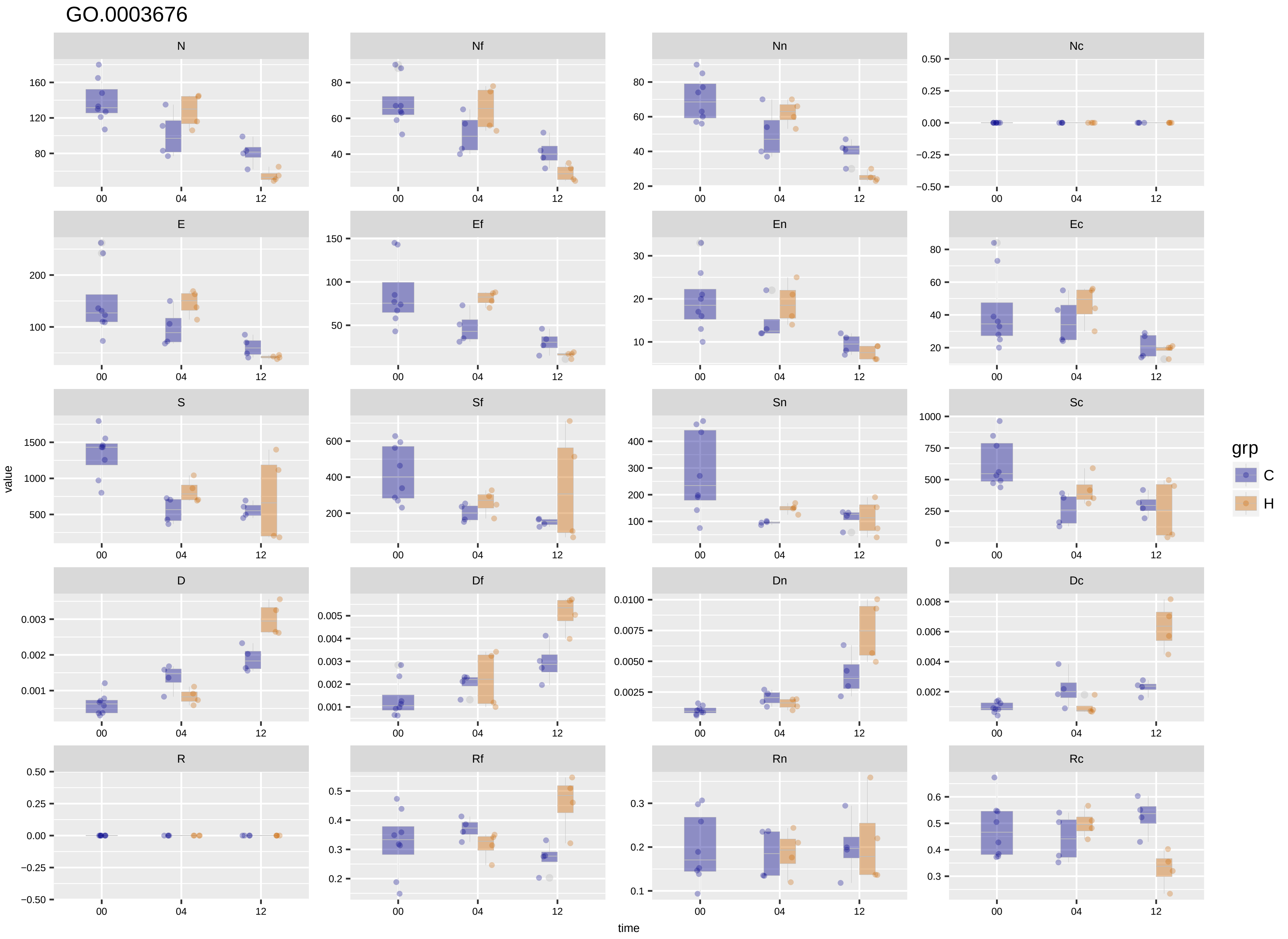
GO.0003676
N
Nf
Nn
Nc
E
Ef
En
Ec
S
Sf
Sn
Sc
D
Df
Dn
Dc
R
Rf
Rn
Rc
value
time
00
04
12
grp
C
H

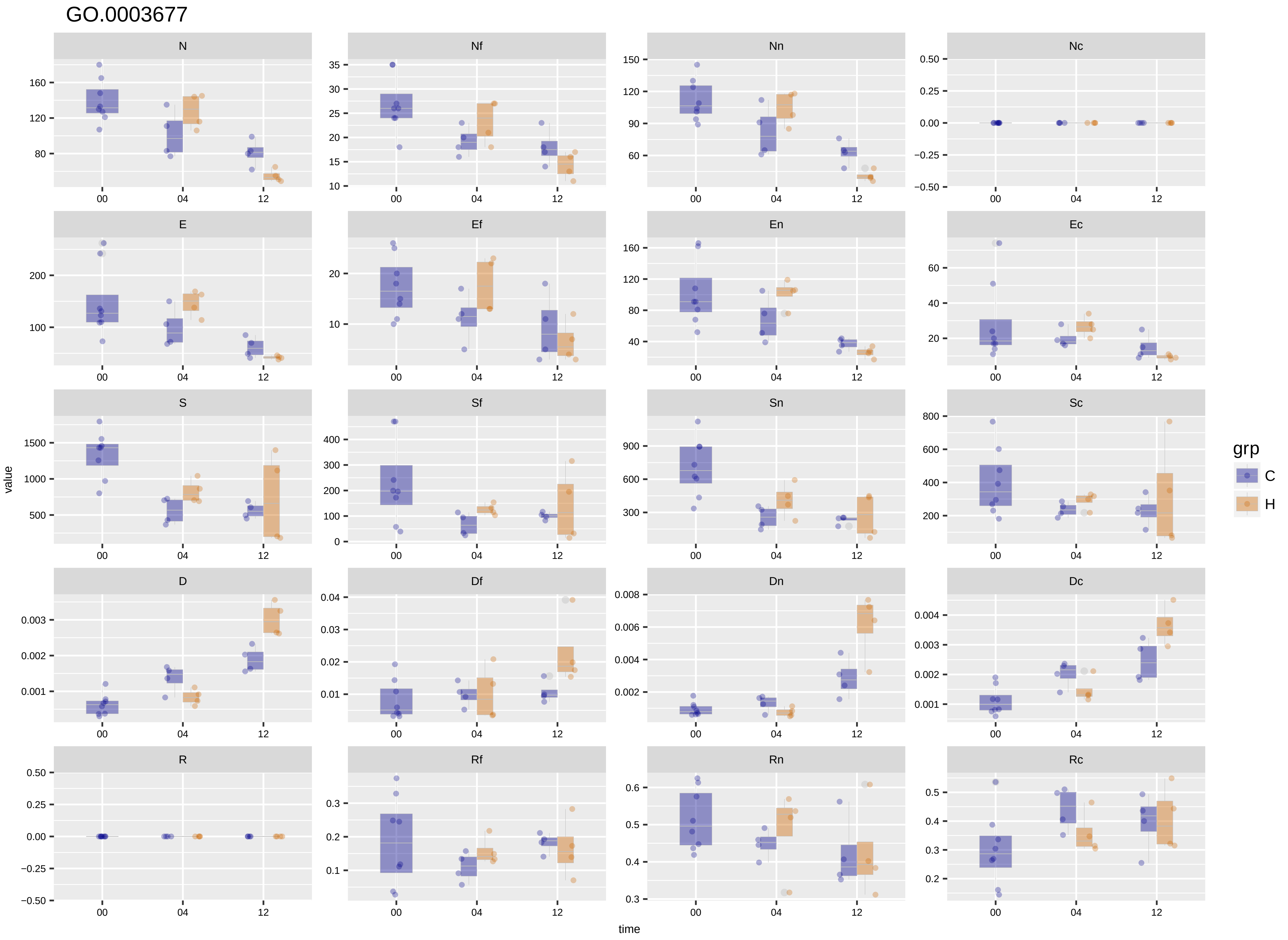

GO.0003677
N
Nf
Nn
Nc
E
Ef
En
Ec
S
Sf
Sn
Sc
D
Df
Dn
Dc
R
Rf
Rn
Rc
value
time
00
04
12
grp
C
H

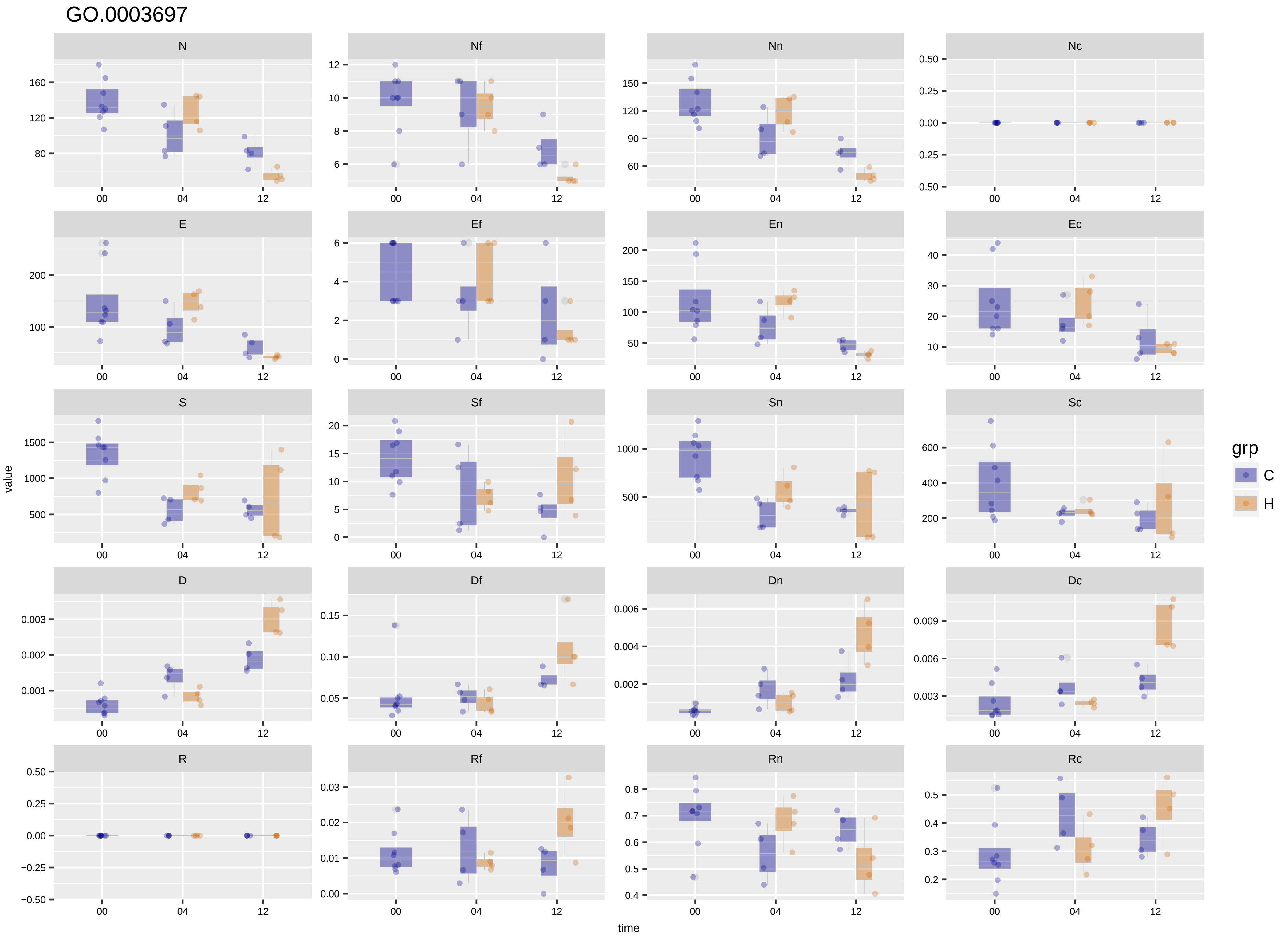
GO.0003697
N
Nf
Nn
Nc
E
Ef
En
Ec
S
Sf
Sn
Sc
D
Df
Dn
Dc
R
Rf
Rn
Rc
00
04
12
value
time
grp
C
H

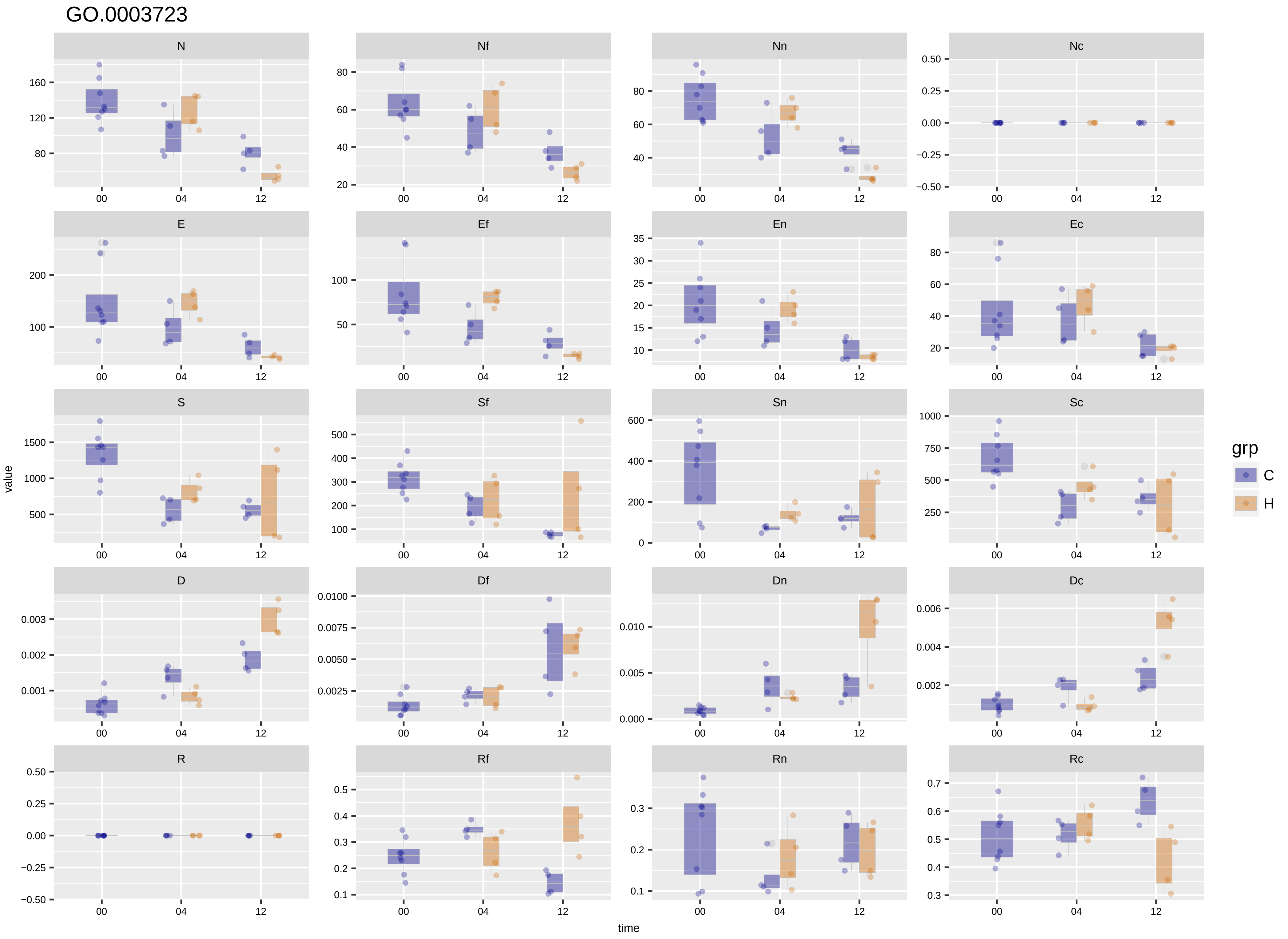
GO.0003723
N
Nf
Nn
Nc
E
Ef
En
Ec
S
Sf
Sn
Sc
D
Df
Dn
Dc
R
Rf
Rn
Rc
value
time
00
04
12
grp
C
H

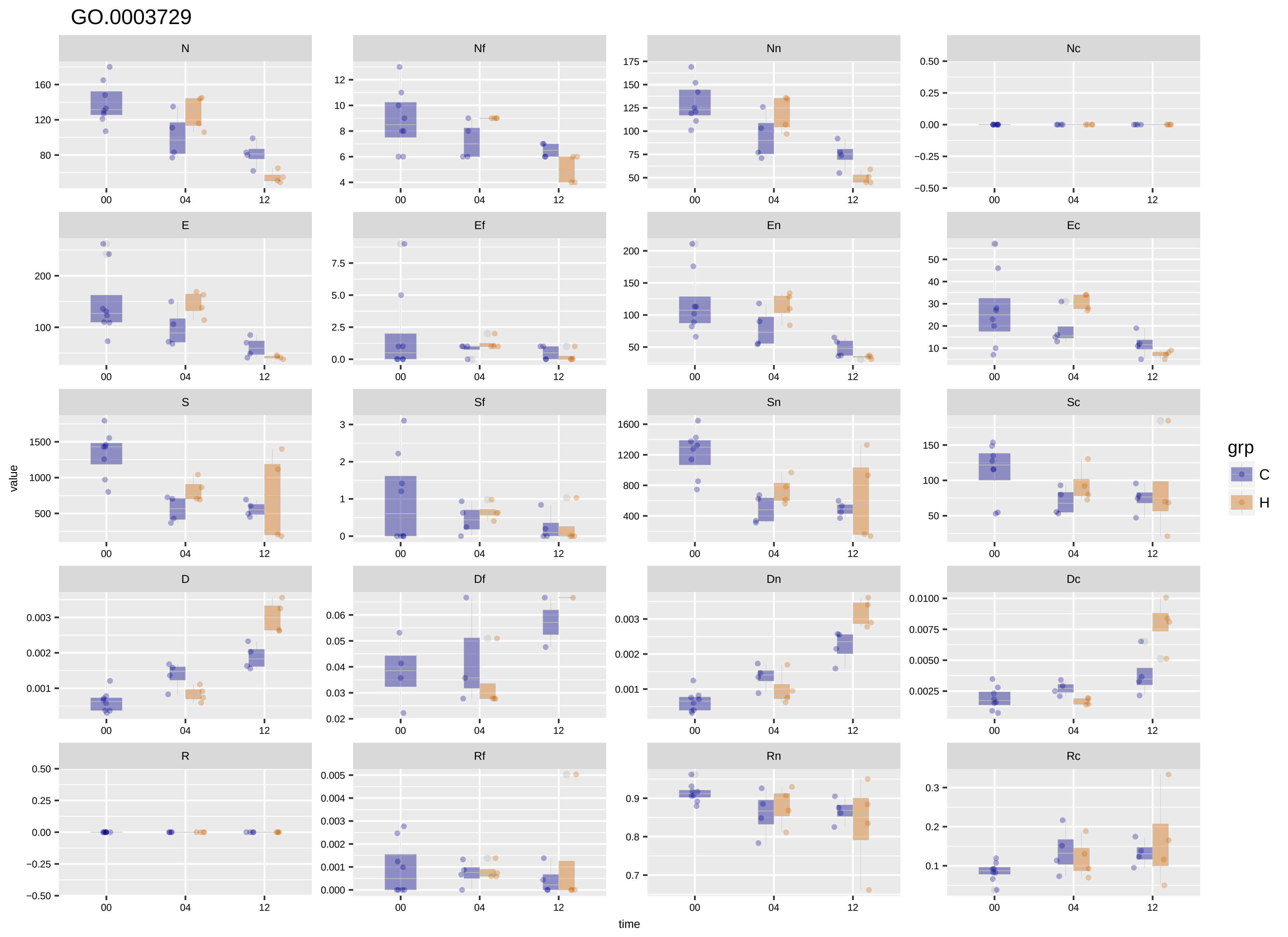

GO.0003729
N
Nf
Nn
Nc
E
Ef
En
Ec
S
Sf
Sn
Sc
D
Df
Dn
Dc
R
Rf
Rn
Rc
value
time
00
04
12
grp
C
H

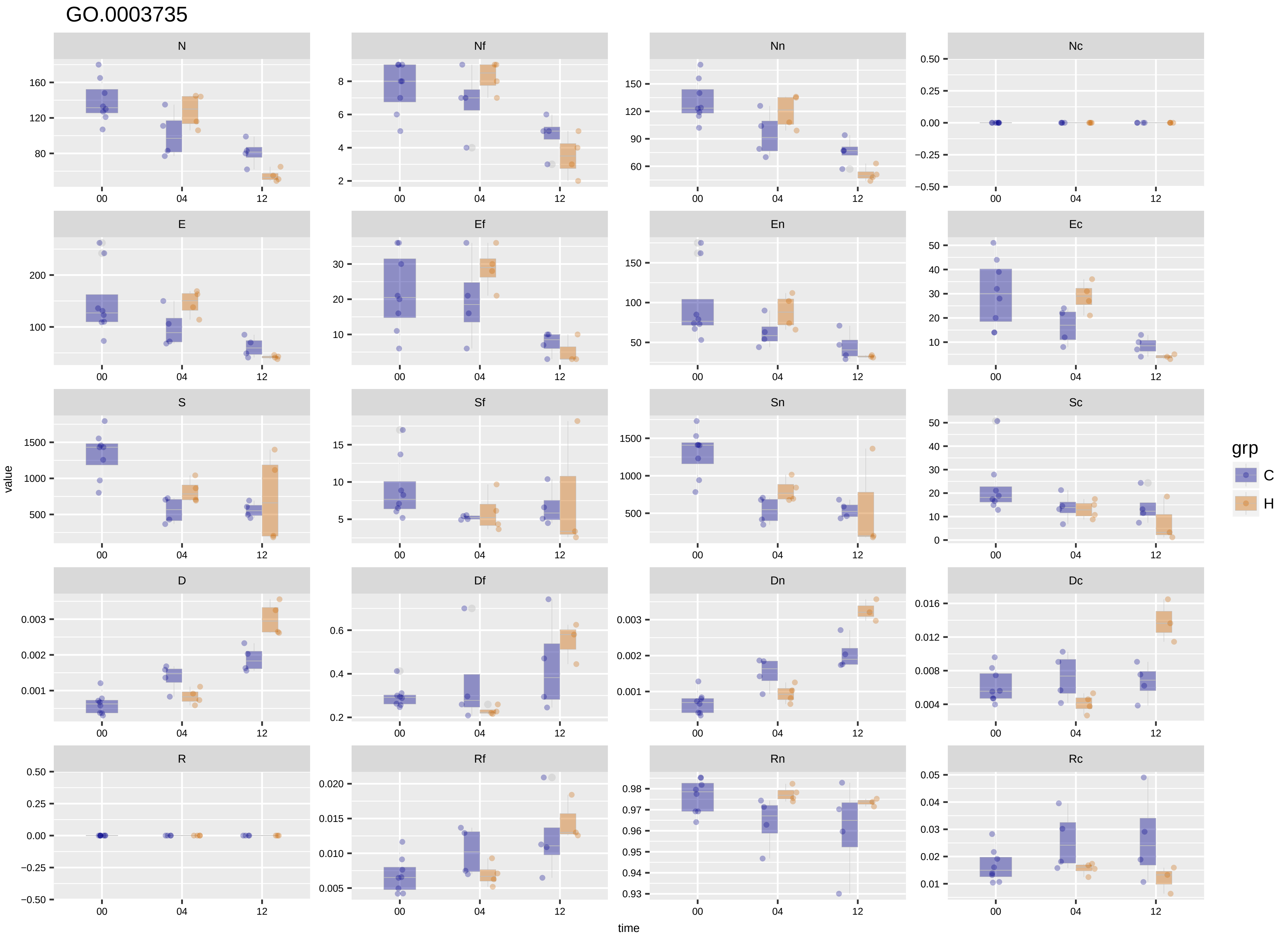

GO.0003735
N
Nf
Nn
Nc
E
Ef
En
Ec
S
Sf
Sn
Sc
D
Df
Dn
Dc
R
Rf
Rn
Rc
value
time
grp
C
H
00
04
12

GO.0005198

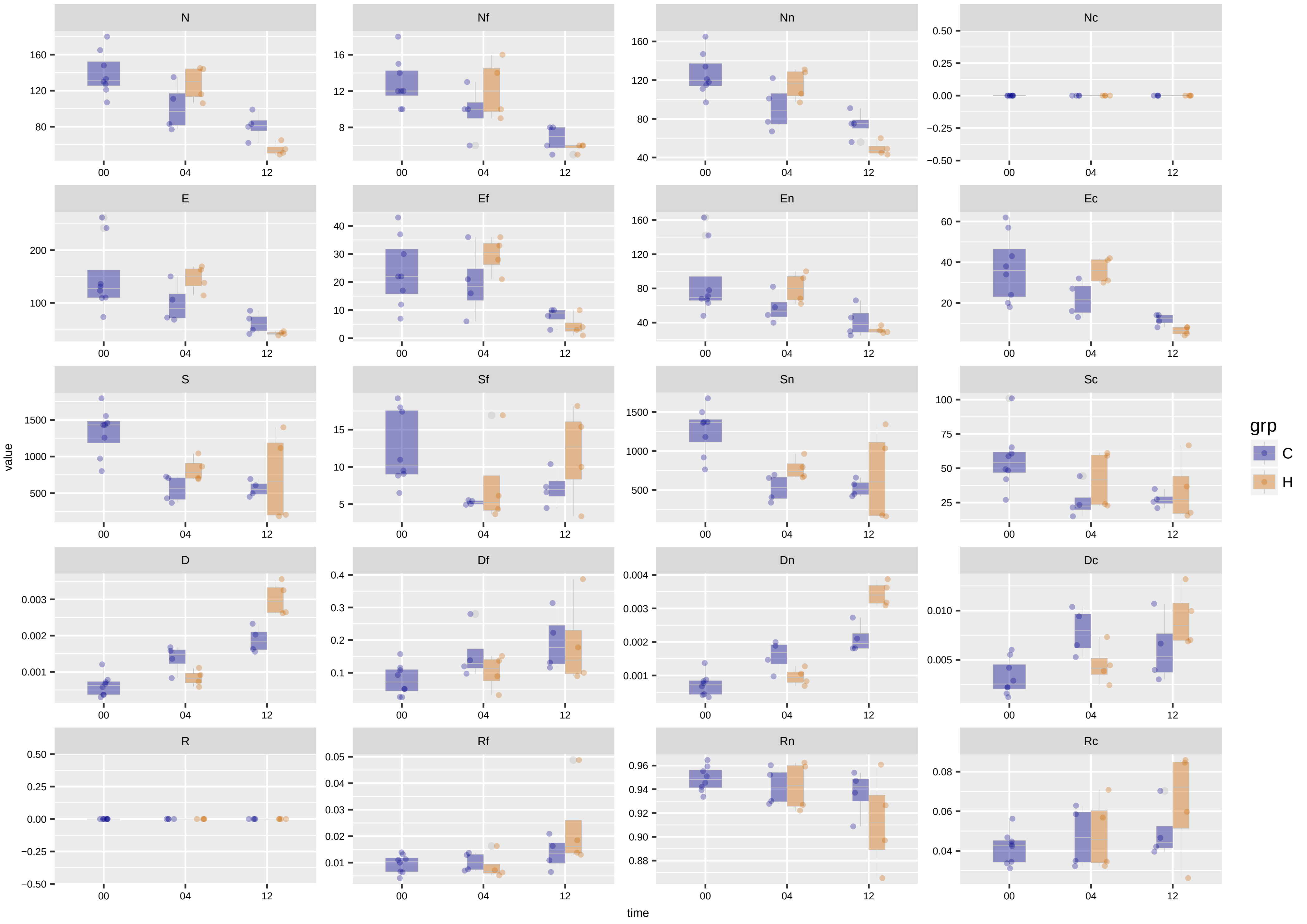

N
Nf
Nn
Nc
E
Ef
En
Ec
S
Sf
Sn
Sc
D
Df
Dn
Dc
R
Rf
Rn
Rc
00
04
12
value
time
grp
C
H

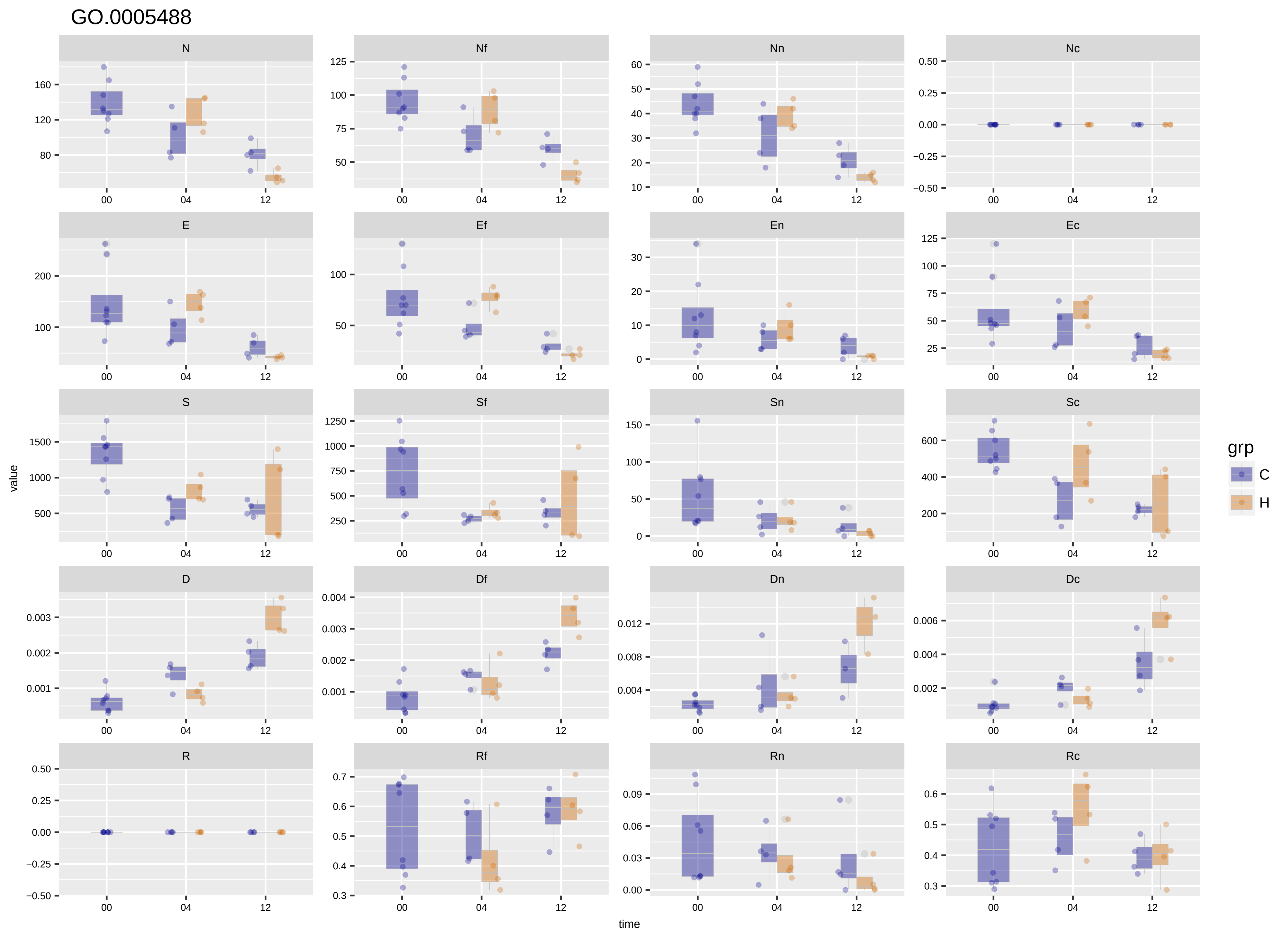

GO.0005488
N
Nf
Nn
Nc
E
Ef
En
Ec
S
Sf
Sn
Sc
D
Df
Dn
Dc
R
Rf
Rn
Rc
00
04
12
value
time
grp
C
H

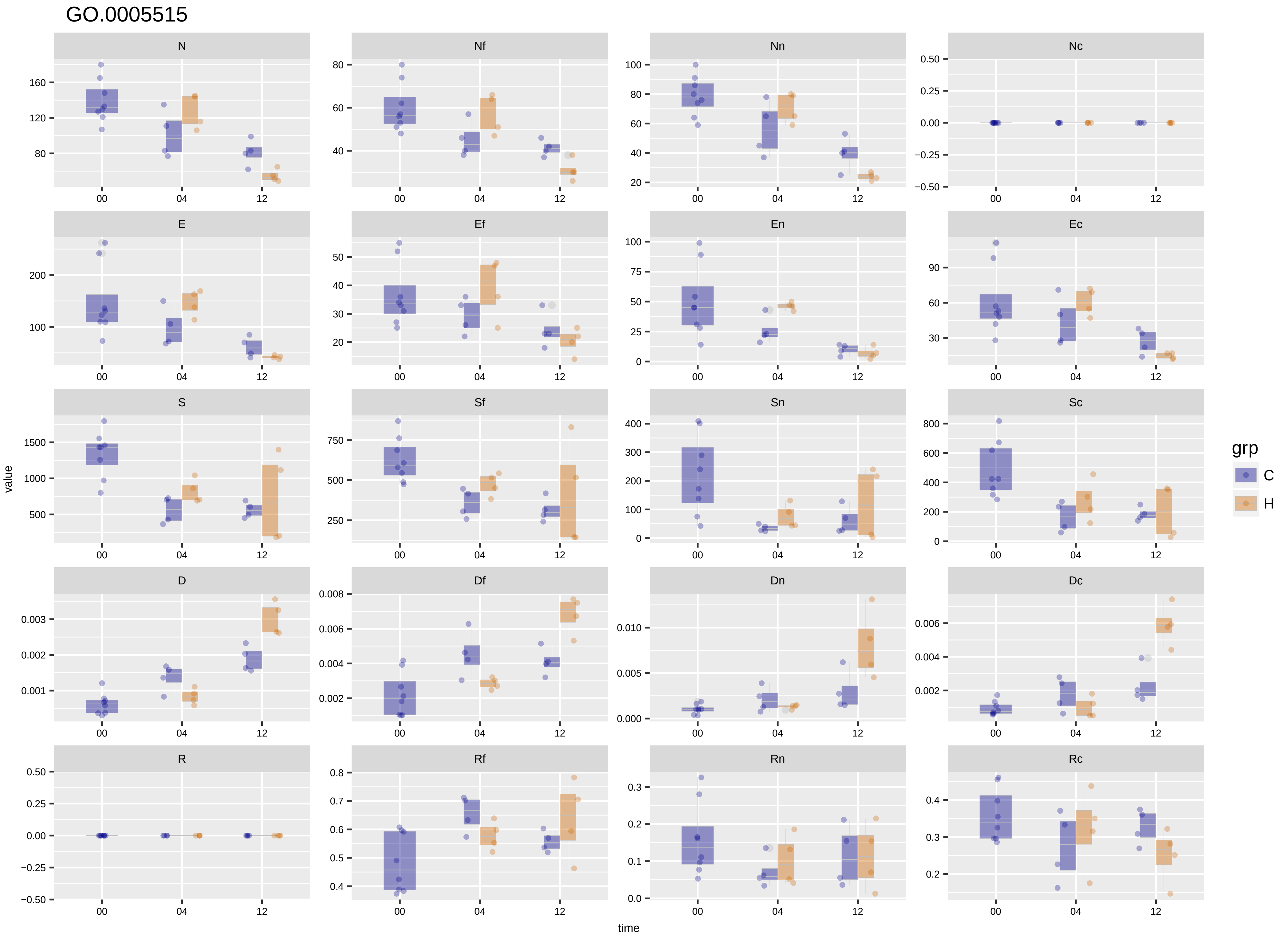
GO.0005515
N
Nf
Nn
Nc
E
Ef
En
Ec
S
Sf
Sn
Sc
D
Df
Dn
Dc
R
Rf
Rn
Rc
value
time
grp
C
H

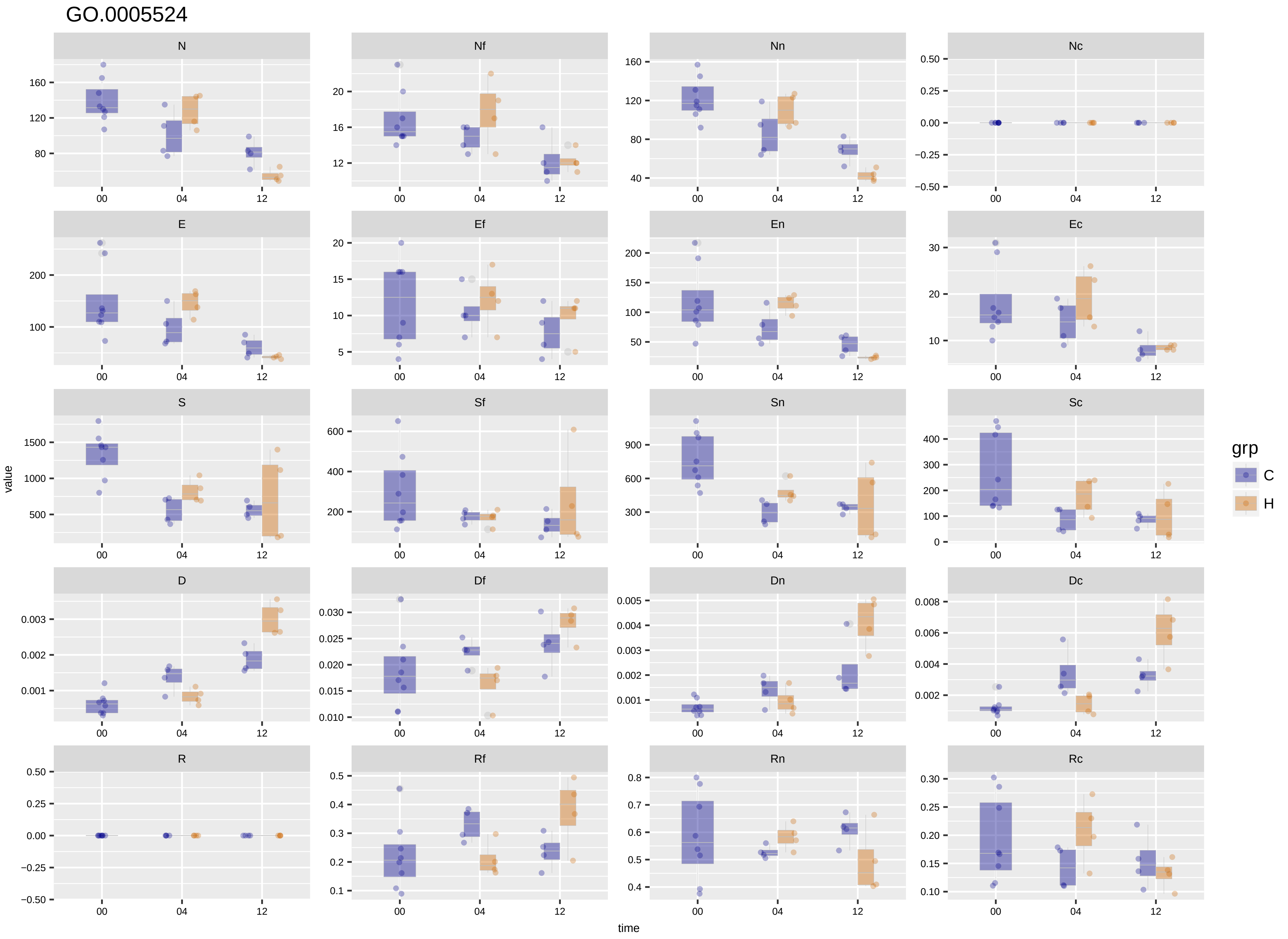
GO.0005524
N
Nf
Nn
Nc
E
Ef
En
Ec
S
Sf
Sn
Sc
D
Df
Dn
Dc
R
Rf
Rn
Rc
value
time
grp
C
H

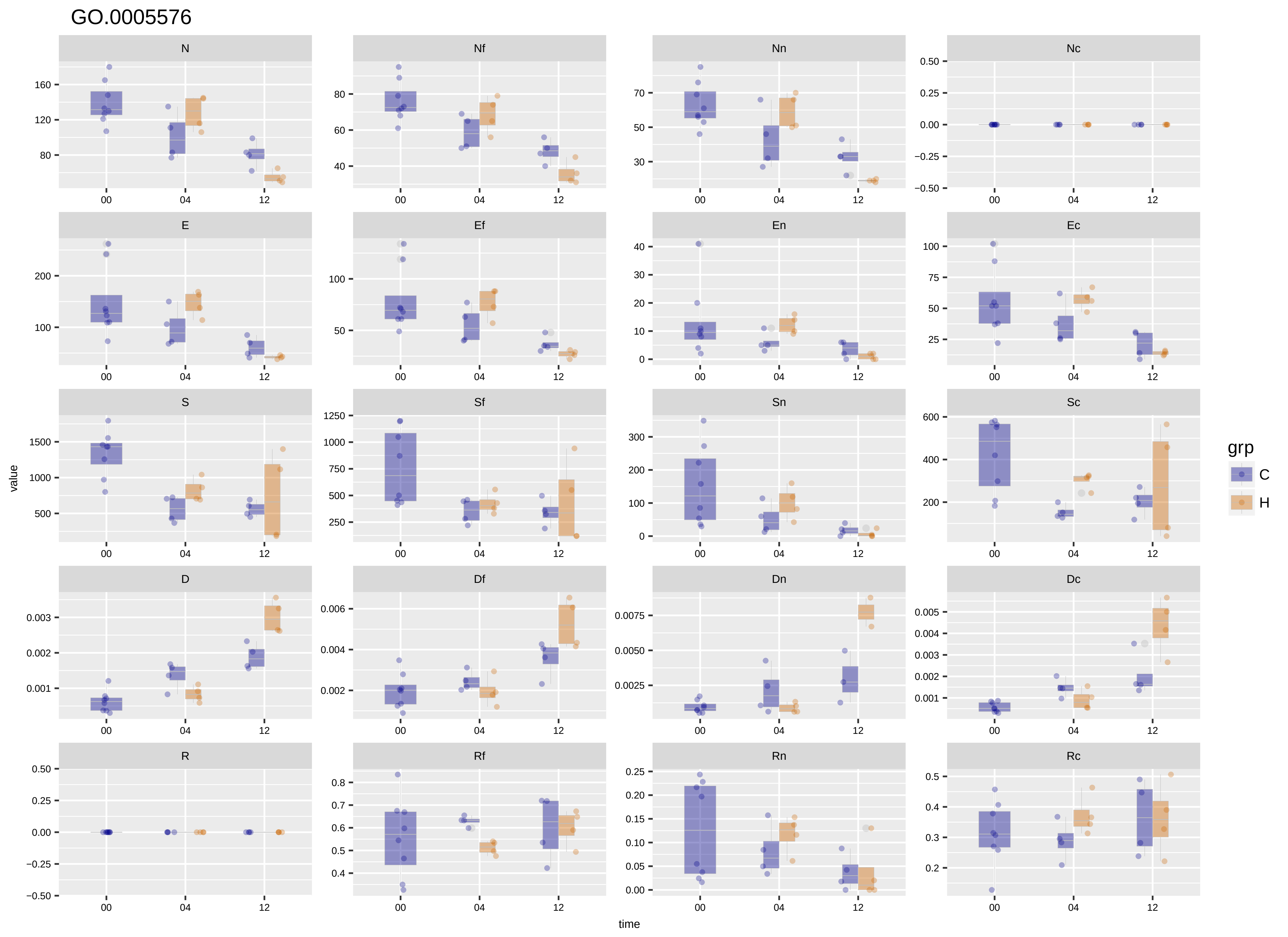
GO.0005576
N
Nf
Nn
Nc
E
Ef
En
Ec
S
Sf
Sn
Sc
D
Df
Dn
Dc
R
Rf
Rn
Rc
value
time
grp
C
H
00
04
12

GO.0005615

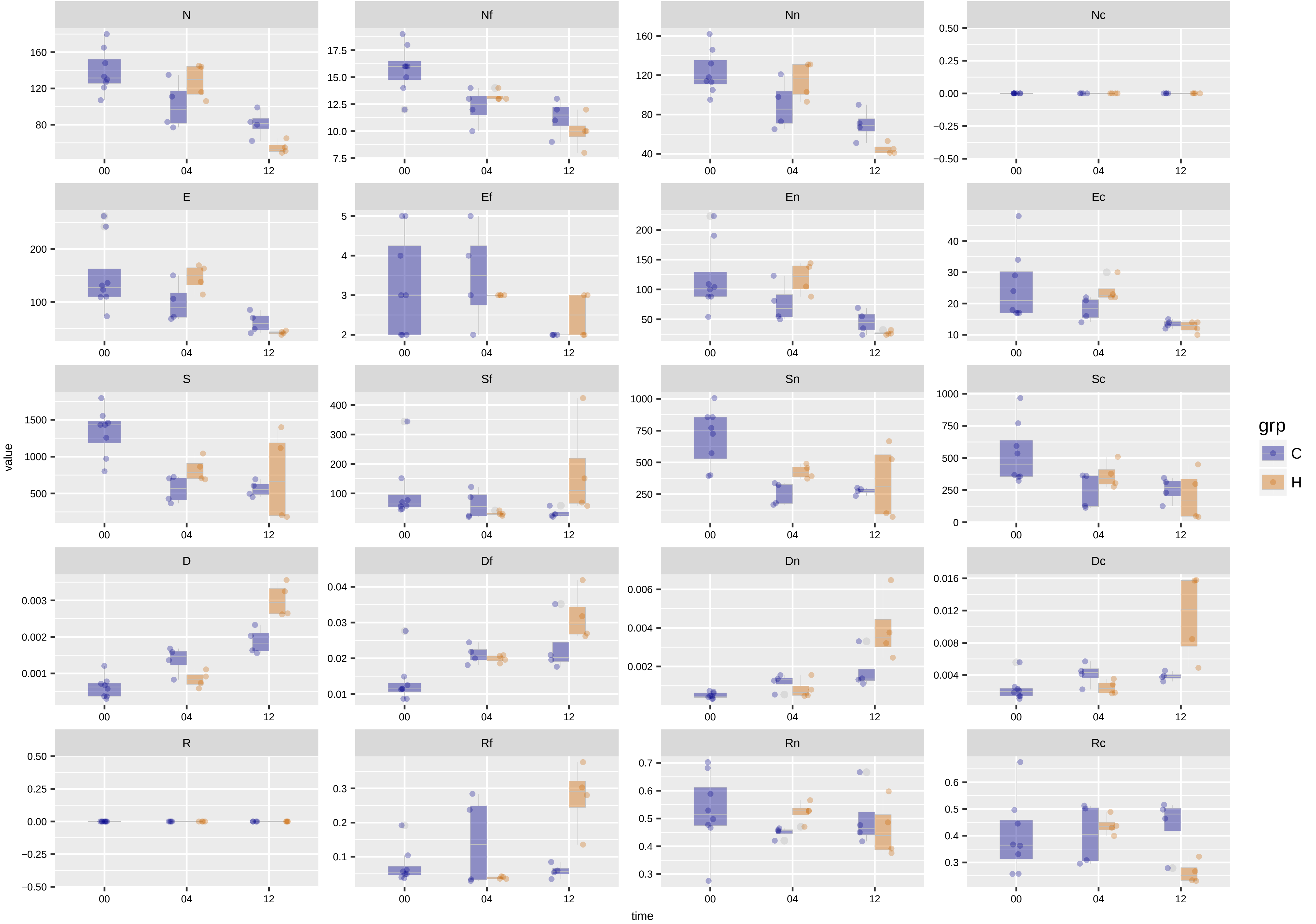

N
Nf
Nn
Nc
E
Ef
En
Ec
S
Sf
Sn
Sc
D
Df
Dn
Dc
R
Rf
Rn
Rc
00
04
12
value
time
grp
C
H

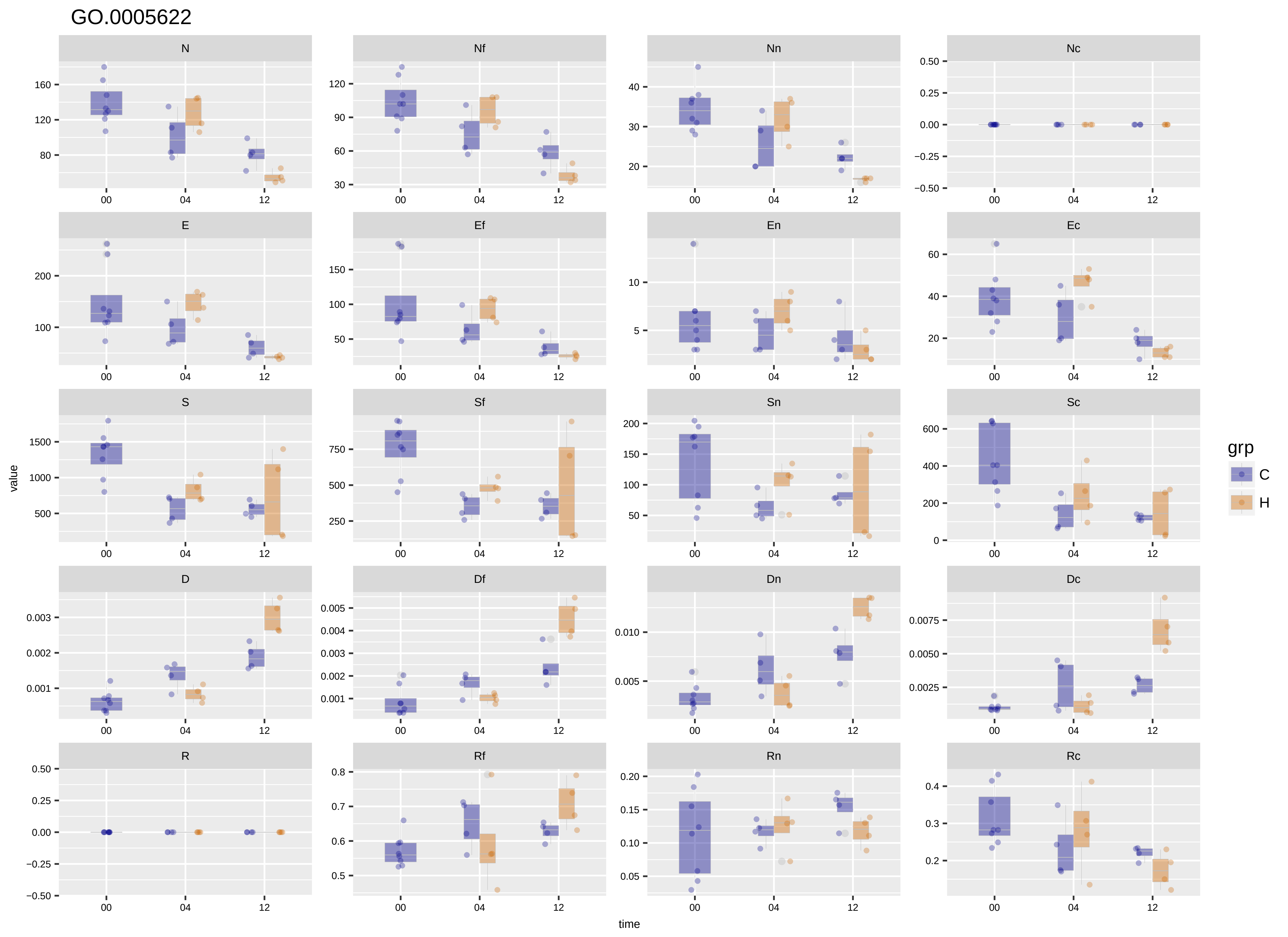
GO.0005622
N
Nf
Nn
Nc
E
Ef
En
Ec
S
Sf
Sn
Sc
D
Df
Dn
Dc
R
Rf
Rn
Rc
value
time
grp
C
H

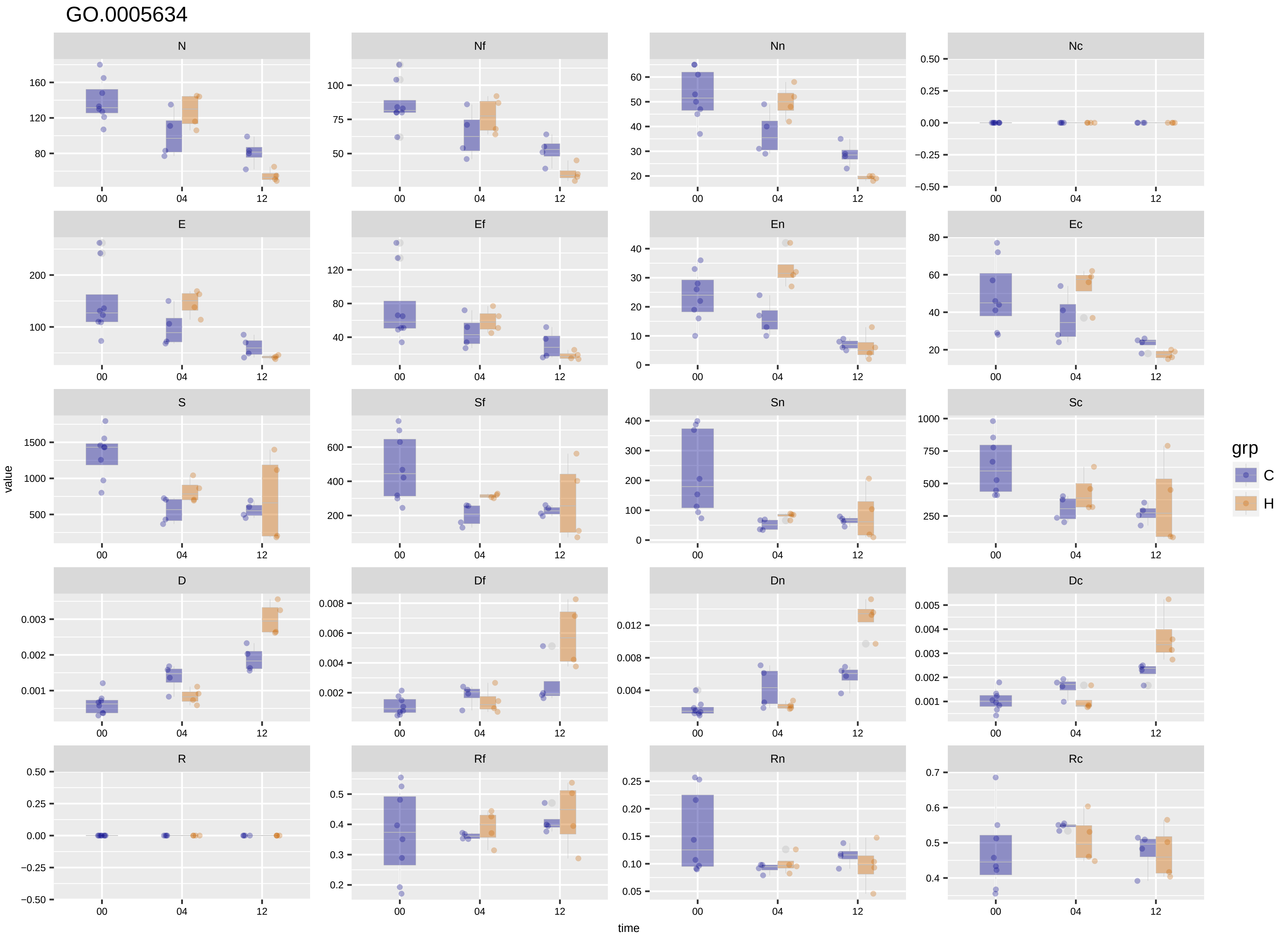
GO.0005634
N
Nf
Nn
Nc
E
Ef
En
Ec
S
Sf
Sn
Sc
D
Df
Dn
Dc
R
Rf
Rn
Rc
value
time
00
04
12
grp
C
H

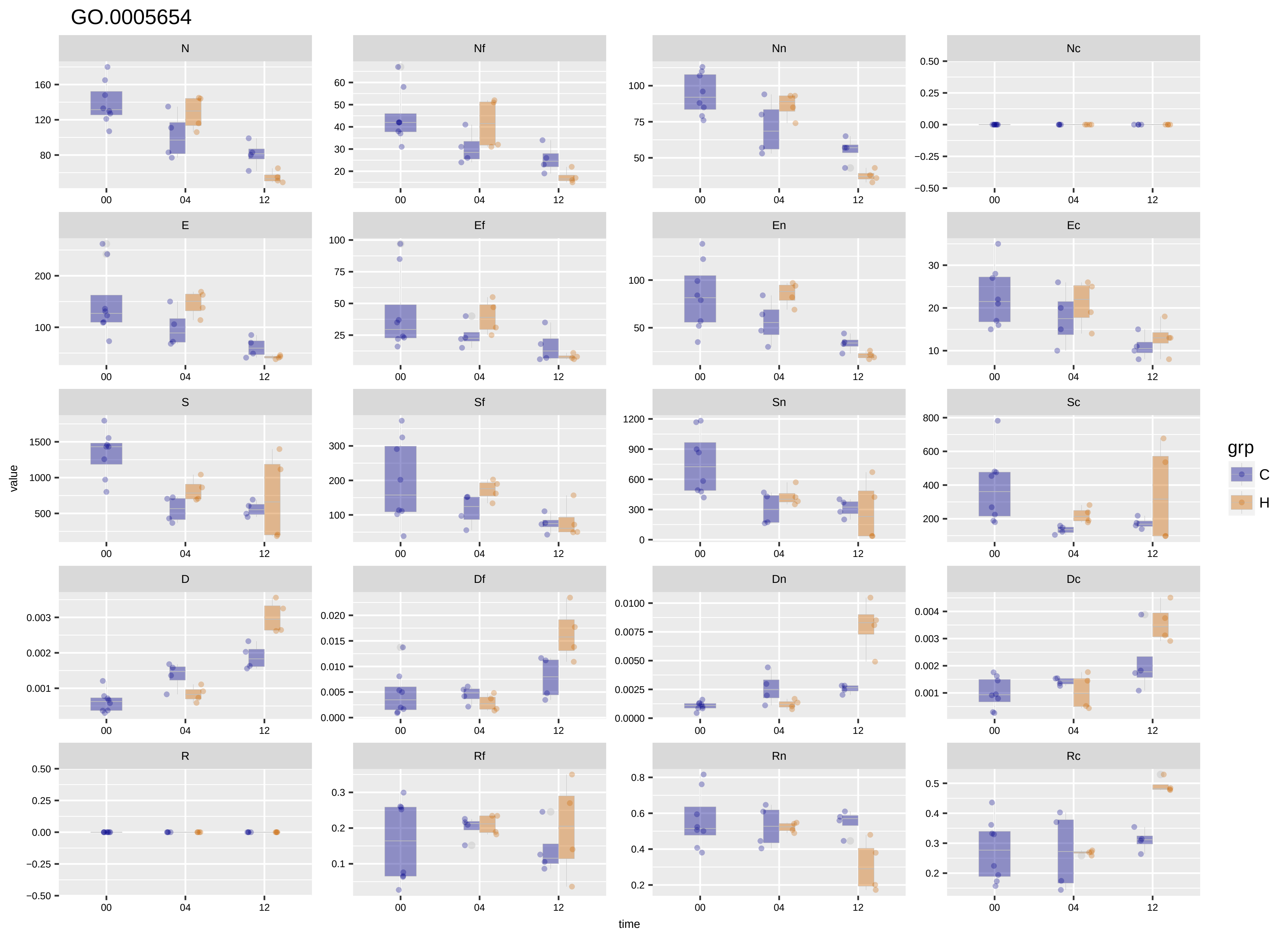
GO.0005654
N
Nf
Nn
Nc
E
Ef
En
Ec
S
Sf
Sn
Sc
D
Df
Dn
Dc
R
Rf
Rn
Rc
value
time
grp
C
H

GO.0005681

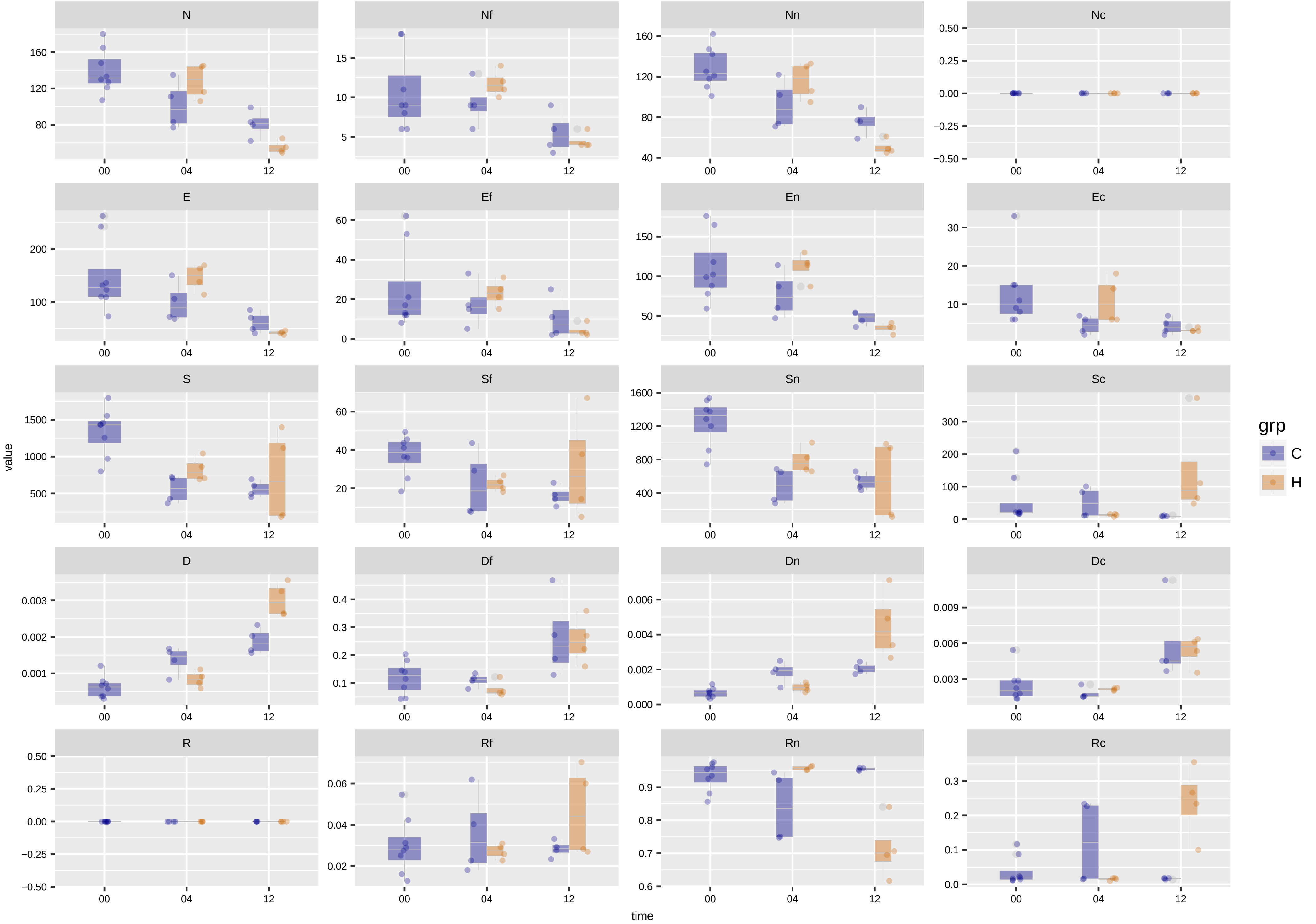

N
Nf
Nn
Nc
E
Ef
En
Ec
S
Sf
Sn
Sc
D
Df
Dn
Dc
R
Rf
Rn
Rc
value
time
grp
C
H
00
04
12

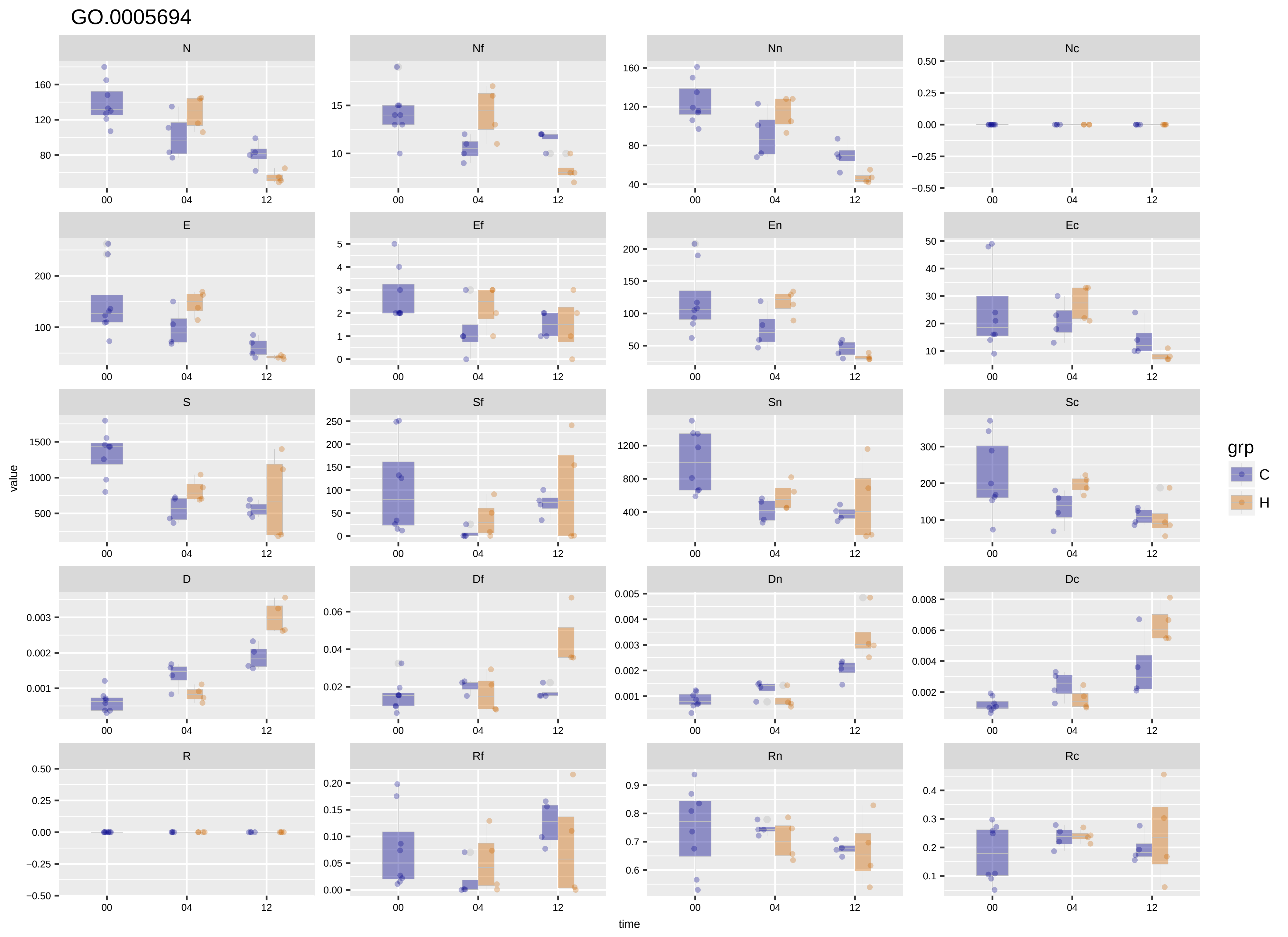
GO.0005694
N
Nf
Nn
Nc
E
Ef
En
Ec
S
Sf
Sn
Sc
D
Df
Dn
Dc
R
Rf
Rn
Rc
00
04
12
value
time
grp
C
H

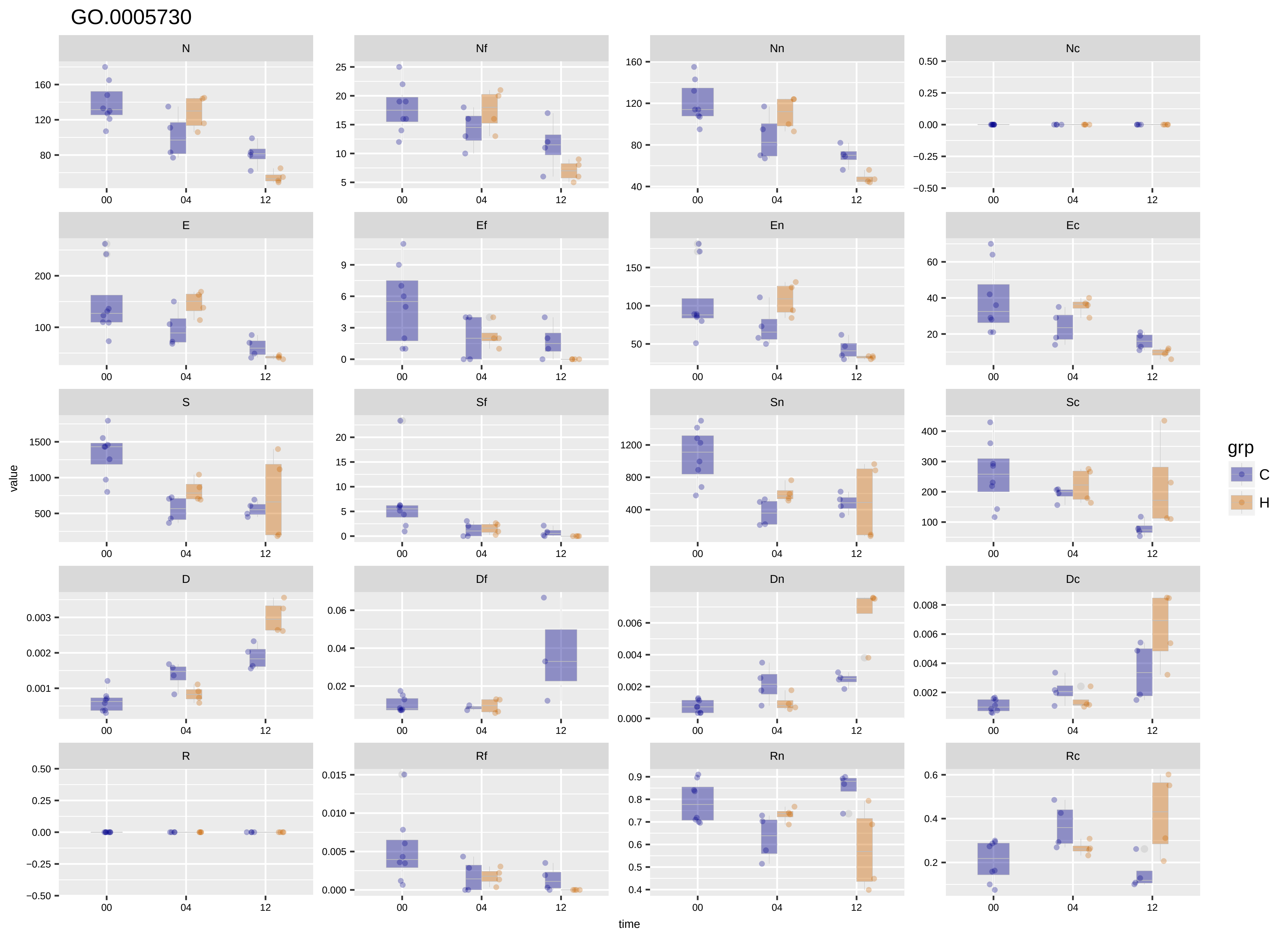
GO.0005730
N
Nf
Nn
Nc
E
Ef
En
Ec
S
Sf
Sn
Sc
D
Df
Dn
Dc
R
Rf
Rn
Rc
value
time
grp
C
H

GO.0005737

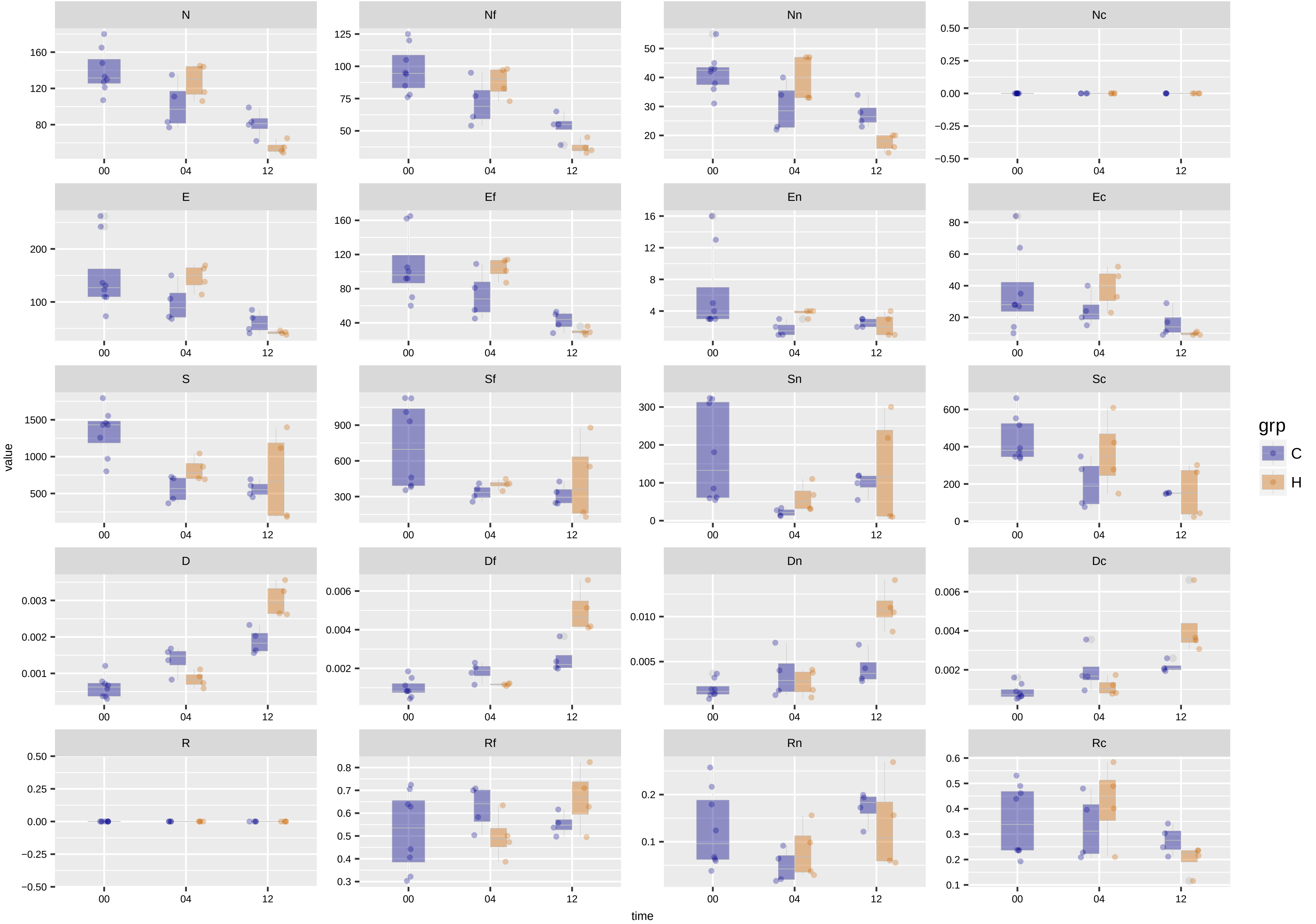

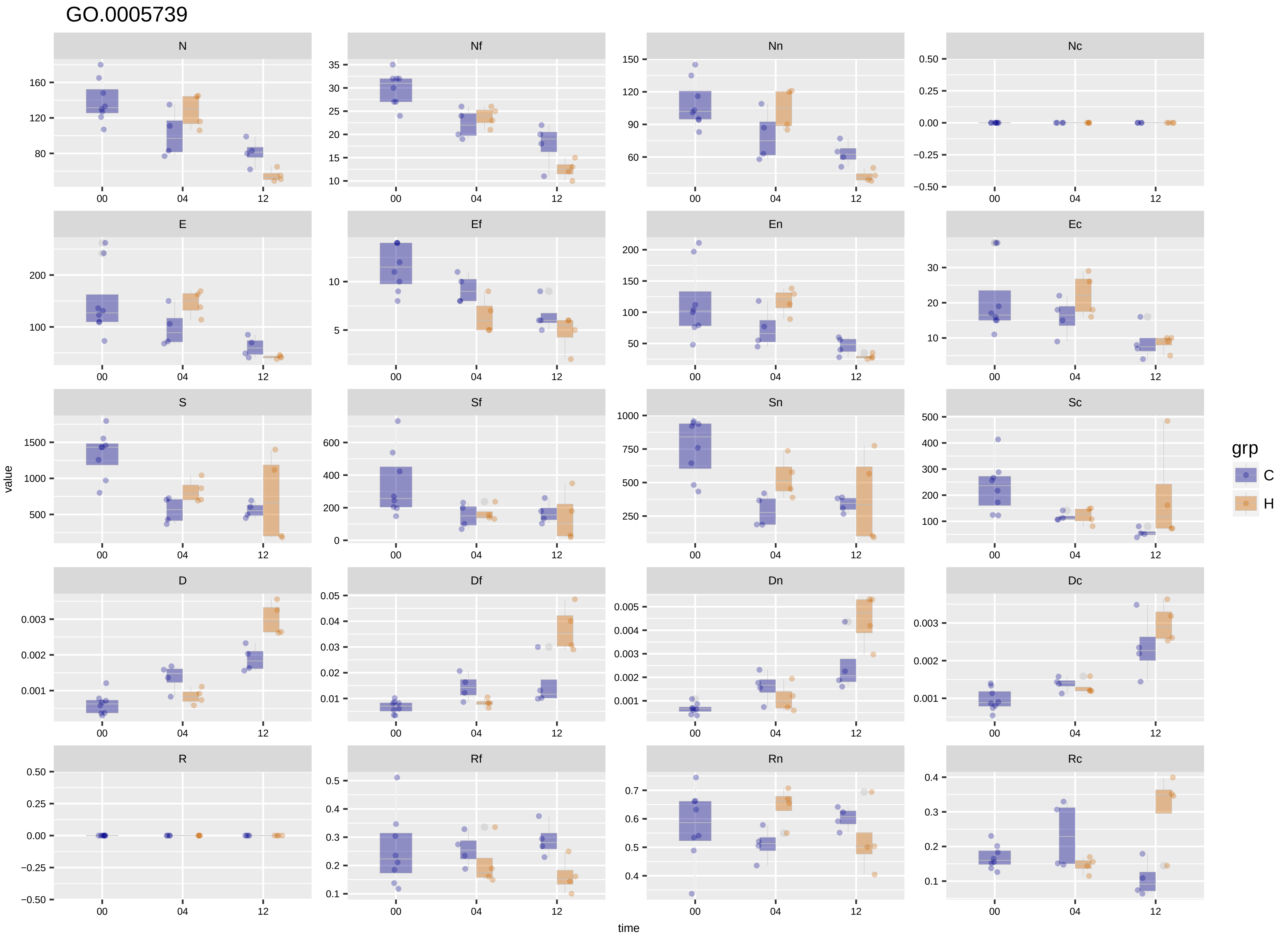

GO.0005739
N
Nf
Nn
Nc
E
Ef
En
Ec
S
Sf
Sn
Sc
D
Df
Dn
Dc
R
Rf
Rn
Rc
value
time
grp
C
H

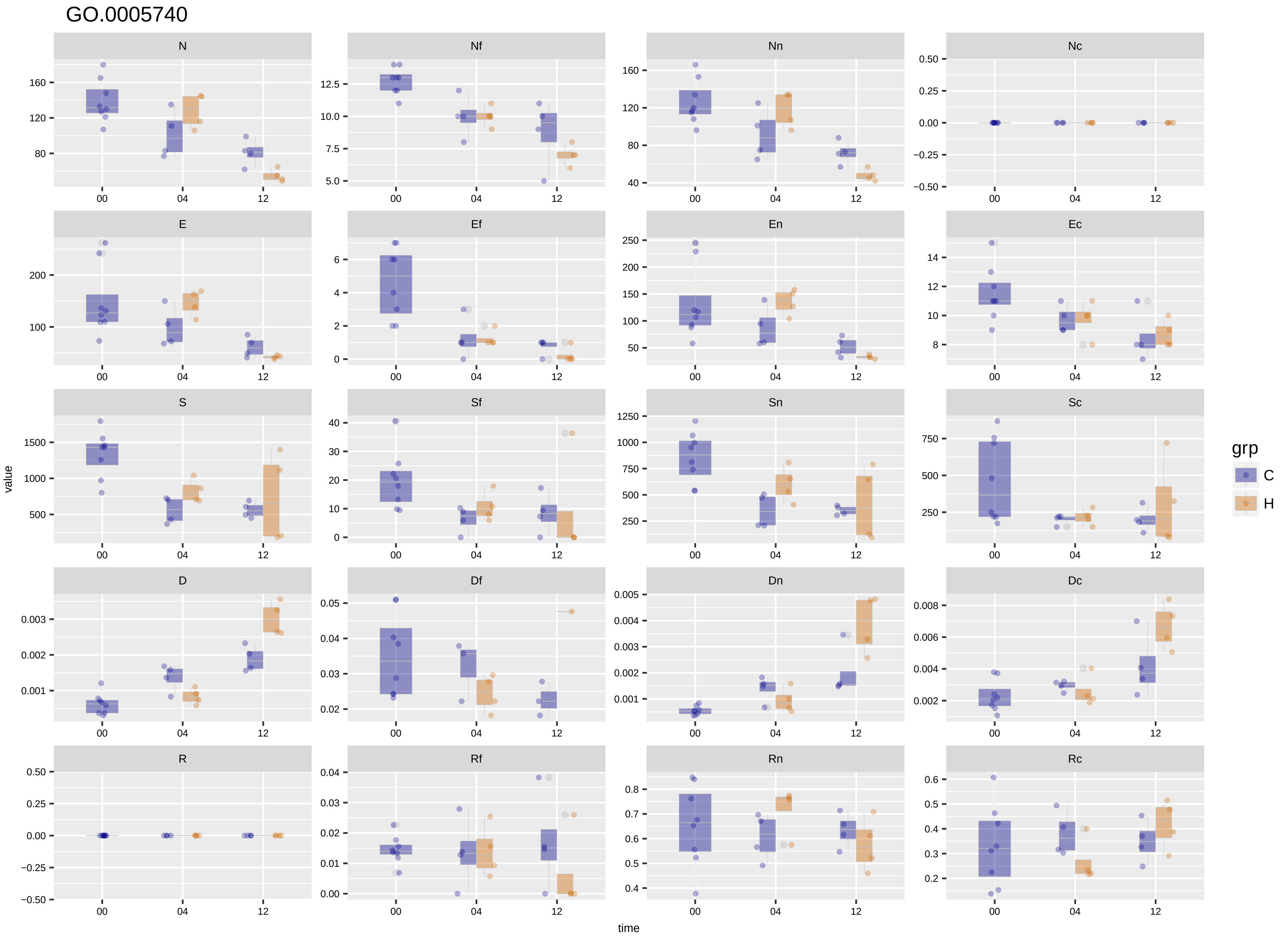
GO.0005740
N
Nf
Nn
Nc
E
Ef
En
Ec
S
Sf
Sn
Sc
D
Df
Dn
Dc
R
Rf
Rn
Rc
value
time
00
04
12
grp
C
H

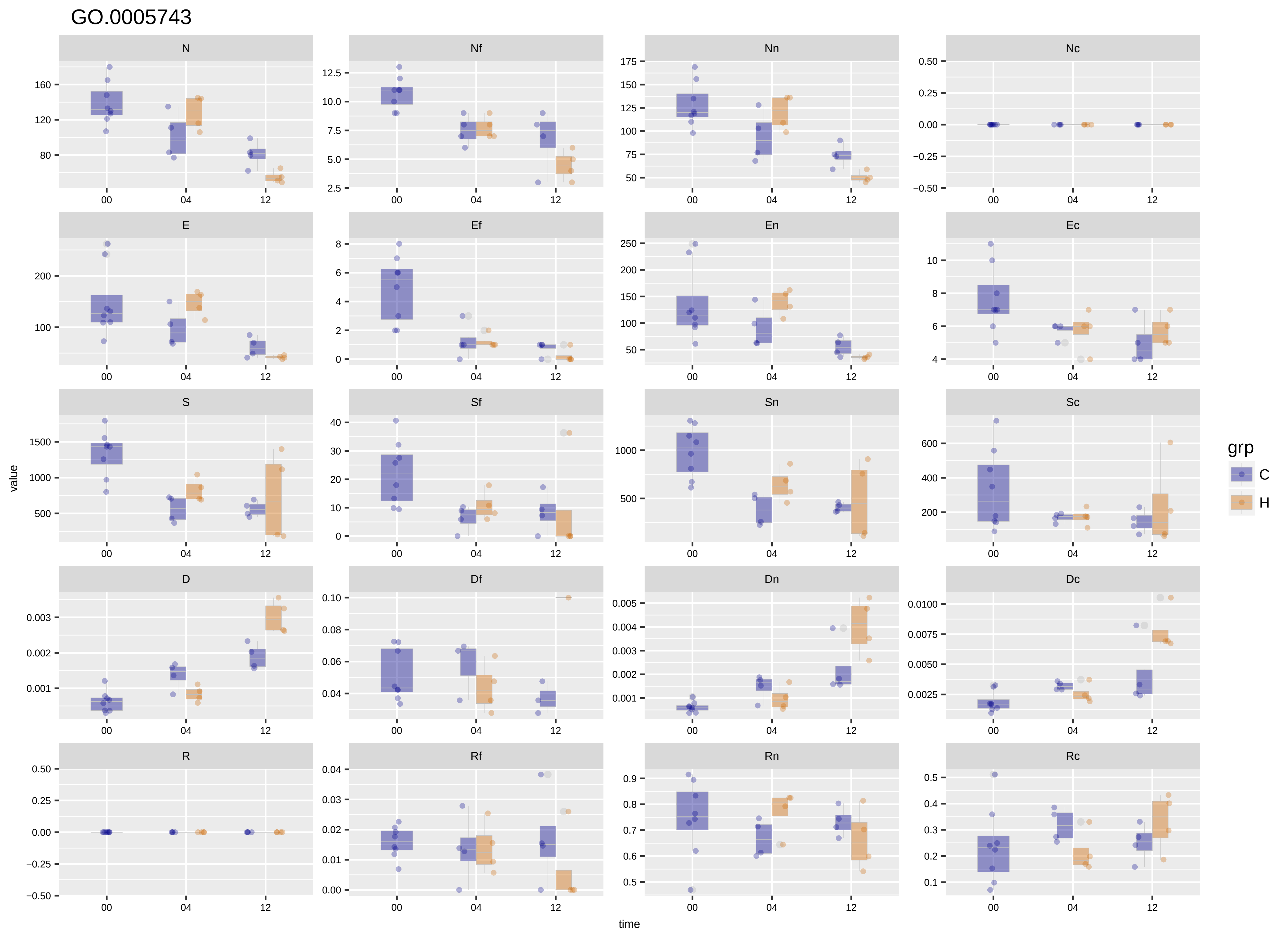

GO.0005743
N
Nf
Nn
Nc
E
Ef
En
Ec
S
Sf
Sn
Sc
D
Df
Dn
Dc
R
Rf
Rn
Rc
00
04
12
value
time
grp
C
H

GO.0005759

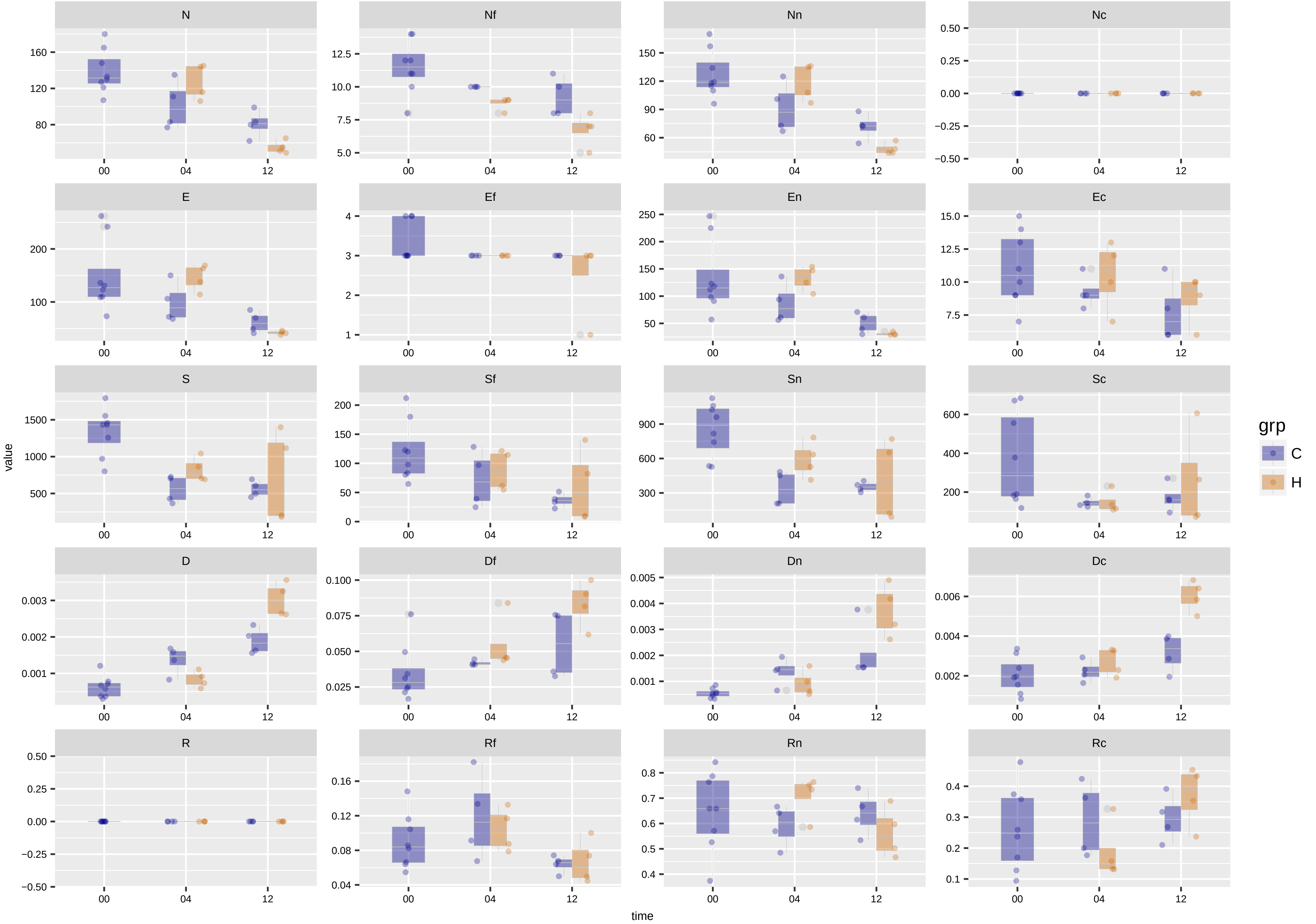
N
Nf
Nn
Nc
E
Ef
En
Ec
S
Sf
Sn
Sc
D
Df
Dn
Dc
R
Rf
Rn
Rc
value
time
00
04
12
grp
C
H

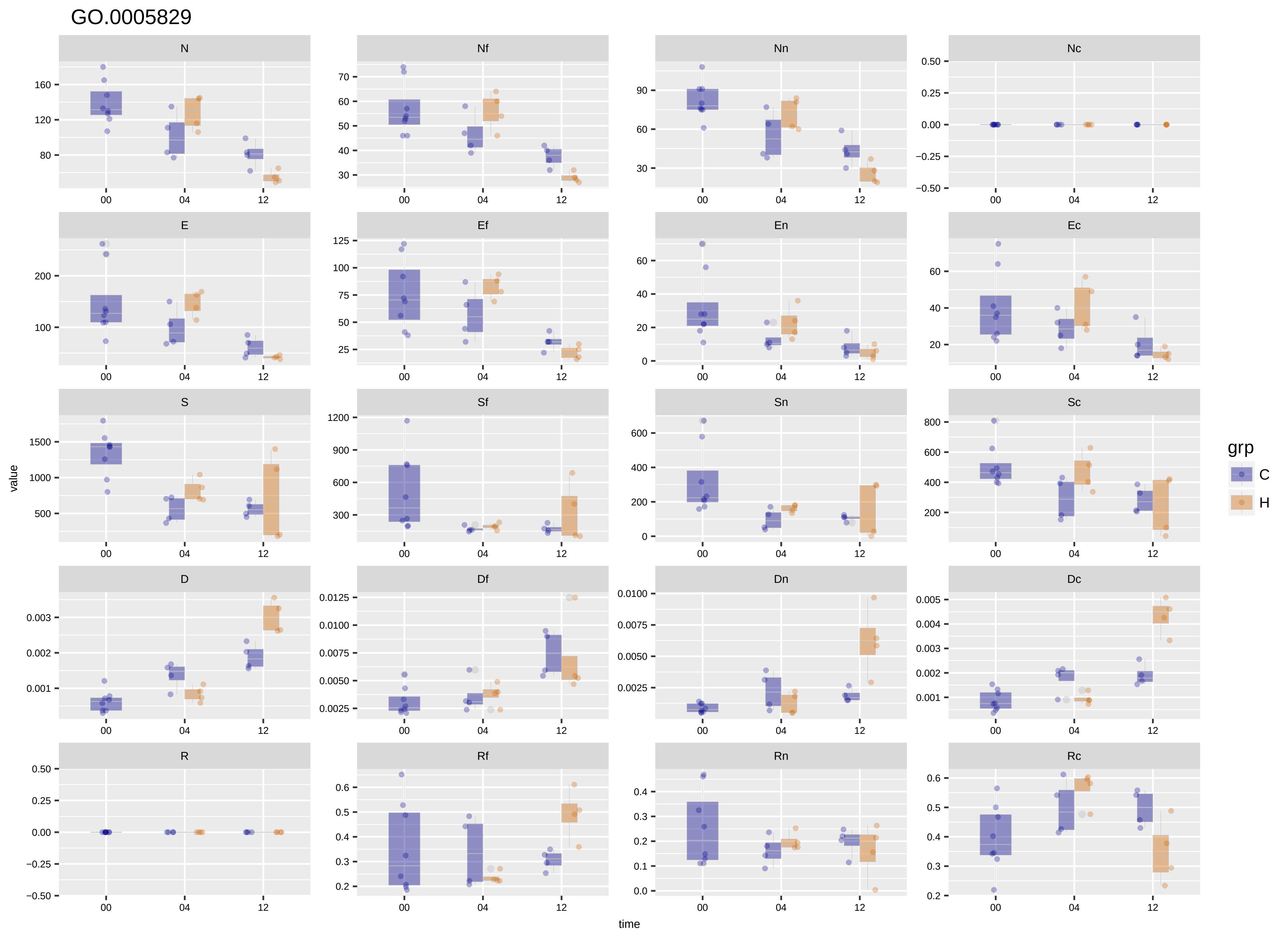
GO.0005829
N
Nf
Nn
Nc
E
Ef
En
Ec
S
Sf
Sn
Sc
D
Df
Dn
Dc
R
Rf
Rn
Rc
value
time
grp
C
H

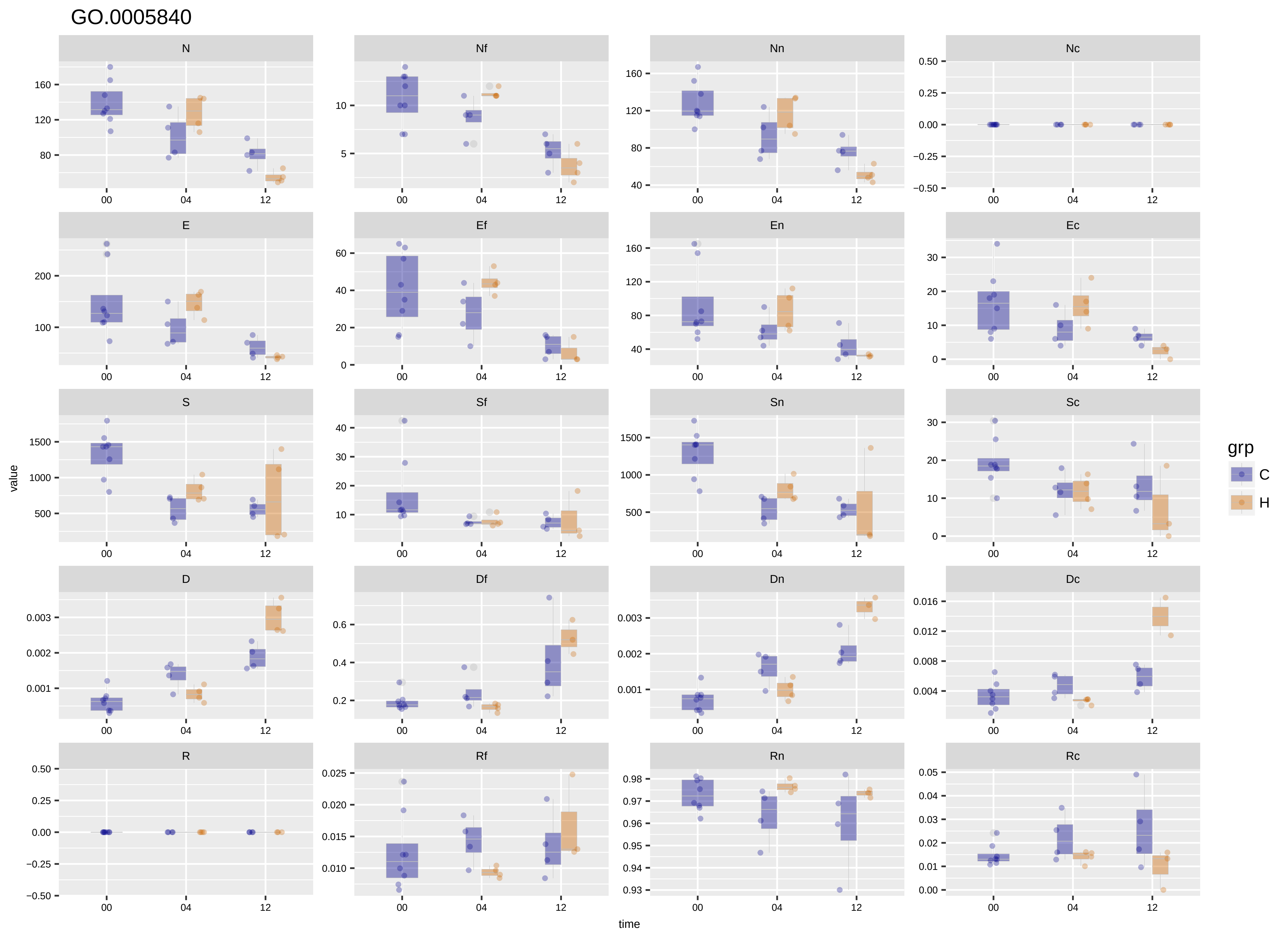

GO.0005840
N
Nf
Nn
Nc
E
Ef
En
Ec
S
Sf
Sn
Sc
D
Df
Dn
Dc
R
Rf
Rn
Rc
value
time
00
04
12
grp
C
H

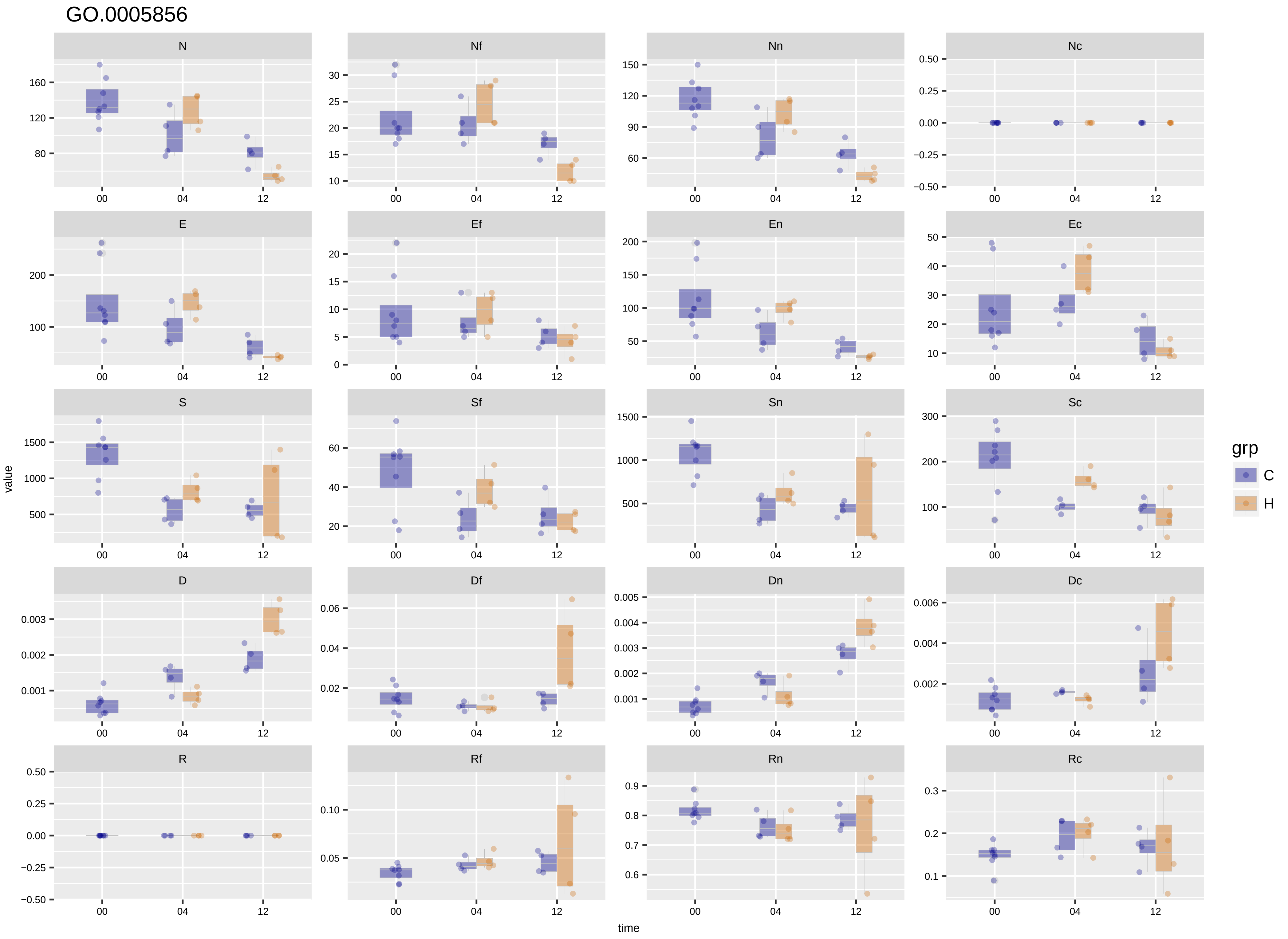

GO.0005856
N
Nf
Nn
Nc
E
Ef
En
Ec
S
Sf
Sn
Sc
D
Df
Dn
Dc
R
Rf
Rn
Rc
value
time
00
04
12
grp
C
H

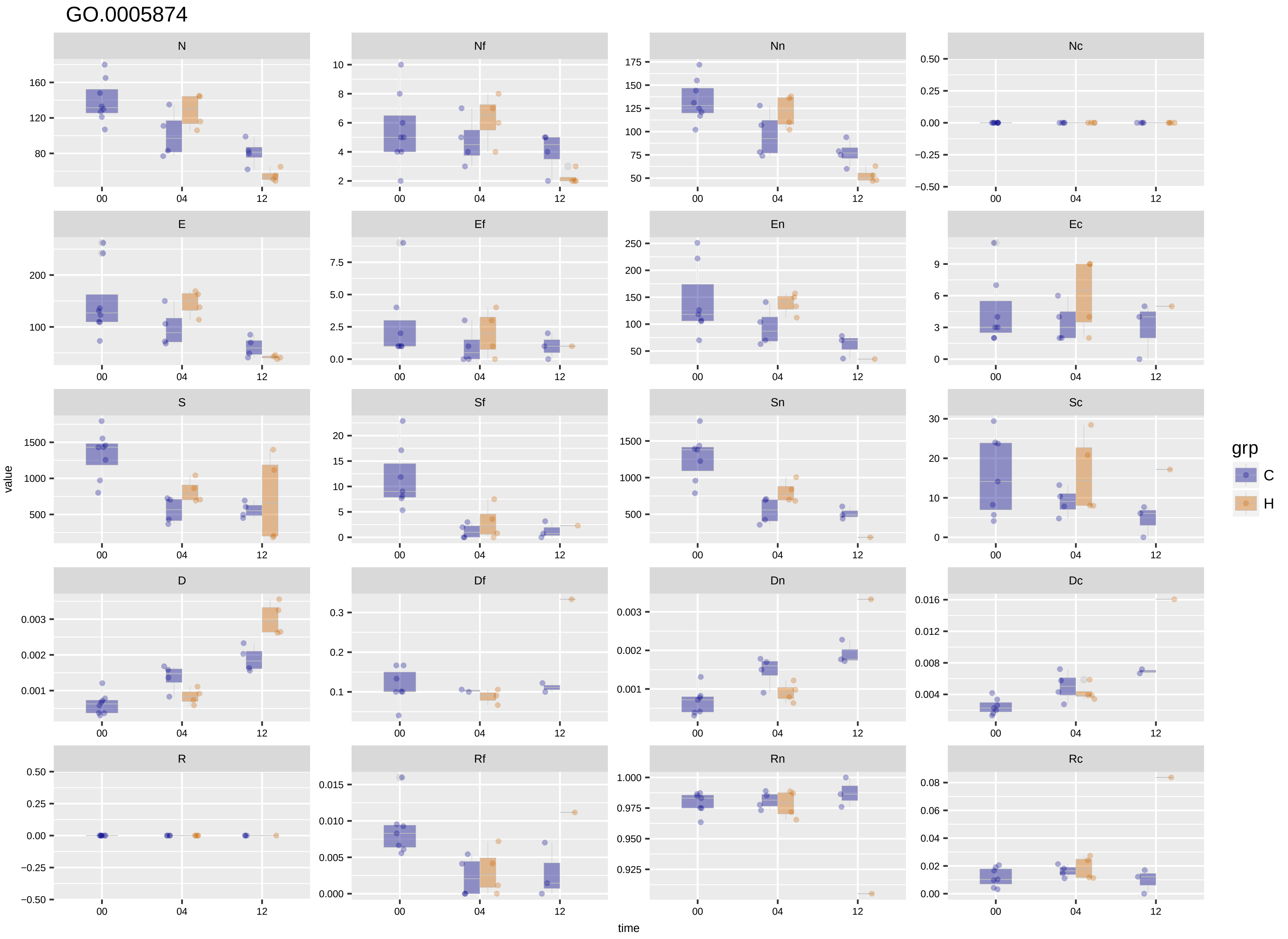

GO.0005874
N
Nf
Nn
Nc
E
Ef
En
Ec
S
Sf
Sn
Sc
D
Df
Dn
Dc
R
Rf
Rn
Rc
value
time
grp
C
H

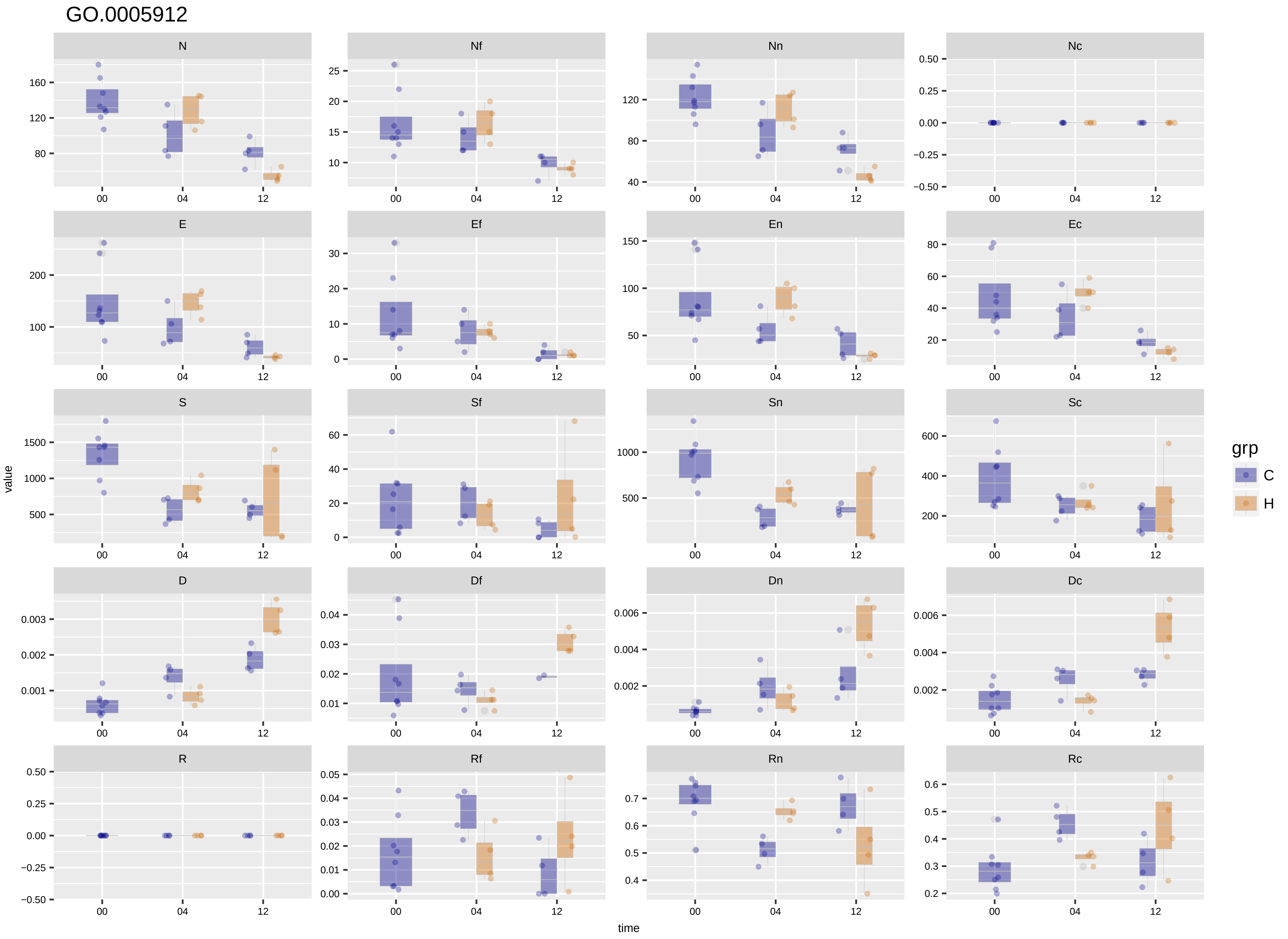
GO.0005912
N
Nf
Nn
Nc
E
Ef
En
Ec
S
Sf
Sn
Sc
D
Df
Dn
Dc
R
Rf
Rn
Rc
value
time
grp
C
H

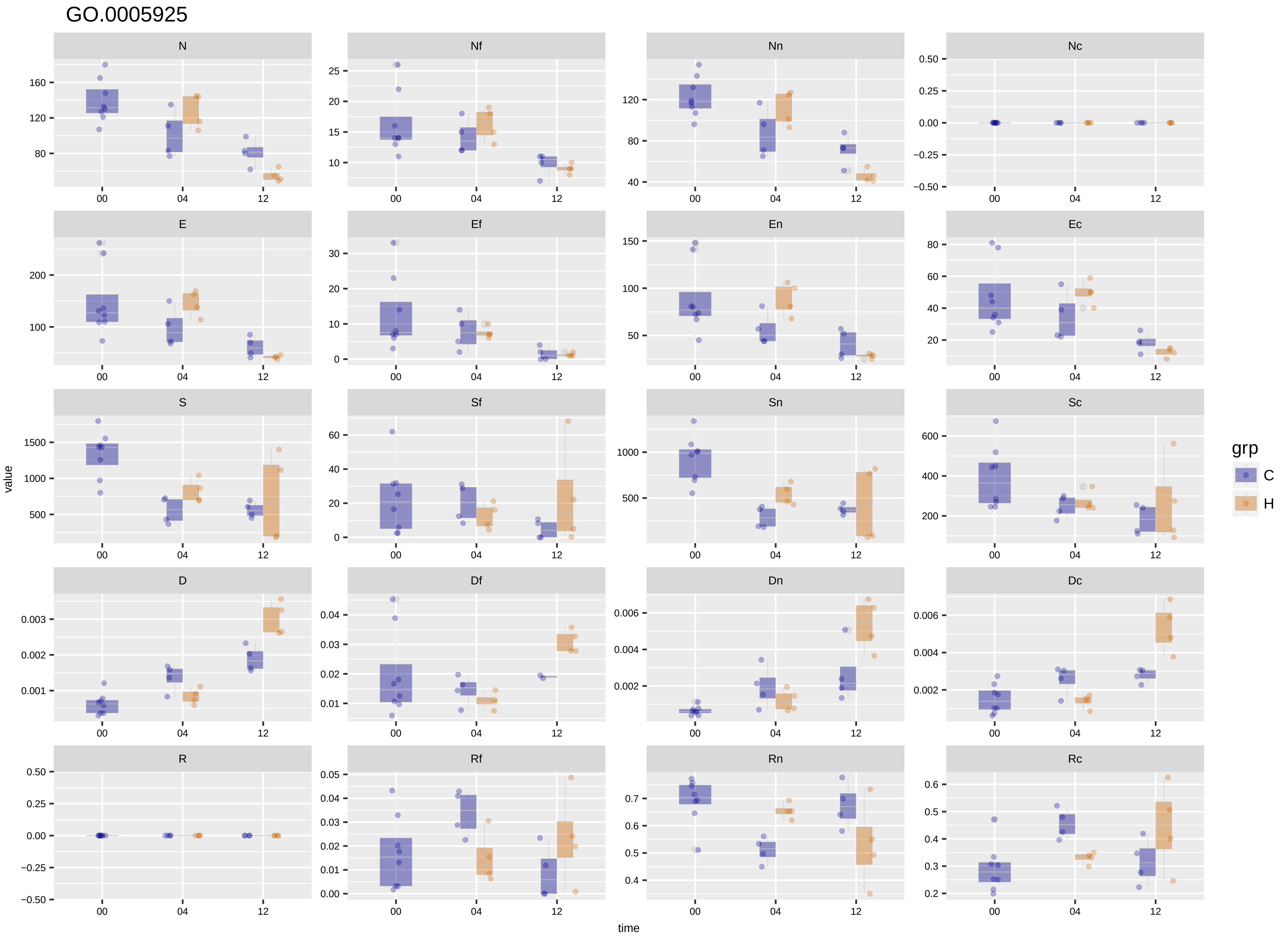
GO.0005925
N
Nf
Nn
Nc
E
Ef
En
Ec
S
Sf
Sn
Sc
D
Df
Dn
Dc
R
Rf
Rn
Rc
00
04
12
value
time
grp
C
H

GO.0006091

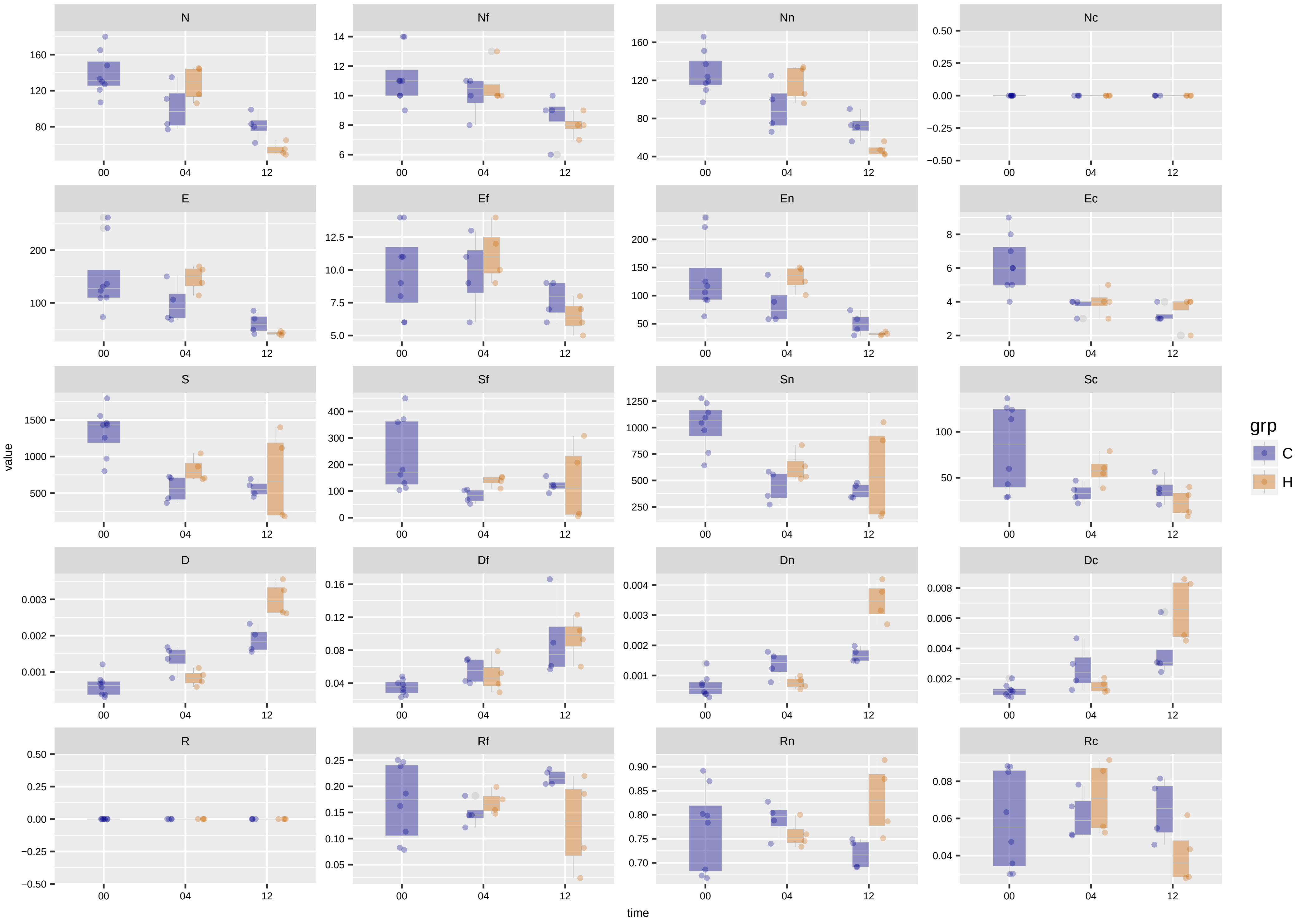

N
Nf
Nn
Nc
E
Ef
En
Ec
S
Sf
Sn
Sc
D
Df
Dn
Dc
R
Rf
Rn
Rc
value
time
00
04
12
grp
C
H

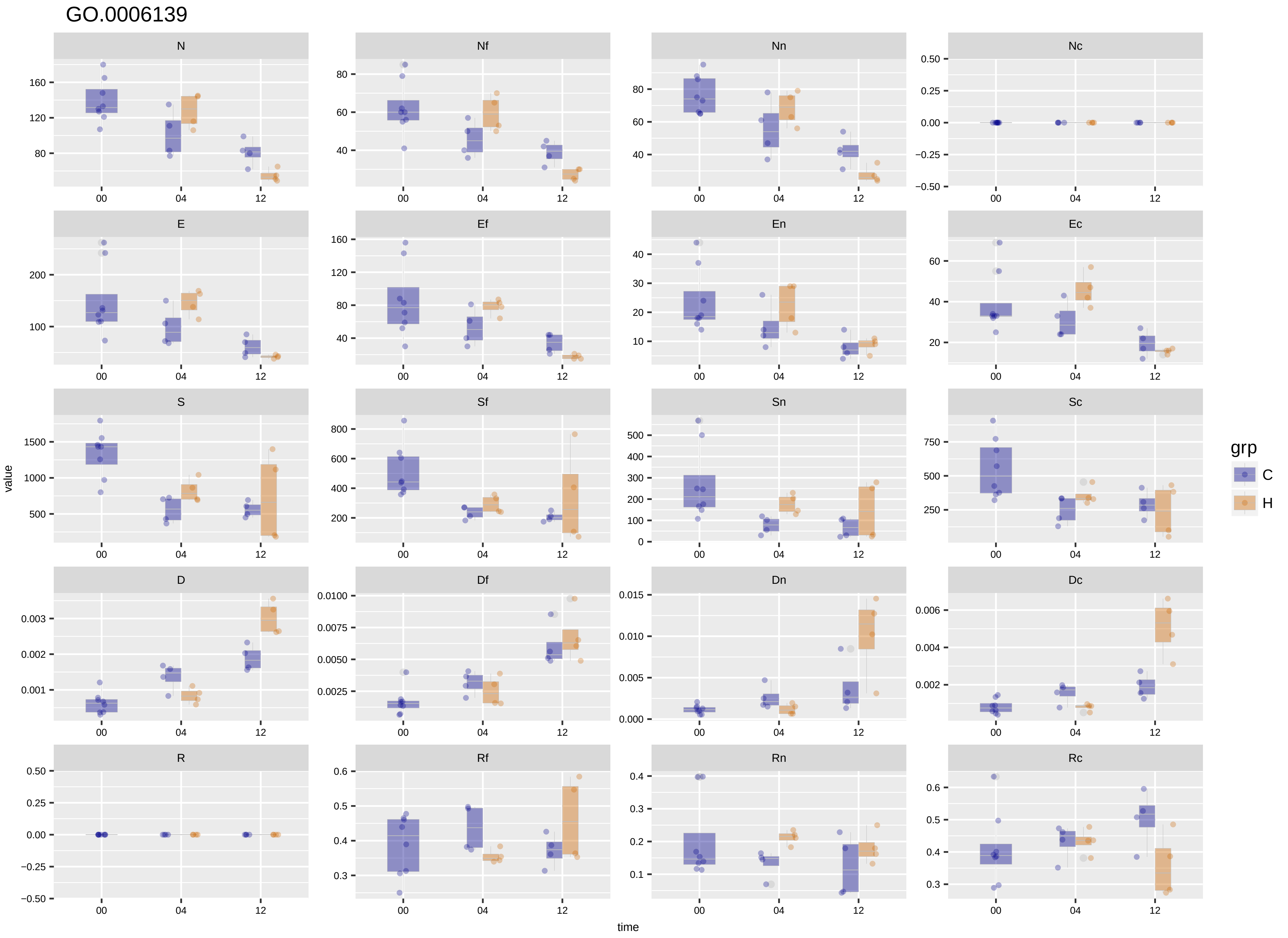
GO.0006139
N
Nf
Nn
Nc
E
Ef
En
Ec
S
Sf
Sn
Sc
D
Df
Dn
Dc
R
Rf
Rn
Rc
value
time
grp
C
H

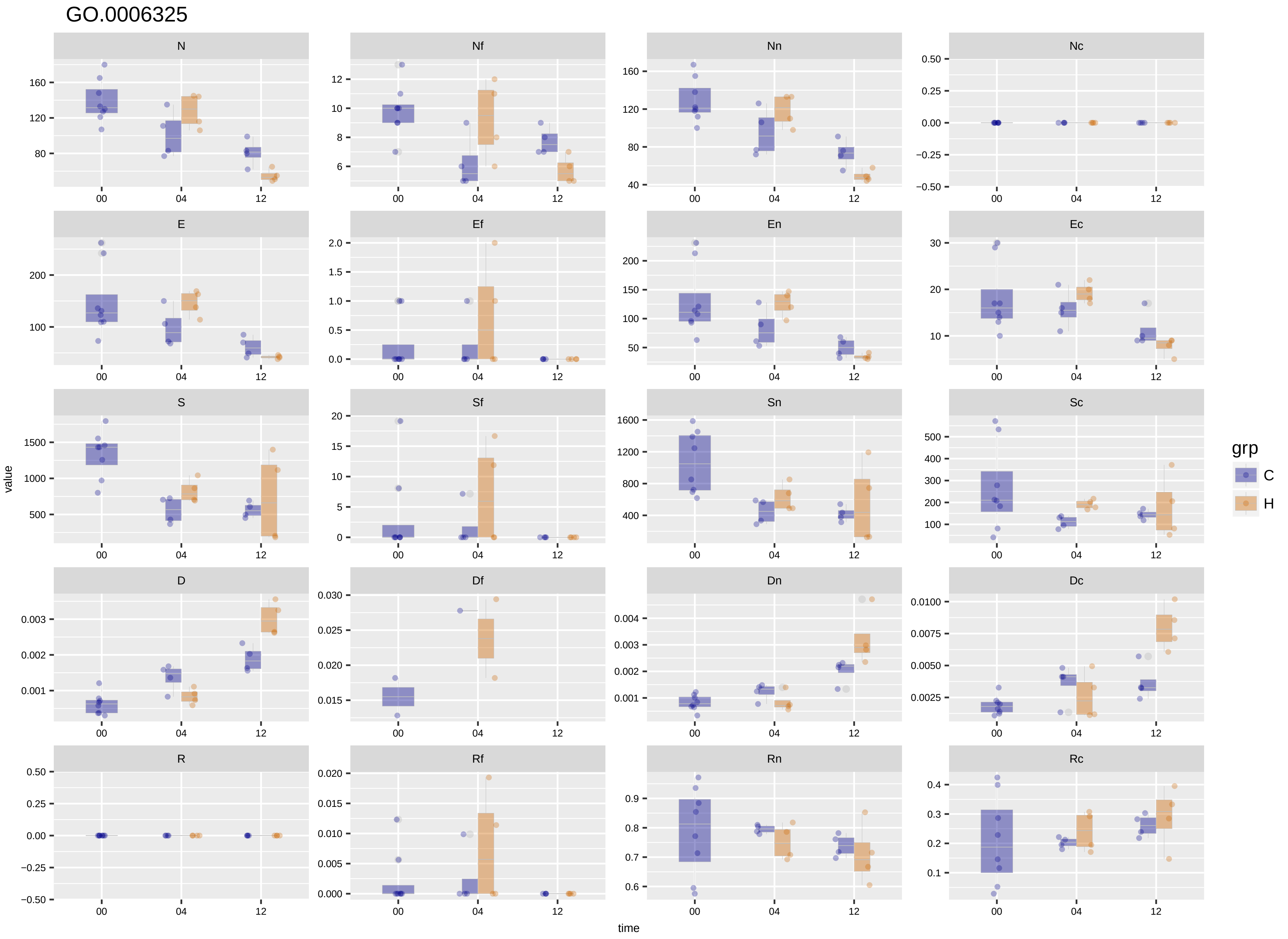

GO.0006325
N
Nf
Nn
Nc
E
Ef
En
Ec
S
Sf
Sn
Sc
D
Df
Dn
Dc
R
Rf
Rn
Rc
value
time
00
04
12
grp
C
H

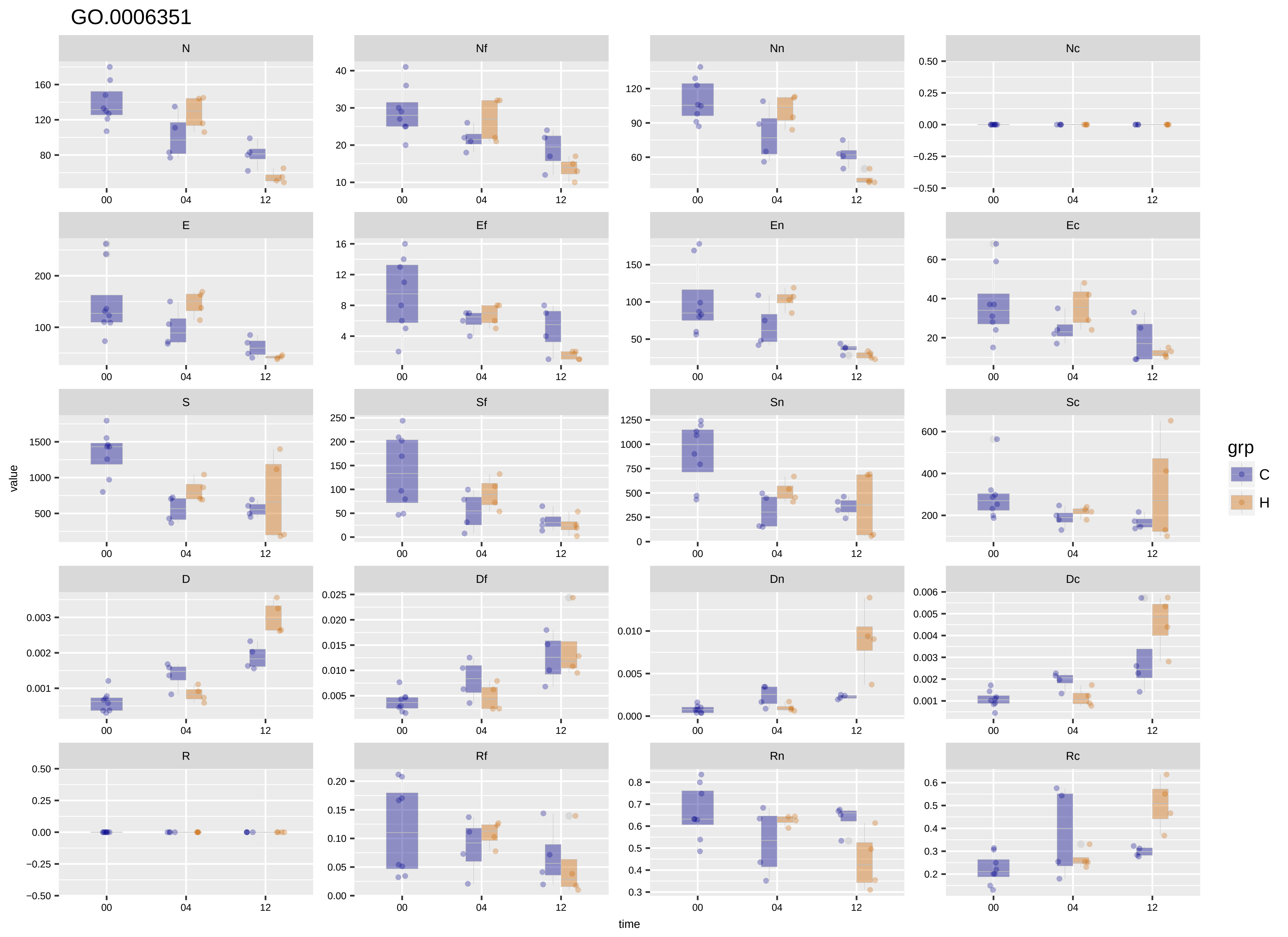

GO.0006351
N
Nf
Nn
Nc
E
Ef
En
Ec
S
Sf
Sn
Sc
D
Df
Dn
Dc
R
Rf
Rn
Rc
value
time
grp
C
H

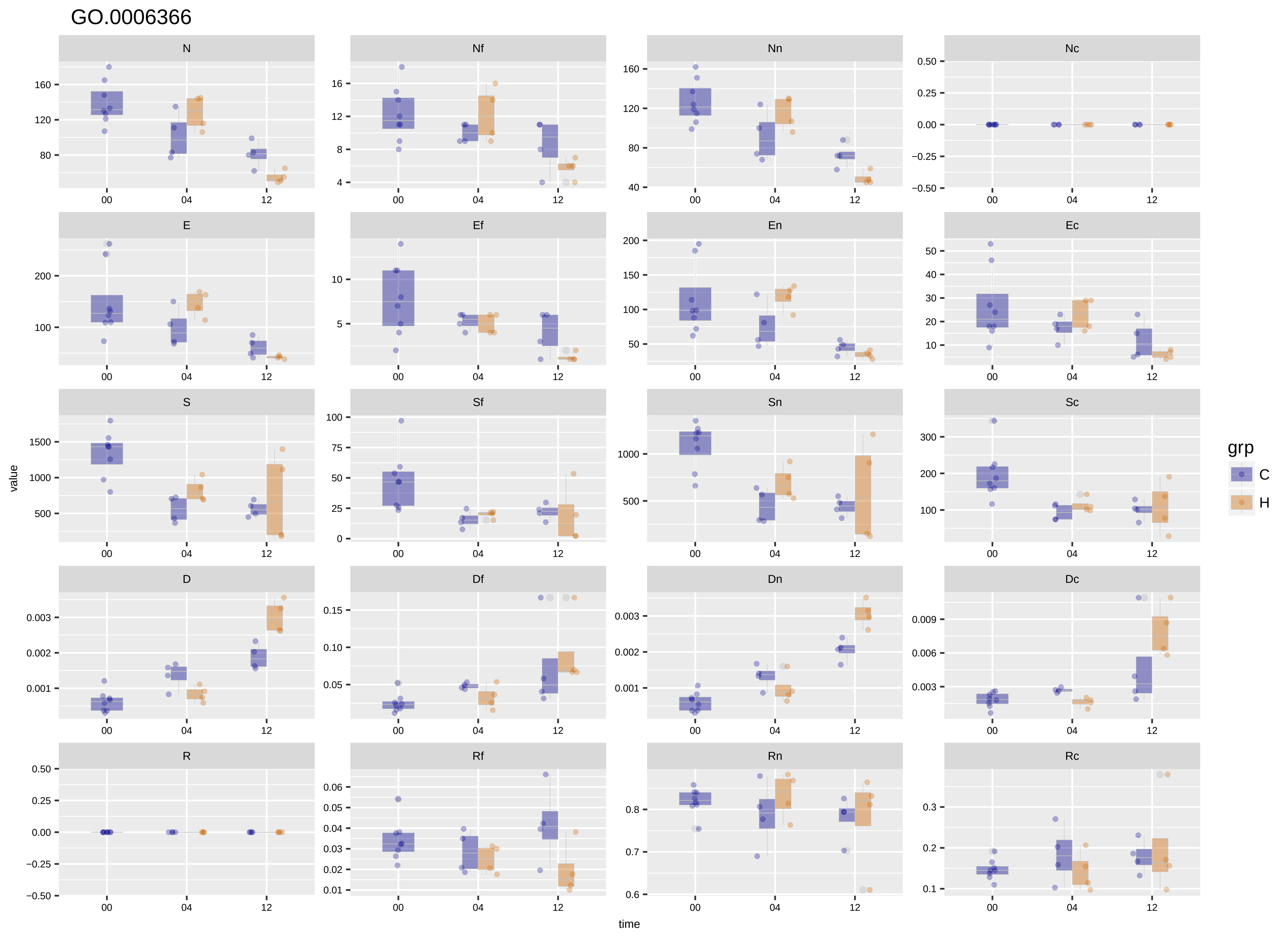
GO.0006366
N
Nf
Nn
Nc
E
Ef
En
Ec
S
Sf
Sn
Sc
D
Df
Dn
Dc
R
Rf
Rn
Rc
00
04
12
value
time
grp
C
H

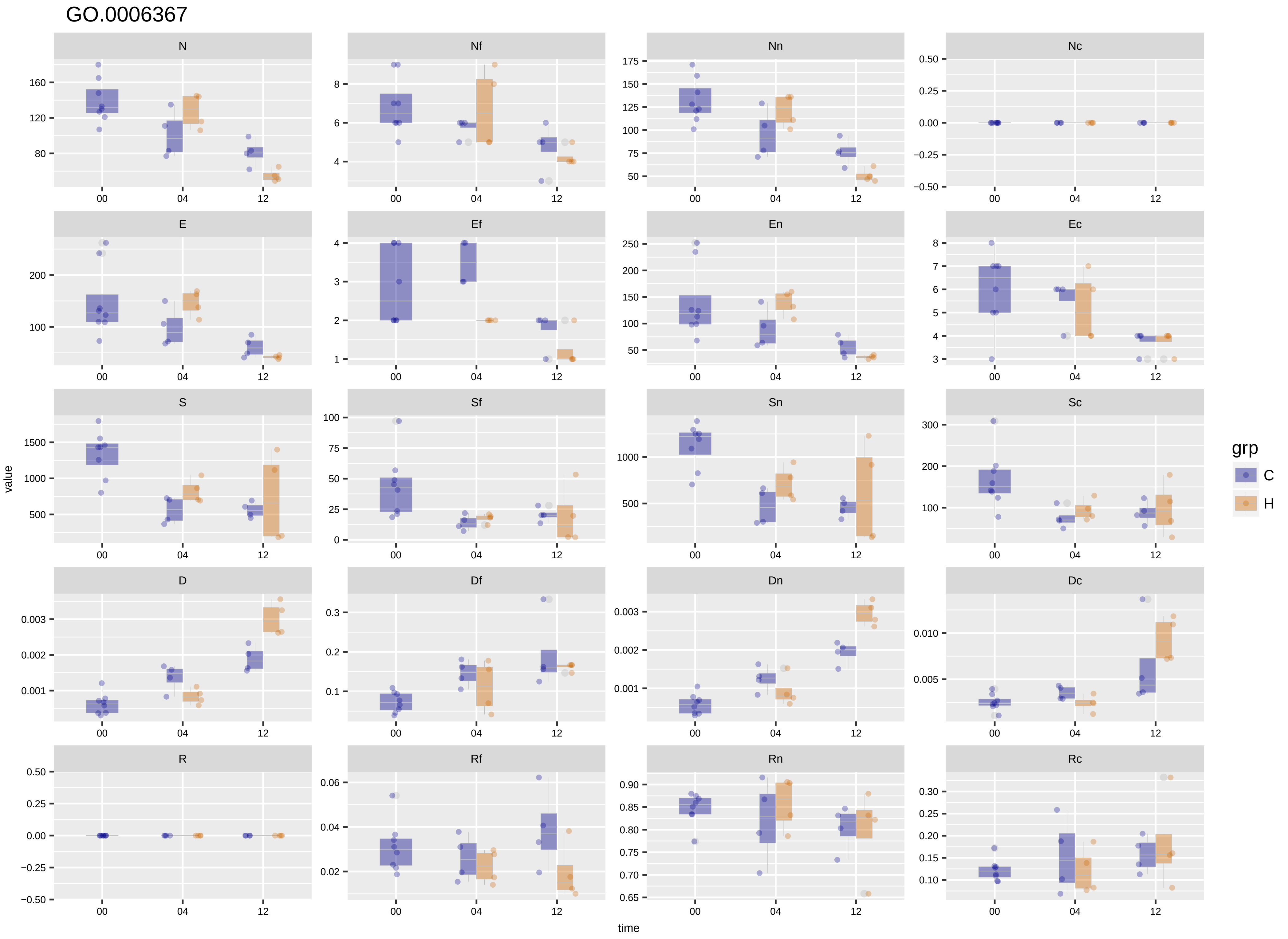

GO.0006367
N
Nf
Nn
Nc
E
Ef
En
Ec
S
Sf
Sn
Sc
D
Df
Dn
Dc
R
Rf
Rn
Rc
value
time
00
04
12
grp
C
H

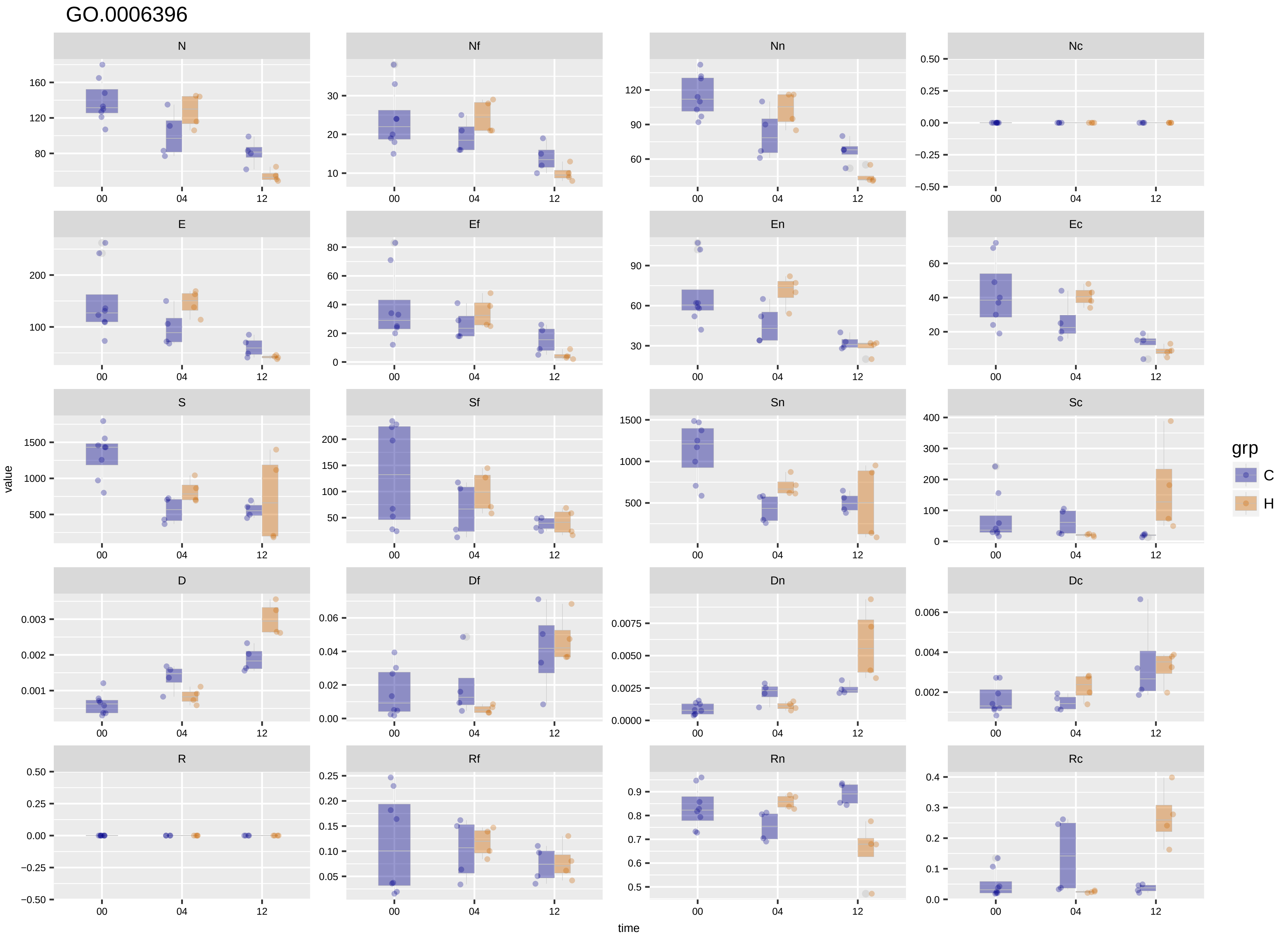
GO.0006396
N
Nf
Nn
Nc
E
Ef
En
Ec
S
Sf
Sn
Sc
D
Df
Dn
Dc
R
Rf
Rn
Rc
value
time
00
04
12
grp
C
H

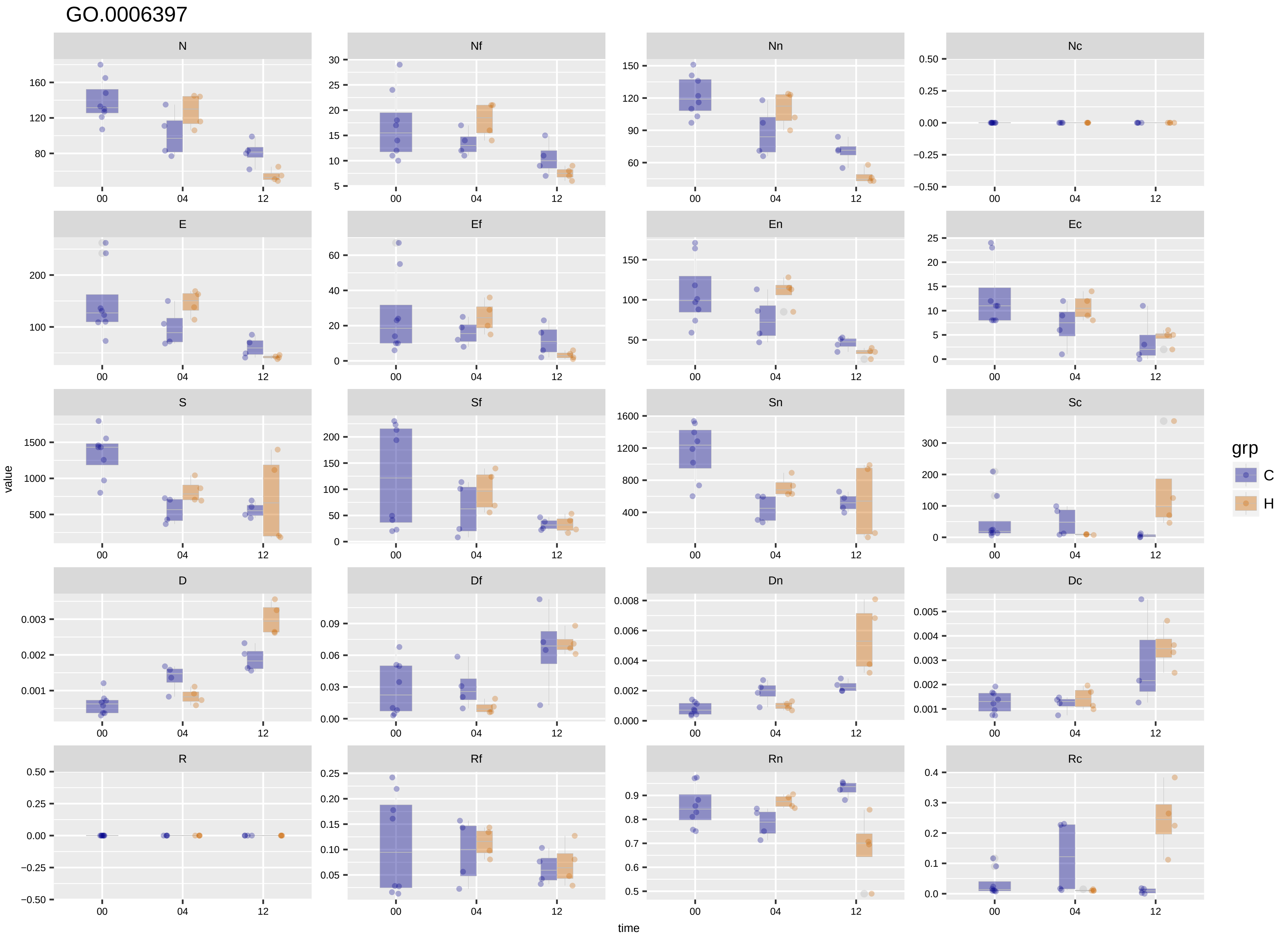

GO.0006397
N
Nf
Nn
Nc
E
Ef
En
Ec
S
Sf
Sn
Sc
D
Df
Dn
Dc
R
Rf
Rn
Rc
value
time
grp
C
H

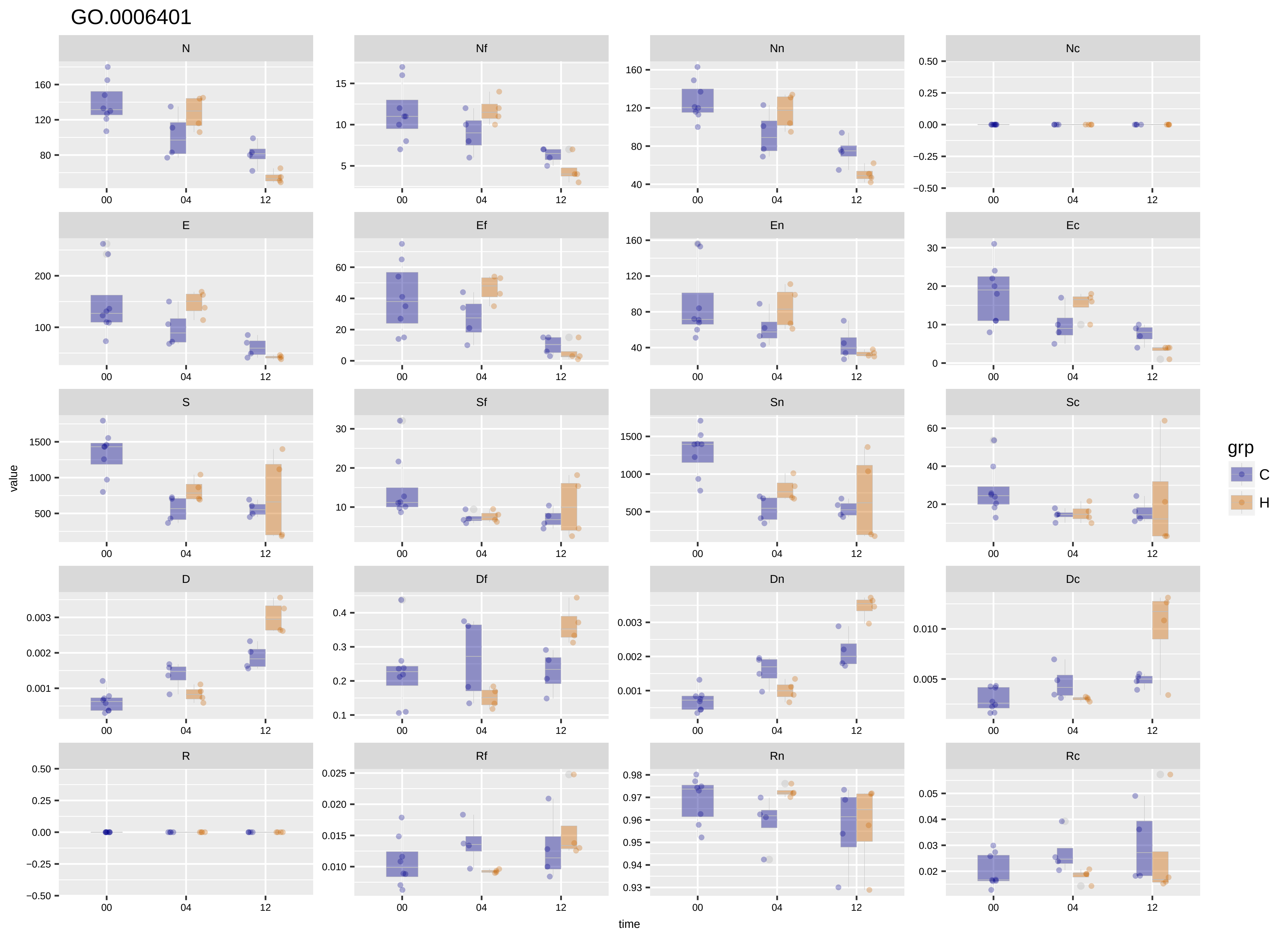

GO.0006401
N
Nf
Nn
Nc
E
Ef
En
Ec
S
Sf
Sn
Sc
D
Df
Dn
Dc
R
Rf
Rn
Rc
value
time
grp
C
H
00
04
12

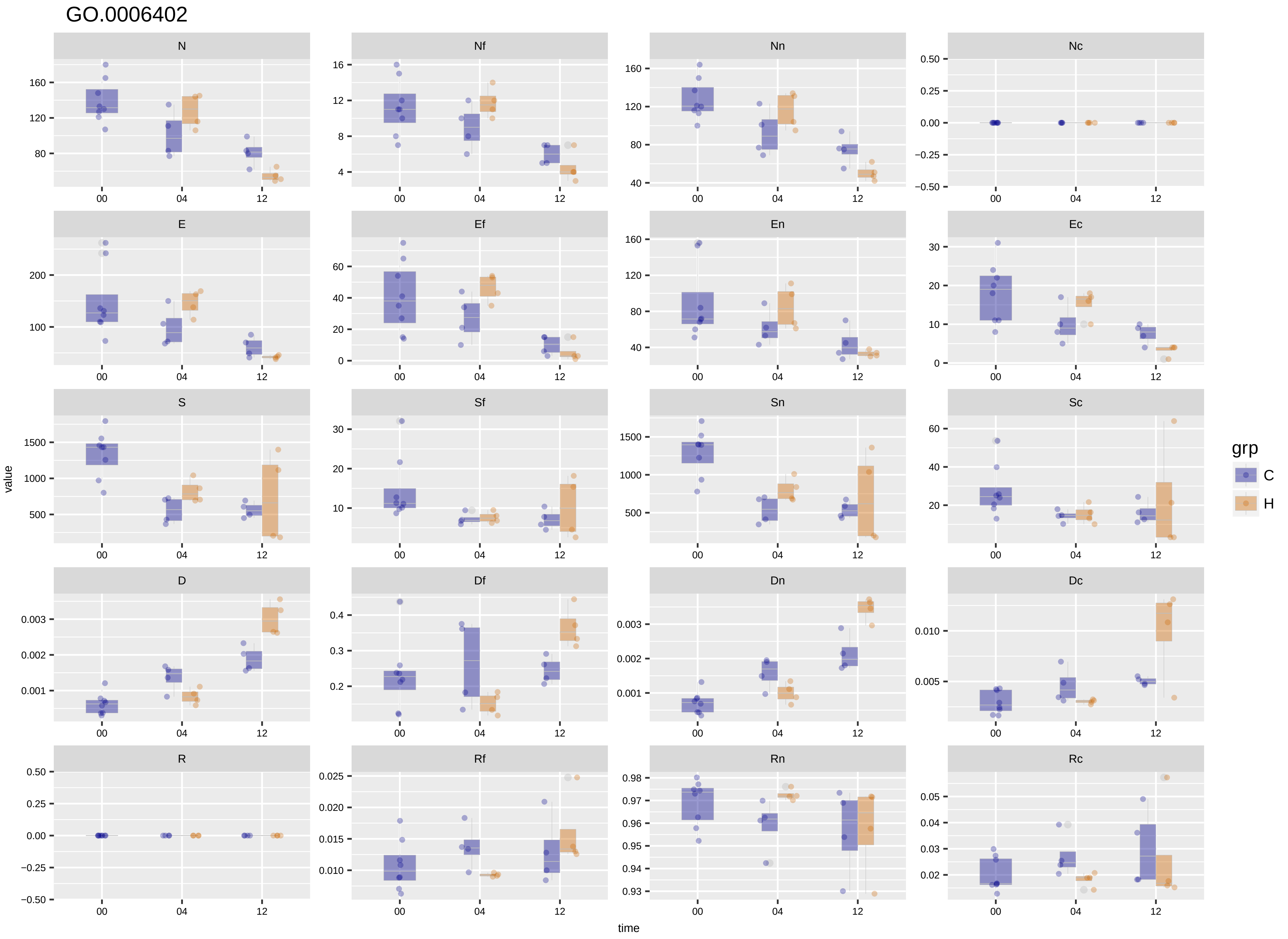

GO.0006402
N
Nf
Nn
Nc
E
Ef
En
Ec
S
Sf
Sn
Sc
D
Df
Dn
Dc
R
Rf
Rn
Rc
00
04
12
value
time
grp
C
H

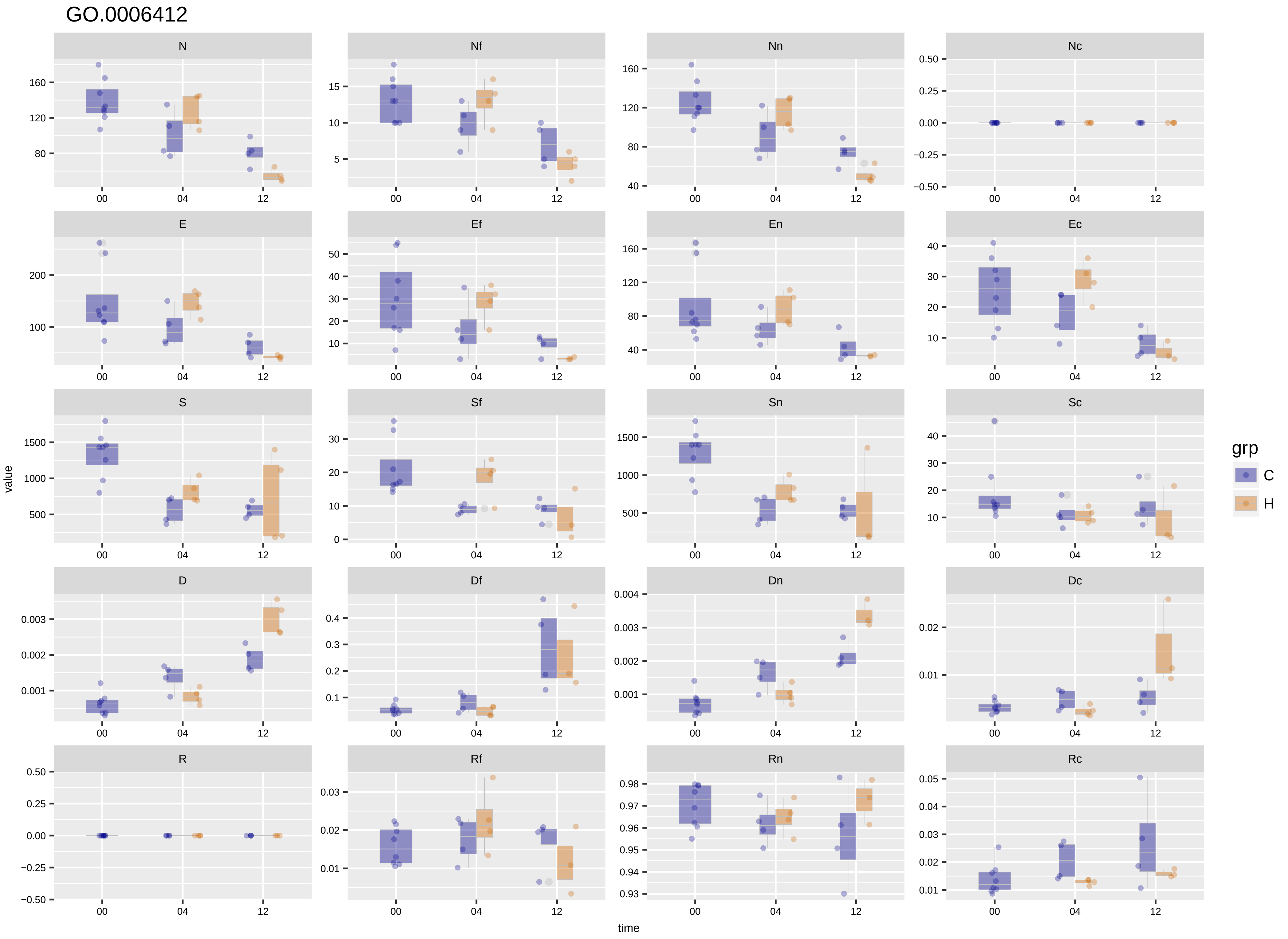
GO.0006412
N
Nf
Nn
Nc
E
Ef
En
Ec
S
Sf
Sn
Sc
D
Df
Dn
Dc
R
Rf
Rn
Rc
value
time
00
04
12
grp
C
H

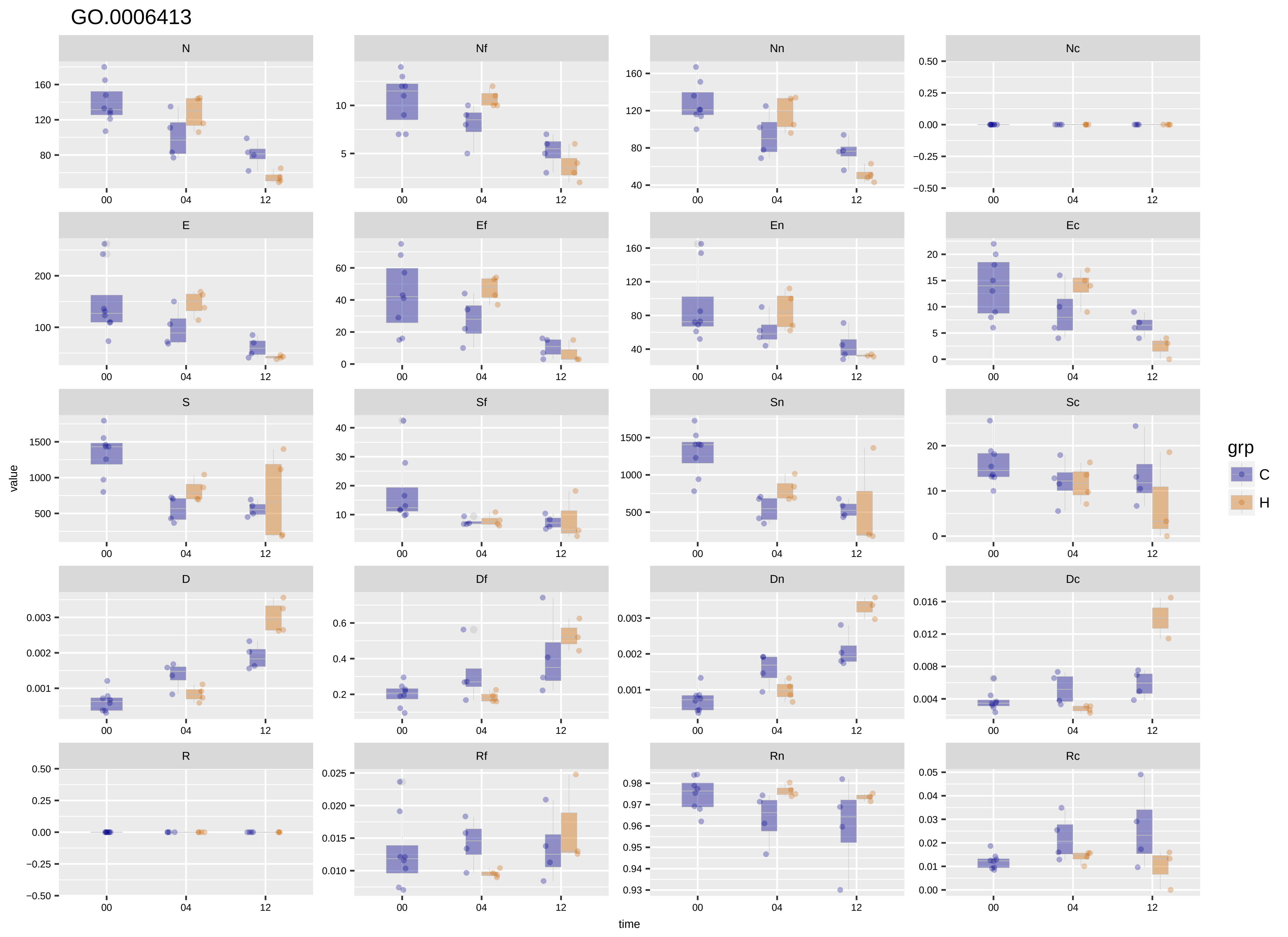
GO.0006413
N
Nf
Nn
Nc
E
Ef
En
Ec
S
Sf
Sn
Sc
D
Df
Dn
Dc
R
Rf
Rn
Rc
value
time
00
04
12
grp
C
H

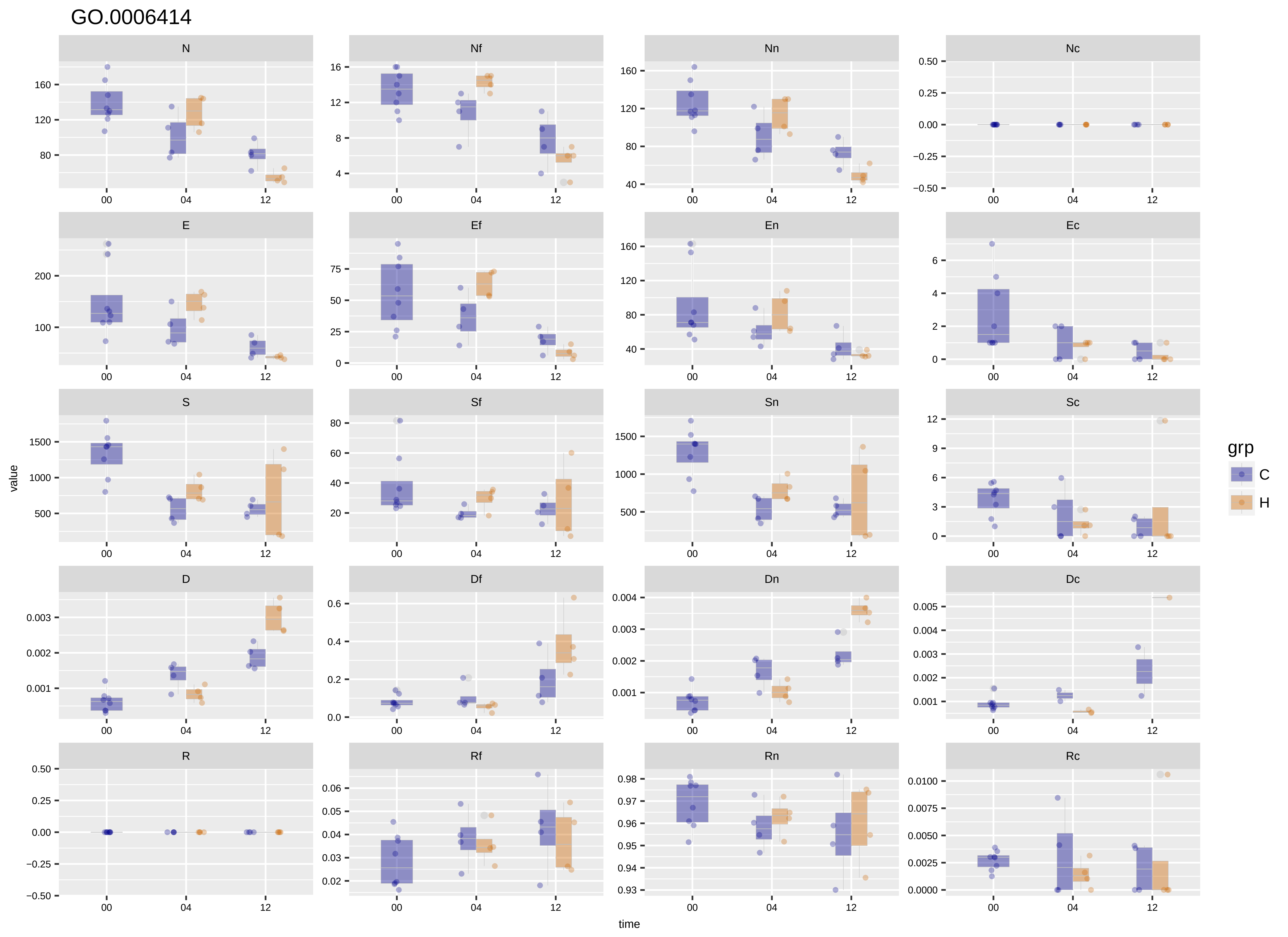
GO.0006414
N
Nf
Nn
Nc
E
Ef
En
Ec
S
Sf
Sn
Sc
D
Df
Dn
Dc
R
Rf
Rn
Rc
value
time
grp
C
H

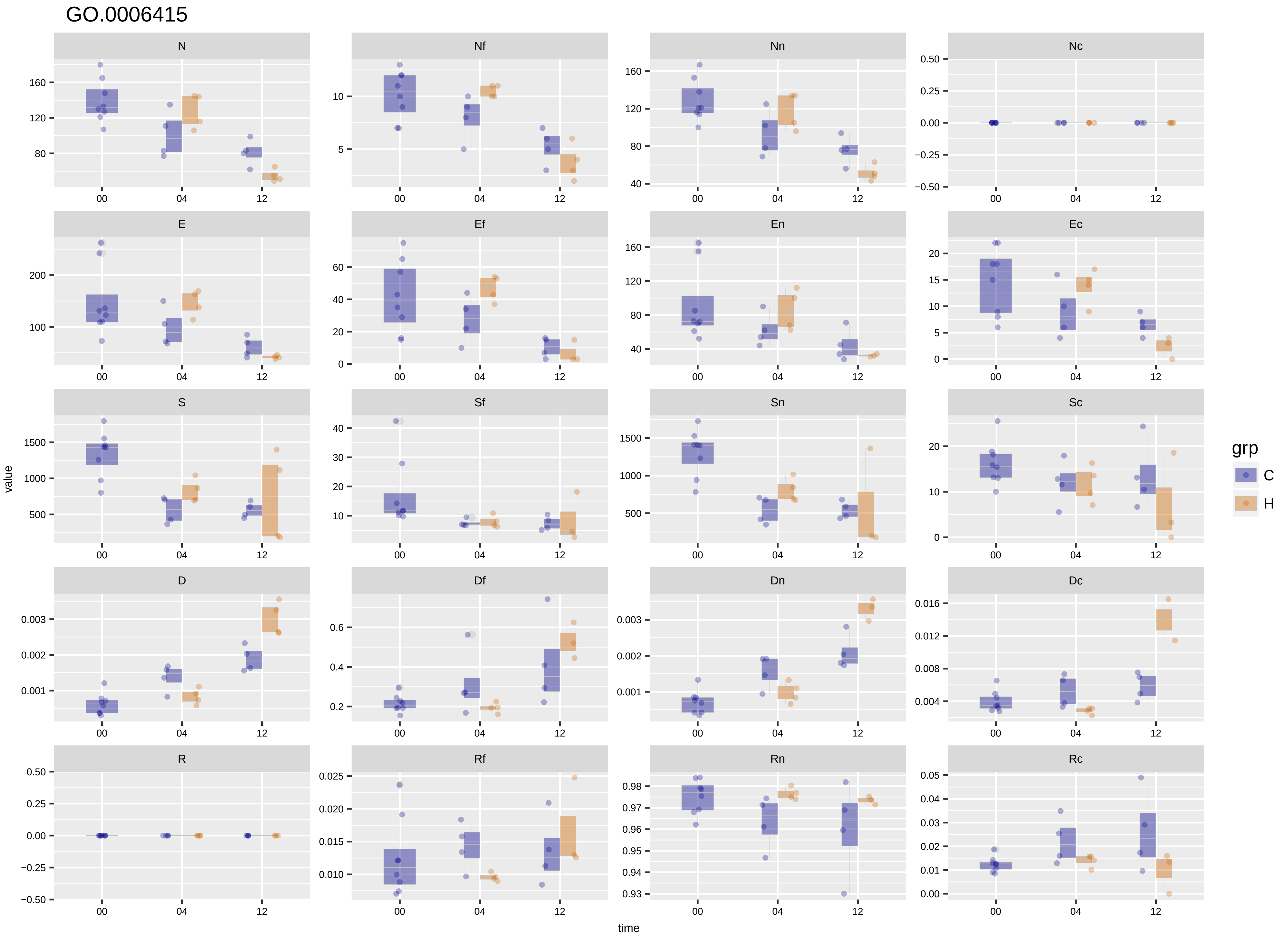
GO.0006415
N
Nf
Nn
Nc
E
Ef
En
Ec
S
Sf
Sn
Sc
D
Df
Dn
Dc
R
Rf
Rn
Rc
value
time
grp
C
H

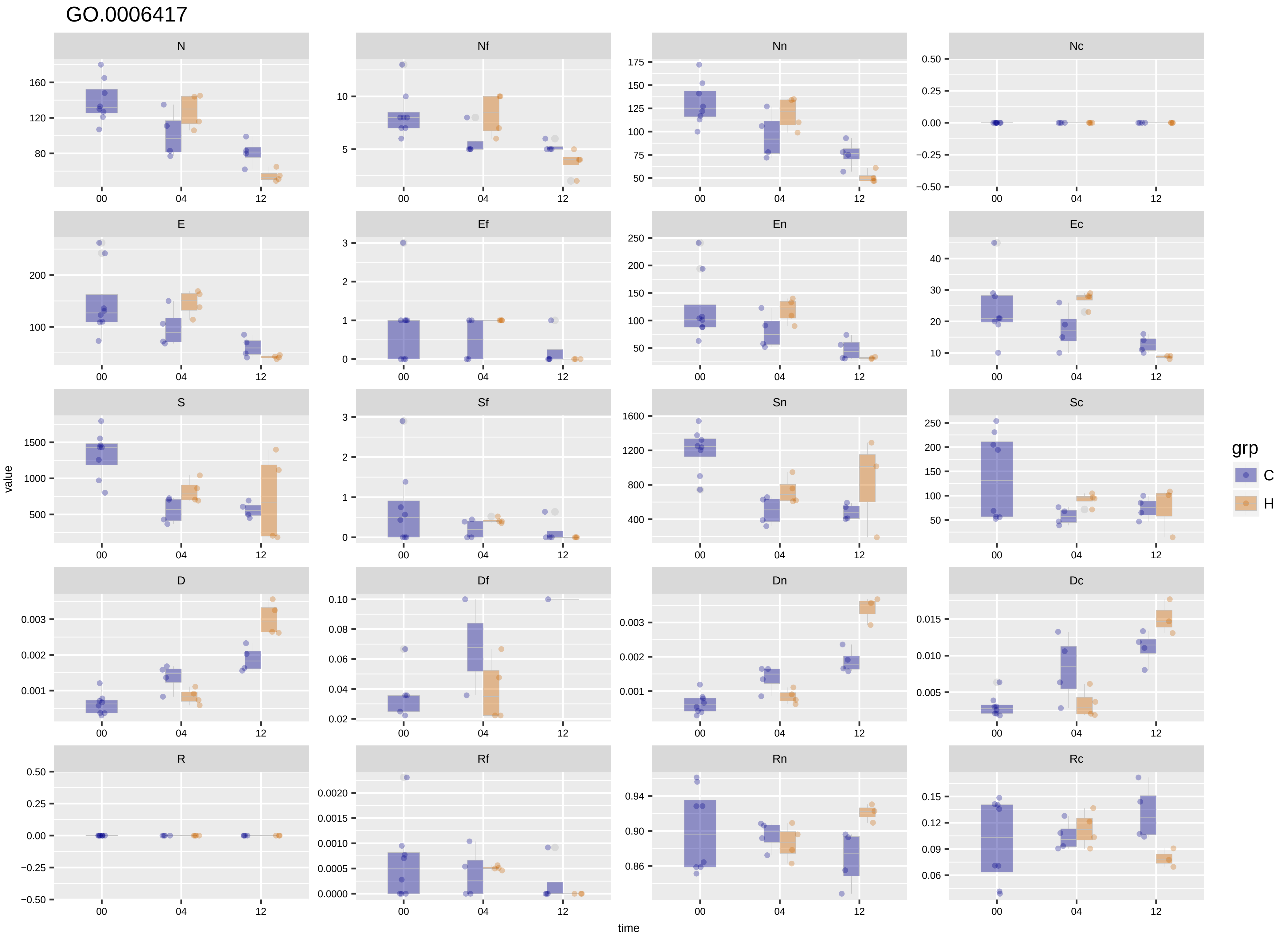
GO.0006417
N
Nf
Nn
Nc
E
Ef
En
Ec
S
Sf
Sn
Sc
D
Df
Dn
Dc
R
Rf
Rn
Rc
value
time
grp
C
H

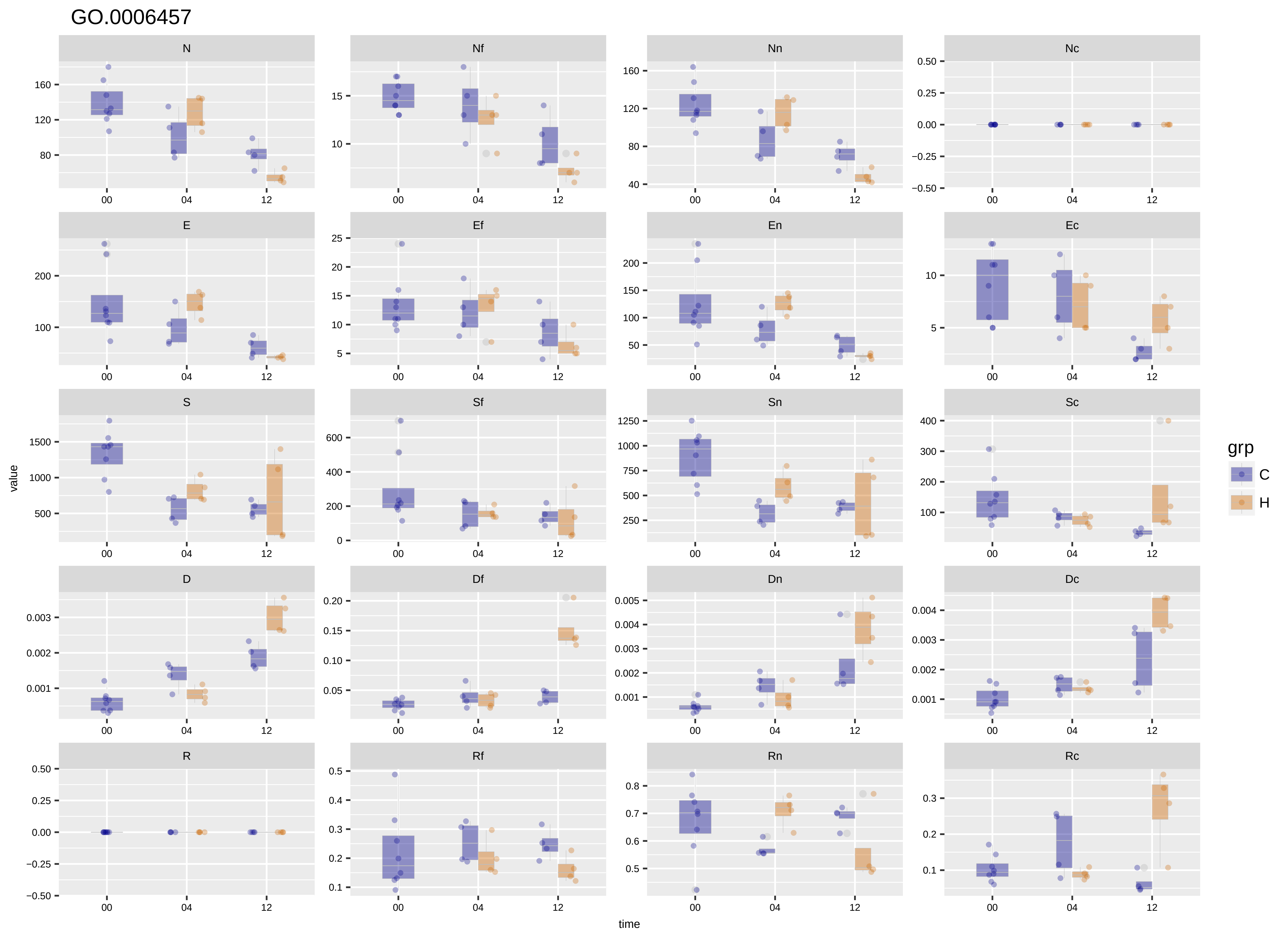
GO.0006457
N
Nf
Nn
Nc
E
Ef
En
Ec
S
Sf
Sn
Sc
D
Df
Dn
Dc
R
Rf
Rn
Rc
value
time
grp
C
H

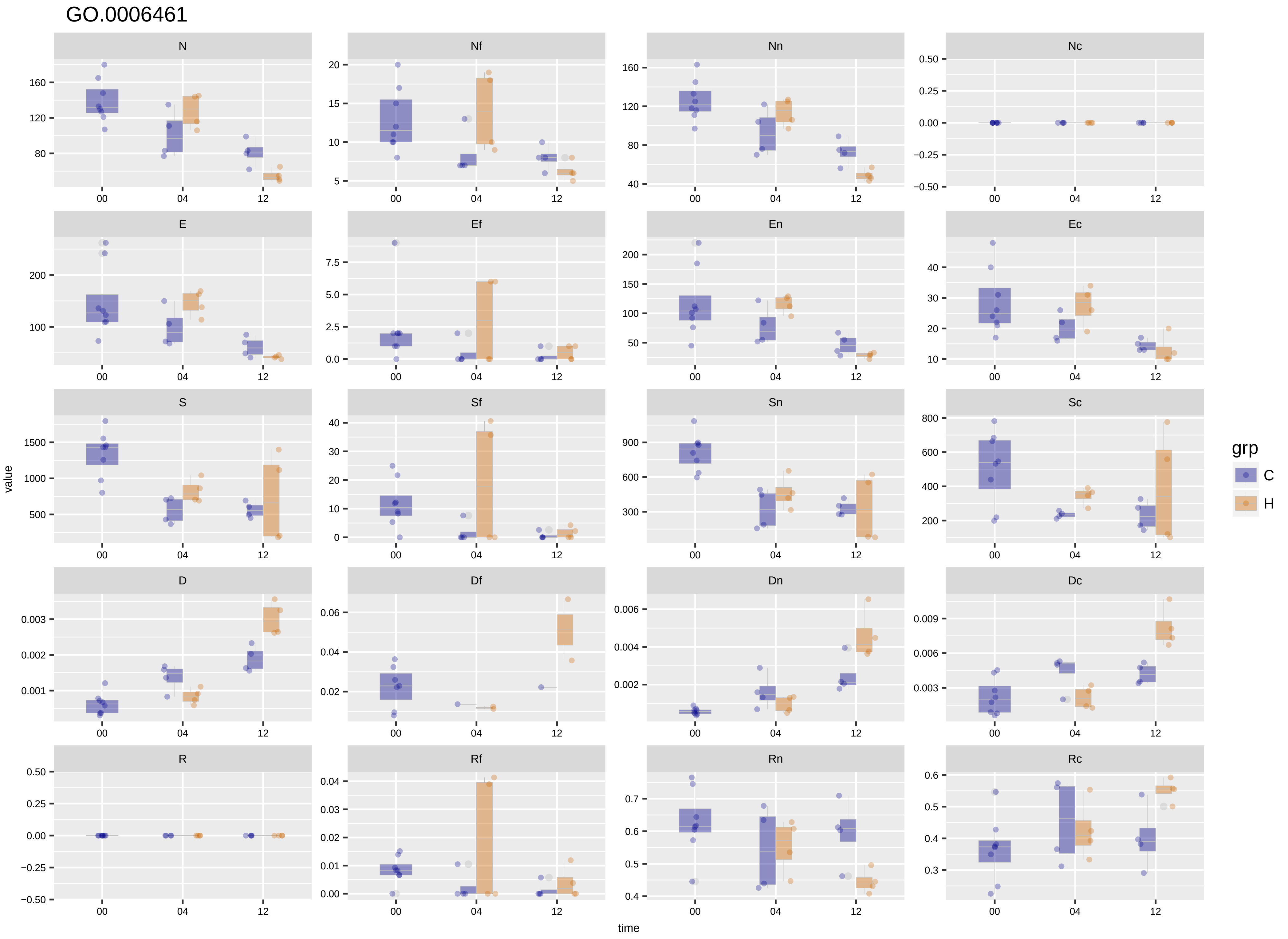
GO.0006461
N
Nf
Nn
Nc
E
Ef
En
Ec
S
Sf
Sn
Sc
D
Df
Dn
Dc
R
Rf
Rn
Rc
value
time
grp
C
H

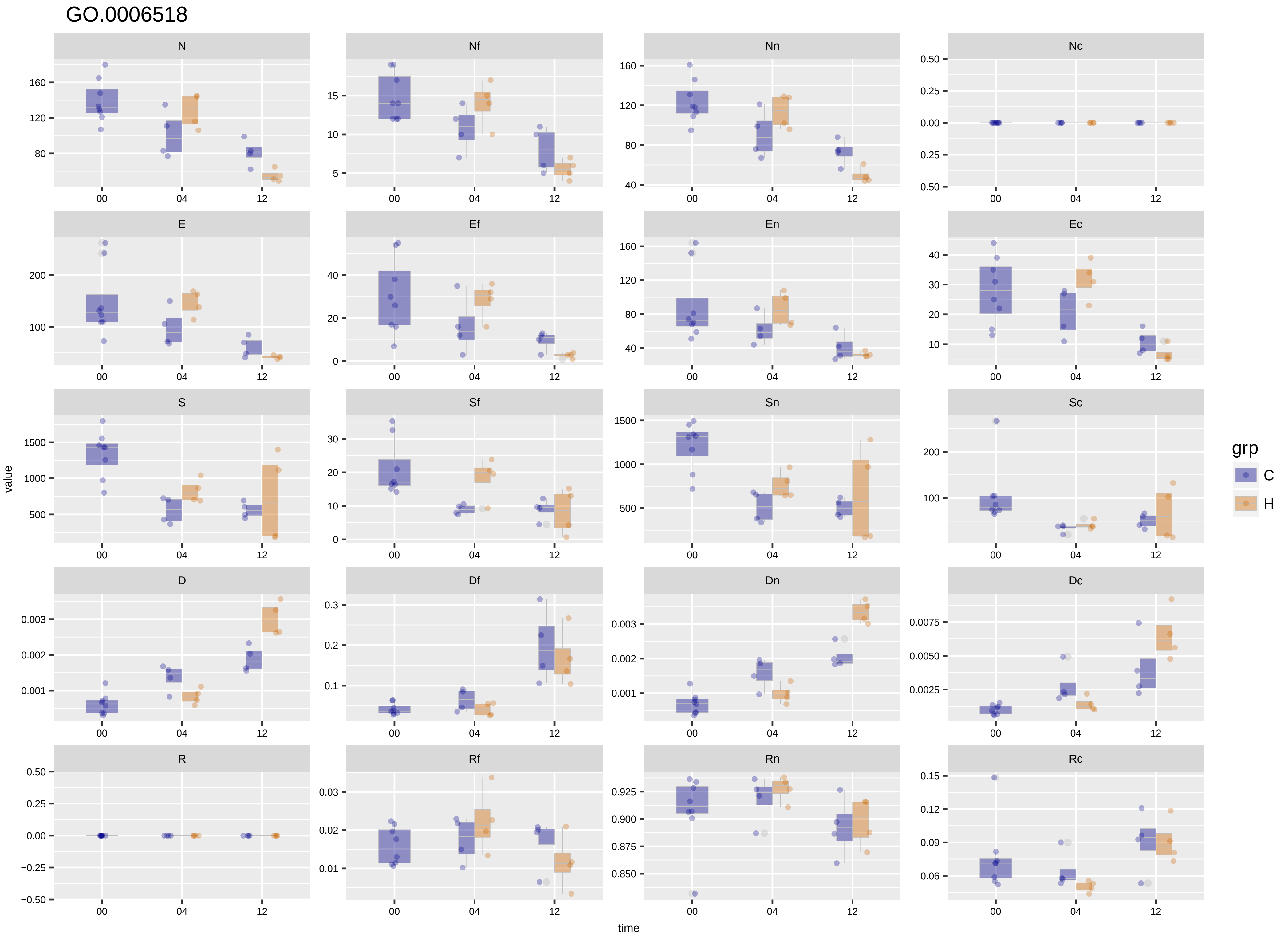

GO.0006518
N
Nf
Nn
Nc
E
Ef
En
Ec
S
Sf
Sn
Sc
D
Df
Dn
Dc
R
Rf
Rn
Rc
value
time
00
04
12
grp
C
H

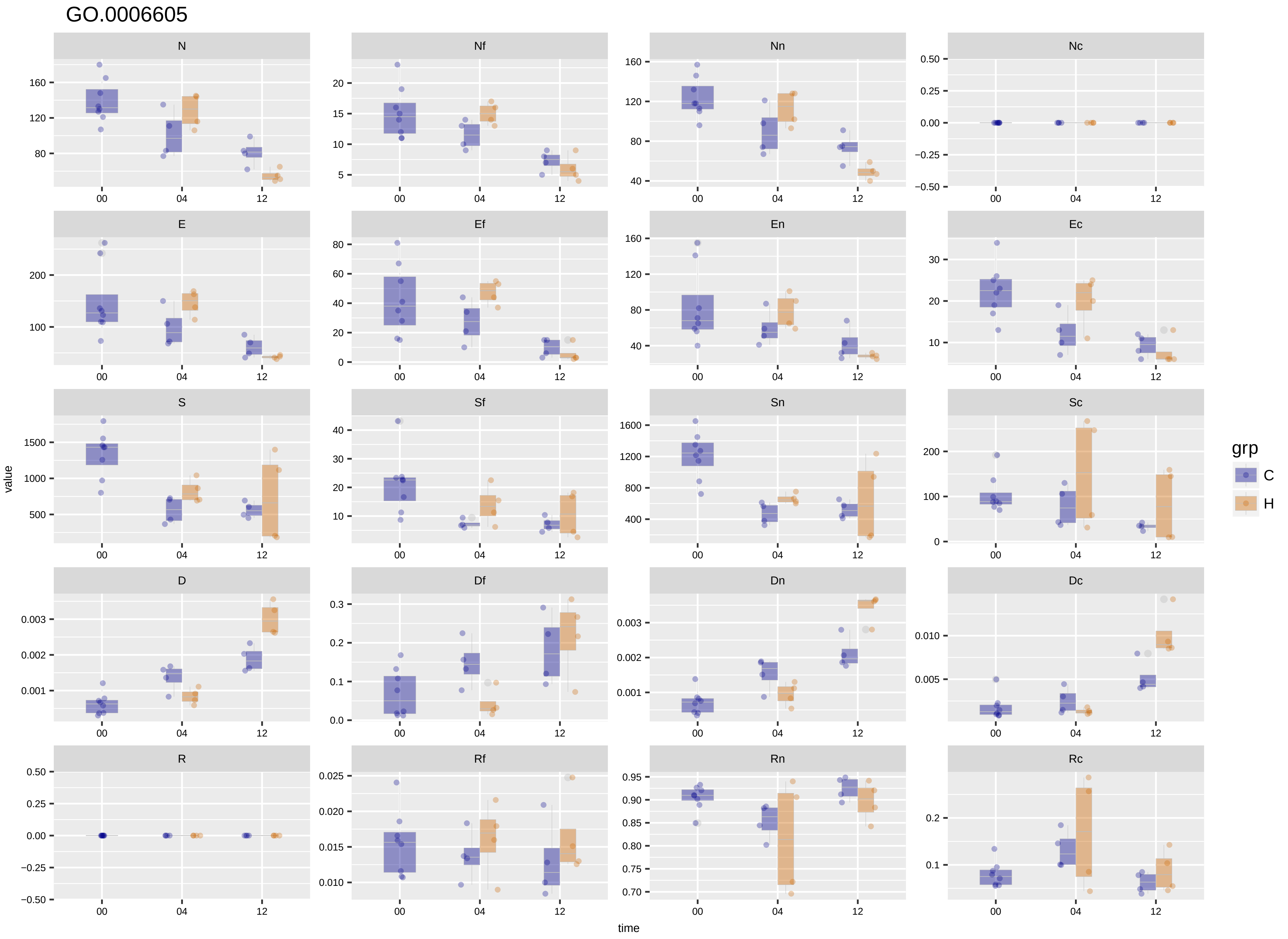

GO.0006605
N
Nf
Nn
Nc
E
Ef
En
Ec
S
Sf
Sn
Sc
D
Df
Dn
Dc
R
Rf
Rn
Rc
value
time
grp
C
H

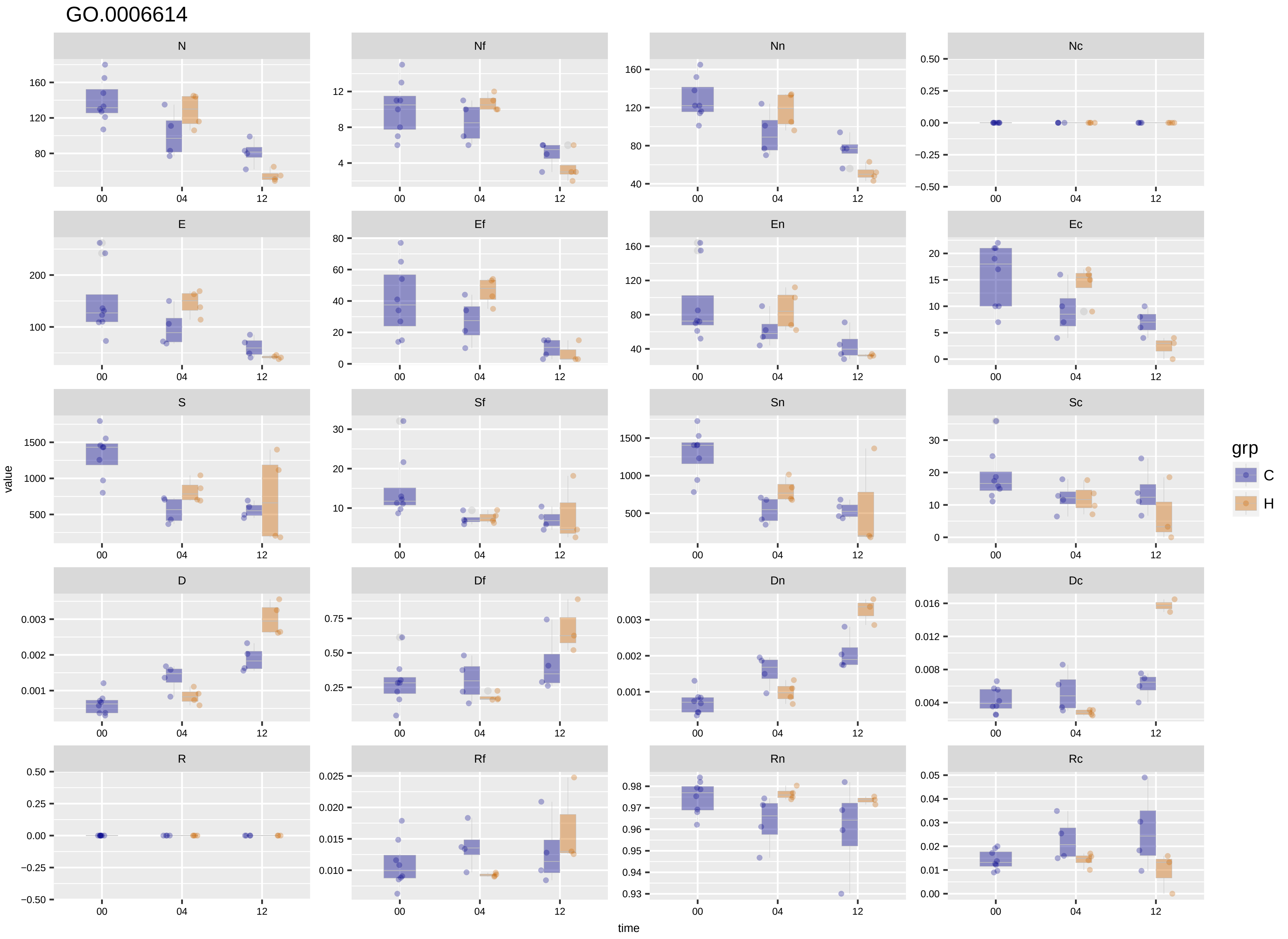

GO.0006614
N
Nf
Nn
Nc
E
Ef
En
Ec
S
Sf
Sn
Sc
D
Df
Dn
Dc
R
Rf
Rn
Rc
value
time
grp
C
H

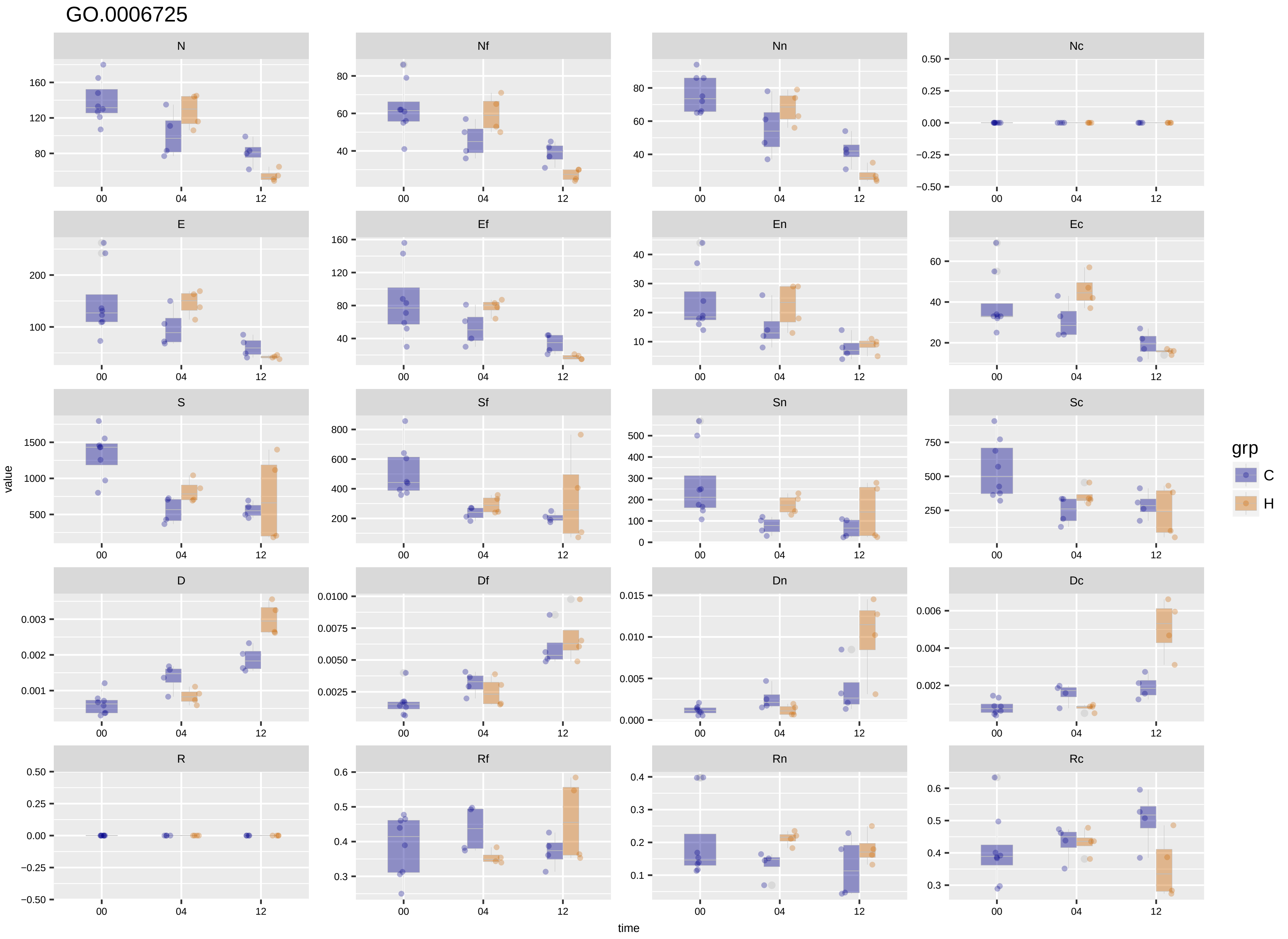
GO.0006725
N
Nf
Nn
Nc
E
Ef
En
Ec
S
Sf
Sn
Sc
D
Df
Dn
Dc
R
Rf
Rn
Rc
value
time
grp
C
H

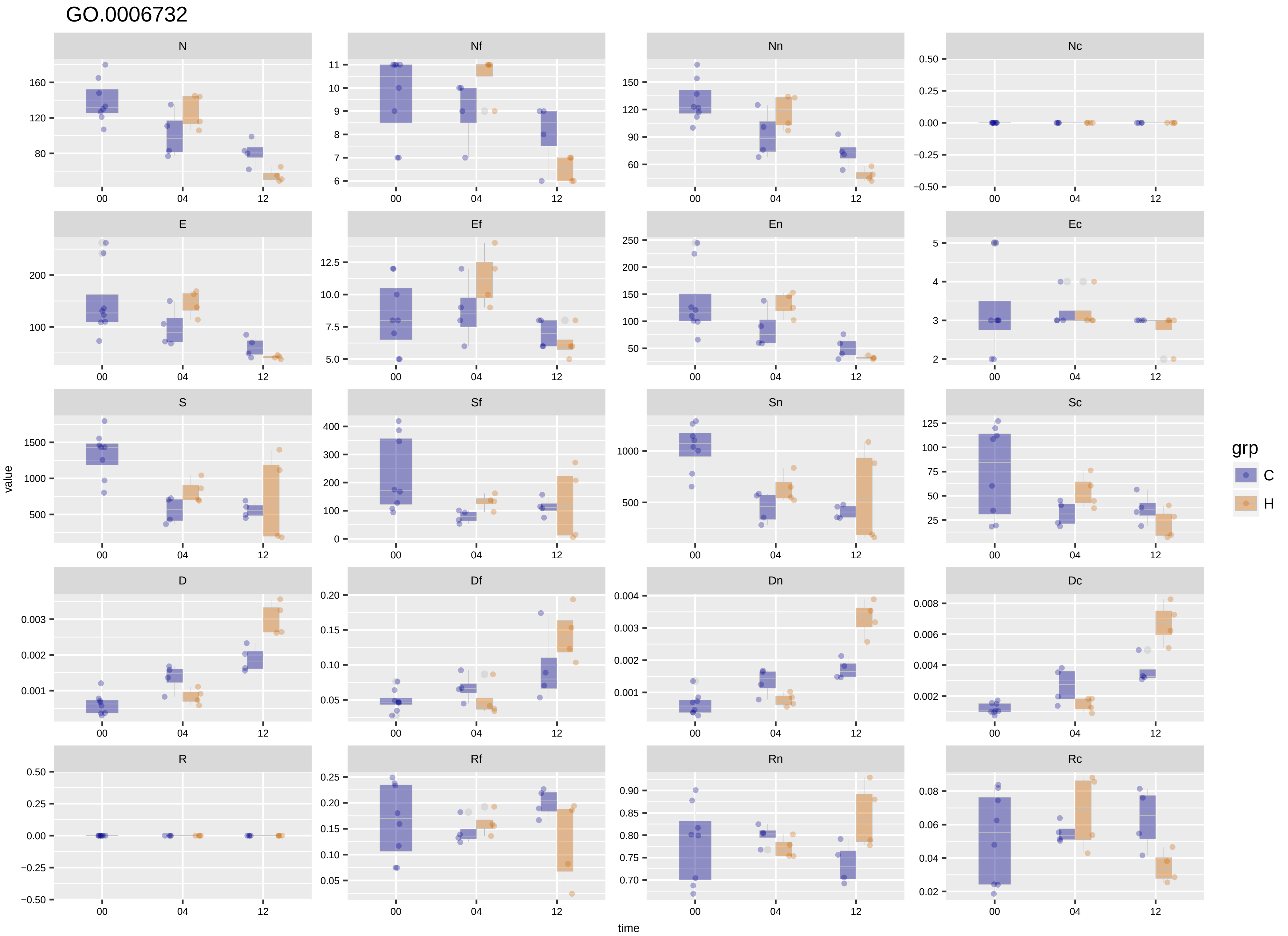

GO.0006732
N
Nf
Nn
Nc
E
Ef
En
Ec
S
Sf
Sn
Sc
D
Df
Dn
Dc
R
Rf
Rn
Rc
value
time
00
04
12
grp
C
H

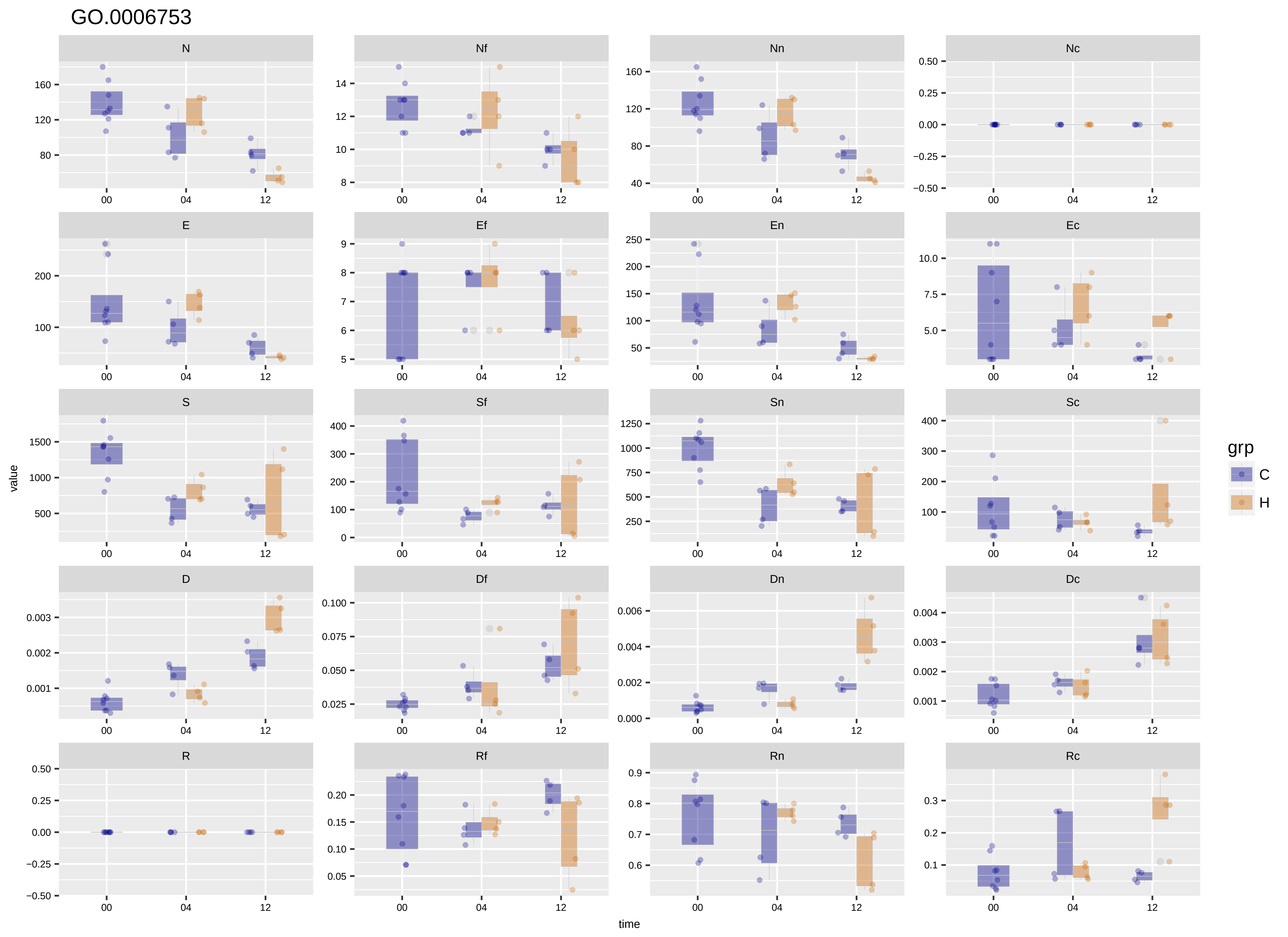

GO.0006753
N
Nf
Nn
Nc
E
Ef
En
Ec
S
Sf
Sn
Sc
D
Df
Dn
Dc
R
Rf
Rn
Rc
value
time
00
04
12
grp
C
H

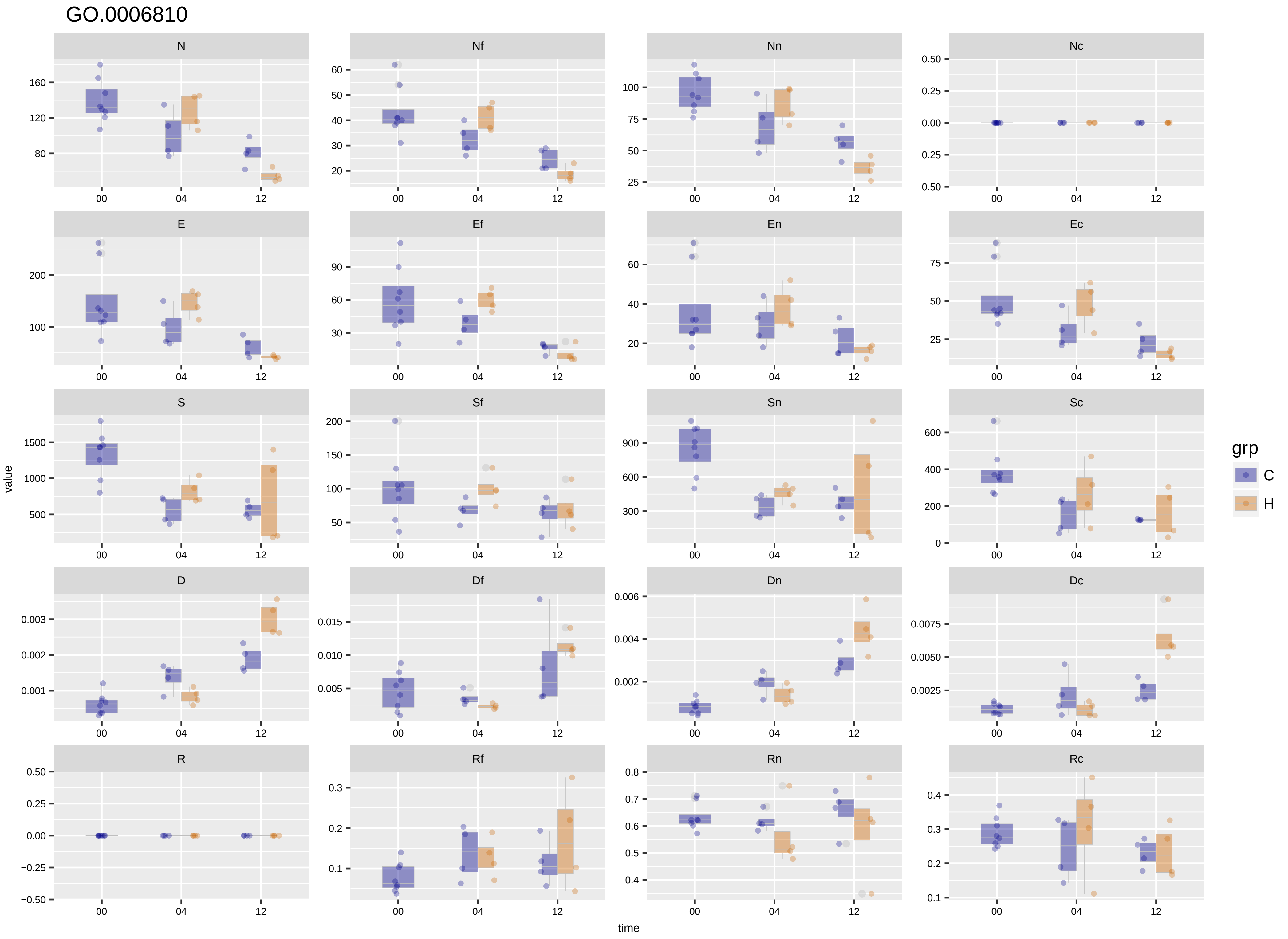
GO.0006810
N
Nf
Nn
Nc
E
Ef
En
Ec
S
Sf
Sn
Sc
D
Df
Dn
Dc
R
Rf
Rn
Rc
value
time
00
04
12
grp
C
H

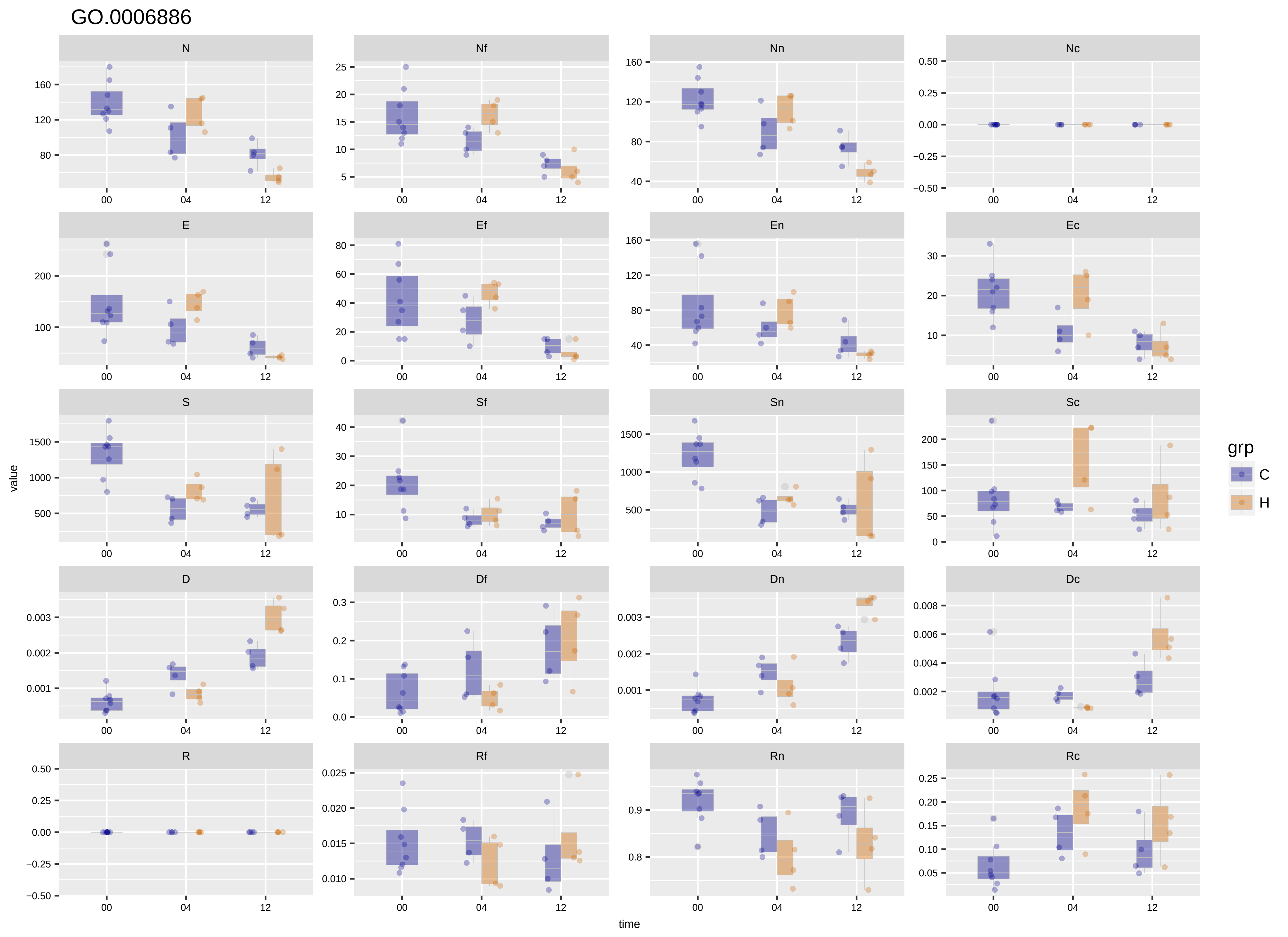

GO.0006886
N
Nf
Nn
Nc
E
Ef
En
Ec
S
Sf
Sn
Sc
D
Df
Dn
Dc
R
Rf
Rn
Rc
value
time
grp
C
H

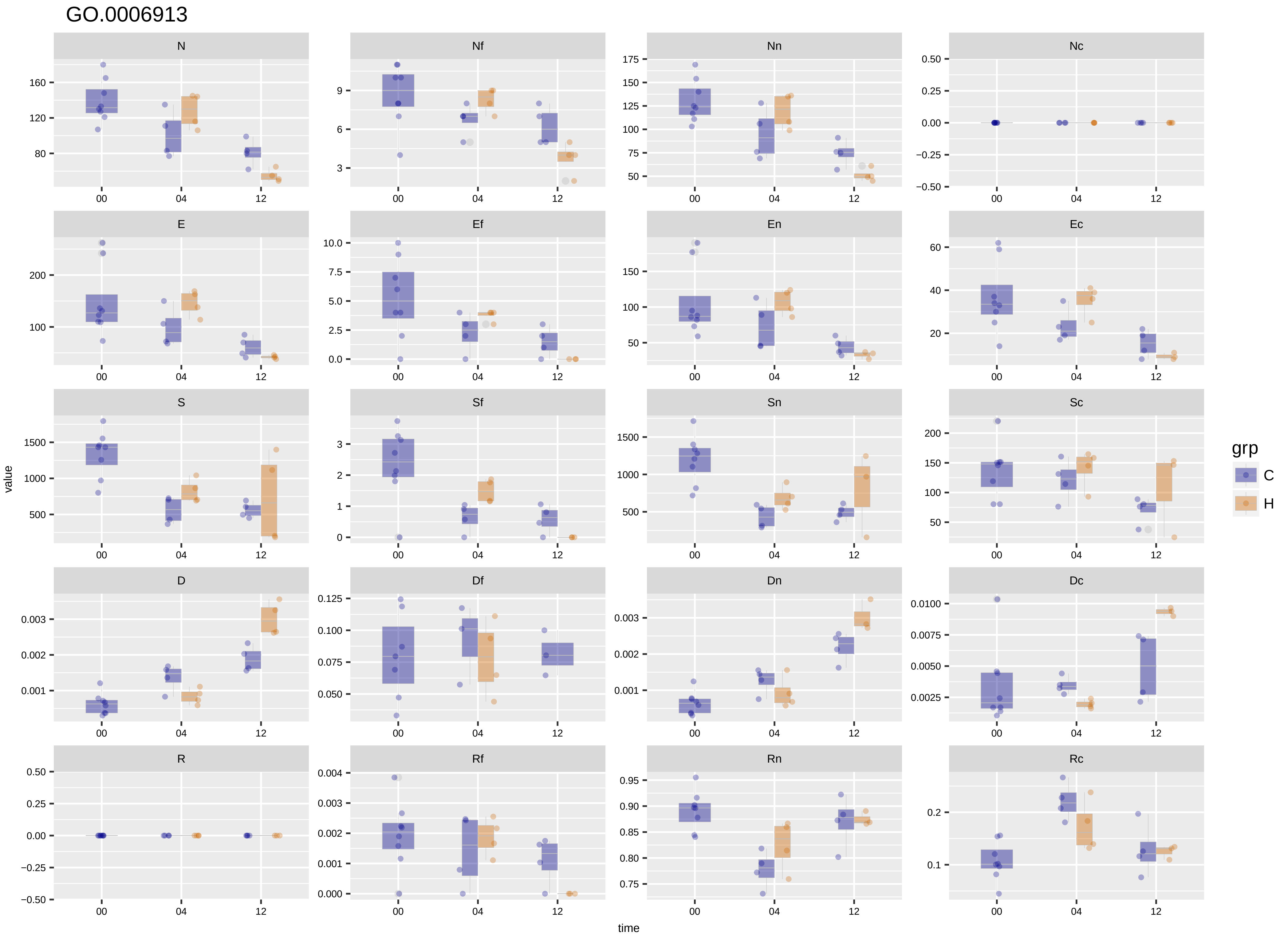
GO.0006913
N
Nf
Nn
Nc
E
Ef
En
Ec
S
Sf
Sn
Sc
D
Df
Dn
Dc
R
Rf
Rn
Rc
value
time
grp
C
H

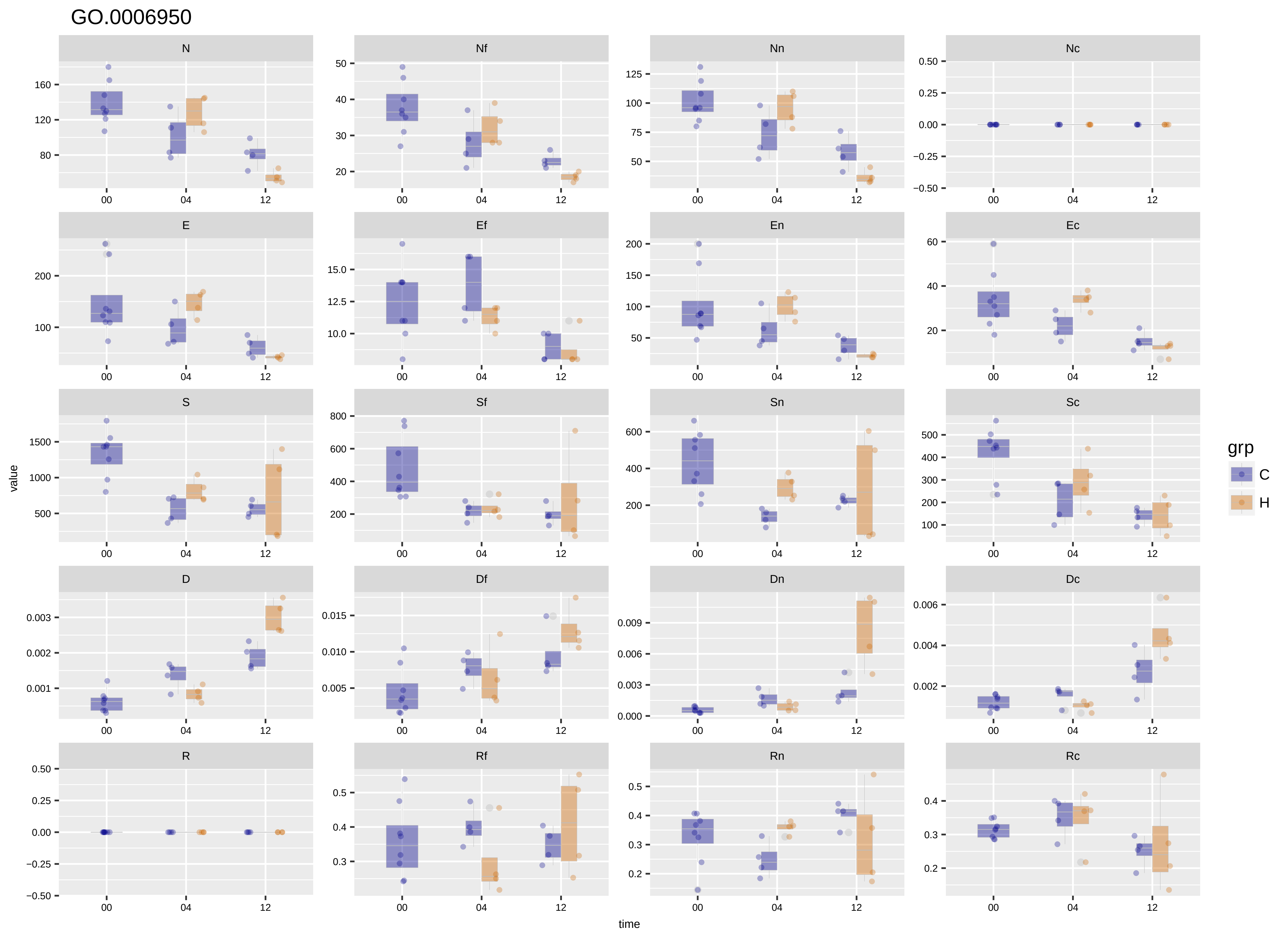

GO.0006950
N
Nf
Nn
Nc
E
Ef
En
Ec
S
Sf
Sn
Sc
D
Df
Dn
Dc
R
Rf
Rn
Rc
00
04
12
value
time
grp
C
H

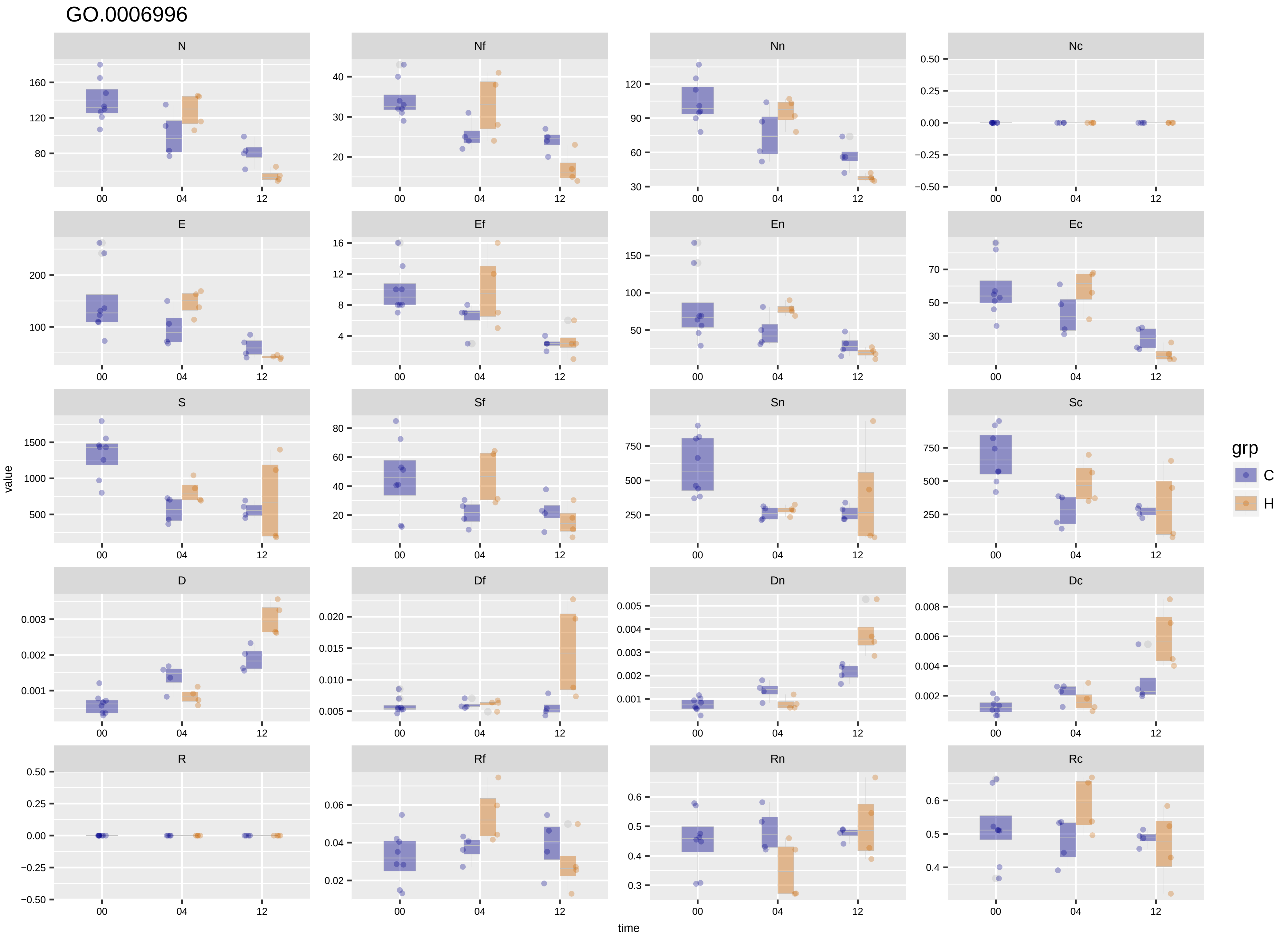

GO.0006996
N
Nf
Nn
Nc
E
Ef
En
Ec
S
Sf
Sn
Sc
D
Df
Dn
Dc
R
Rf
Rn
Rc
value
time
00
04
12
grp
C
H

GO.0008104

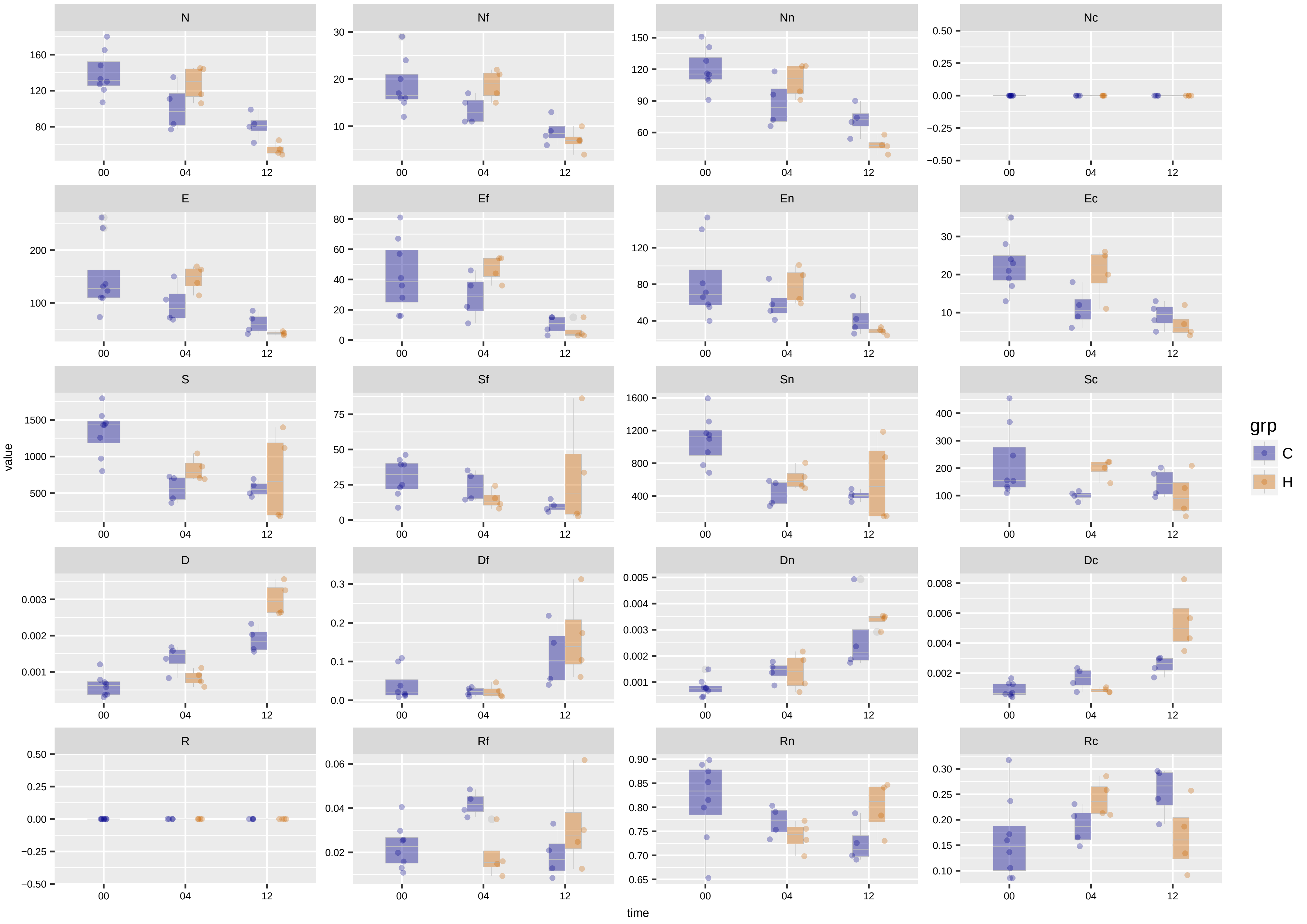

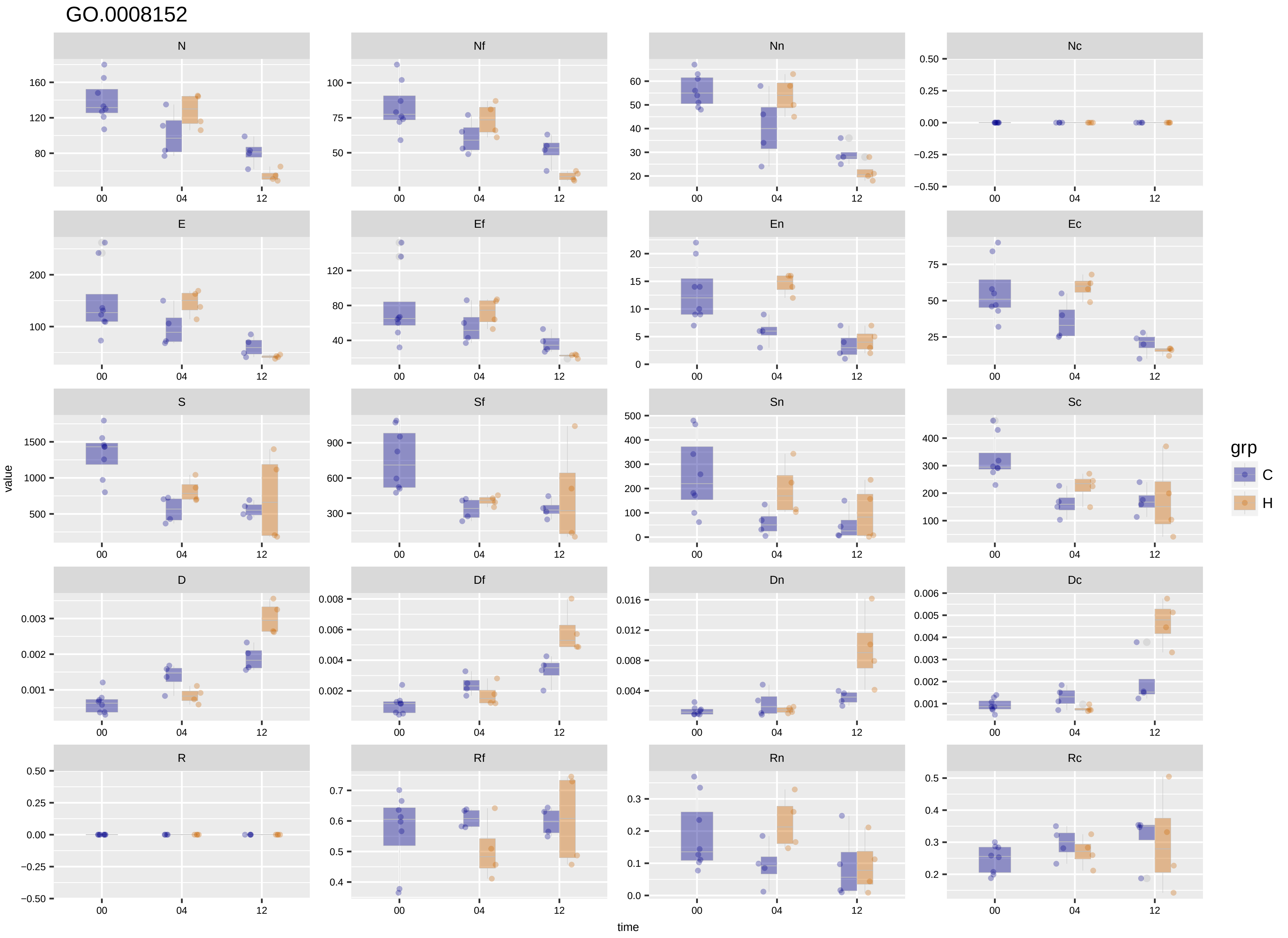

GO.0008152
N
Nf
Nn
Nc
E
Ef
En
Ec
S
Sf
Sn
Sc
D
Df
Dn
Dc
R
Rf
Rn
Rc
value
time
grp
C
H
00
04
12

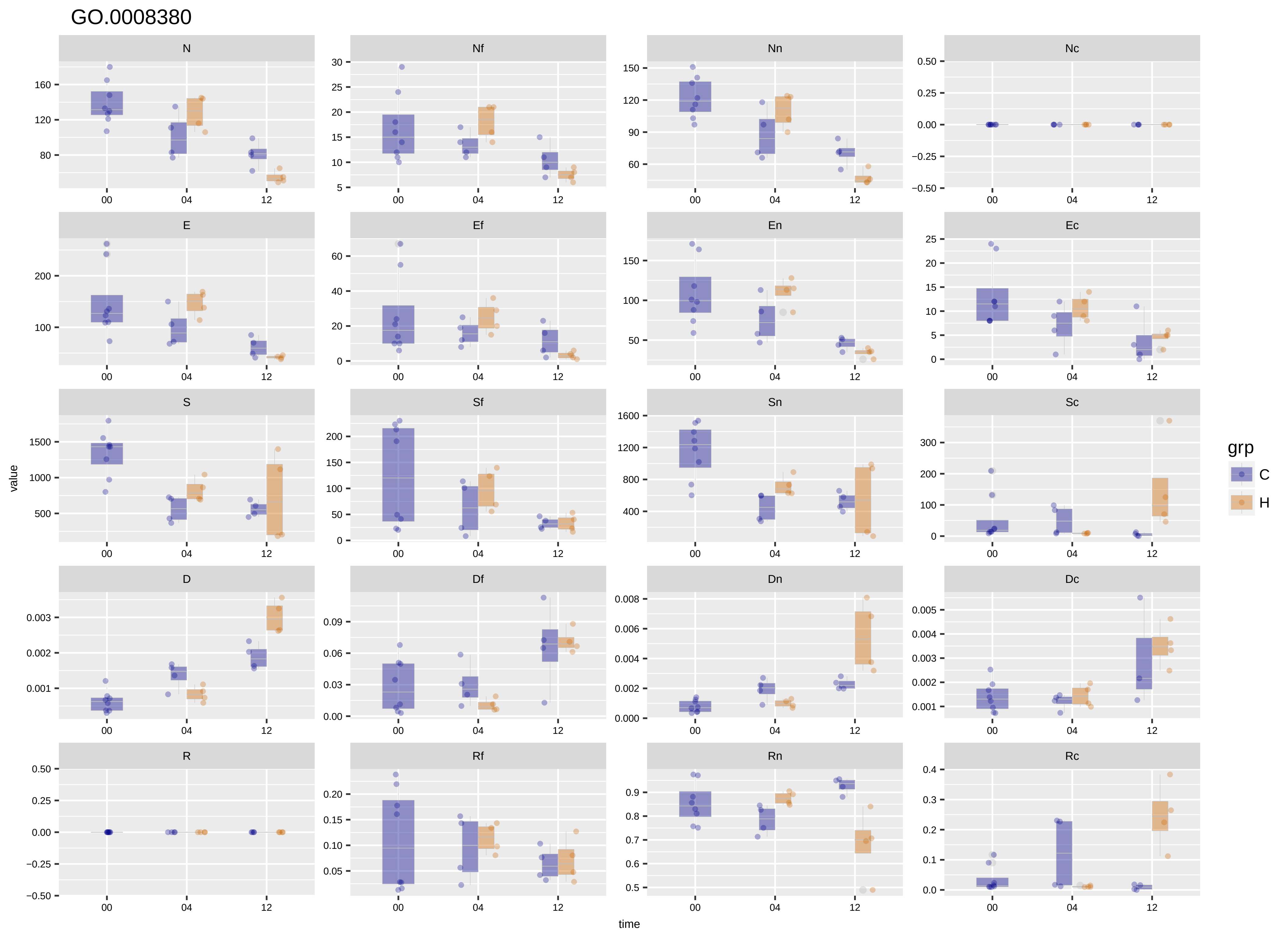

GO.0008380
N
Nf
Nn
Nc
E
Ef
En
Ec
S
Sf
Sn
Sc
D
Df
Dn
Dc
R
Rf
Rn
Rc
value
time
grp
C
H

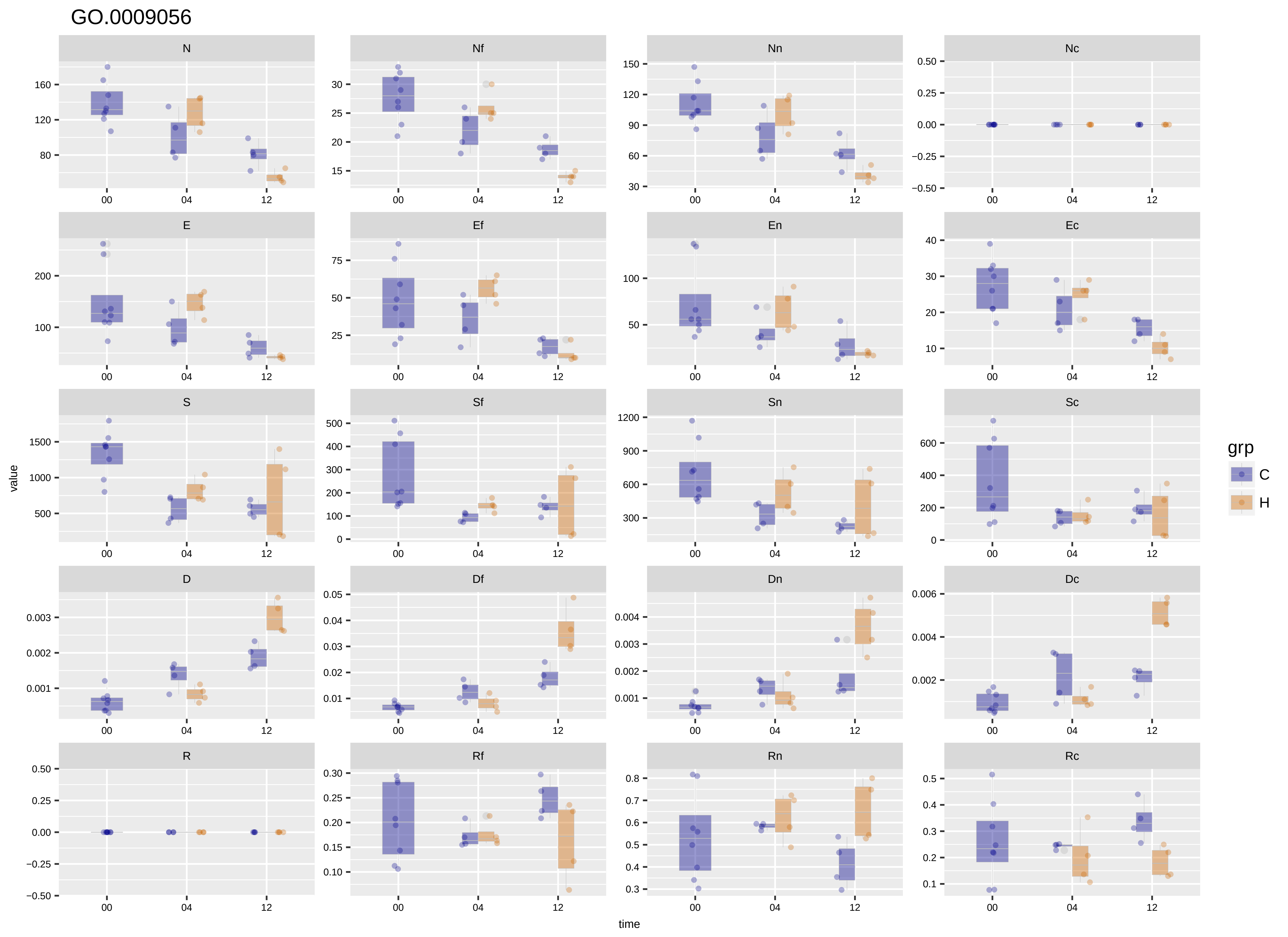

GO.0009056
N
Nf
Nn
Nc
E
Ef
En
Ec
S
Sf
Sn
Sc
D
Df
Dn
Dc
R
Rf
Rn
Rc
value
time
grp
C
H
00
04
12

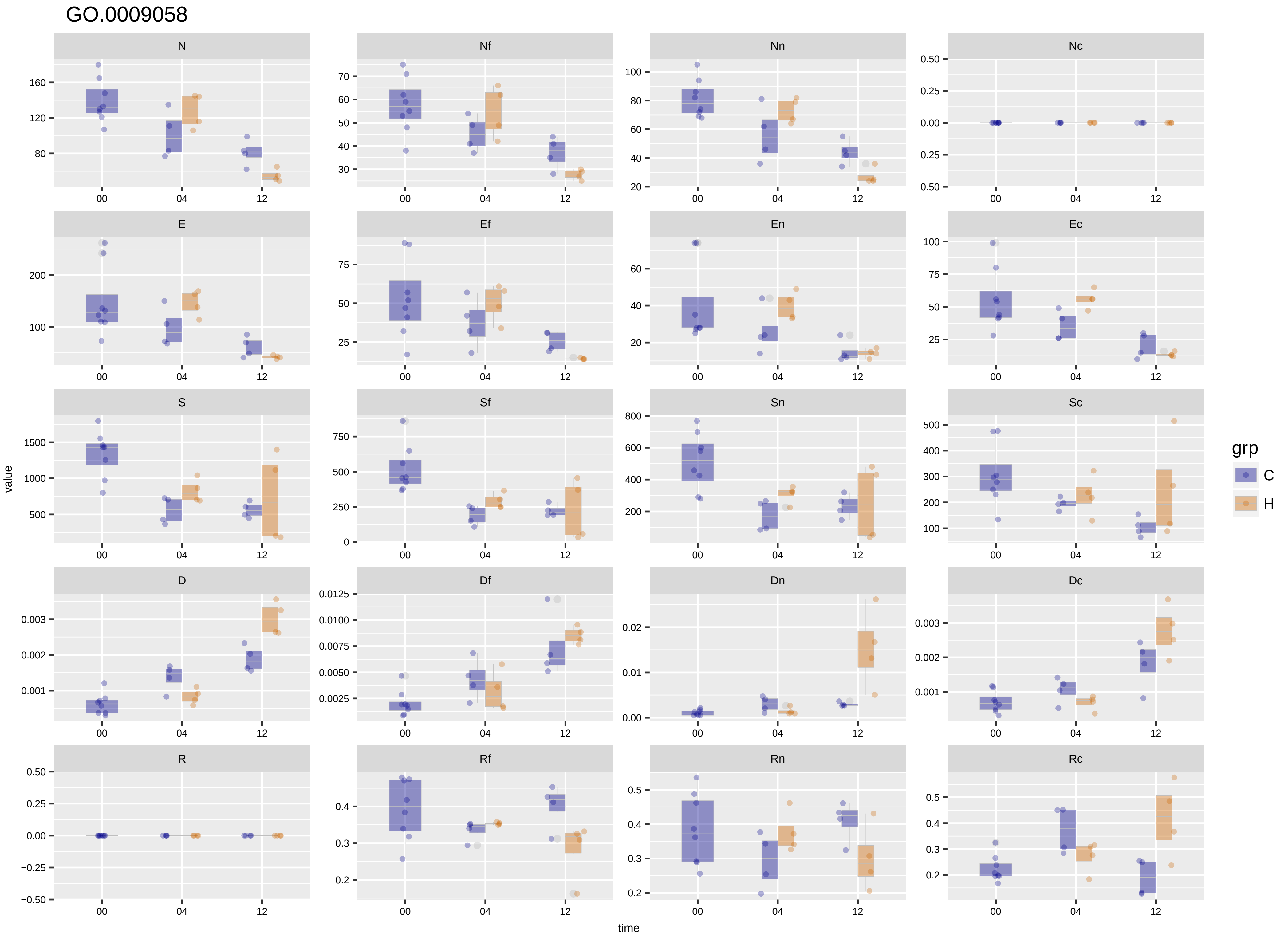

GO.0009058
N
Nf
Nn
Nc
E
Ef
En
Ec
S
Sf
Sn
Sc
D
Df
Dn
Dc
R
Rf
Rn
Rc
value
time
grp
C
H
00
04
12

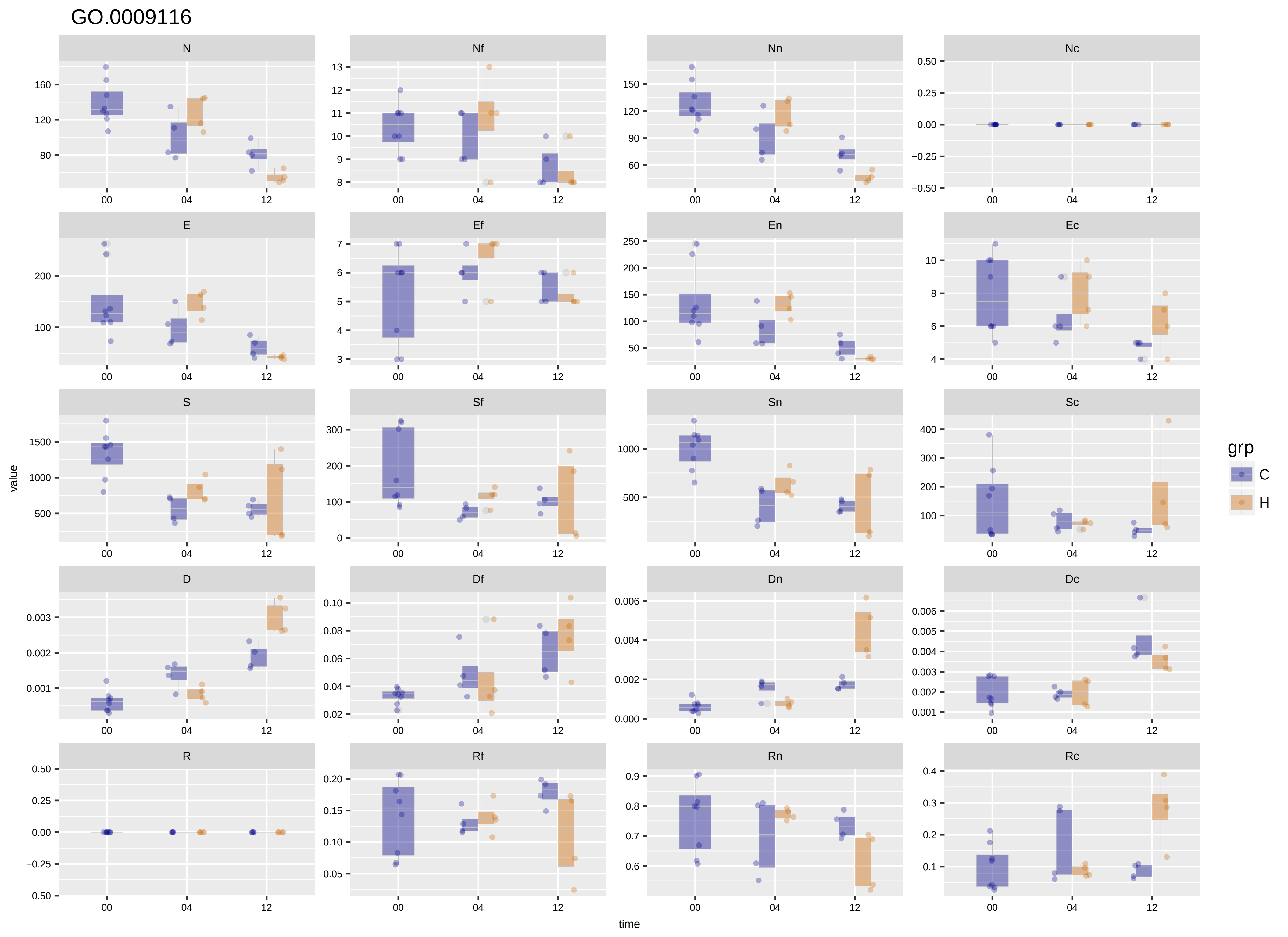
GO.0009116
N
Nf
Nn
Nc
E
Ef
En
Ec
S
Sf
Sn
Sc
D
Df
Dn
Dc
R
Rf
Rn
Rc
value
time
grp
C
H

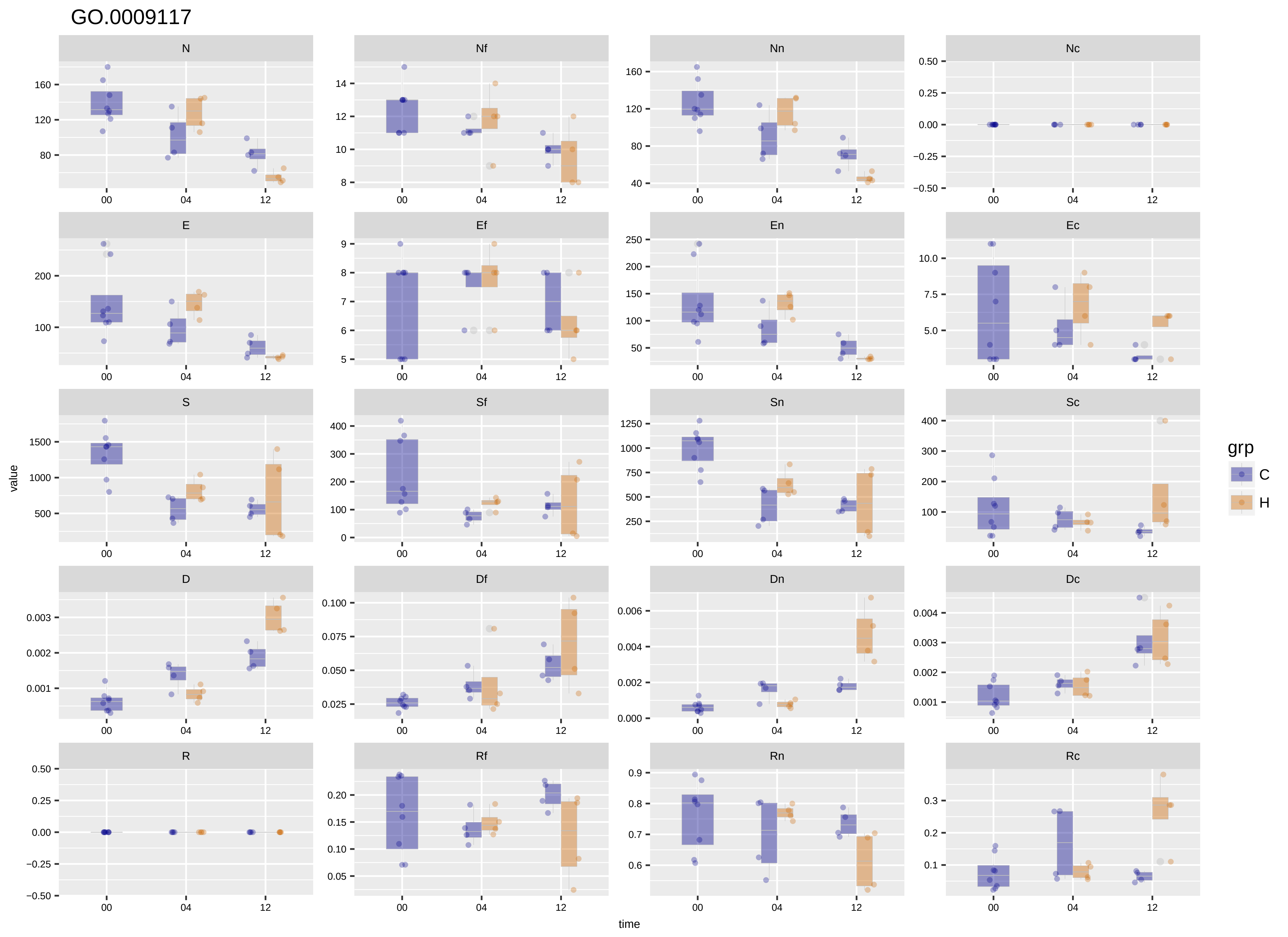

GO.0009117
N
Nf
Nn
Nc
E
Ef
En
Ec
S
Sf
Sn
Sc
D
Df
Dn
Dc
R
Rf
Rn
Rc
value
time
grp
C
H

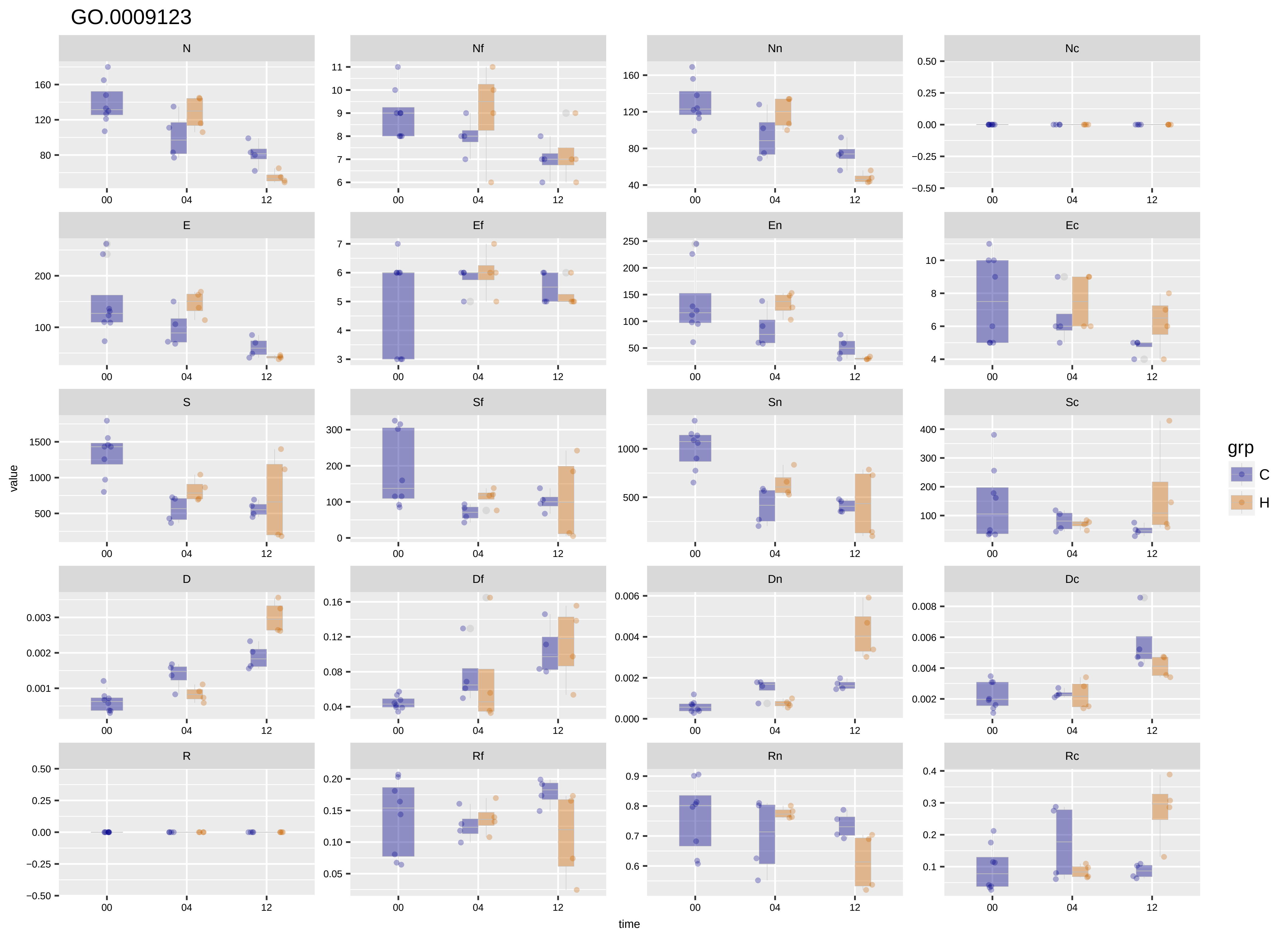
GO.0009123
N
Nf
Nn
Nc
E
Ef
En
Ec
S
Sf
Sn
Sc
D
Df
Dn
Dc
R
Rf
Rn
Rc
value
time
00
04
12
grp
C
H

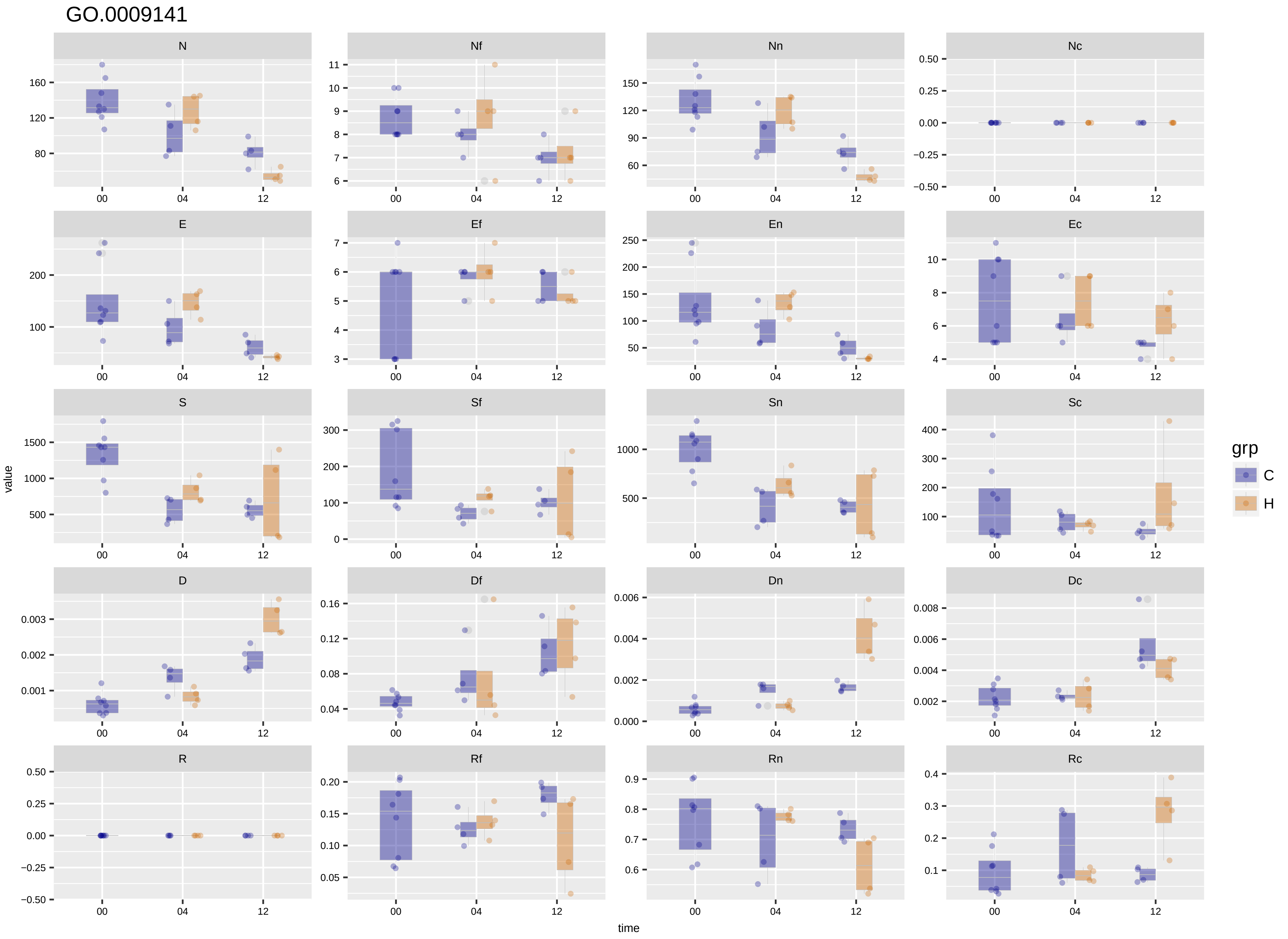
GO.0009141
N
Nf
Nn
Nc
E
Ef
En
Ec
S
Sf
Sn
Sc
D
Df
Dn
Dc
R
Rf
Rn
Rc
00
04
12
value
time
grp
C
H

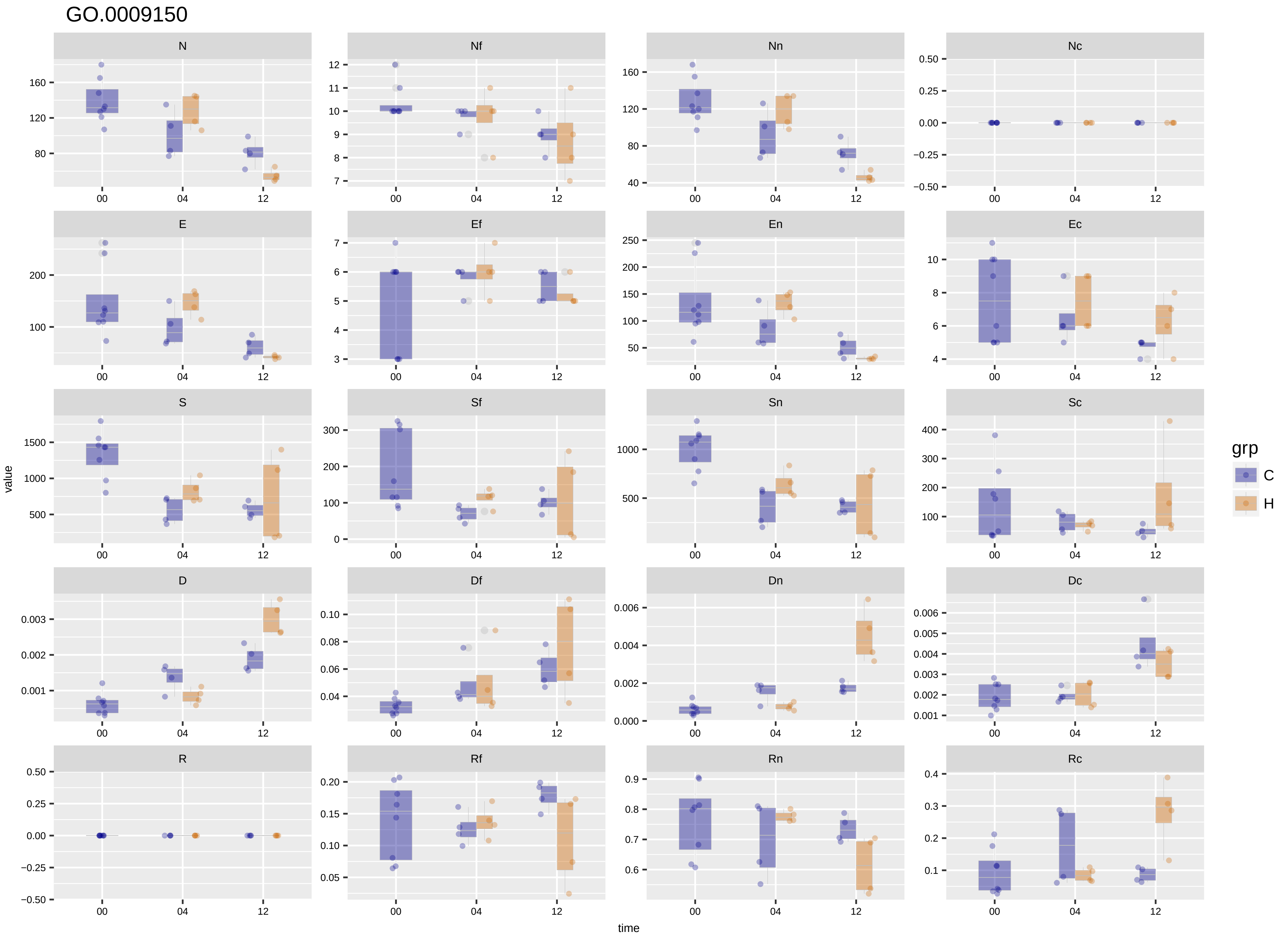
GO.0009150
N
Nf
Nn
Nc
E
Ef
En
Ec
S
Sf
Sn
Sc
D
Df
Dn
Dc
R
Rf
Rn
Rc
00
04
12
value
time
grp
C
H

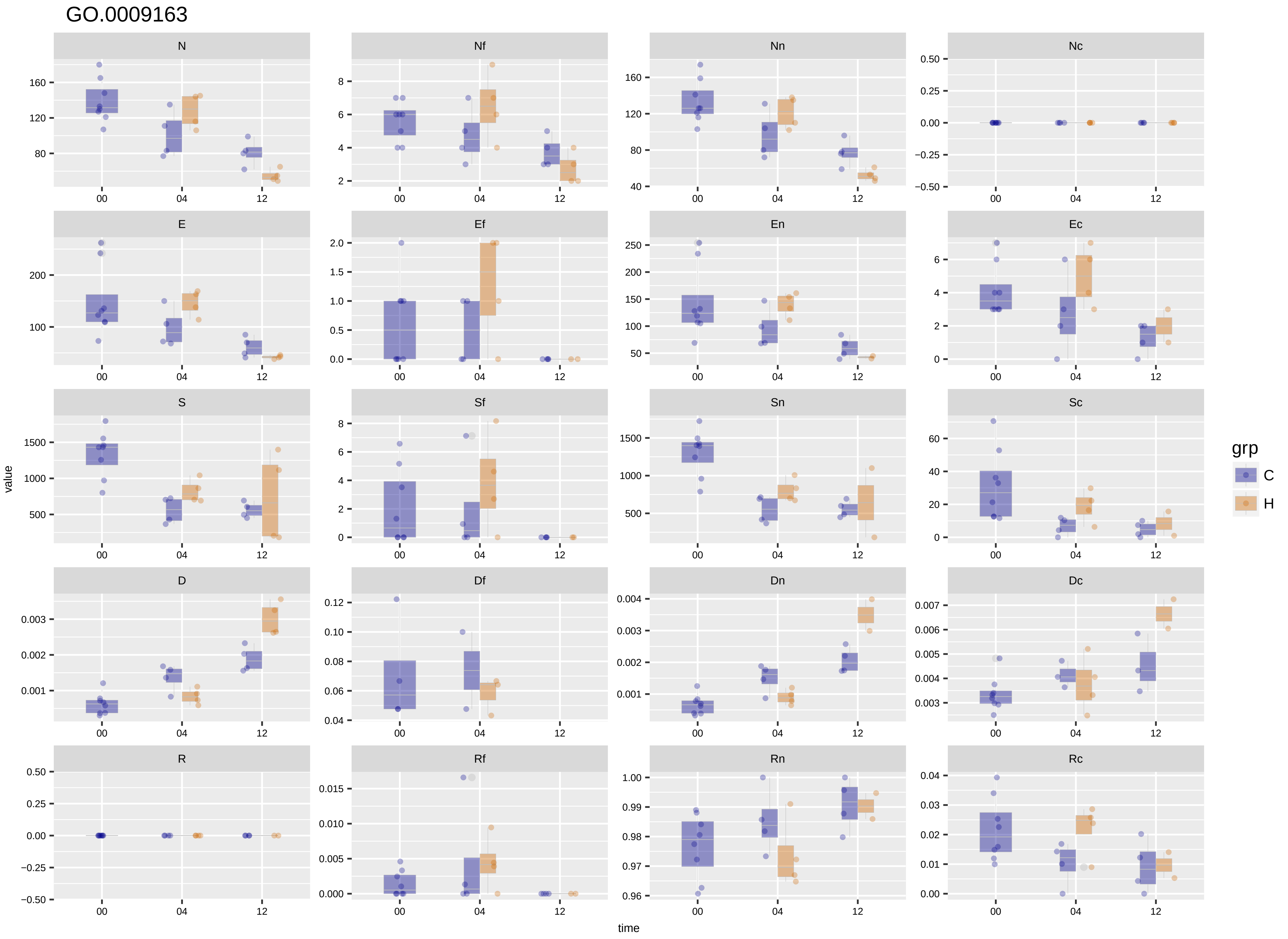
GO.0009163
N
Nf
Nn
Nc
E
Ef
En
Ec
S
Sf
Sn
Sc
D
Df
Dn
Dc
R
Rf
Rn
Rc
value
time
grp
C
H

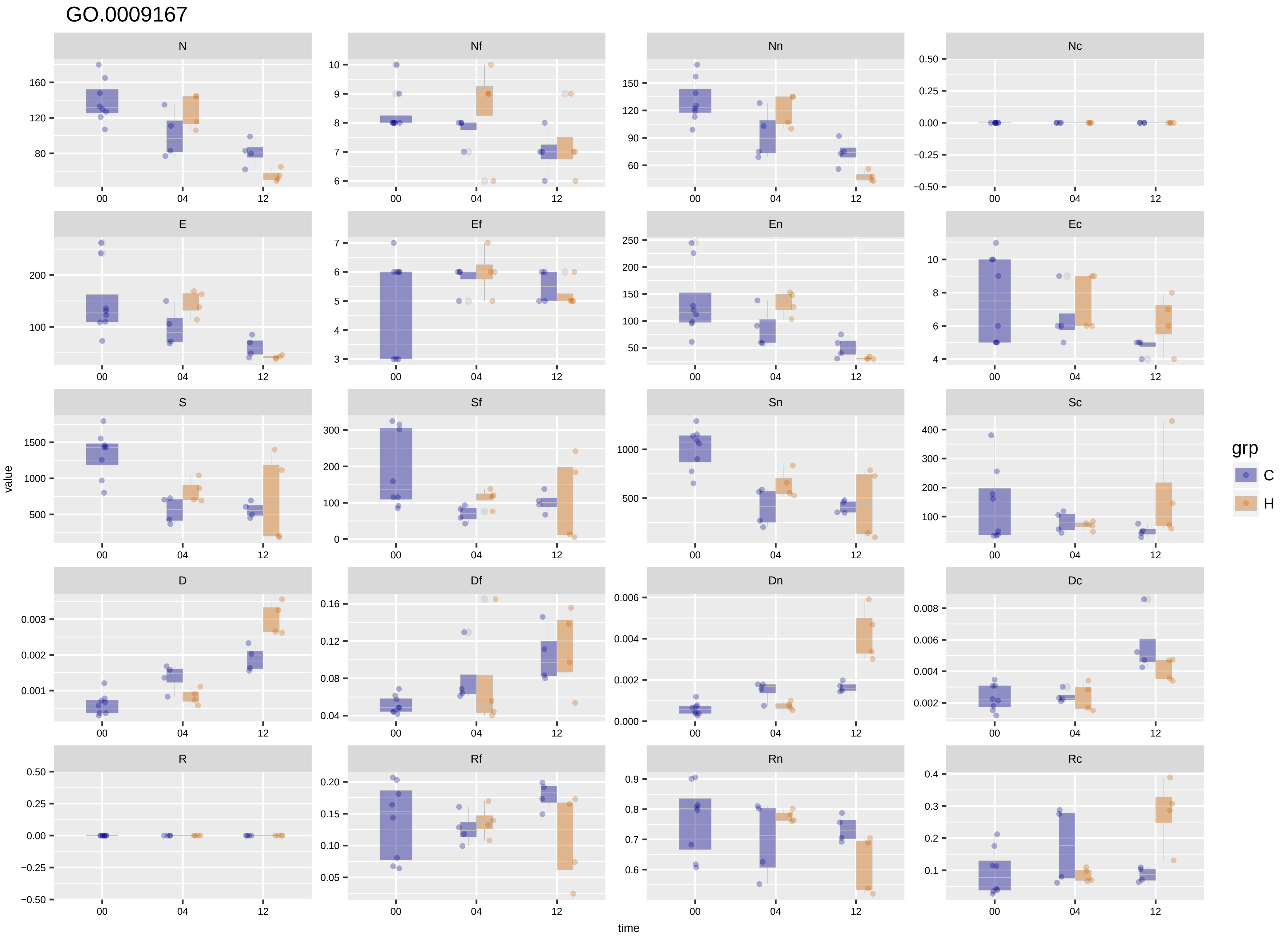

GO.0009167
N
Nf
Nn
Nc
E
Ef
En
Ec
S
Sf
Sn
Sc
D
Df
Dn
Dc
R
Rf
Rn
Rc
value
time
grp
C
H

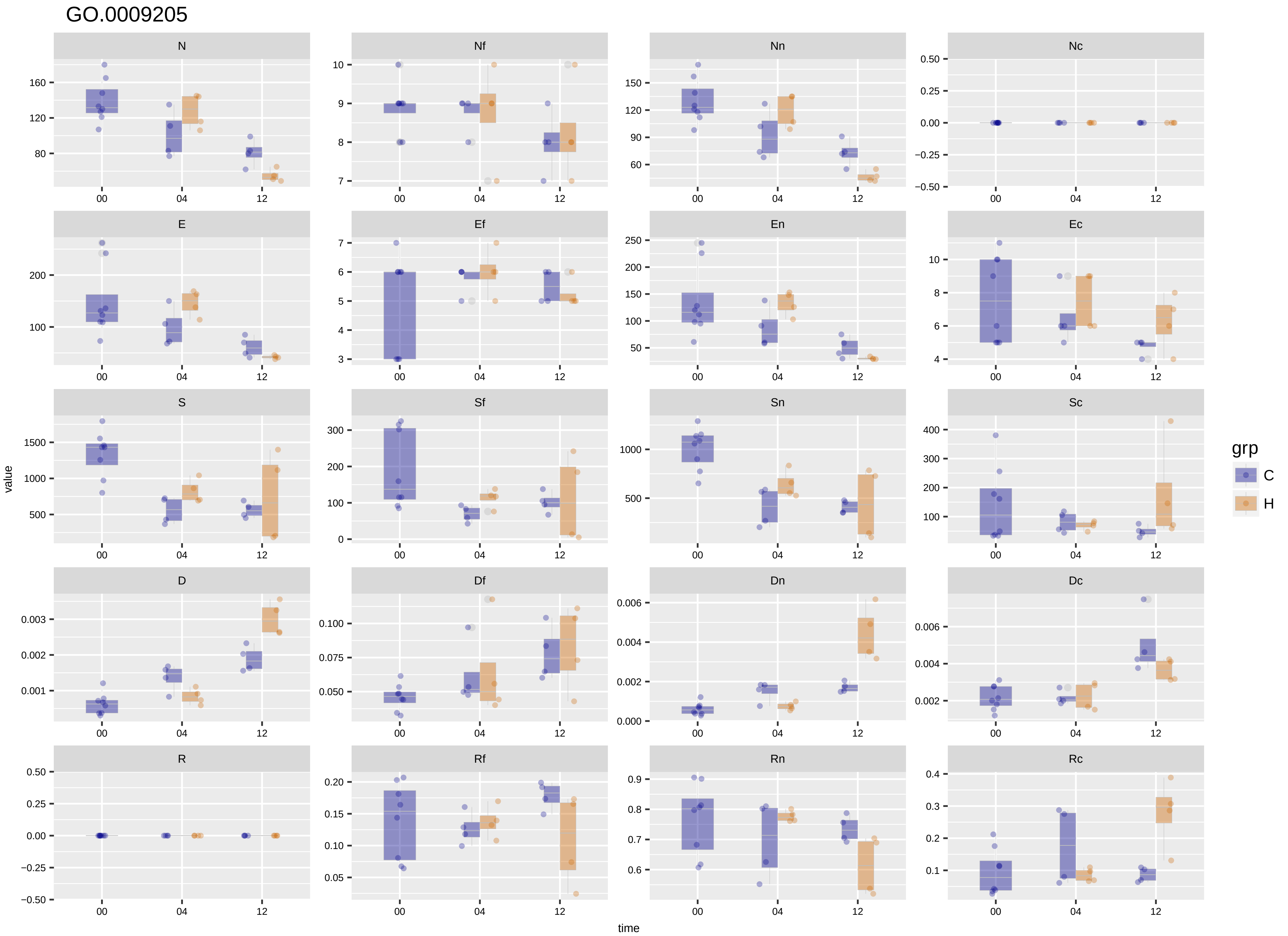
GO.0009205
N
Nf
Nn
Nc
E
Ef
En
Ec
S
Sf
Sn
Sc
D
Df
Dn
Dc
R
Rf
Rn
Rc
00
04
12
value
time
grp
C
H

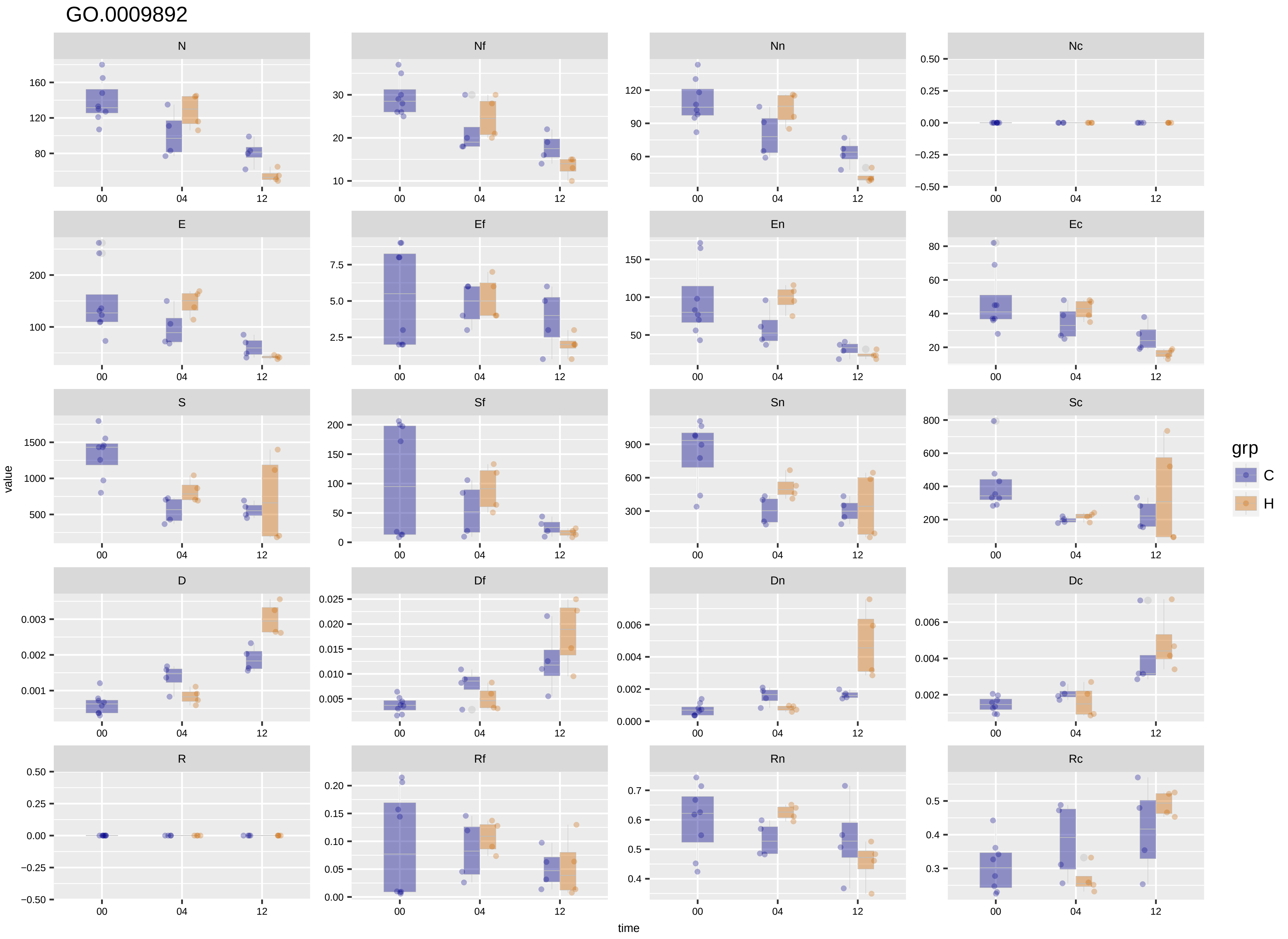

GO.0009892
N
Nf
Nn
Nc
E
Ef
En
Ec
S
Sf
Sn
Sc
D
Df
Dn
Dc
R
Rf
Rn
Rc
value
time
grp
C
H

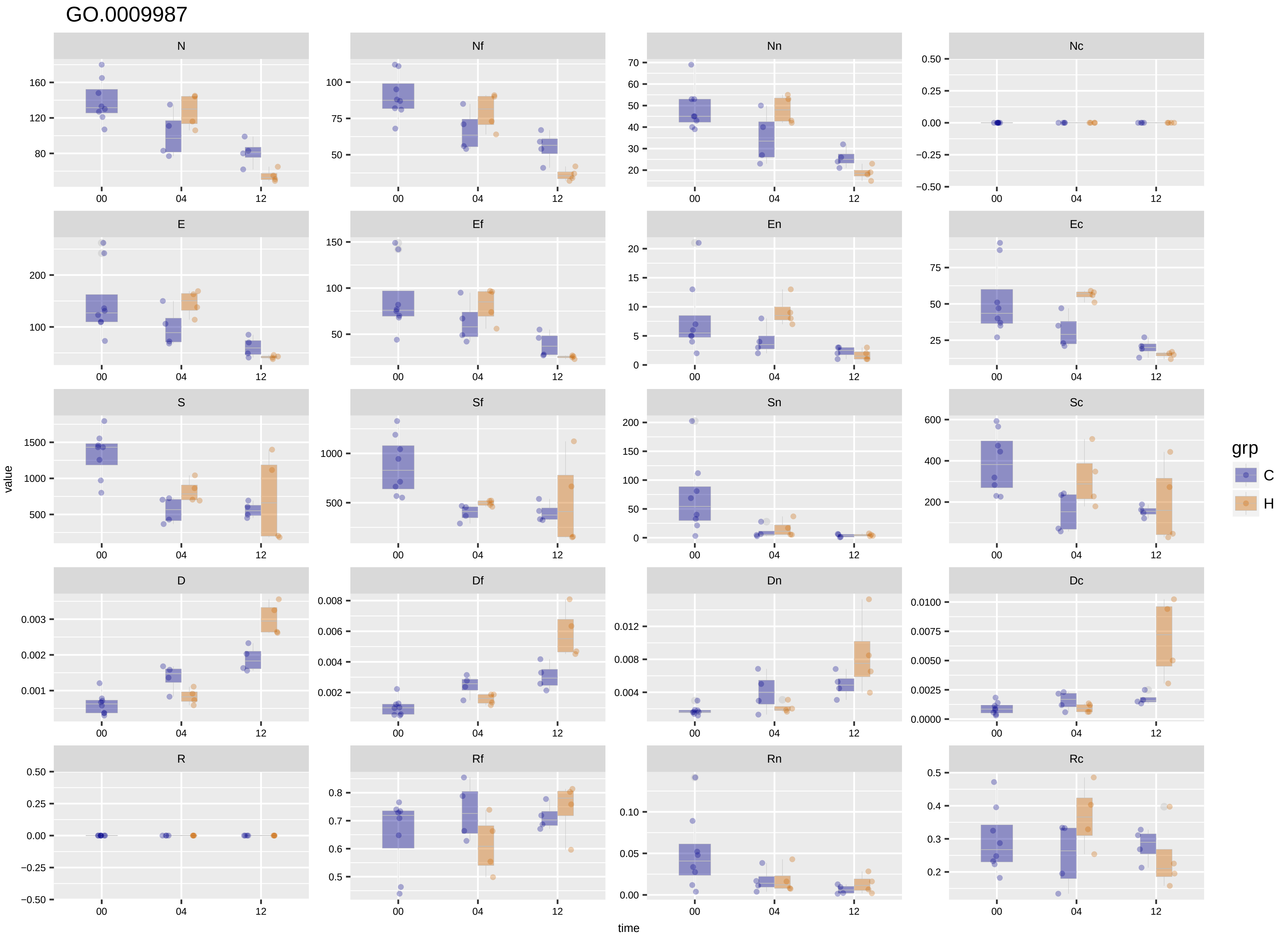

GO.0009987
N
Nf
Nn
Nc
E
Ef
En
Ec
S
Sf
Sn
Sc
D
Df
Dn
Dc
R
Rf
Rn
Rc
00
04
12
value
time
grp
C
H

GO.0010467

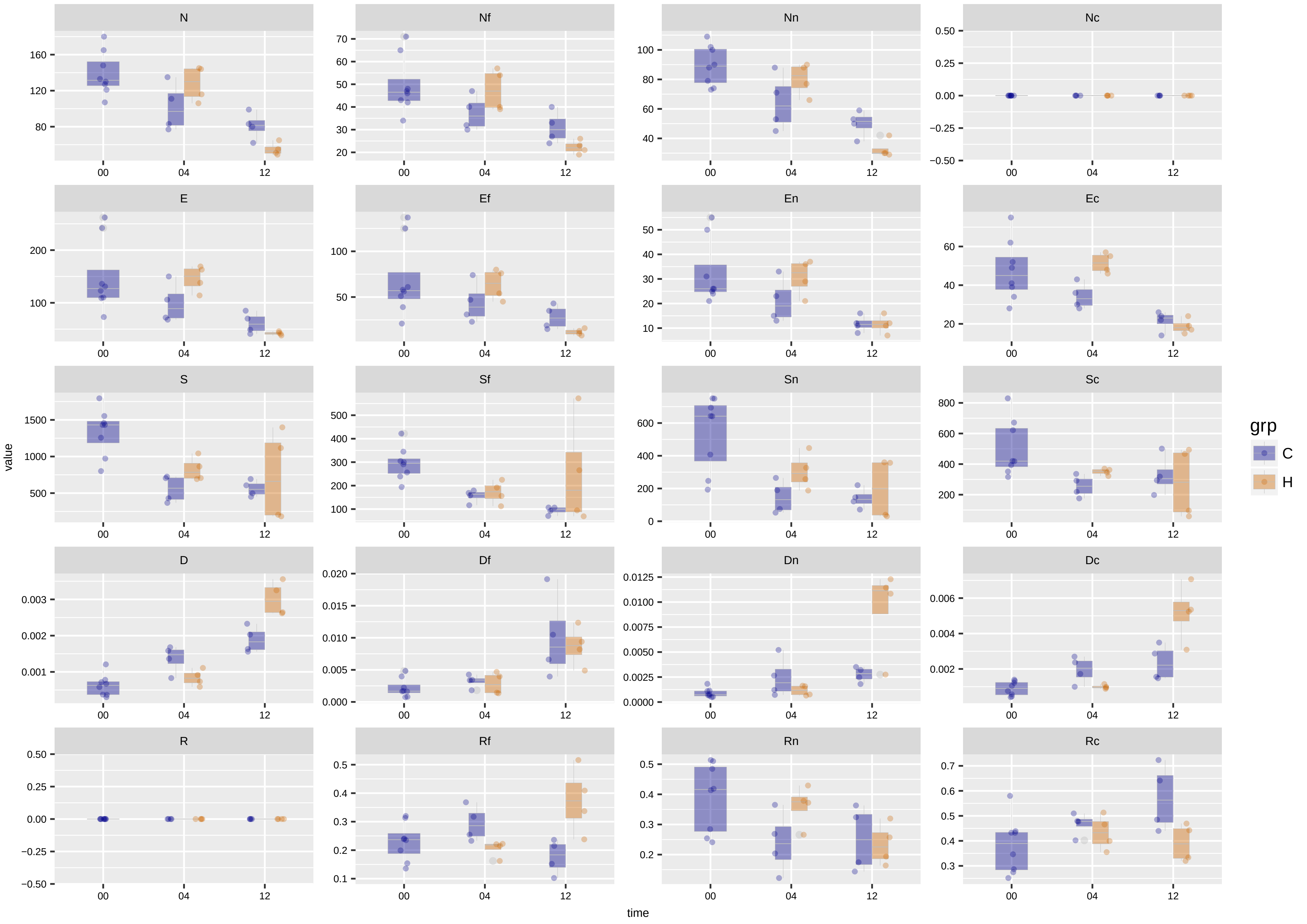

N
Nf
Nn
Nc
E
Ef
En
Ec
S
Sf
Sn
Sc
D
Df
Dn
Dc
R
Rf
Rn
Rc
value
time
grp
C
H
00
04
12

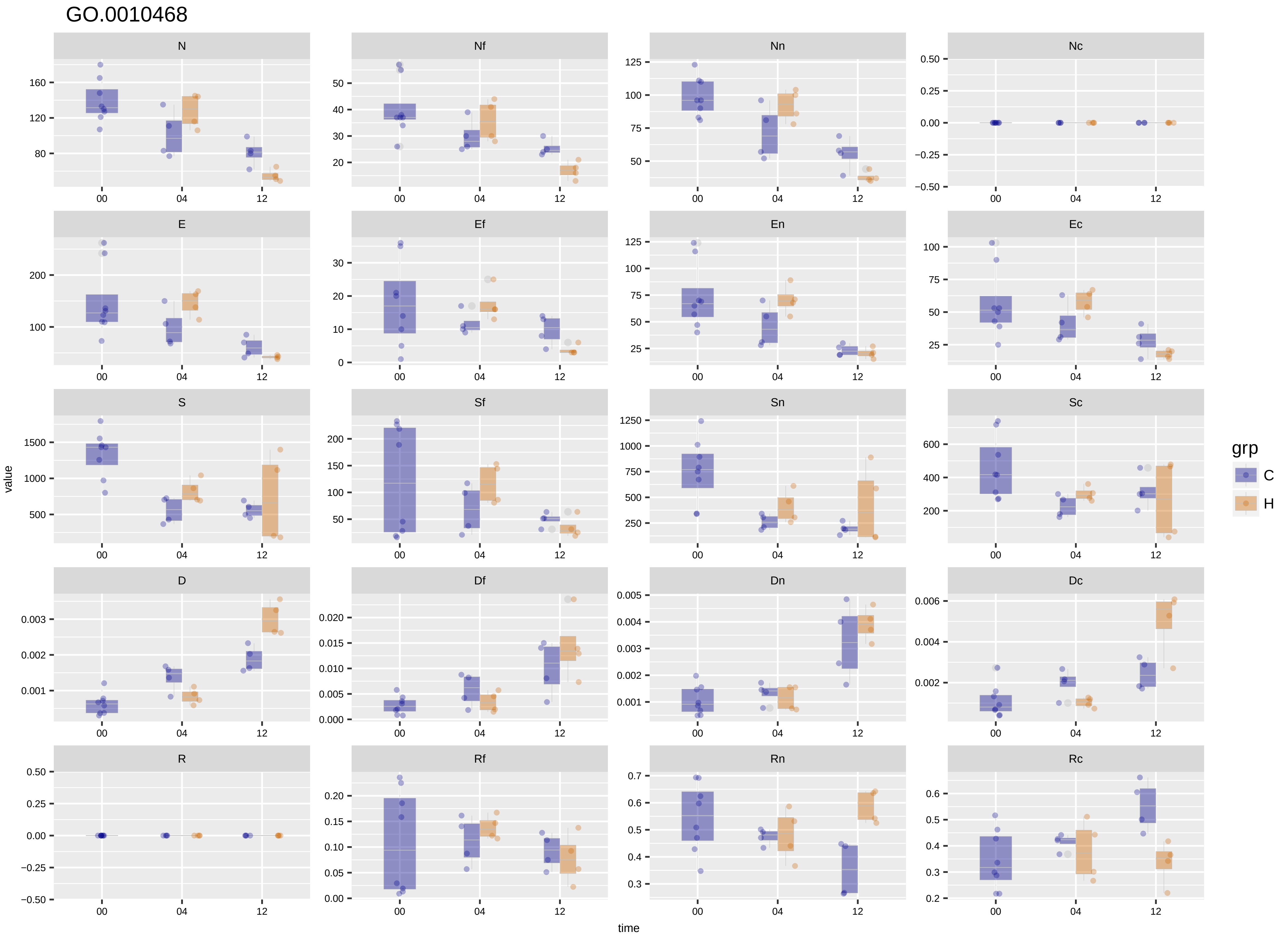
GO.0010468
N
Nf
Nn
Nc
E
Ef
En
Ec
S
Sf
Sn
Sc
D
Df
Dn
Dc
R
Rf
Rn
Rc
value
time
grp
C
H
00
04
12

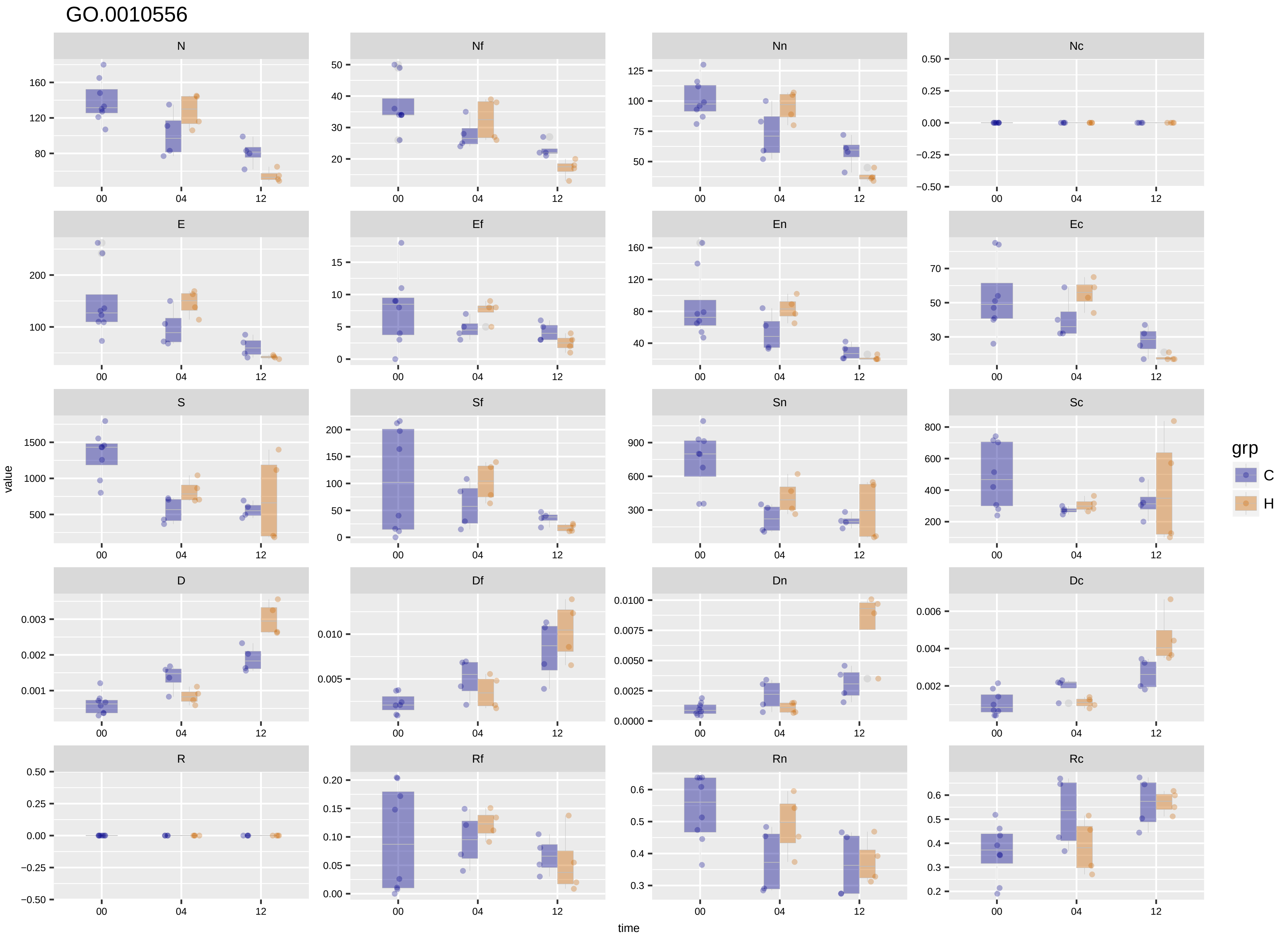
GO.0010556
N
Nf
Nn
Nc
E
Ef
En
Ec
S
Sf
Sn
Sc
D
Df
Dn
Dc
R
Rf
Rn
Rc
value
time
grp
C
H

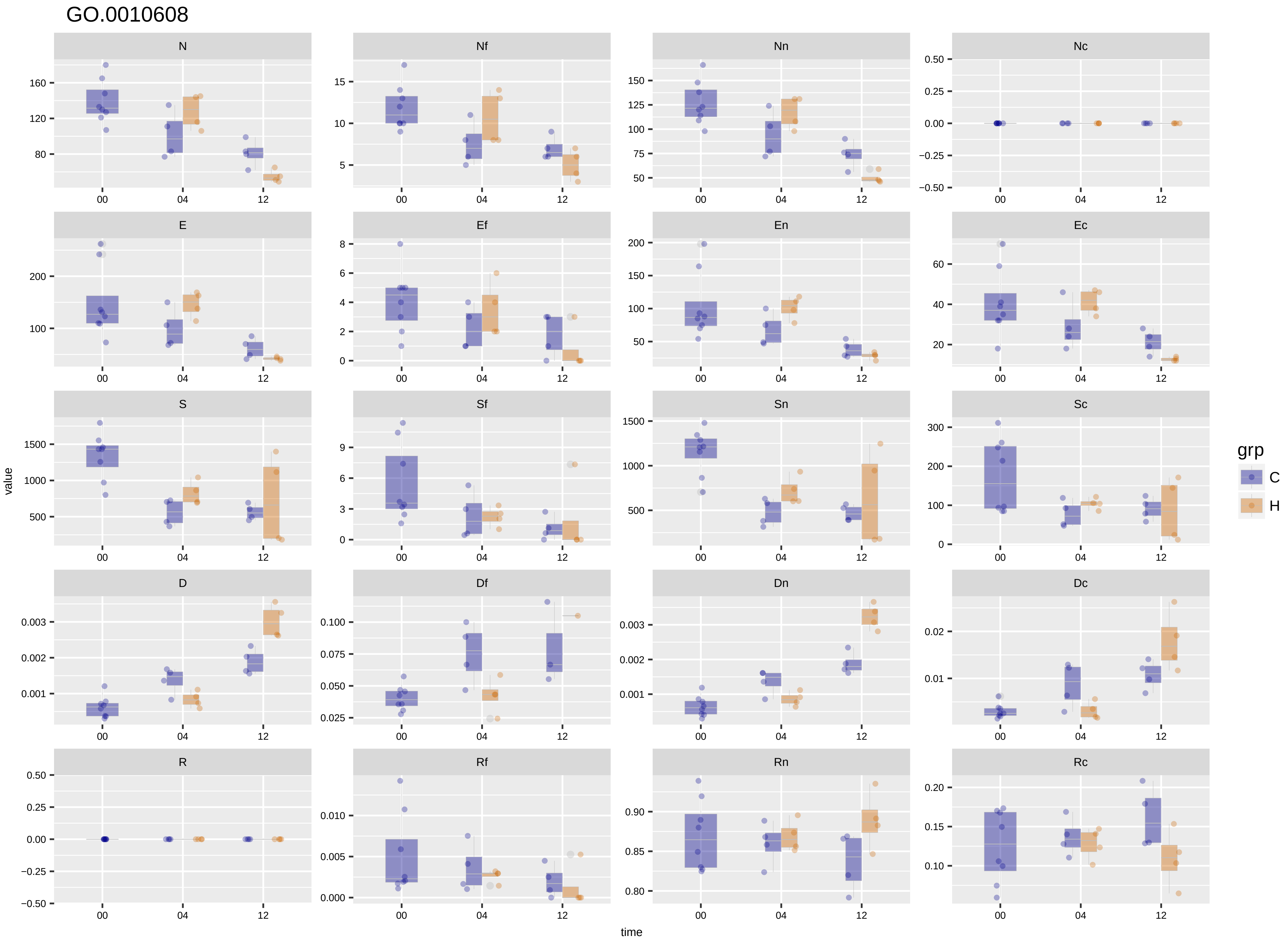
GO.0010608
N
Nf
Nn
Nc
E
Ef
En
Ec
S
Sf
Sn
Sc
D
Df
Dn
Dc
R
Rf
Rn
Rc
value
time
grp
C
H

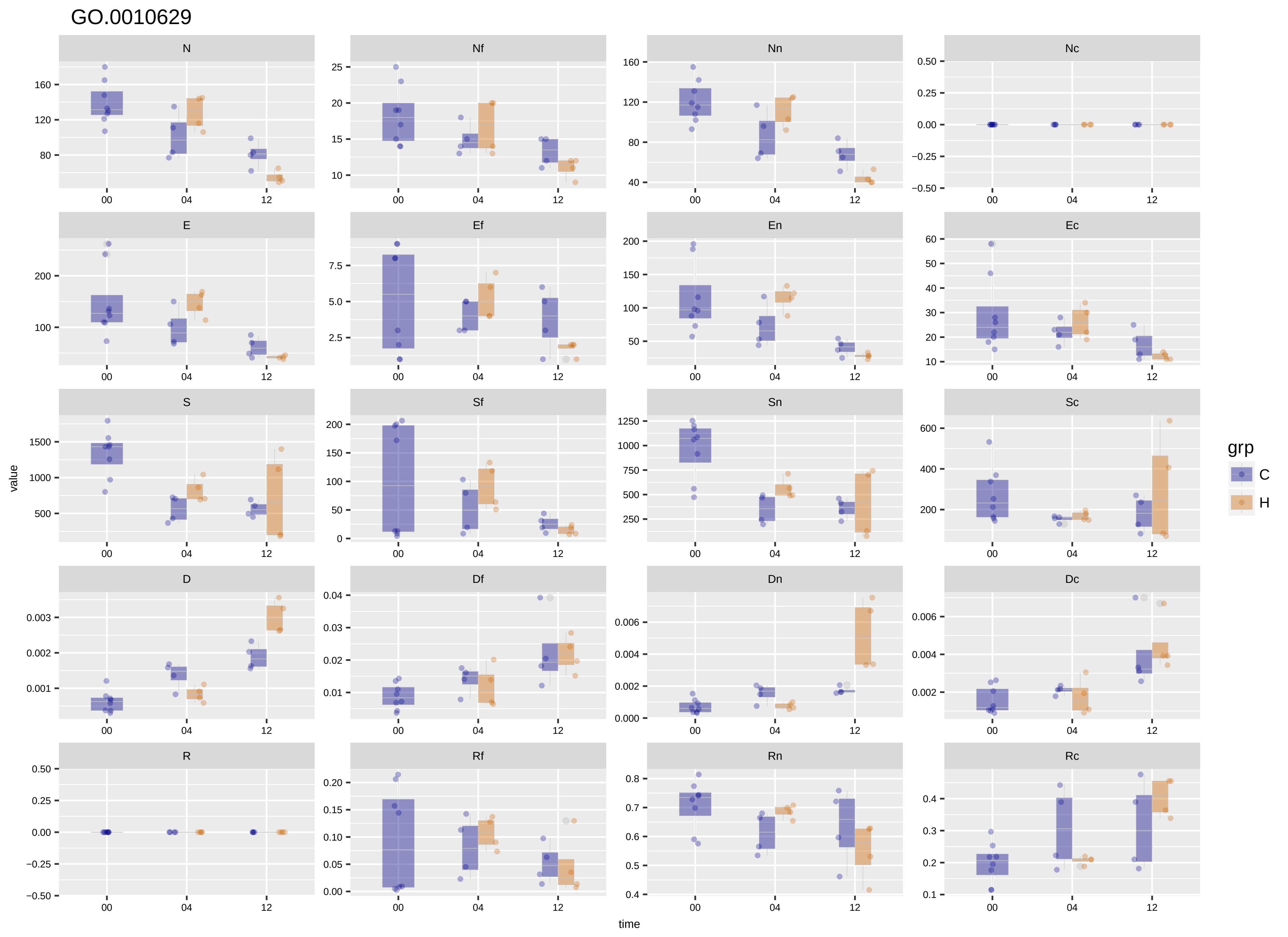

GO.0010629
N
Nf
Nn
Nc
E
Ef
En
Ec
S
Sf
Sn
Sc
D
Df
Dn
Dc
R
Rf
Rn
Rc
value
time
grp
C
H

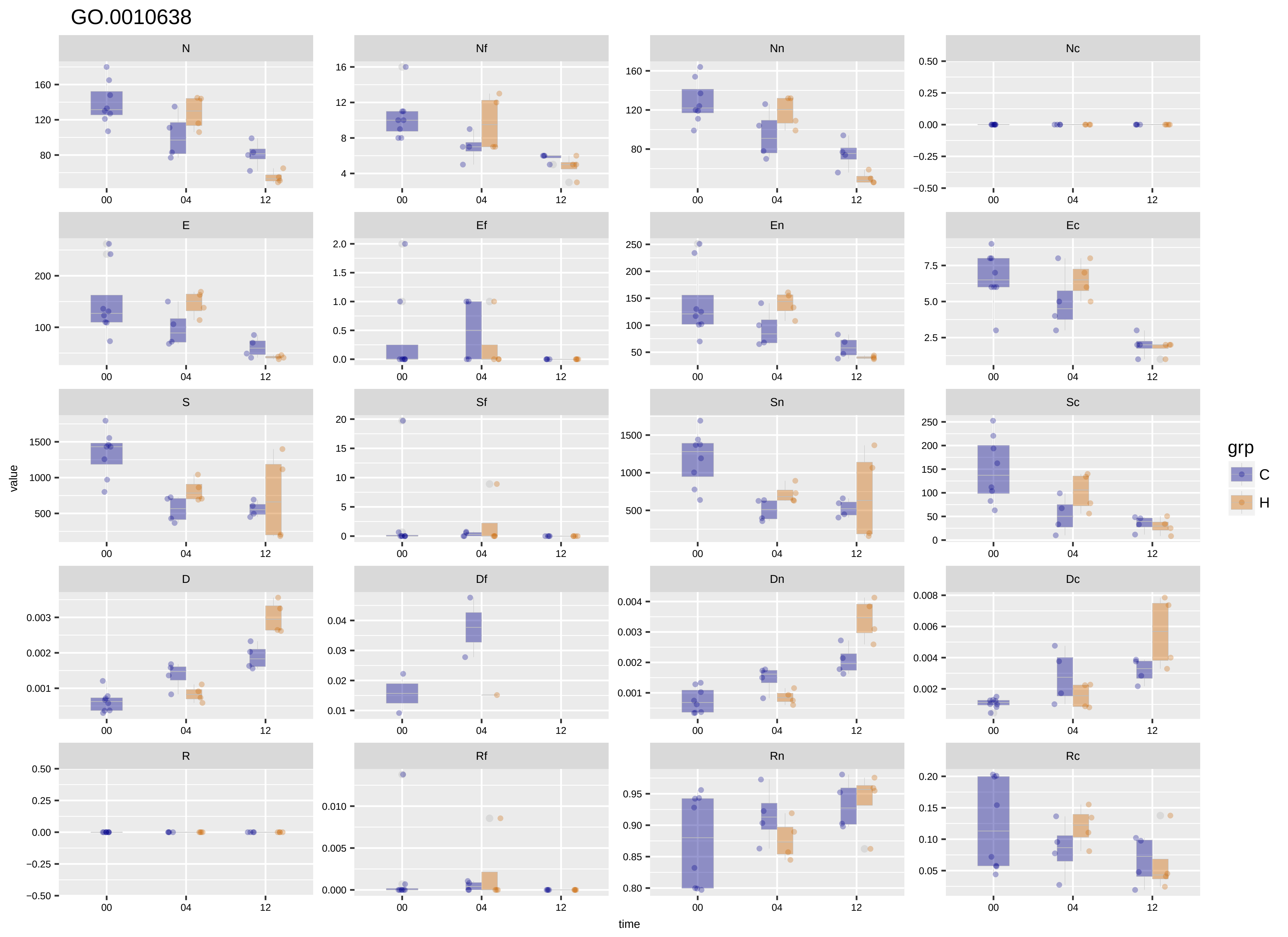

GO.0010638
N
Nf
Nn
Nc
E
Ef
En
Ec
S
Sf
Sn
Sc
D
Df
Dn
Dc
R
Rf
Rn
Rc
value
time
00
04
12
grp
C
H

GO.0010821

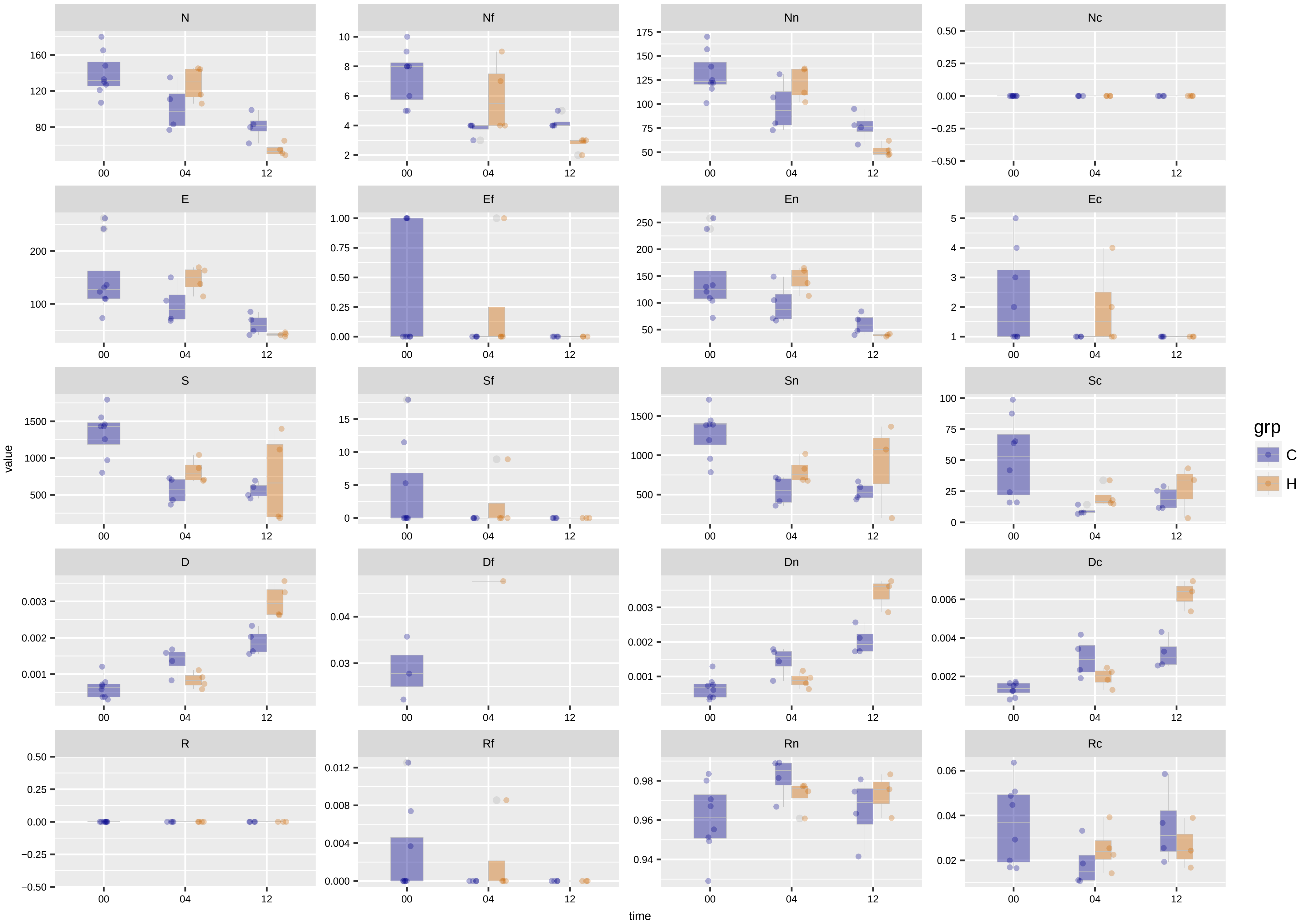

N
Nf
Nn
Nc
E
Ef
En
Ec
S
Sf
Sn
Sc
D
Df
Dn
Dc
R
Rf
Rn
Rc
00
04
12
value
time
grp
C
H

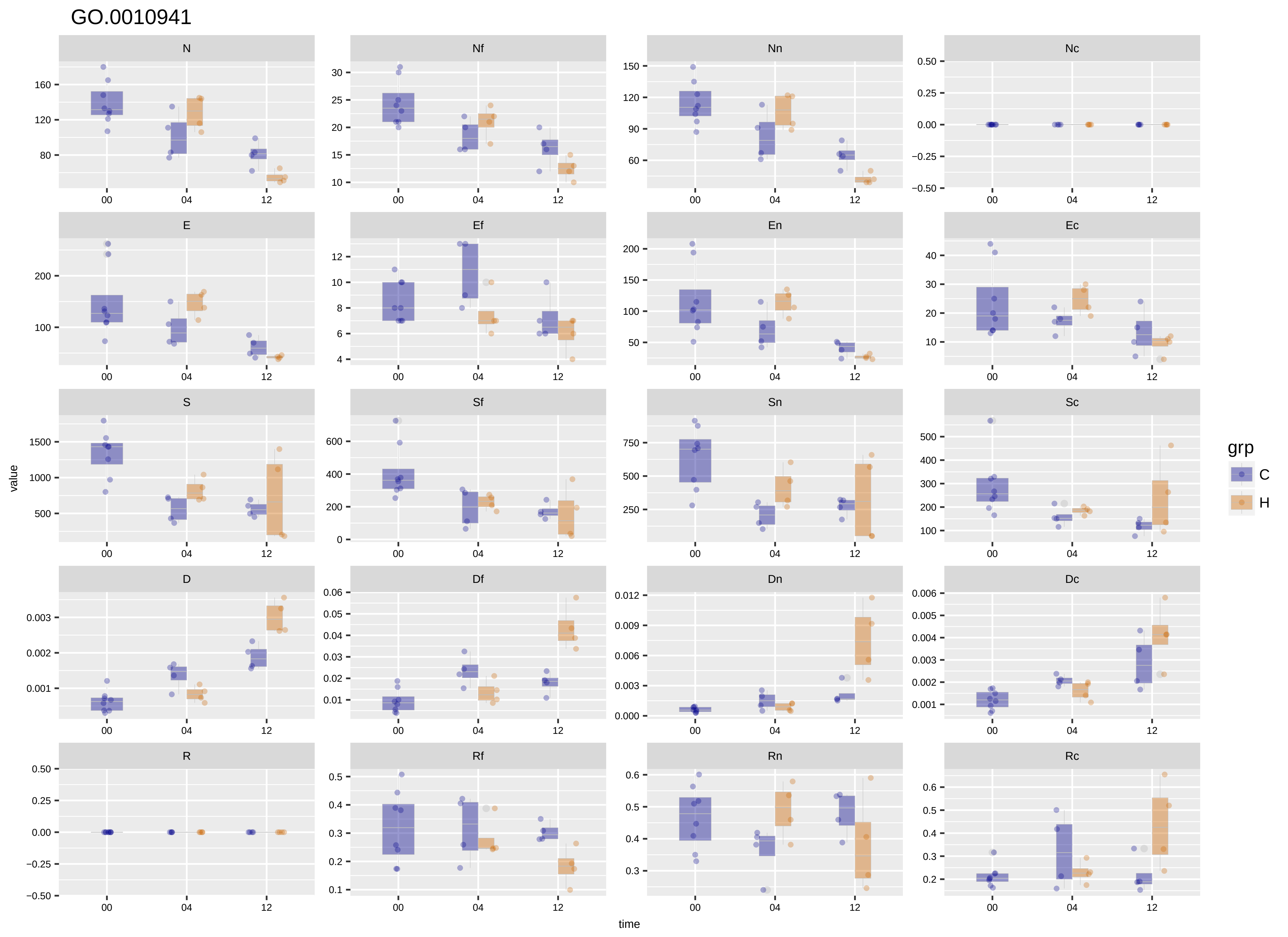
GO.0010941
N
Nf
Nn
Nc
E
Ef
En
Ec
S
Sf
Sn
Sc
D
Df
Dn
Dc
R
Rf
Rn
Rc
value
time
00
04
12
grp
C
H

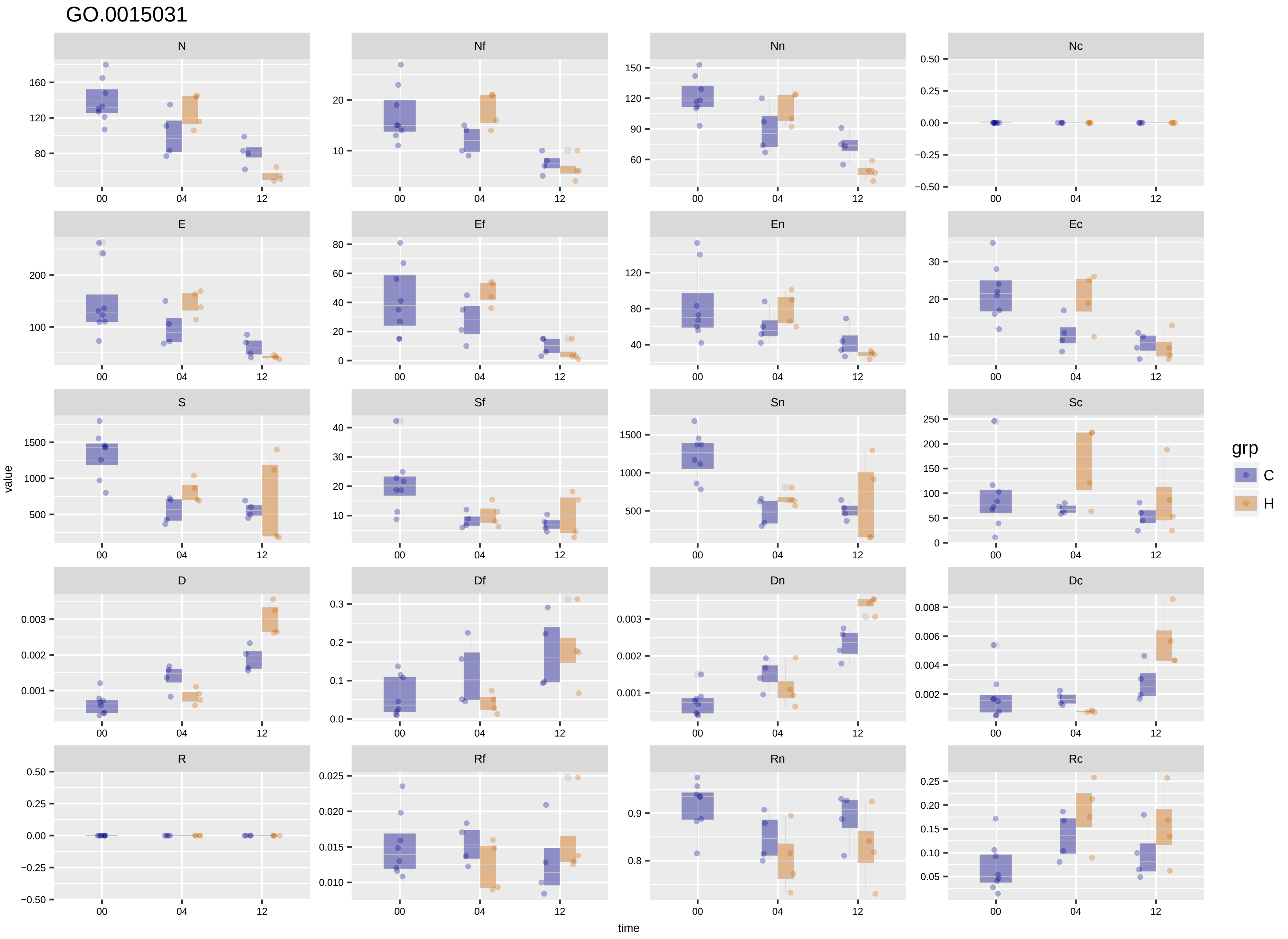

GO.0015031
N
Nf
Nn
Nc
E
Ef
En
Ec
S
Sf
Sn
Sc
D
Df
Dn
Dc
R
Rf
Rn
Rc
value
time
grp
C
H

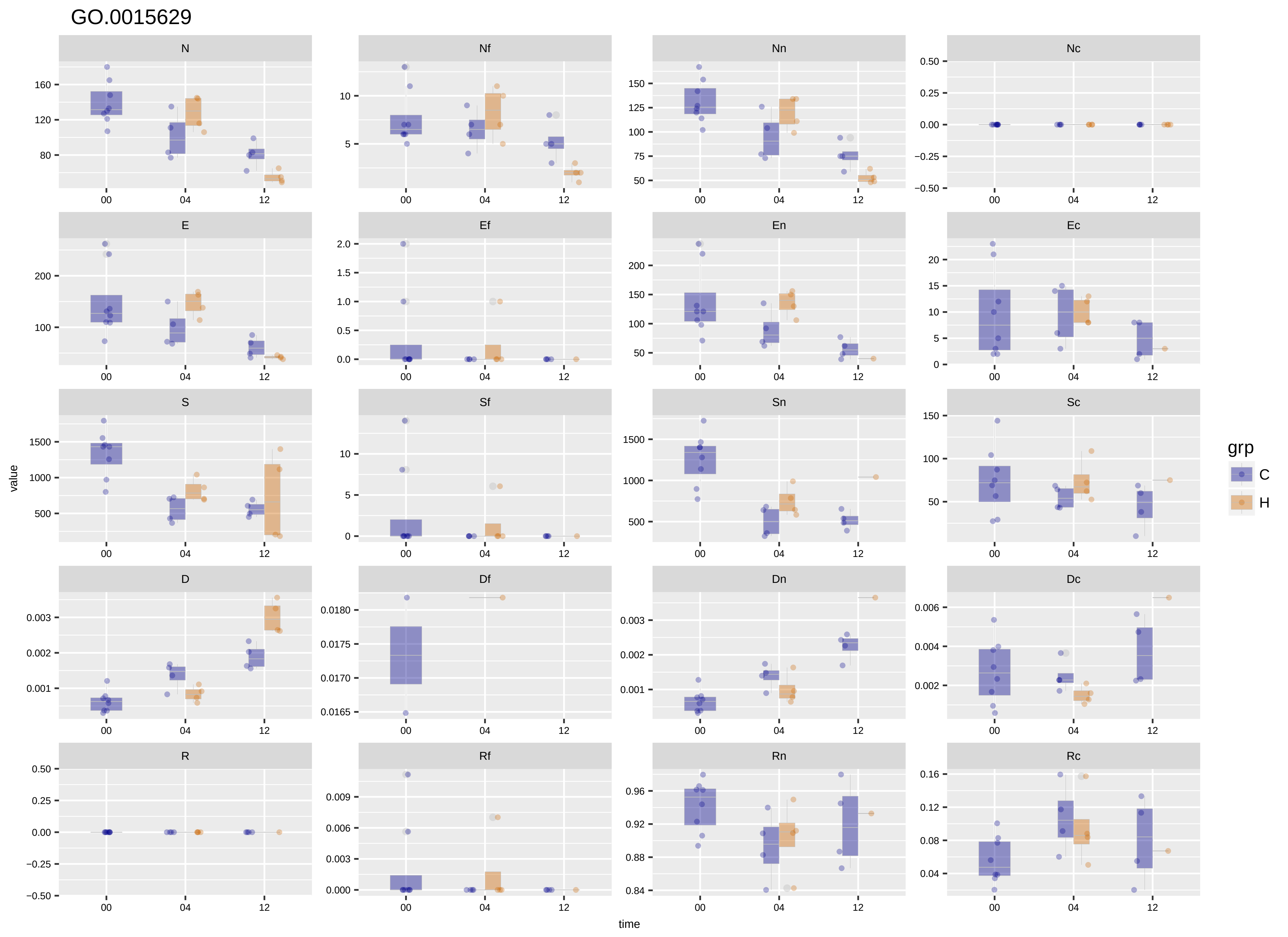

GO.0015629
N
Nf
Nn
Nc
E
Ef
En
Ec
S
Sf
Sn
Sc
D
Df
Dn
Dc
R
Rf
Rn
Rc
00
04
12
value
time
grp
C
H

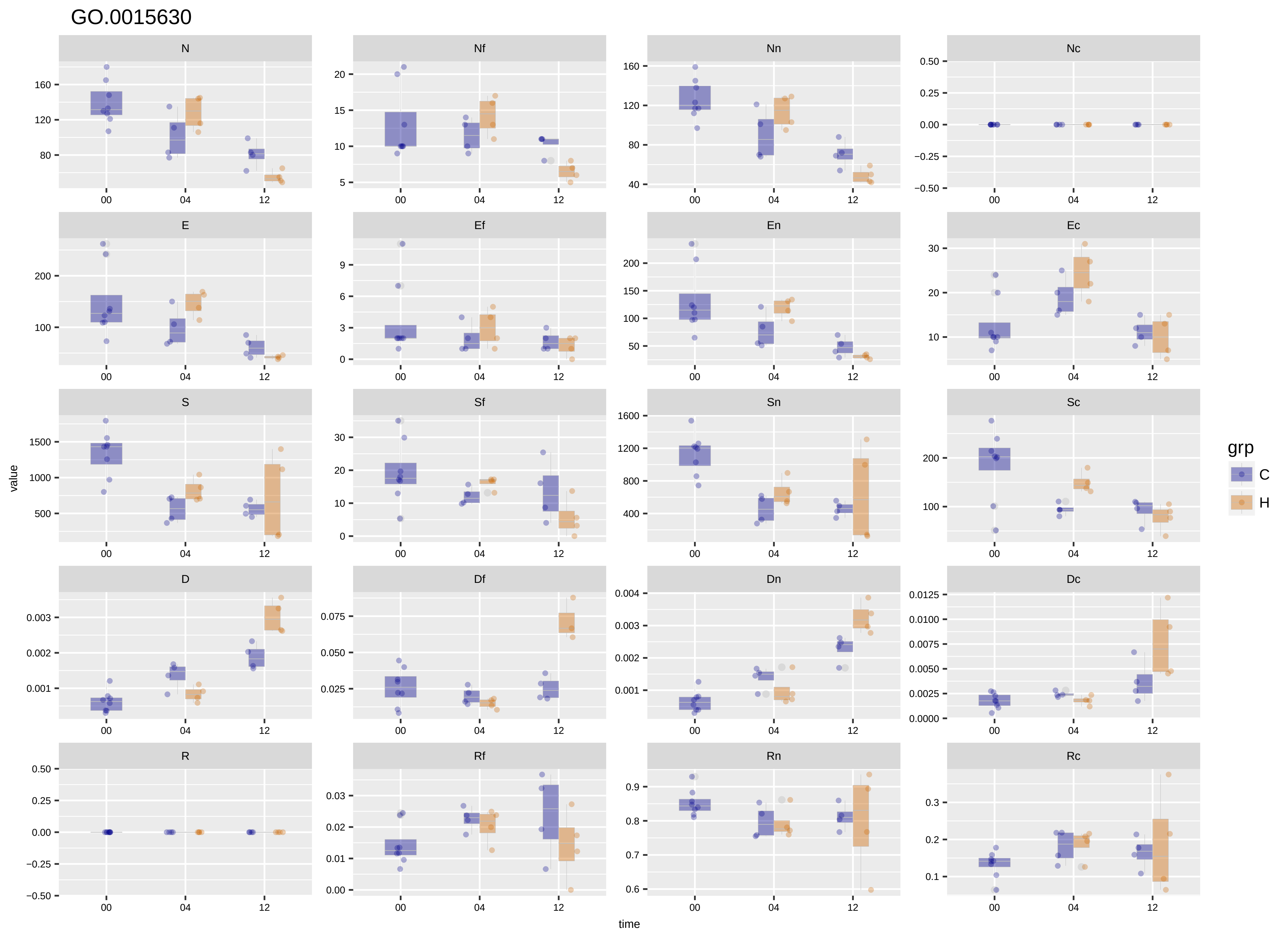

GO.0015630
N
Nf
Nn
Nc
E
Ef
En
Ec
S
Sf
Sn
Sc
D
Df
Dn
Dc
R
Rf
Rn
Rc
value
time
grp
C
H

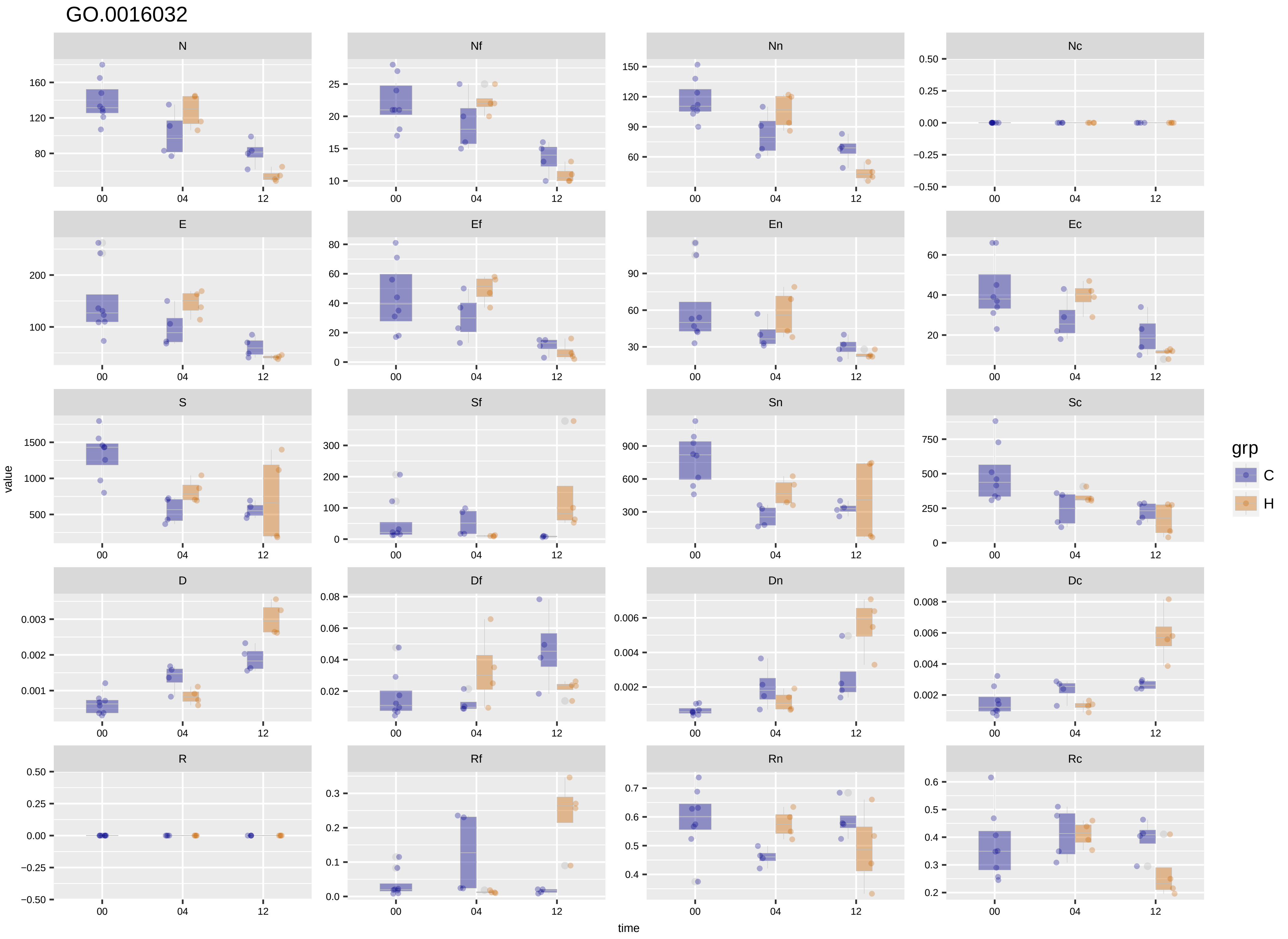
GO.0016032
N
Nf
Nn
Nc
E
Ef
En
Ec
S
Sf
Sn
Sc
D
Df
Dn
Dc
R
Rf
Rn
Rc
value
time
00
04
12
grp
C
H

GO.0016043

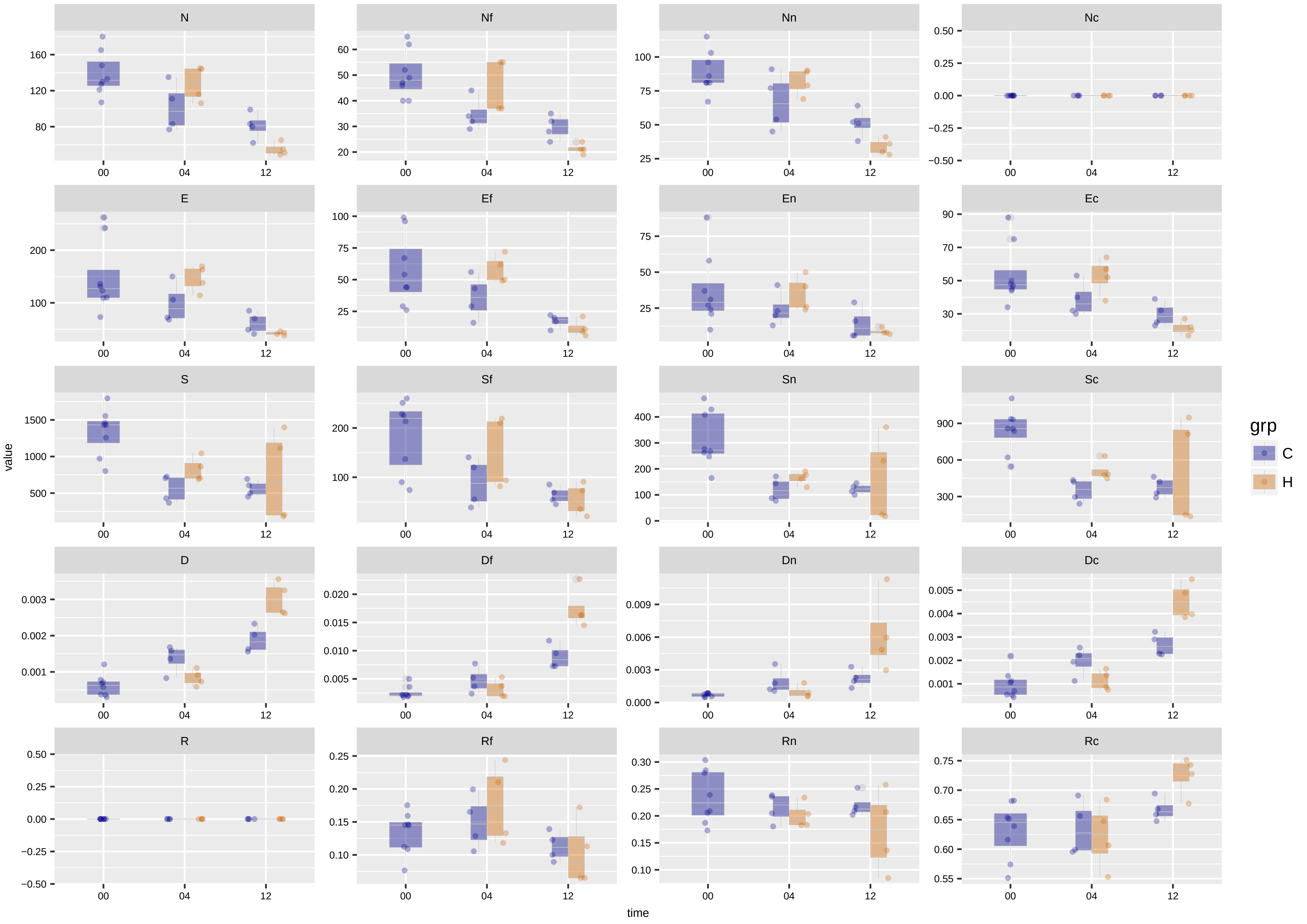

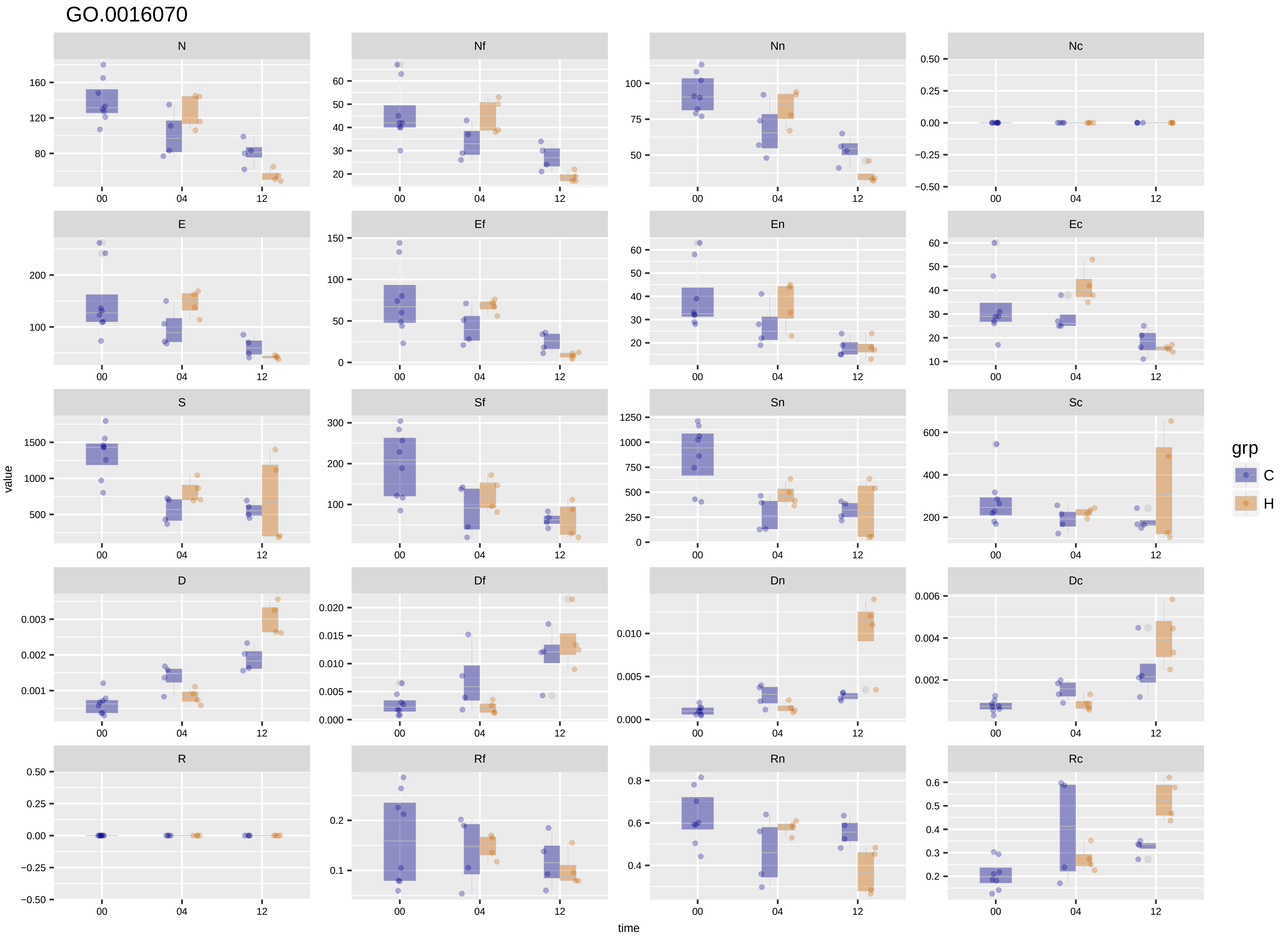
GO.0016070
N
Nf
Nn
Nc
E
Ef
En
Ec
S
Sf
Sn
Sc
D
Df
Dn
Dc
R
Rf
Rn
Rc
value
time
grp
C
H
00
04
12

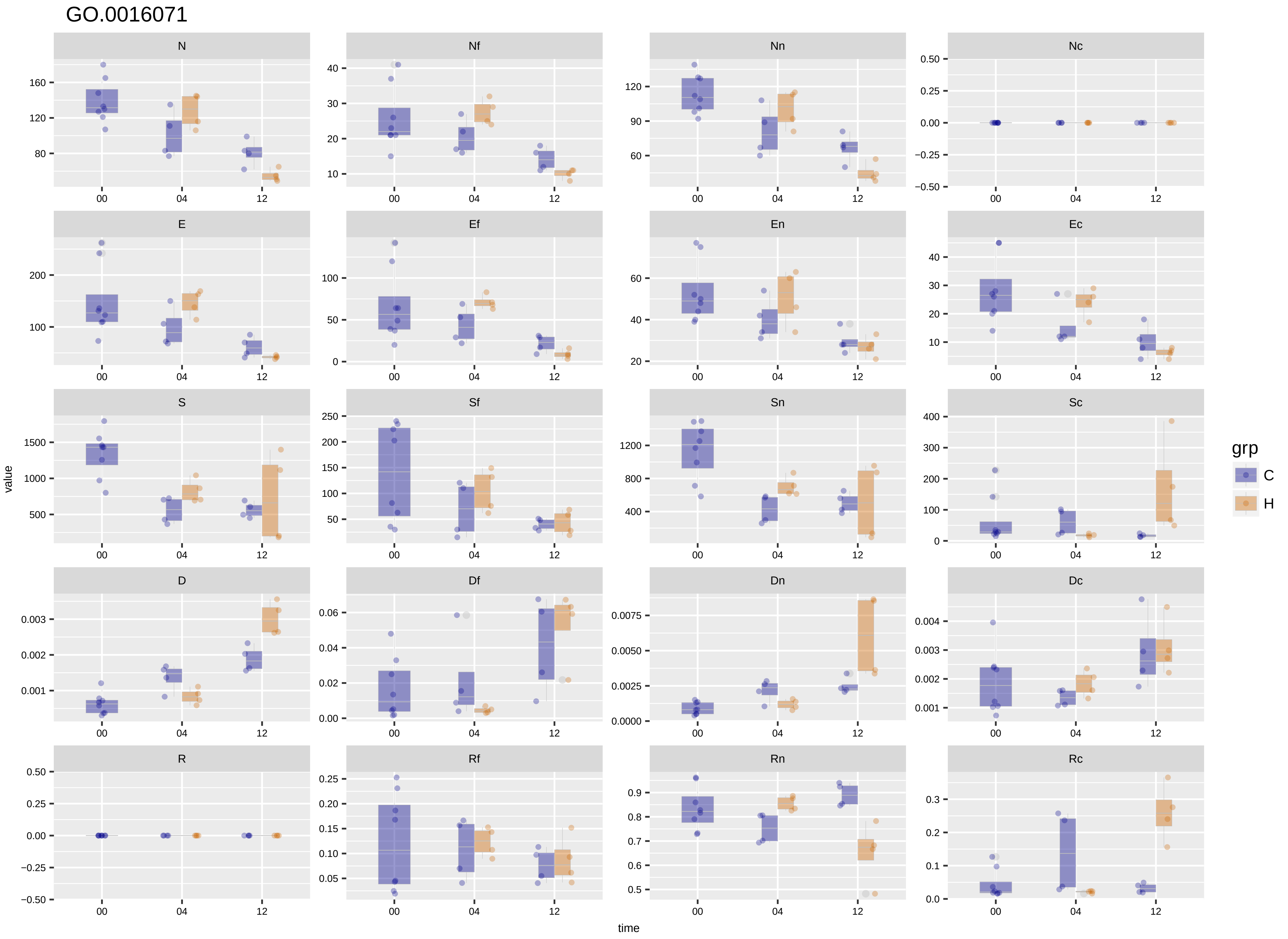
GO.0016071
N
Nf
Nn
Nc
E
Ef
En
Ec
S
Sf
Sn
Sc
D
Df
Dn
Dc
R
Rf
Rn
Rc
value
time
grp
C
H

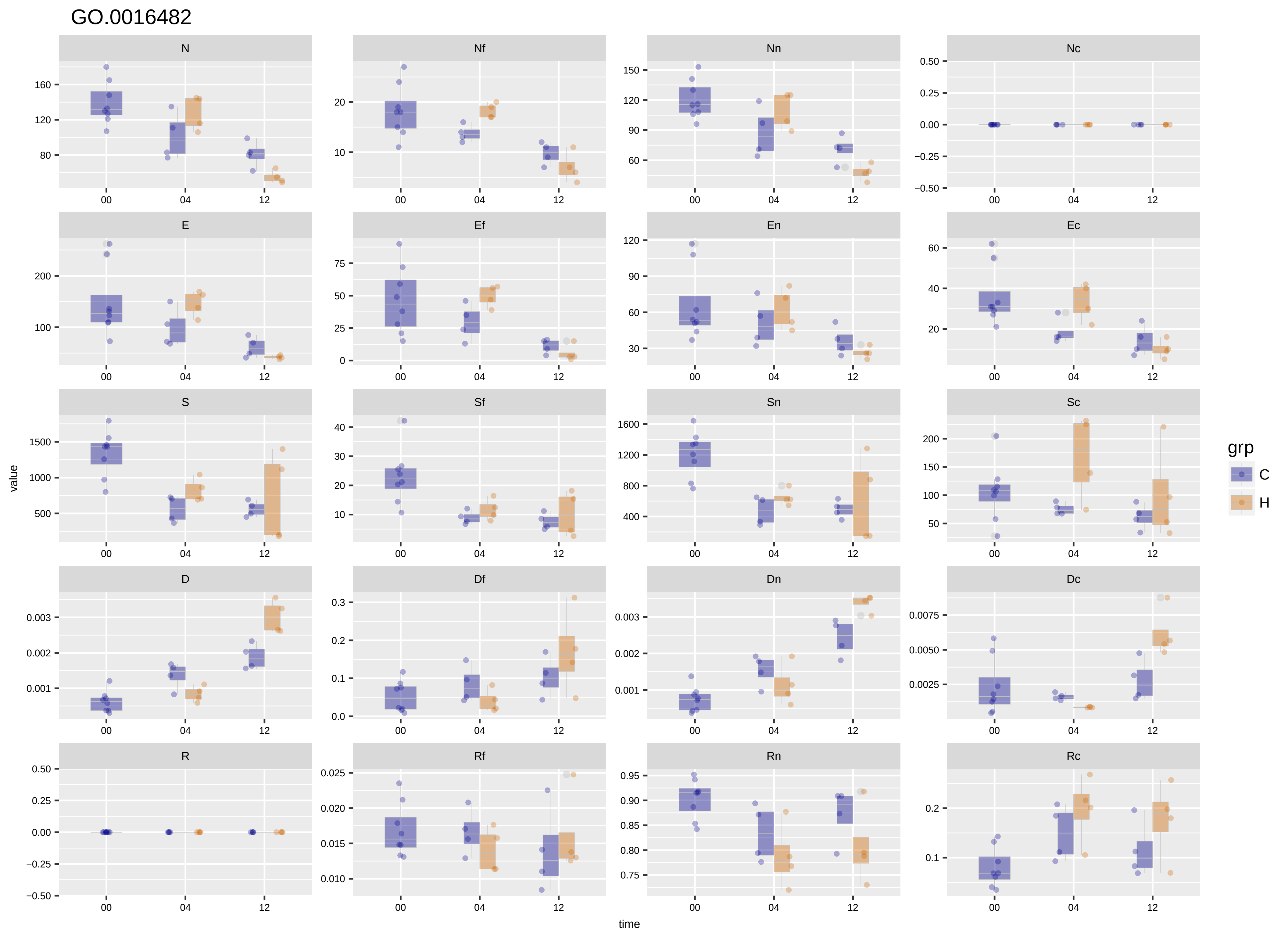
GO.0016482
N
Nf
Nn
Nc
E
Ef
En
Ec
S
Sf
Sn
Sc
D
Df
Dn
Dc
R
Rf
Rn
Rc
00
04
12
value
time
grp
C
H

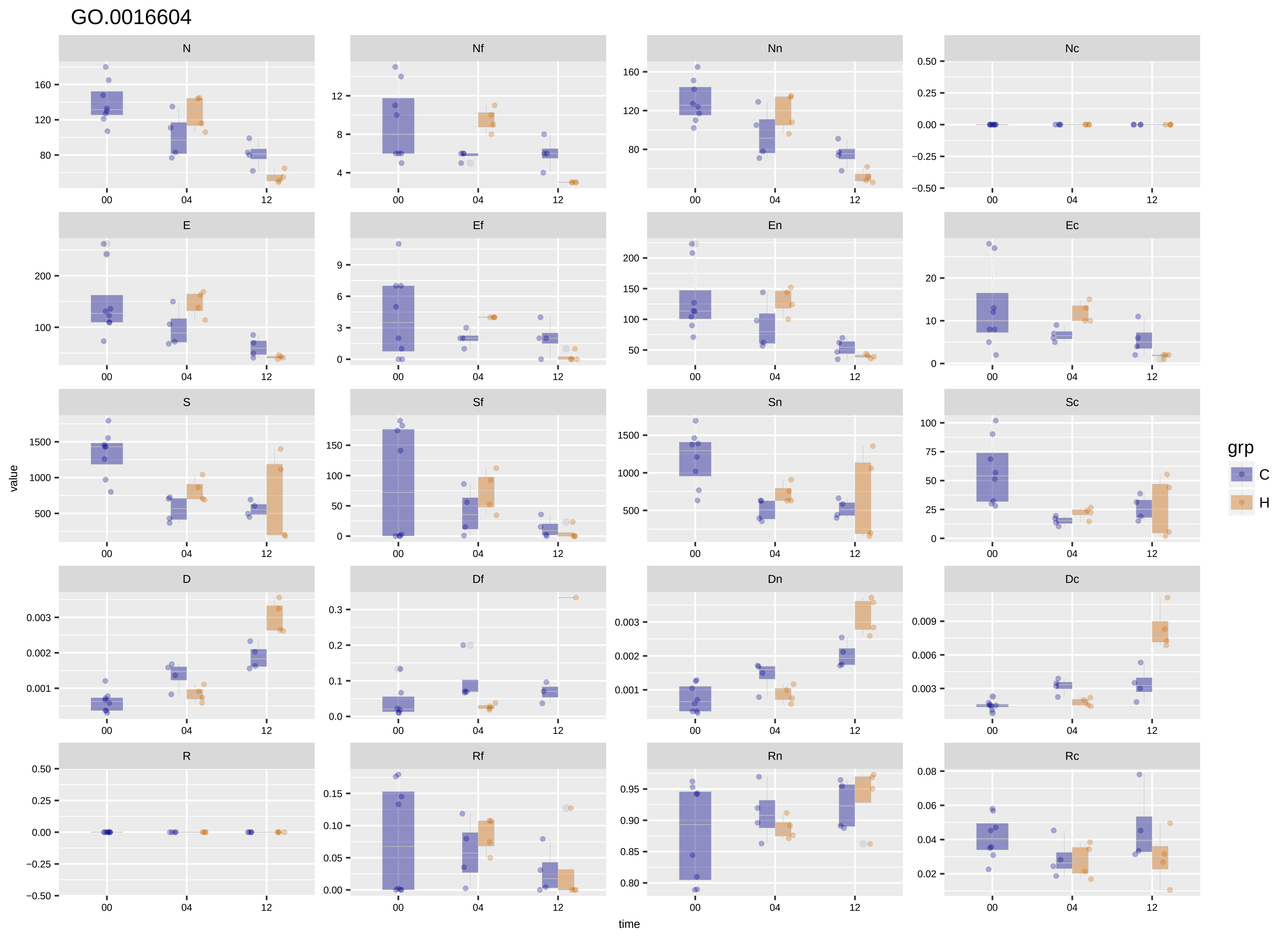

GO.0016604
N
Nf
Nn
Nc
E
Ef
En
Ec
S
Sf
Sn
Sc
D
Df
Dn
Dc
R
Rf
Rn
Rc
value
time
grp
C
H

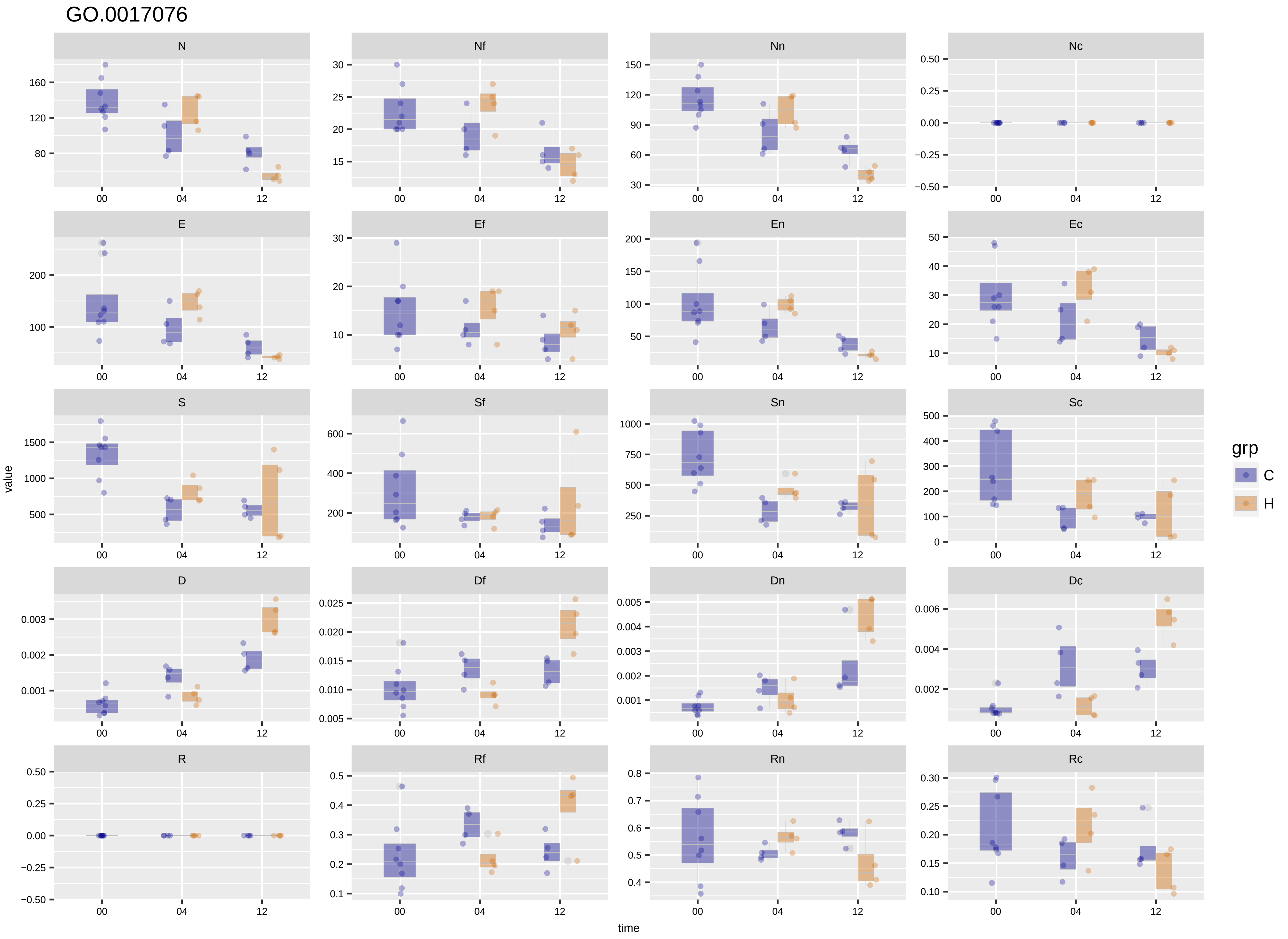

GO.0017076
N
Nf
Nn
Nc
E
Ef
En
Ec
S
Sf
Sn
Sc
D
Df
Dn
Dc
R
Rf
Rn
Rc
00
04
12
value
time
grp
C
H

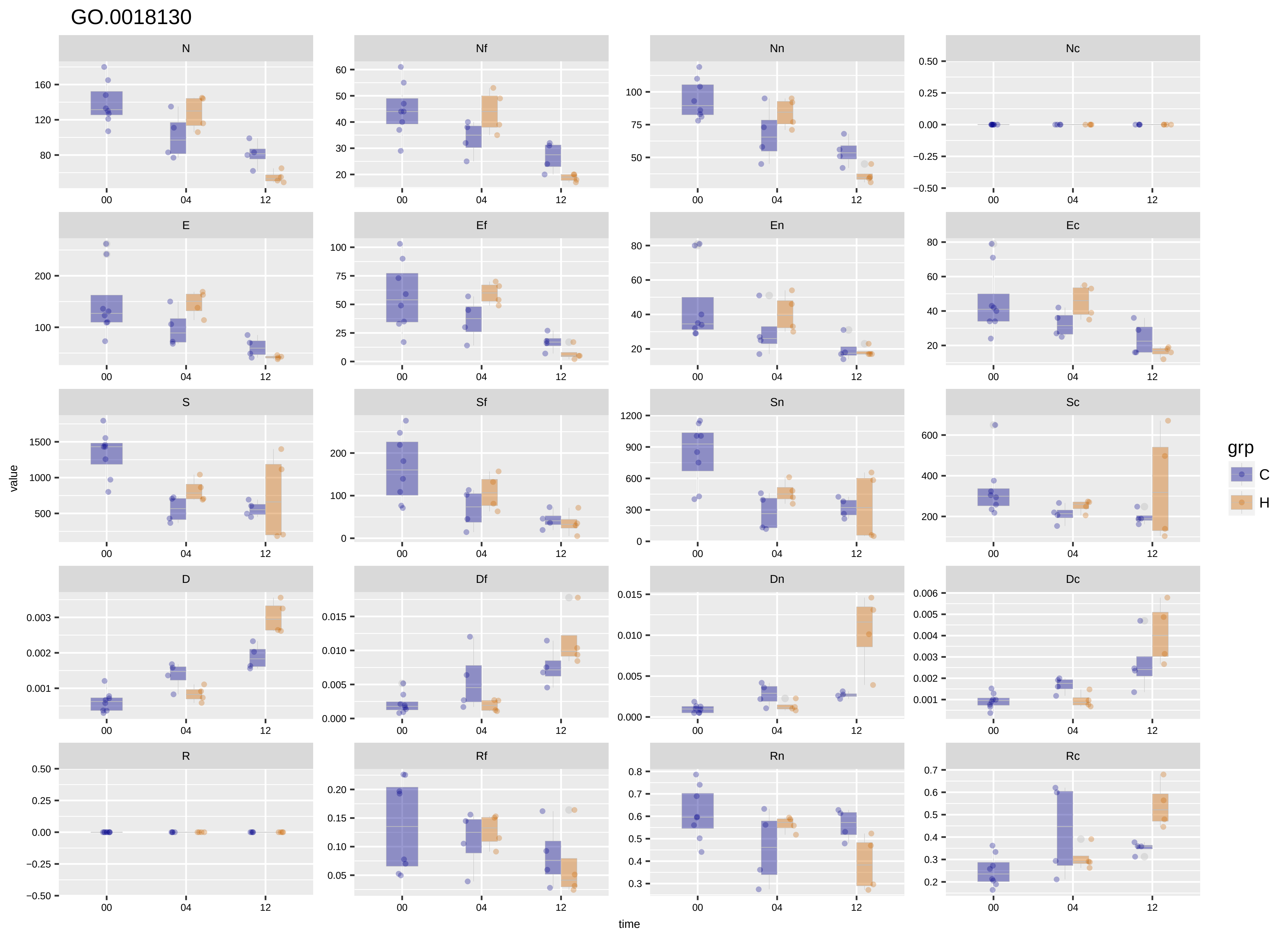
GO.0018130
N
Nf
Nn
Nc
E
Ef
En
Ec
S
Sf
Sn
Sc
D
Df
Dn
Dc
R
Rf
Rn
Rc
value
time
grp
C
H

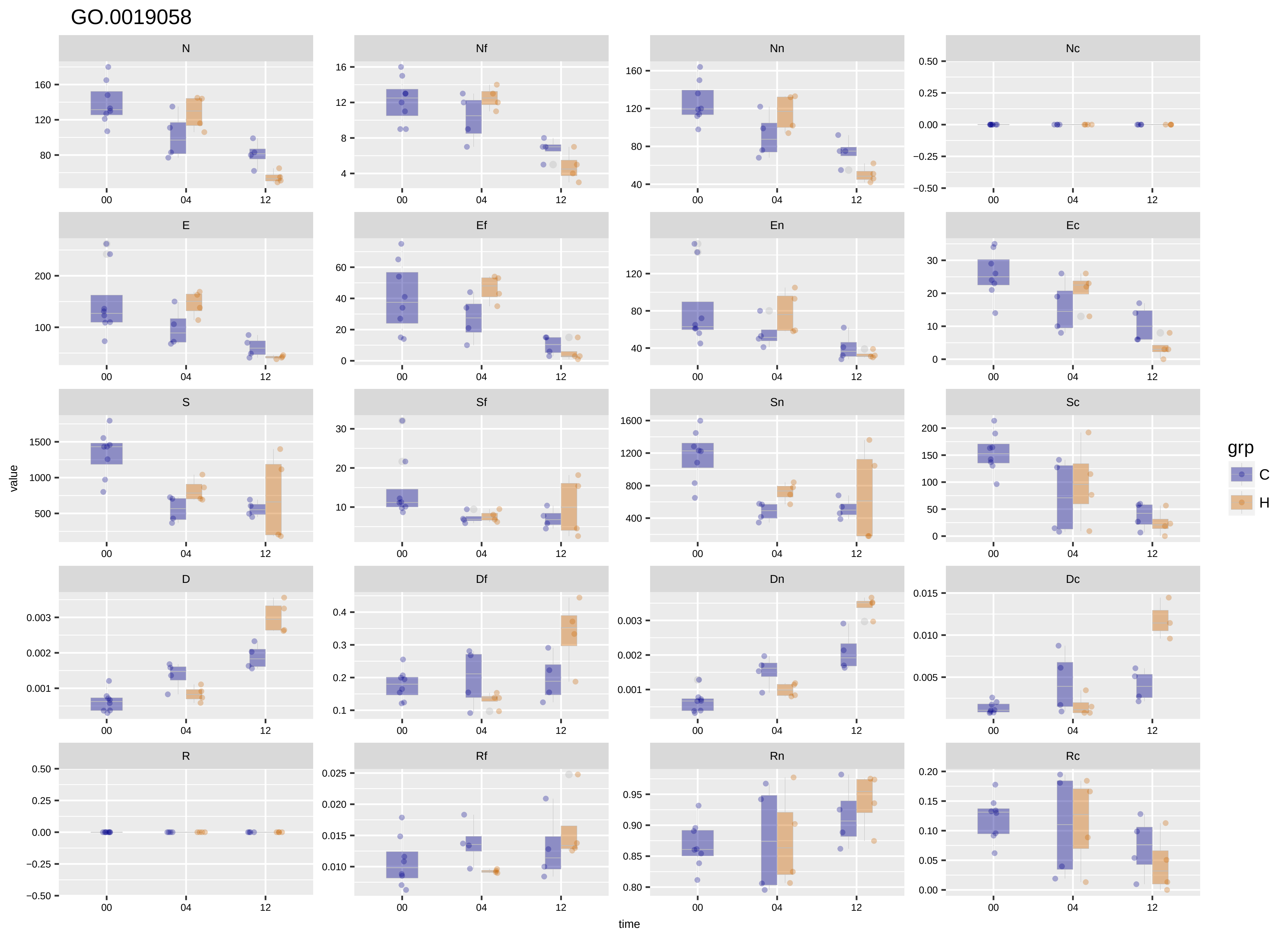
GO.0019058
N
Nf
Nn
Nc
E
Ef
En
Ec
S
Sf
Sn
Sc
D
Df
Dn
Dc
R
Rf
Rn
Rc
value
time
grp
C
H

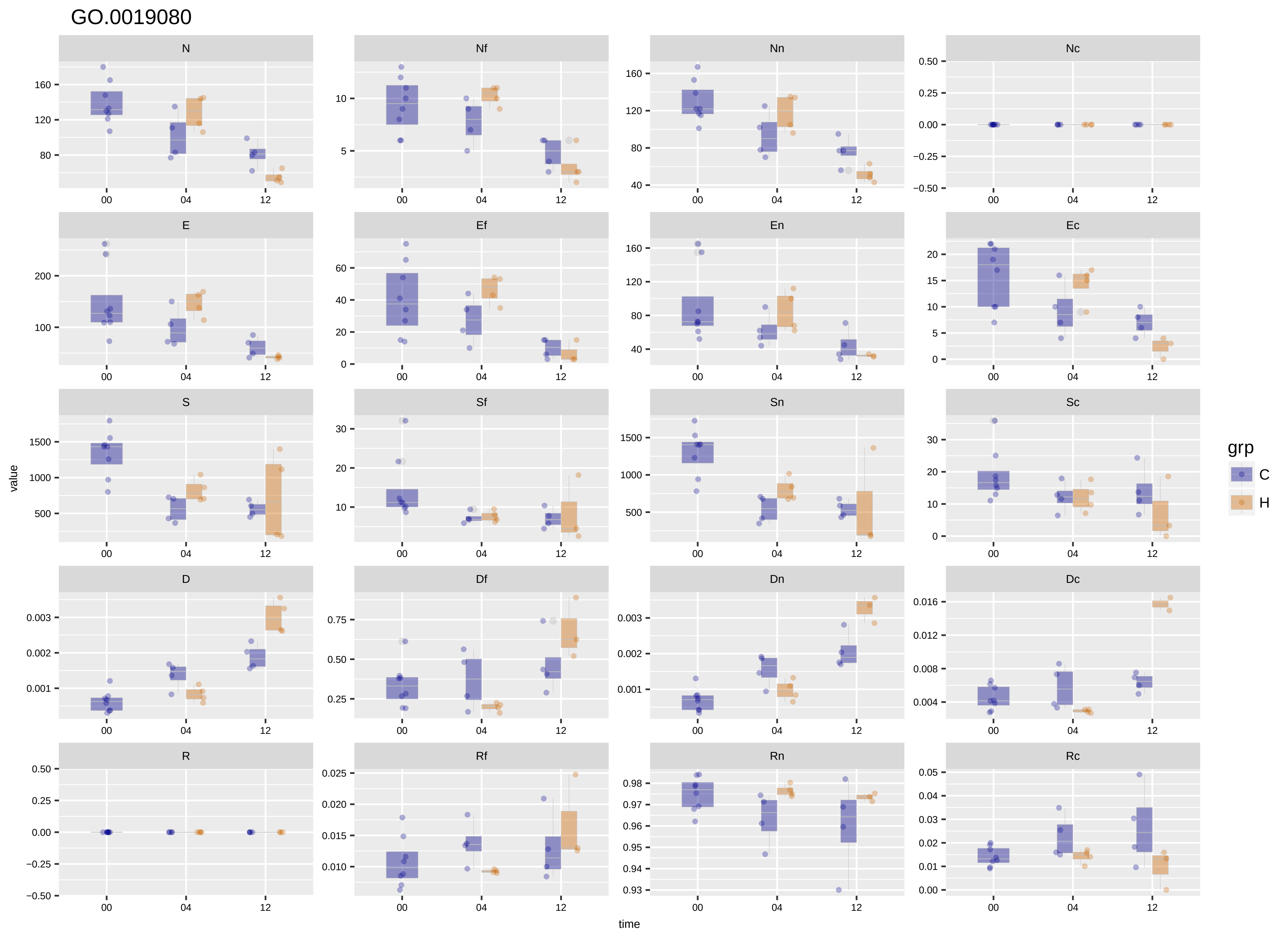

GO.0019080
N
Nf
Nn
Nc
E
Ef
En
Ec
S
Sf
Sn
Sc
D
Df
Dn
Dc
R
Rf
Rn
Rc
value
time
grp
C
H

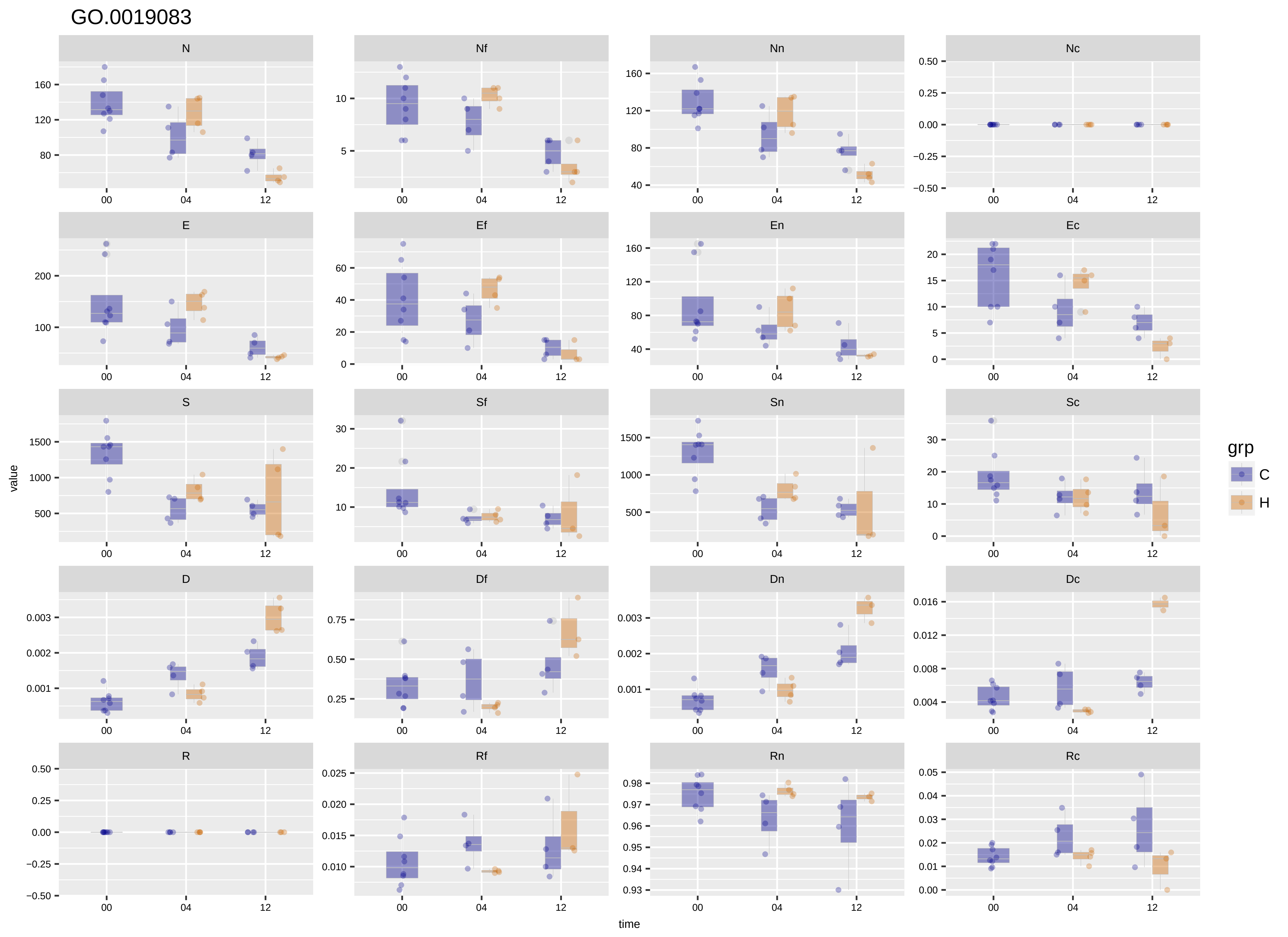

GO.0019083
N
Nf
Nn
Nc
E
Ef
En
Ec
S
Sf
Sn
Sc
D
Df
Dn
Dc
R
Rf
Rn
Rc
00
04
12
value
time
grp
C
H

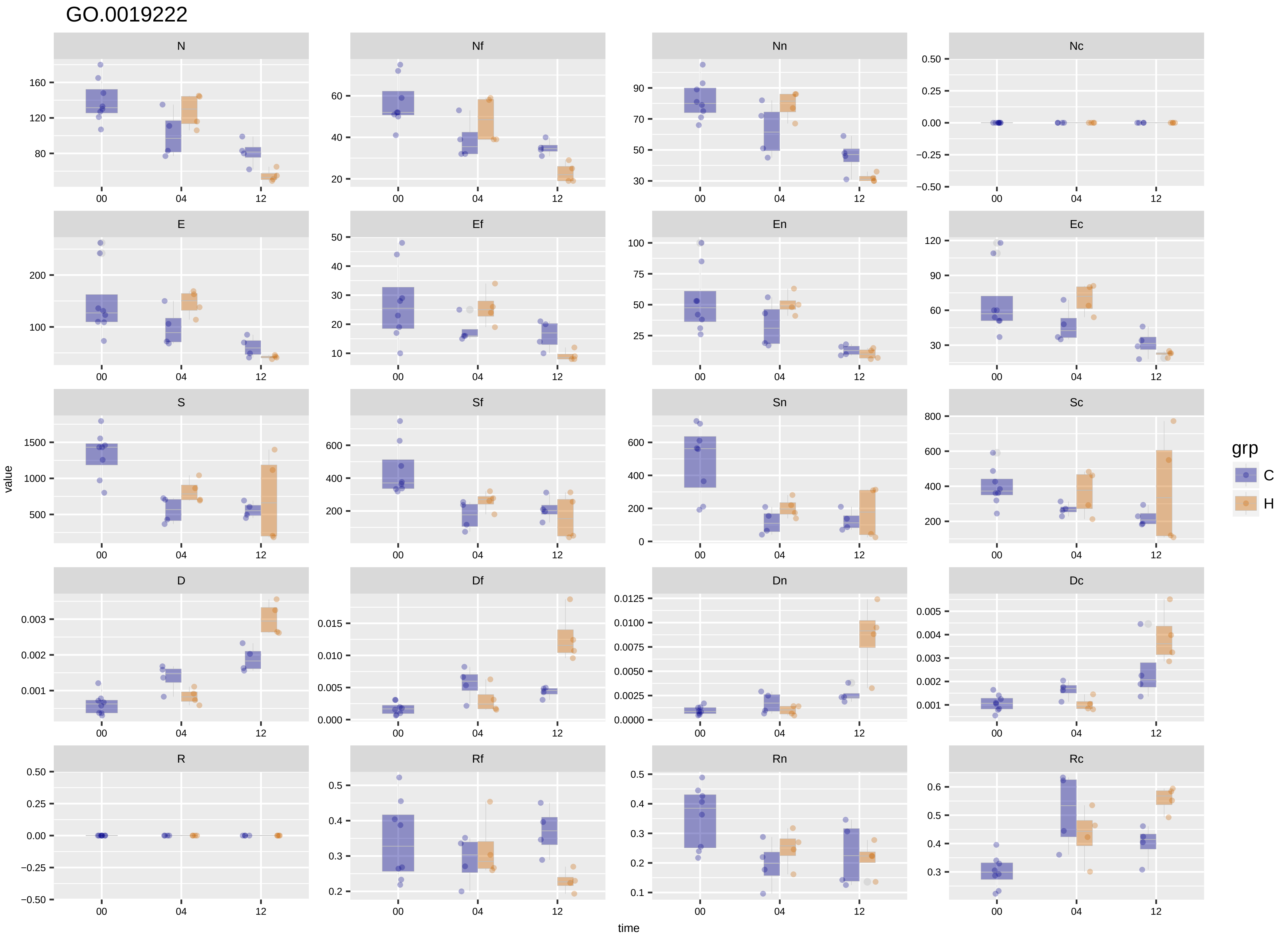
GO.0019222
N
Nf
Nn
Nc
E
Ef
En
Ec
S
Sf
Sn
Sc
D
Df
Dn
Dc
R
Rf
Rn
Rc
value
time
grp
C
H

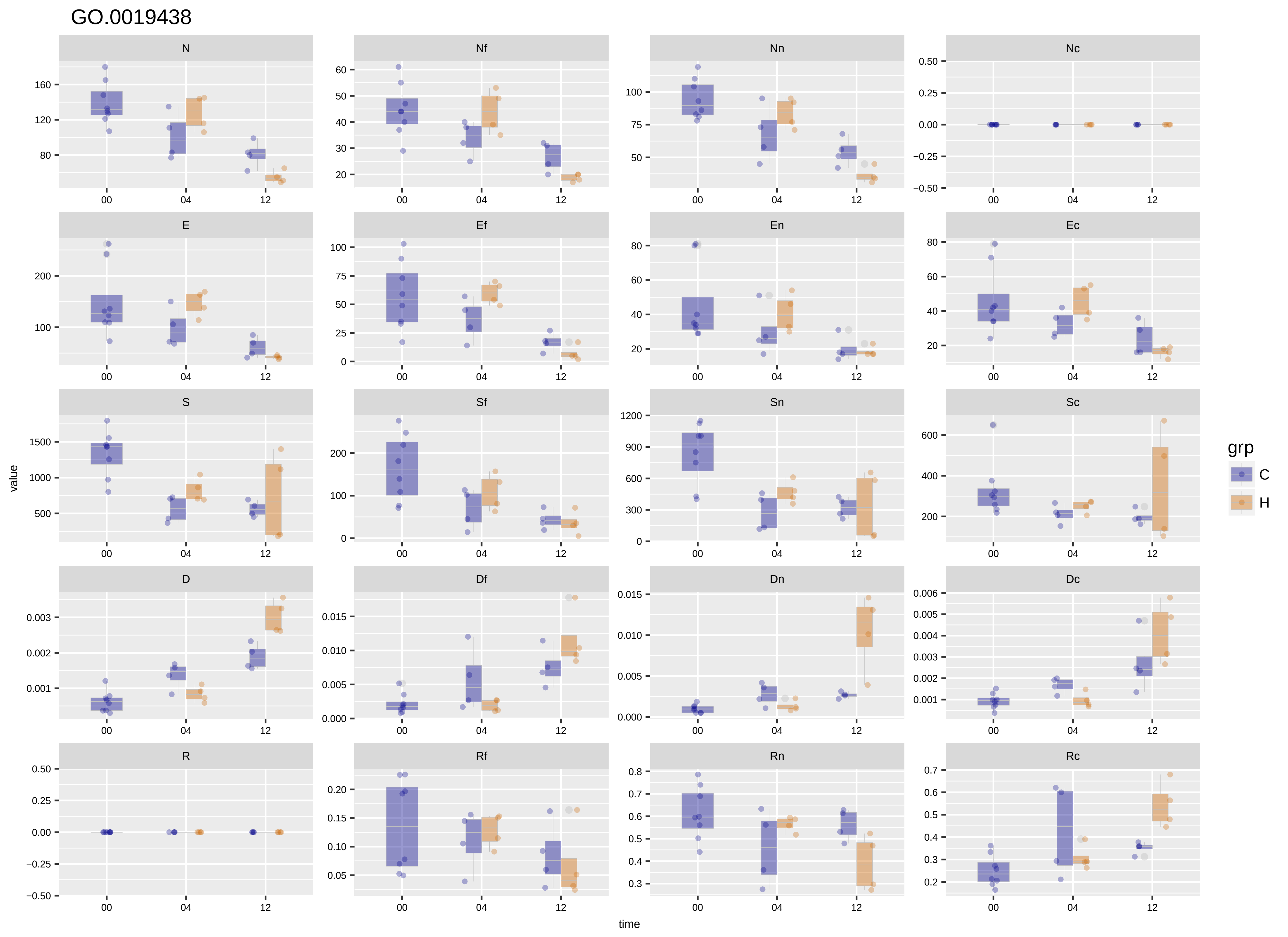

GO.0019438
N
Nf
Nn
Nc
E
Ef
En
Ec
S
Sf
Sn
Sc
D
Df
Dn
Dc
R
Rf
Rn
Rc
00
04
12
value
time
grp
C
H

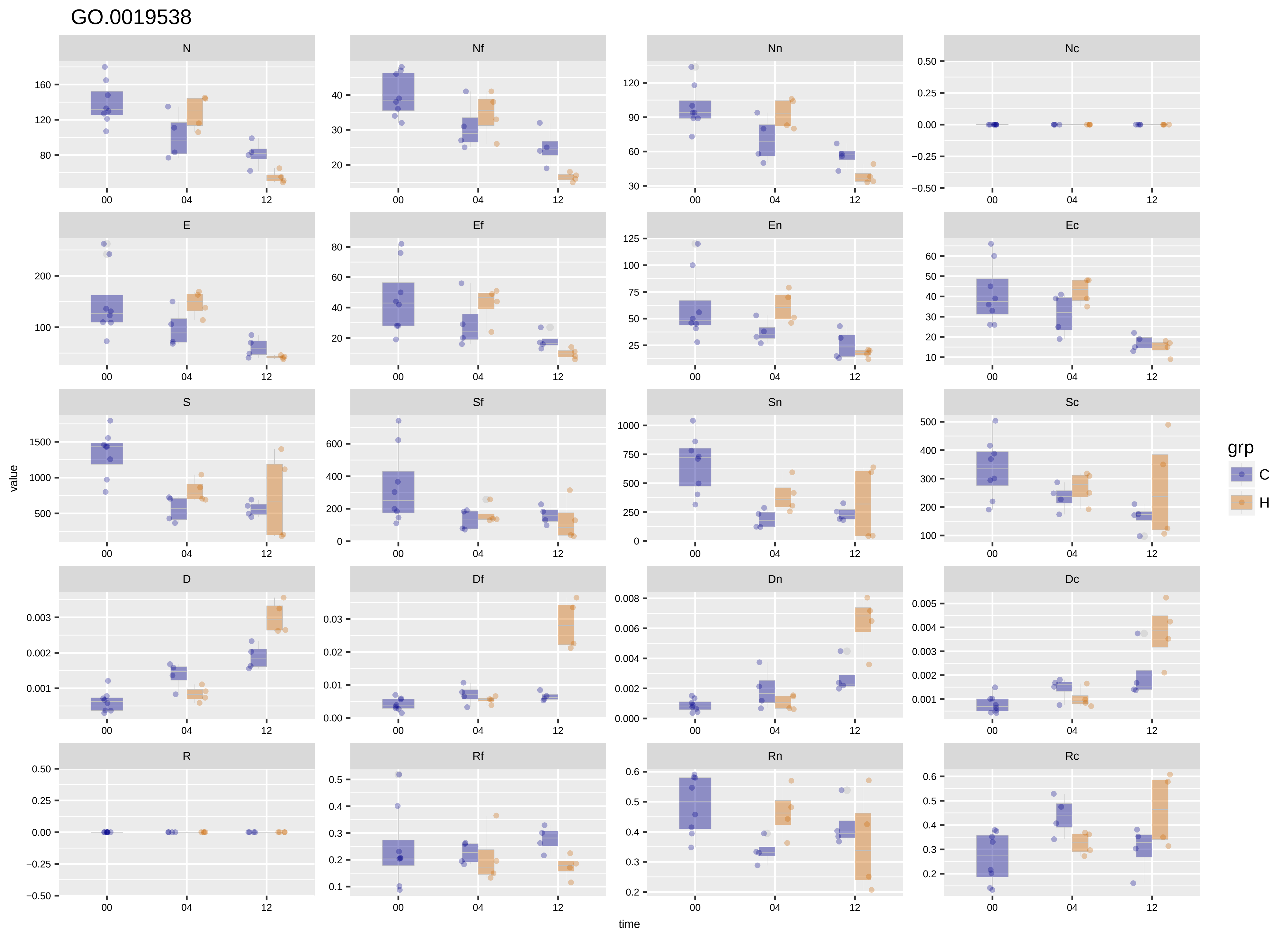
GO.0019538
N
Nf
Nn
Nc
E
Ef
En
Ec
S
Sf
Sn
Sc
D
Df
Dn
Dc
R
Rf
Rn
Rc
value
time
grp
C
H
00
04
12

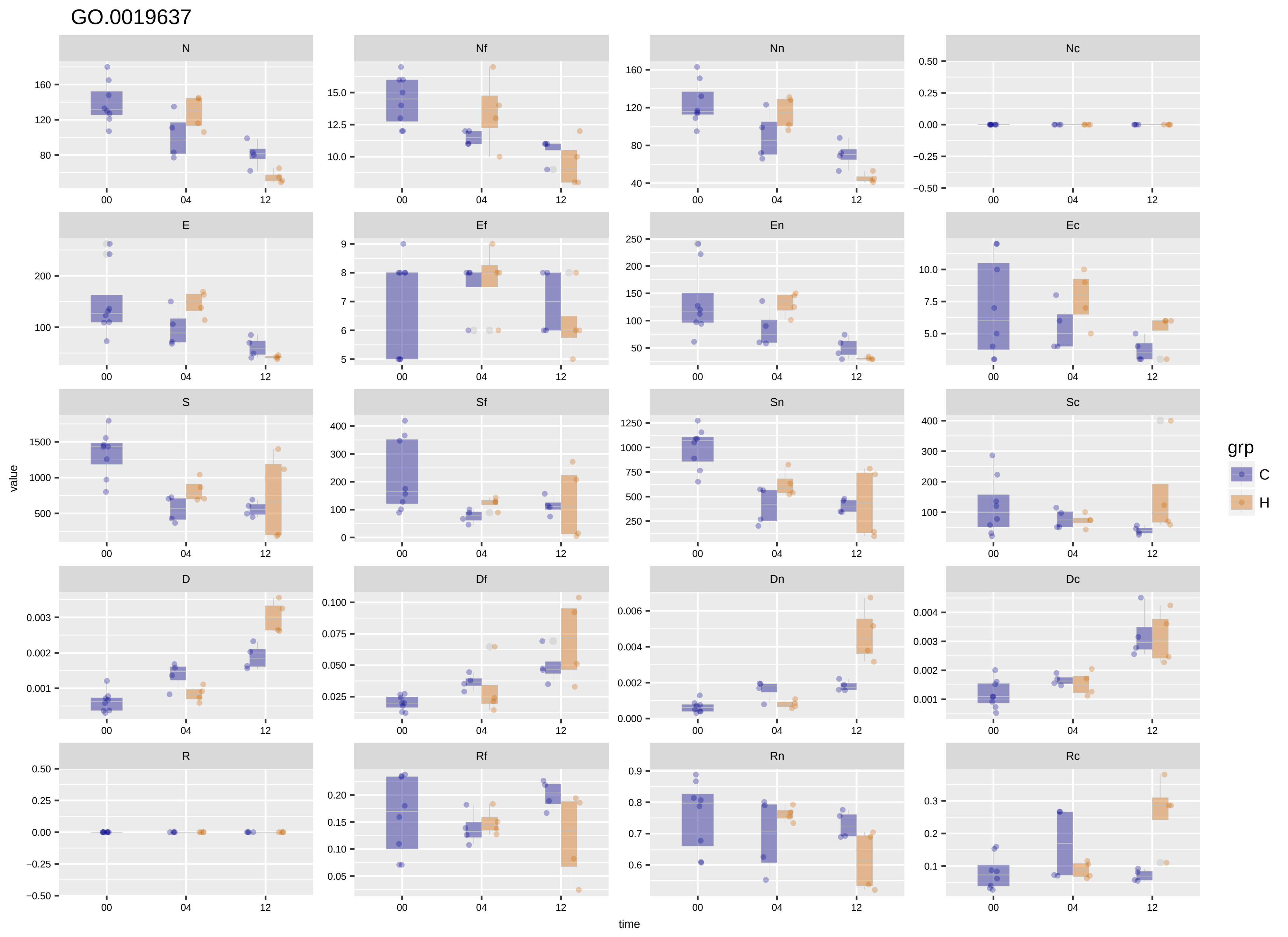

GO.0019637
N
Nf
Nn
Nc
E
Ef
En
Ec
S
Sf
Sn
Sc
D
Df
Dn
Dc
R
Rf
Rn
Rc
value
time
grp
C
H

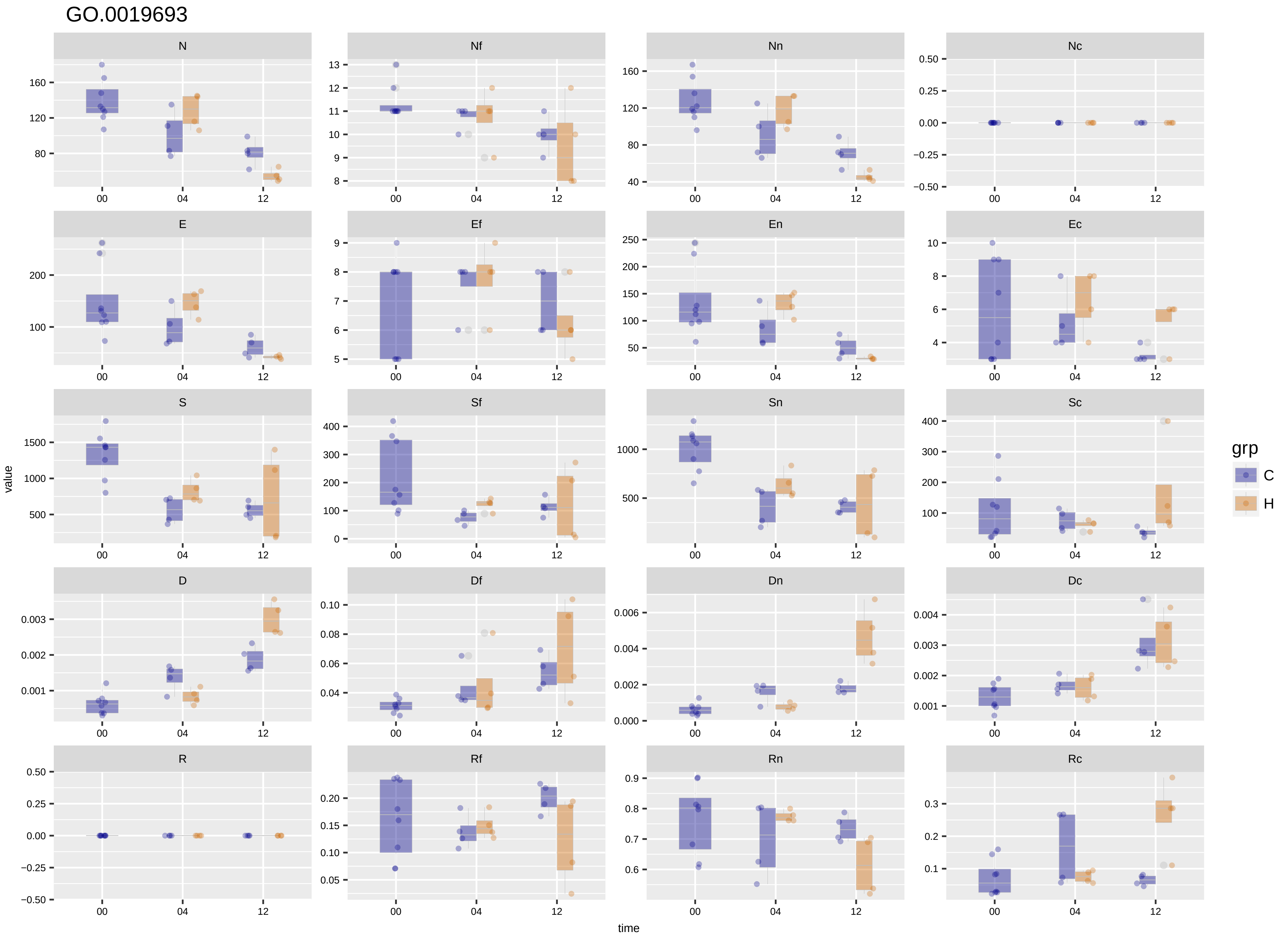
GO.0019693
N
Nf
Nn
Nc
E
Ef
En
Ec
S
Sf
Sn
Sc
D
Df
Dn
Dc
R
Rf
Rn
Rc
value
time
grp
C
H

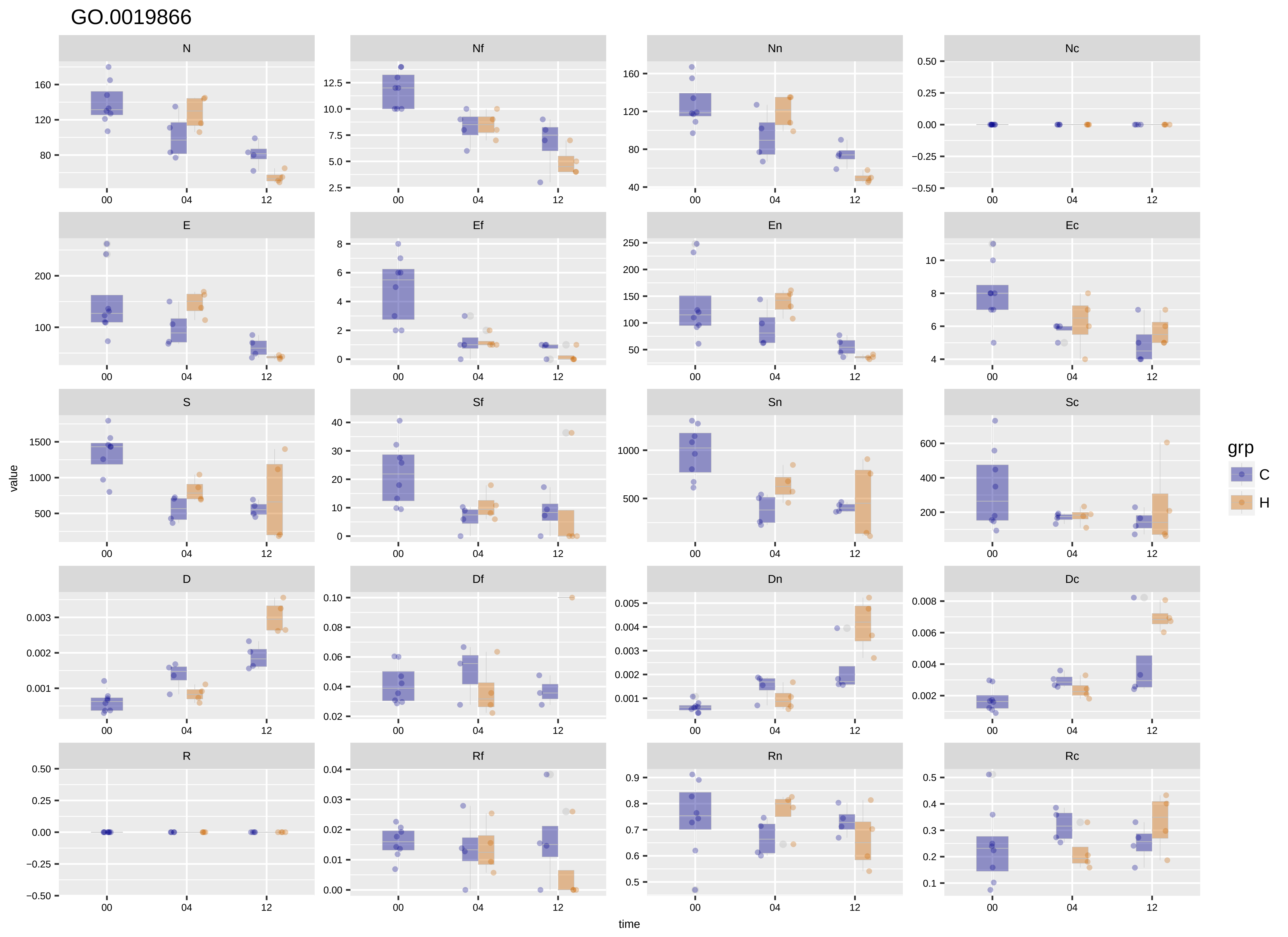
GO.0019866
N
Nf
Nn
Nc
E
Ef
En
Ec
S
Sf
Sn
Sc
D
Df
Dn
Dc
R
Rf
Rn
Rc
value
time
grp
C
H

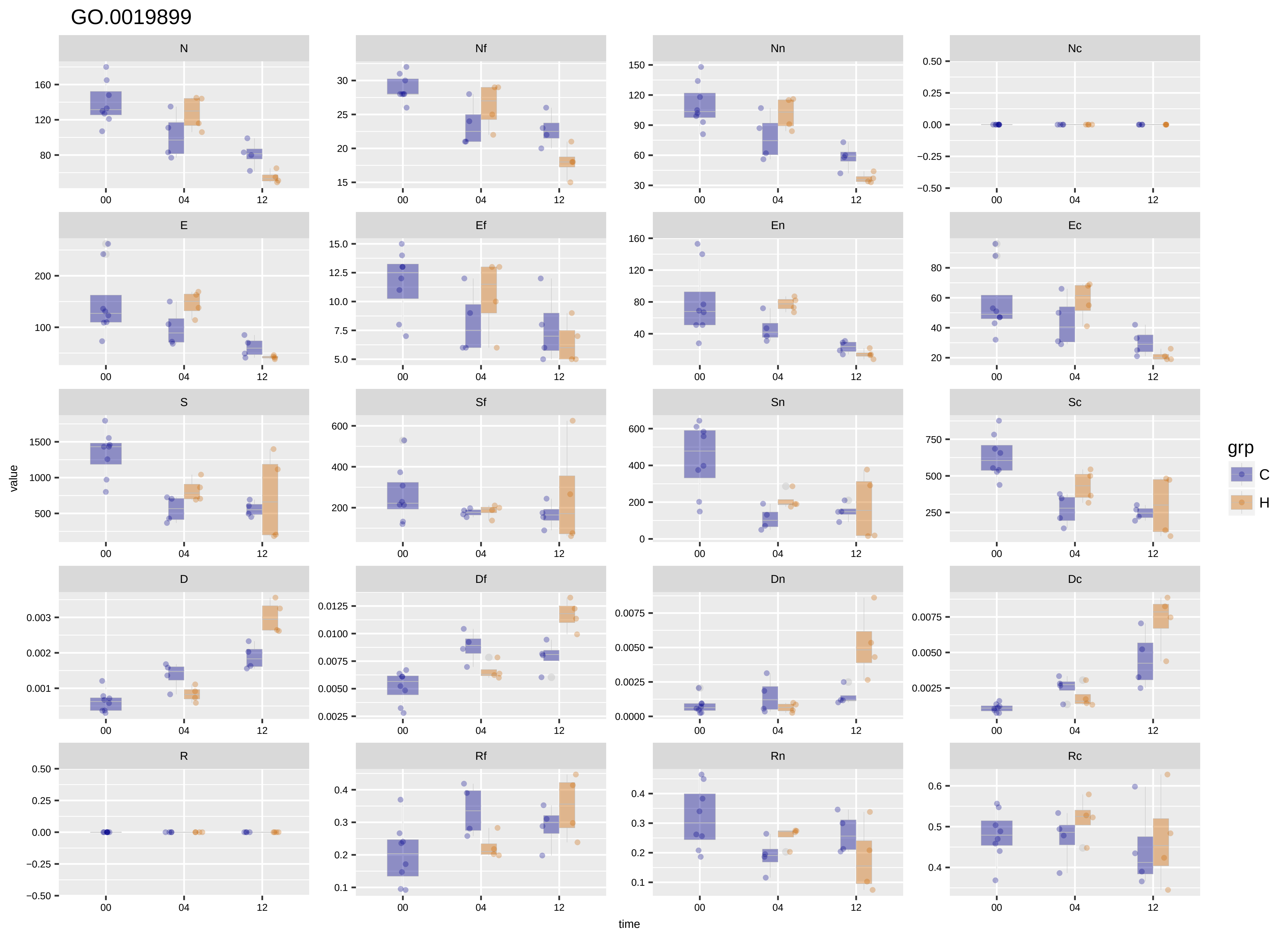
GO.0019899
N
Nf
Nn
Nc
E
Ef
En
Ec
S
Sf
Sn
Sc
D
Df
Dn
Dc
R
Rf
Rn
Rc
00
04
12
value
time
grp
C
H

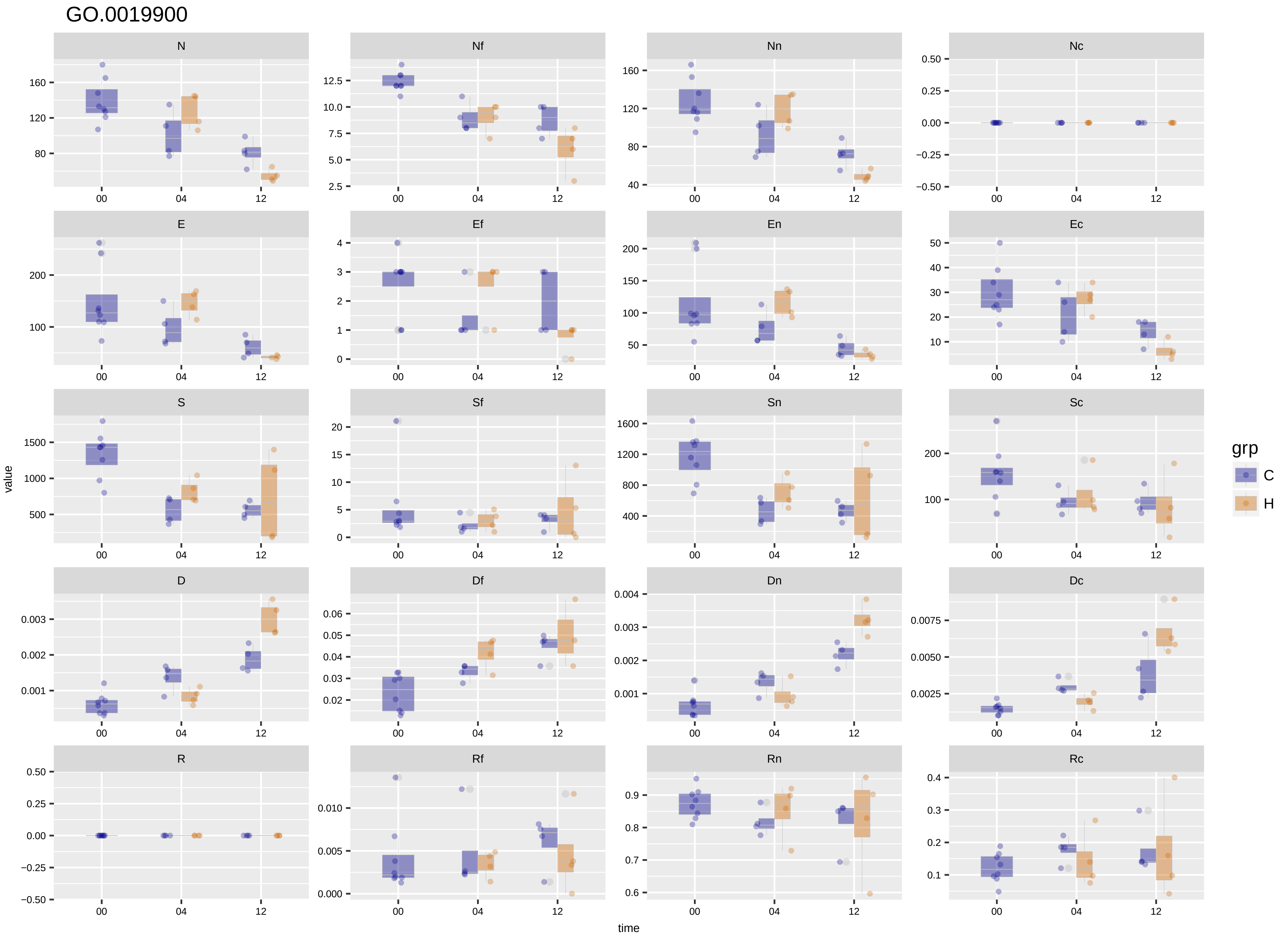

GO.0019900
N
Nf
Nn
Nc
E
Ef
En
Ec
S
Sf
Sn
Sc
D
Df
Dn
Dc
R
Rf
Rn
Rc
value
time
grp
C
H

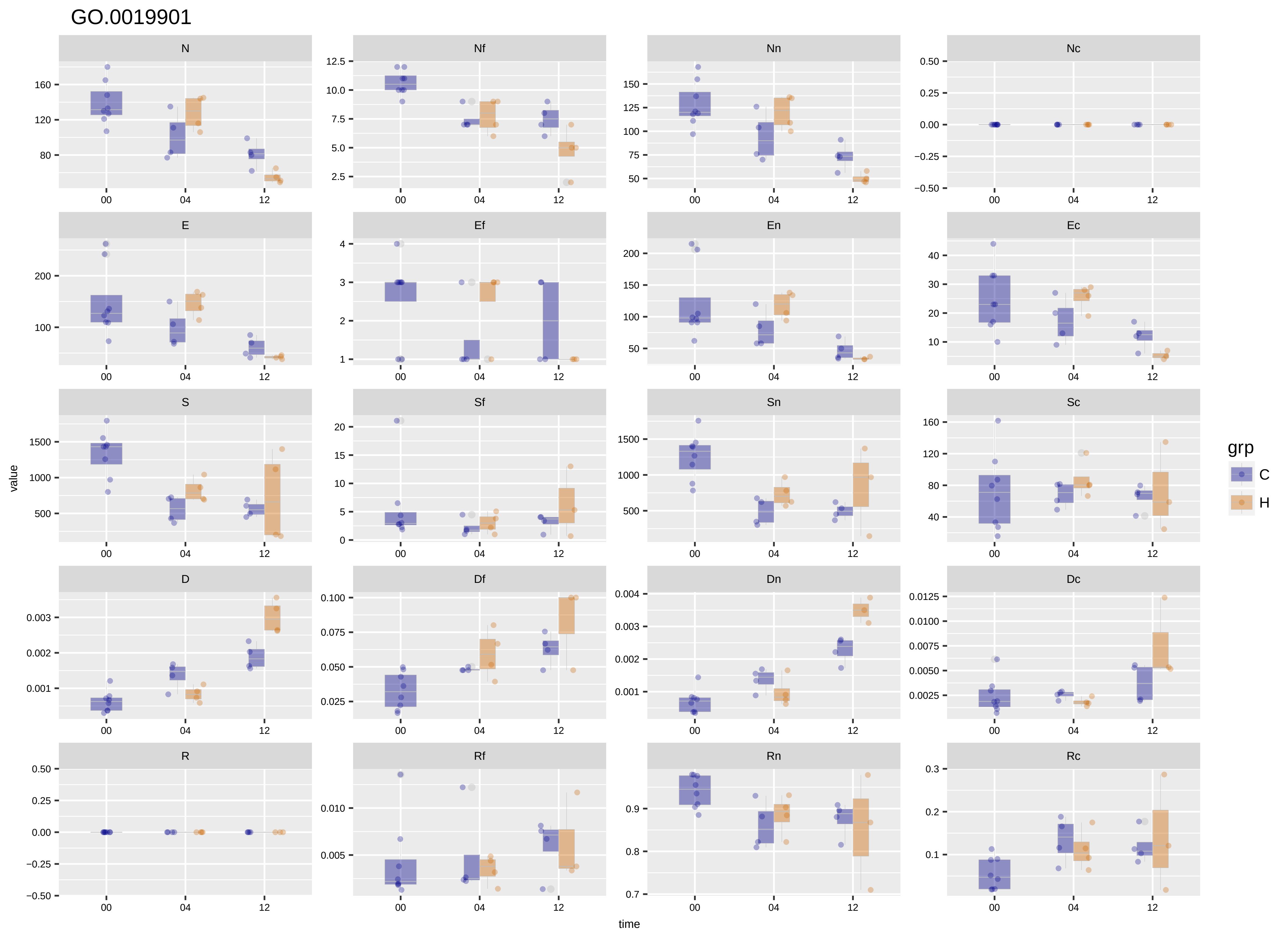
GO.0019901
N
Nf
Nn
Nc
E
Ef
En
Ec
S
Sf
Sn
Sc
D
Df
Dn
Dc
R
Rf
Rn
Rc
value
time
00
04
12
grp
C
H

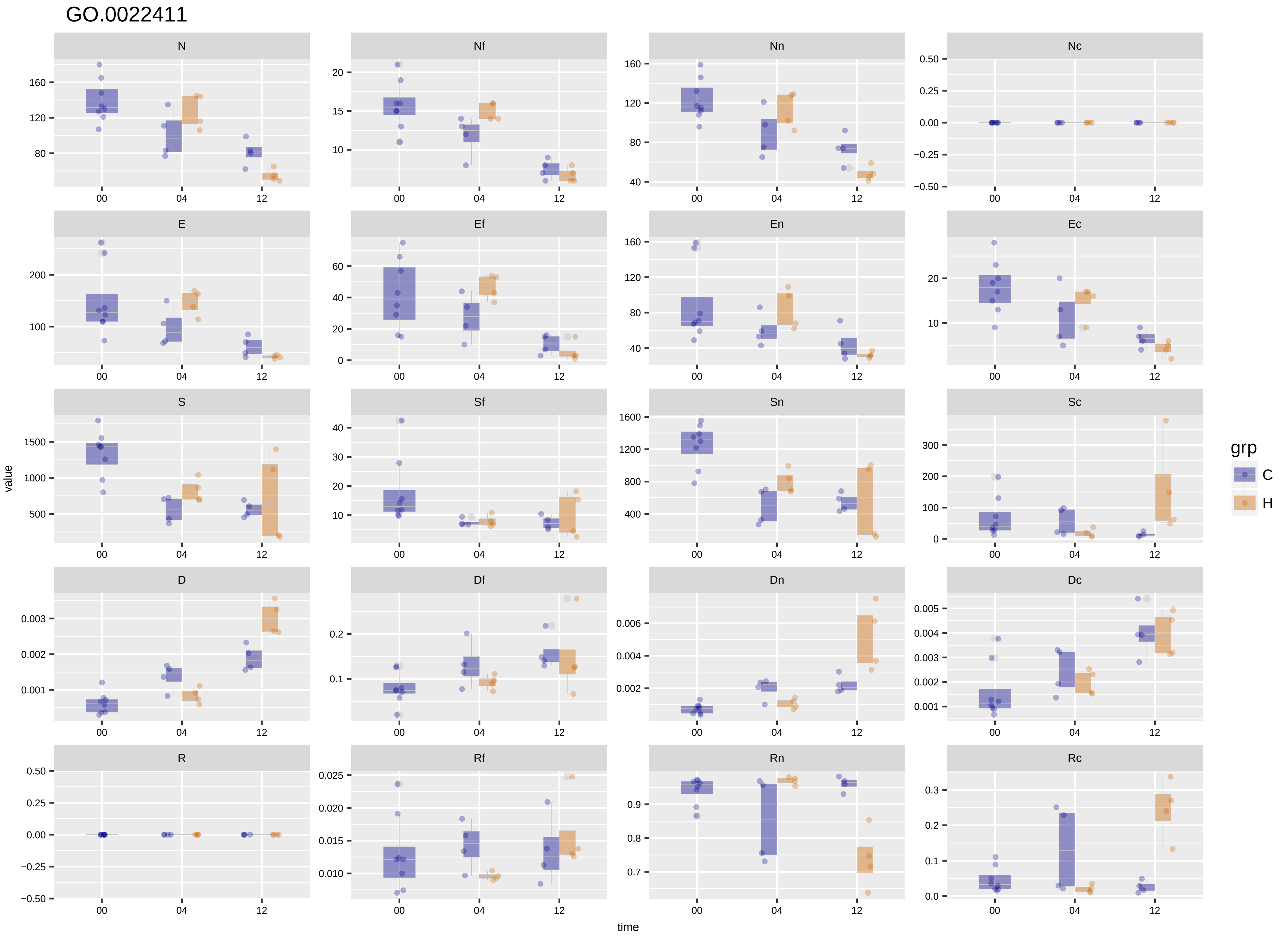

GO.0022411
N
Nf
Nn
Nc
E
Ef
En
Ec
S
Sf
Sn
Sc
D
Df
Dn
Dc
R
Rf
Rn
Rc
value
time
grp
C
H

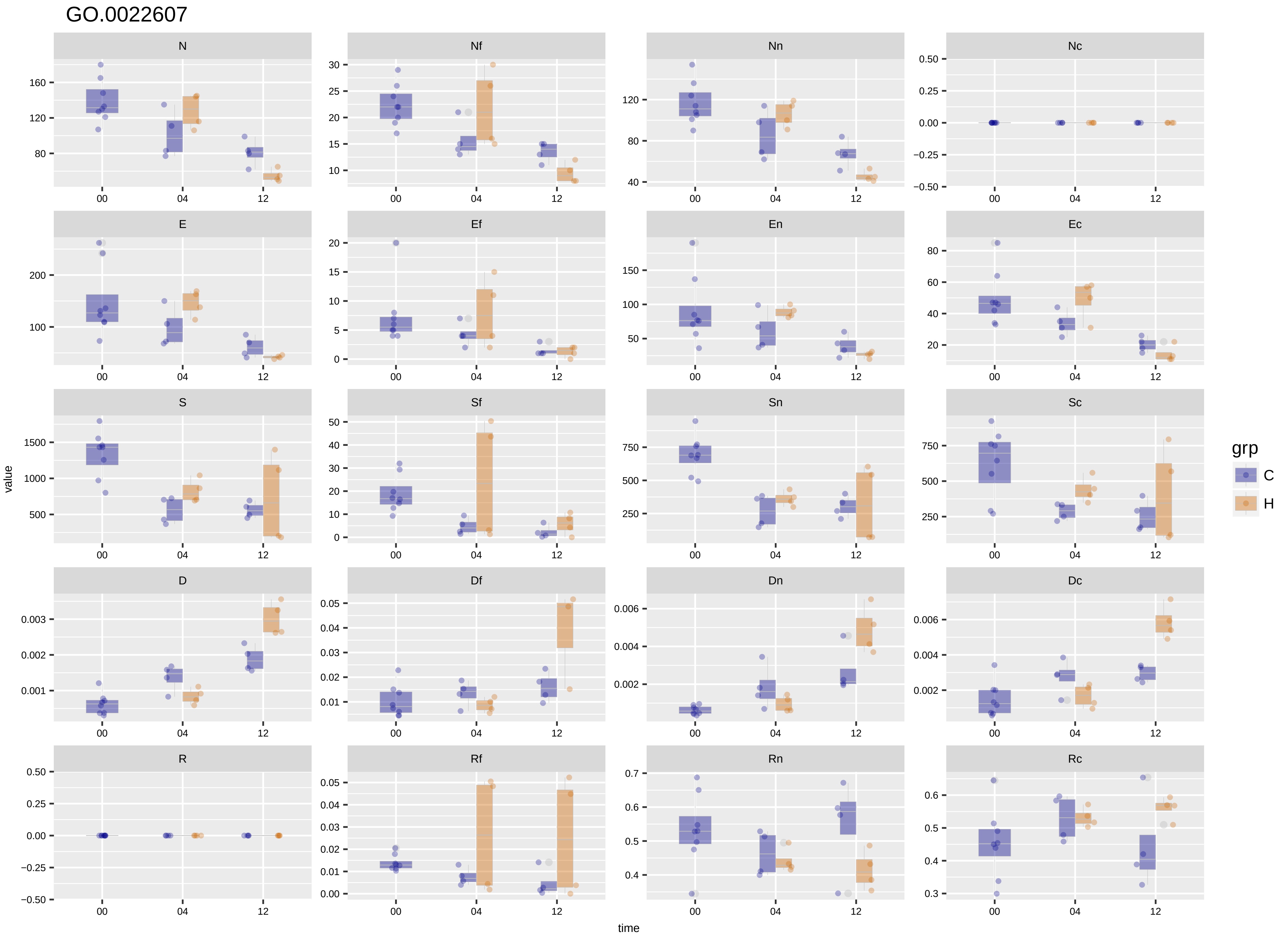

GO.0022607
N
Nf
Nn
Nc
E
Ef
En
Ec
S
Sf
Sn
Sc
D
Df
Dn
Dc
R
Rf
Rn
Rc
value
time
grp
C
H
00
04
12

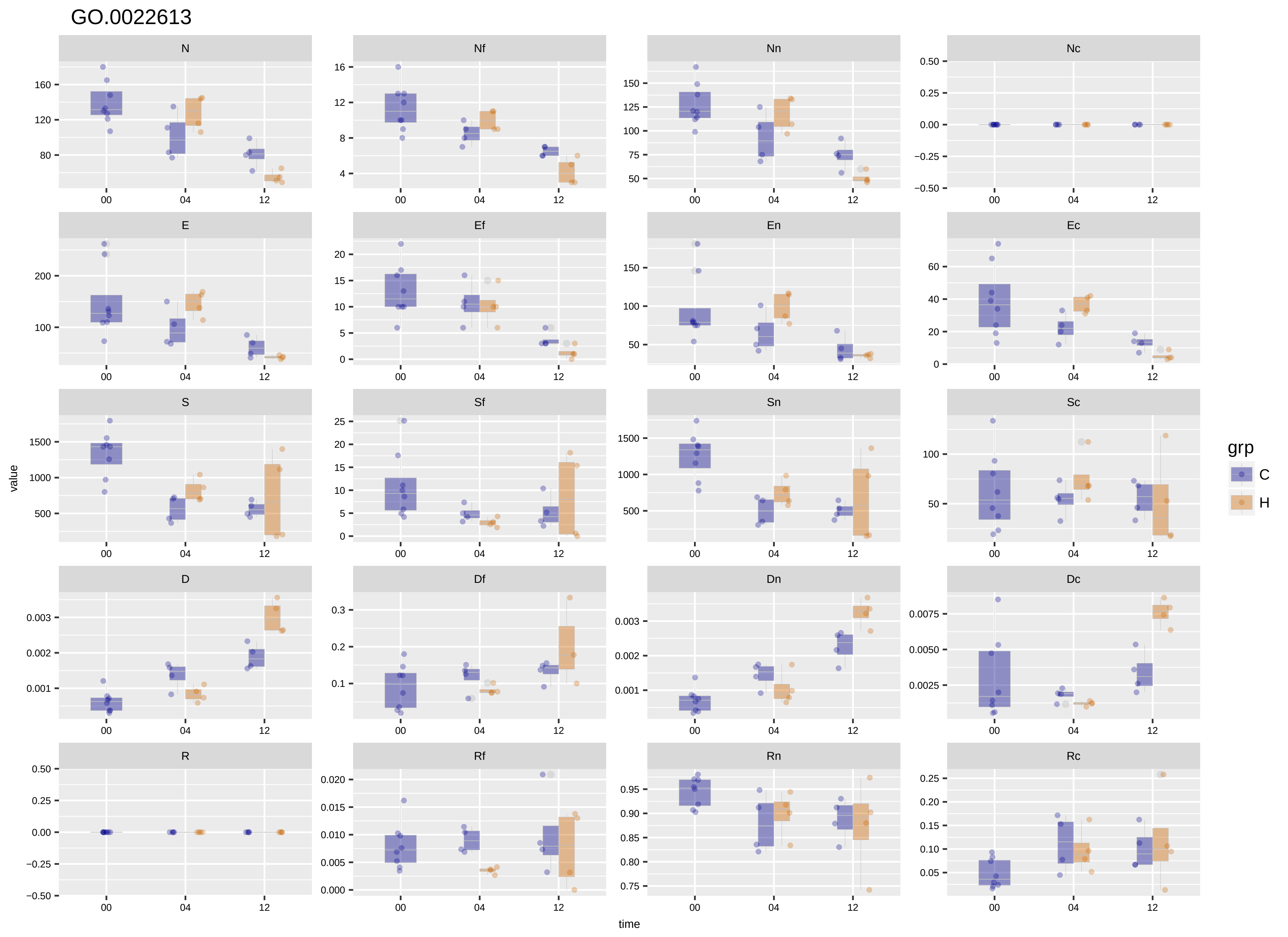
GO.0022613
N
Nf
Nn
Nc
E
Ef
En
Ec
S
Sf
Sn
Sc
D
Df
Dn
Dc
R
Rf
Rn
Rc
00
04
12
value
time
grp
C
H

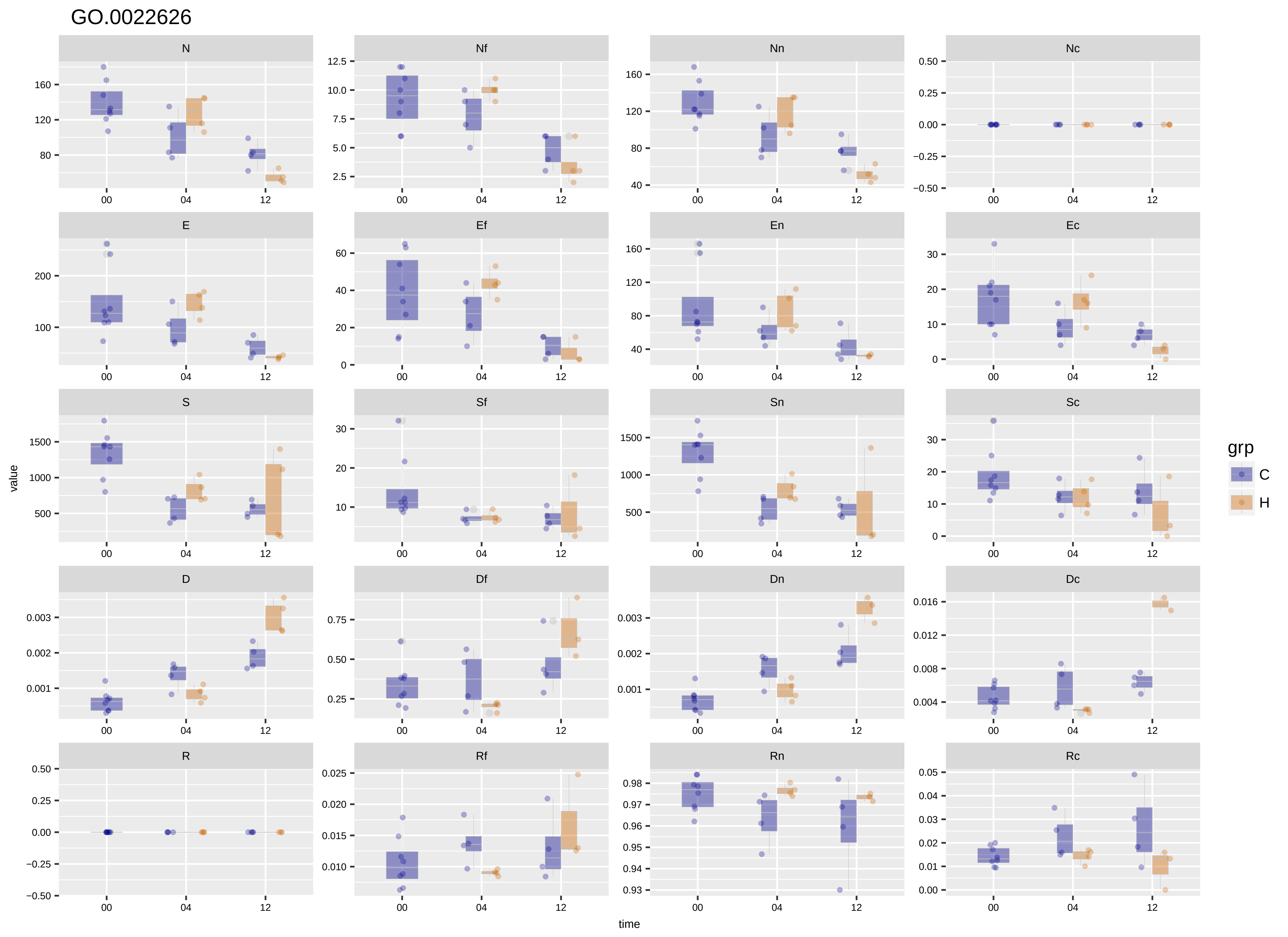

GO.0022626
N
Nf
Nn
Nc
E
Ef
En
Ec
S
Sf
Sn
Sc
D
Df
Dn
Dc
R
Rf
Rn
Rc
value
time
00
04
12
grp
C
H

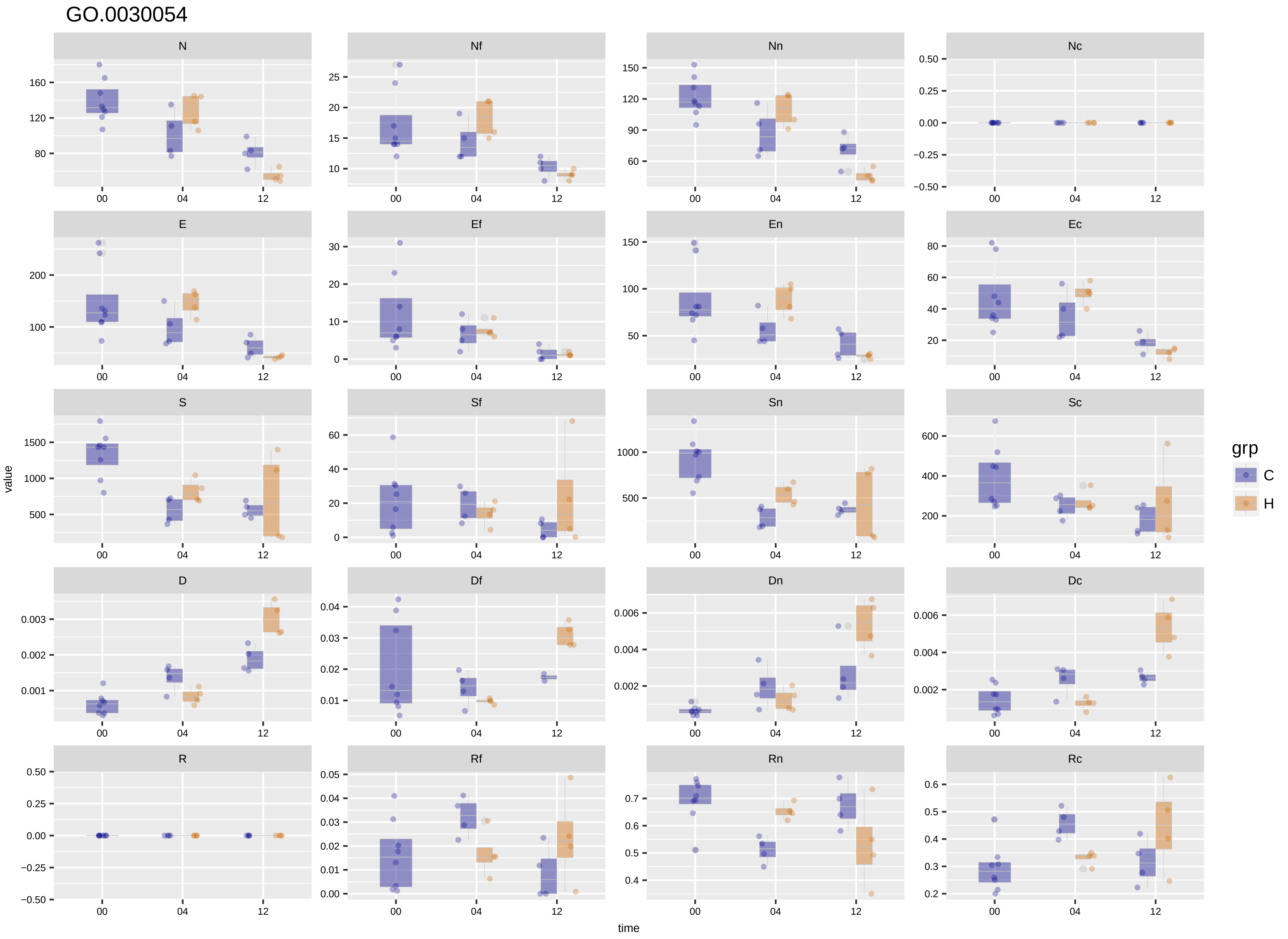

GO.0030054
N
Nf
Nn
Nc
E
Ef
En
Ec
S
Sf
Sn
Sc
D
Df
Dn
Dc
R
Rf
Rn
Rc
value
time
grp
C
H

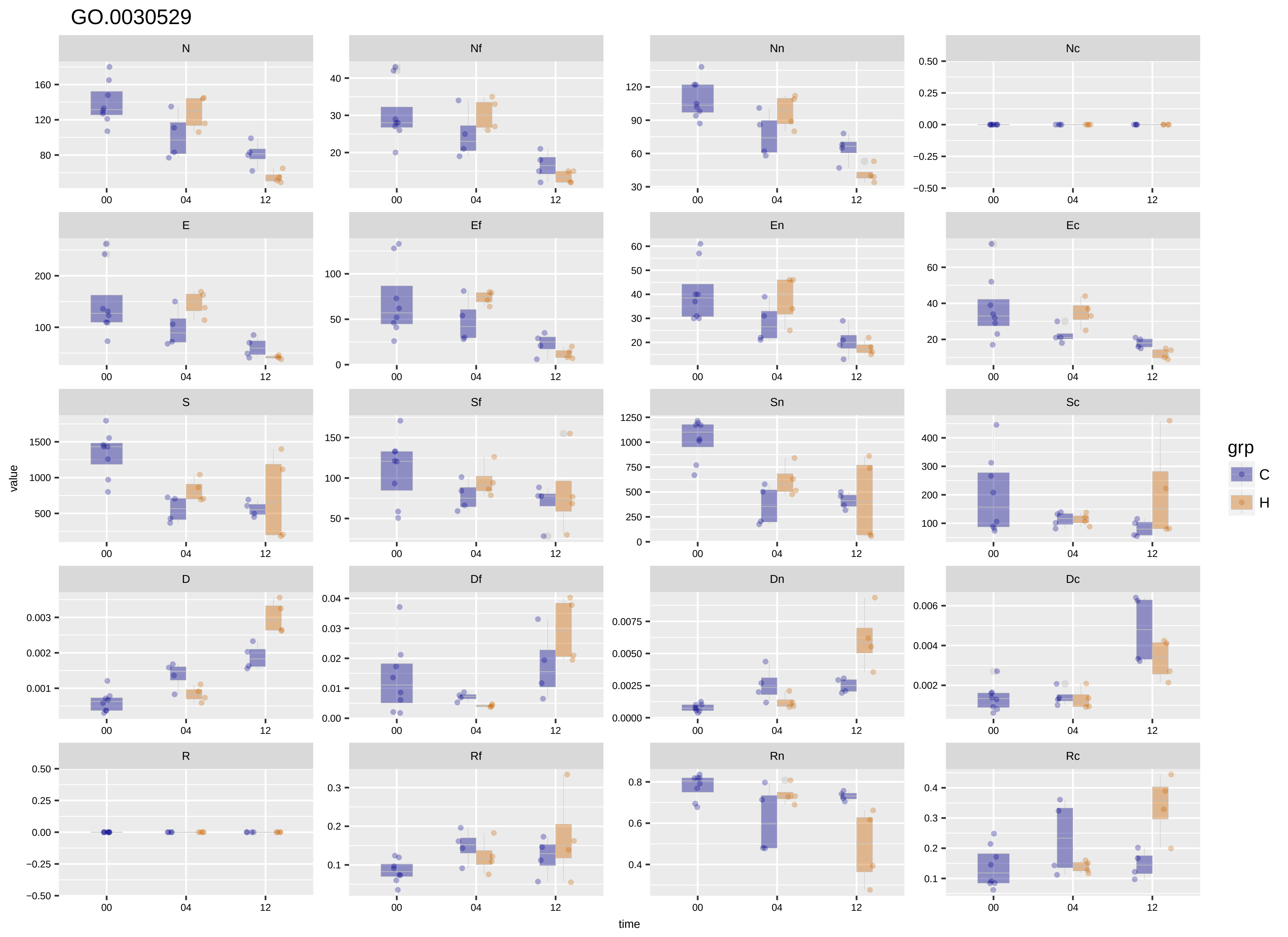
GO.0030529
N
Nf
Nn
Nc
E
Ef
En
Ec
S
Sf
Sn
Sc
D
Df
Dn
Dc
R
Rf
Rn
Rc
value
time
grp
C
H

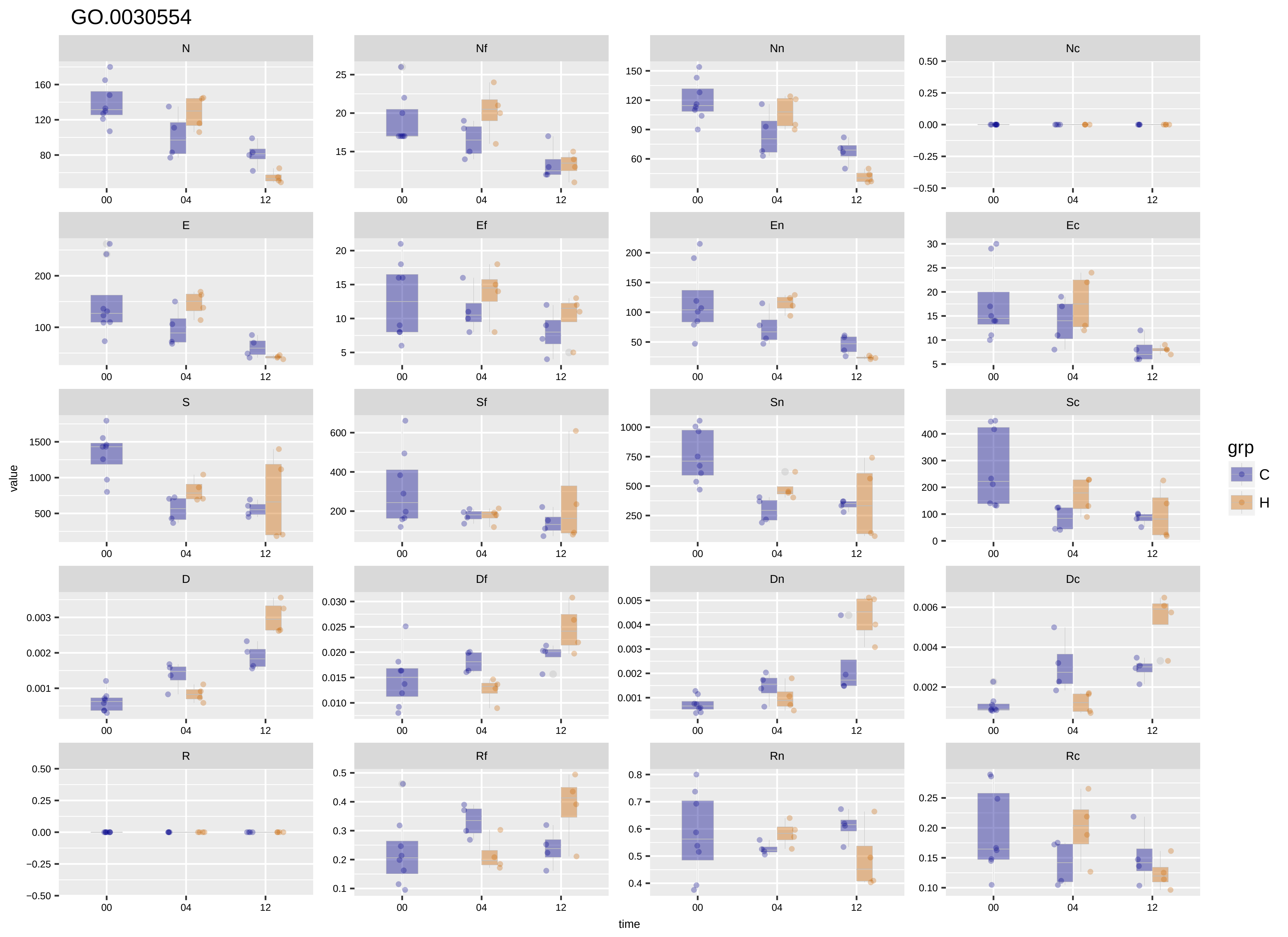

GO.0030554
N
Nf
Nn
Nc
E
Ef
En
Ec
S
Sf
Sn
Sc
D
Df
Dn
Dc
R
Rf
Rn
Rc
value
time
grp
C
H
00
04
12

GO.0031625

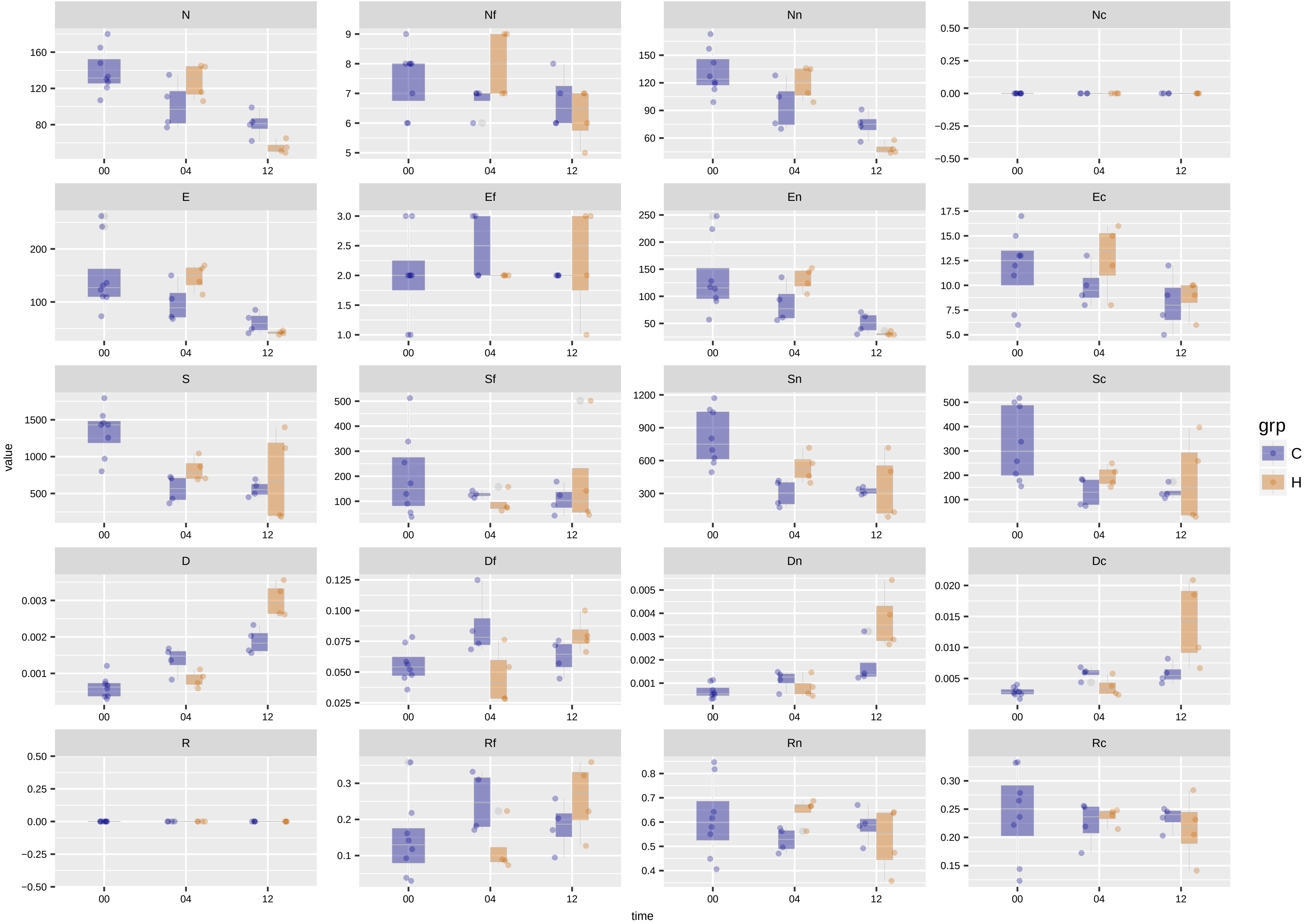

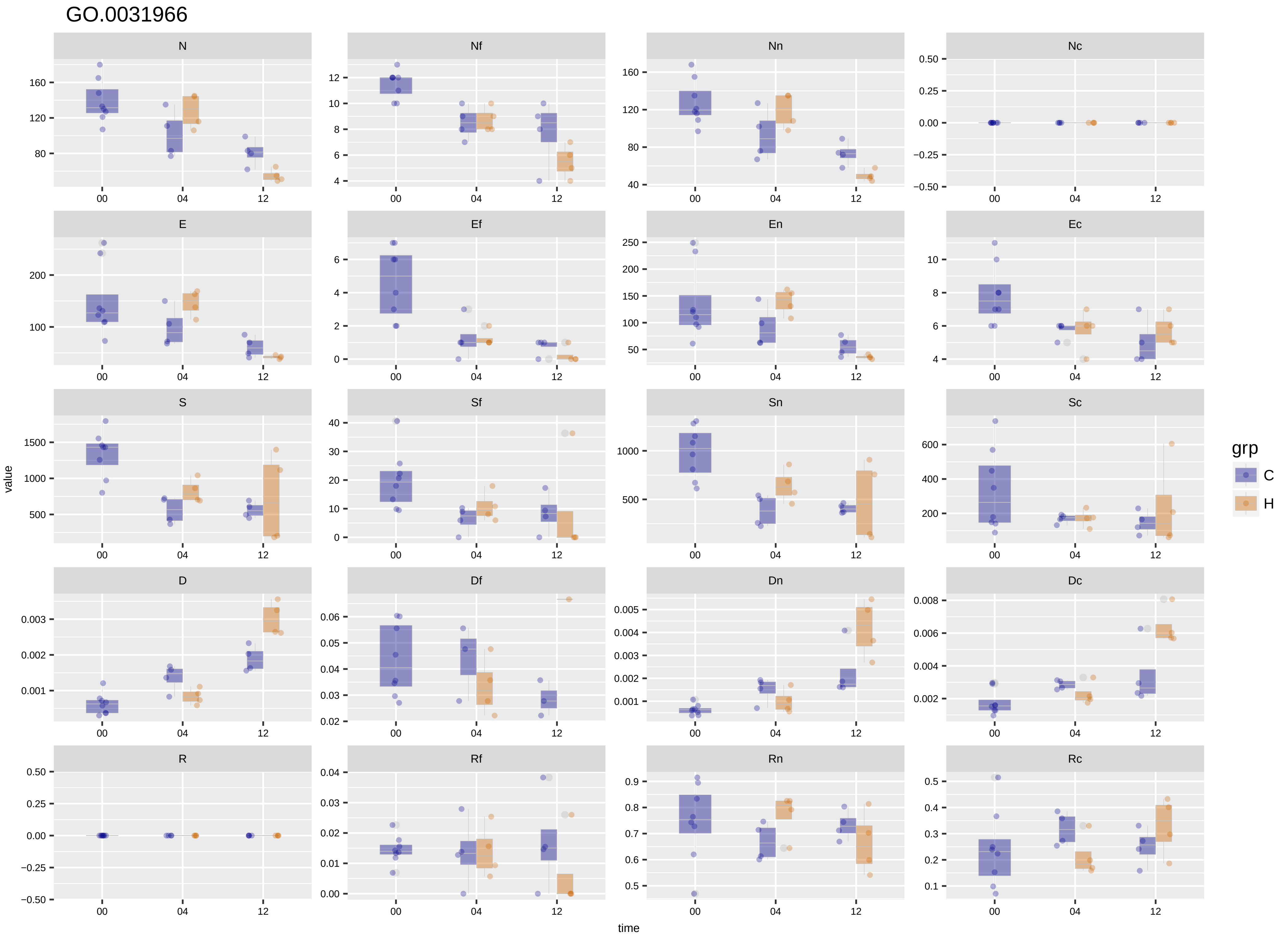

GO.0031966
N
Nf
Nn
Nc
E
Ef
En
Ec
S
Sf
Sn
Sc
D
Df
Dn
Dc
R
Rf
Rn
Rc
00
04
12
value
time
grp
C
H

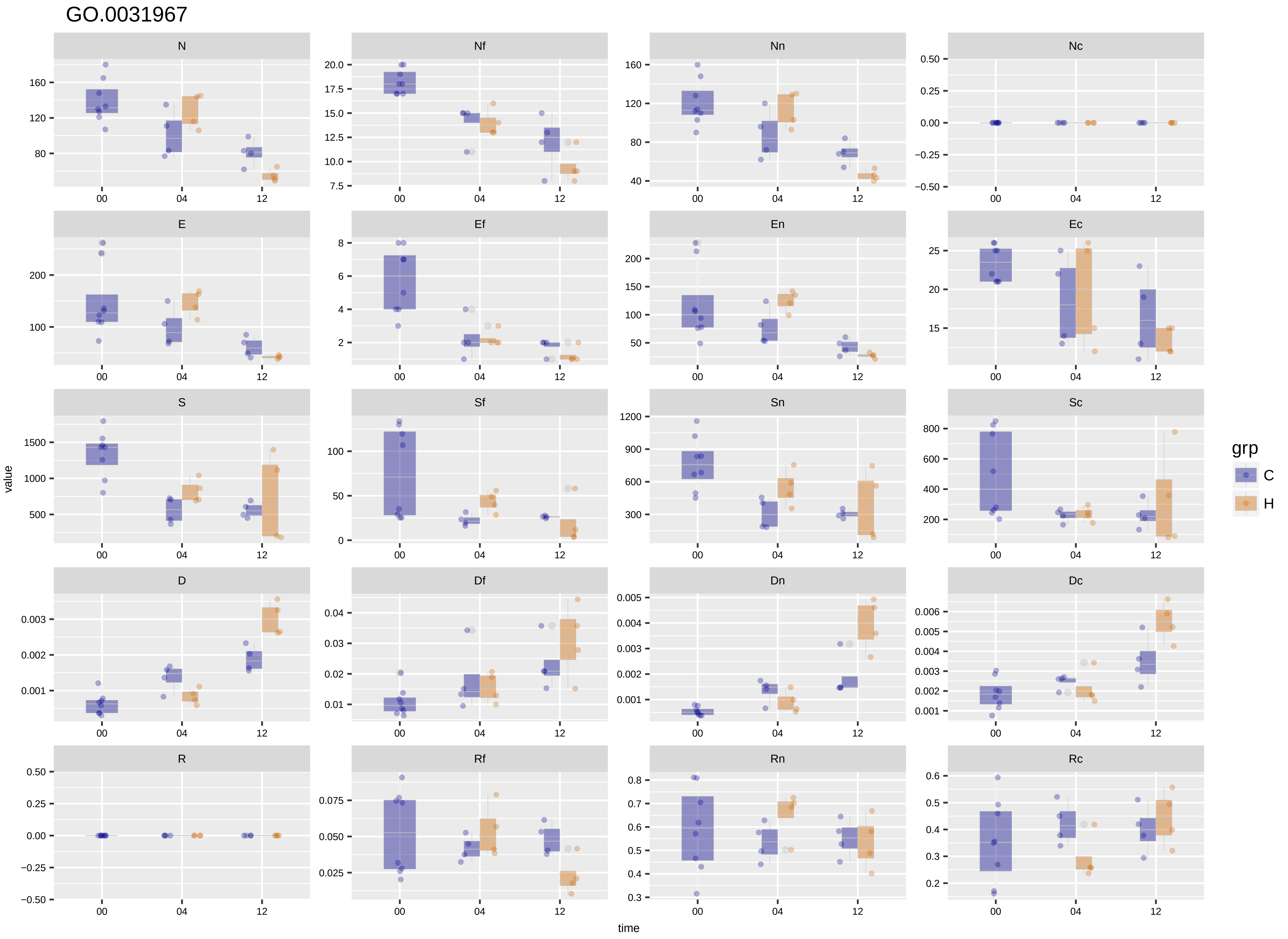
GO.0031967
N
Nf
Nn
Nc
E
Ef
En
Ec
S
Sf
Sn
Sc
D
Df
Dn
Dc
R
Rf
Rn
Rc
value
time
grp
C
H

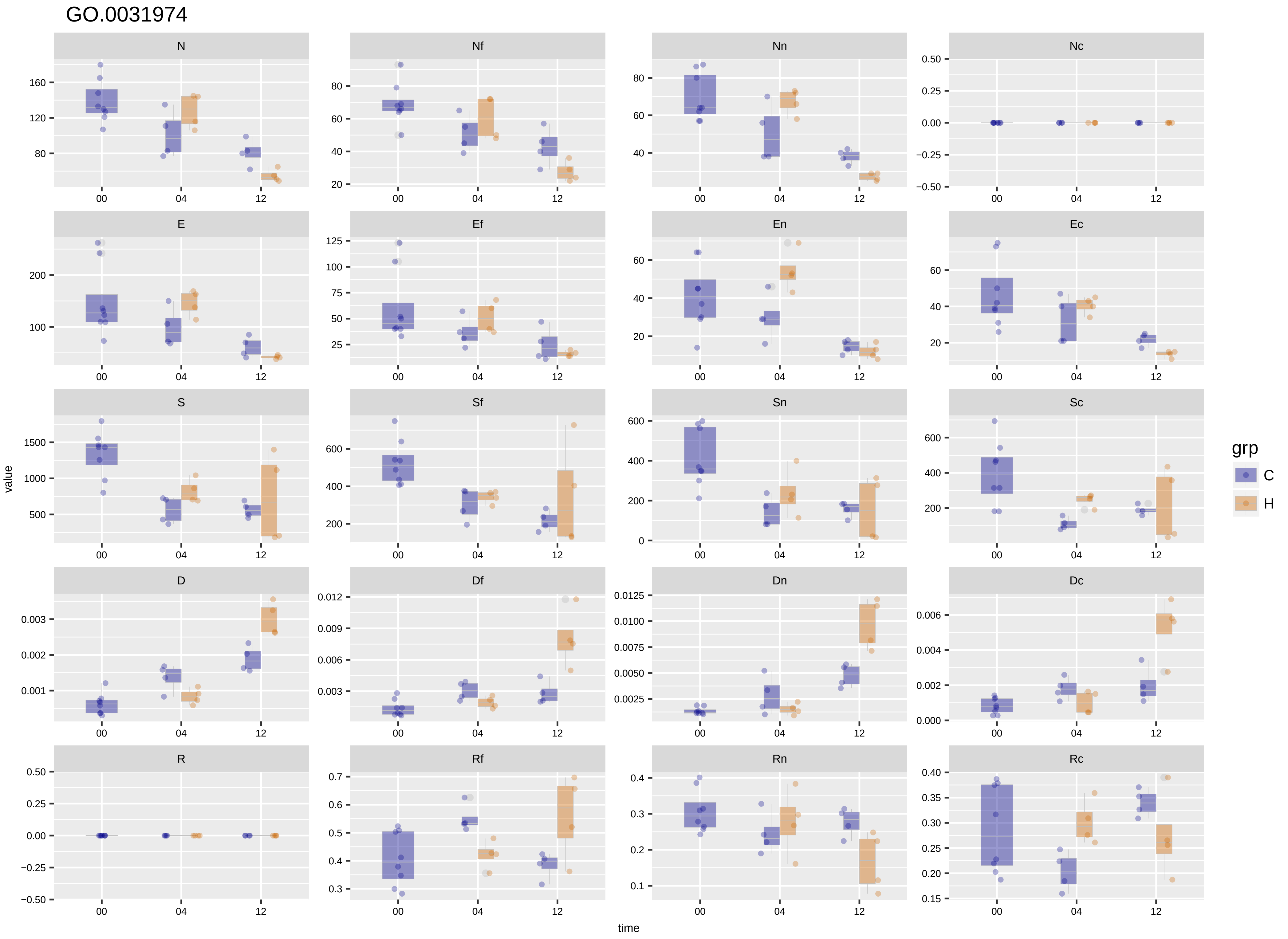

GO.0031974
N
Nf
Nn
Nc
E
Ef
En
Ec
S
Sf
Sn
Sc
D
Df
Dn
Dc
R
Rf
Rn
Rc
value
time
grp
C
H

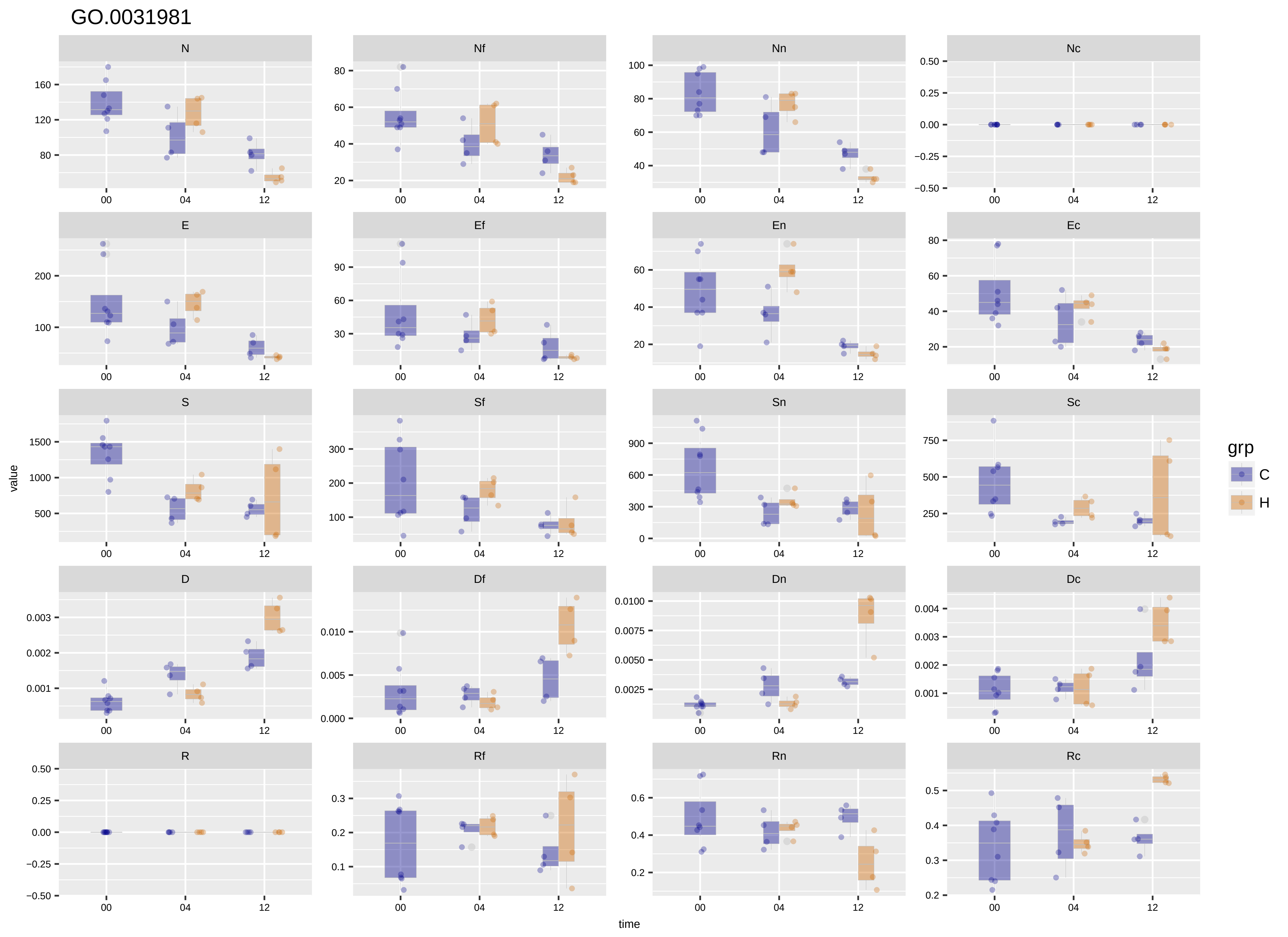

GO.0031981
N
Nf
Nn
Nc
E
Ef
En
Ec
S
Sf
Sn
Sc
D
Df
Dn
Dc
R
Rf
Rn
Rc
value
time
00
04
12
grp
C
H

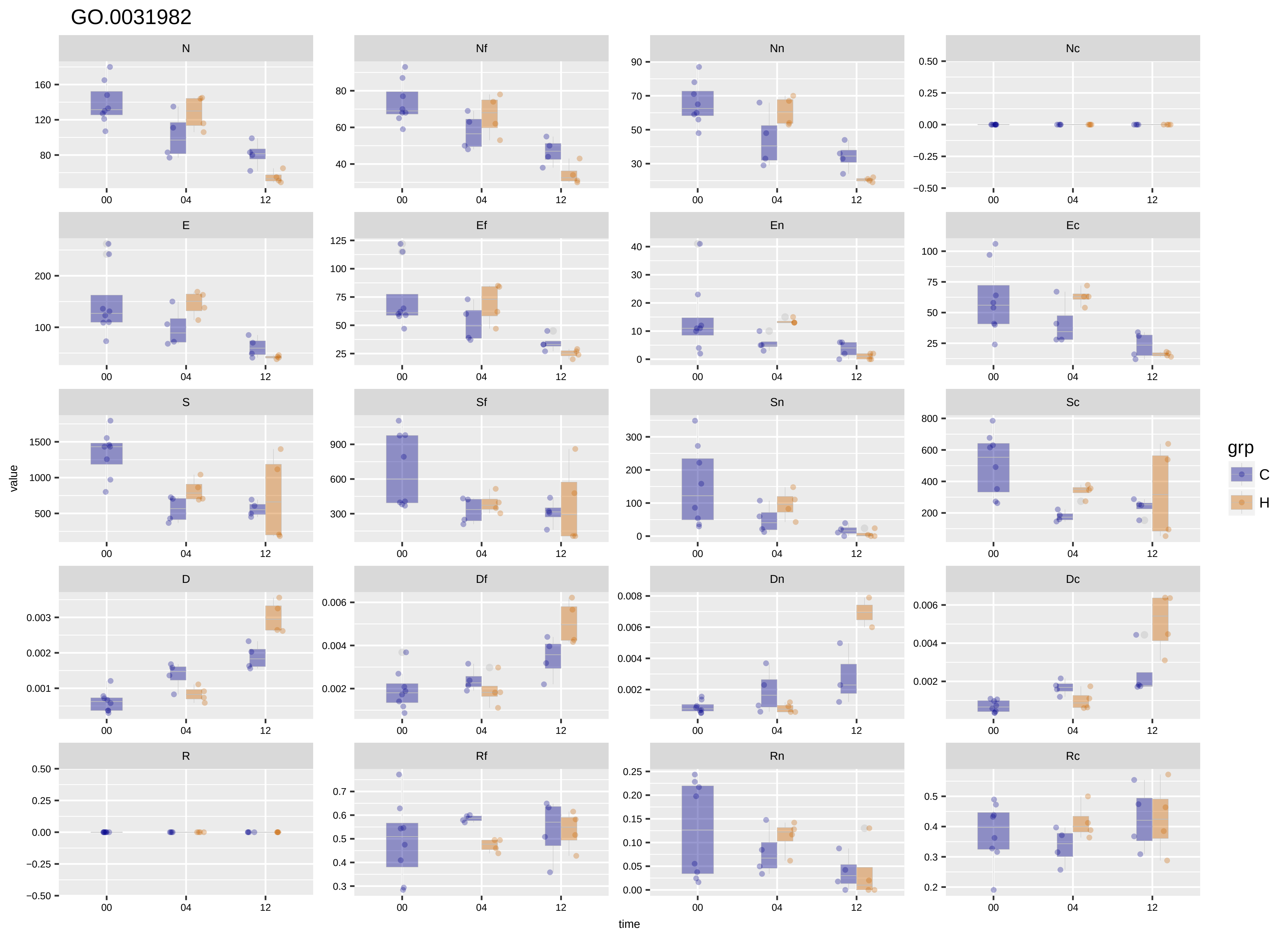

GO.0031982
N
Nf
Nn
Nc
E
Ef
En
Ec
S
Sf
Sn
Sc
D
Df
Dn
Dc
R
Rf
Rn
Rc
value
time
00
04
12
grp
C
H

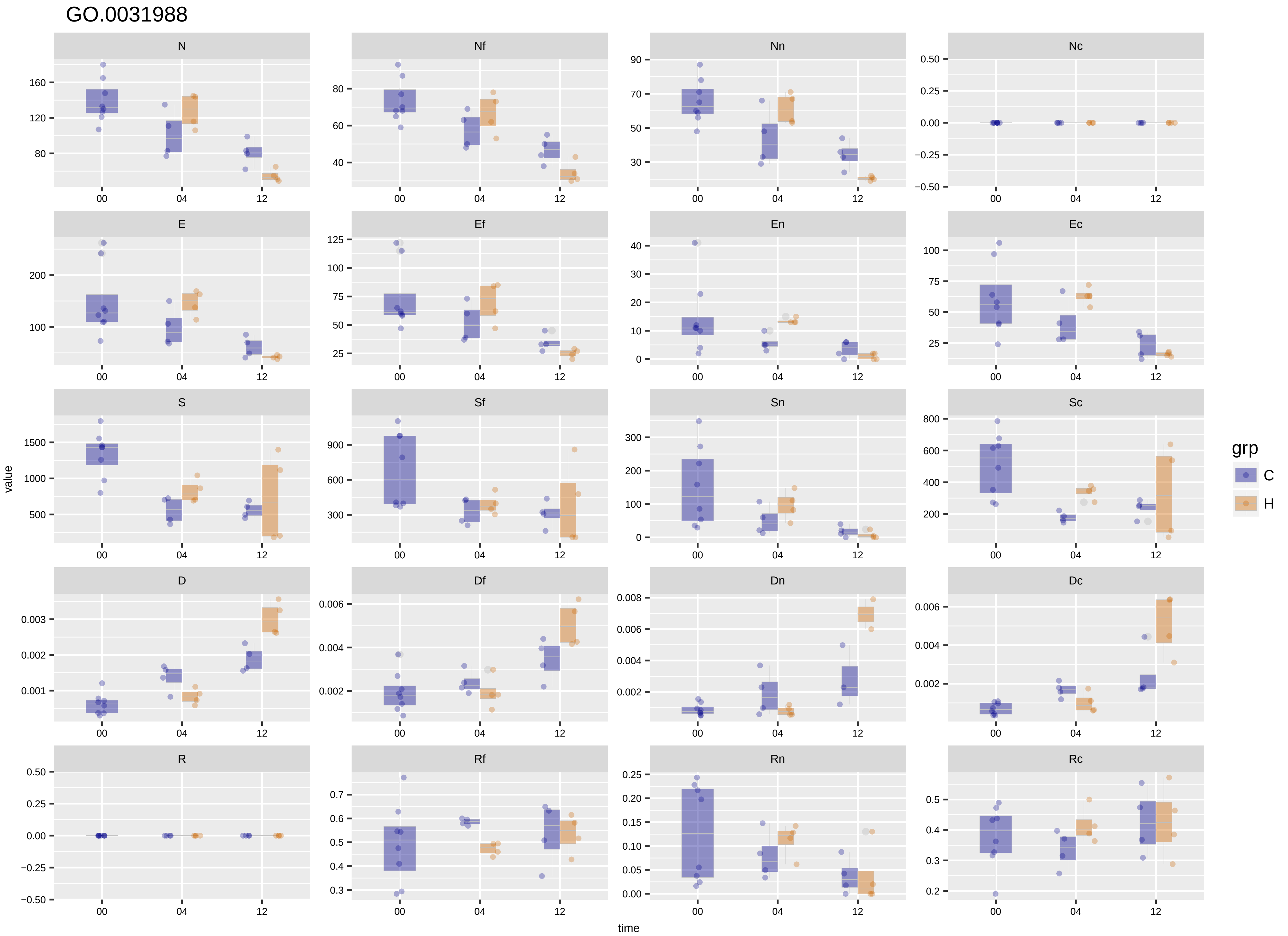

GO.0031988
N
Nf
Nn
Nc
E
Ef
En
Ec
S
Sf
Sn
Sc
D
Df
Dn
Dc
R
Rf
Rn
Rc
00
04
12
value
time
grp
C
H

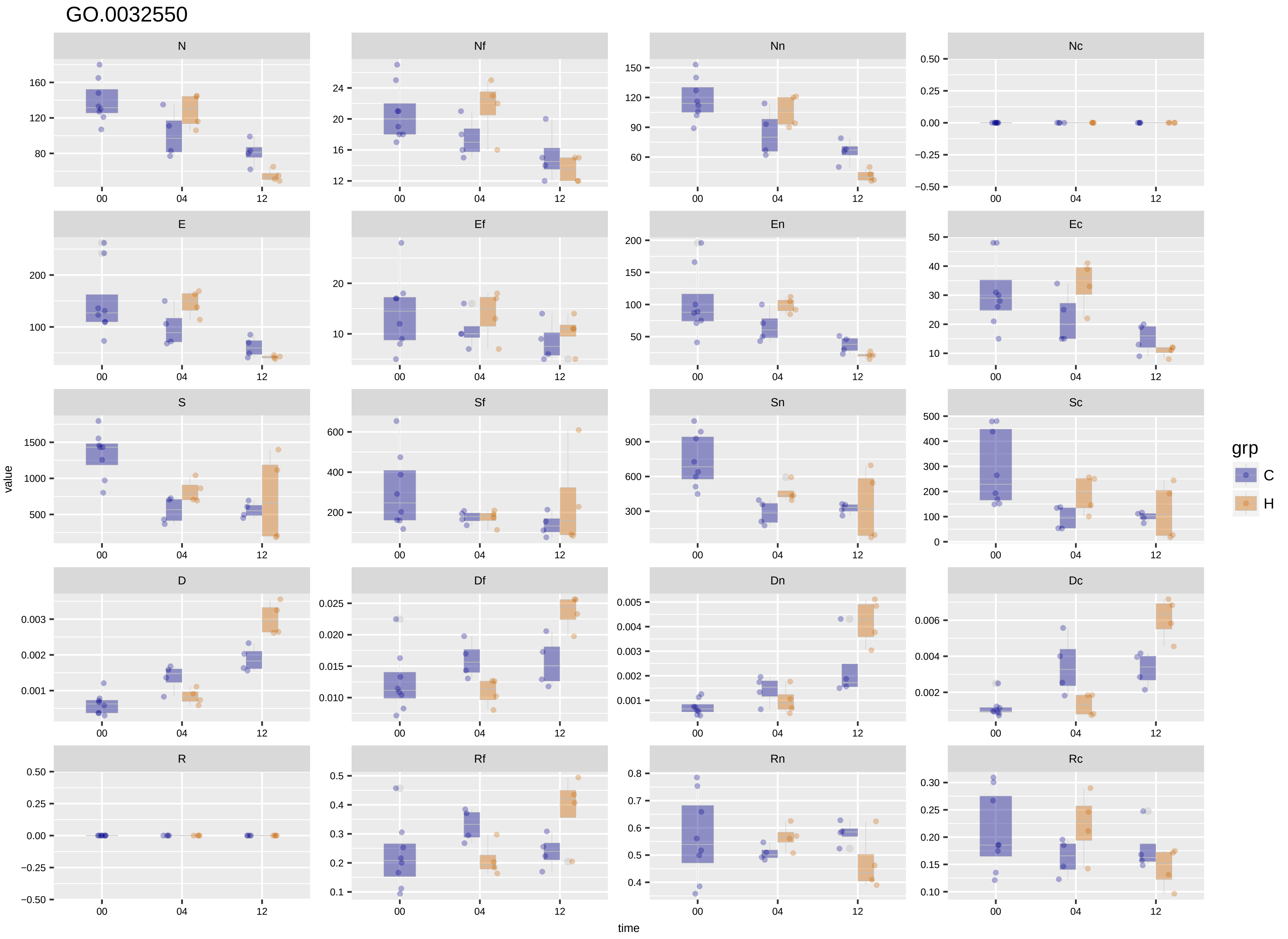

GO.0032550
N
Nf
Nn
Nc
E
Ef
En
Ec
S
Sf
Sn
Sc
D
Df
Dn
Dc
R
Rf
Rn
Rc
value
time
00
04
12
grp
C
H

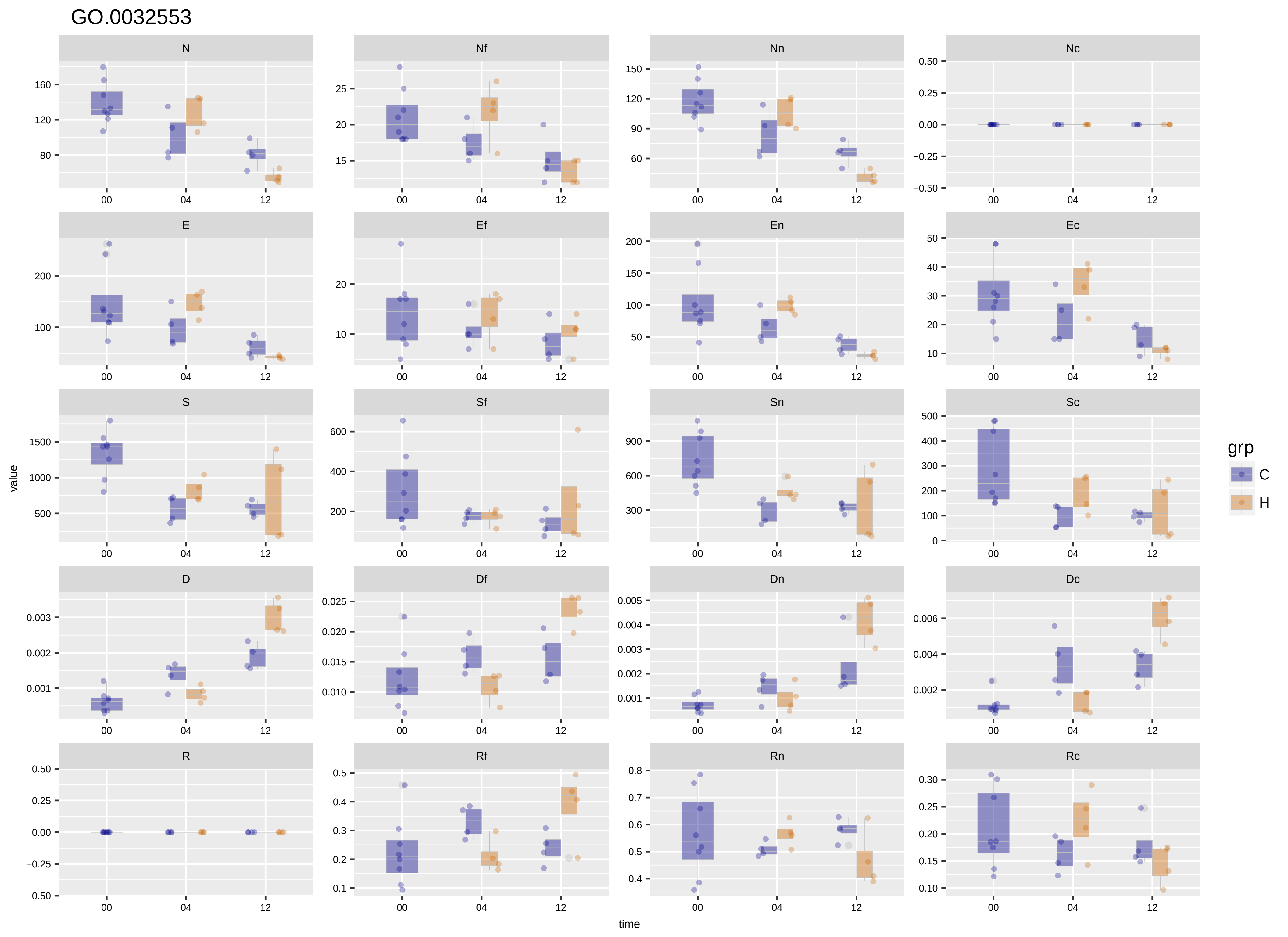

GO.0032553
N
Nf
Nn
Nc
E
Ef
En
Ec
S
Sf
Sn
Sc
D
Df
Dn
Dc
R
Rf
Rn
Rc
value
time
grp
C
H

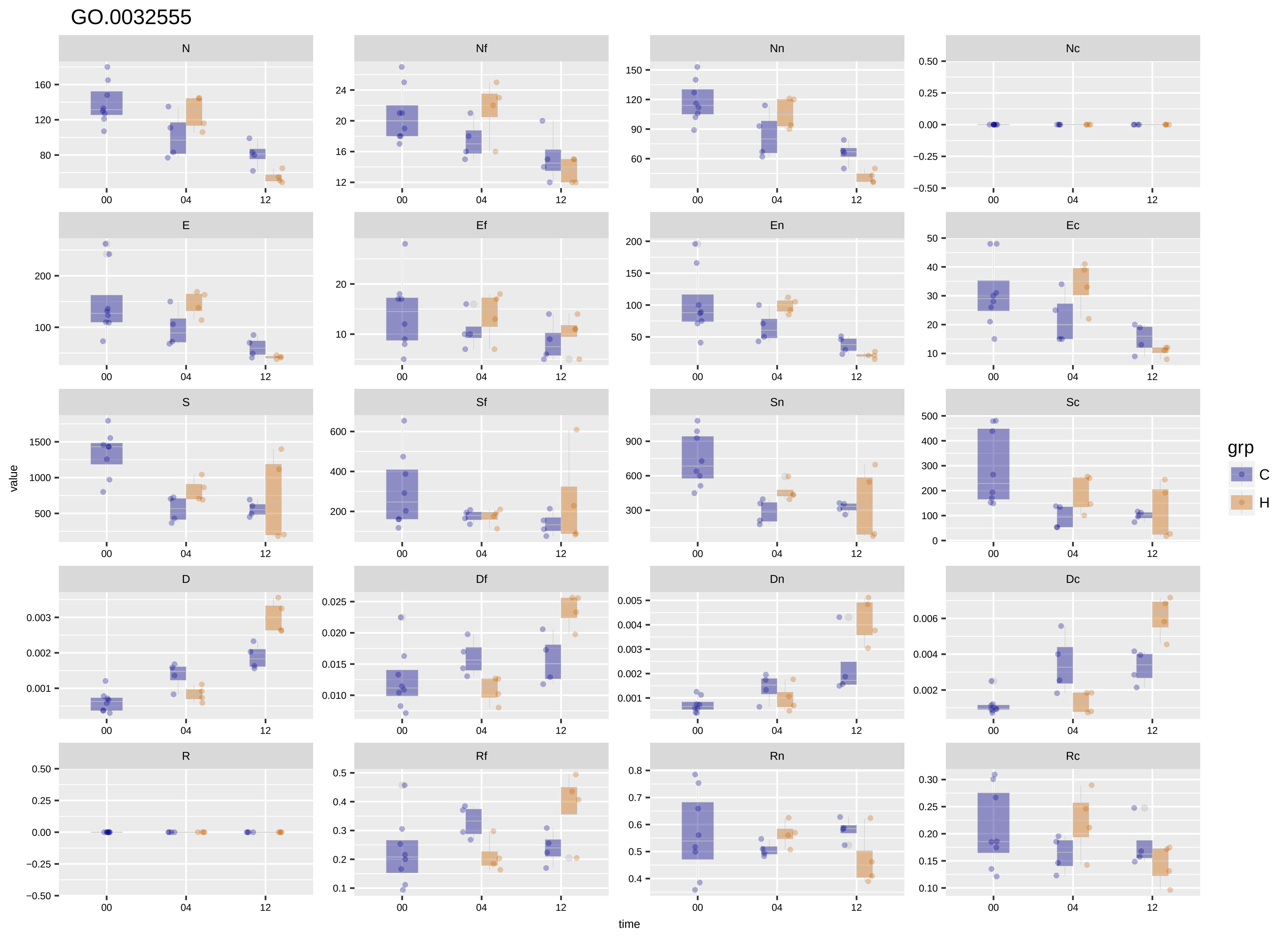

GO.0032555
N
Nf
Nn
Nc
E
Ef
En
Ec
S
Sf
Sn
Sc
D
Df
Dn
Dc
R
Rf
Rn
Rc
value
time
grp
C
H

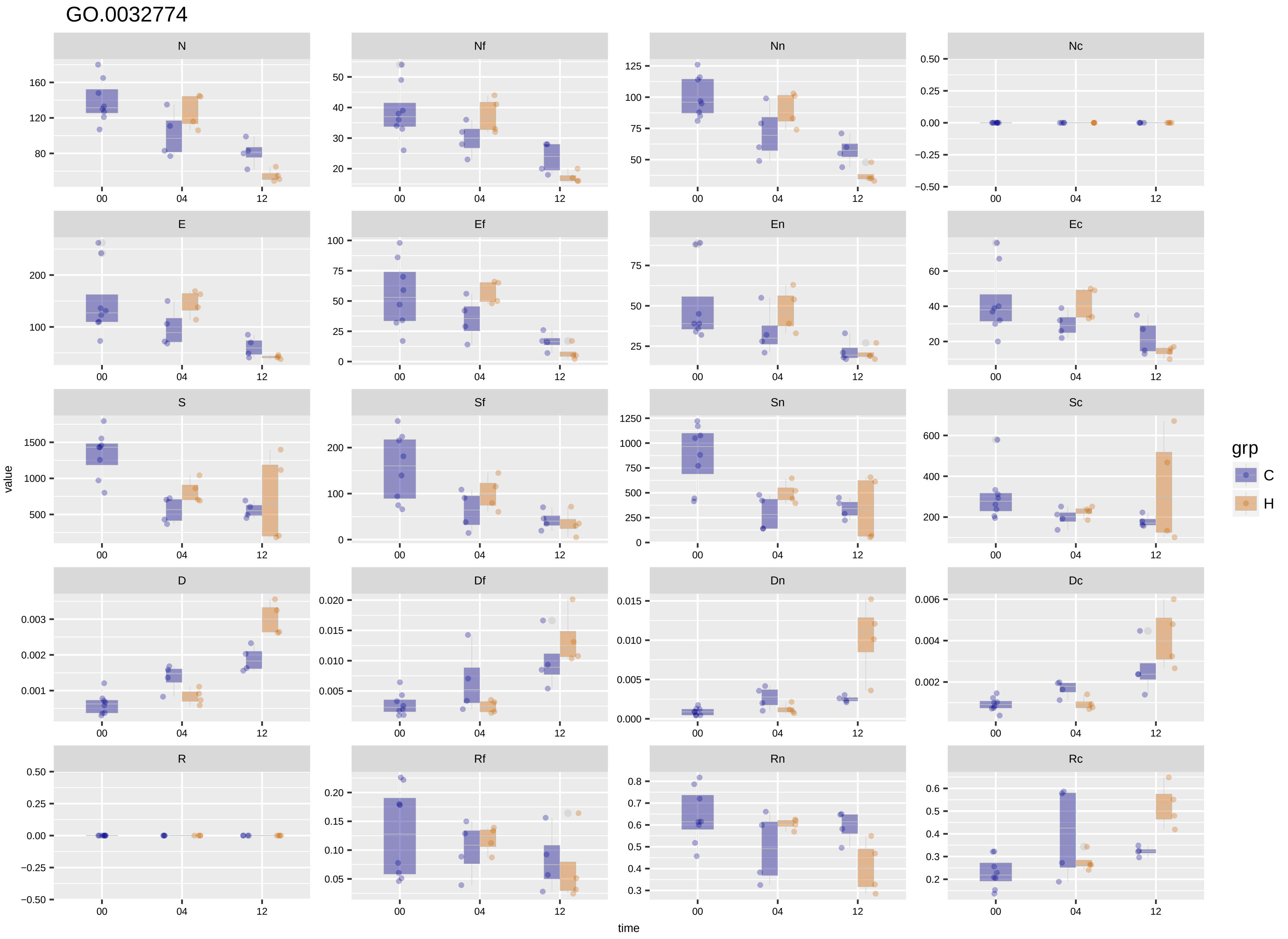

GO.0032774
N
Nf
Nn
Nc
E
Ef
En
Ec
S
Sf
Sn
Sc
D
Df
Dn
Dc
R
Rf
Rn
Rc
00
04
12
value
time
grp
C
H

GO.0032991

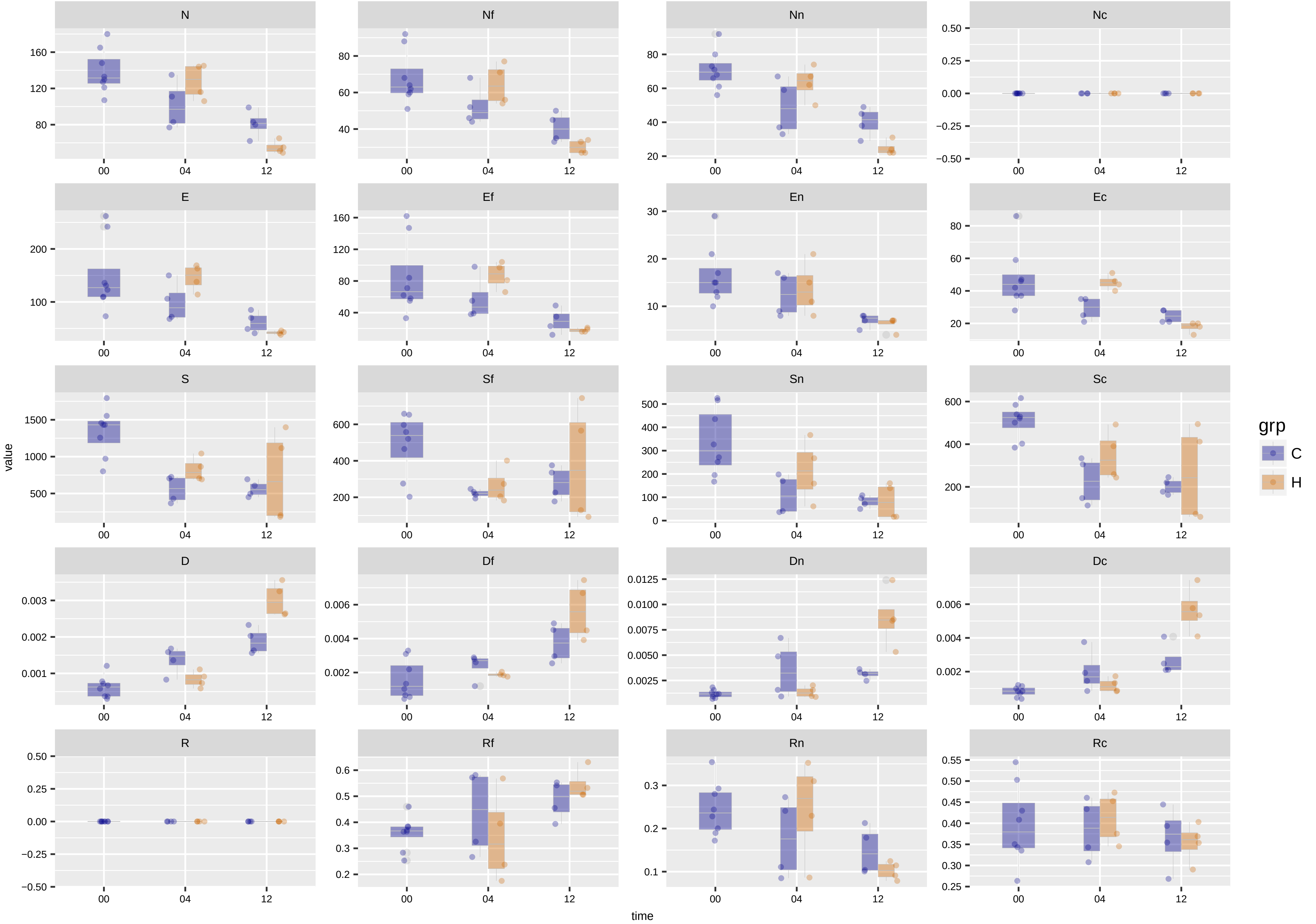

N
Nf
Nn
Nc
E
Ef
En
Ec
S
Sf
Sn
Sc
D
Df
Dn
Dc
R
Rf
Rn
Rc
value
time
00
04
12
grp
C
H

GO.0033036

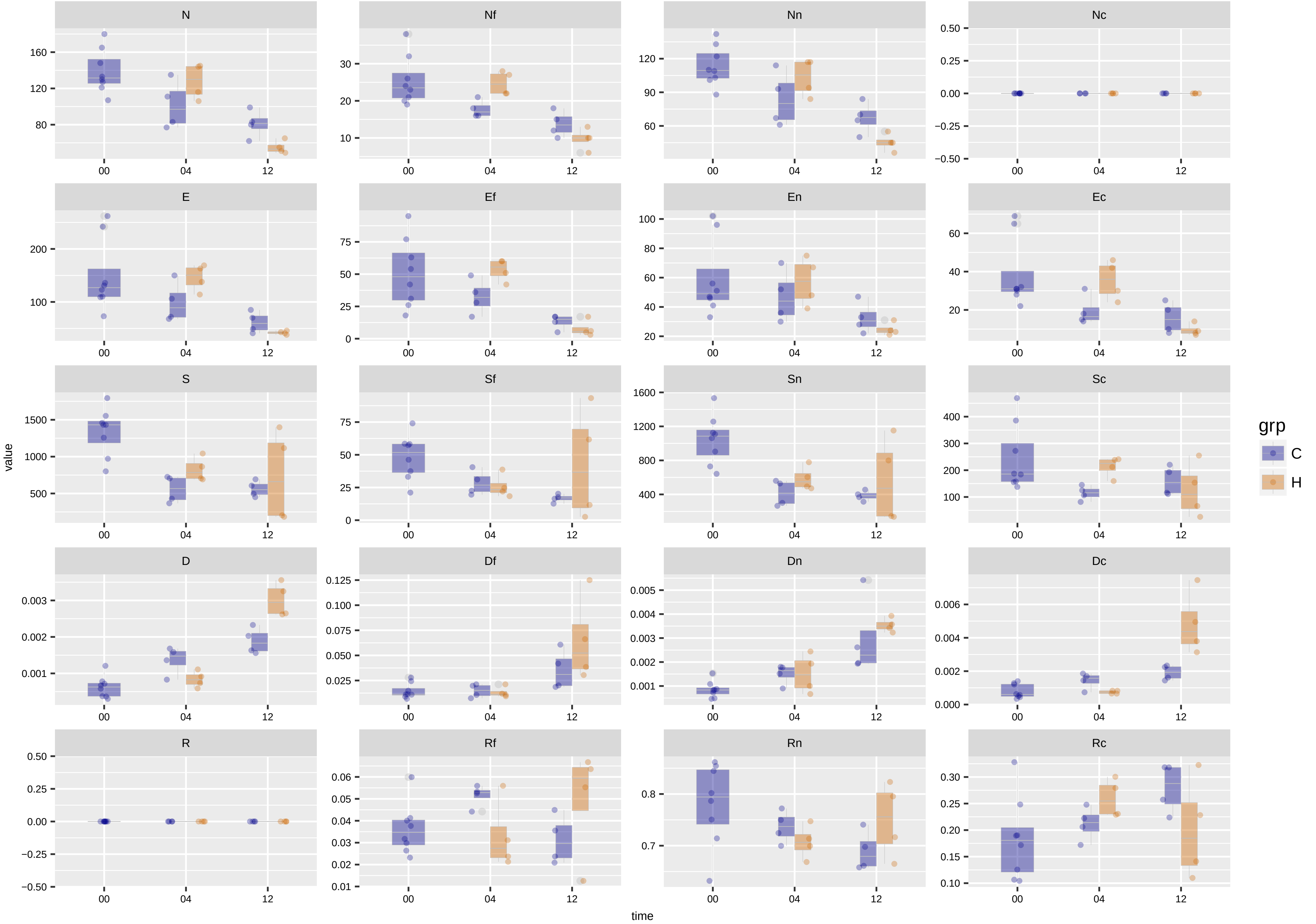

N
Nf
Nn
Nc
E
Ef
En
Ec
S
Sf
Sn
Sc
D
Df
Dn
Dc
R
Rf
Rn
Rc
value
time
00
04
12
grp
C
H

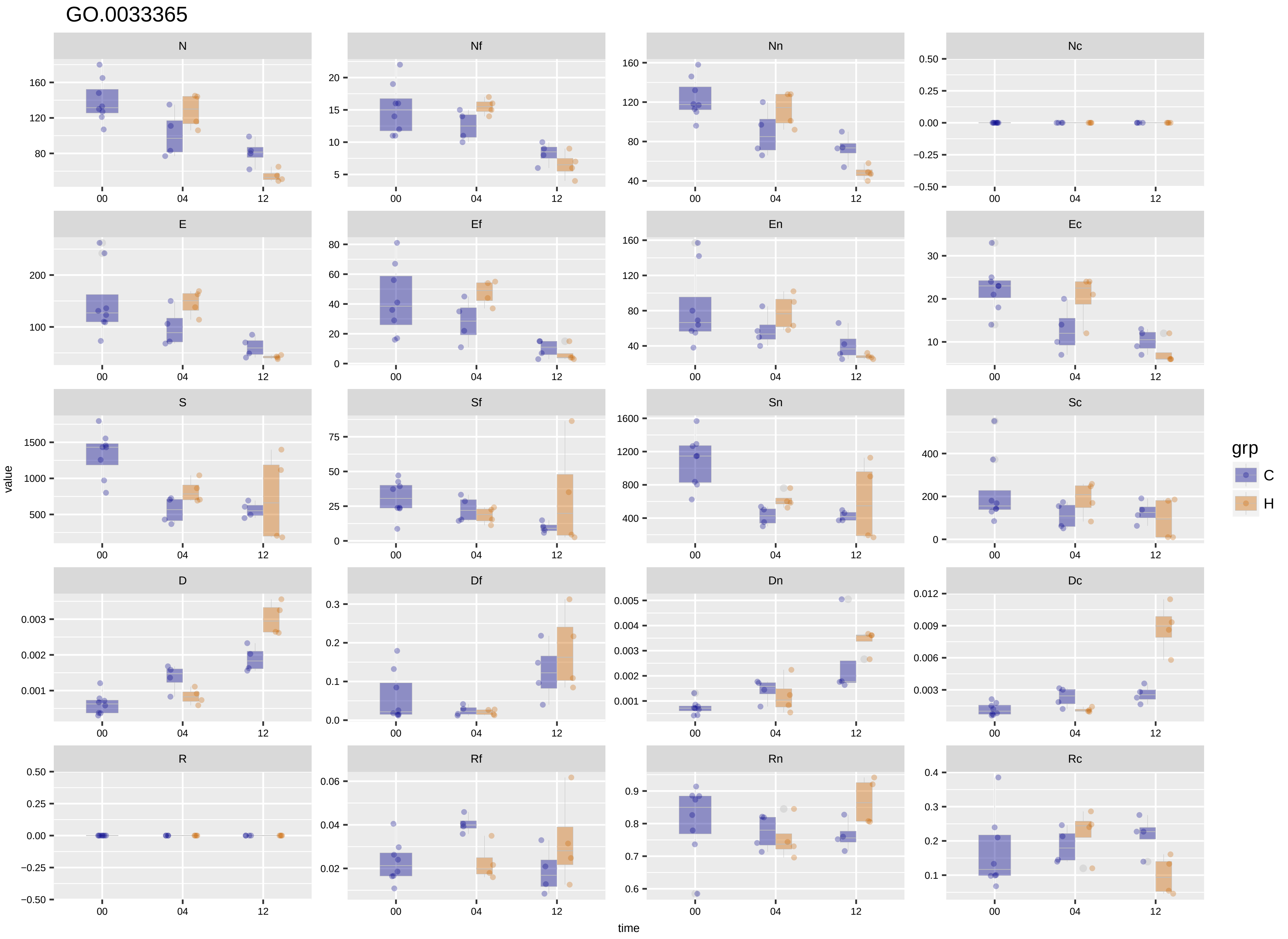
GO.0033365
N
Nf
Nn
Nc
E
Ef
En
Ec
S
Sf
Sn
Sc
D
Df
Dn
Dc
R
Rf
Rn
Rc
value
time
grp
C
H

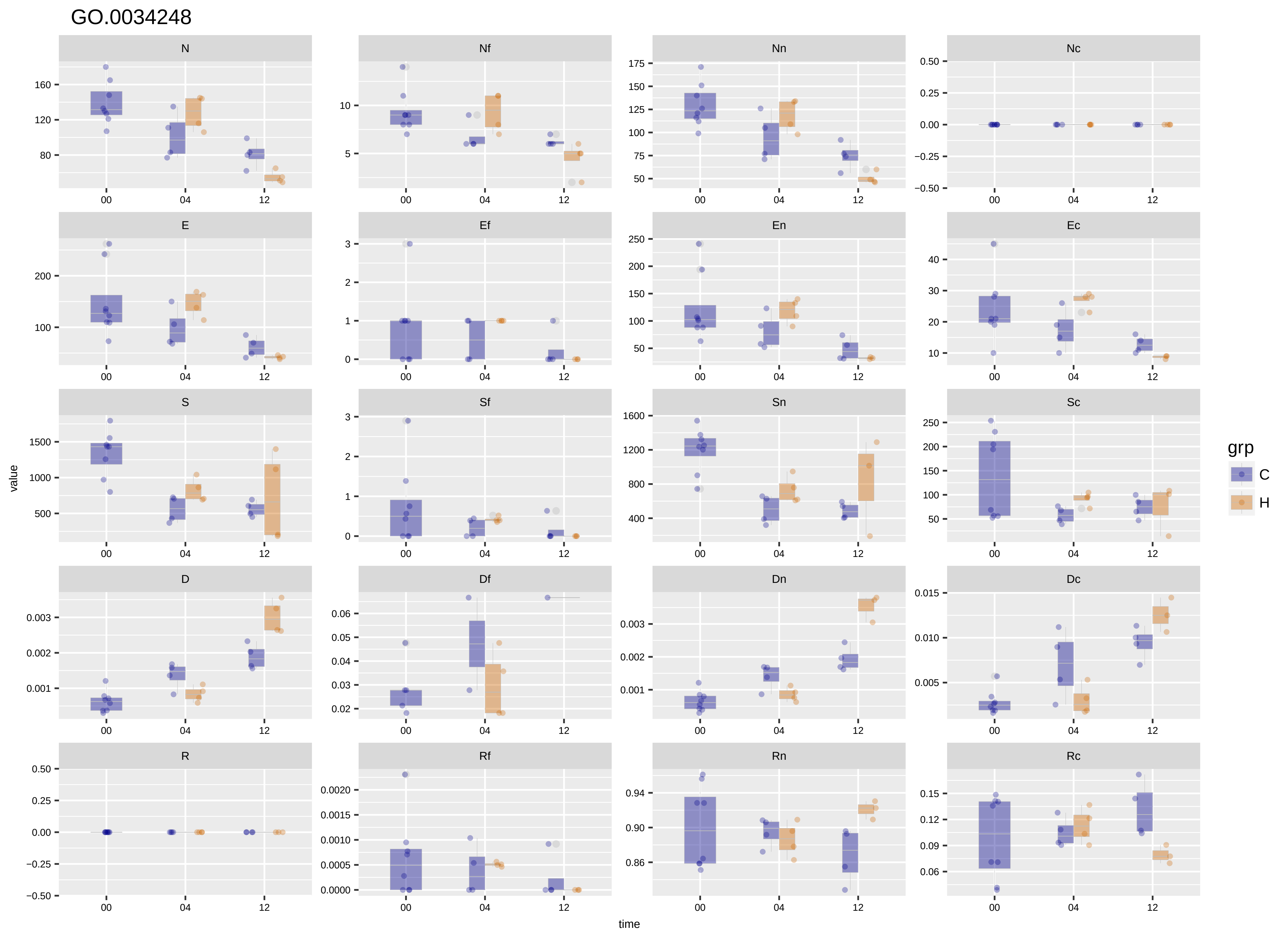

GO.0034248
N
Nf
Nn
Nc
E
Ef
En
Ec
S
Sf
Sn
Sc
D
Df
Dn
Dc
R
Rf
Rn
Rc
value
time
grp
C
H

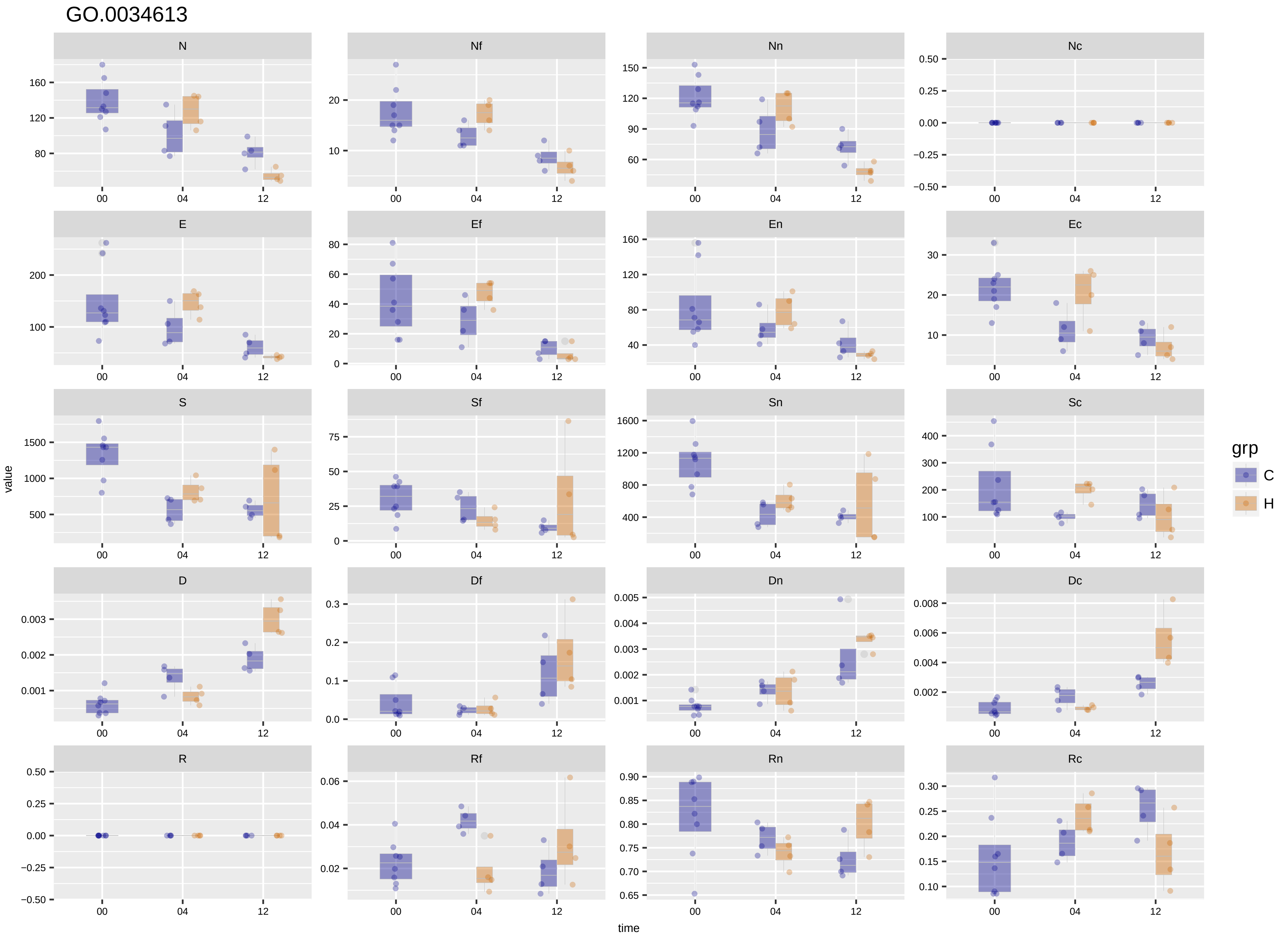

GO.0034613
N
Nf
Nn
Nc
E
Ef
En
Ec
S
Sf
Sn
Sc
D
Df
Dn
Dc
R
Rf
Rn
Rc
value
time
grp
C
H

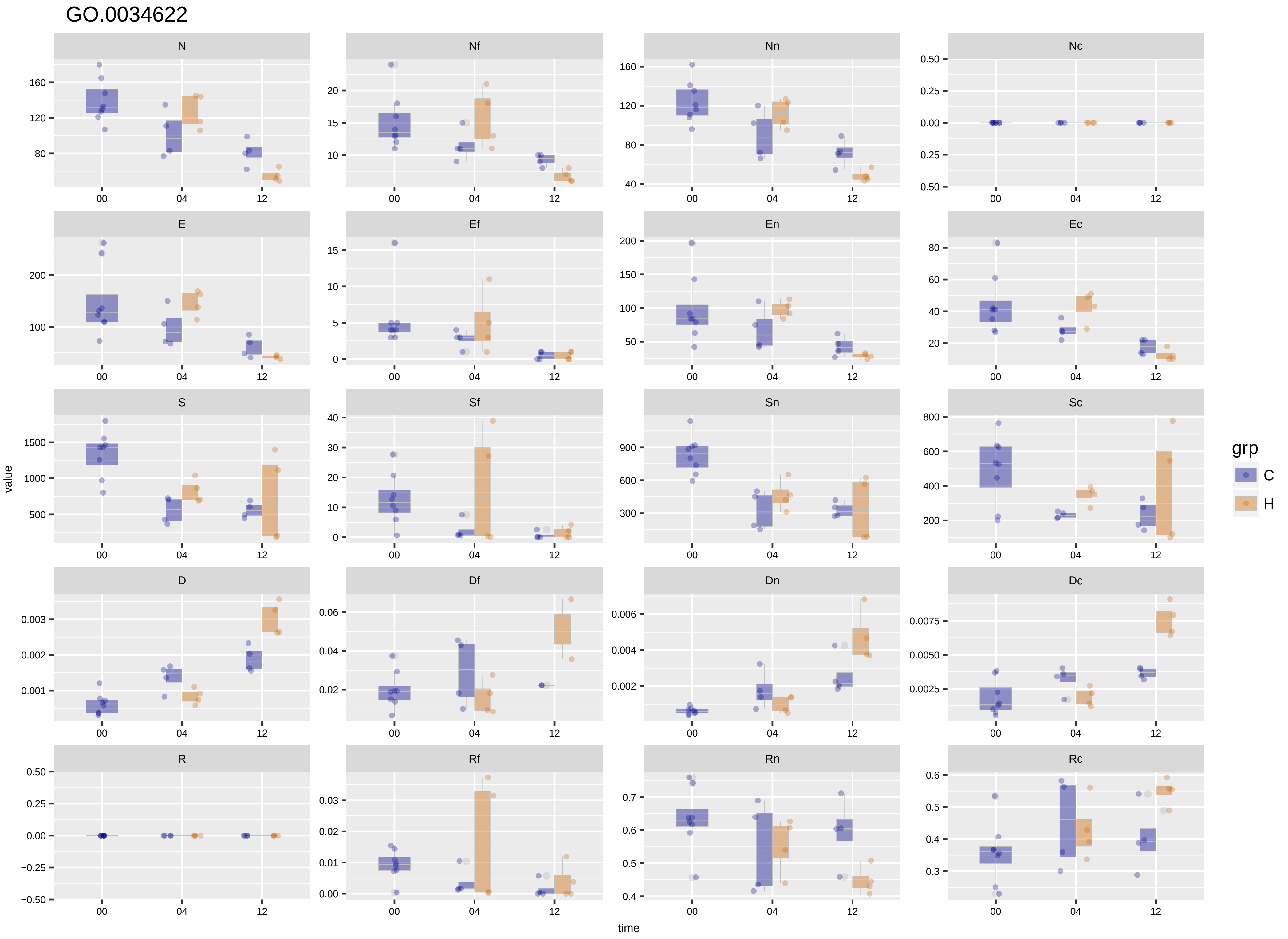

GO.0034622
N
Nf
Nn
Nc
E
Ef
En
Ec
S
Sf
Sn
Sc
D
Df
Dn
Dc
R
Rf
Rn
Rc
00
04
12
value
time
grp
C
H

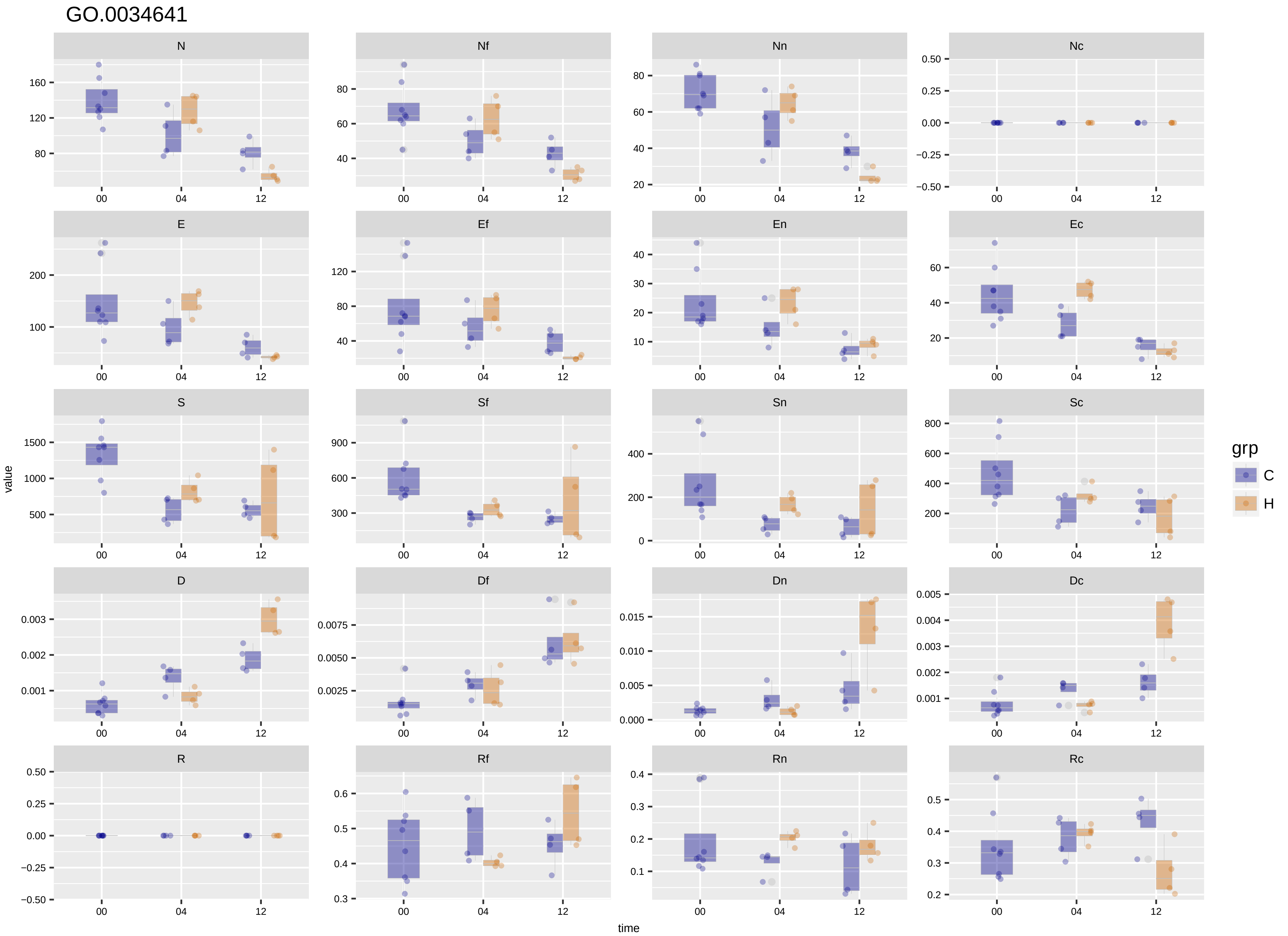

GO.0034641
N
Nf
Nn
Nc
E
Ef
En
Ec
S
Sf
Sn
Sc
D
Df
Dn
Dc
R
Rf
Rn
Rc
value
time
grp
C
H

GO.0034645

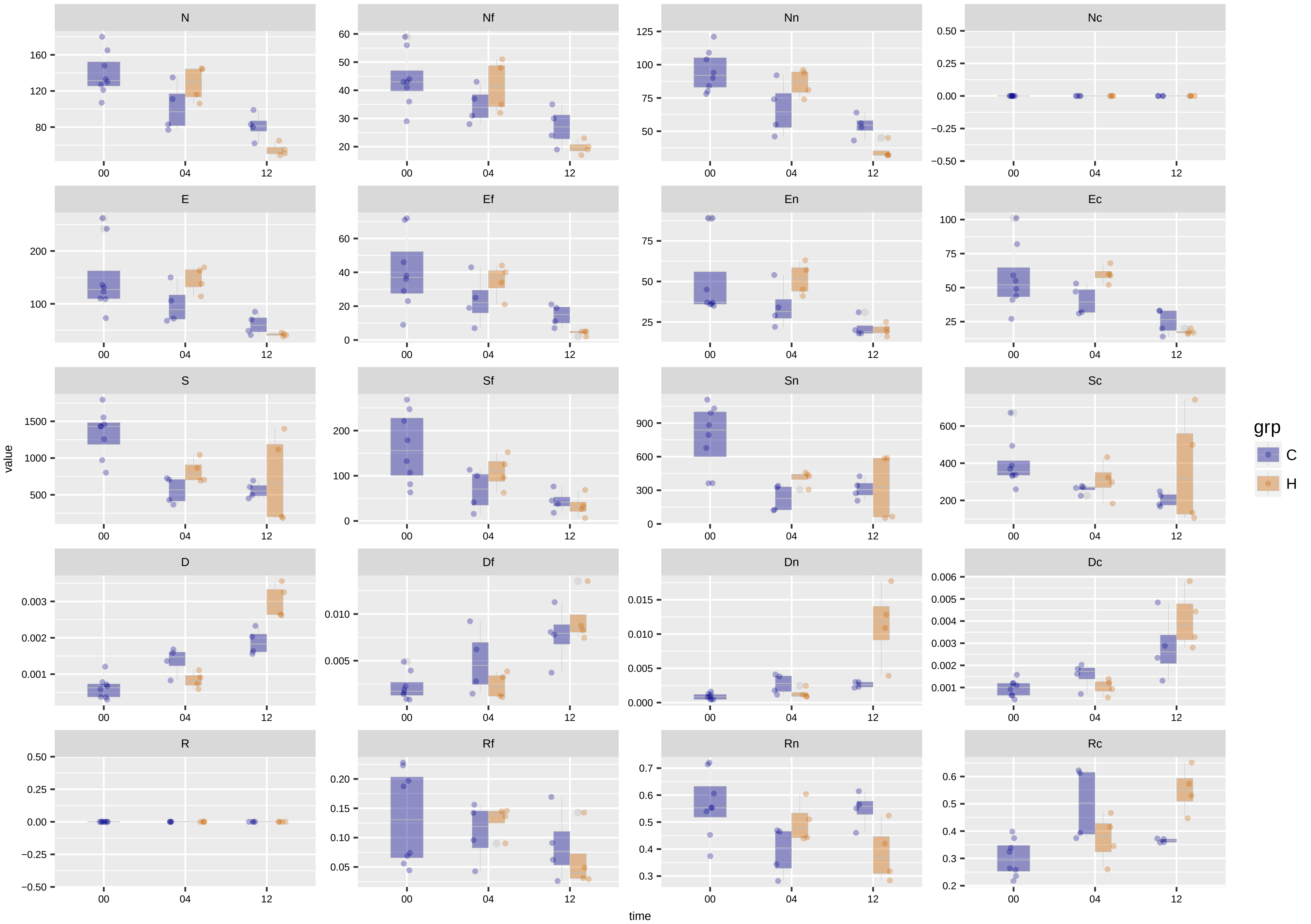

N
Nf
Nn
Nc
E
Ef
En
Ec
S
Sf
Sn
Sc
D
Df
Dn
Dc
R
Rf
Rn
Rc
00
04
12
value
time
grp
C
H

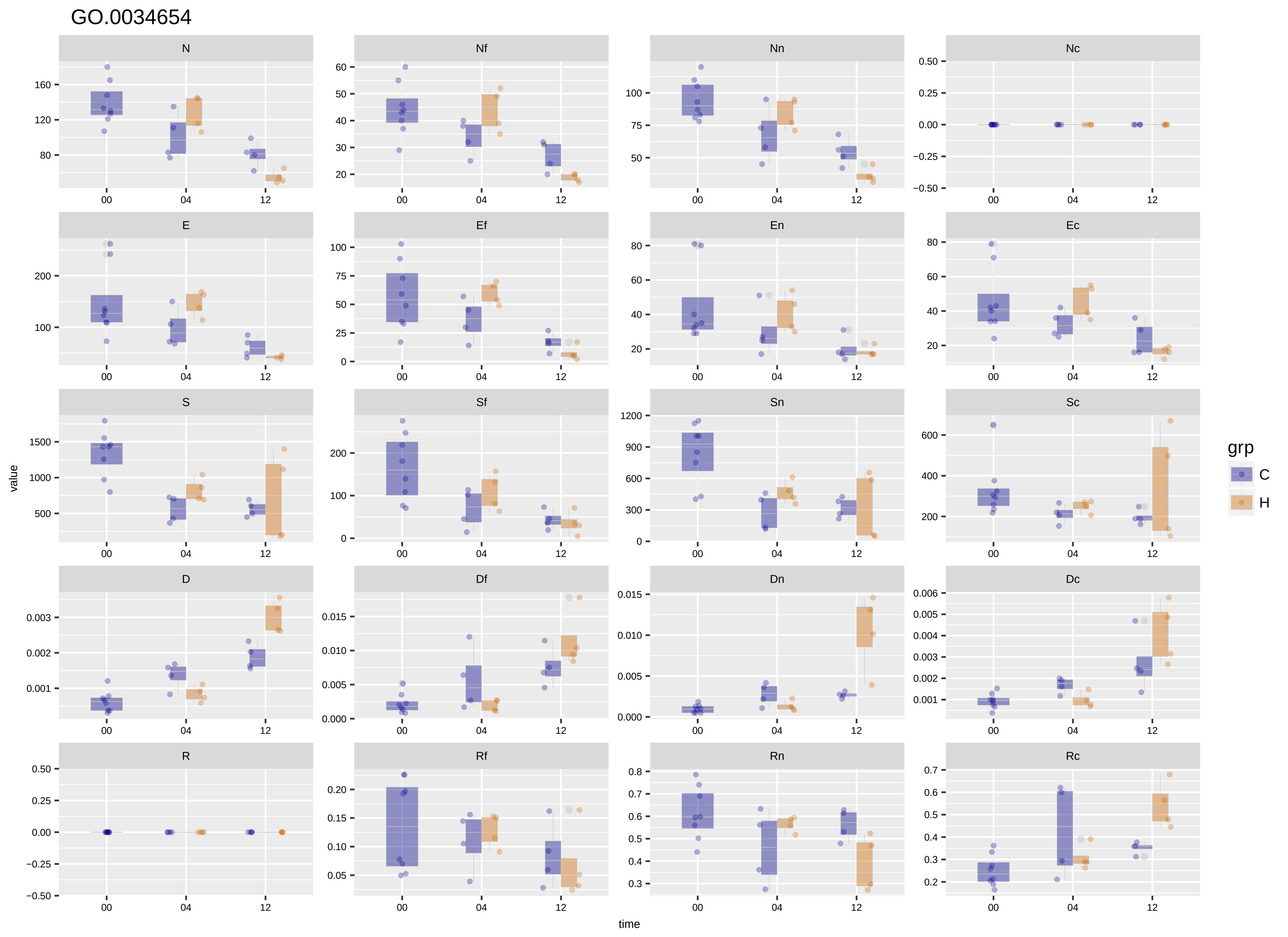
GO.0034654
N
Nf
Nn
Nc
E
Ef
En
Ec
S
Sf
Sn
Sc
D
Df
Dn
Dc
R
Rf
Rn
Rc
value
time
grp
C
H

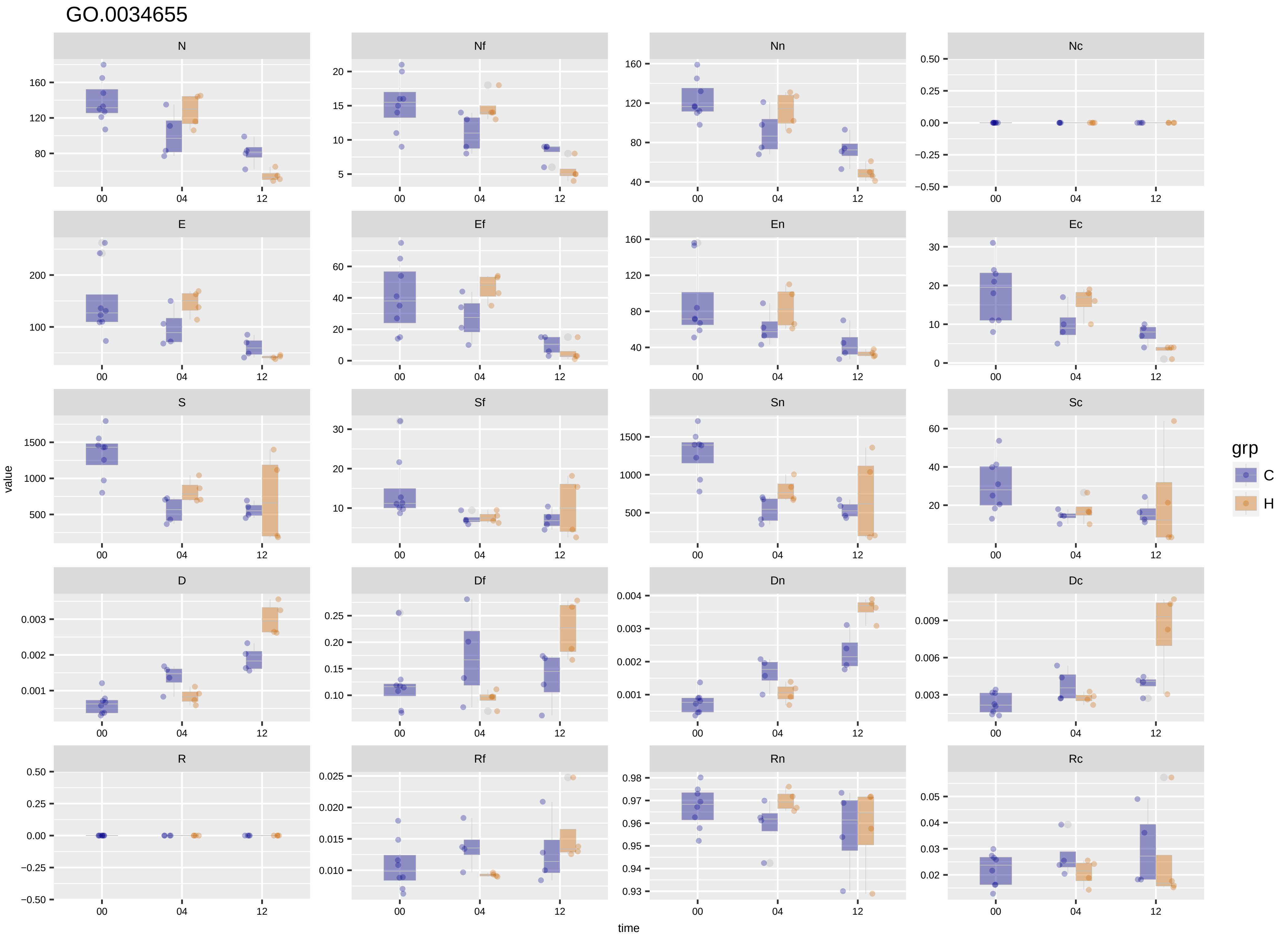

GO.0034655
N
Nf
Nn
Nc
E
Ef
En
Ec
S
Sf
Sn
Sc
D
Df
Dn
Dc
R
Rf
Rn
Rc
value
time
grp
C
H

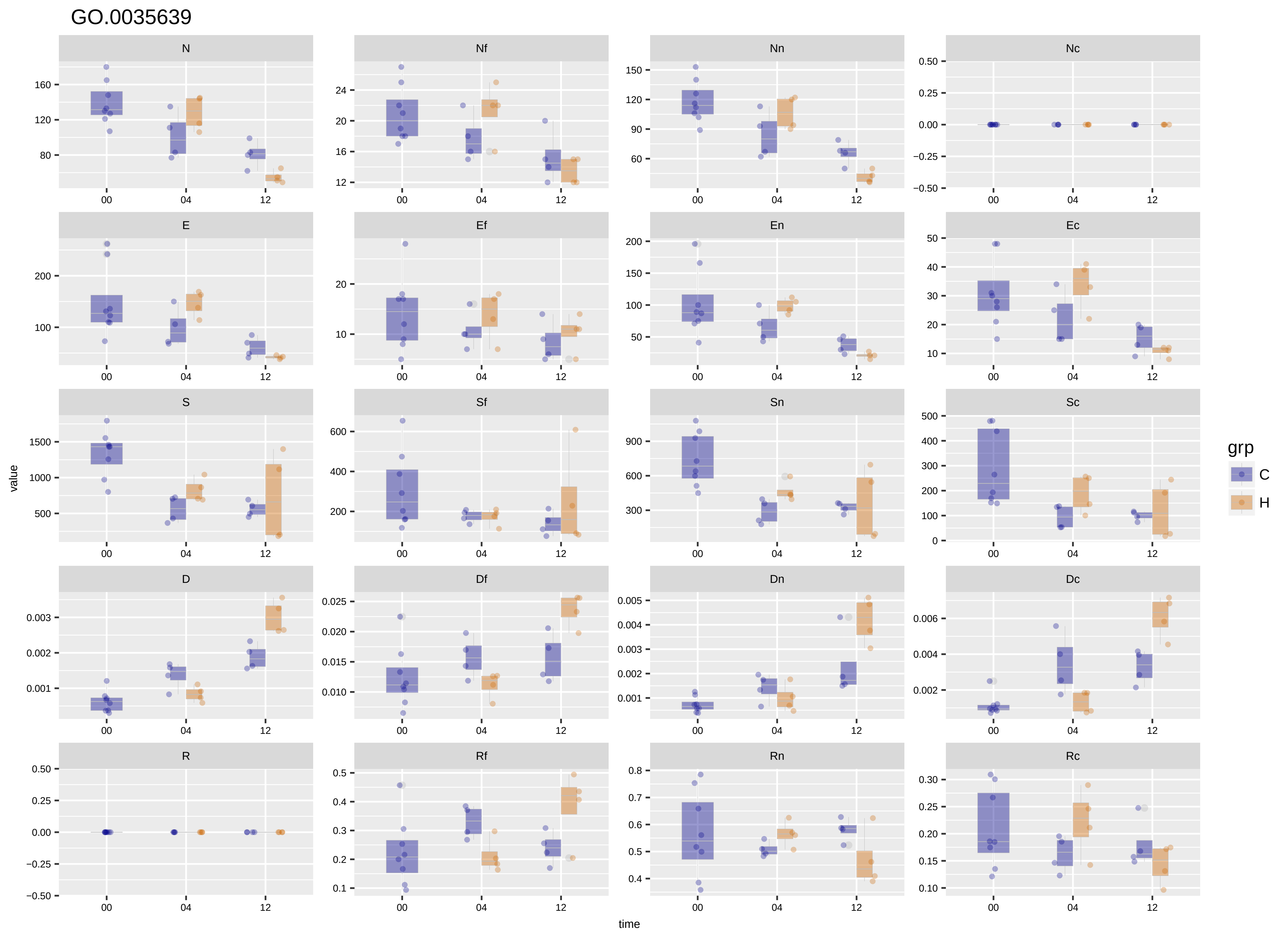
GO.0035639
N
Nf
Nn
Nc
E
Ef
En
Ec
S
Sf
Sn
Sc
D
Df
Dn
Dc
R
Rf
Rn
Rc
value
time
00
04
12
grp
C
H

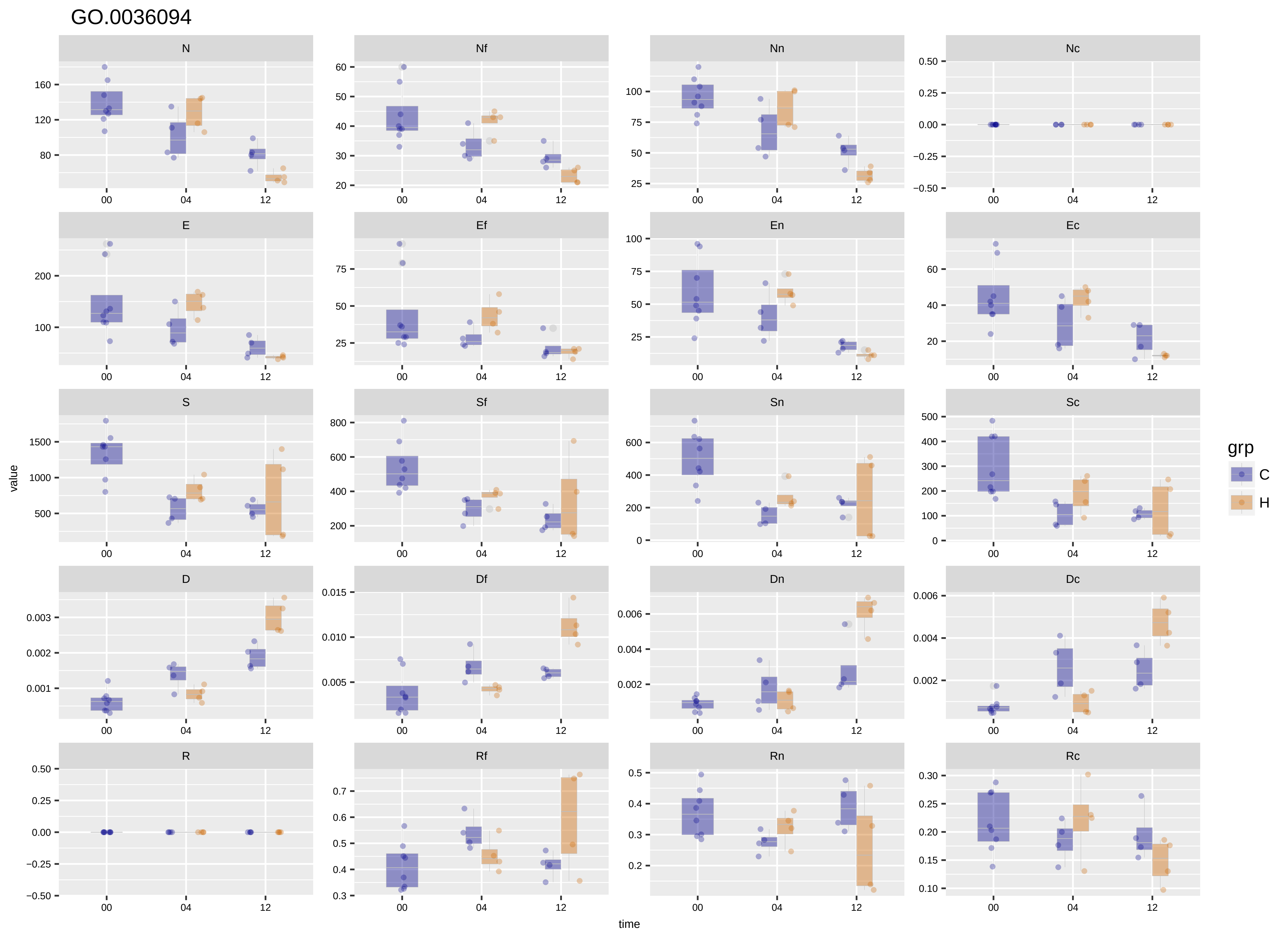
GO.0036094
N
Nf
Nn
Nc
E
Ef
En
Ec
S
Sf
Sn
Sc
D
Df
Dn
Dc
R
Rf
Rn
Rc
value
time
grp
C
H

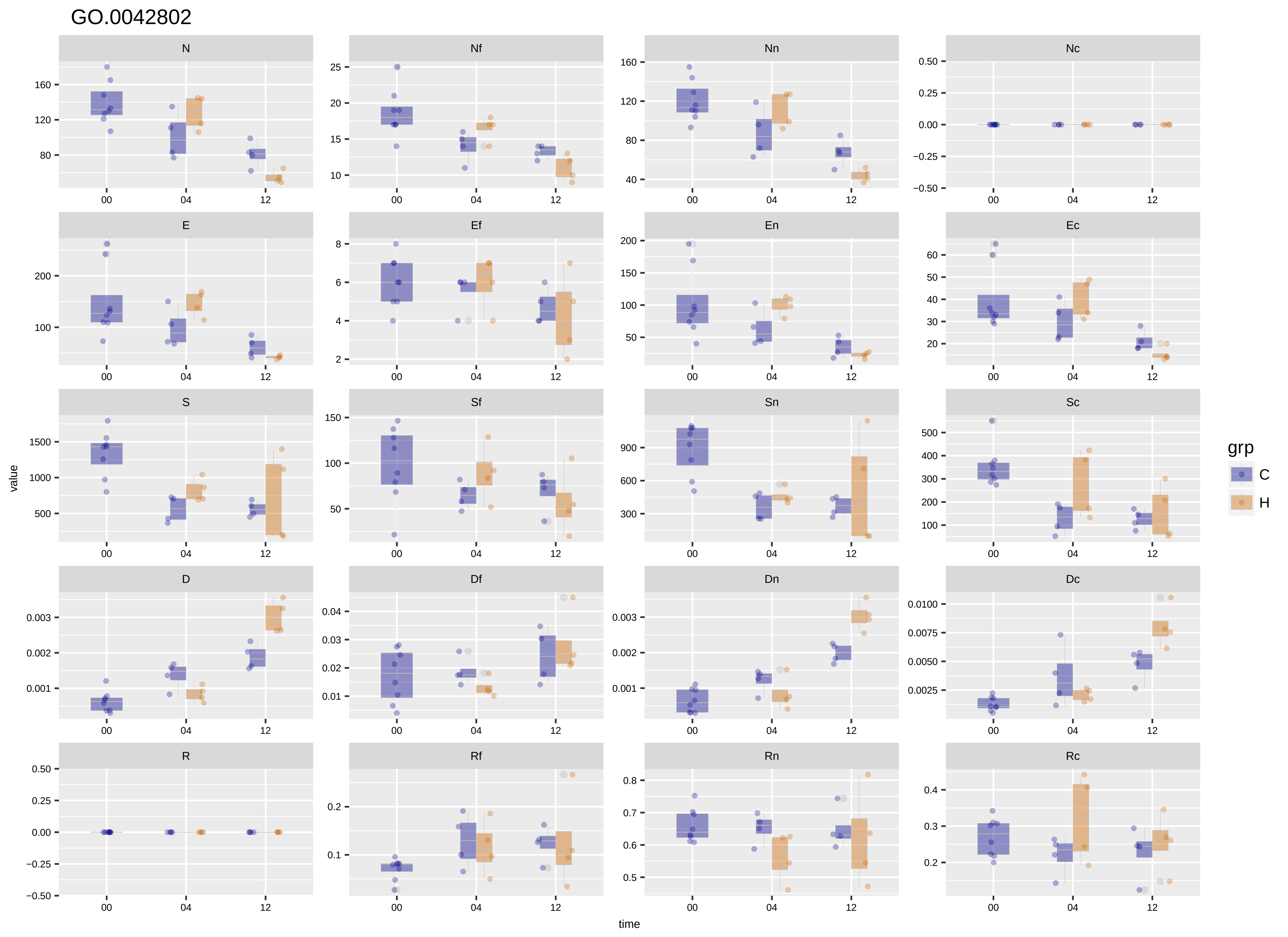
GO.0042802
N
Nf
Nn
Nc
E
Ef
En
Ec
S
Sf
Sn
Sc
D
Df
Dn
Dc
R
Rf
Rn
Rc
value
time
grp
C
H

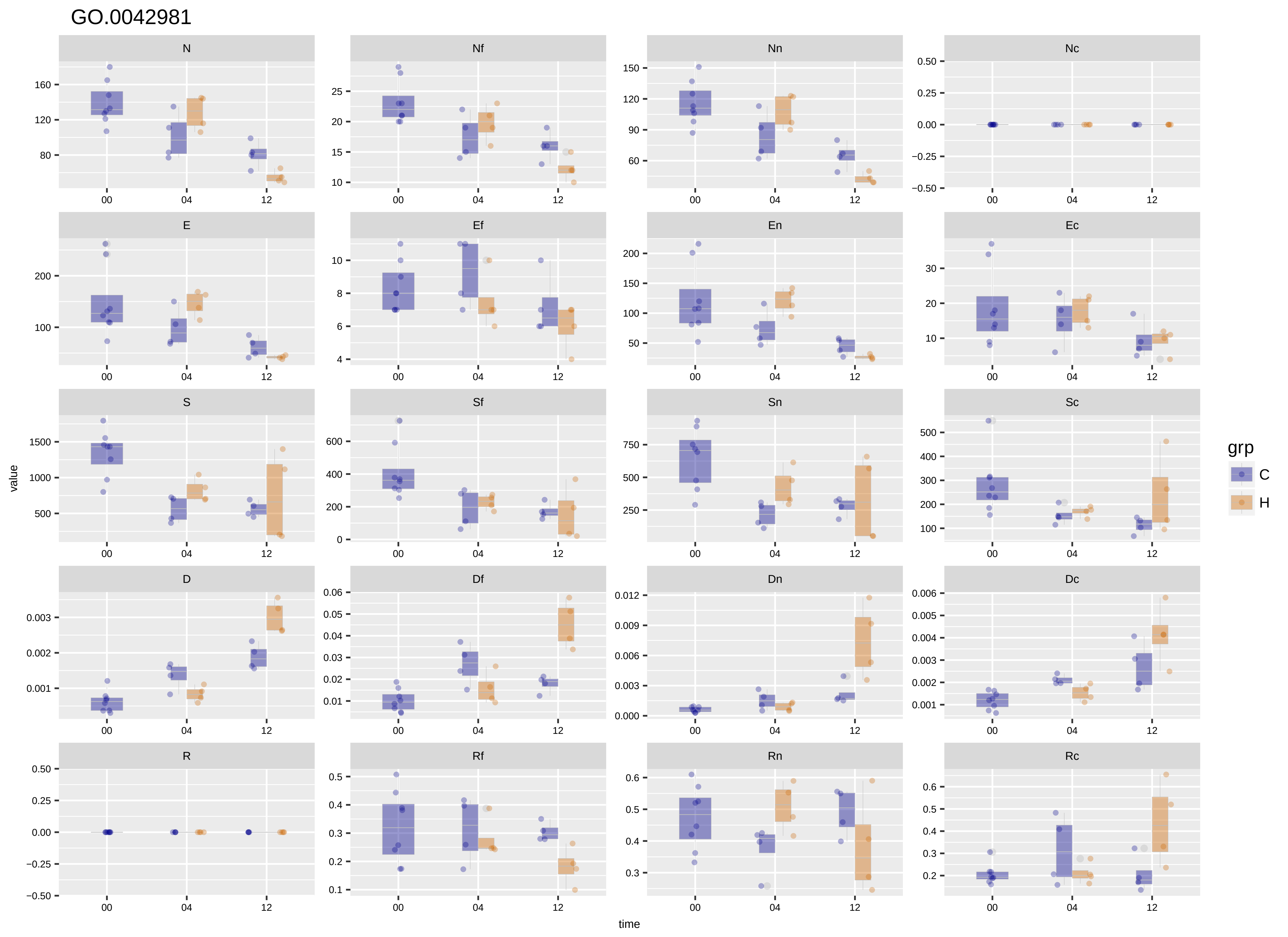

GO.0042981
N
Nf
Nn
Nc
E
Ef
En
Ec
S
Sf
Sn
Sc
D
Df
Dn
Dc
R
Rf
Rn
Rc
00
04
12
value
time
grp
C
H

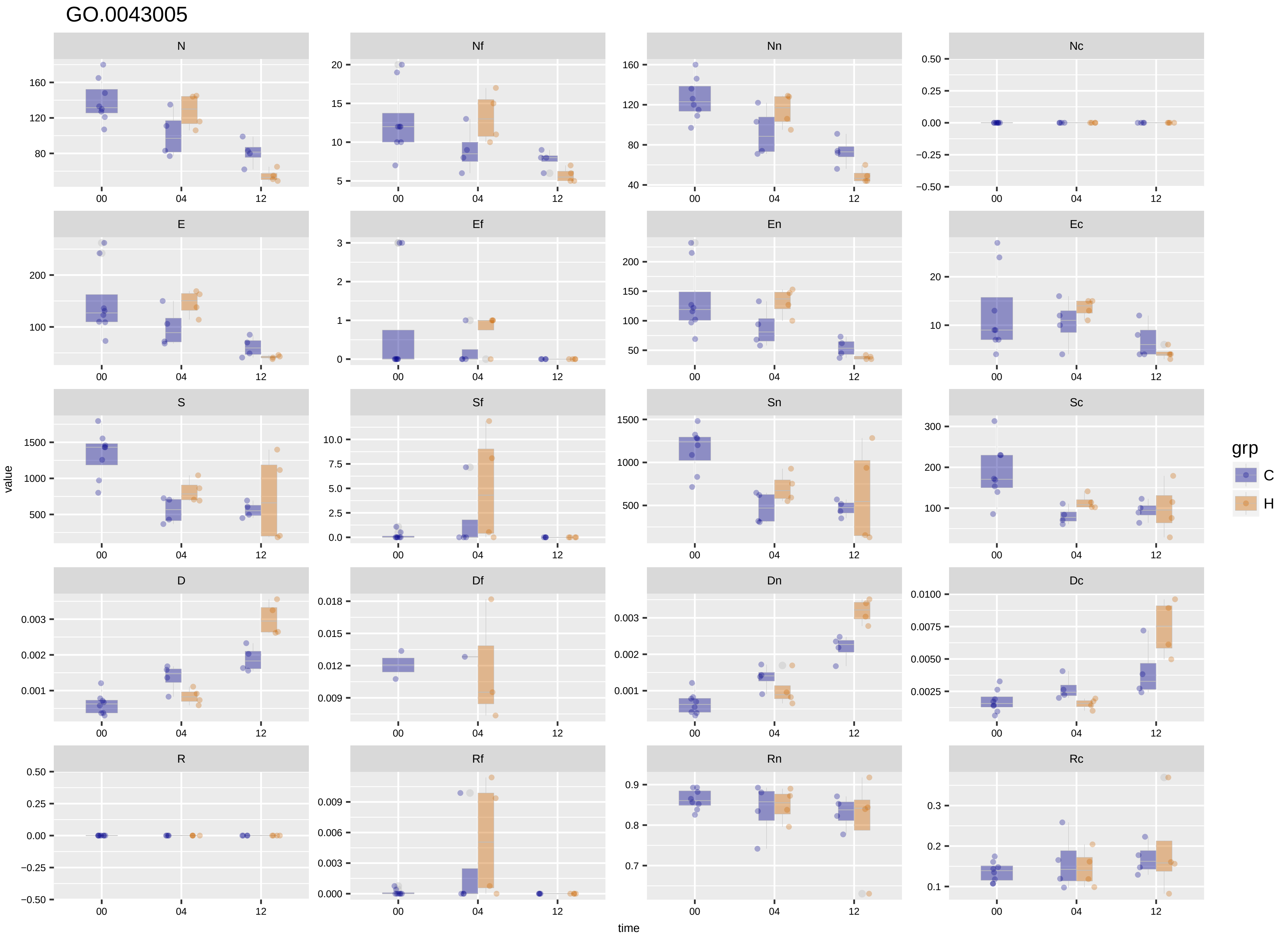
GO.0043005
N
Nf
Nn
Nc
E
Ef
En
Ec
S
Sf
Sn
Sc
D
Df
Dn
Dc
R
Rf
Rn
Rc
value
time
grp
C
H

GO.0043065

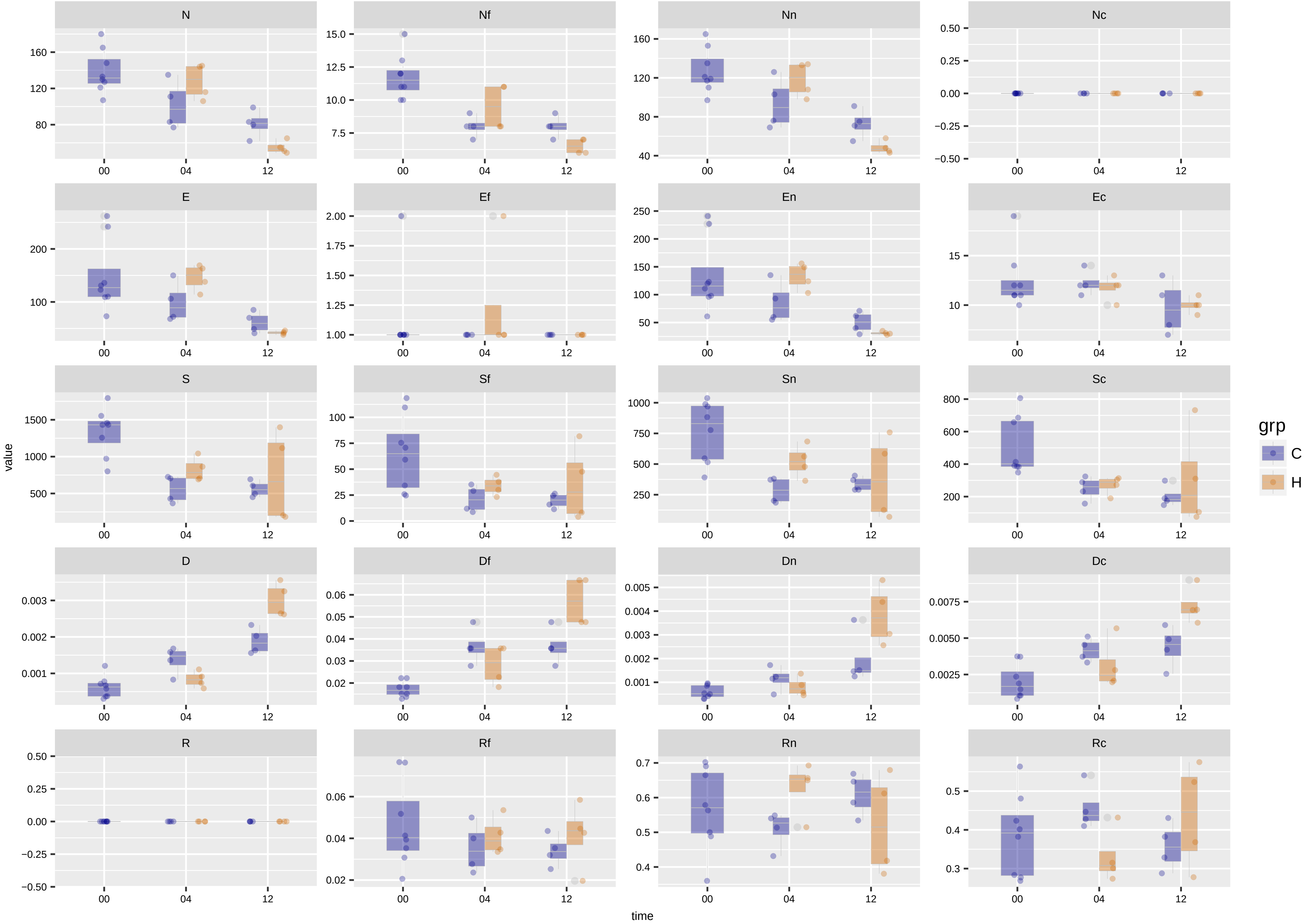

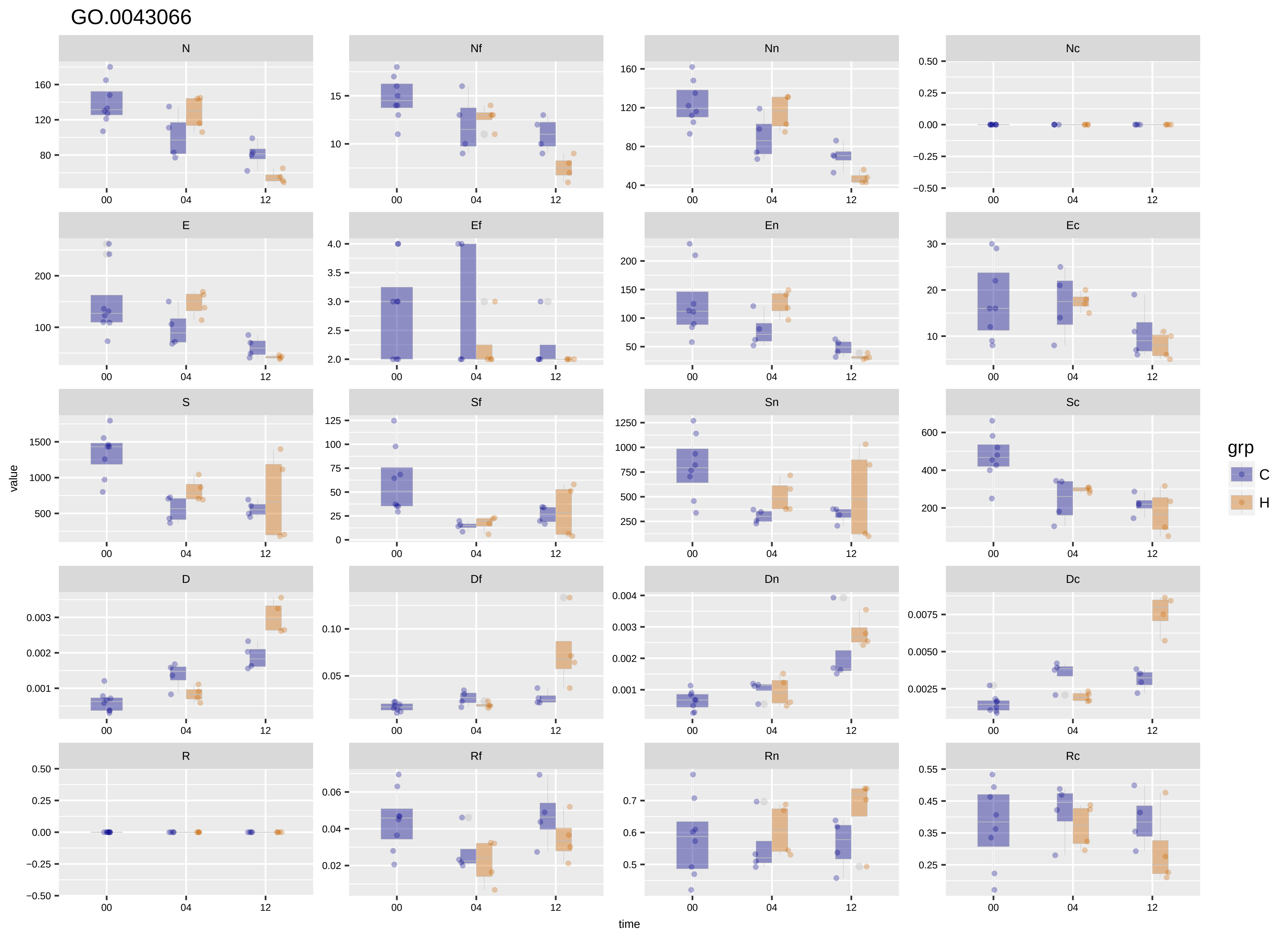
GO.0043066
N
Nf
Nn
Nc
E
Ef
En
Ec
S
Sf
Sn
Sc
D
Df
Dn
Dc
R
Rf
Rn
Rc
value
time
grp
C
H

GO.0043209

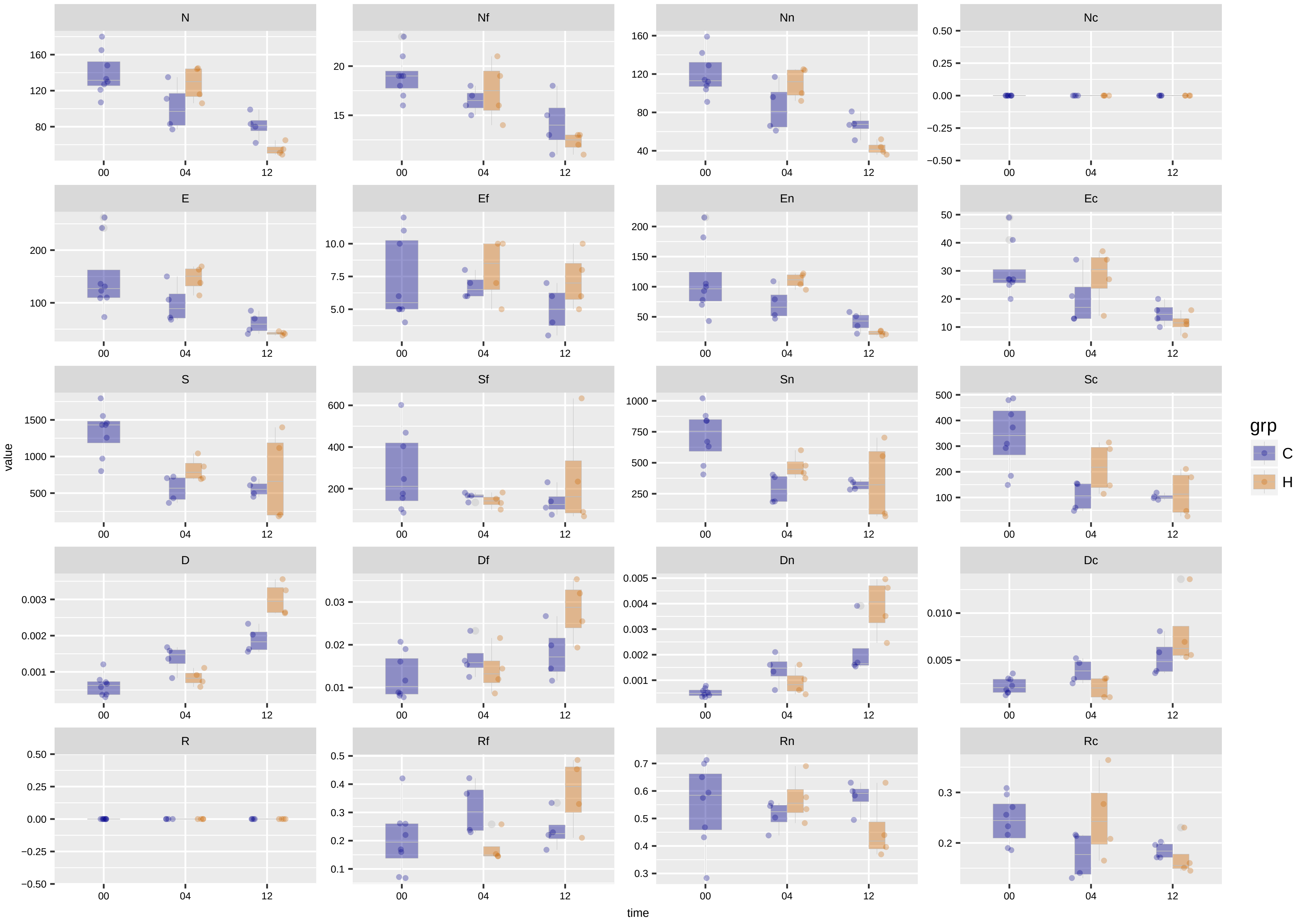

N
Nf
Nn
Nc
E
Ef
En
Ec
S
Sf
Sn
Sc
D
Df
Dn
Dc
R
Rf
Rn
Rc
00
04
12
value
time
grp
C
H

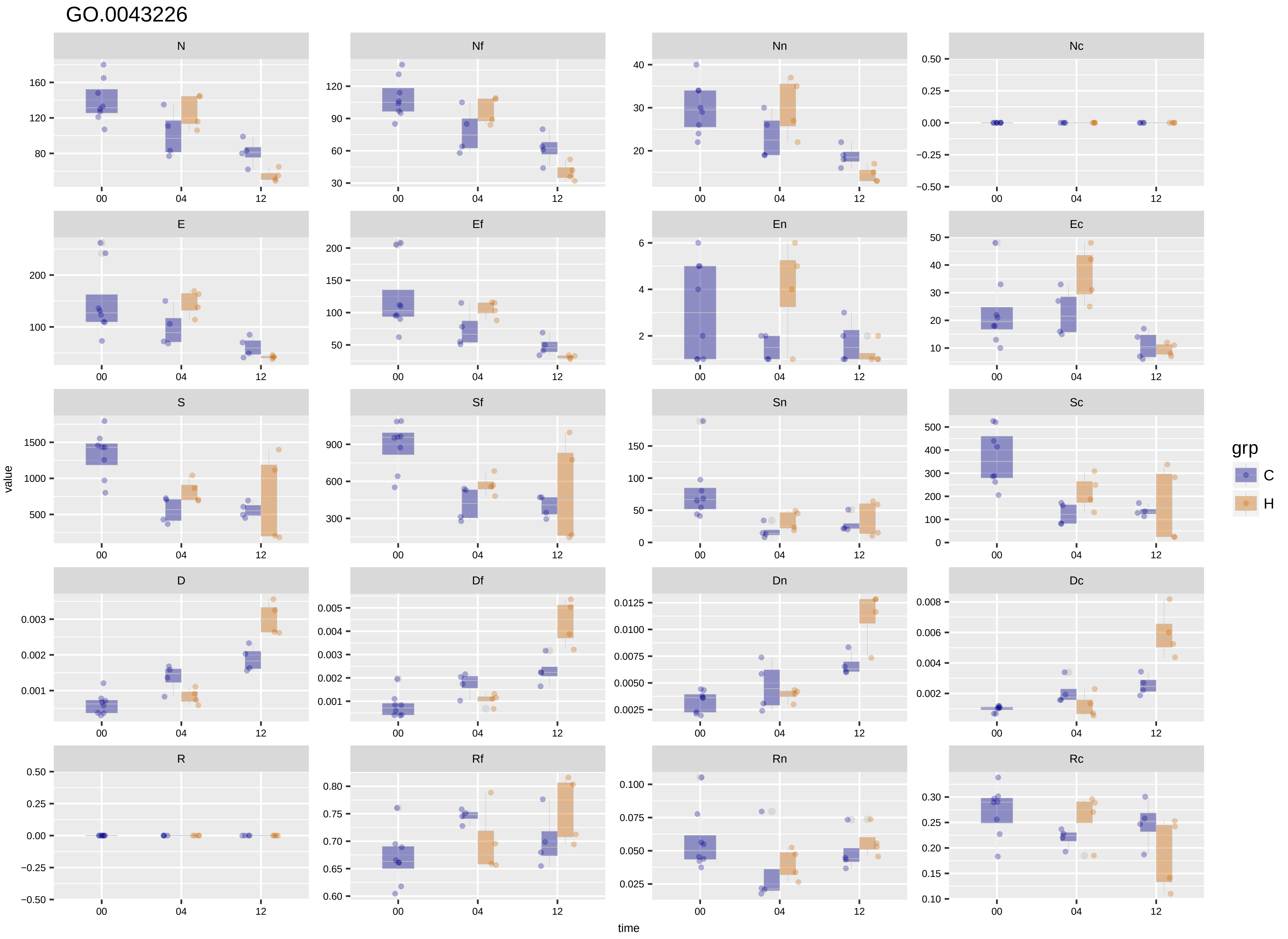

GO.0043226
N
Nf
Nn
Nc
E
Ef
En
Ec
S
Sf
Sn
Sc
D
Df
Dn
Dc
R
Rf
Rn
Rc
value
time
grp
C
H
00
04
12

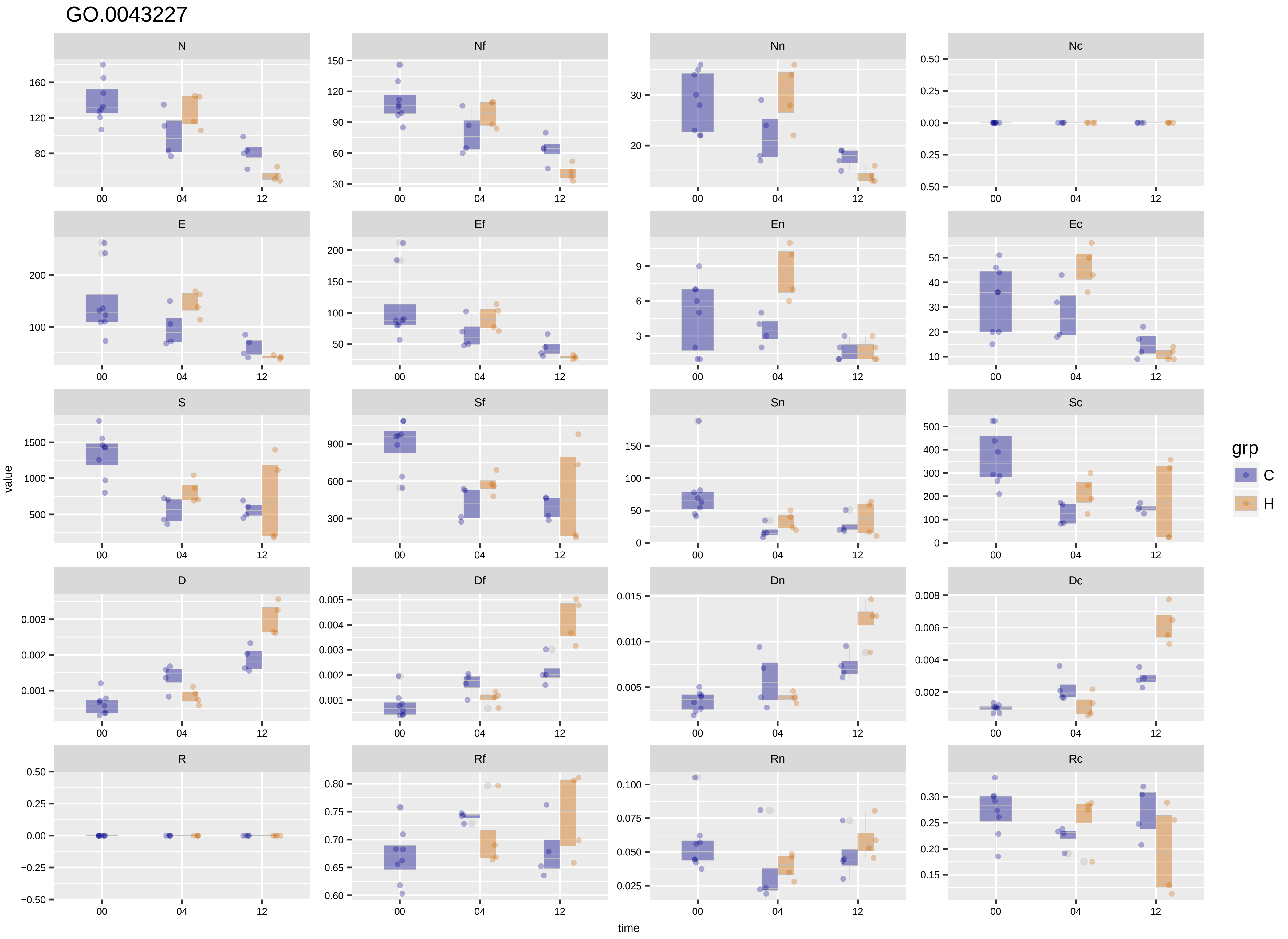

GO.0043227
N
Nf
Nn
Nc
E
Ef
En
Ec
S
Sf
Sn
Sc
D
Df
Dn
Dc
R
Rf
Rn
Rc
value
time
00
04
12
grp
C
H

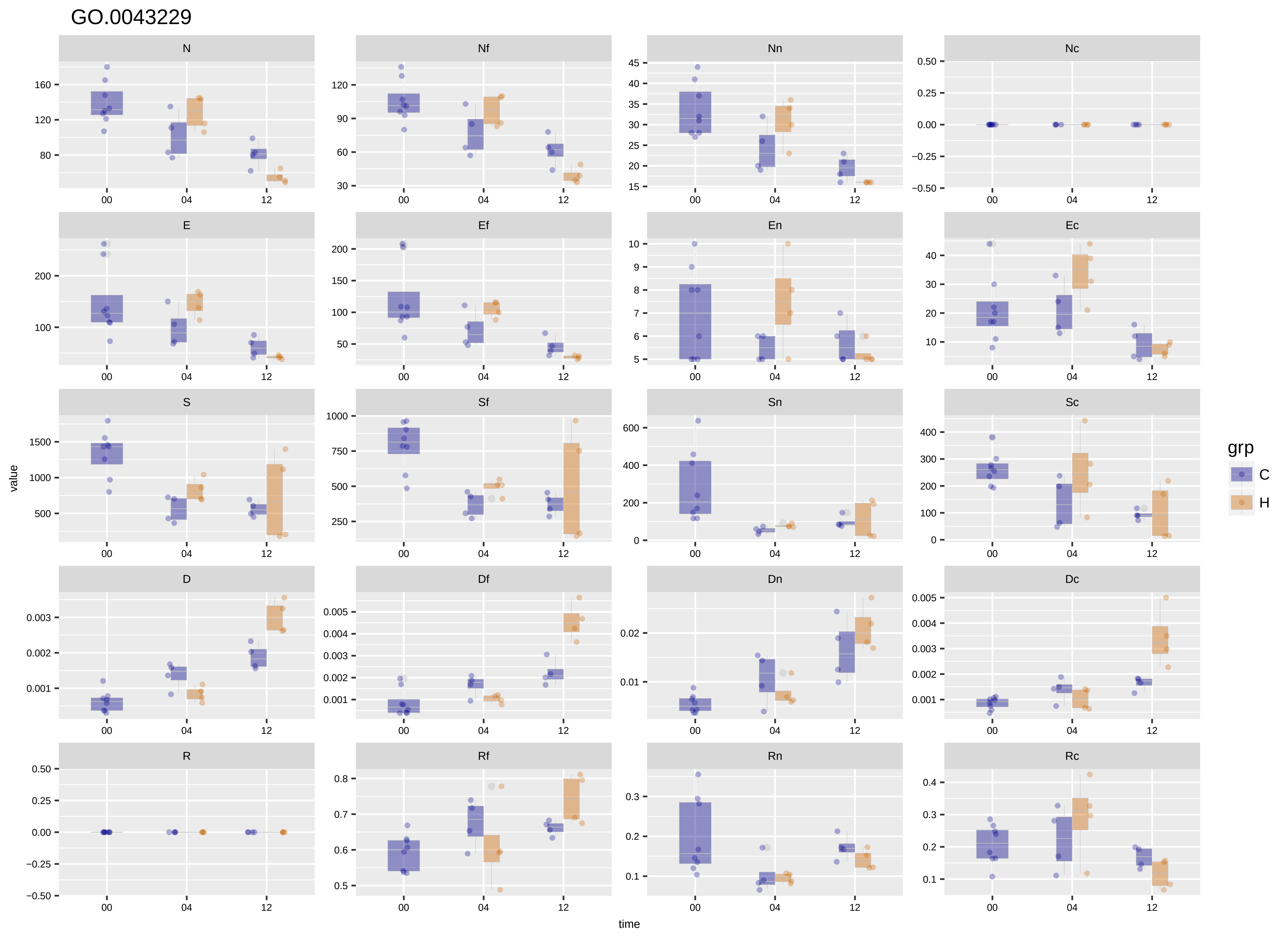

GO.0043229
N
Nf
Nn
Nc
E
Ef
En
Ec
S
Sf
Sn
Sc
D
Df
Dn
Dc
R
Rf
Rn
Rc
value
time
grp
C
H

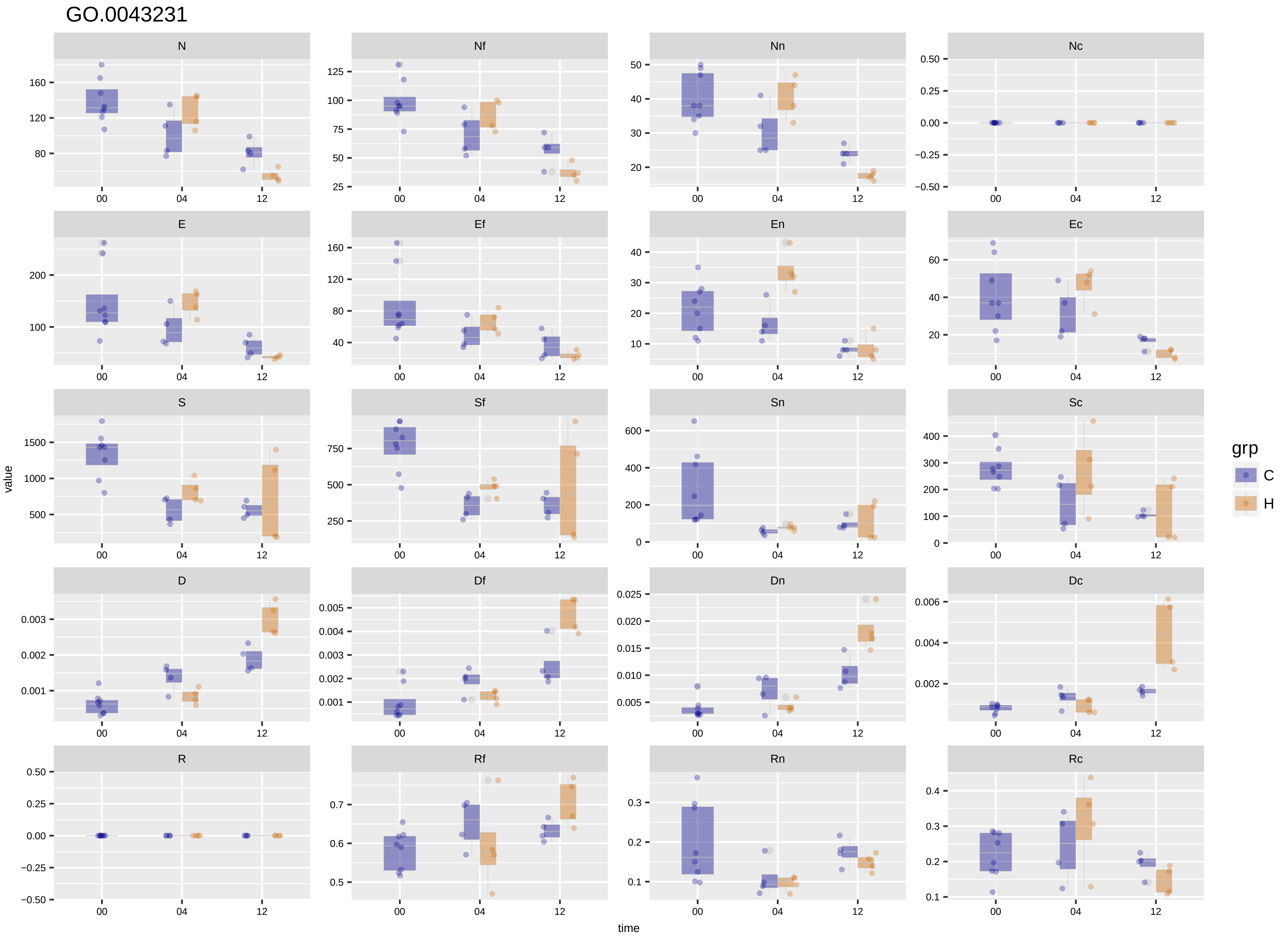
GO.0043231
N
Nf
Nn
Nc
E
Ef
En
Ec
S
Sf
Sn
Sc
D
Df
Dn
Dc
R
Rf
Rn
Rc
value
time
grp
C
H

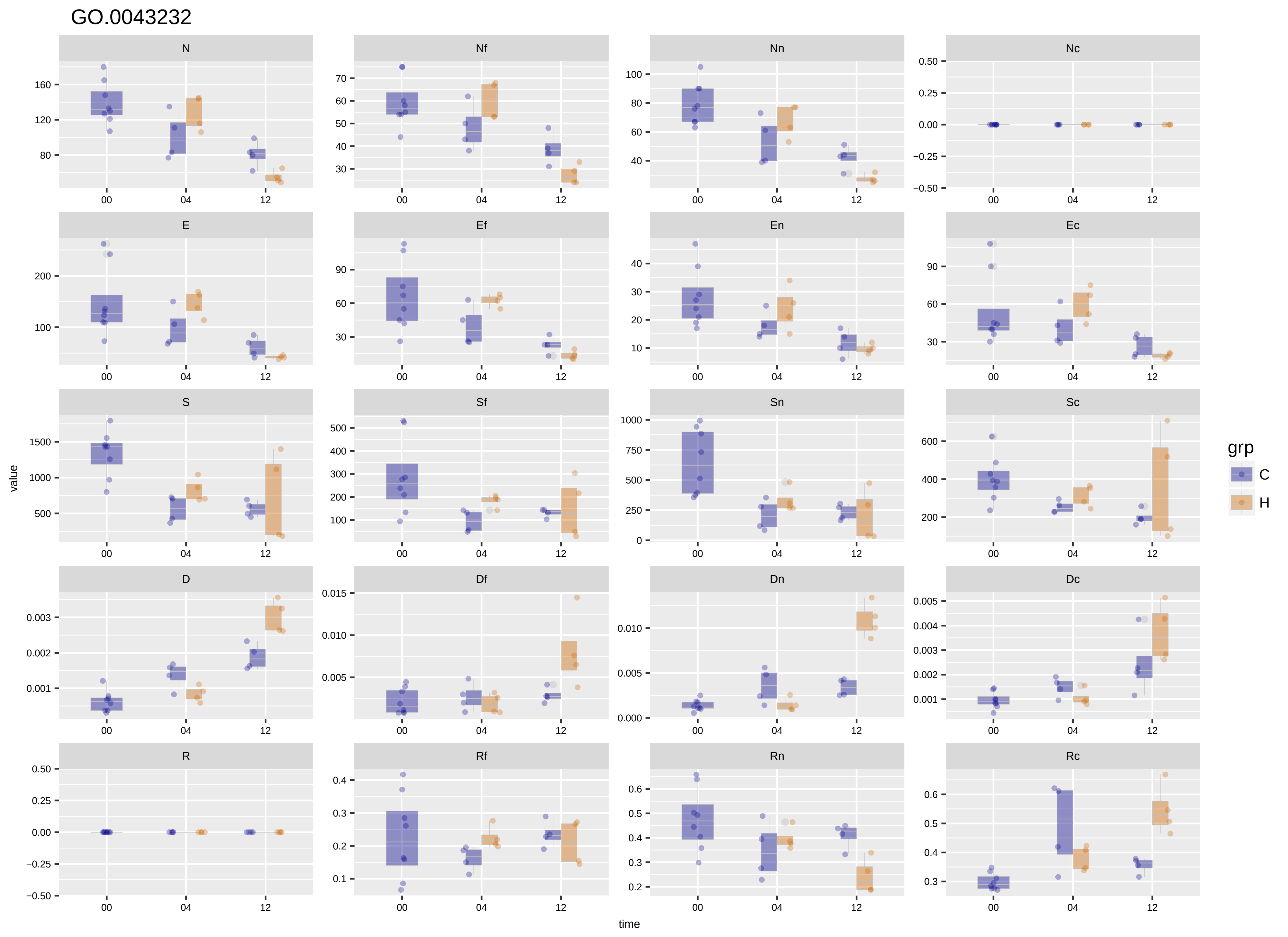
GO.0043232
N
Nf
Nn
Nc
E
Ef
En
Ec
S
Sf
Sn
Sc
D
Df
Dn
Dc
R
Rf
Rn
Rc
00
04
12
value
time
grp
C
H

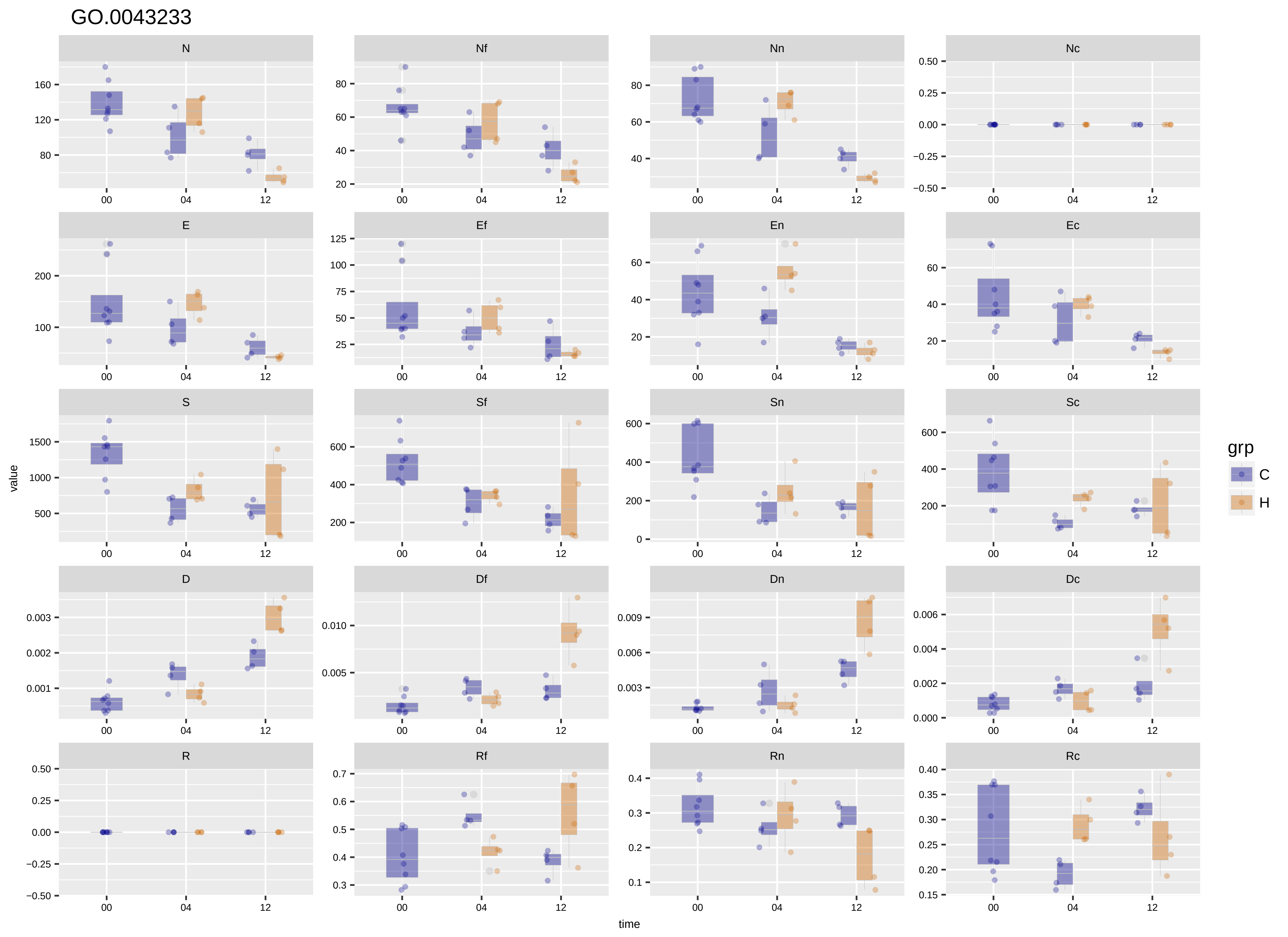

GO.0043233
N
Nf
Nn
Nc
E
Ef
En
Ec
S
Sf
Sn
Sc
D
Df
Dn
Dc
R
Rf
Rn
Rc
value
time
grp
C
H

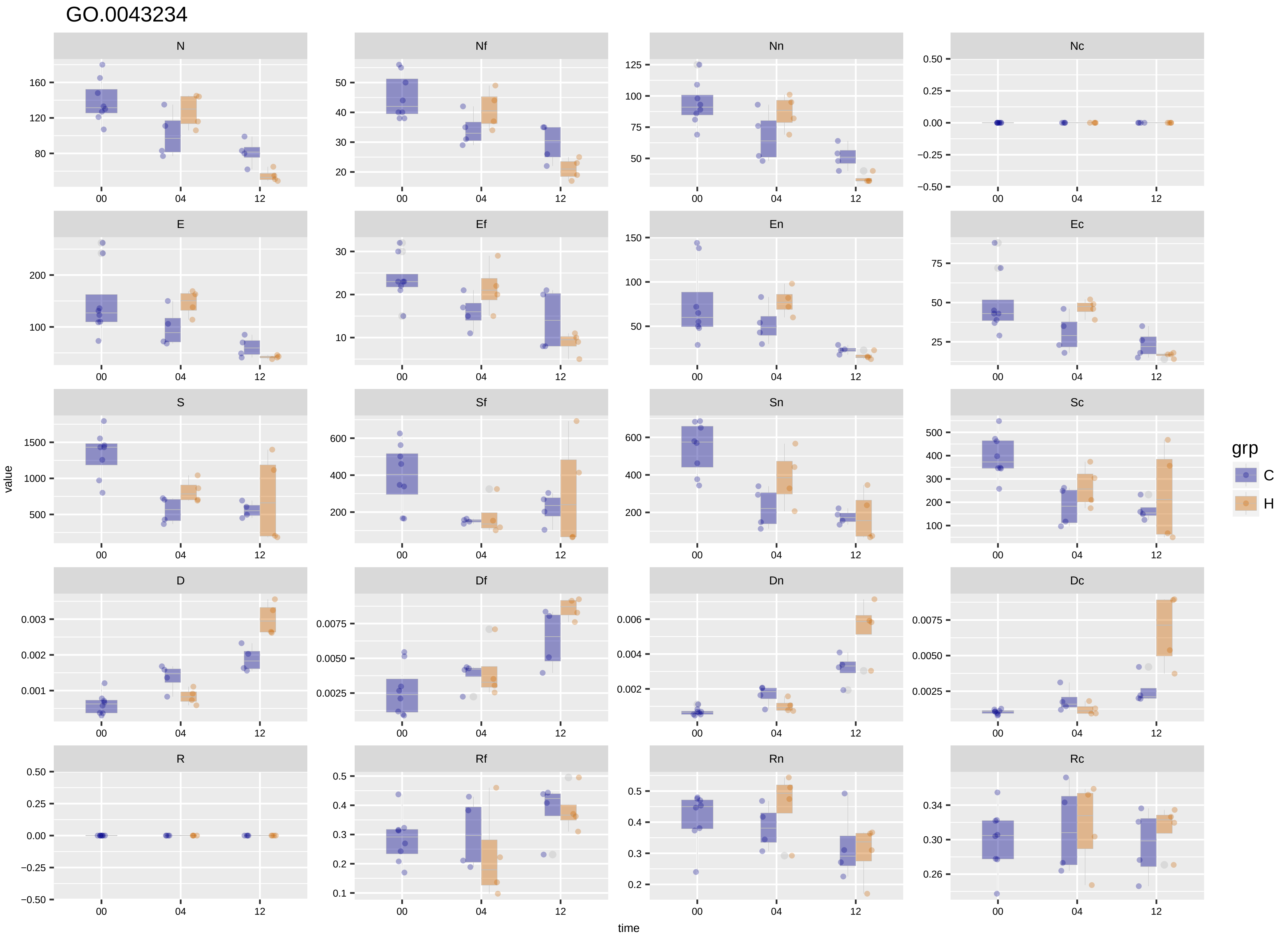

GO.0043234
N
Nf
Nn
Nc
E
Ef
En
Ec
S
Sf
Sn
Sc
D
Df
Dn
Dc
R
Rf
Rn
Rc
value
time
00
04
12
grp
C
H

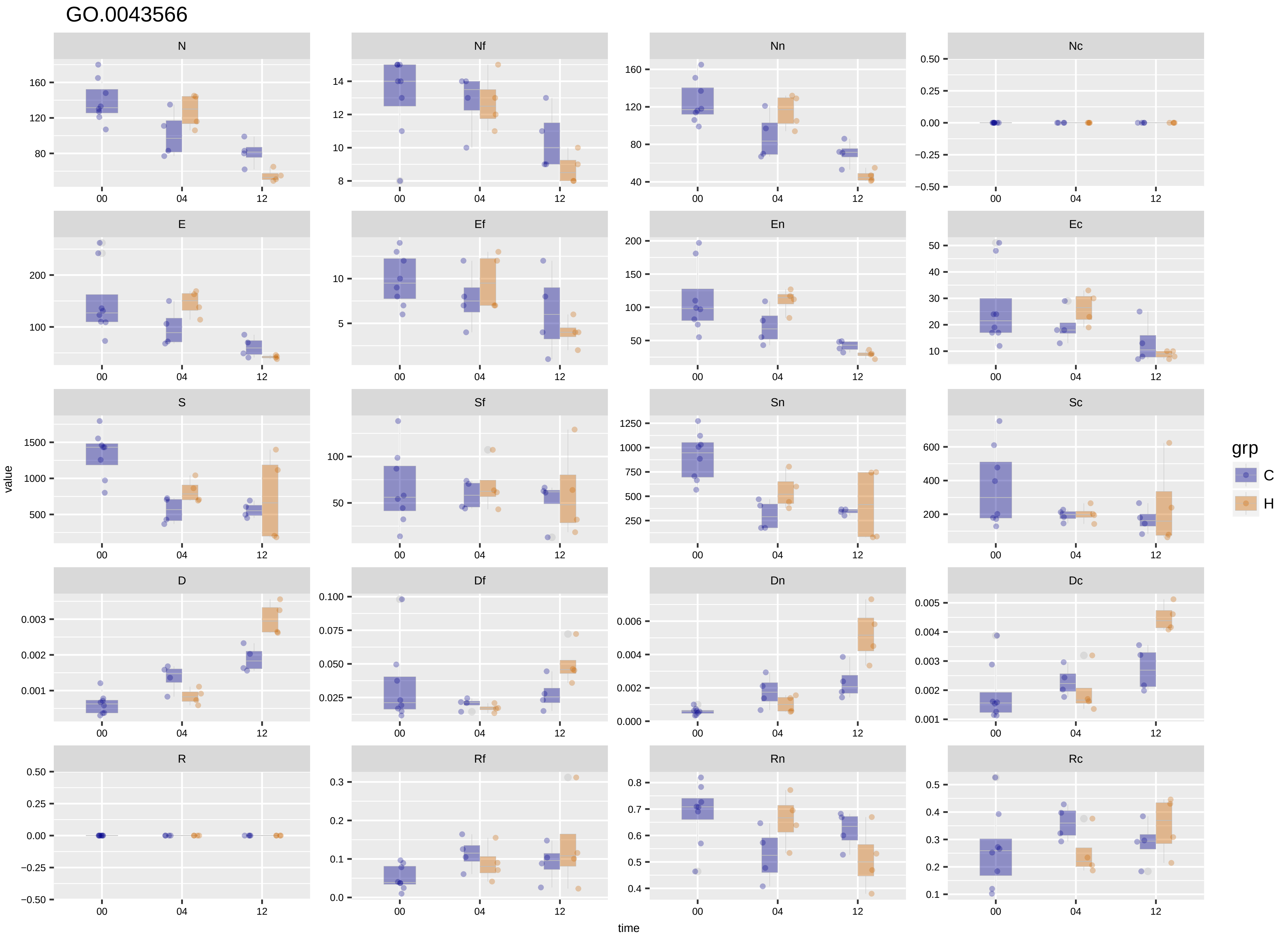

GO.0043566
N
Nf
Nn
Nc
E
Ef
En
Ec
S
Sf
Sn
Sc
D
Df
Dn
Dc
R
Rf
Rn
Rc
value
time
grp
C
H

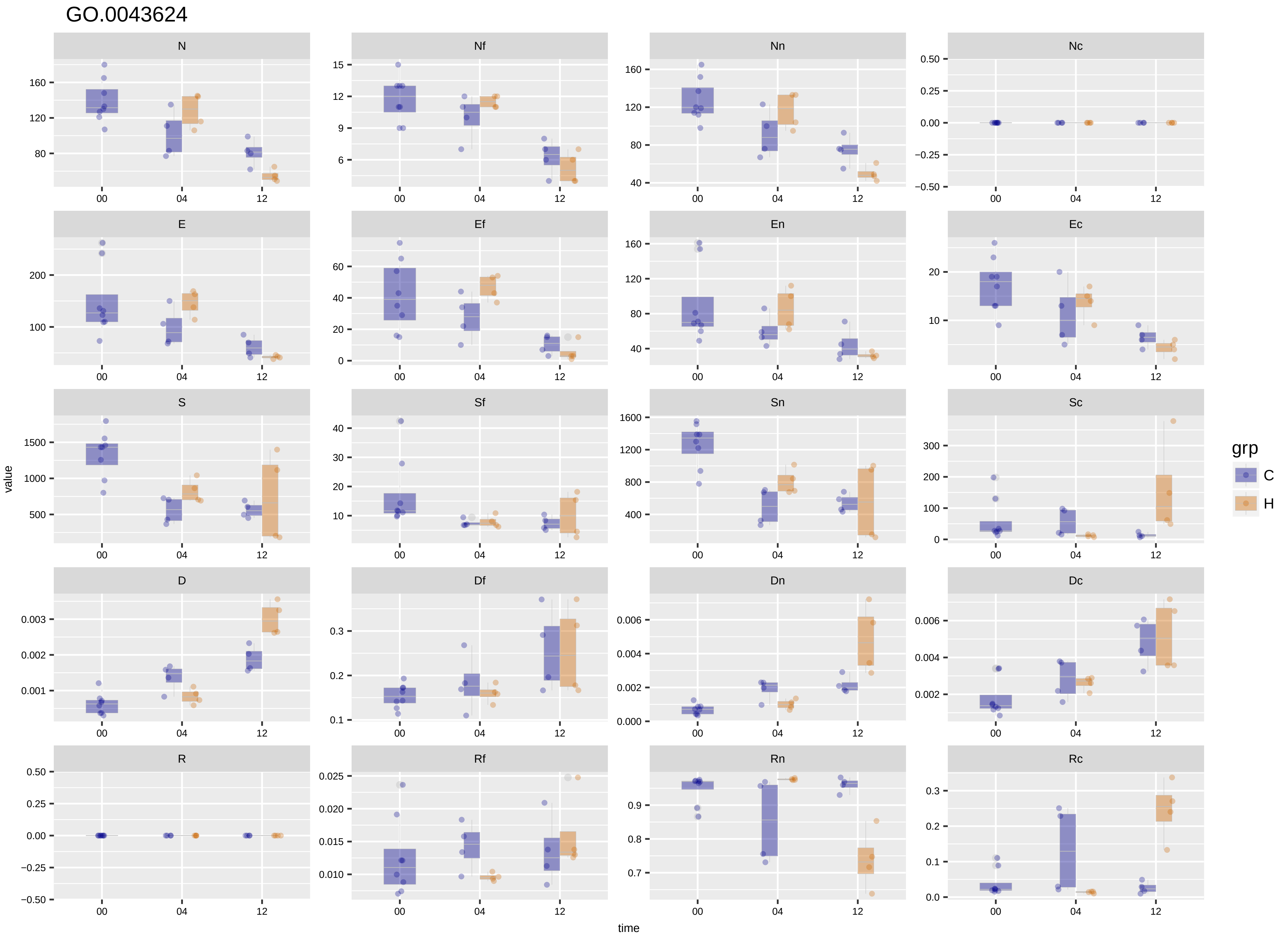
GO.0043624
N
Nf
Nn
Nc
E
Ef
En
Ec
S
Sf
Sn
Sc
D
Df
Dn
Dc
R
Rf
Rn
Rc
value
time
grp
C
H
00
04
12

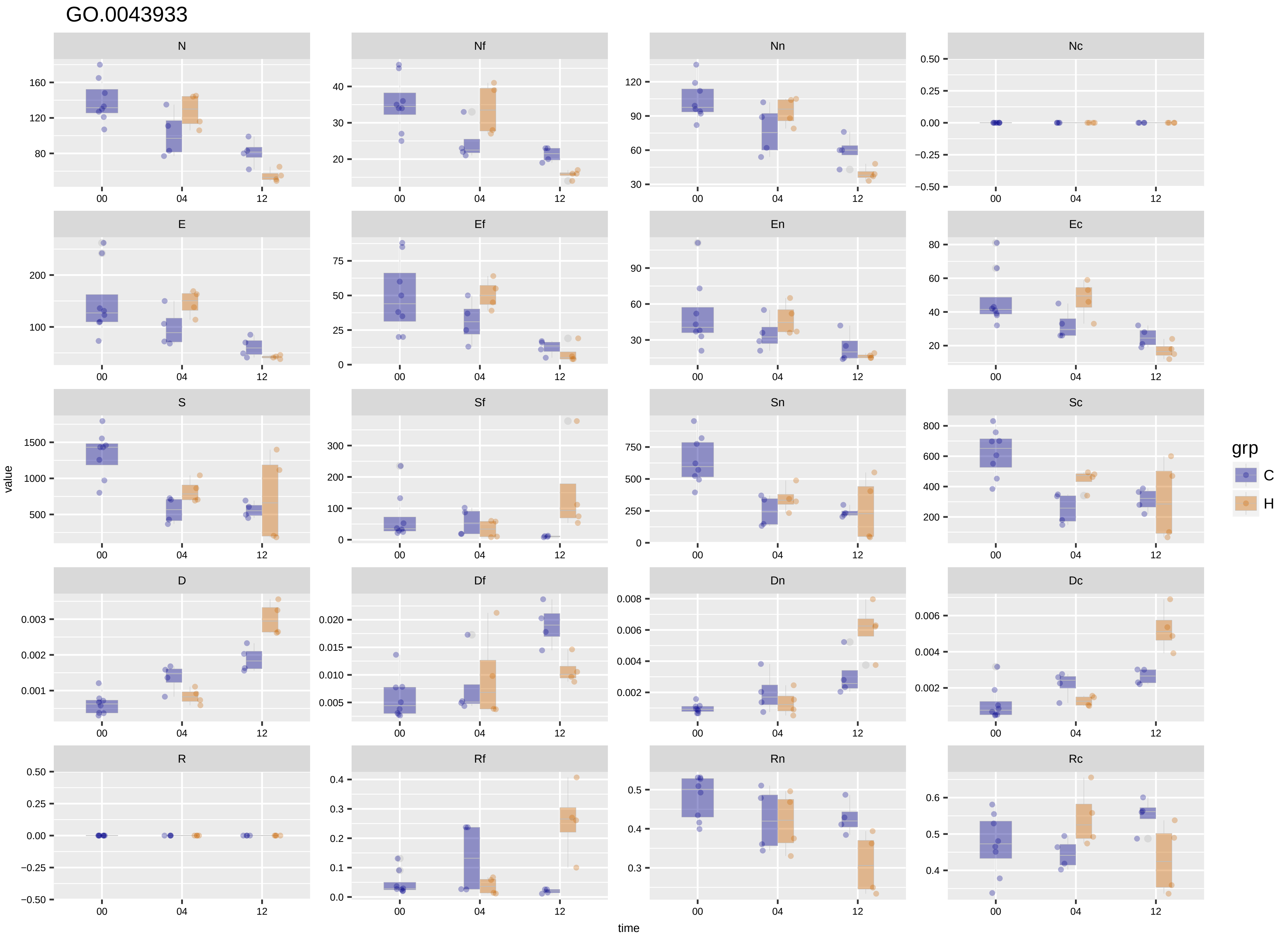
GO.0043933
N
Nf
Nn
Nc
E
Ef
En
Ec
S
Sf
Sn
Sc
D
Df
Dn
Dc
R
Rf
Rn
Rc
value
time
grp
C
H

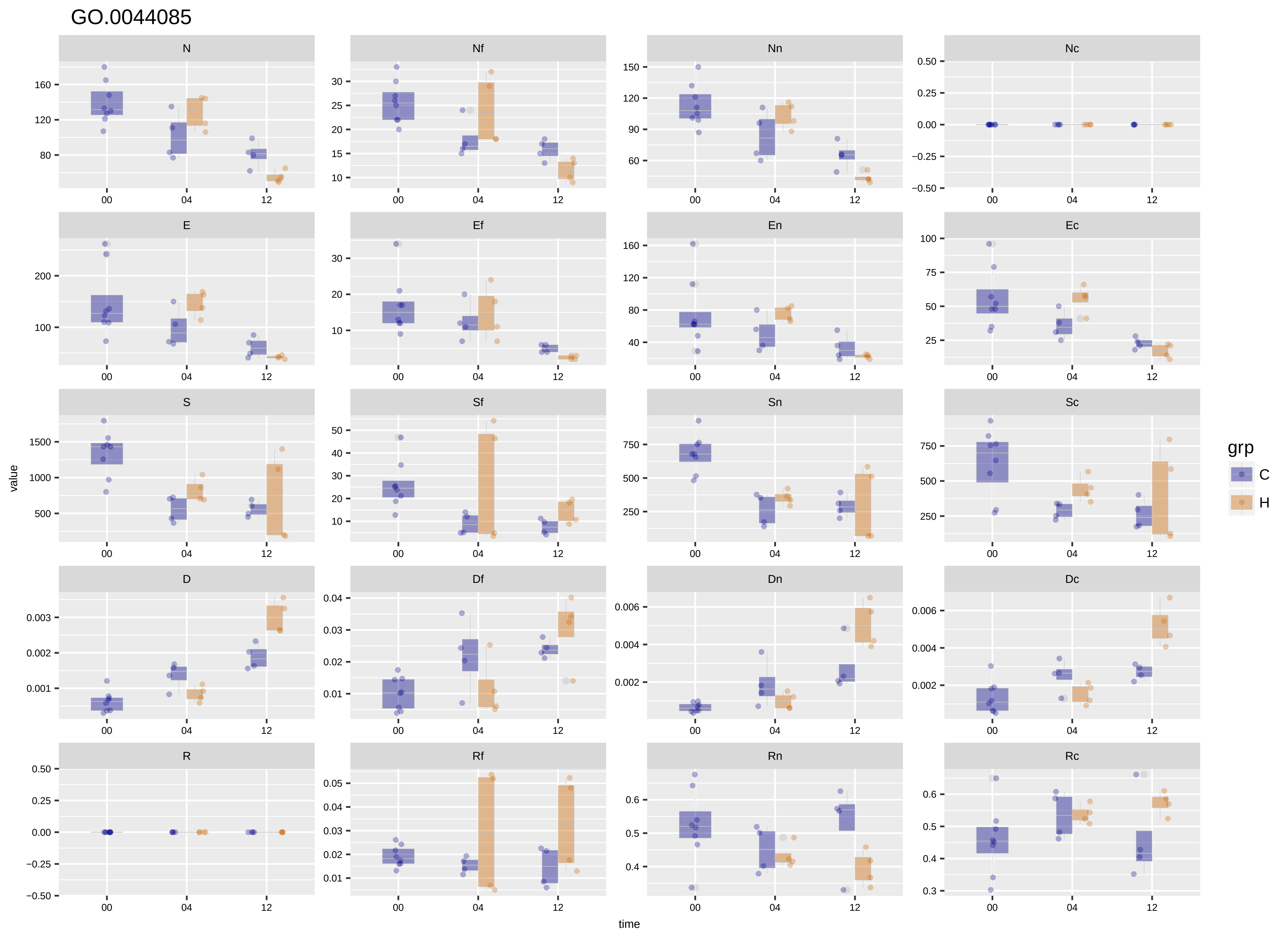

GO.0044085
N
Nf
Nn
Nc
E
Ef
En
Ec
S
Sf
Sn
Sc
D
Df
Dn
Dc
R
Rf
Rn
Rc
00
04
12
value
time
grp
C
H

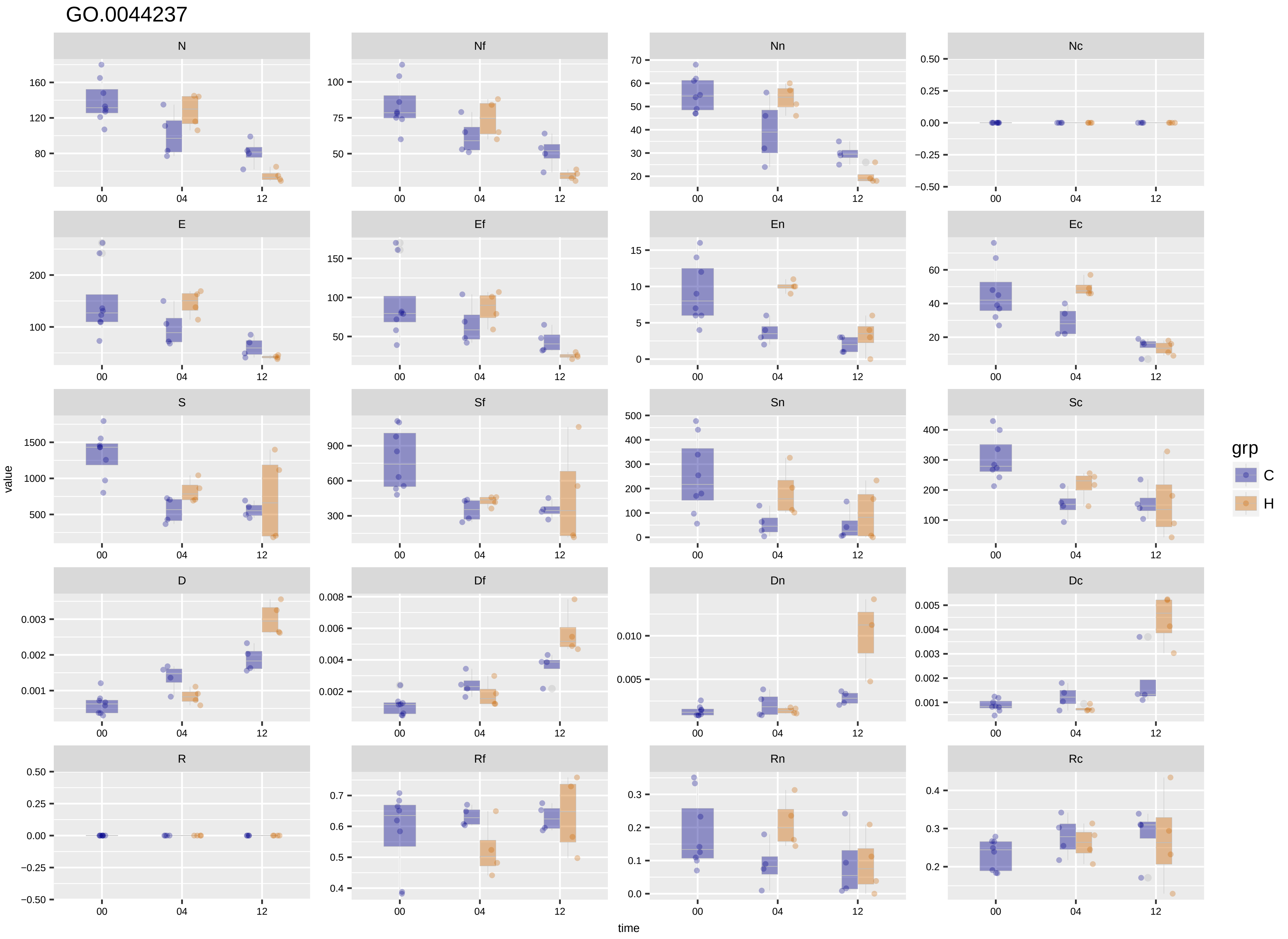

GO.0044237
N
Nf
Nn
Nc
E
Ef
En
Ec
S
Sf
Sn
Sc
D
Df
Dn
Dc
R
Rf
Rn
Rc
00
04
12
value
time
grp
C
H

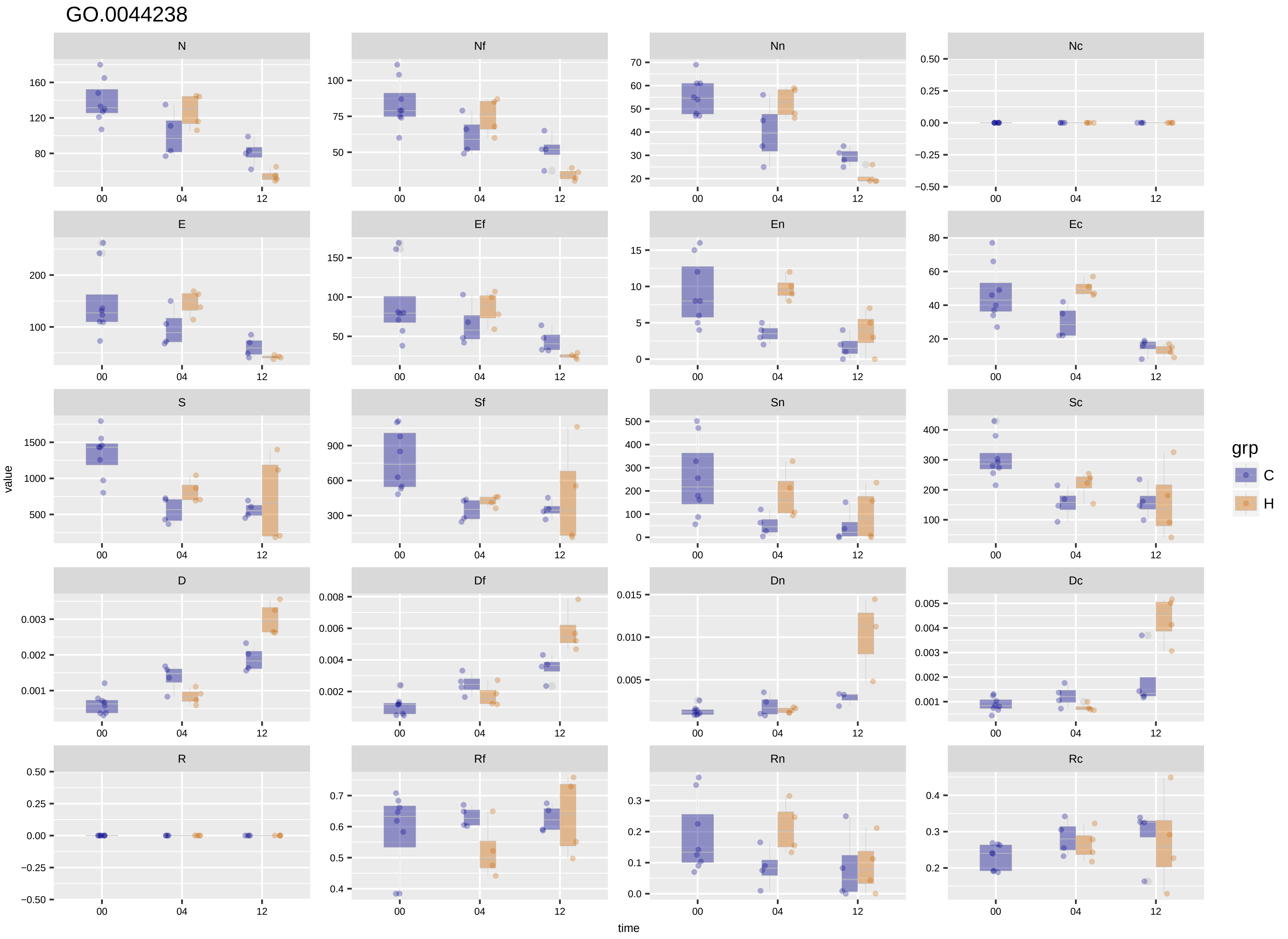

GO.0044238
N
Nf
Nn
Nc
E
Ef
En
Ec
S
Sf
Sn
Sc
D
Df
Dn
Dc
R
Rf
Rn
Rc
00
04
12
value
time
grp
C
H

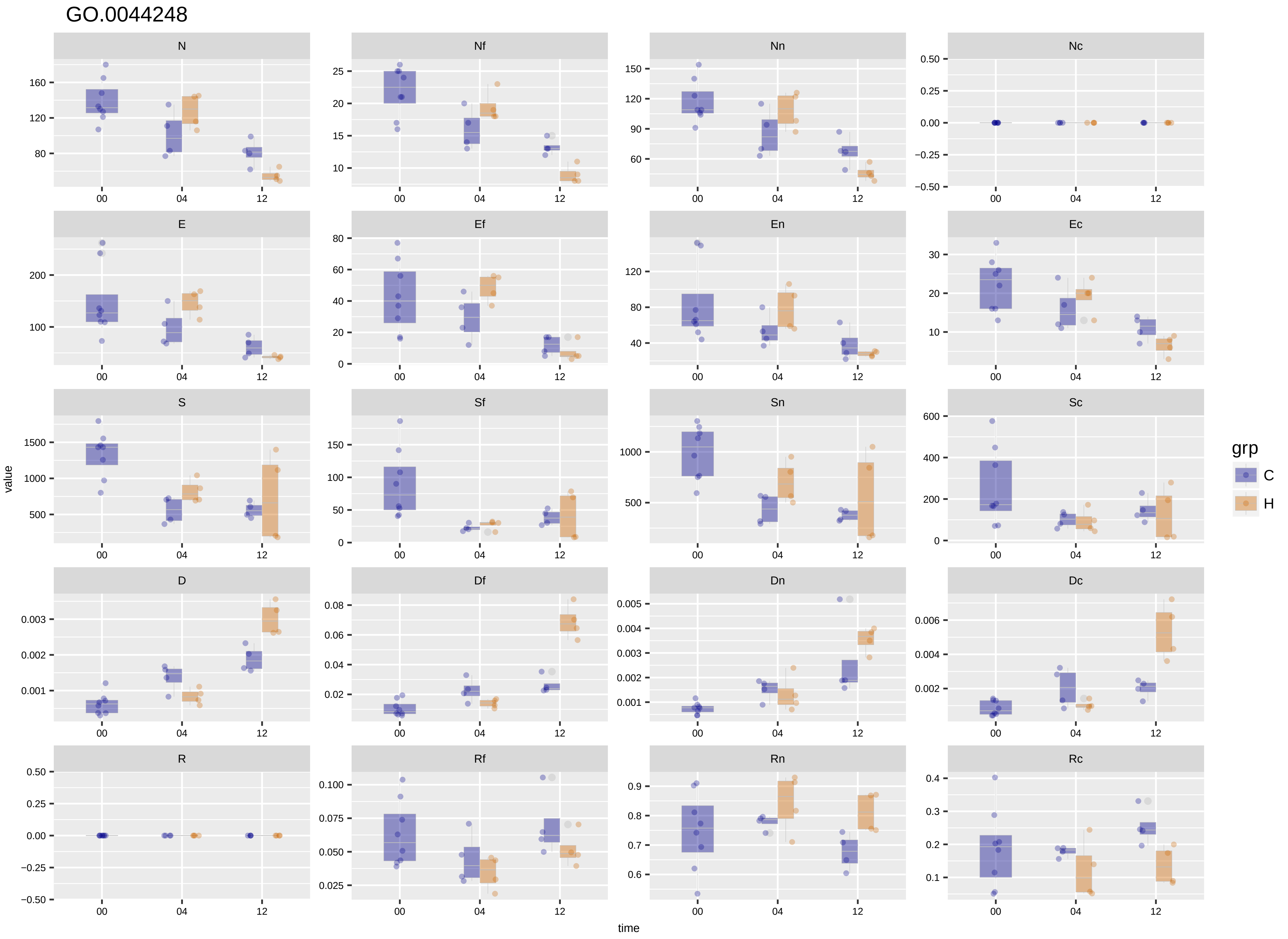
GO.0044248
N
Nf
Nn
Nc
E
Ef
En
Ec
S
Sf
Sn
Sc
D
Df
Dn
Dc
R
Rf
Rn
Rc
00
04
12
value
time
grp
C
H

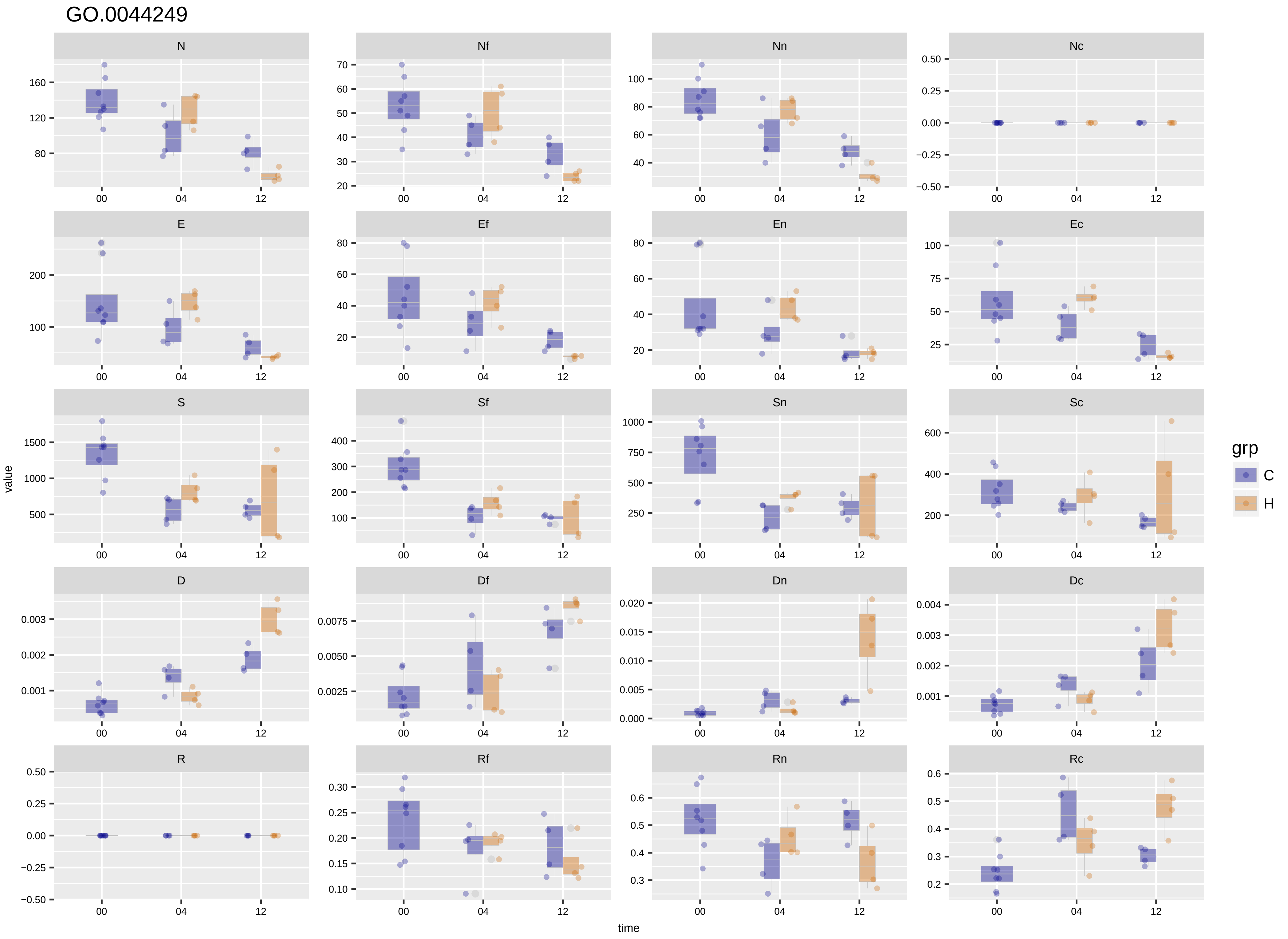

GO.0044249
N
Nf
Nn
Nc
E
Ef
En
Ec
S
Sf
Sn
Sc
D
Df
Dn
Dc
R
Rf
Rn
Rc
00
04
12
value
time
grp
C
H

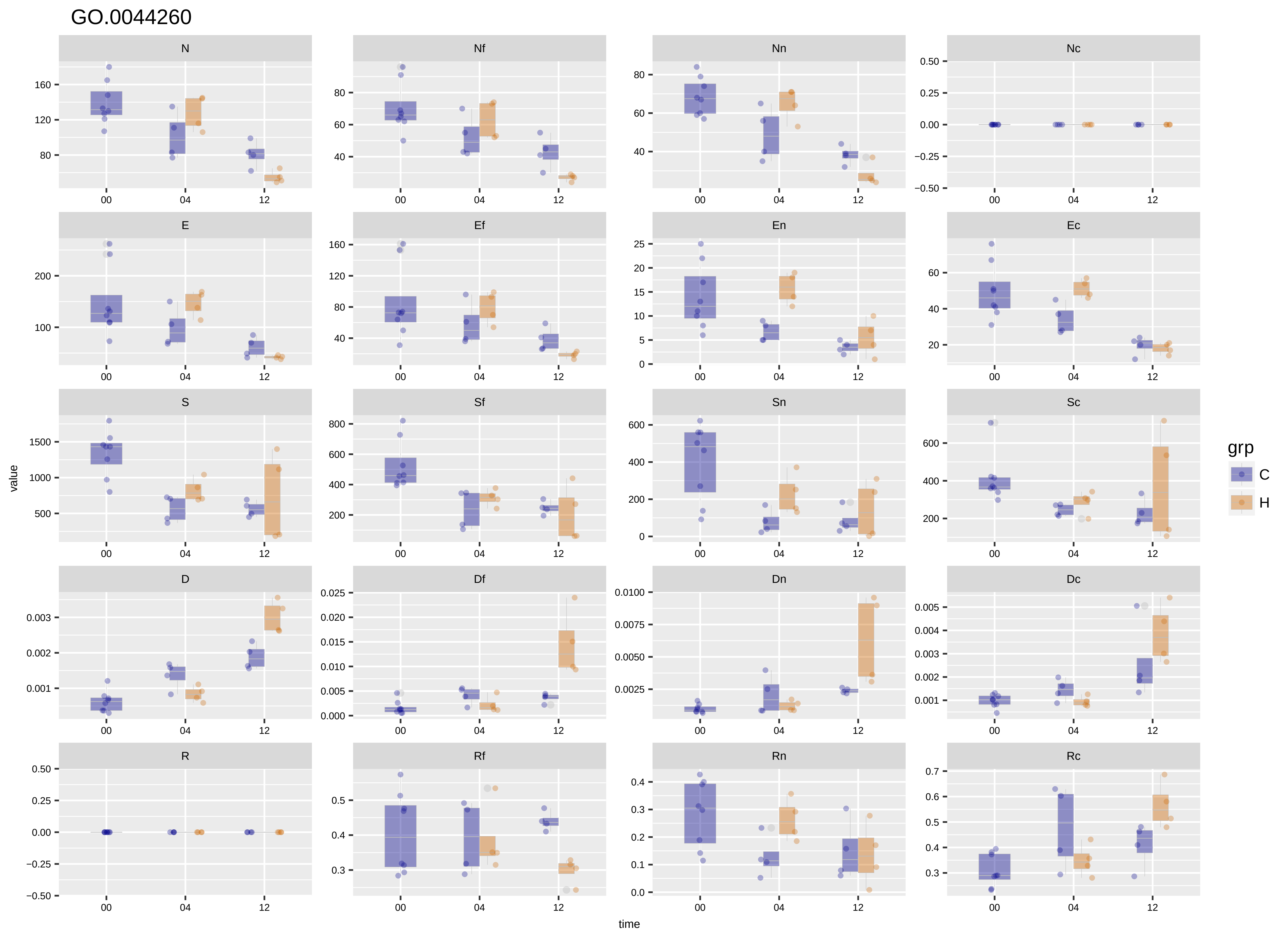
GO.0044260
N
Nf
Nn
Nc
E
Ef
En
Ec
S
Sf
Sn
Sc
D
Df
Dn
Dc
R
Rf
Rn
Rc
value
time
grp
C
H

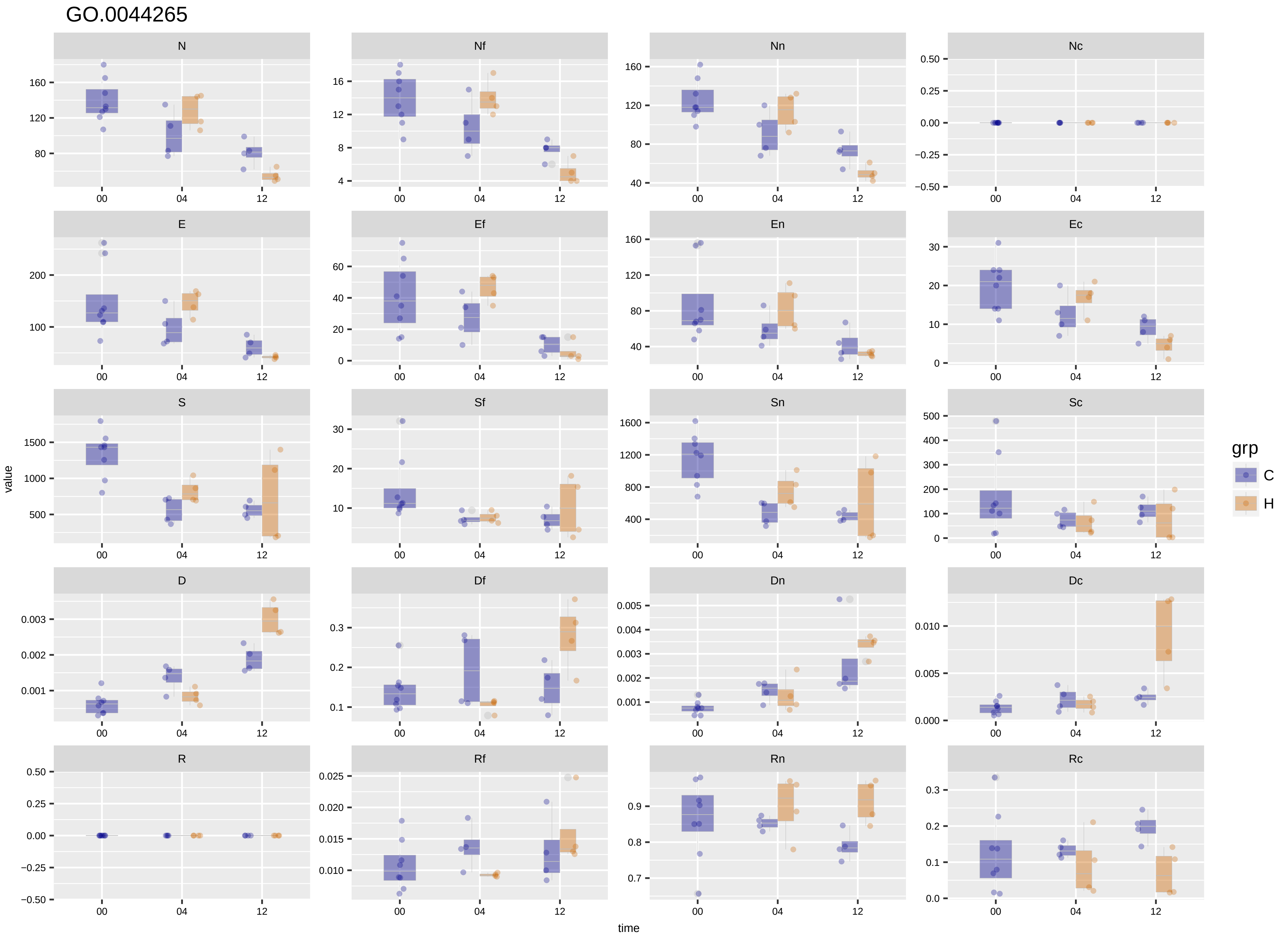

GO.0044265
N
Nf
Nn
Nc
E
Ef
En
Ec
S
Sf
Sn
Sc
D
Df
Dn
Dc
R
Rf
Rn
Rc
value
time
grp
C
H

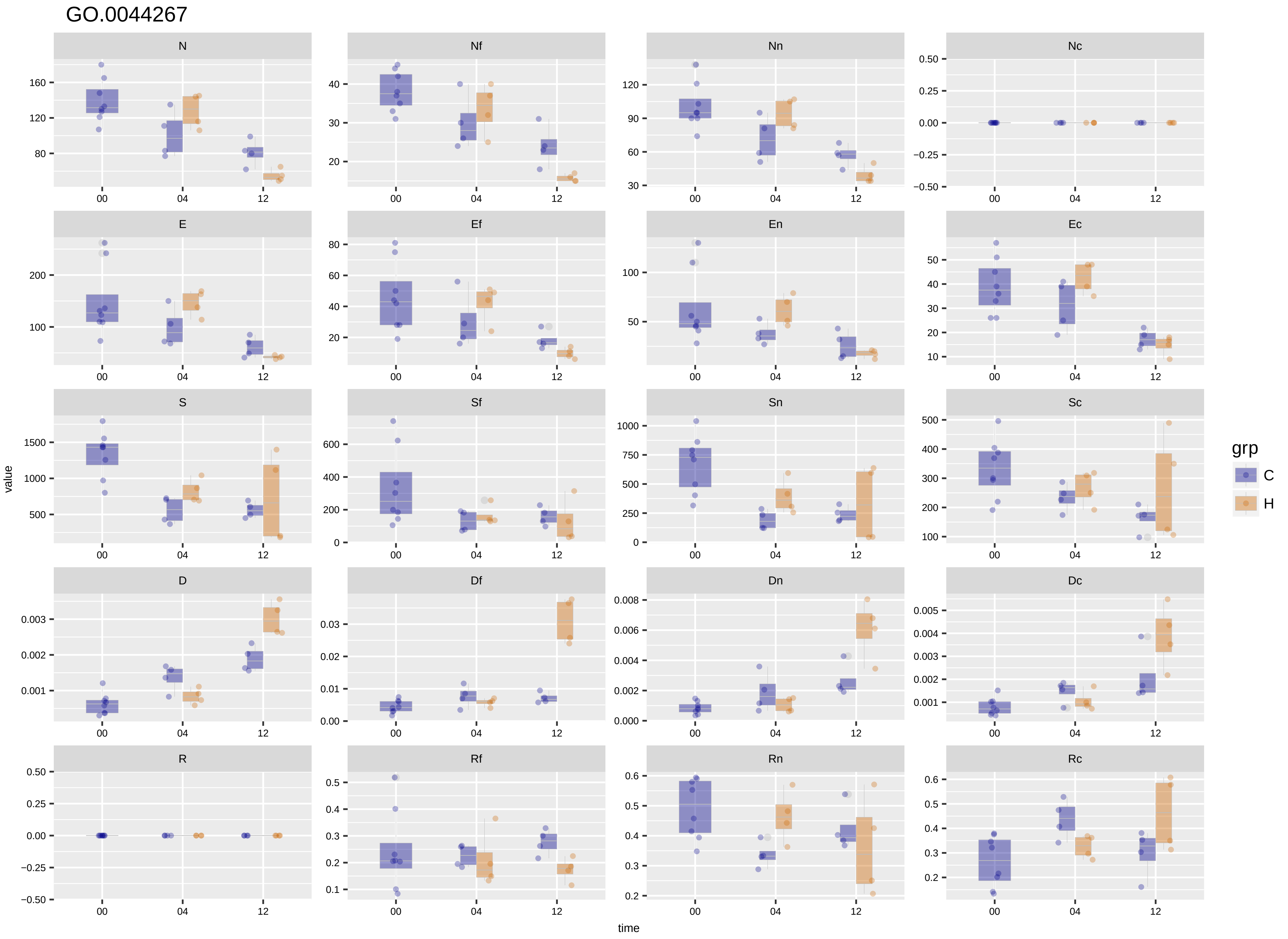
GO.0044267
N
Nf
Nn
Nc
E
Ef
En
Ec
S
Sf
Sn
Sc
D
Df
Dn
Dc
R
Rf
Rn
Rc
00
04
12
value
time
grp
C
H

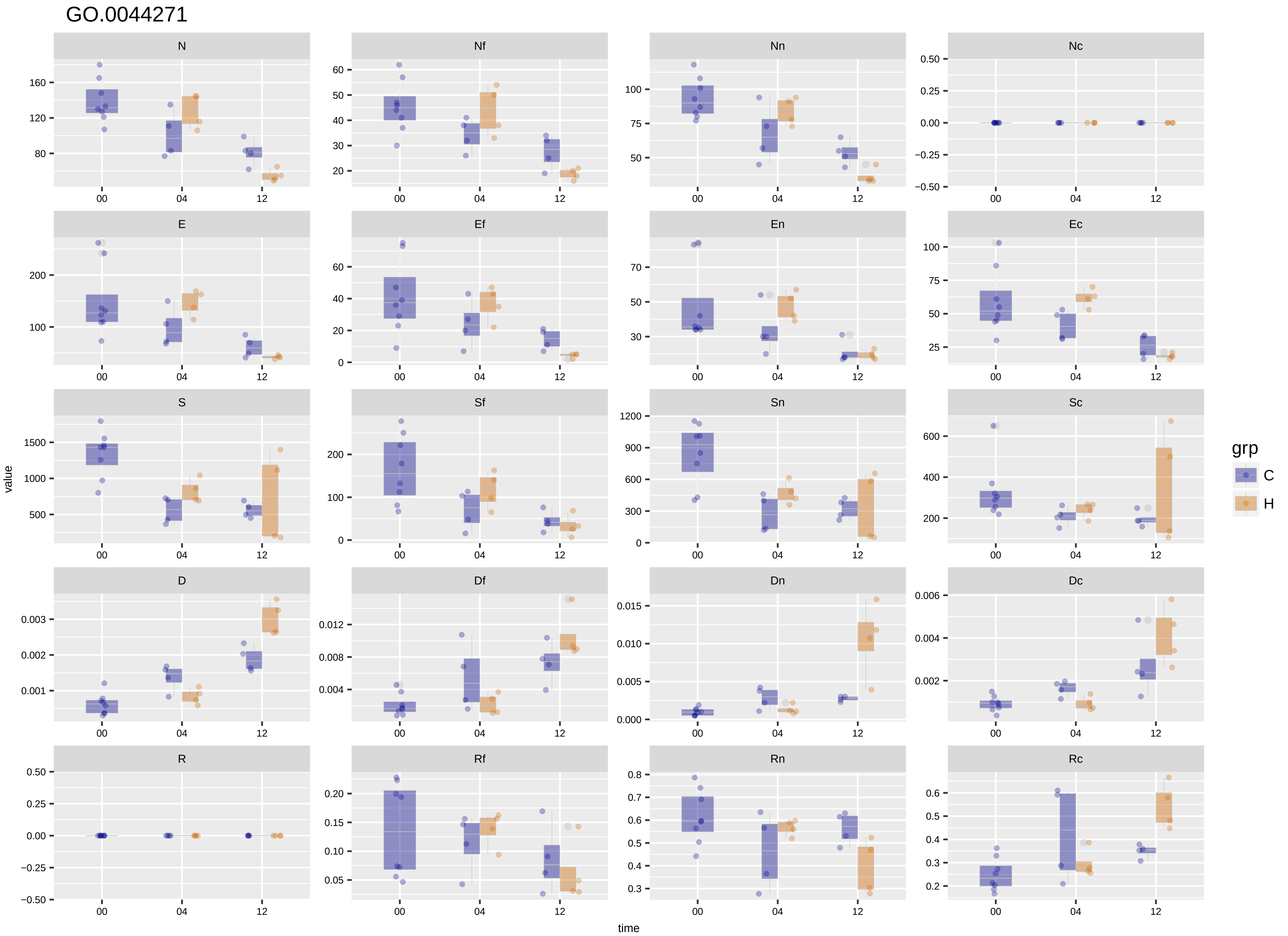

GO.0044271
N
Nf
Nn
Nc
E
Ef
En
Ec
S
Sf
Sn
Sc
D
Df
Dn
Dc
R
Rf
Rn
Rc
00
04
12
value
time
grp
C
H

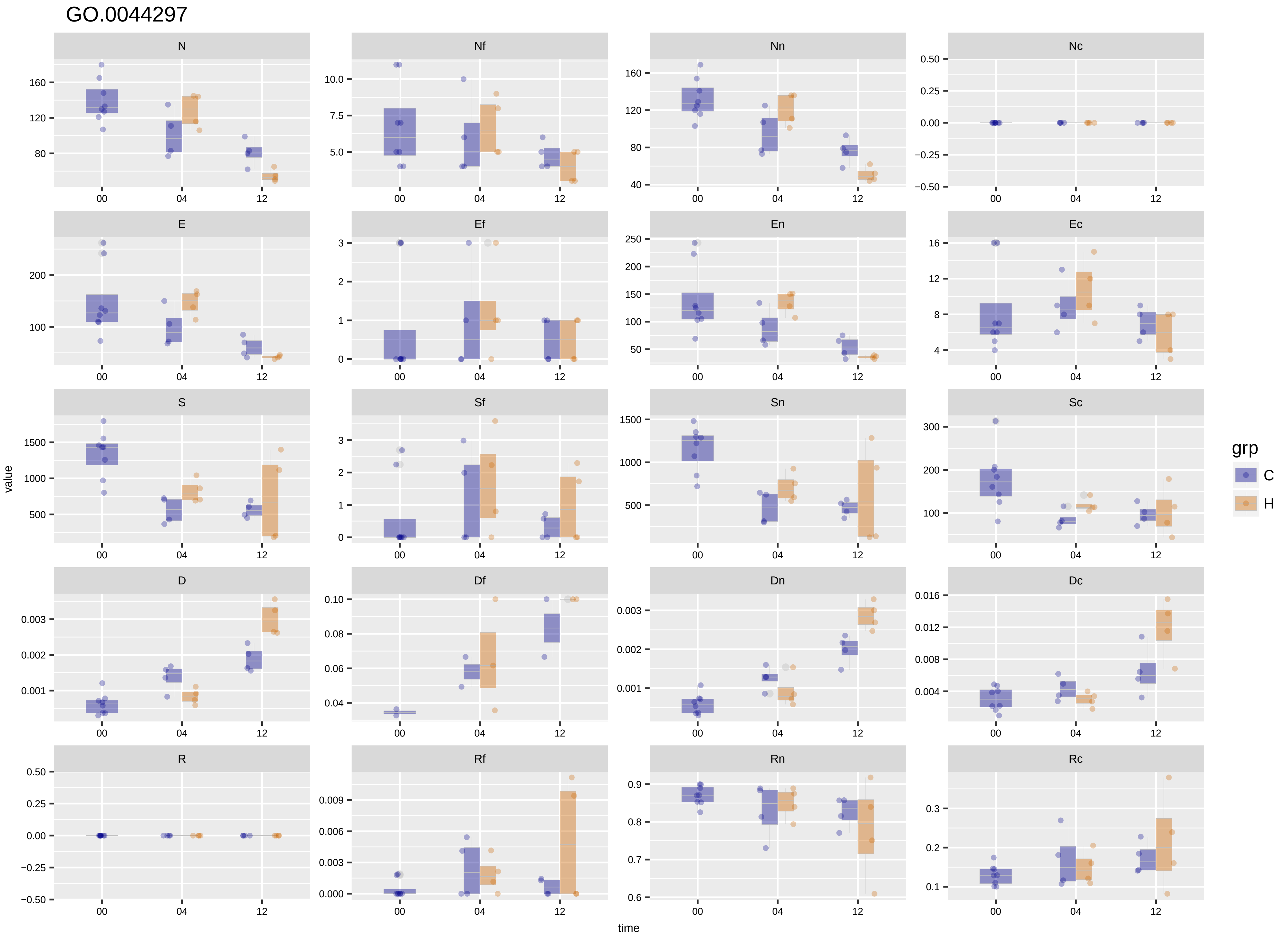

GO.0044297
N
Nf
Nn
Nc
E
Ef
En
Ec
S
Sf
Sn
Sc
D
Df
Dn
Dc
R
Rf
Rn
Rc
value
time
grp
C
H

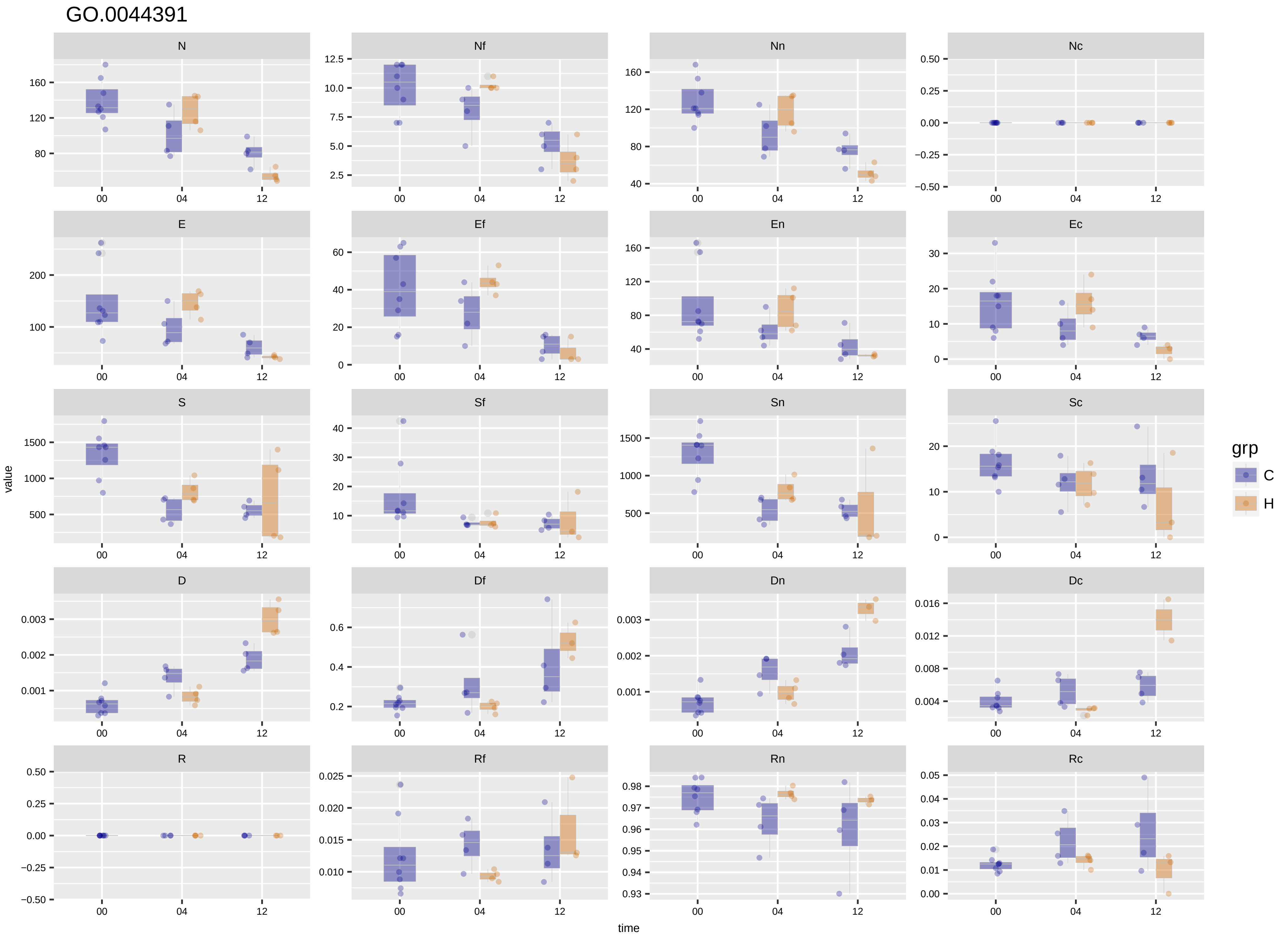
GO.0044391
N
Nf
Nn
Nc
E
Ef
En
Ec
S
Sf
Sn
Sc
D
Df
Dn
Dc
R
Rf
Rn
Rc
value
time
grp
C
H

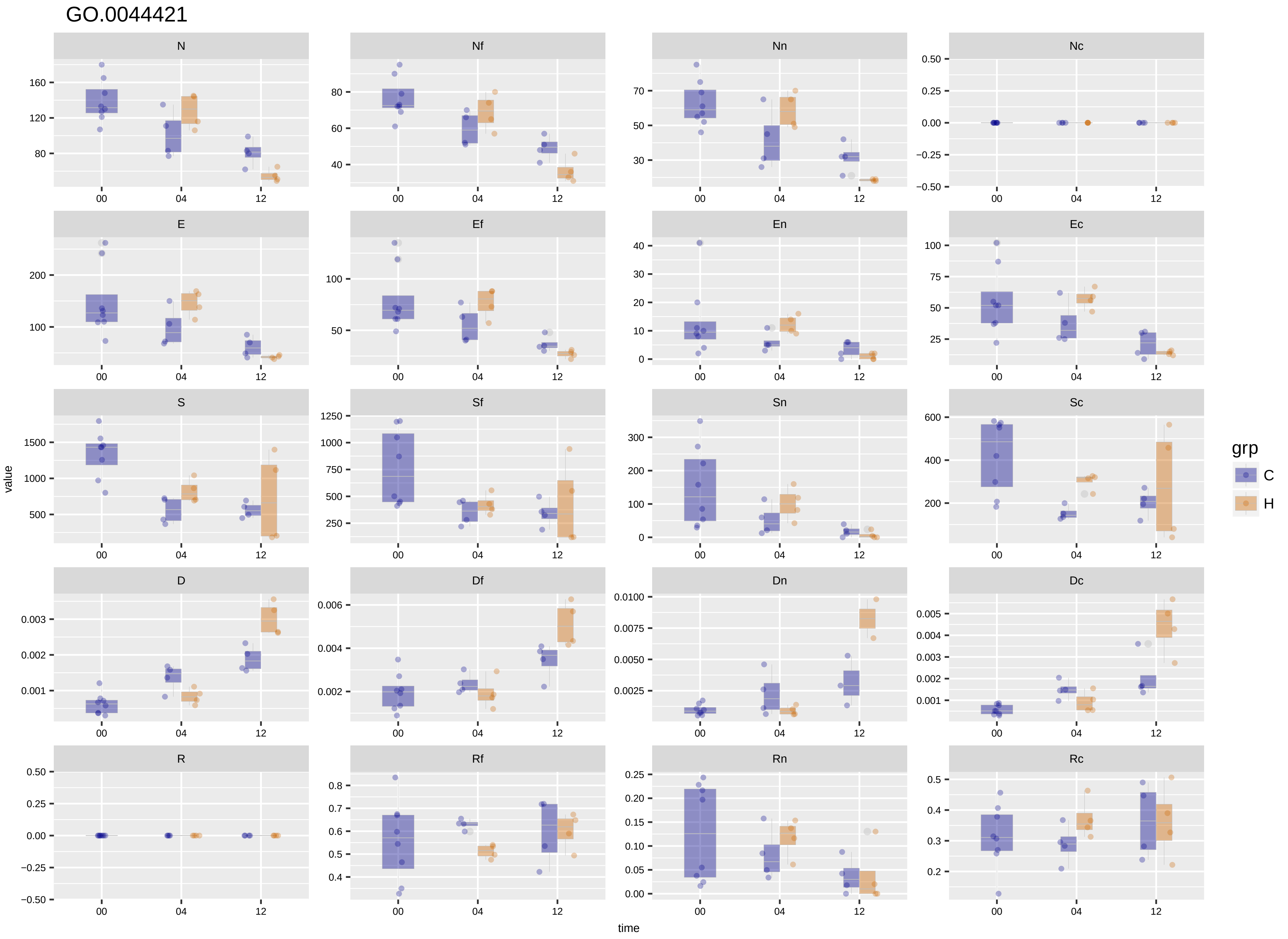

GO.0044421
N
Nf
Nn
Nc
E
Ef
En
Ec
S
Sf
Sn
Sc
D
Df
Dn
Dc
R
Rf
Rn
Rc
value
time
grp
C
H

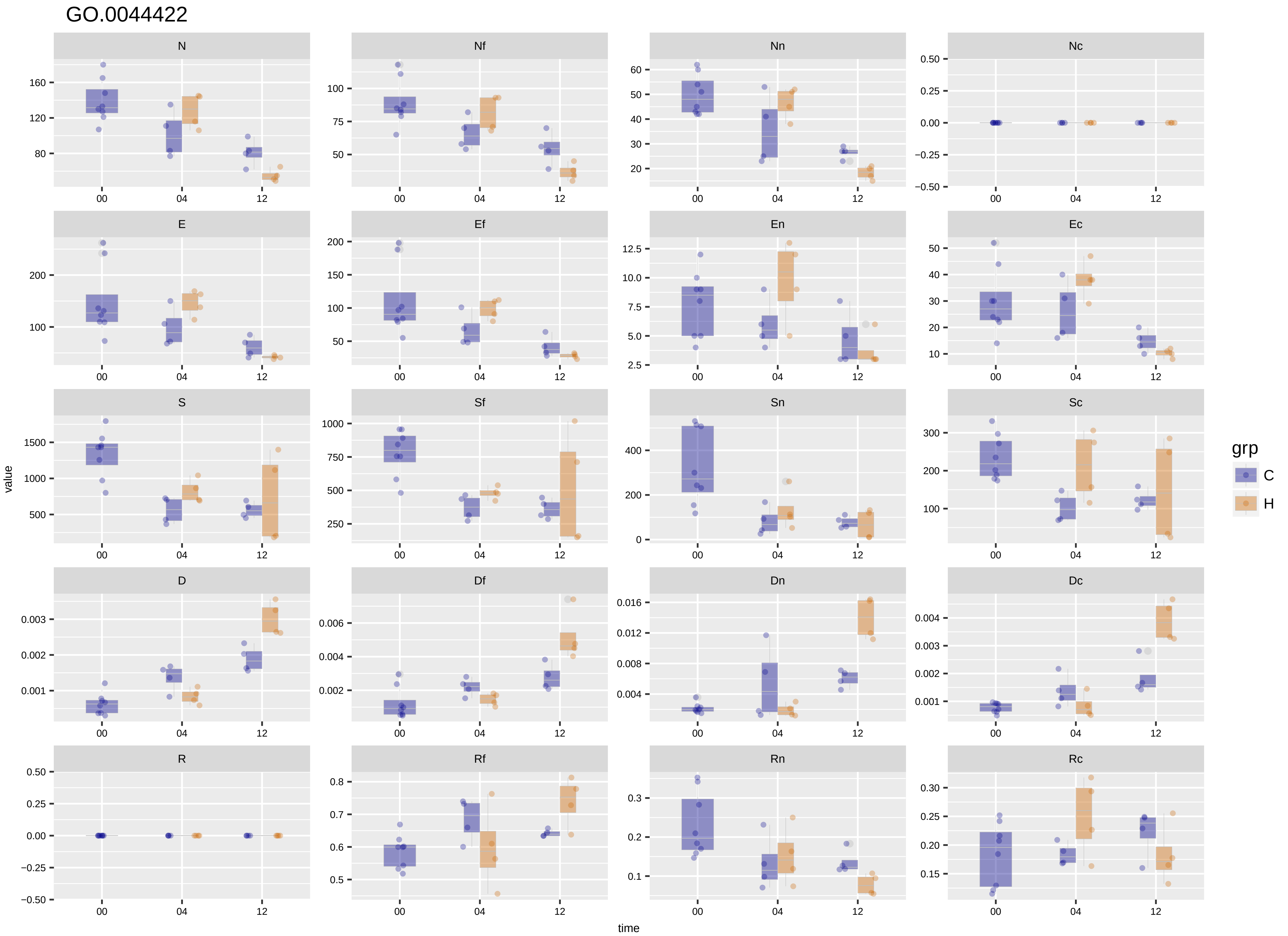

GO.0044422
N
Nf
Nn
Nc
E
Ef
En
Ec
S
Sf
Sn
Sc
D
Df
Dn
Dc
R
Rf
Rn
Rc
value
time
00
04
12
grp
C
H

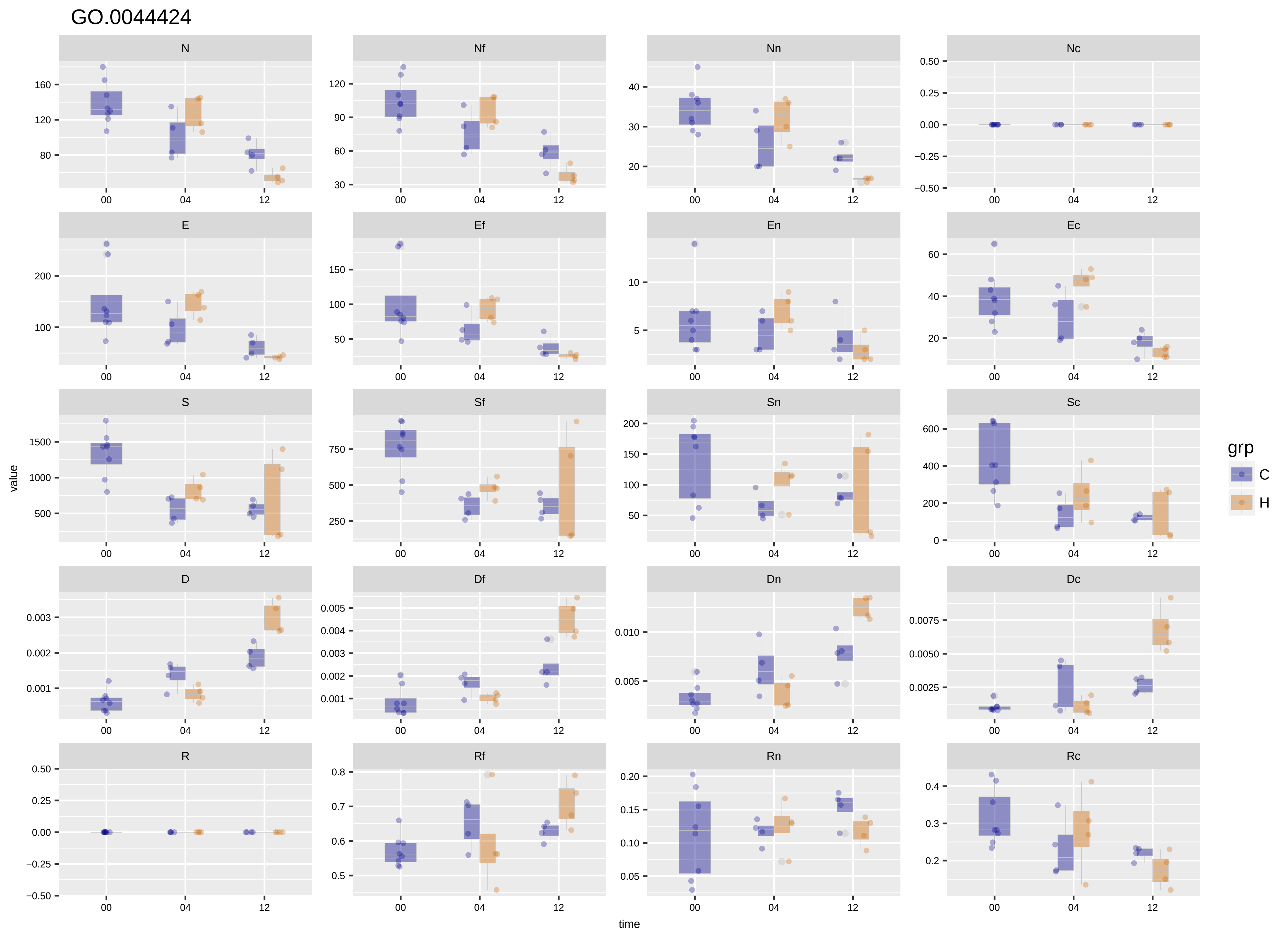
GO.0044424
N
Nf
Nn
Nc
E
Ef
En
Ec
S
Sf
Sn
Sc
D
Df
Dn
Dc
R
Rf
Rn
Rc
value
time
grp
C
H

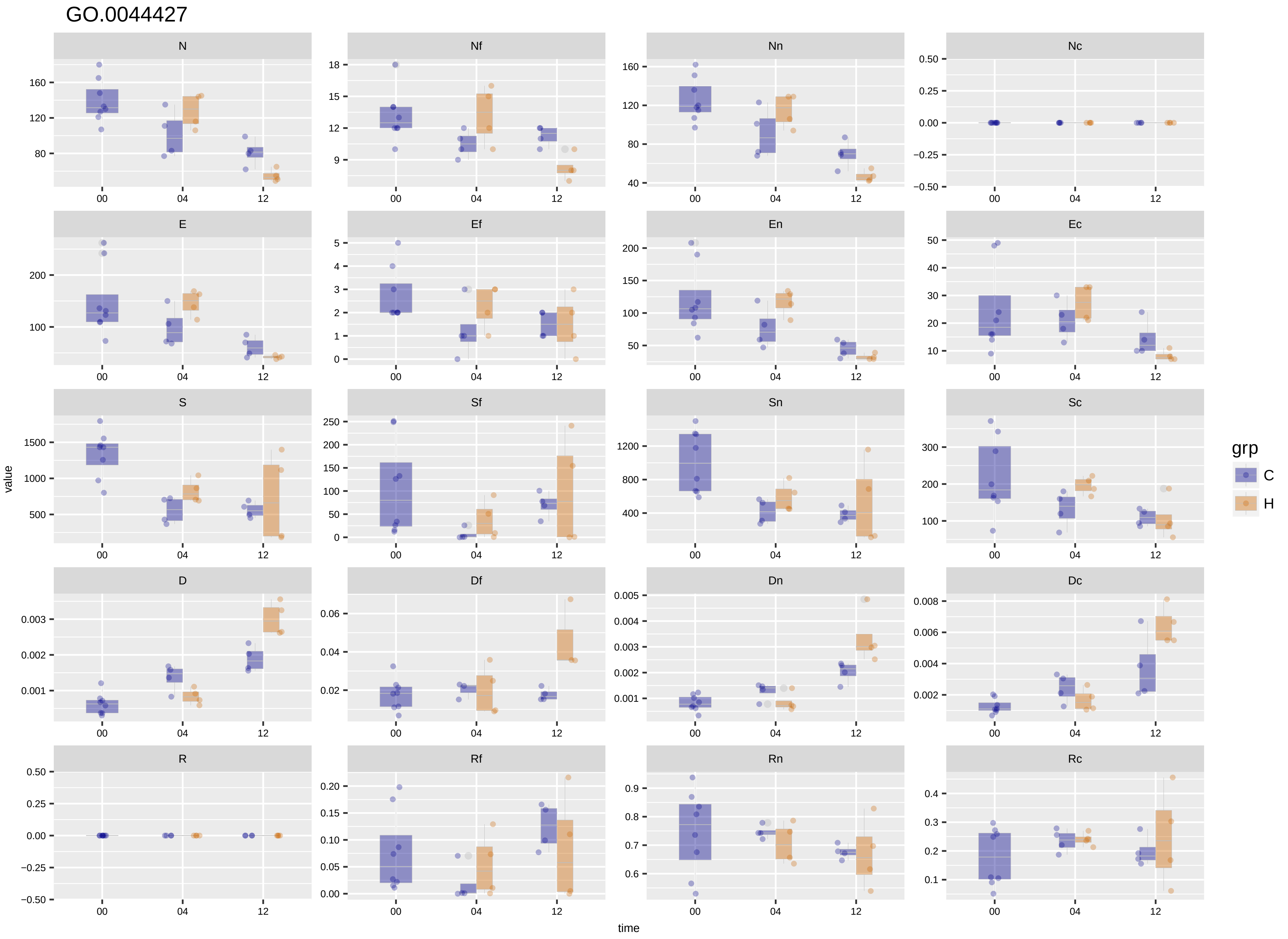
GO.0044427
N
Nf
Nn
Nc
E
Ef
En
Ec
S
Sf
Sn
Sc
D
Df
Dn
Dc
R
Rf
Rn
Rc
00
04
12
value
time
grp
C
H

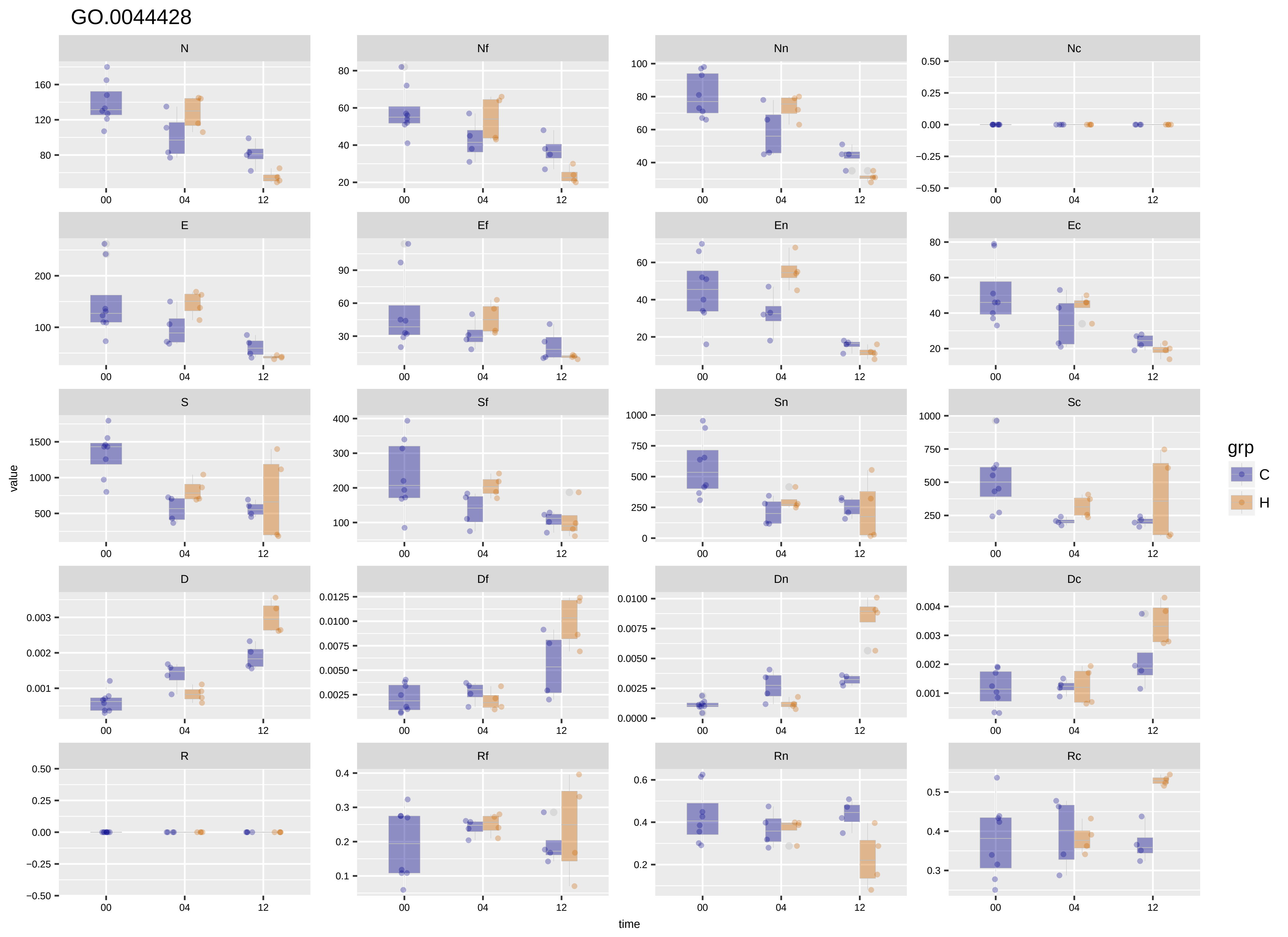

GO.0044428
N
Nf
Nn
Nc
E
Ef
En
Ec
S
Sf
Sn
Sc
D
Df
Dn
Dc
R
Rf
Rn
Rc
value
time
00
04
12
grp
C
H

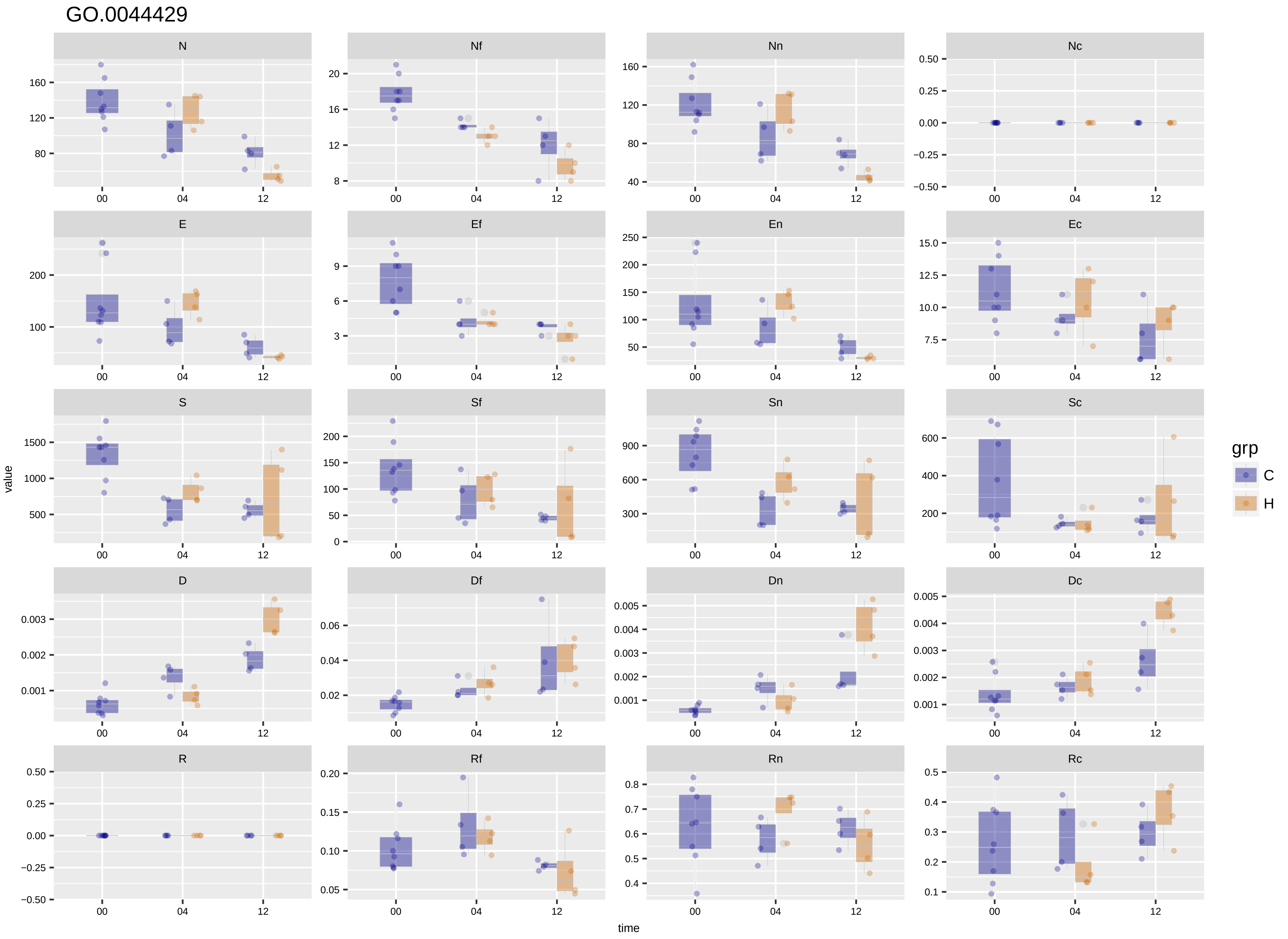

GO.0044429
N
Nf
Nn
Nc
E
Ef
En
Ec
S
Sf
Sn
Sc
D
Df
Dn
Dc
R
Rf
Rn
Rc
value
time
grp
C
H
00
04
12

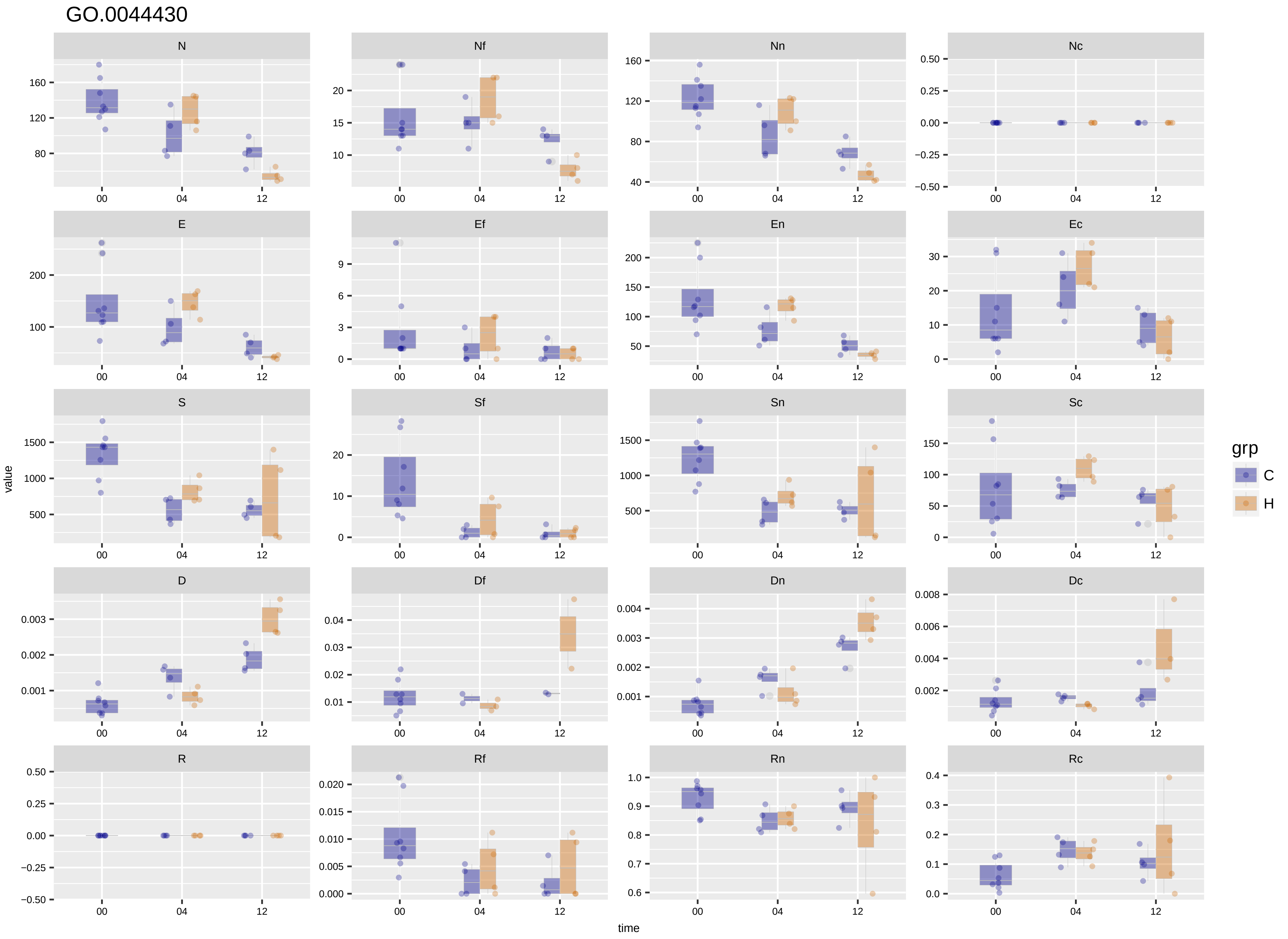

GO.0044430
N
Nf
Nn
Nc
E
Ef
En
Ec
S
Sf
Sn
Sc
D
Df
Dn
Dc
R
Rf
Rn
Rc
value
time
00
04
12
grp
C
H

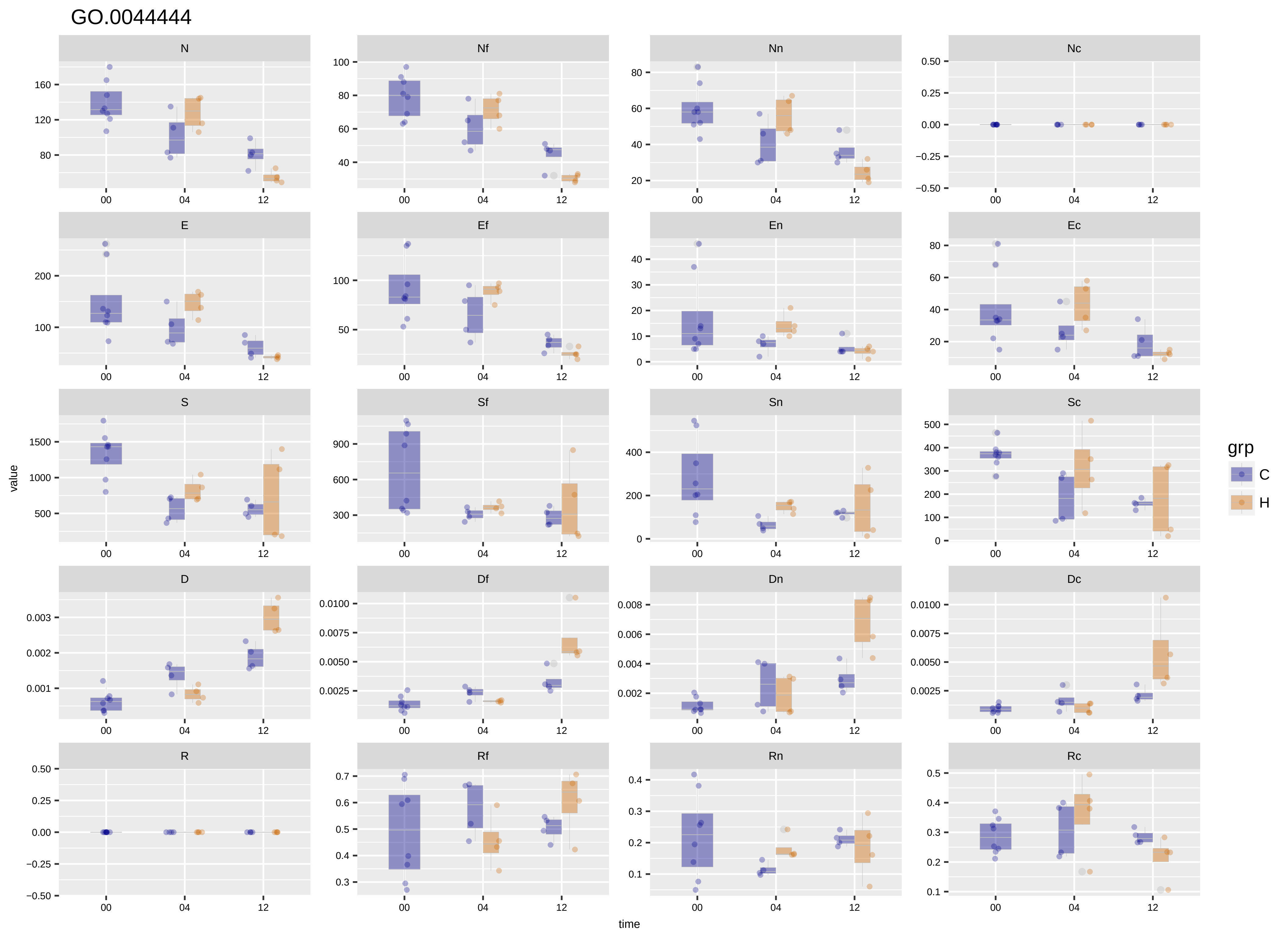
GO.0044444
N
Nf
Nn
Nc
E
Ef
En
Ec
S
Sf
Sn
Sc
D
Df
Dn
Dc
R
Rf
Rn
Rc
value
time
grp
C
H

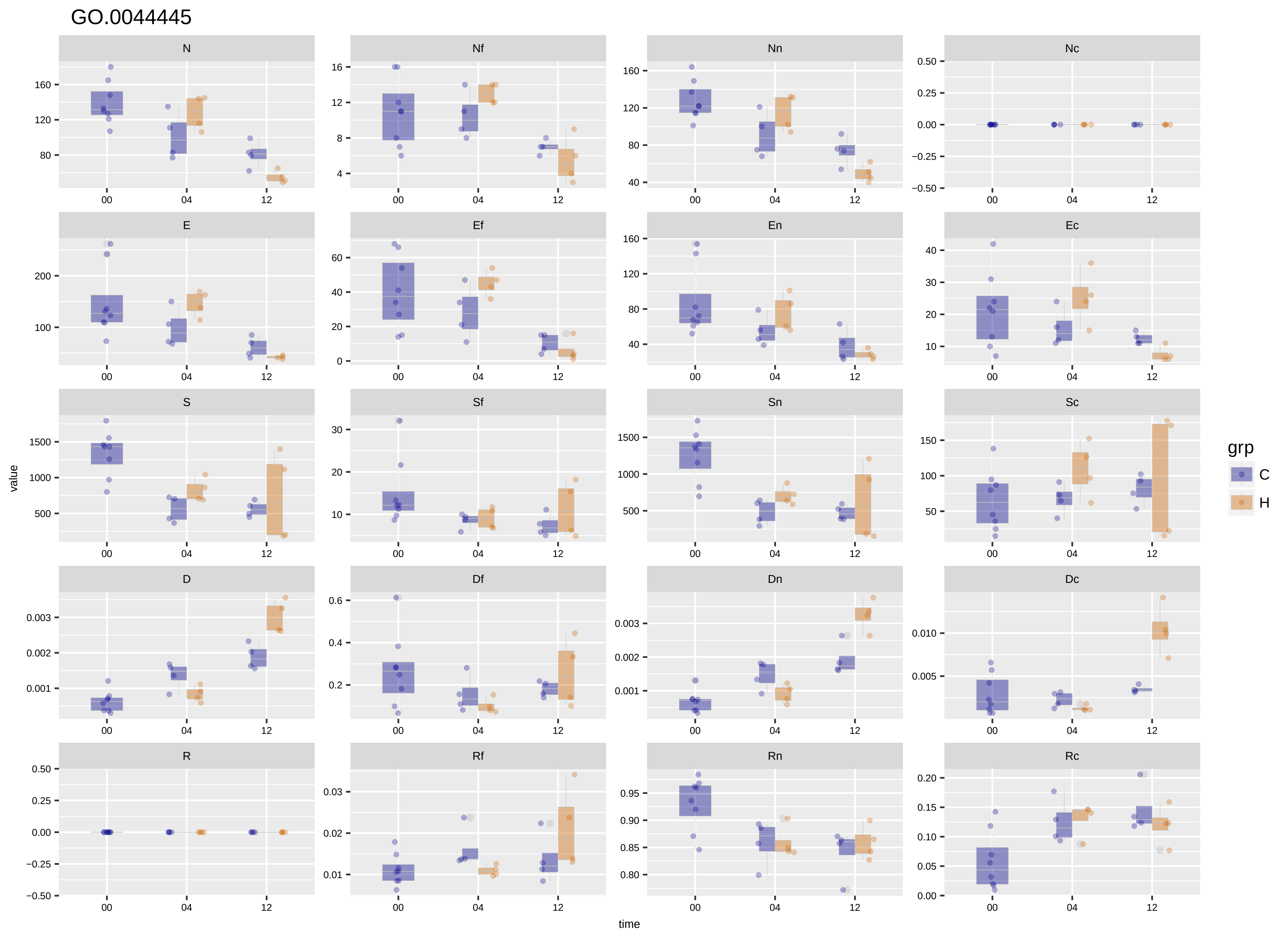

GO.0044445
N
Nf
Nn
Nc
E
Ef
En
Ec
S
Sf
Sn
Sc
D
Df
Dn
Dc
R
Rf
Rn
Rc
value
time
grp
C
H

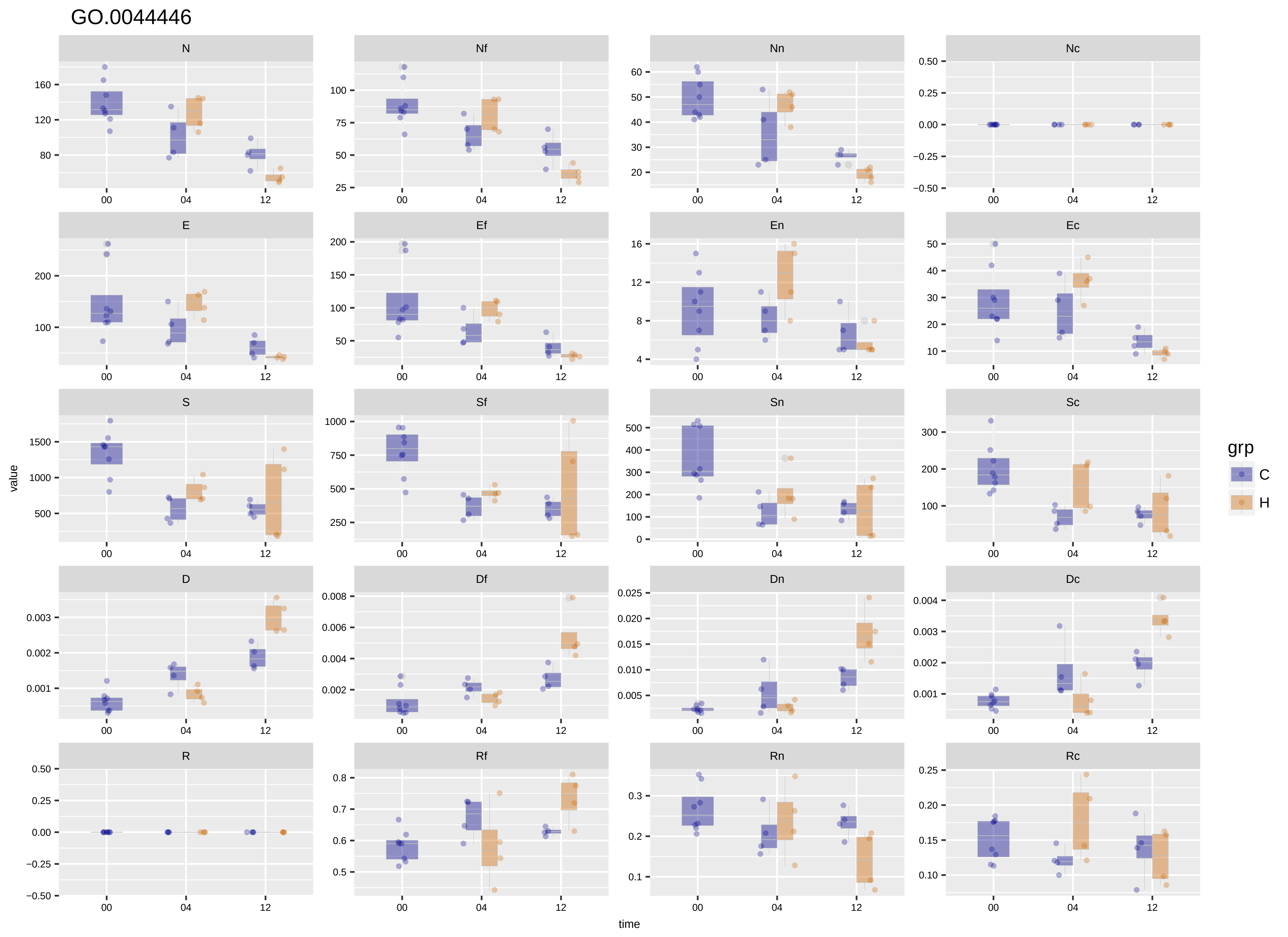

GO.0044446
N
Nf
Nn
Nc
E
Ef
En
Ec
S
Sf
Sn
Sc
D
Df
Dn
Dc
R
Rf
Rn
Rc
value
time
grp
C
H

GO.0044451

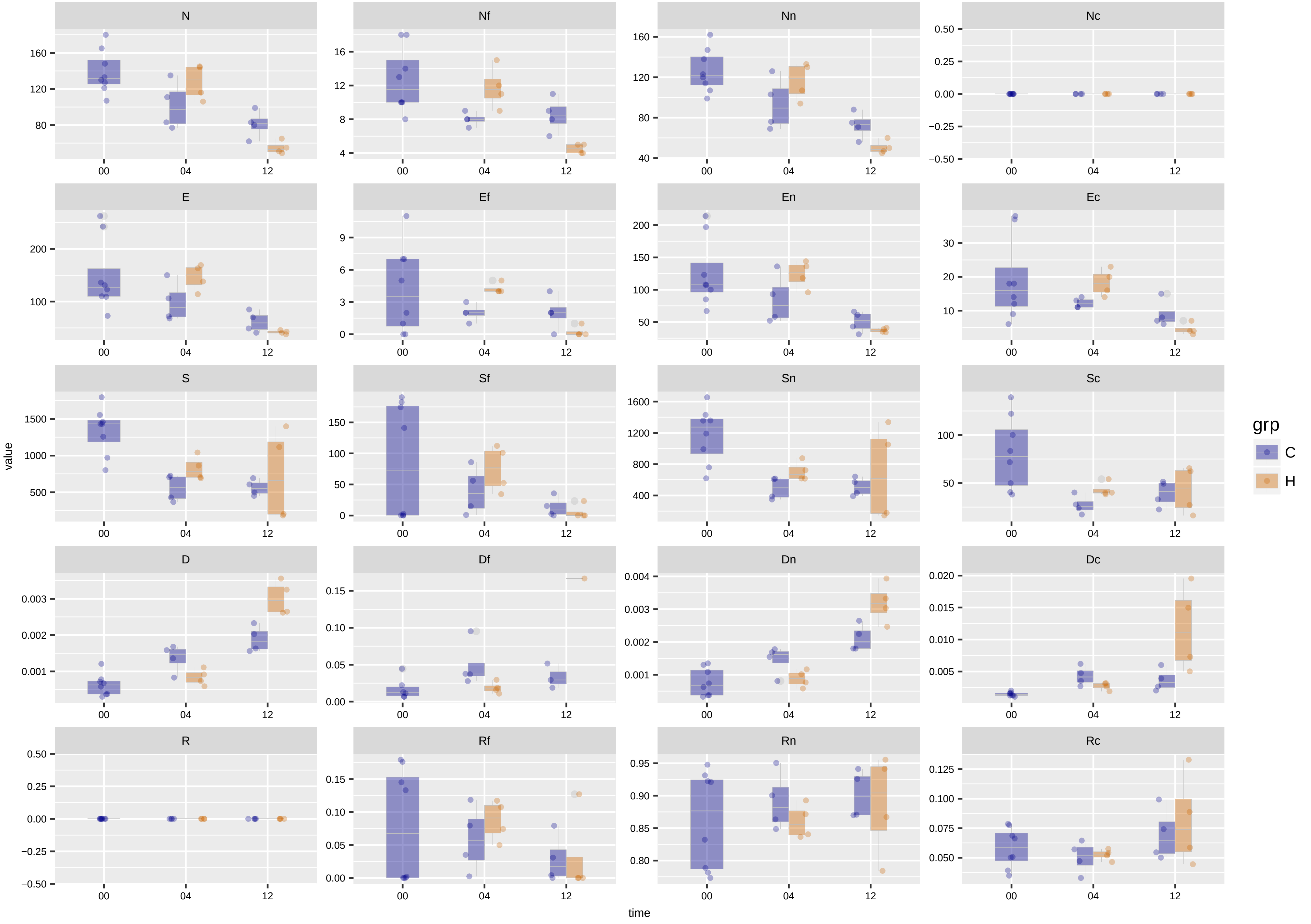

N
Nf
Nn
Nc
E
Ef
En
Ec
S
Sf
Sn
Sc
D
Df
Dn
Dc
R
Rf
Rn
Rc
00
04
12
value
time
grp
C
H

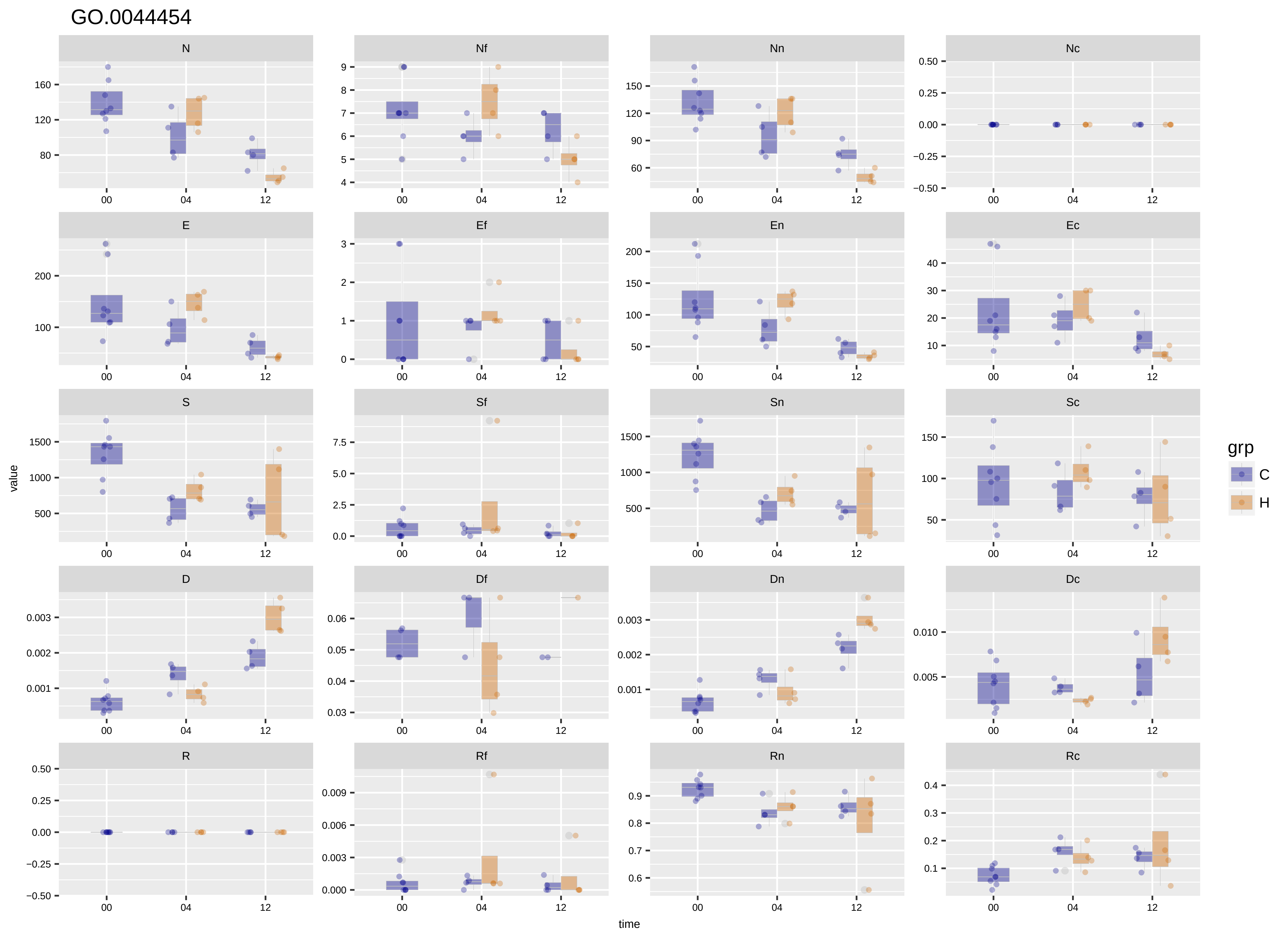

GO.0044454
N
Nf
Nn
Nc
E
Ef
En
Ec
S
Sf
Sn
Sc
D
Df
Dn
Dc
R
Rf
Rn
Rc
value
time
00
04
12
grp
C
H

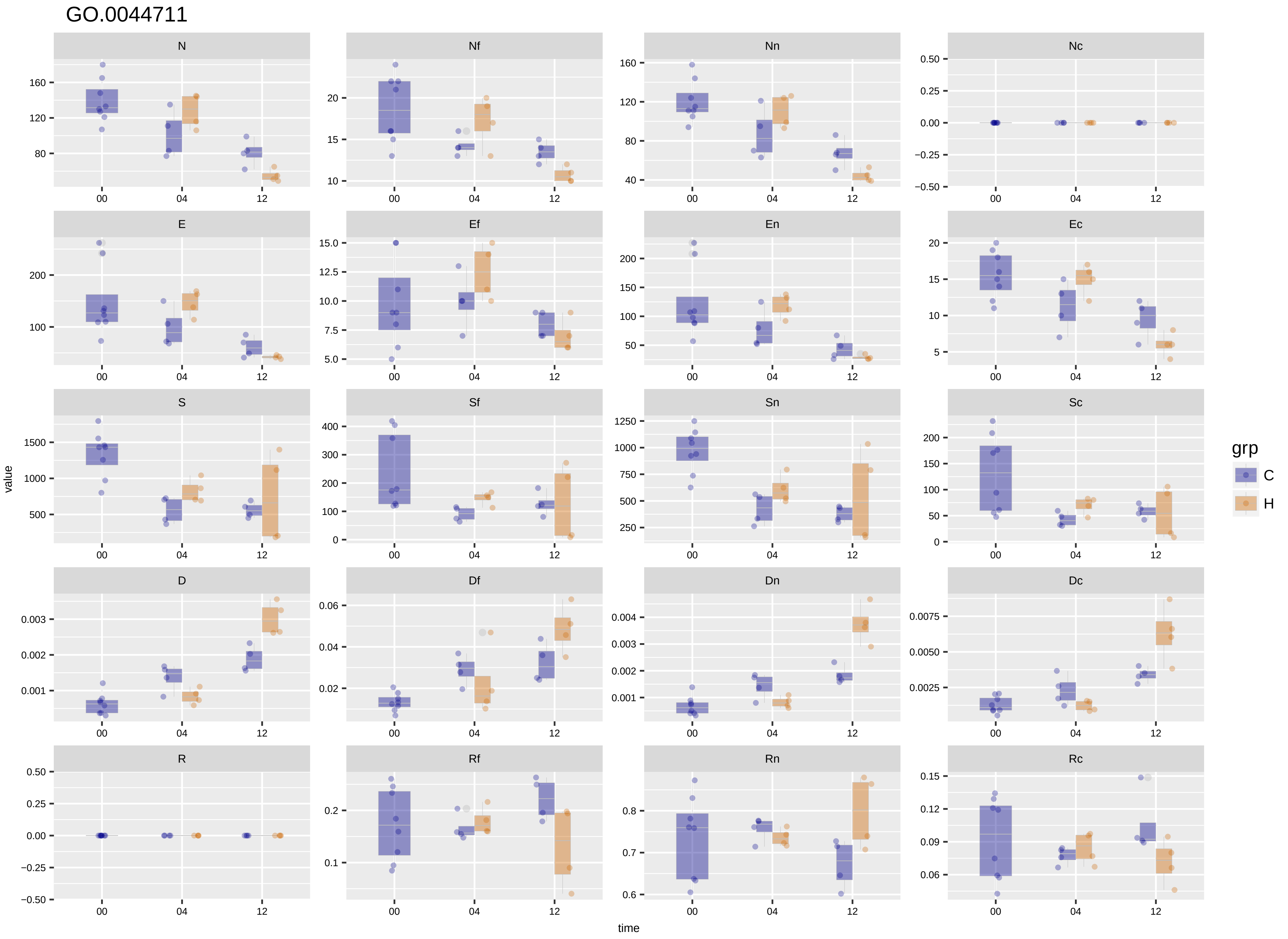
GO.0044711
N
Nf
Nn
Nc
E
Ef
En
Ec
S
Sf
Sn
Sc
D
Df
Dn
Dc
R
Rf
Rn
Rc
value
time
00
04
12
grp
C
H

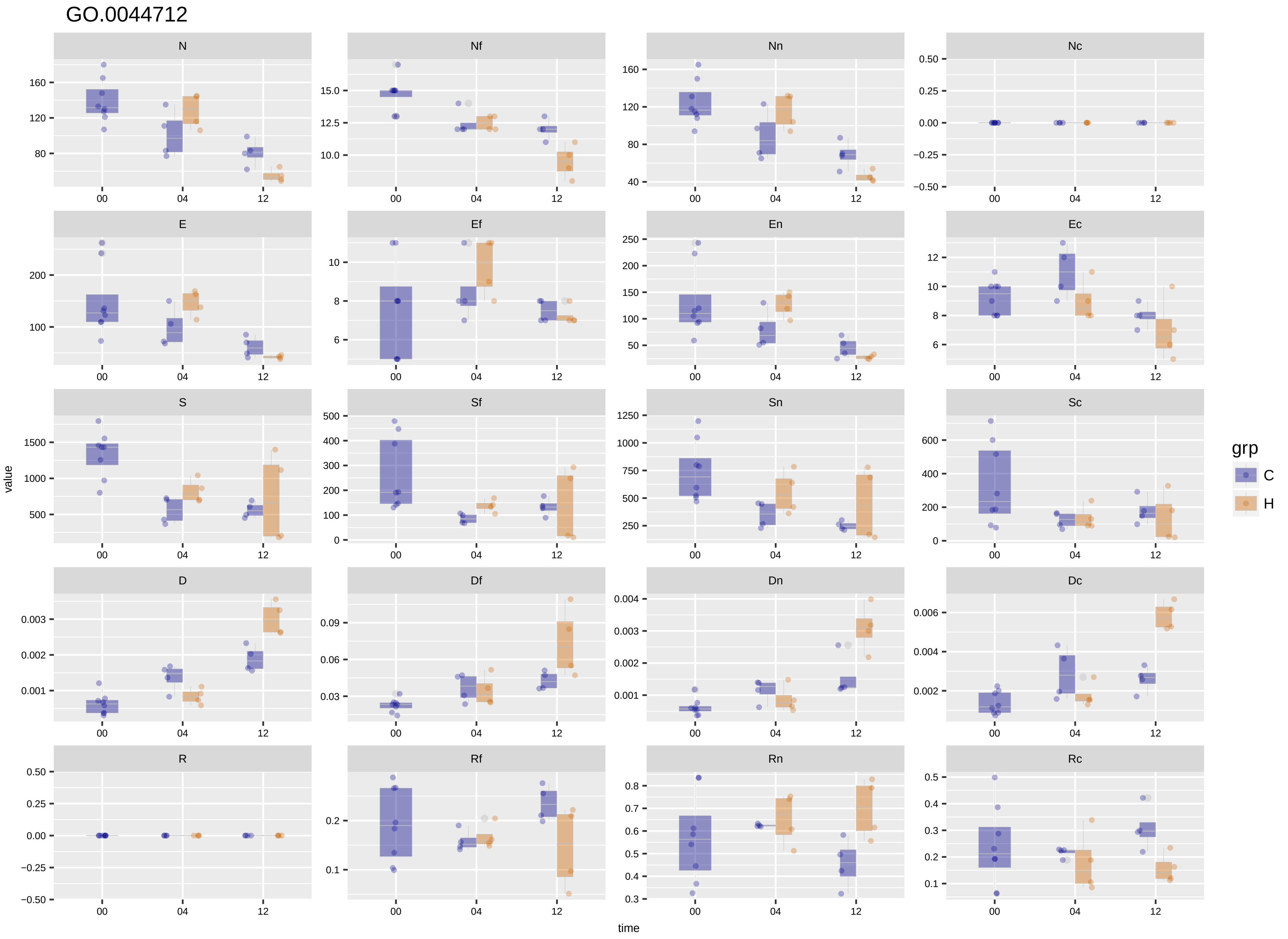

GO.0044712
N
Nf
Nn
Nc
E
Ef
En
Ec
S
Sf
Sn
Sc
D
Df
Dn
Dc
R
Rf
Rn
Rc
value
time
00
04
12
grp
C
H

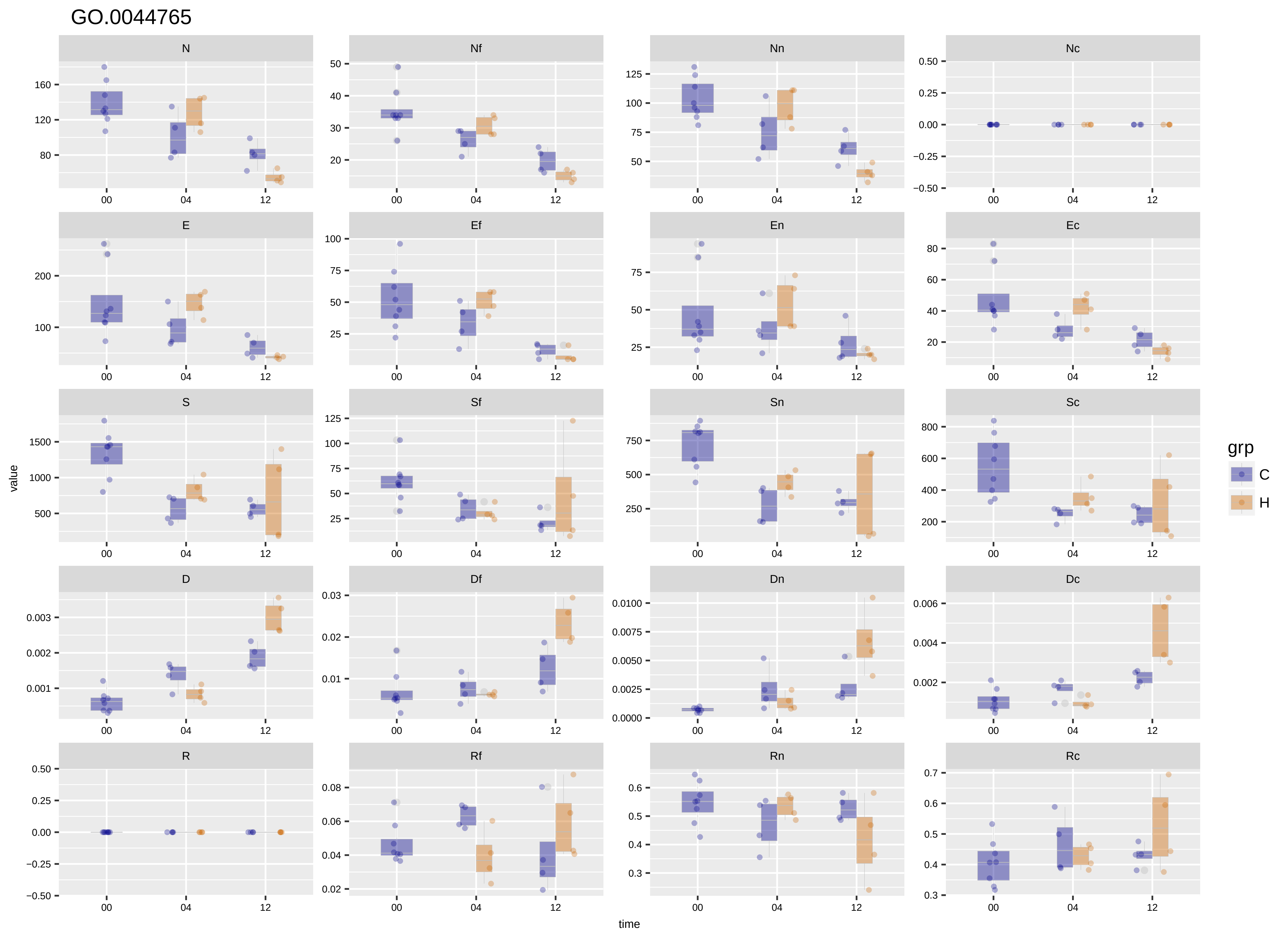

GO.0044765
N
Nf
Nn
Nc
E
Ef
En
Ec
S
Sf
Sn
Sc
D
Df
Dn
Dc
R
Rf
Rn
Rc
value
time
grp
C
H

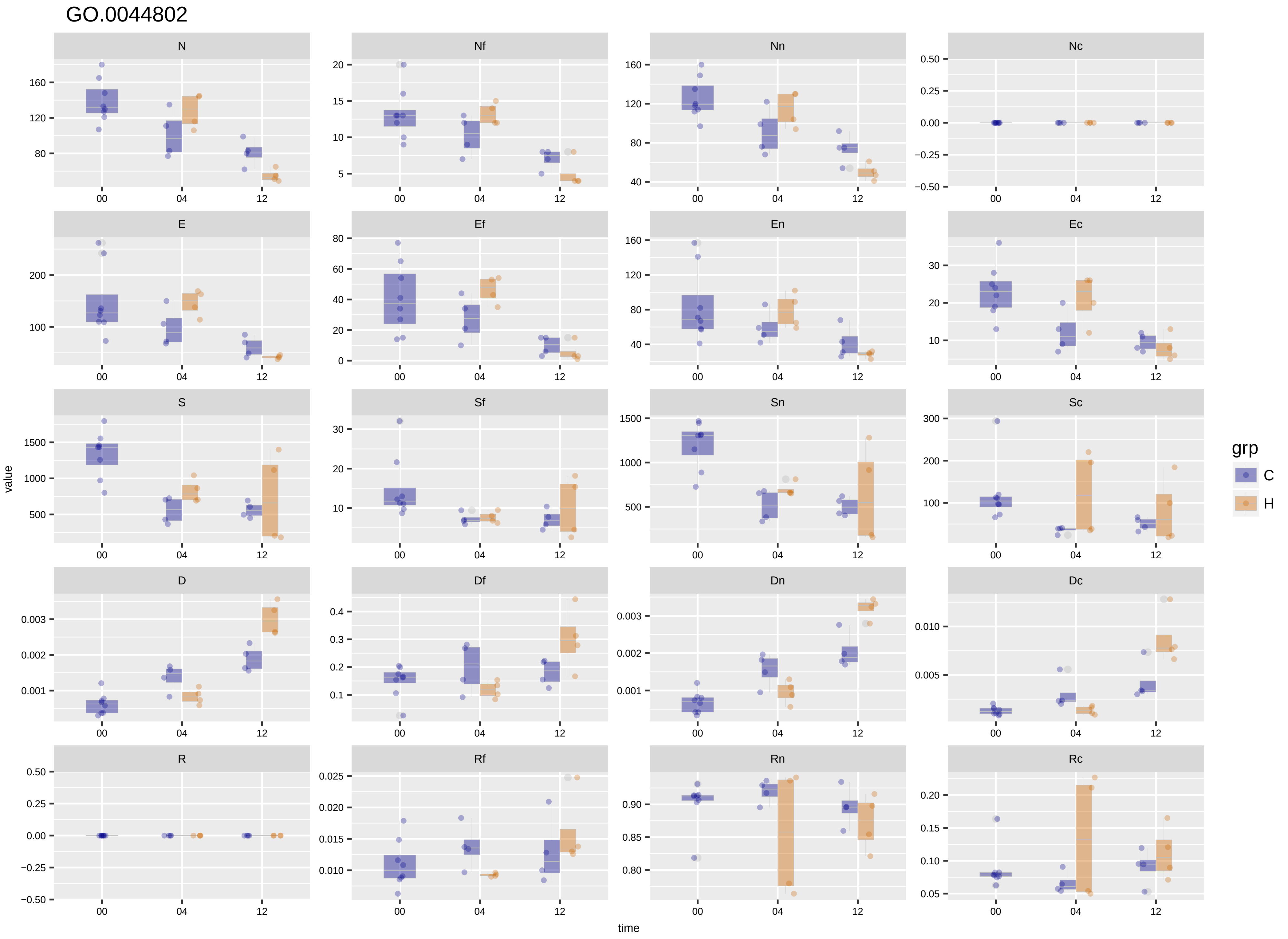

GO.0044802
N
Nf
Nn
Nc
E
Ef
En
Ec
S
Sf
Sn
Sc
D
Df
Dn
Dc
R
Rf
Rn
Rc
value
time
grp
C
H
00
04
12

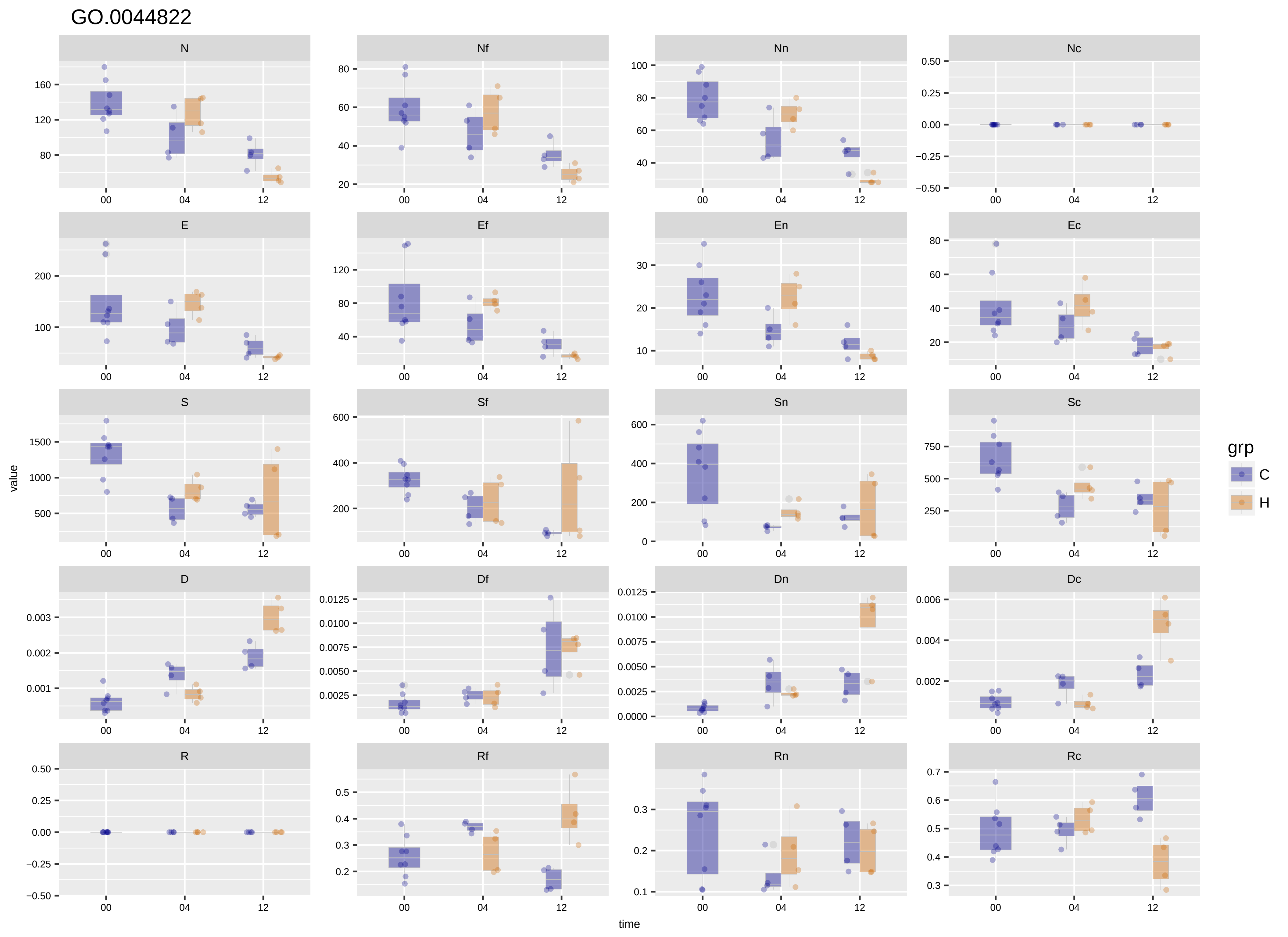
GO.0044822
N
Nf
Nn
Nc
E
Ef
En
Ec
S
Sf
Sn
Sc
D
Df
Dn
Dc
R
Rf
Rn
Rc
value
time
grp
C
H

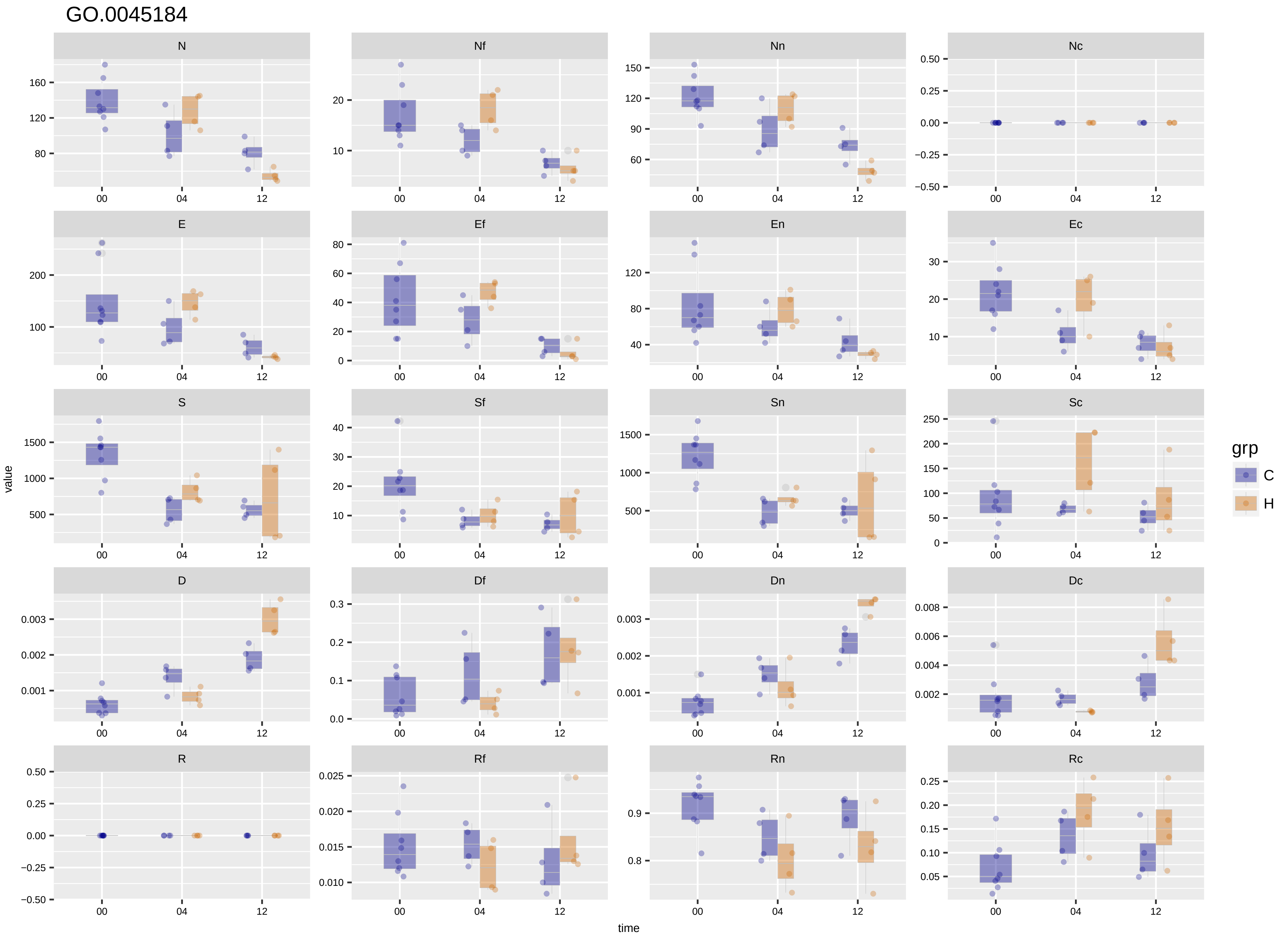
GO.0045184
N
Nf
Nn
Nc
E
Ef
En
Ec
S
Sf
Sn
Sc
D
Df
Dn
Dc
R
Rf
Rn
Rc
value
time
grp
C
H

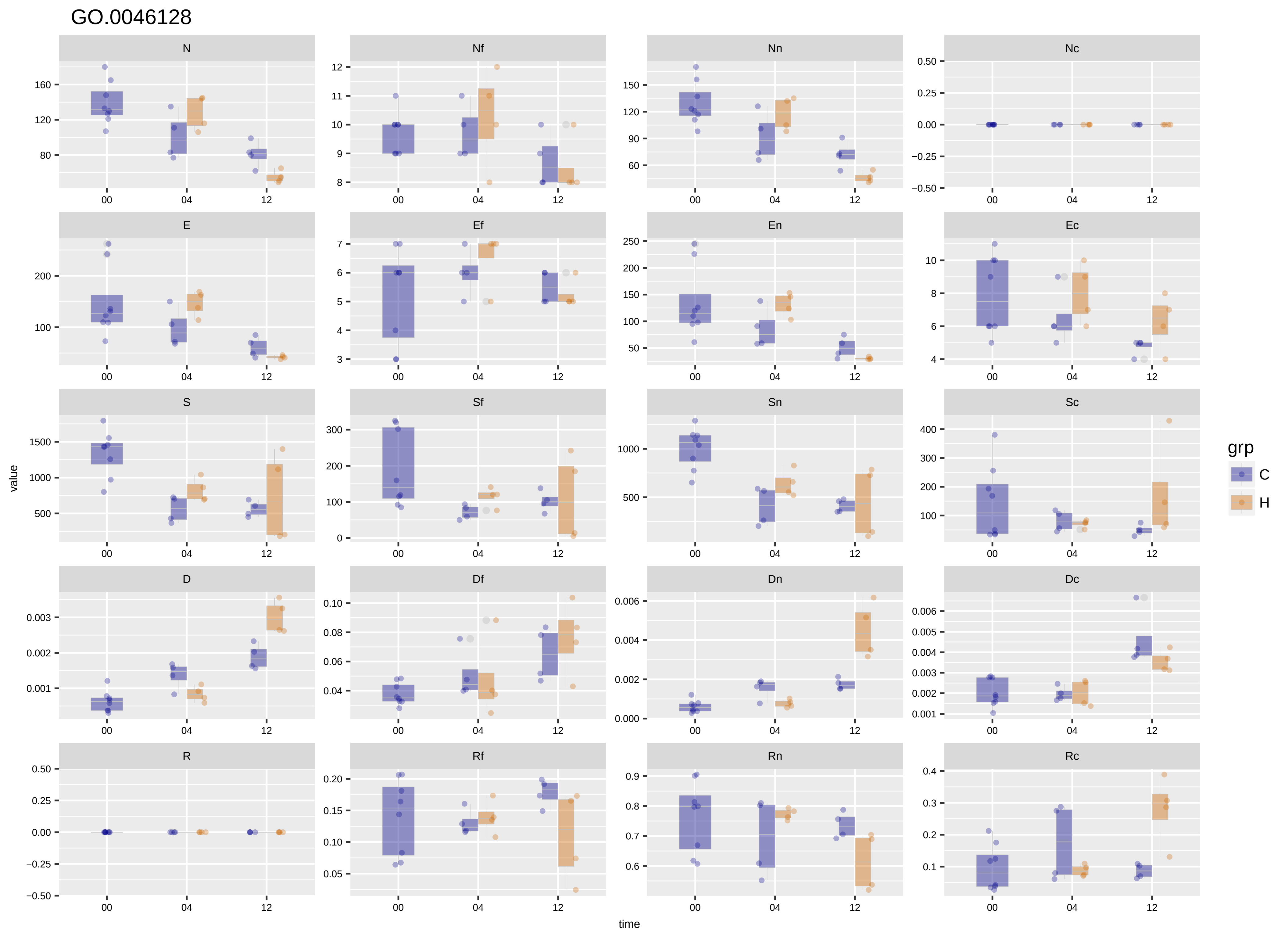
GO.0046128
N
Nf
Nn
Nc
E
Ef
En
Ec
S
Sf
Sn
Sc
D
Df
Dn
Dc
R
Rf
Rn
Rc
00
04
12
value
time
grp
C
H

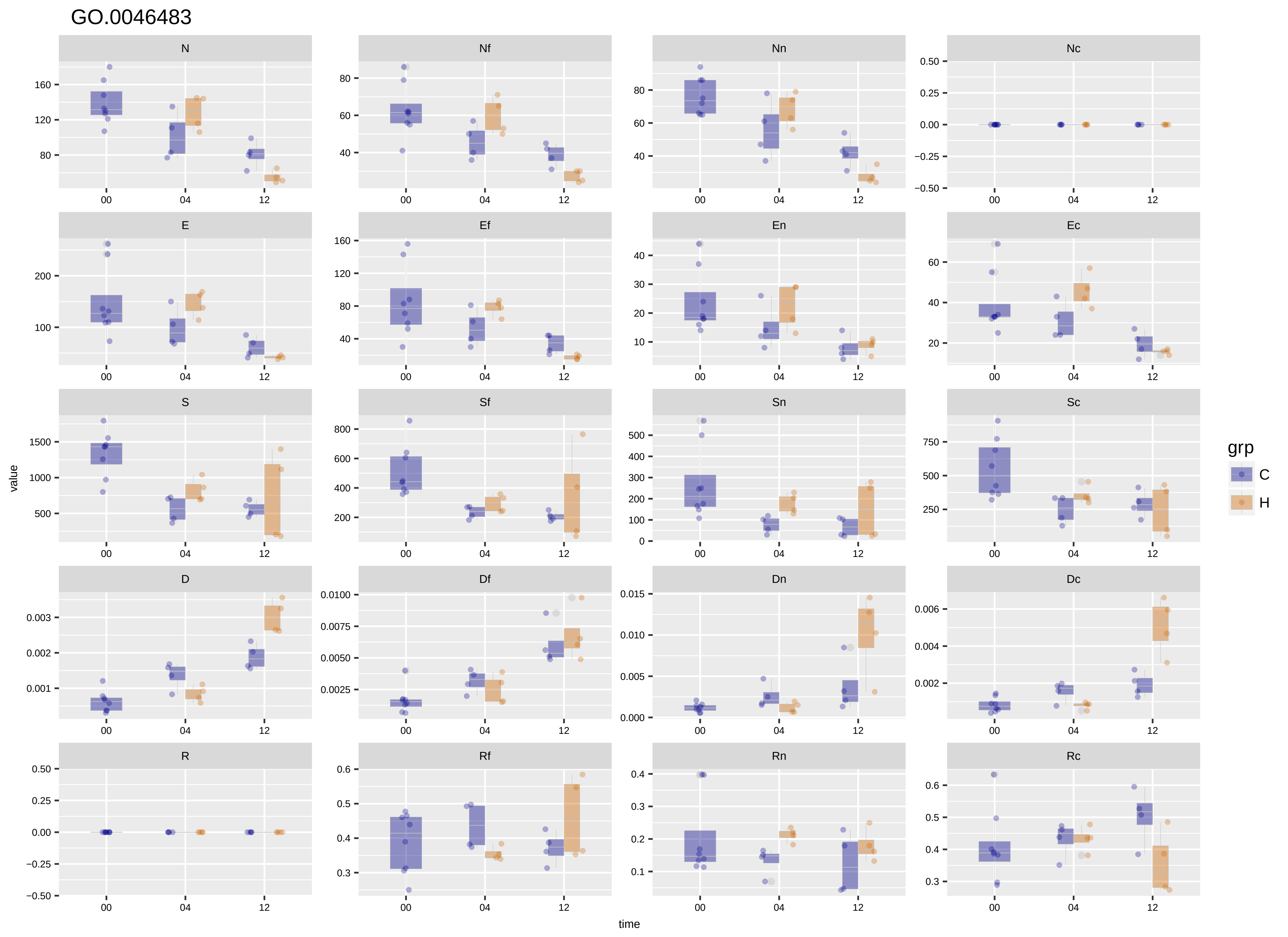

GO.0046483
N
Nf
Nn
Nc
E
Ef
En
Ec
S
Sf
Sn
Sc
D
Df
Dn
Dc
R
Rf
Rn
Rc
value
time
grp
C
H

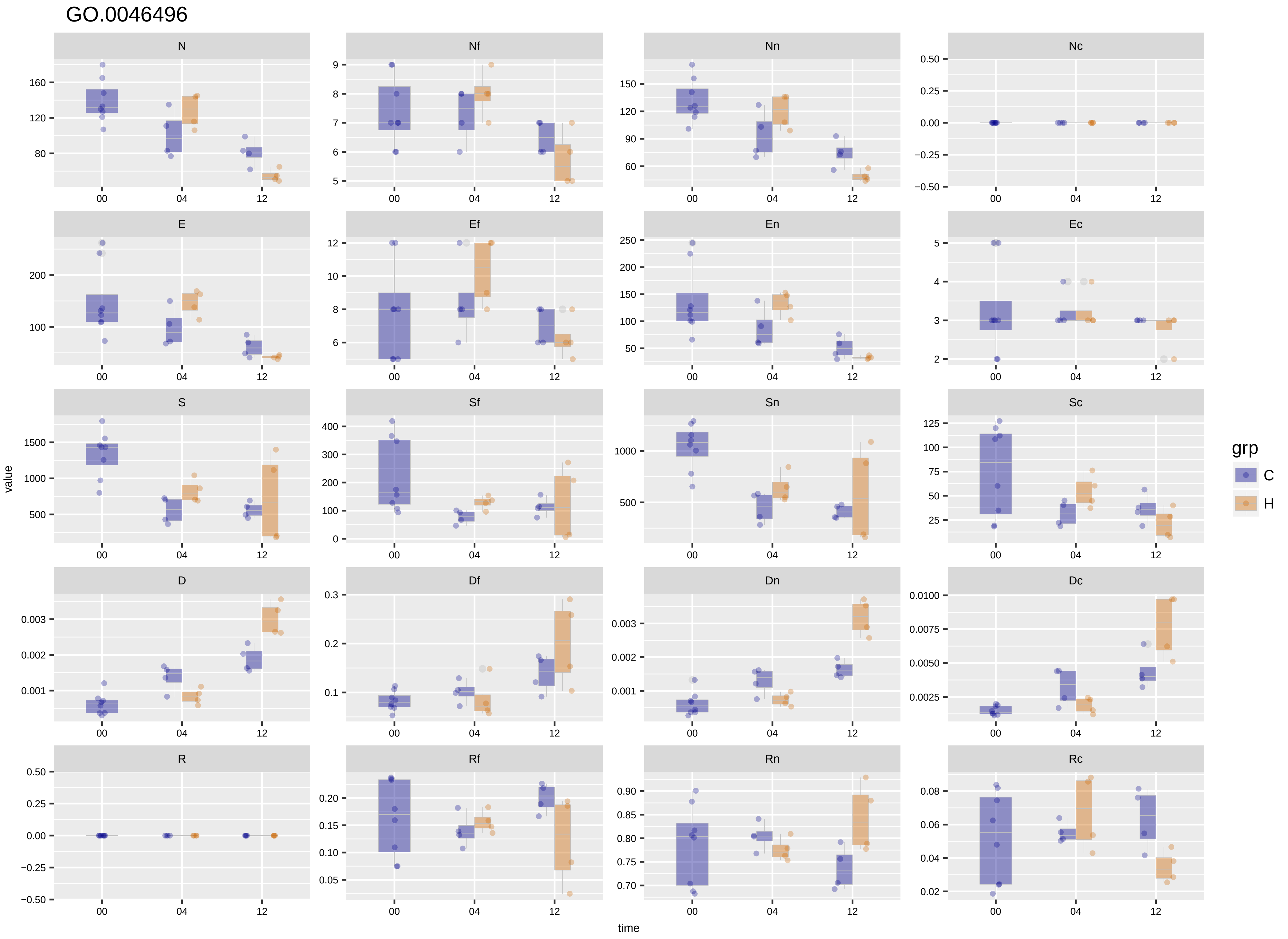
GO.0046496
N
Nf
Nn
Nc
E
Ef
En
Ec
S
Sf
Sn
Sc
D
Df
Dn
Dc
R
Rf
Rn
Rc
value
time
grp
C
H

GO.0046907

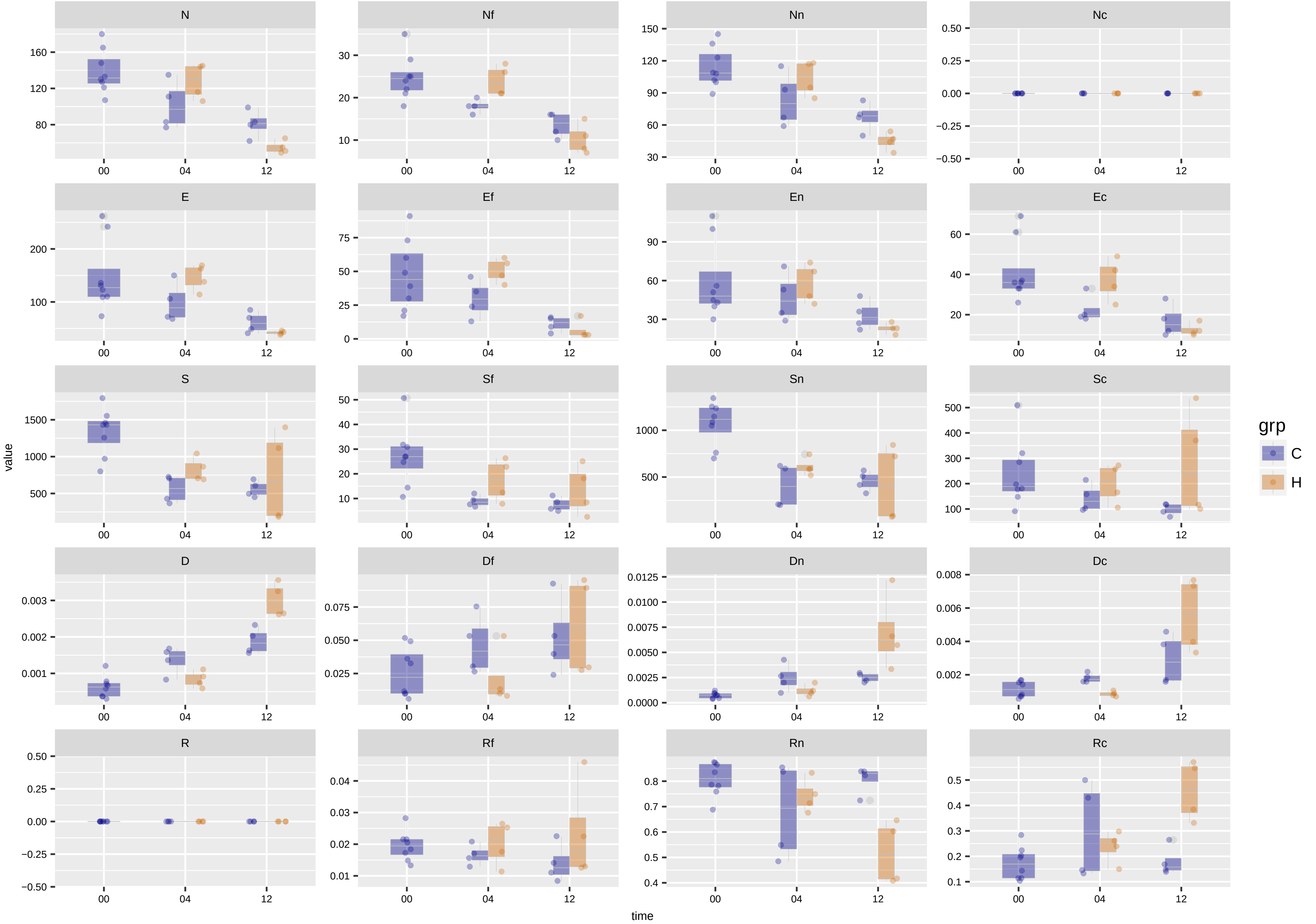

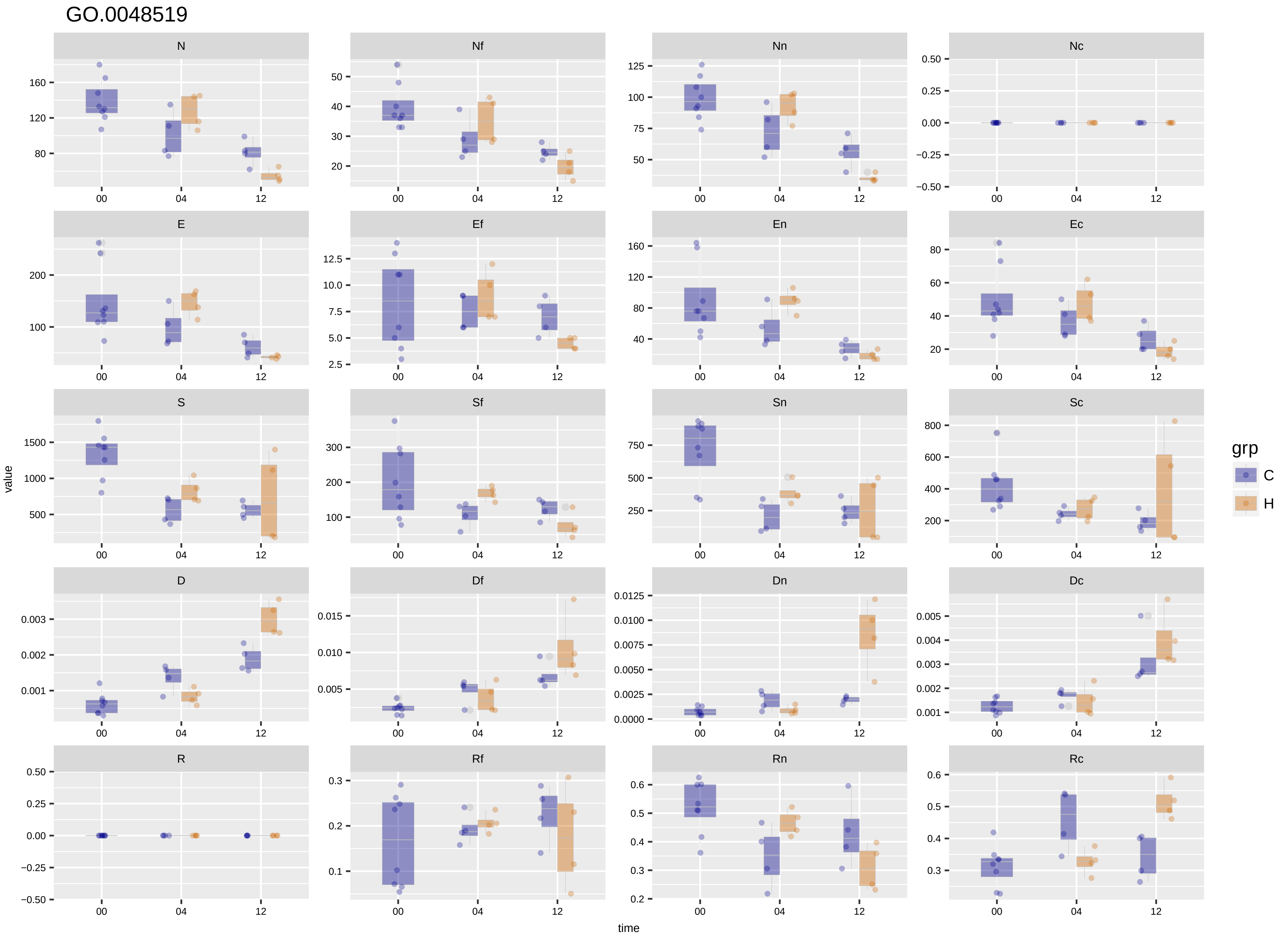
GO.0048519
N
Nf
Nn
Nc
E
Ef
En
Ec
S
Sf
Sn
Sc
D
Df
Dn
Dc
R
Rf
Rn
Rc
value
time
grp
C
H

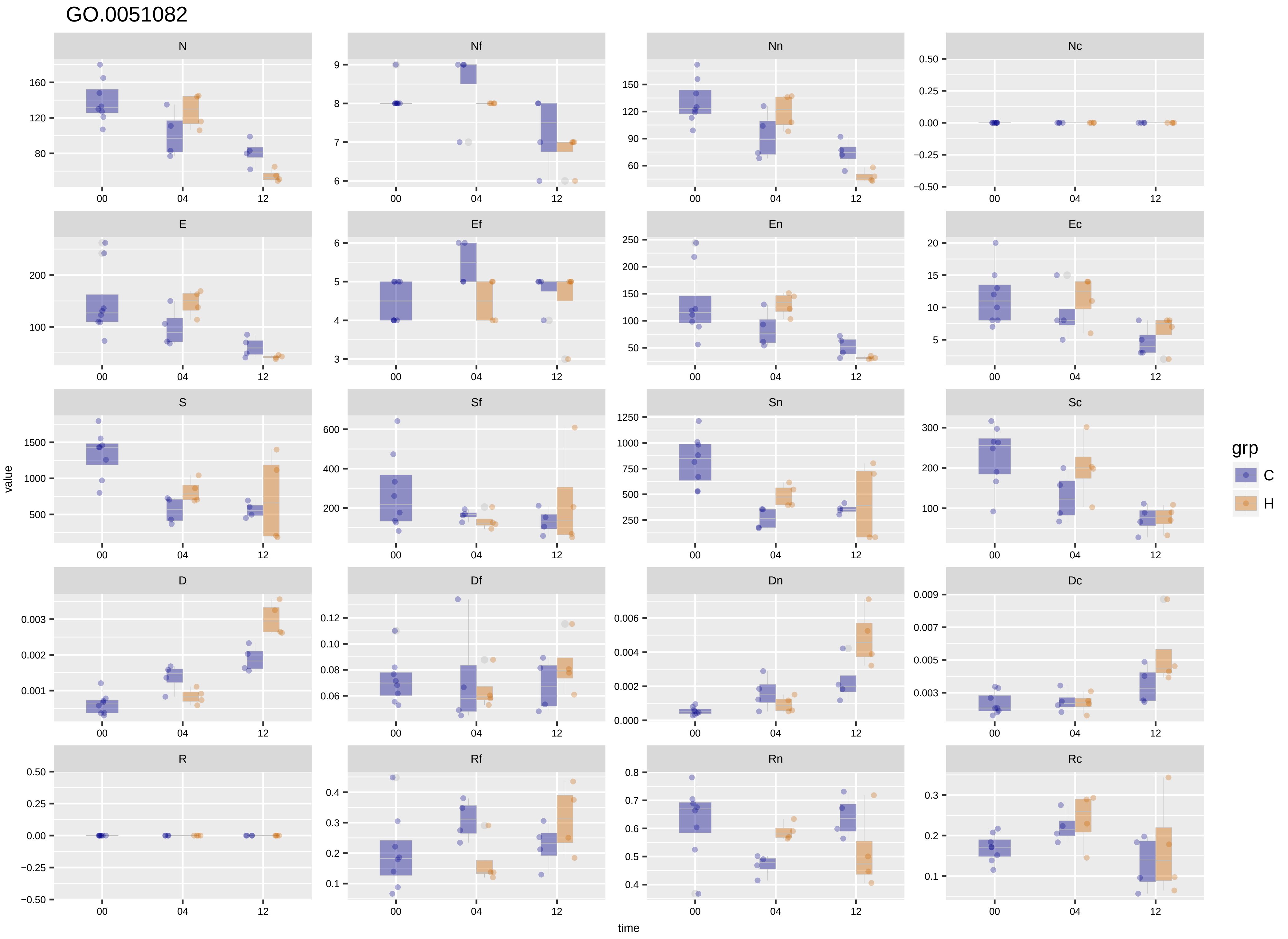
GO.0051082
N
Nf
Nn
Nc
E
Ef
En
Ec
S
Sf
Sn
Sc
D
Df
Dn
Dc
R
Rf
Rn
Rc
00
04
12
value
time
grp
C
H

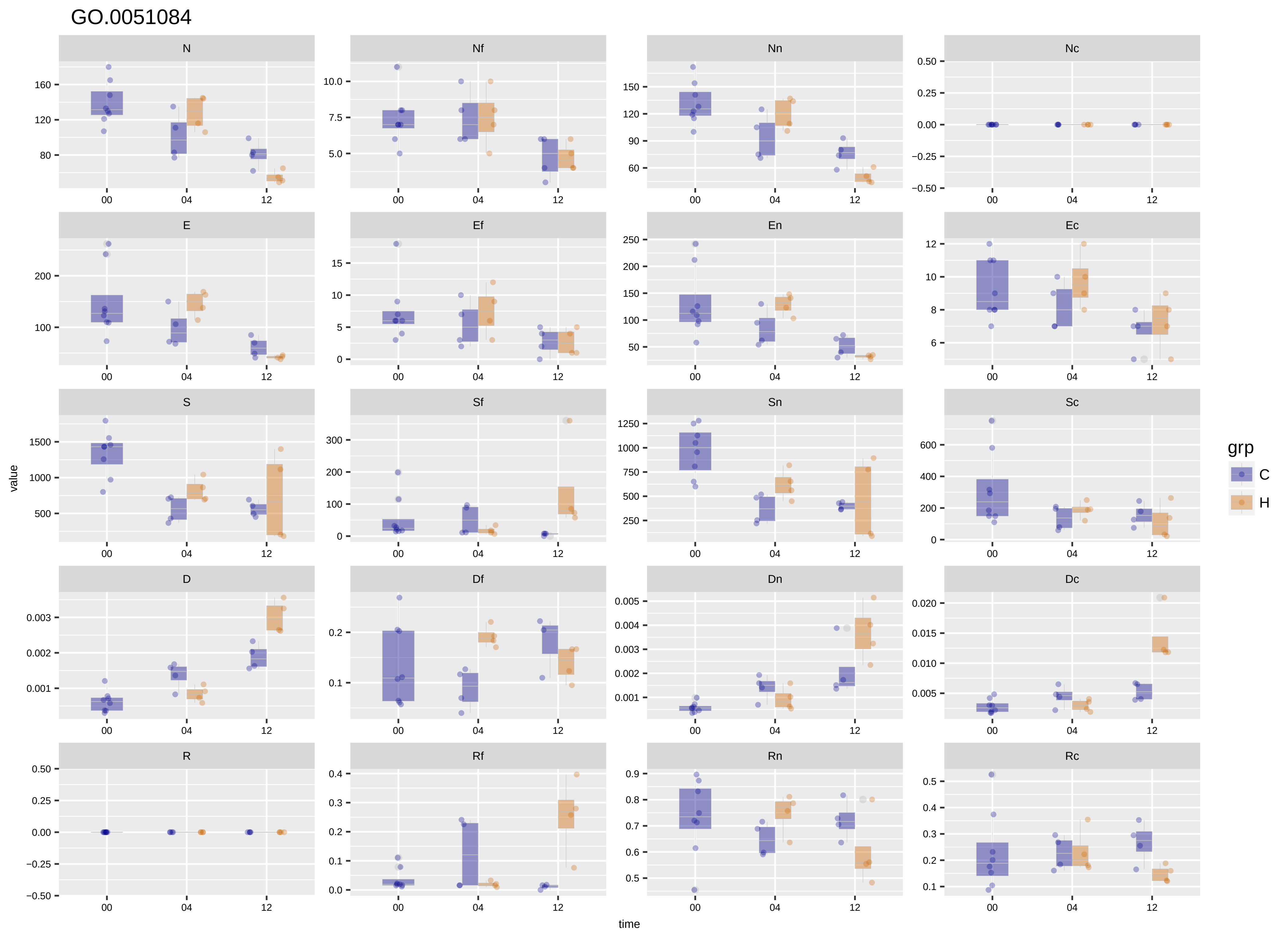

GO.0051084
N
Nf
Nn
Nc
E
Ef
En
Ec
S
Sf
Sn
Sc
D
Df
Dn
Dc
R
Rf
Rn
Rc
value
time
grp
C
H

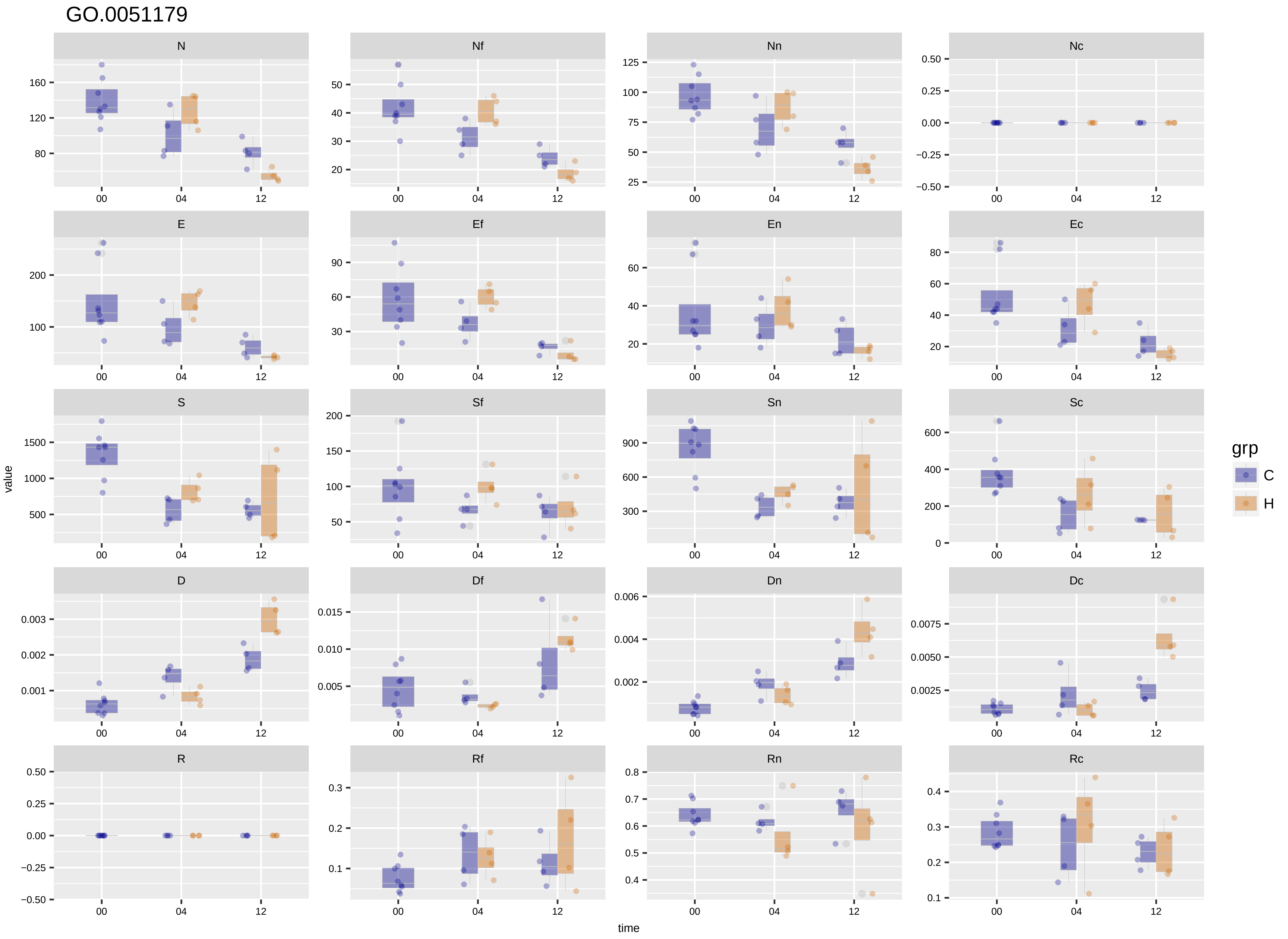

GO.0051179
N
Nf
Nn
Nc
E
Ef
En
Ec
S
Sf
Sn
Sc
D
Df
Dn
Dc
R
Rf
Rn
Rc
value
time
grp
C
H

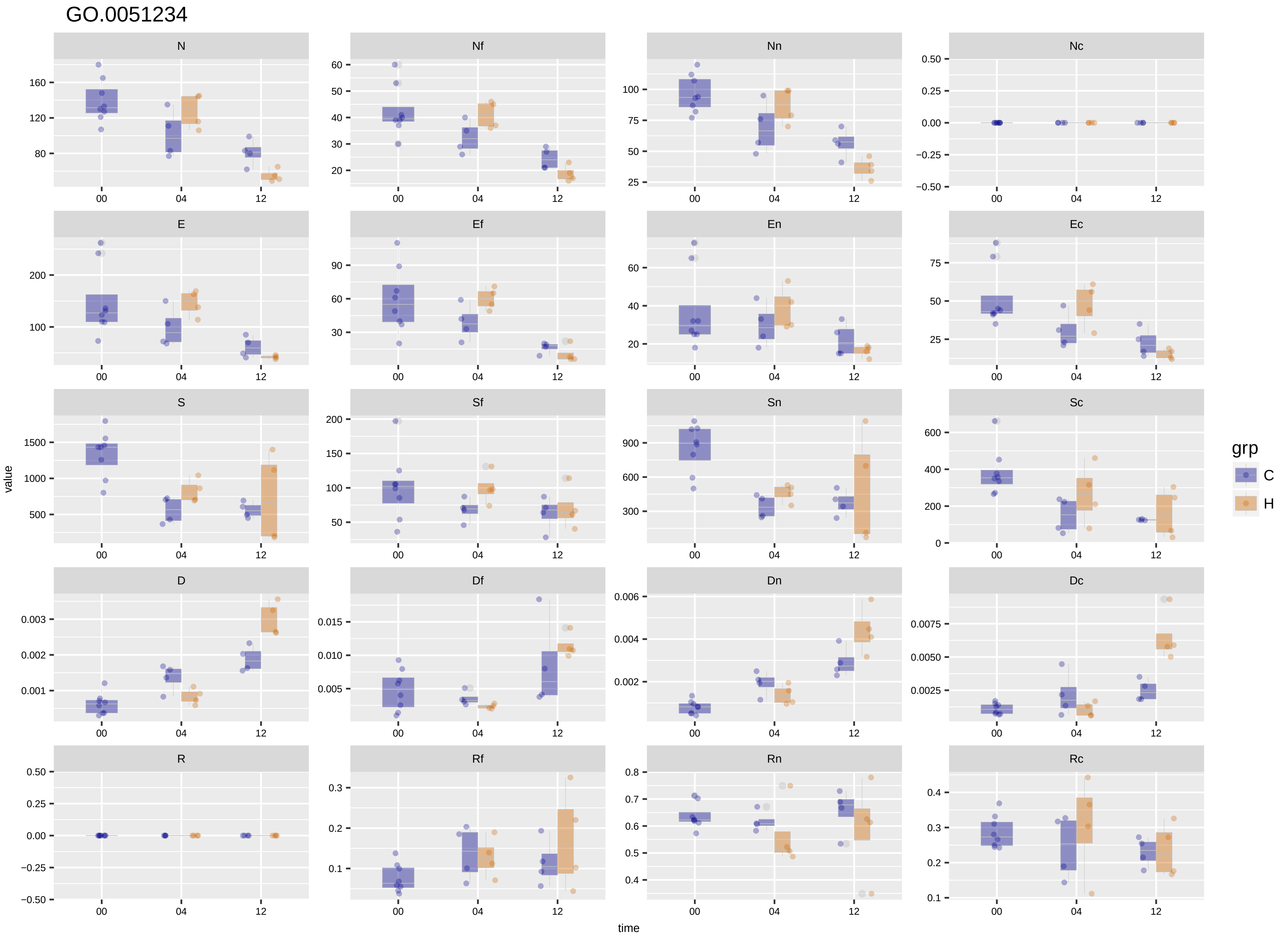

GO.0051234
N
Nf
Nn
Nc
E
Ef
En
Ec
S
Sf
Sn
Sc
D
Df
Dn
Dc
R
Rf
Rn
Rc
value
time
grp
C
H

GO.0051641

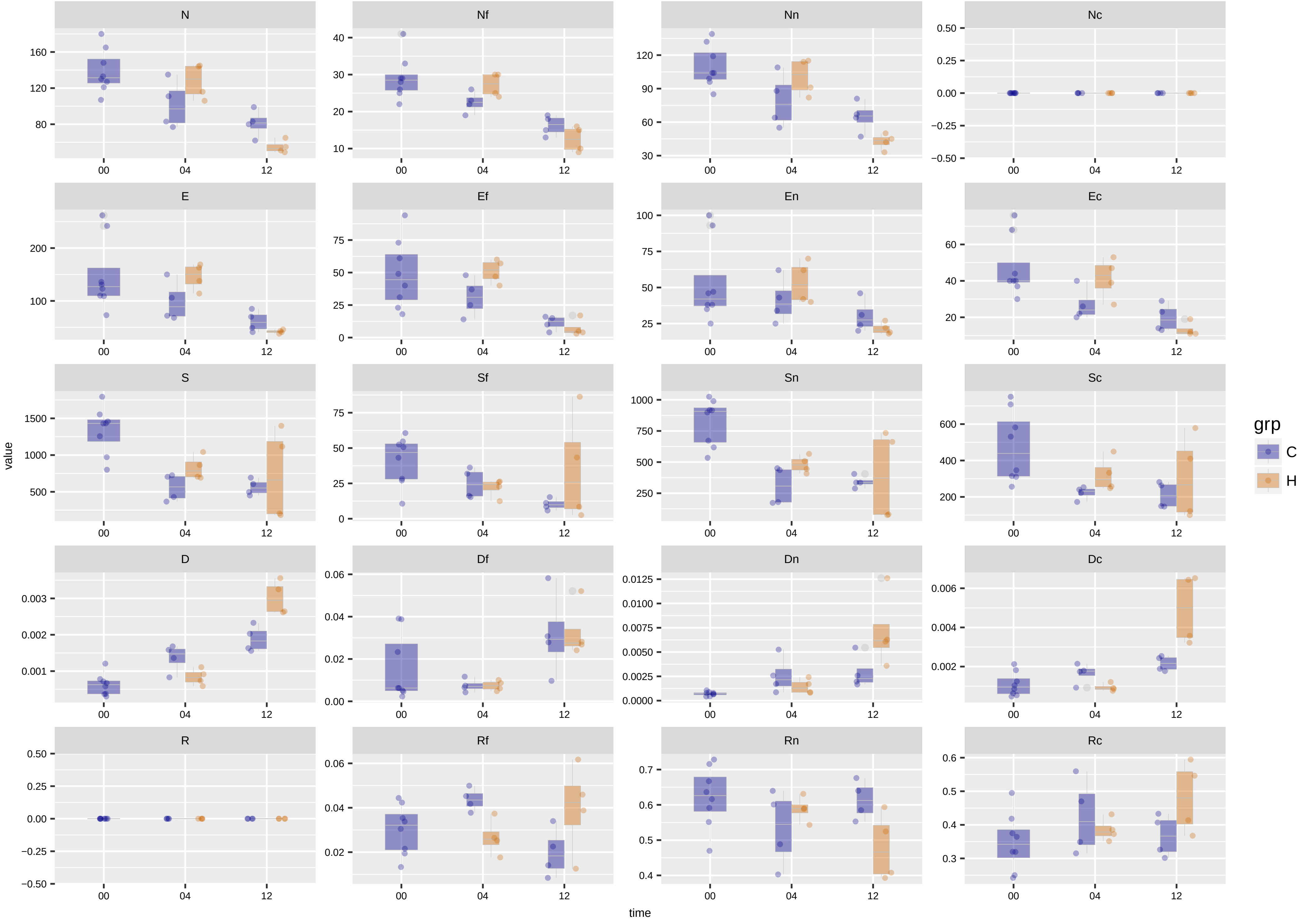

N
Nf
Nn
Nc
E
Ef
En
Ec
S
Sf
Sn
Sc
D
Df
Dn
Dc
R
Rf
Rn
Rc
value
time
grp
C
H
00
04
12

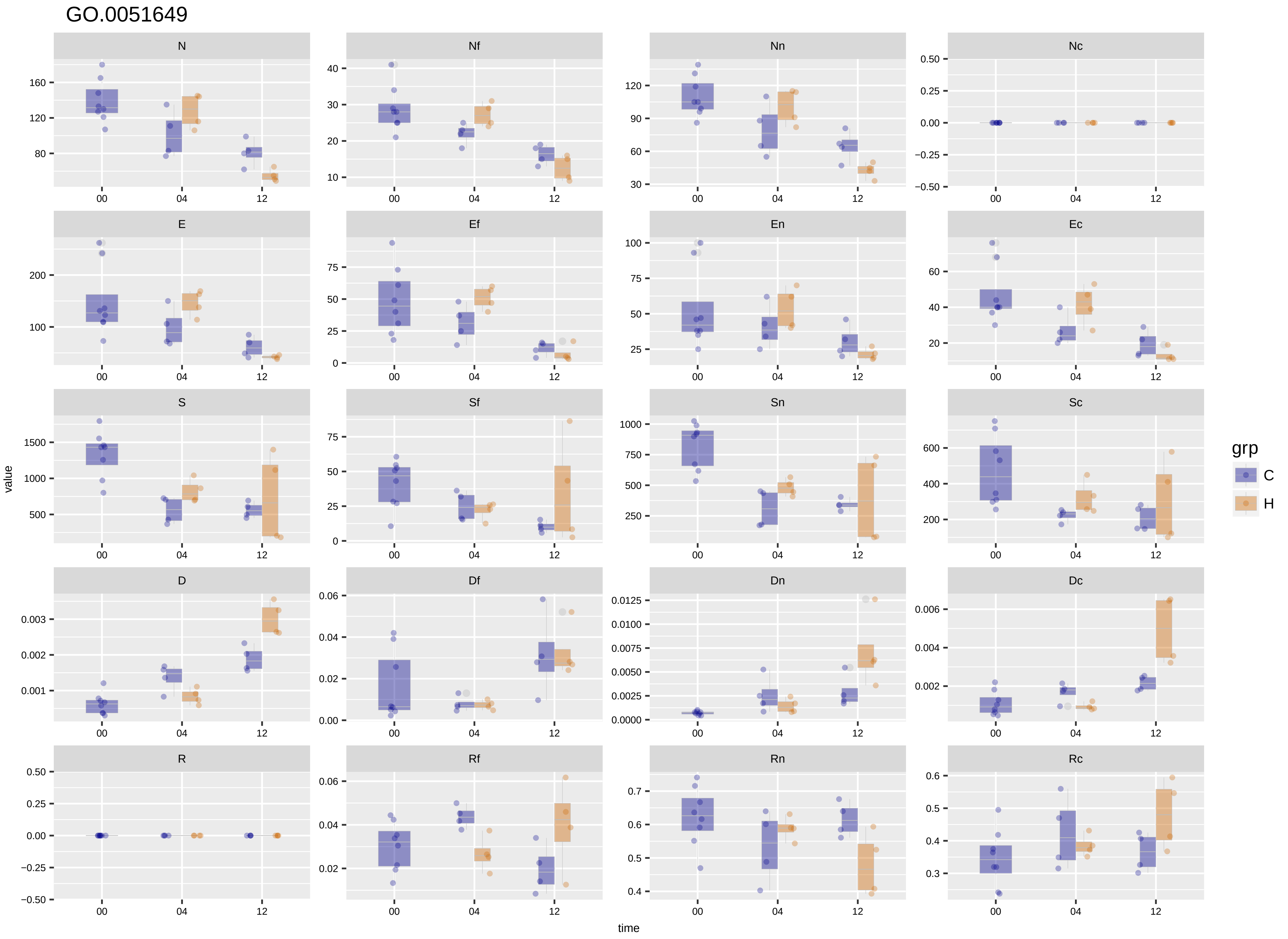
GO.0051649
N
Nf
Nn
Nc
E
Ef
En
Ec
S
Sf
Sn
Sc
D
Df
Dn
Dc
R
Rf
Rn
Rc
value
time
grp
C
H

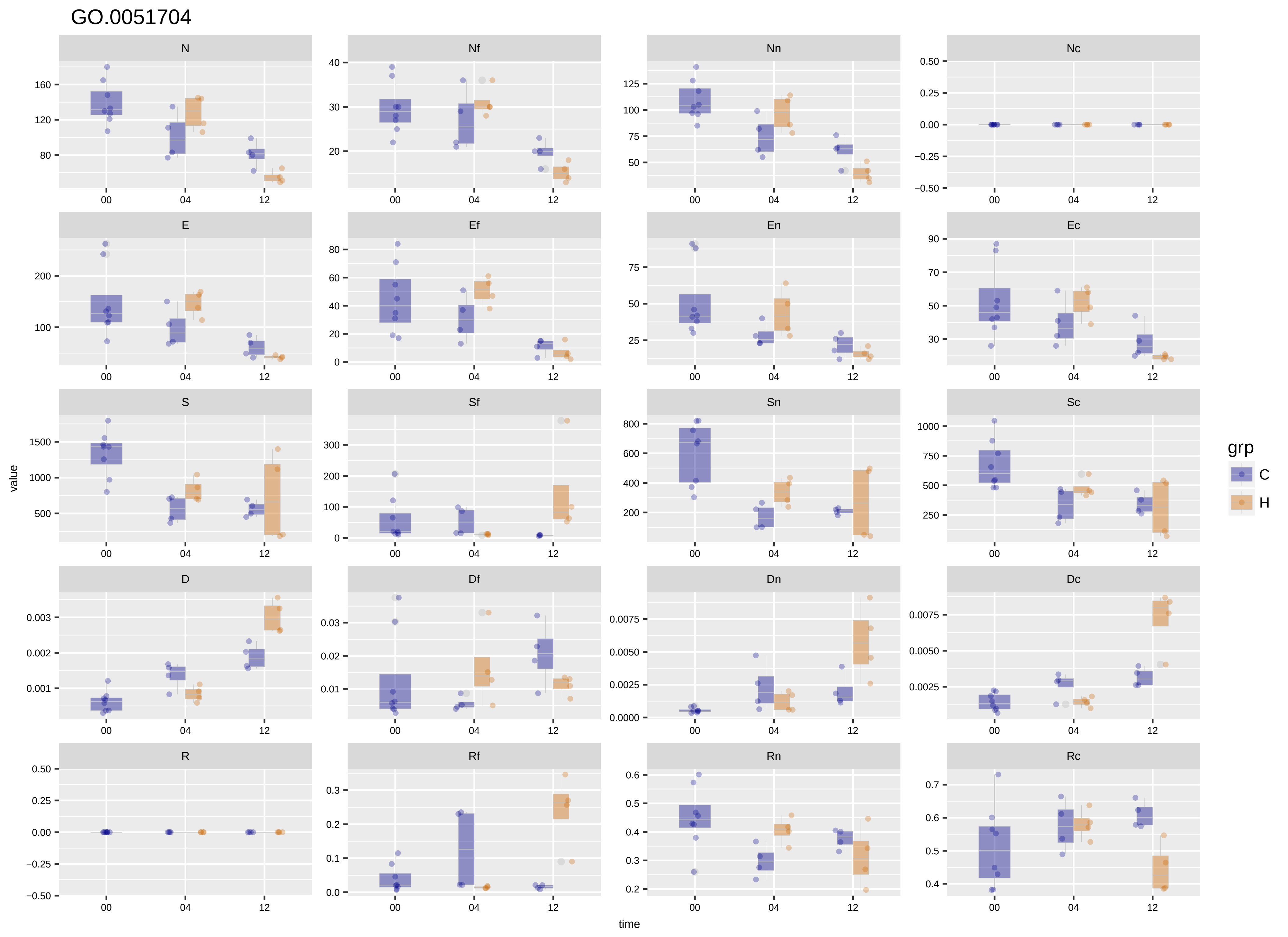

GO.0051704
N
Nf
Nn
Nc
E
Ef
En
Ec
S
Sf
Sn
Sc
D
Df
Dn
Dc
R
Rf
Rn
Rc
value
time
grp
C
H

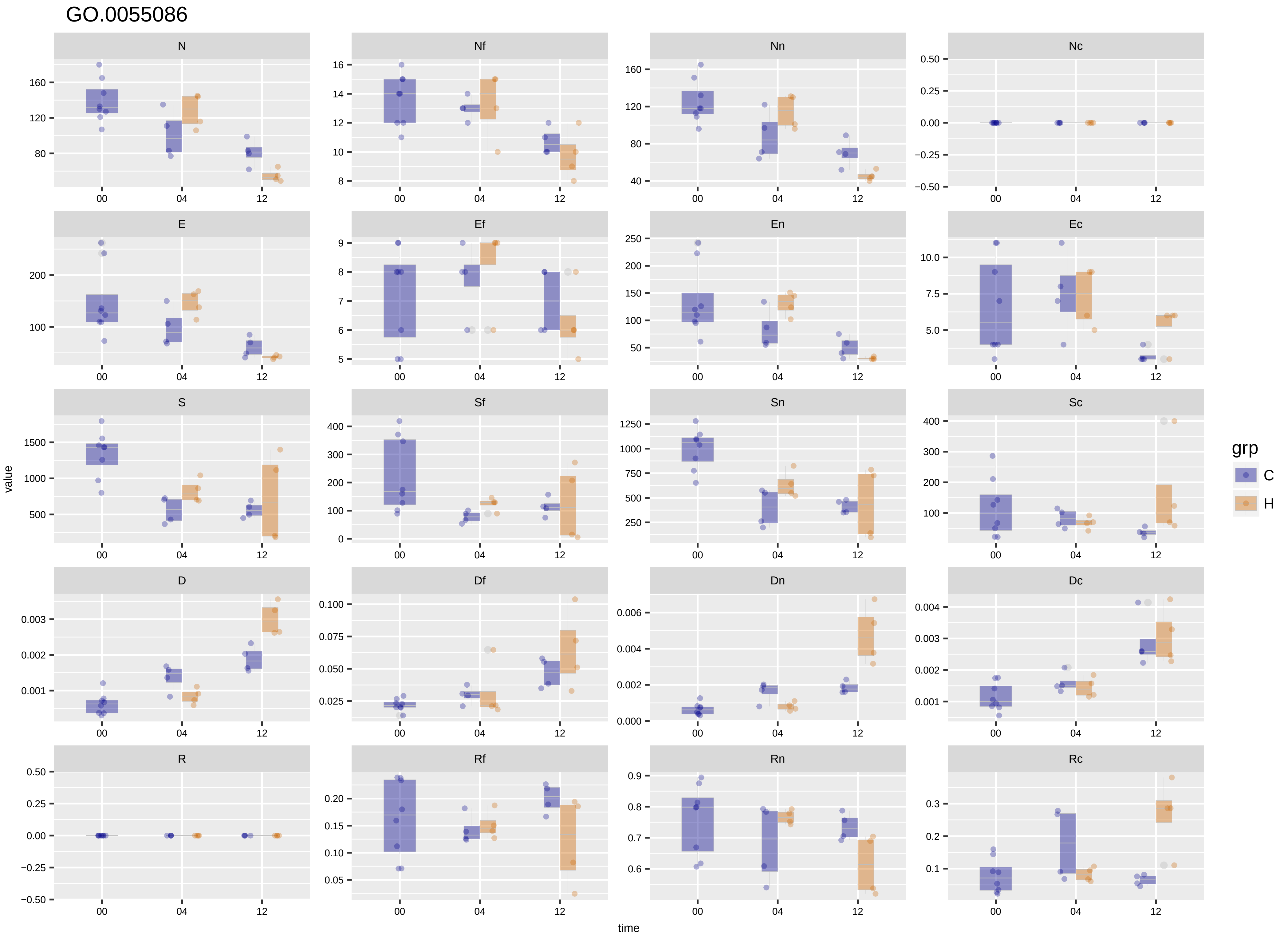

GO.0055086
N
Nf
Nn
Nc
E
Ef
En
Ec
S
Sf
Sn
Sc
D
Df
Dn
Dc
R
Rf
Rn
Rc
00
04
12
value
time
grp
C
H

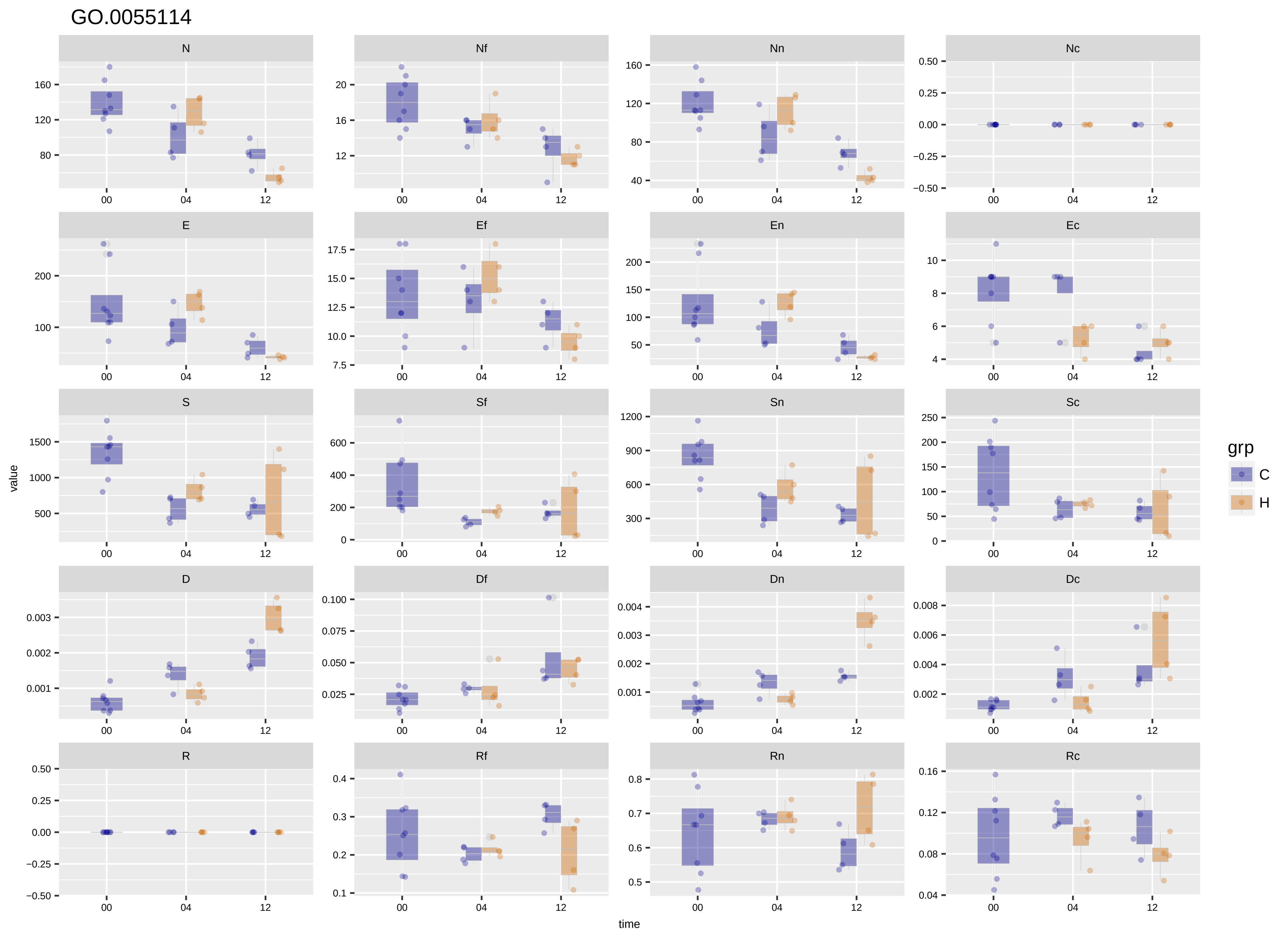
GO.0055114
N
Nf
Nn
Nc
E
Ef
En
Ec
S
Sf
Sn
Sc
D
Df
Dn
Dc
R
Rf
Rn
Rc
value
time
00
04
12
grp
C
H

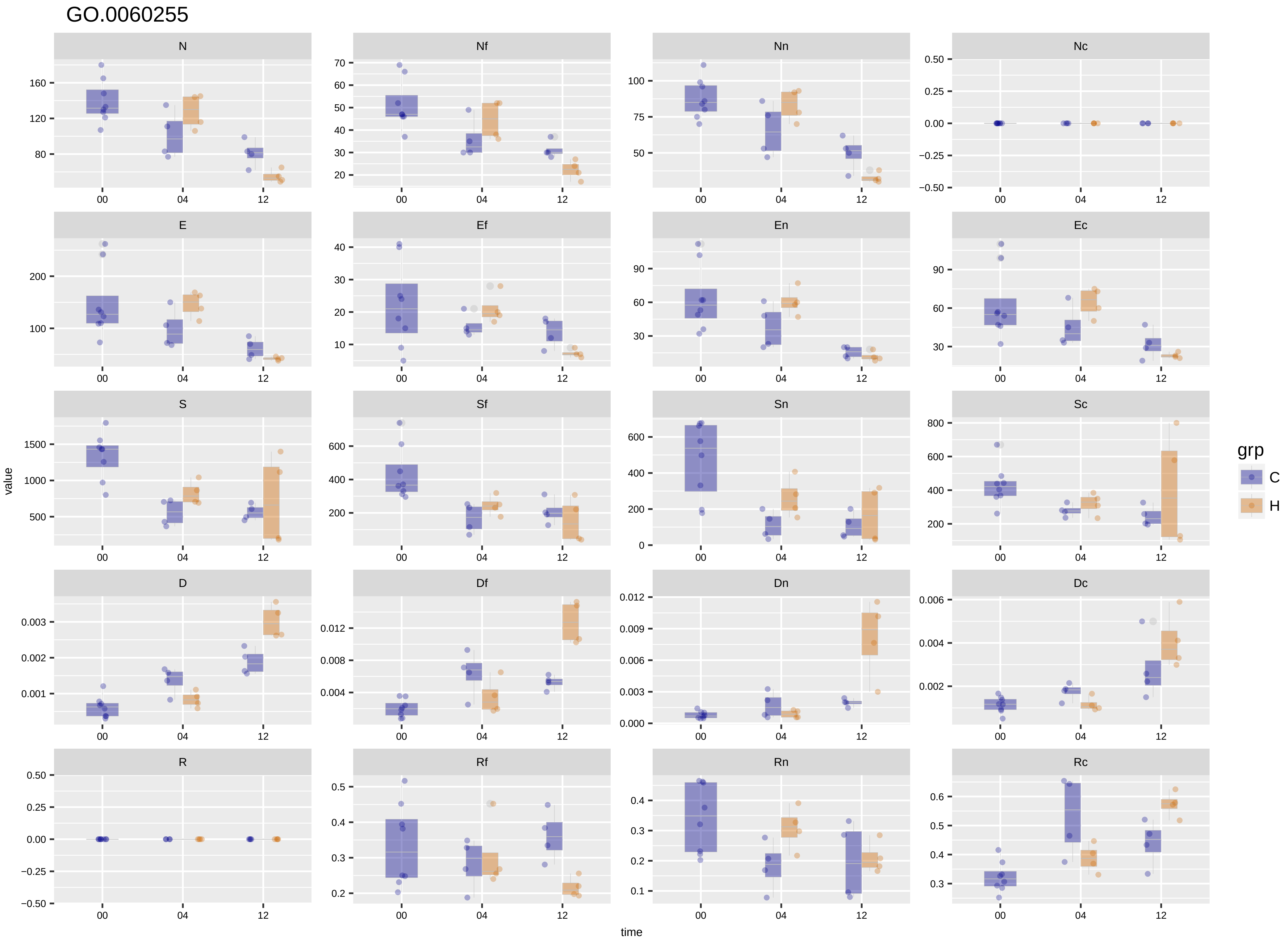
GO.0060255
N
Nf
Nn
Nc
E
Ef
En
Ec
S
Sf
Sn
Sc
D
Df
Dn
Dc
R
Rf
Rn
Rc
value
time
grp
C
H
00
04
12

GO.0060548

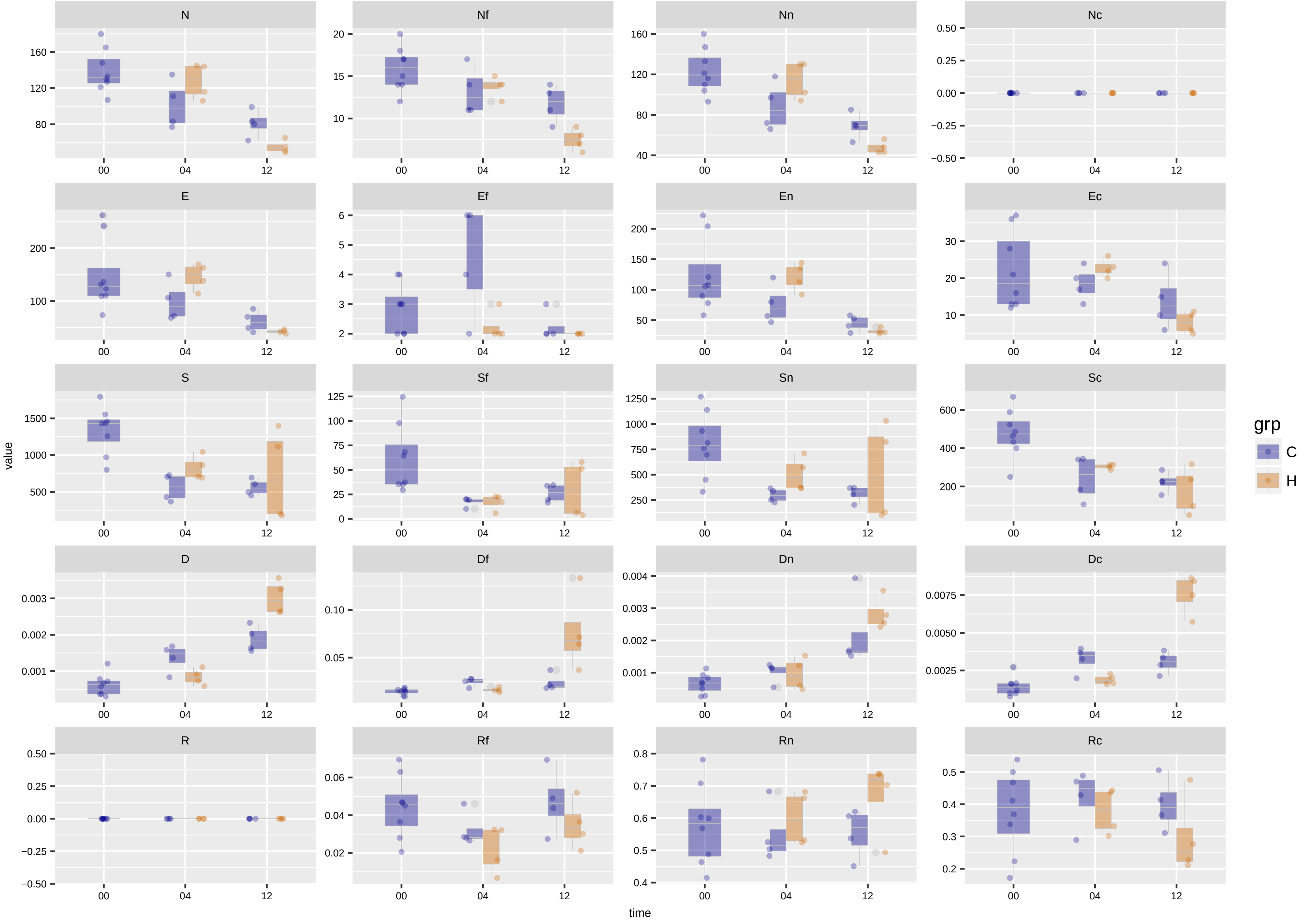

N
Nf
Nn
Nc
E
Ef
En
Ec
S
Sf
Sn
Sc
D
Df
Dn
Dc
R
Rf
Rn
Rc
value
time
00
04
12
grp
C
H

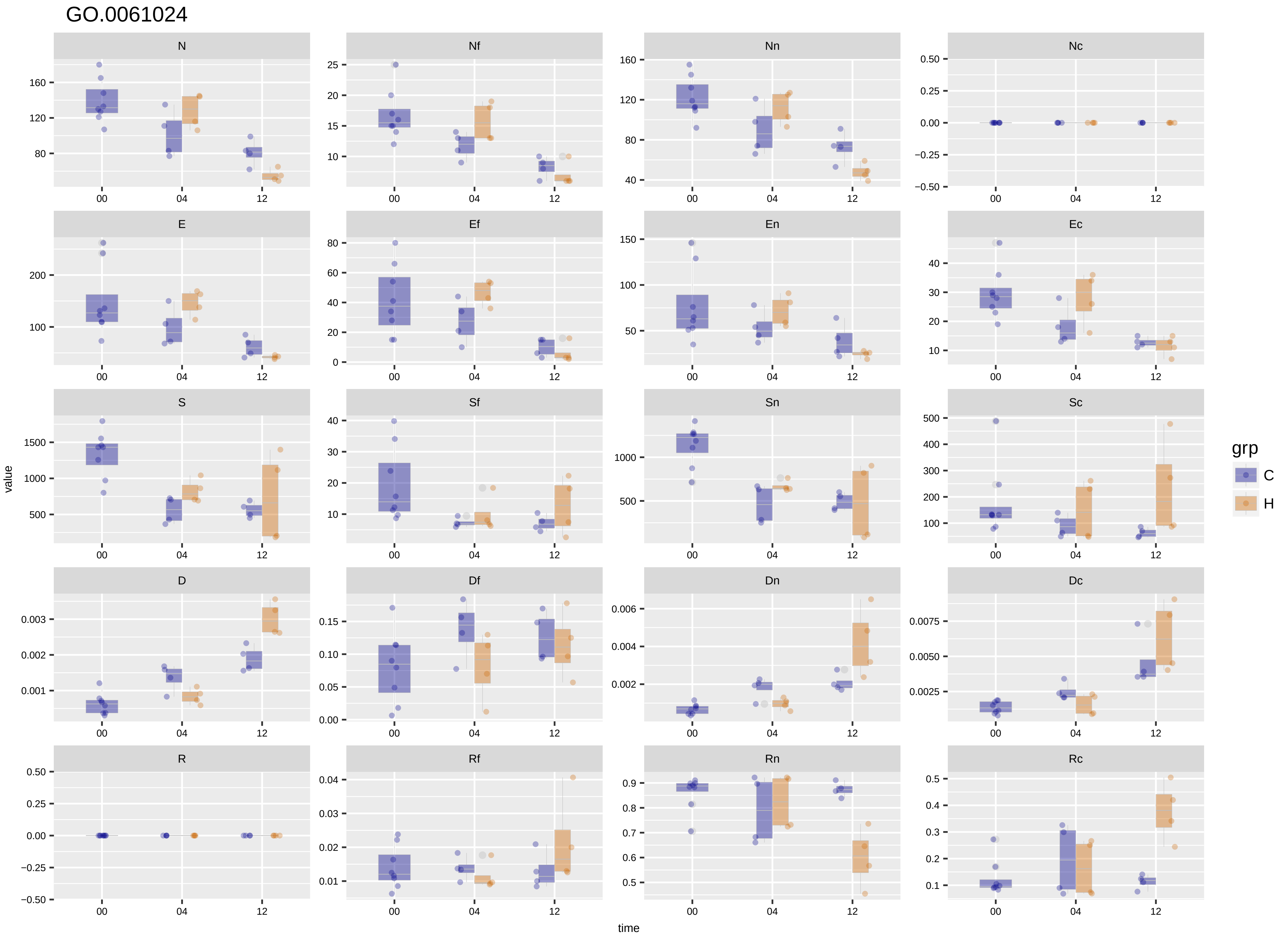

GO.0061024
N
Nf
Nn
Nc
E
Ef
En
Ec
S
Sf
Sn
Sc
D
Df
Dn
Dc
R
Rf
Rn
Rc
value
time
grp
C
H

GO.0065003

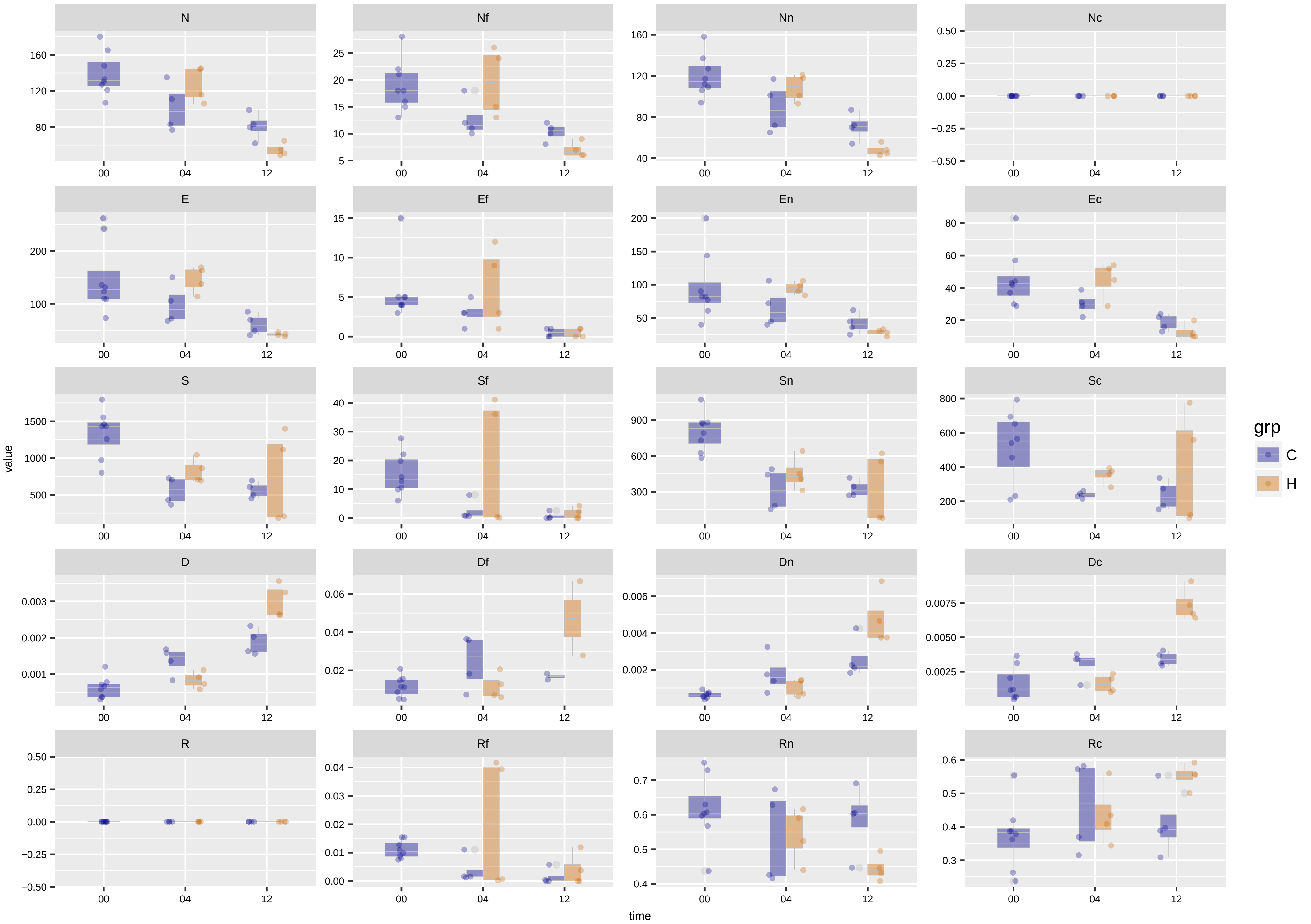

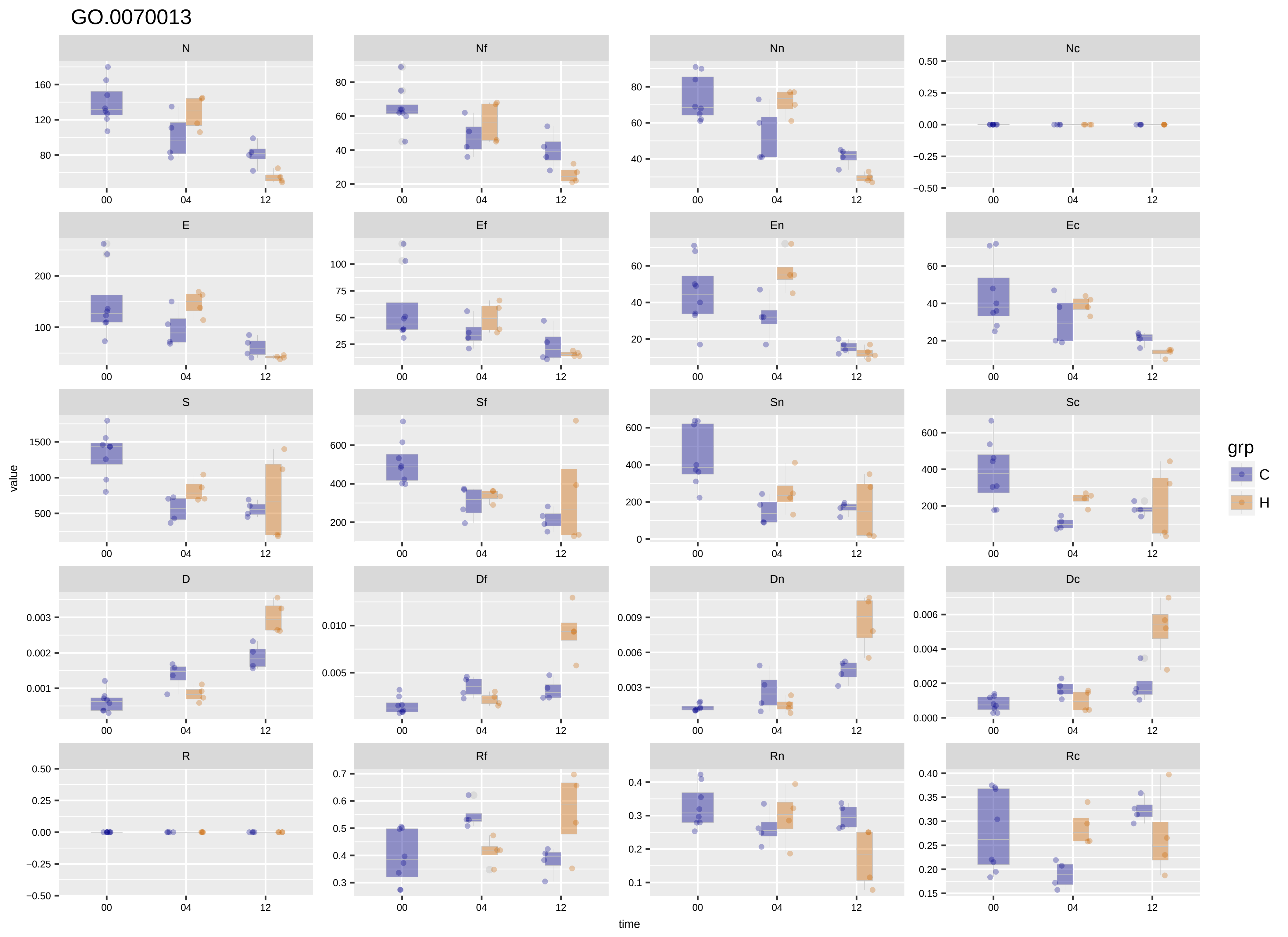

GO.0070013
N
Nf
Nn
Nc
E
Ef
En
Ec
S
Sf
Sn
Sc
D
Df
Dn
Dc
R
Rf
Rn
Rc
value
time
grp
C
H

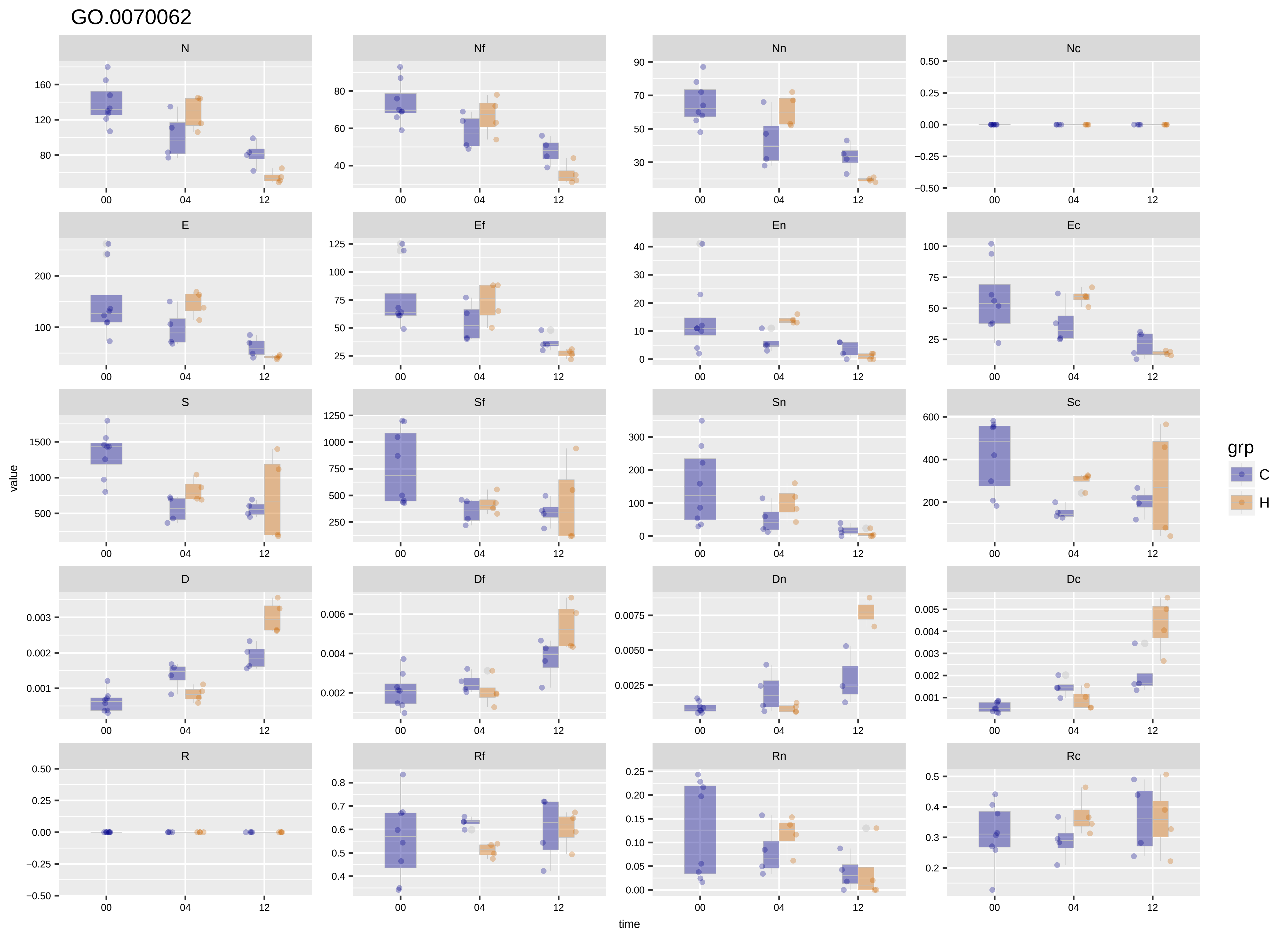
GO.0070062
N
Nf
Nn
Nc
E
Ef
En
Ec
S
Sf
Sn
Sc
D
Df
Dn
Dc
R
Rf
Rn
Rc
value
time
grp
C
H

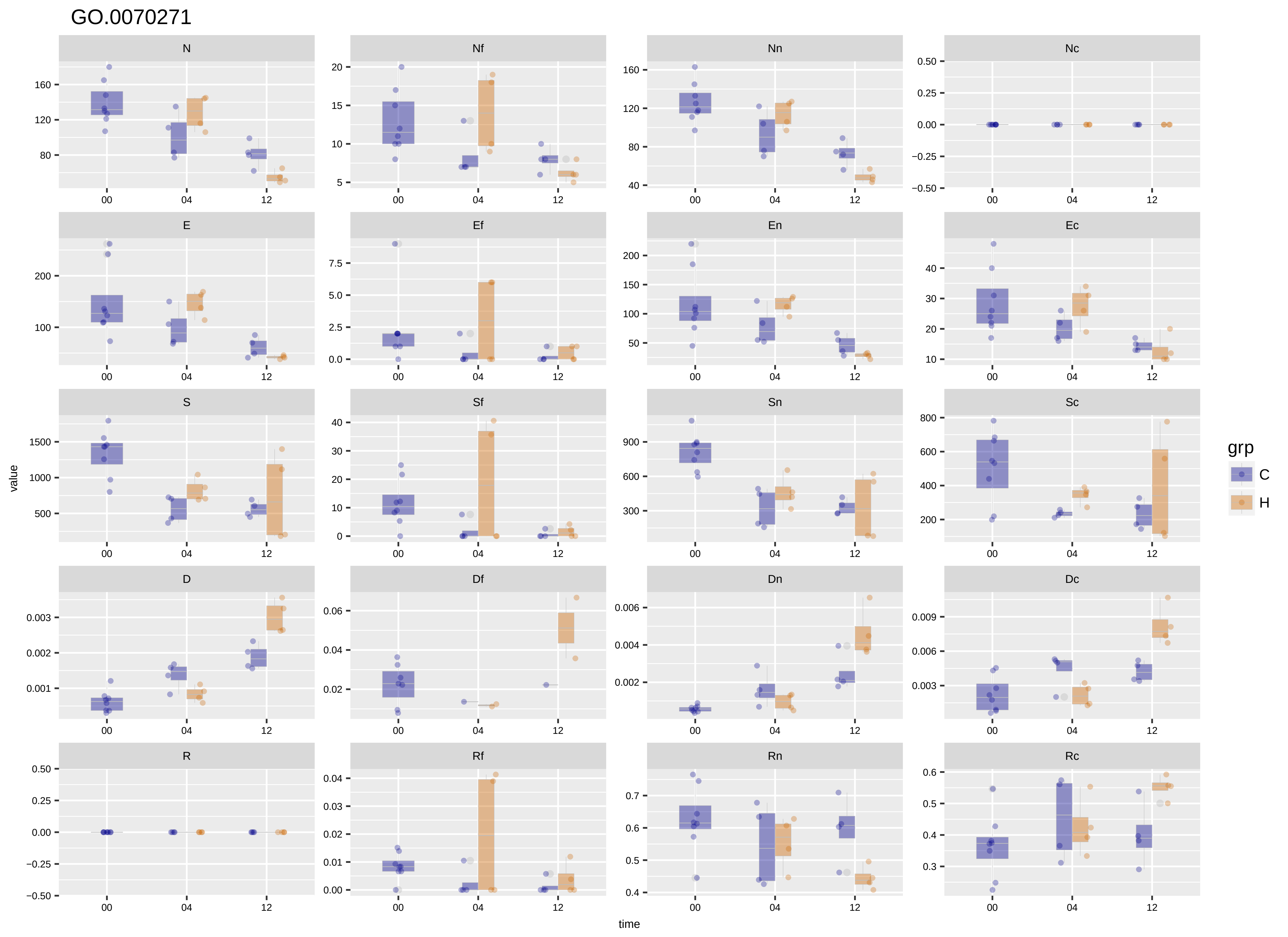

GO.0070271
N
Nf
Nn
Nc
E
Ef
En
Ec
S
Sf
Sn
Sc
D
Df
Dn
Dc
R
Rf
Rn
Rc
value
time
grp
C
H

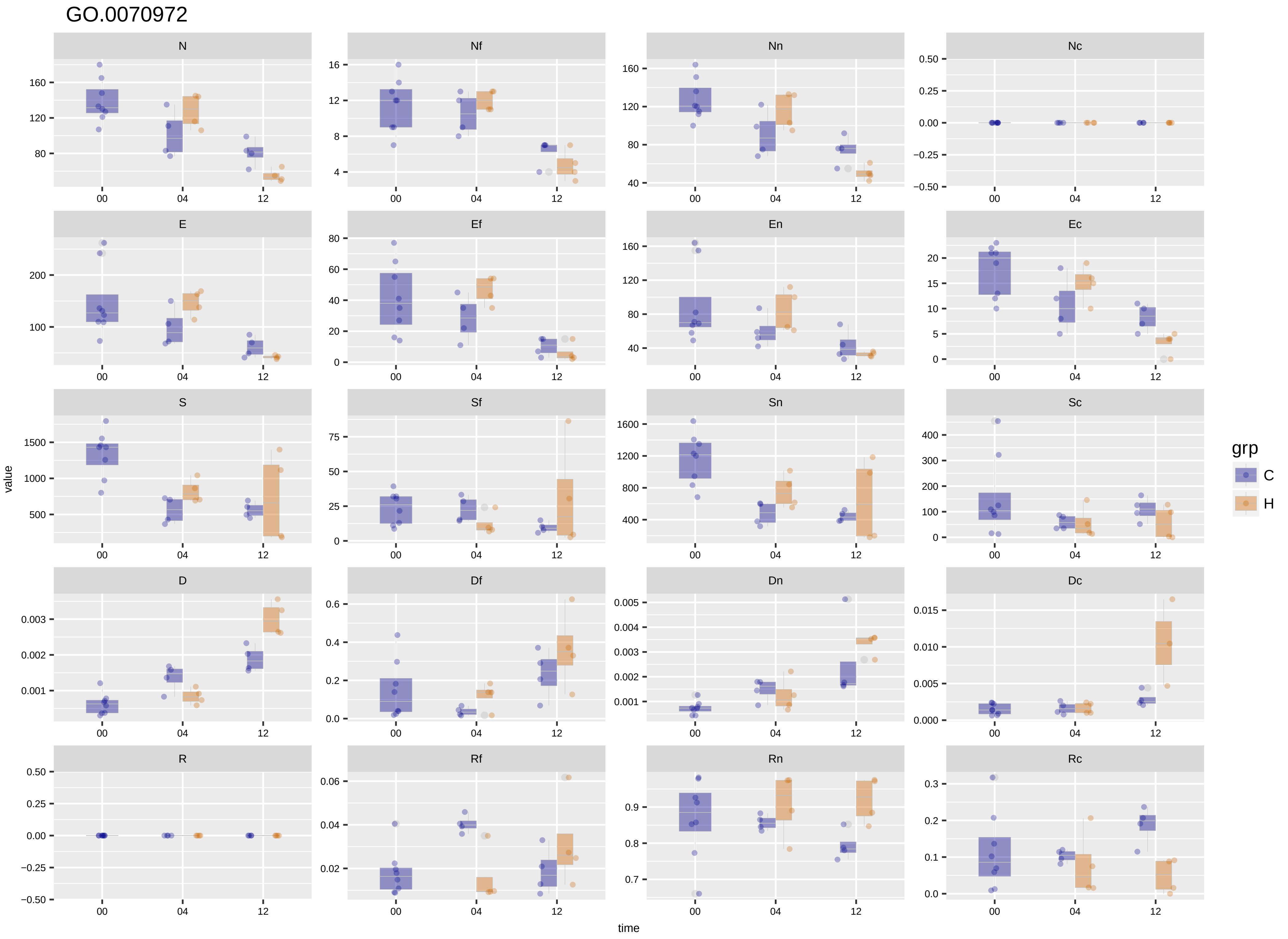

GO.0070972
N
Nf
Nn
Nc
E
Ef
En
Ec
S
Sf
Sn
Sc
D
Df
Dn
Dc
R
Rf
Rn
Rc
value
time
grp
C
H

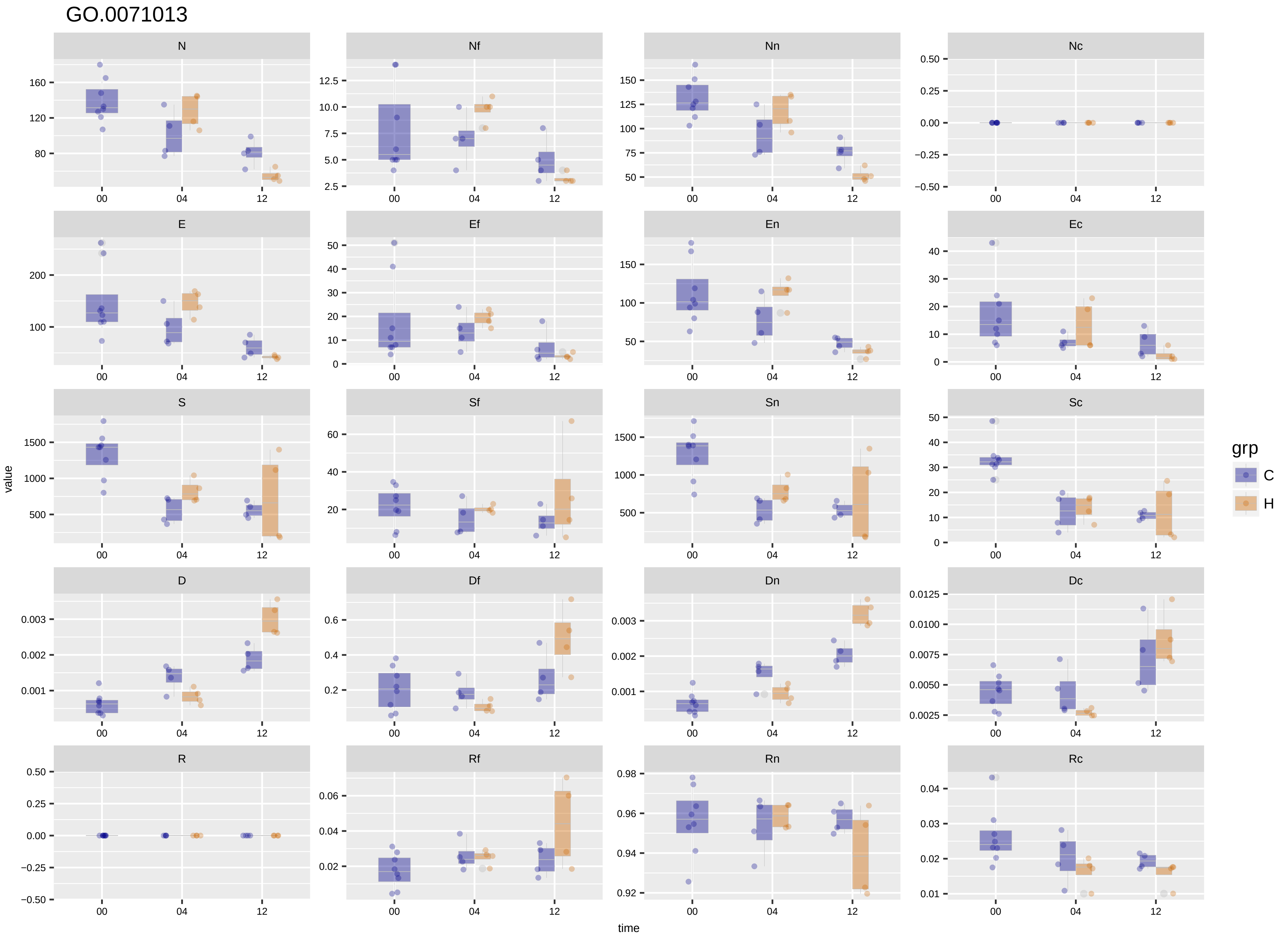
GO.0071013
N
Nf
Nn
Nc
E
Ef
En
Ec
S
Sf
Sn
Sc
D
Df
Dn
Dc
R
Rf
Rn
Rc
value
time
grp
C
H

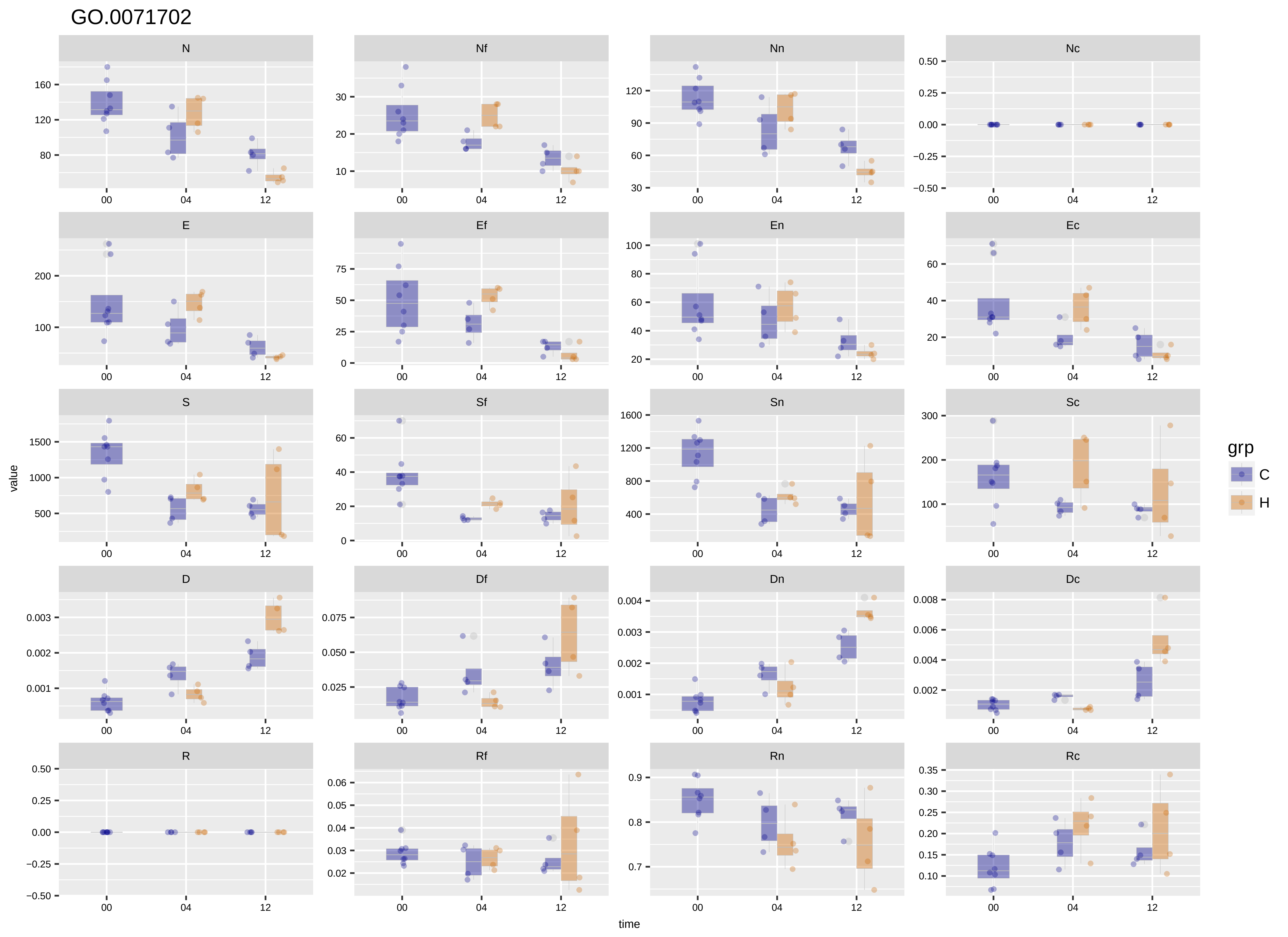

GO.0071702
N
Nf
Nn
Nc
E
Ef
En
Ec
S
Sf
Sn
Sc
D
Df
Dn
Dc
R
Rf
Rn
Rc
value
time
grp
C
H

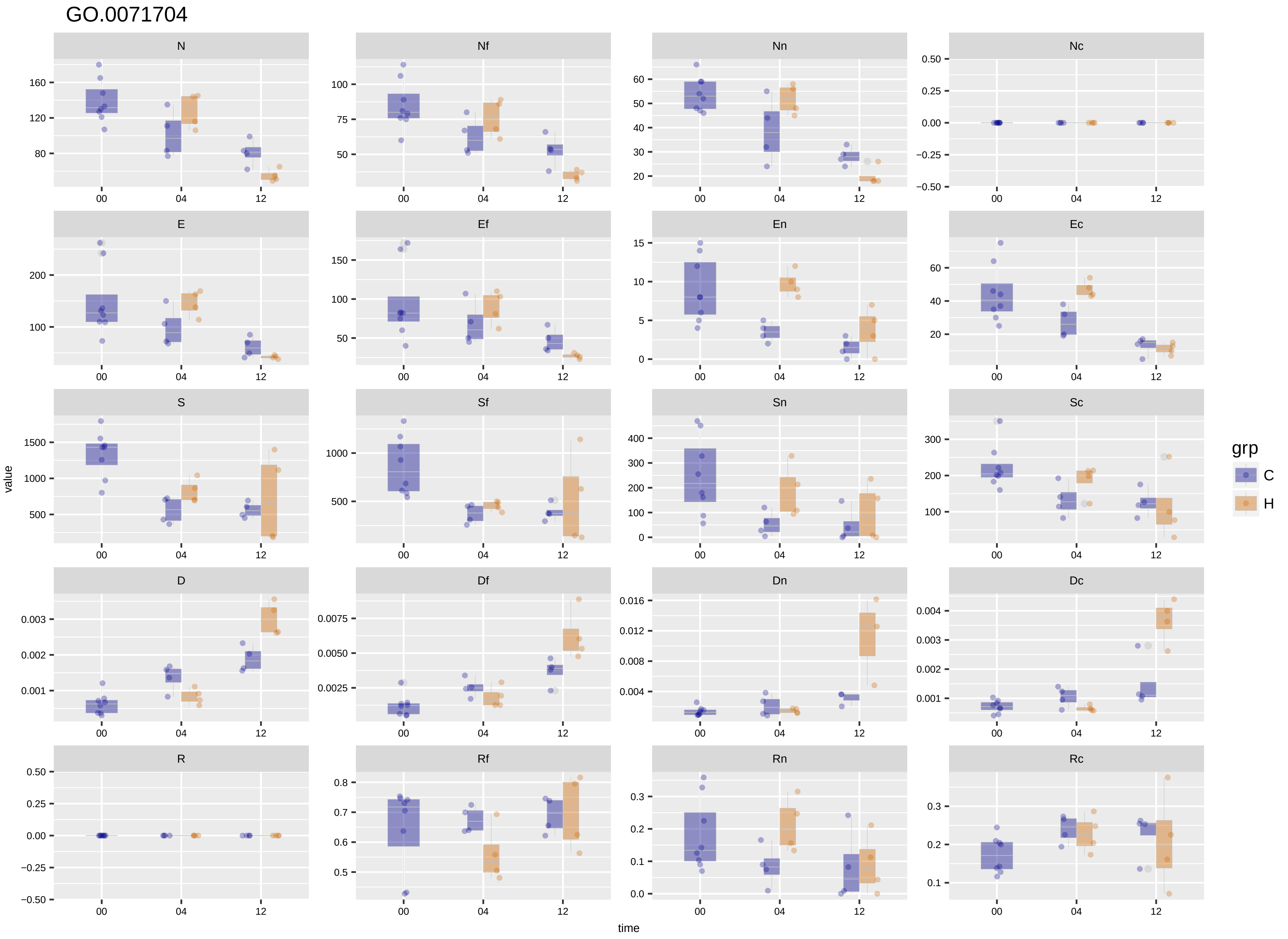

GO.0071704
N
Nf
Nn
Nc
E
Ef
En
Ec
S
Sf
Sn
Sc
D
Df
Dn
Dc
R
Rf
Rn
Rc
value
time
grp
C
H

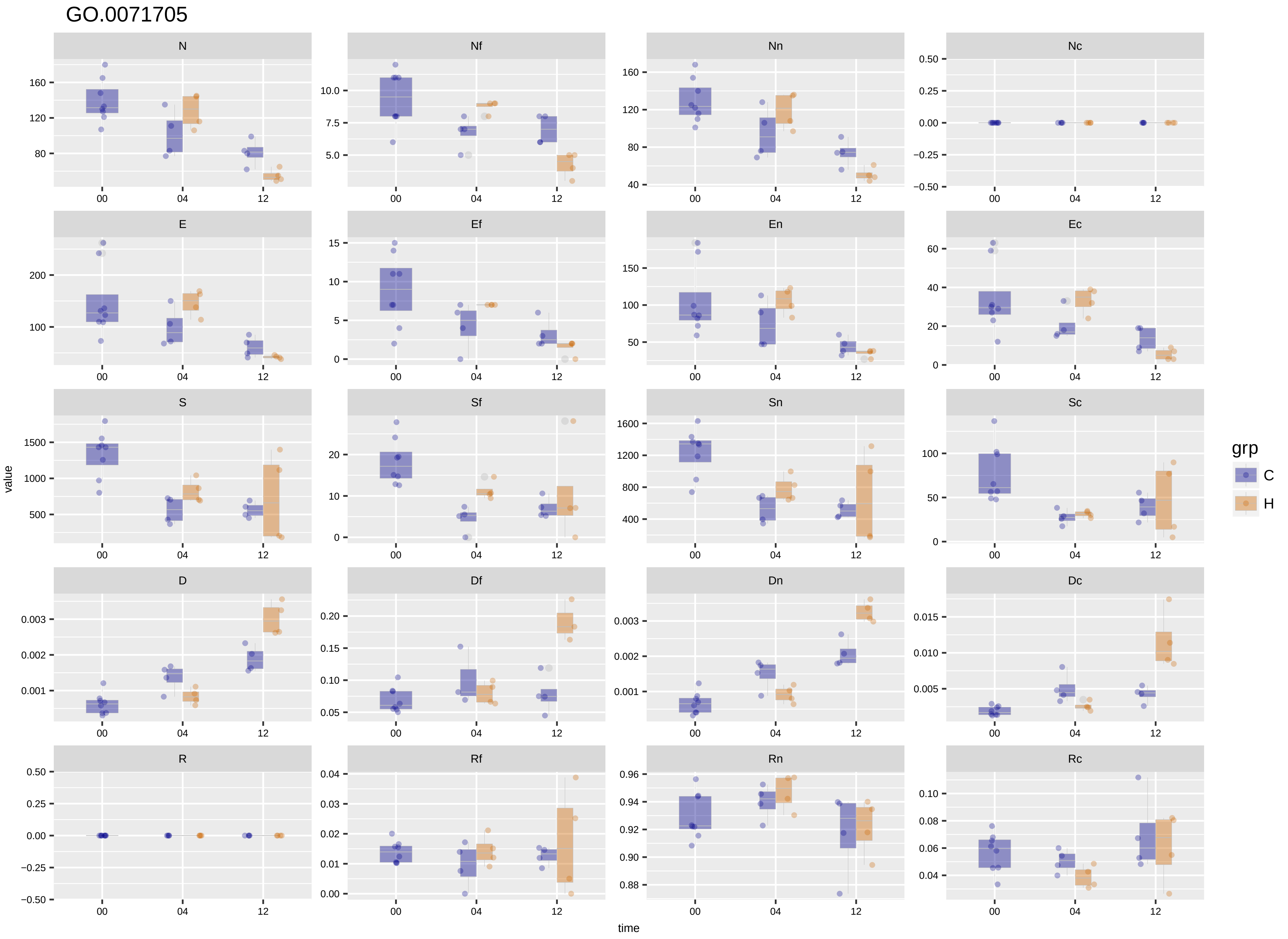

GO.0071705
N
Nf
Nn
Nc
E
Ef
En
Ec
S
Sf
Sn
Sc
D
Df
Dn
Dc
R
Rf
Rn
Rc
value
time
grp
C
H

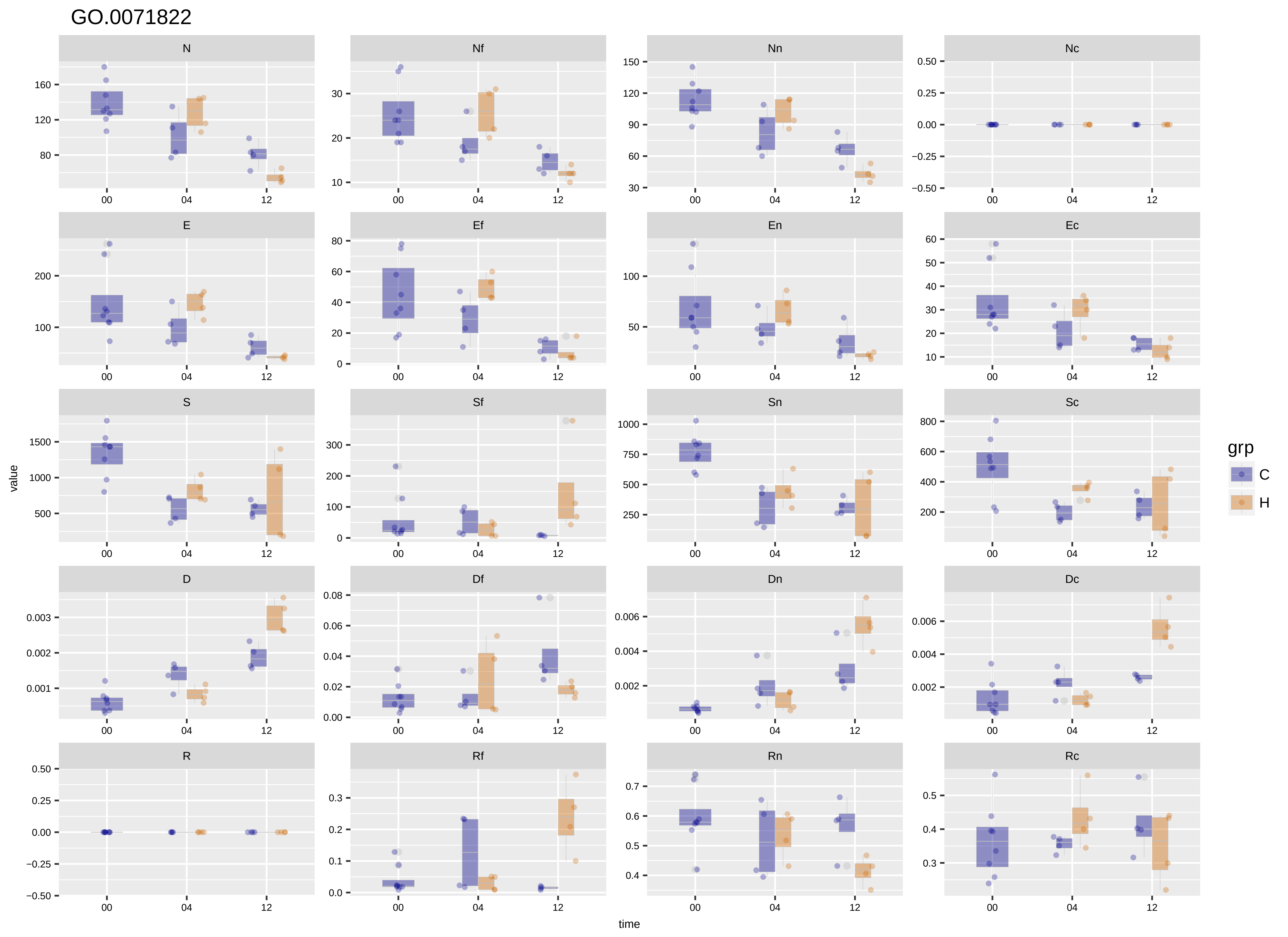
GO.0071822
N
Nf
Nn
Nc
E
Ef
En
Ec
S
Sf
Sn
Sc
D
Df
Dn
Dc
R
Rf
Rn
Rc
00
04
12
value
time
grp
C
H

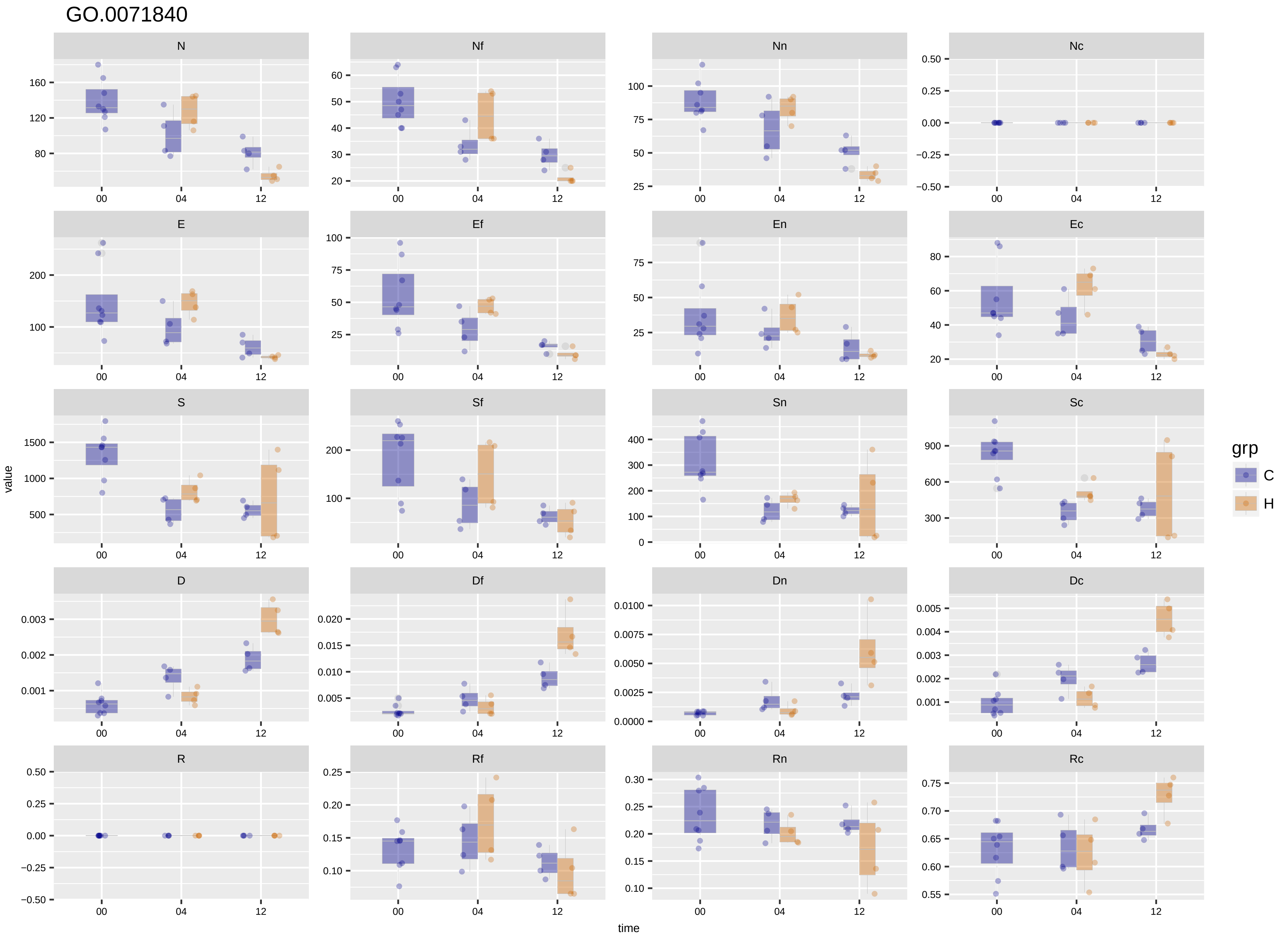

GO.0071840
N
Nf
Nn
Nc
E
Ef
En
Ec
S
Sf
Sn
Sc
D
Df
Dn
Dc
R
Rf
Rn
Rc
00
04
12
value
time
grp
C
H

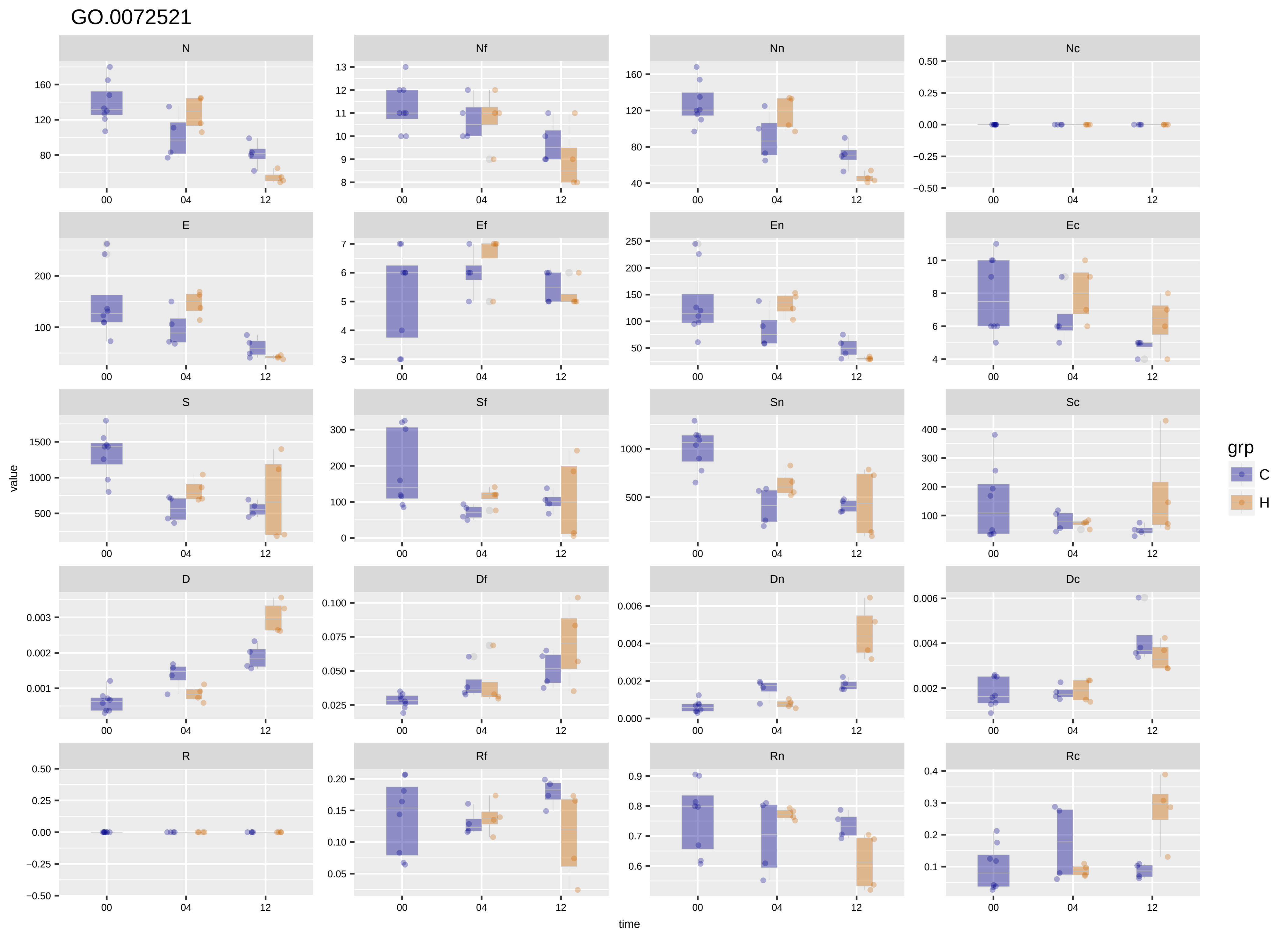

GO.0072521
N
Nf
Nn
Nc
E
Ef
En
Ec
S
Sf
Sn
Sc
D
Df
Dn
Dc
R
Rf
Rn
Rc
value
time
grp
C
H

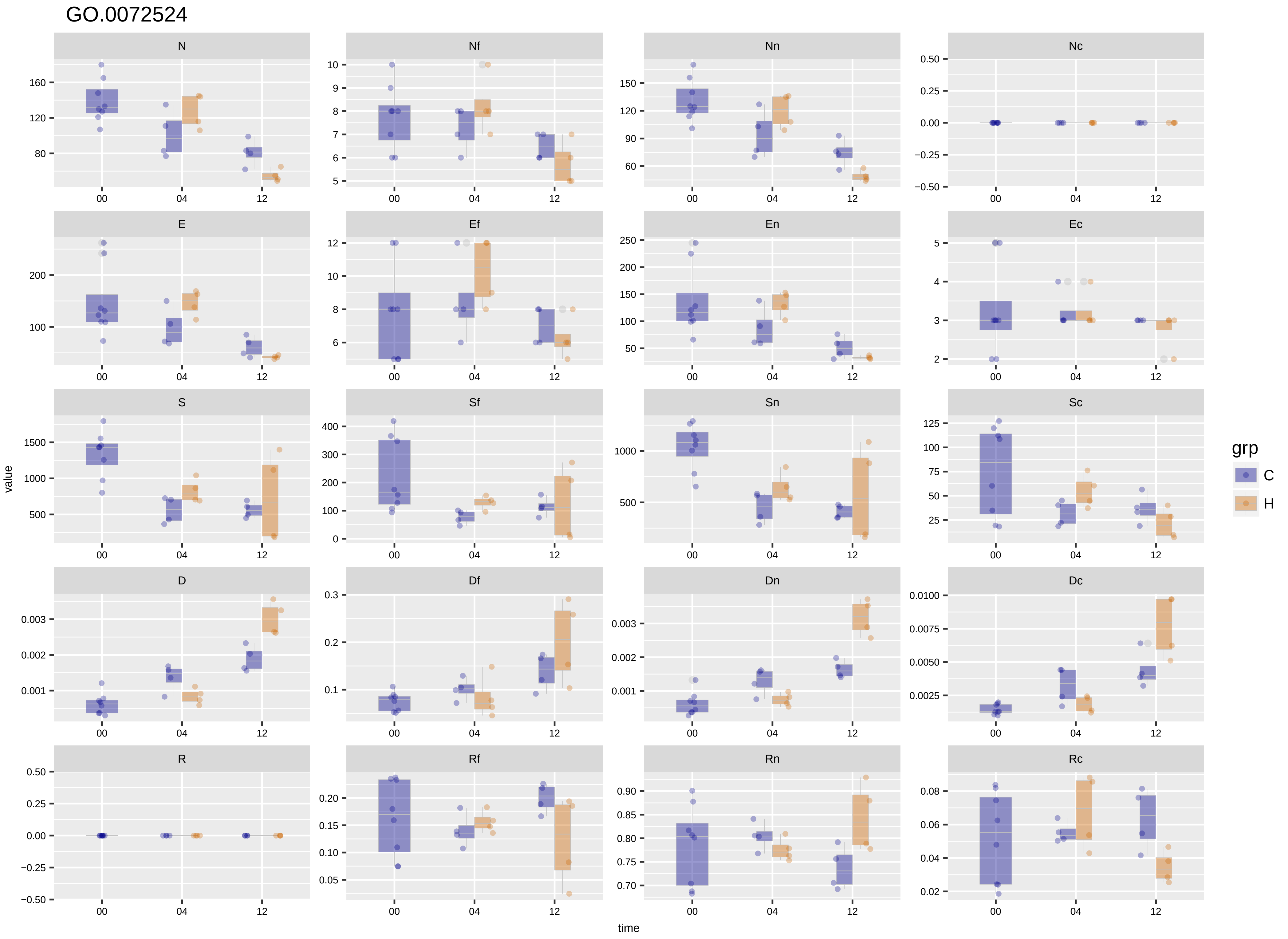
GO.0072524
N
Nf
Nn
Nc
E
Ef
En
Ec
S
Sf
Sn
Sc
D
Df
Dn
Dc
R
Rf
Rn
Rc
value
time
grp
C
H

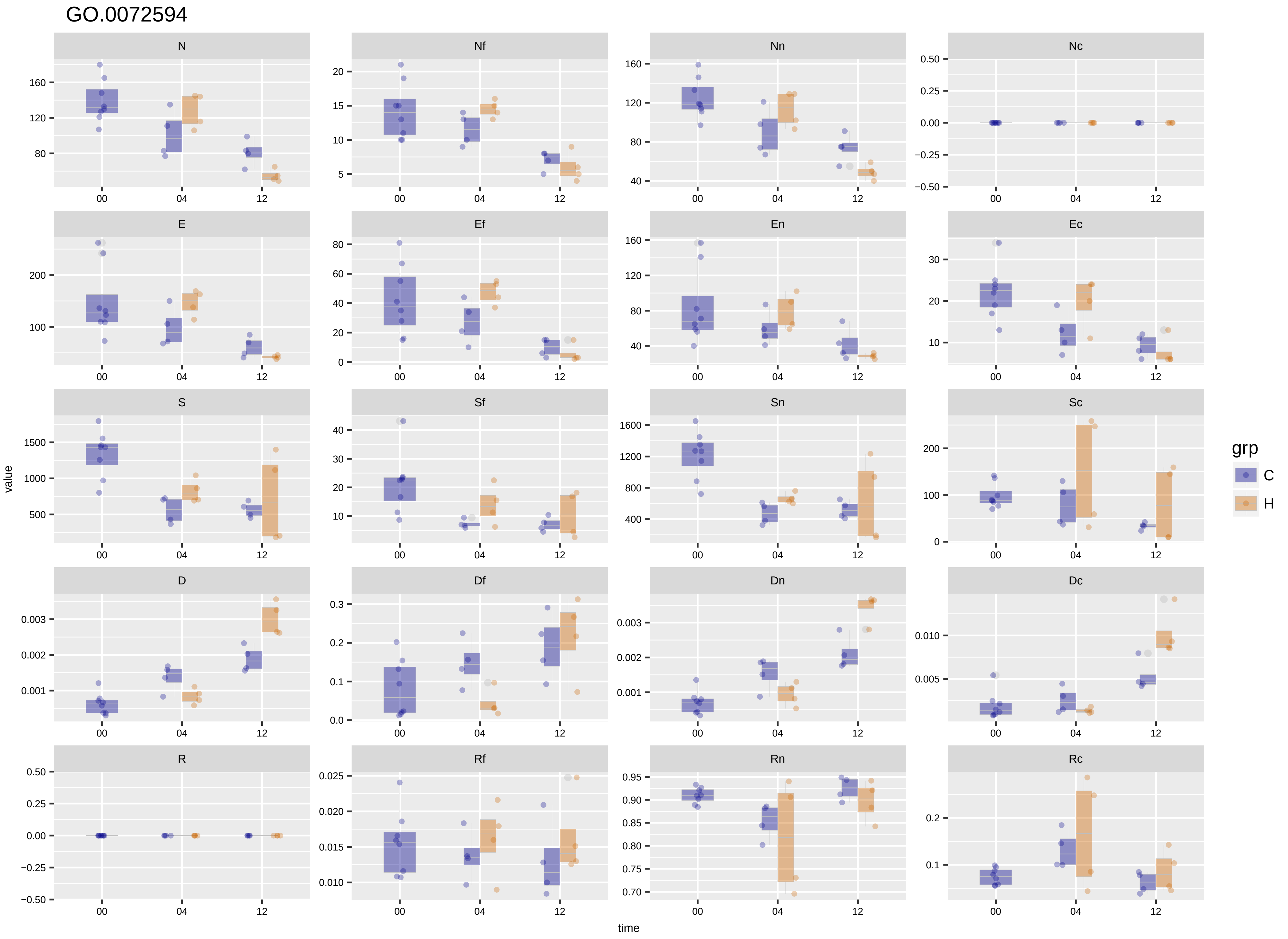
GO.0072594
N
Nf
Nn
Nc
E
Ef
En
Ec
S
Sf
Sn
Sc
D
Df
Dn
Dc
R
Rf
Rn
Rc
value
time
grp
C
H

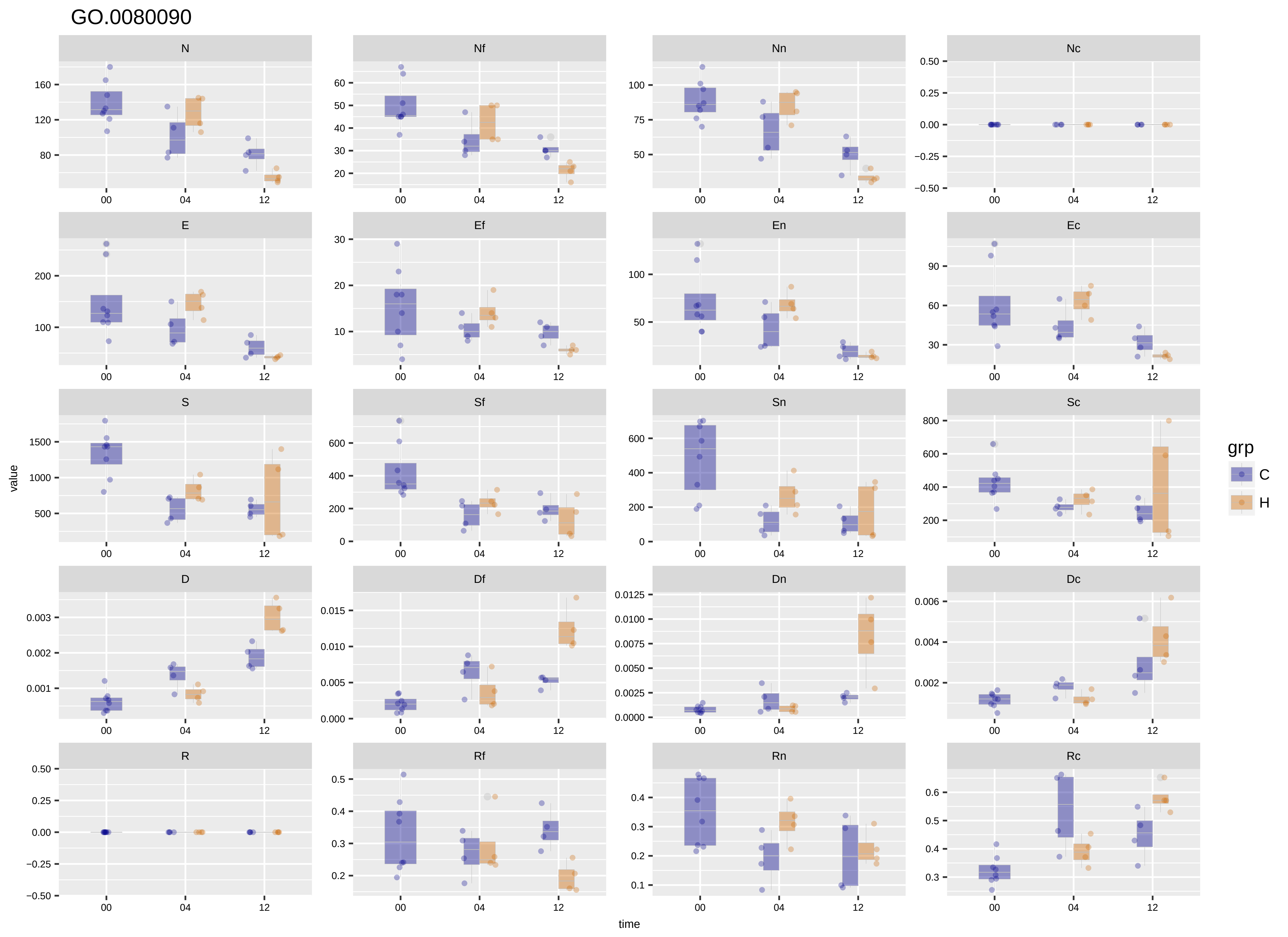

GO.0080090
N
Nf
Nn
Nc
E
Ef
En
Ec
S
Sf
Sn
Sc
D
Df
Dn
Dc
R
Rf
Rn
Rc
00
04
12
value
time
grp
C
H

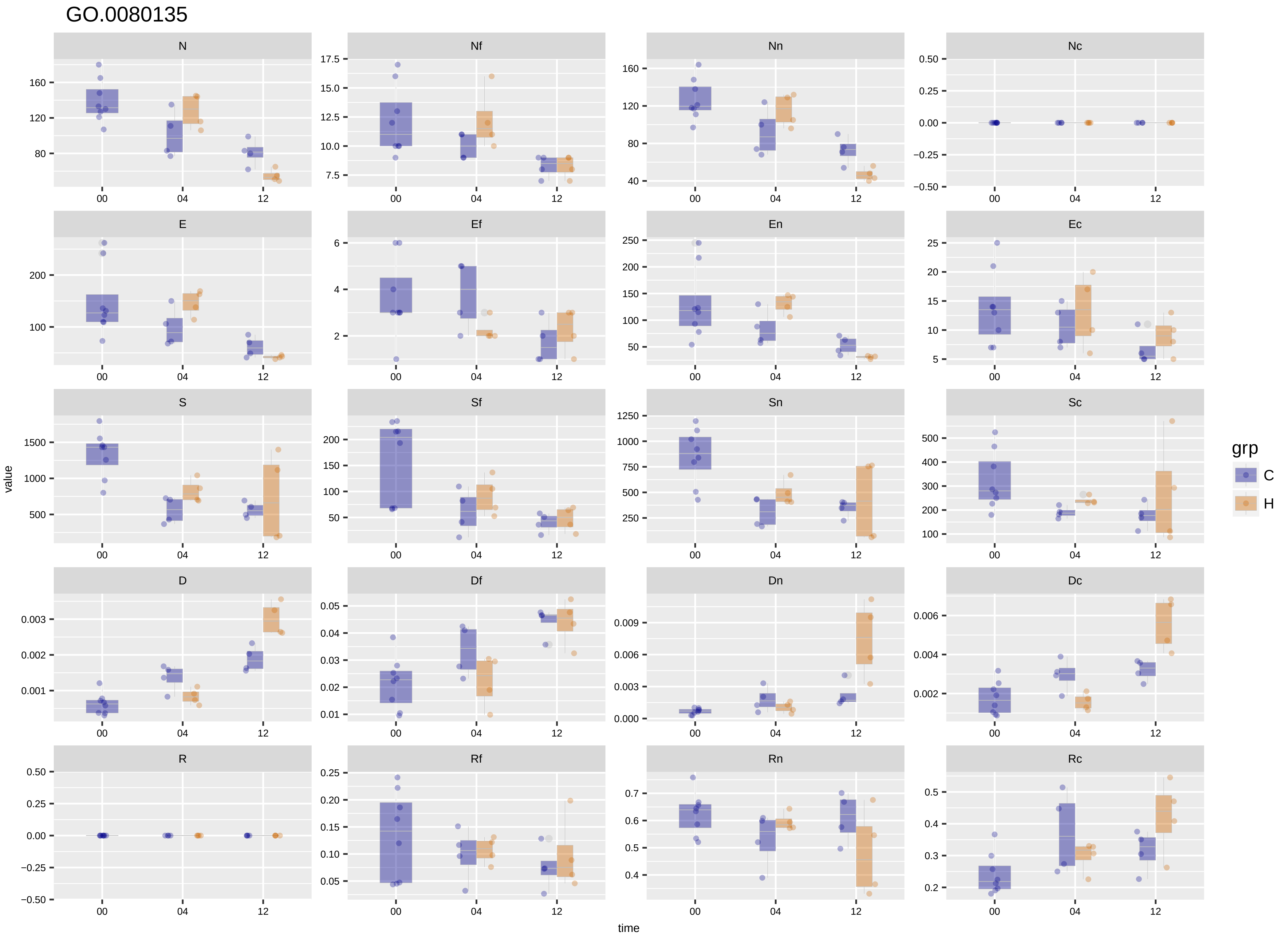
GO.0080135
N
Nf
Nn
Nc
E
Ef
En
Ec
S
Sf
Sn
Sc
D
Df
Dn
Dc
R
Rf
Rn
Rc
00
04
12
value
time
grp
C
H

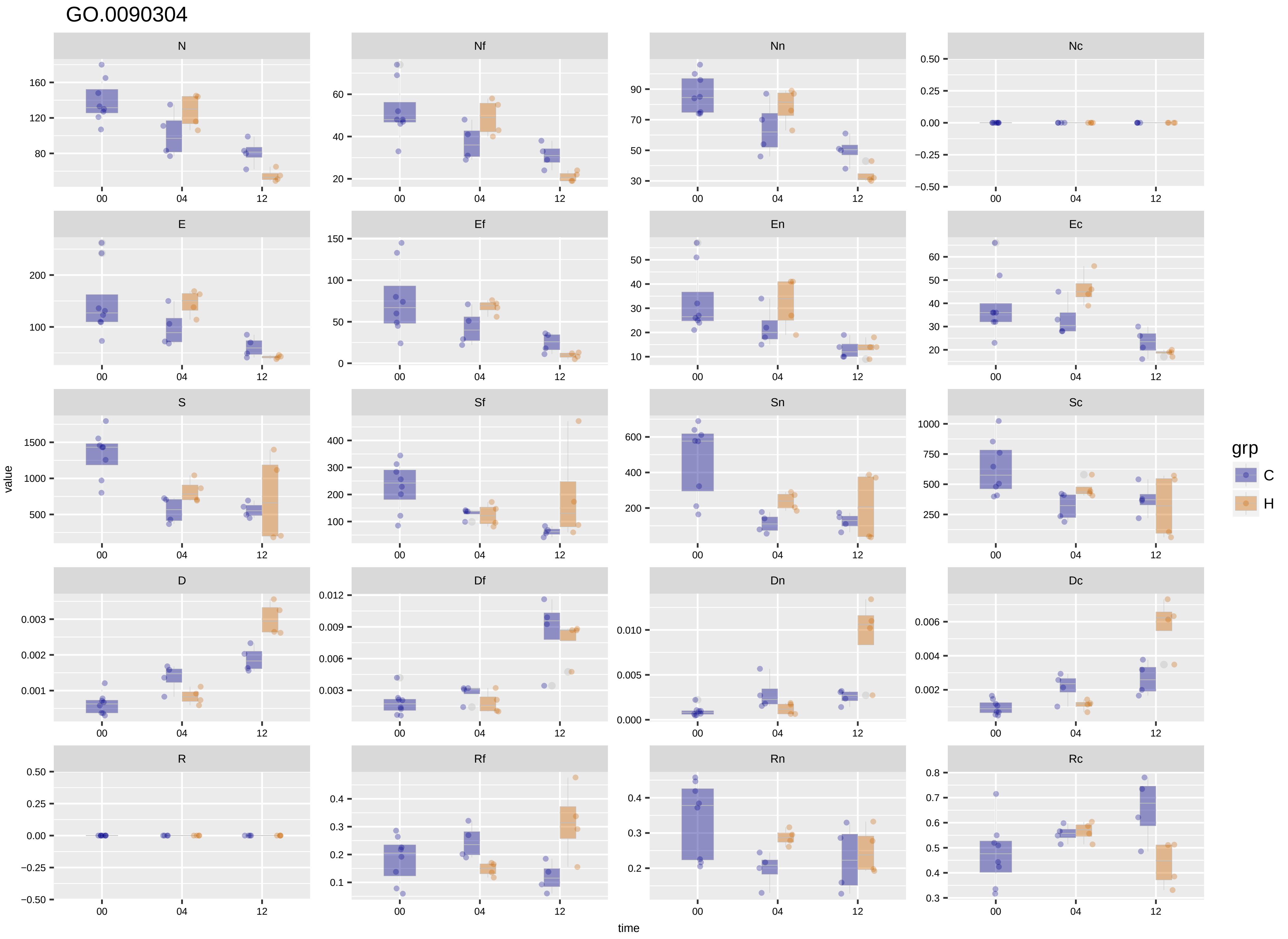

GO.0090304
N
Nf
Nn
Nc
E
Ef
En
Ec
S
Sf
Sn
Sc
D
Df
Dn
Dc
R
Rf
Rn
Rc
value
time
grp
C
H

GO.0097159

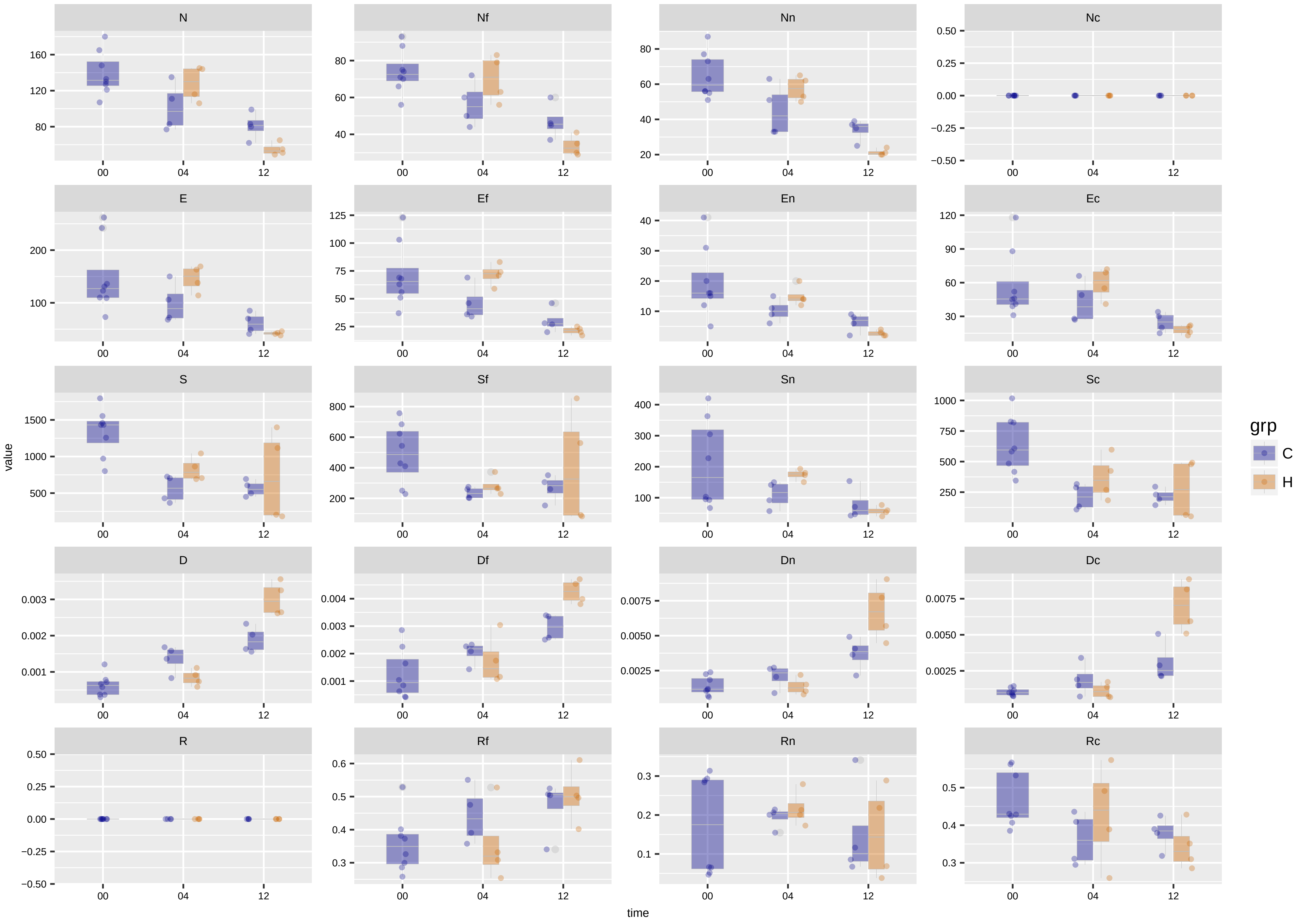
N
Nf
Nn
Nc
E
Ef
En
Ec
S
Sf
Sn
Sc
D
Df
Dn
Dc
R
Rf
Rn
Rc
value
time
00
04
12
grp
C
H

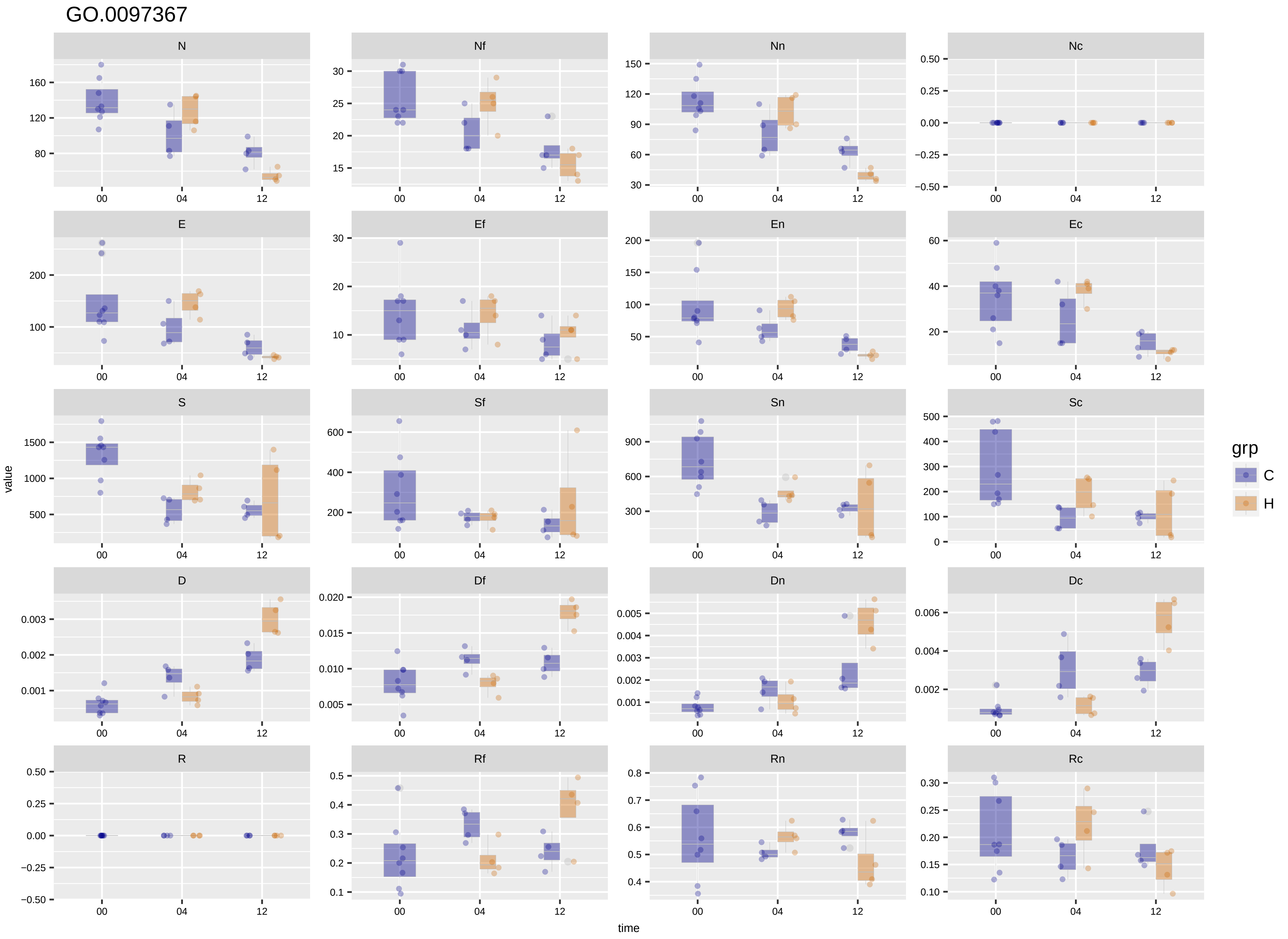
GO.0097367
N
Nf
Nn
Nc
E
Ef
En
Ec
S
Sf
Sn
Sc
D
Df
Dn
Dc
R
Rf
Rn
Rc
00
04
12
value
time
grp
C
H

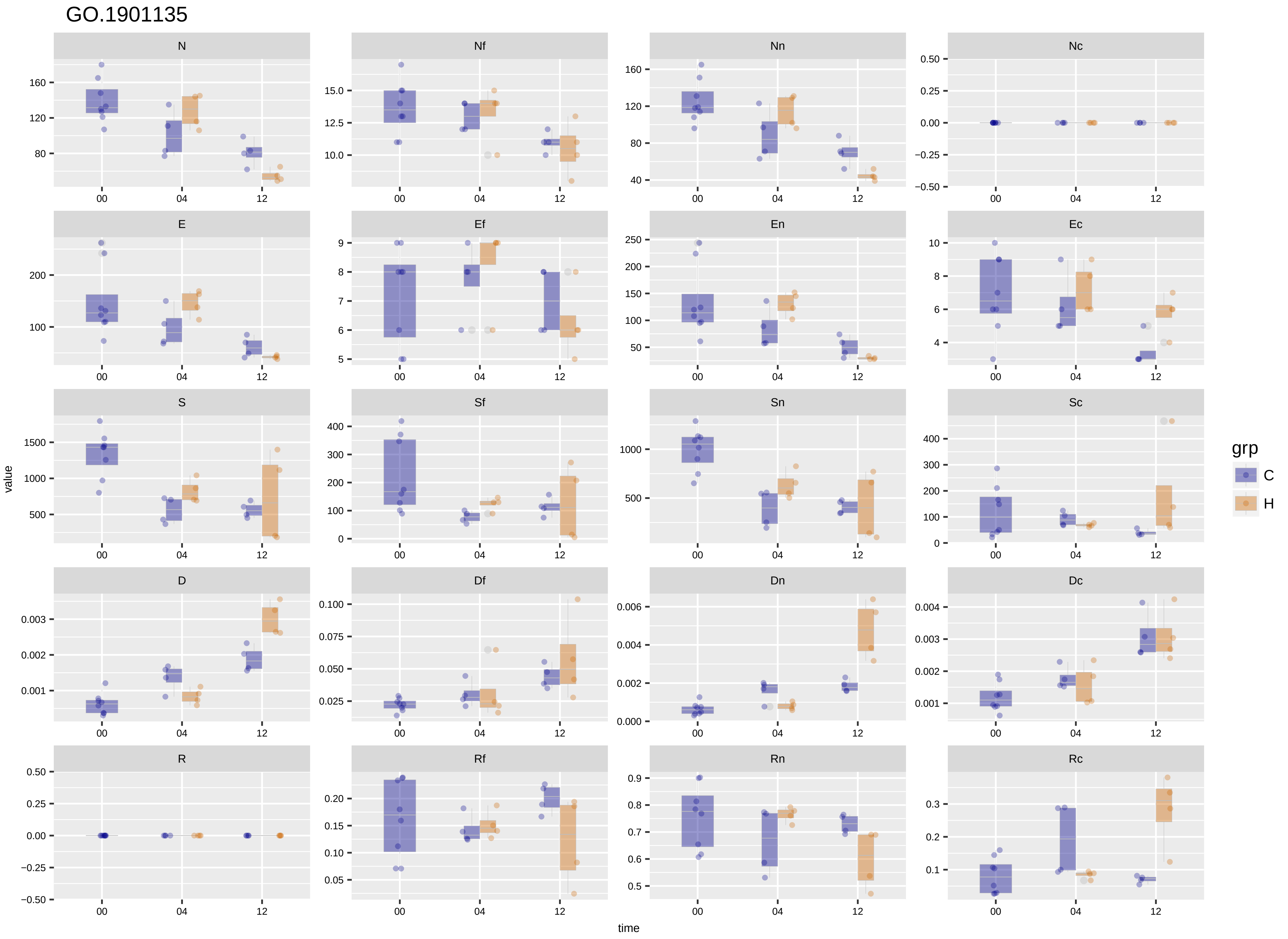
GO.1901135
N
Nf
Nn
Nc
E
Ef
En
Ec
S
Sf
Sn
Sc
D
Df
Dn
Dc
R
Rf
Rn
Rc
value
time
grp
C
H

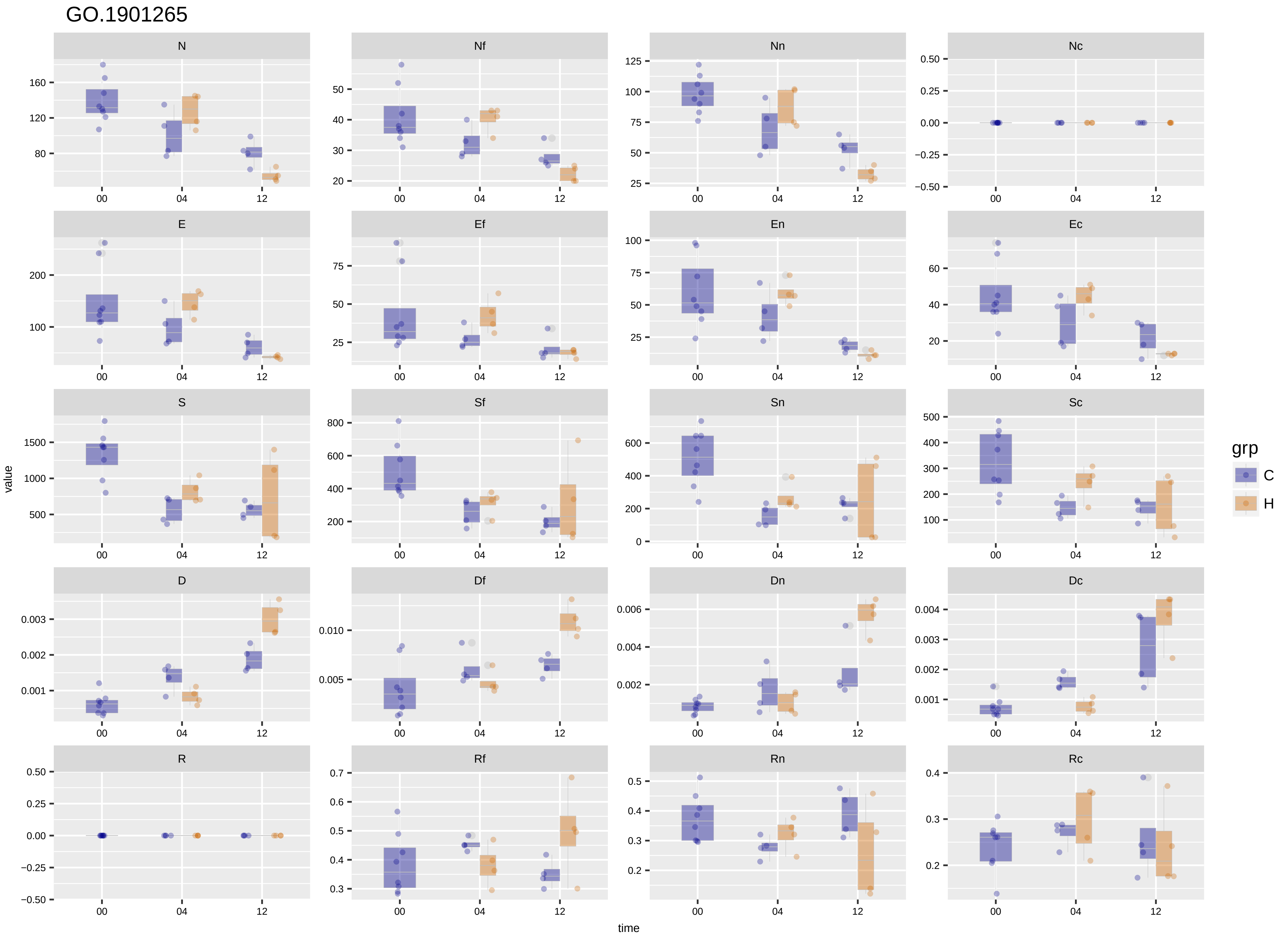

GO.1901265
N
Nf
Nn
Nc
E
Ef
En
Ec
S
Sf
Sn
Sc
D
Df
Dn
Dc
R
Rf
Rn
Rc
value
time
grp
C
H

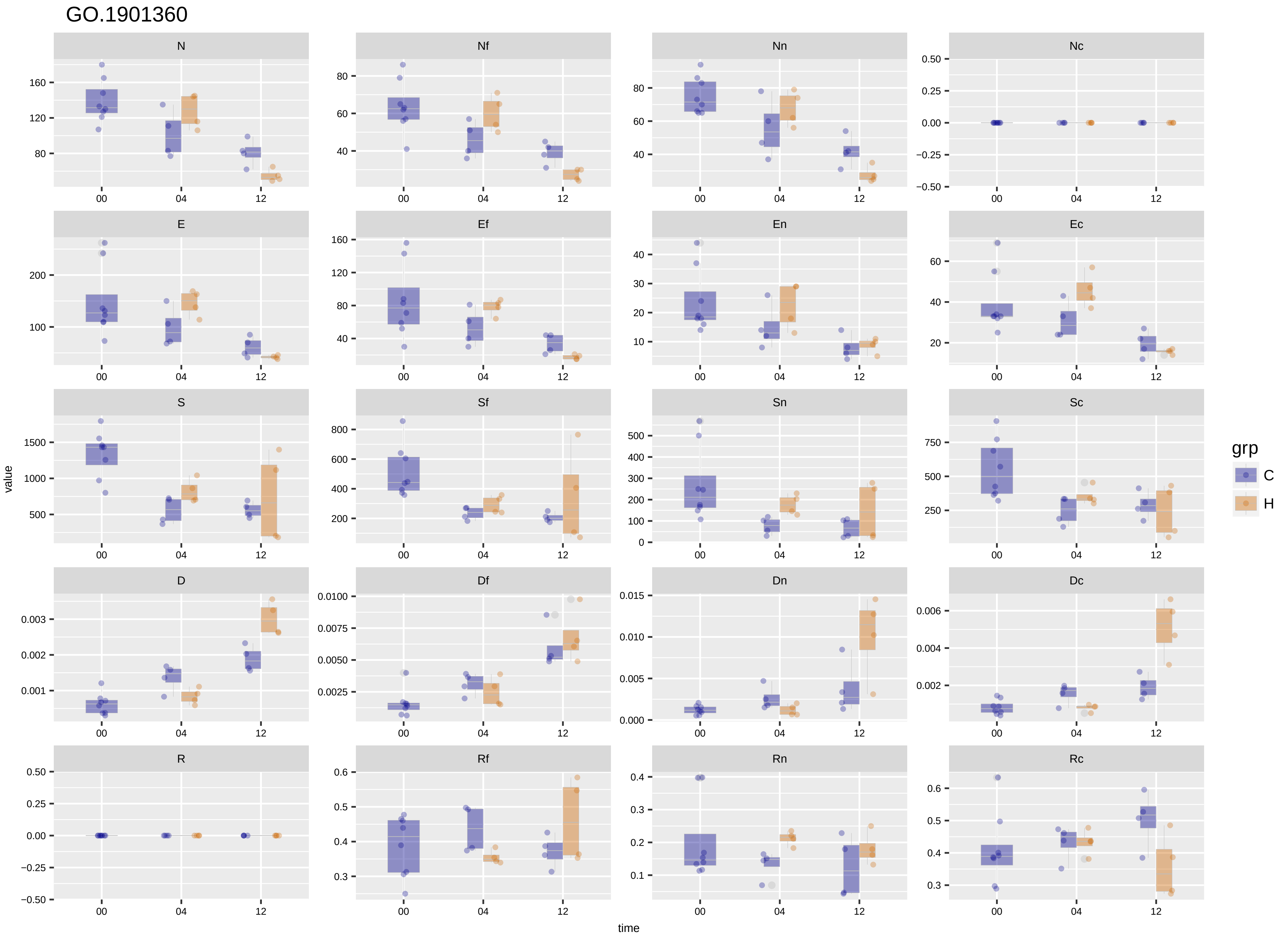

GO.1901360
N
Nf
Nn
Nc
E
Ef
En
Ec
S
Sf
Sn
Sc
D
Df
Dn
Dc
R
Rf
Rn
Rc
value
time
00
04
12
grp
C
H

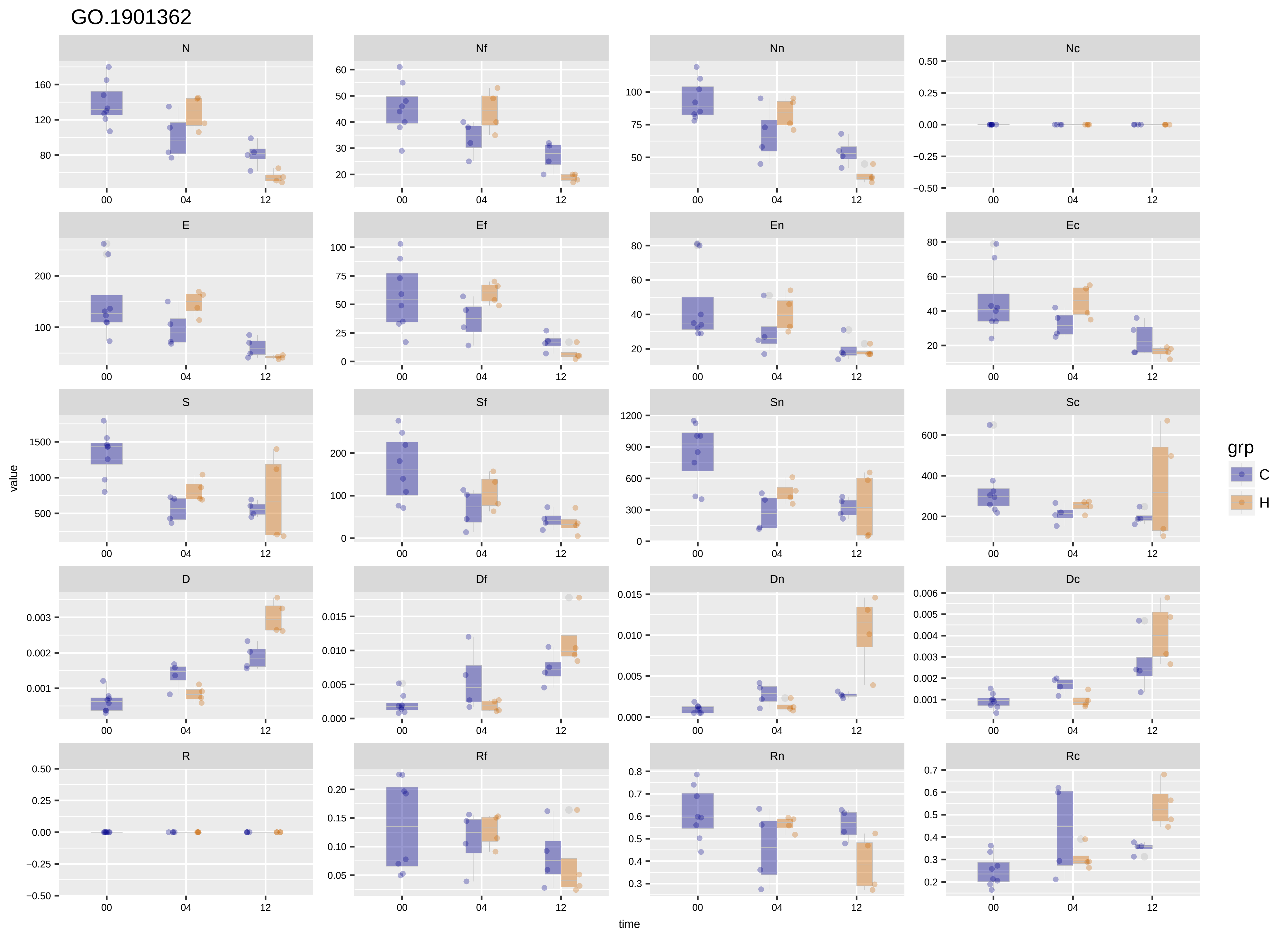
GO.1901362
N
Nf
Nn
Nc
E
Ef
En
Ec
S
Sf
Sn
Sc
D
Df
Dn
Dc
R
Rf
Rn
Rc
value
time
grp
C
H

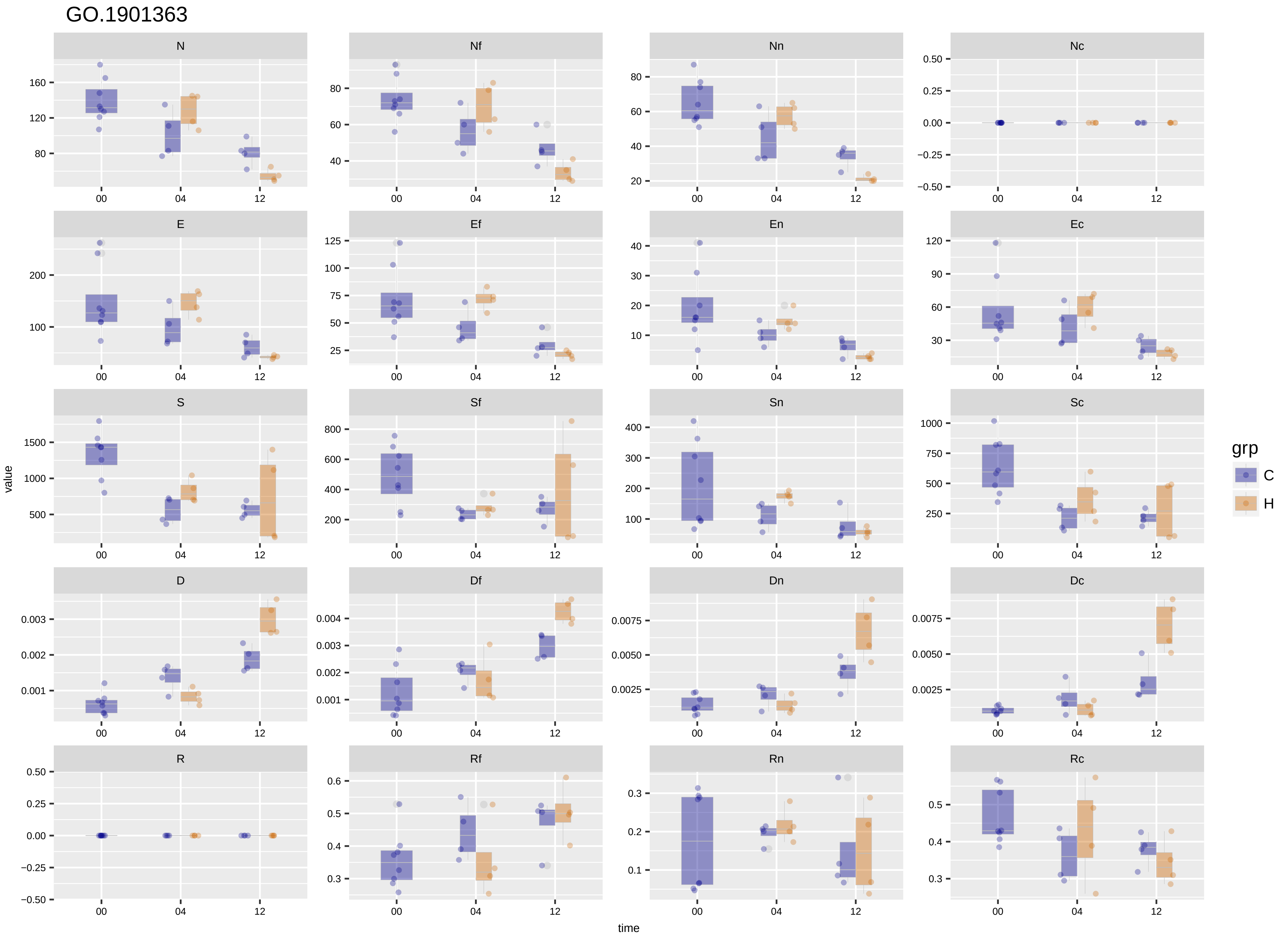

GO.1901363
N
Nf
Nn
Nc
E
Ef
En
Ec
S
Sf
Sn
Sc
D
Df
Dn
Dc
R
Rf
Rn
Rc
value
time
grp
C
H

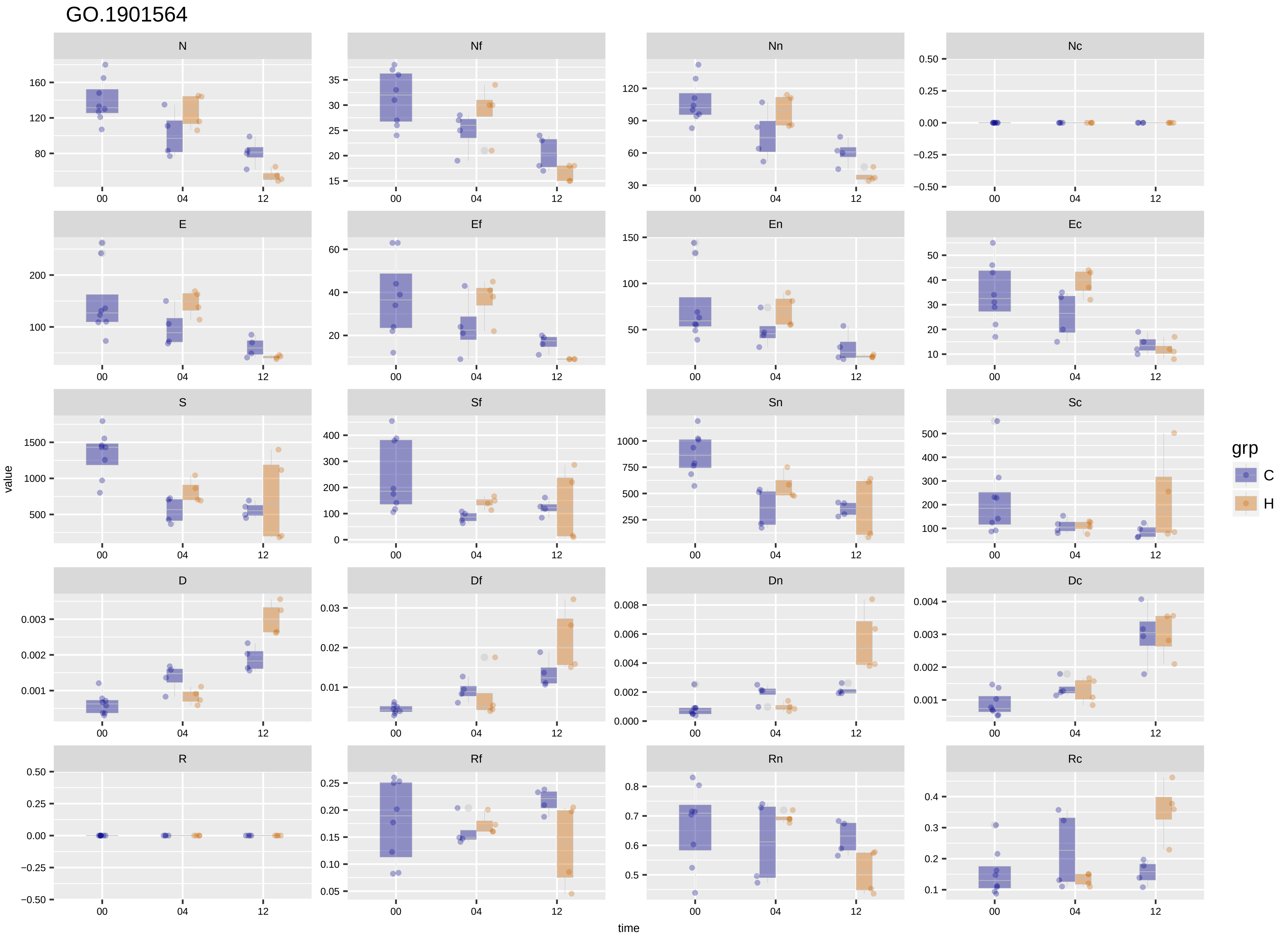

GO.1901564
N
Nf
Nn
Nc
E
Ef
En
Ec
S
Sf
Sn
Sc
D
Df
Dn
Dc
R
Rf
Rn
Rc
value
time
grp
C
H

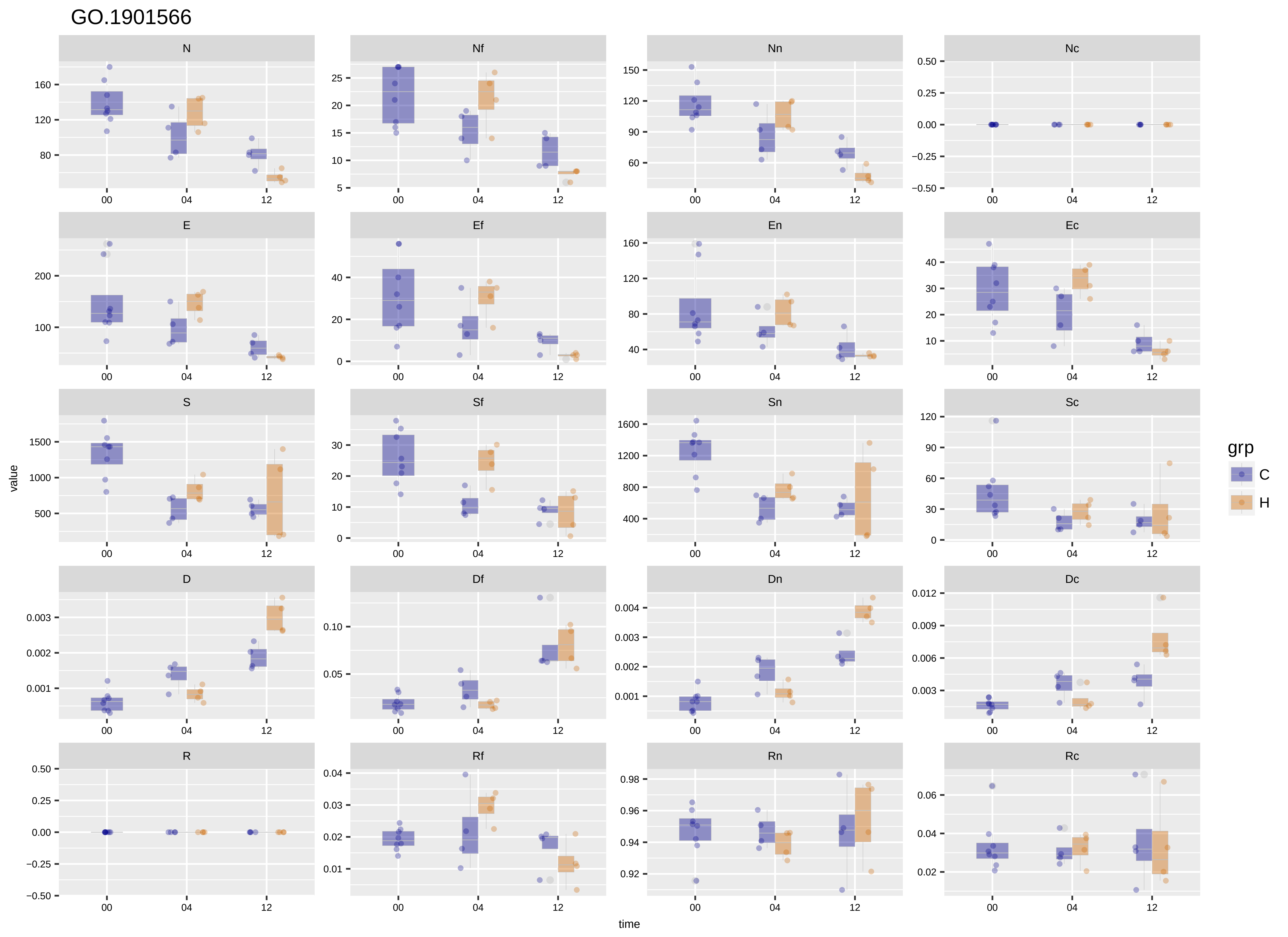

GO.1901566
N
Nf
Nn
Nc
E
Ef
En
Ec
S
Sf
Sn
Sc
D
Df
Dn
Dc
R
Rf
Rn
Rc
00
04
12
value
time
grp
C
H

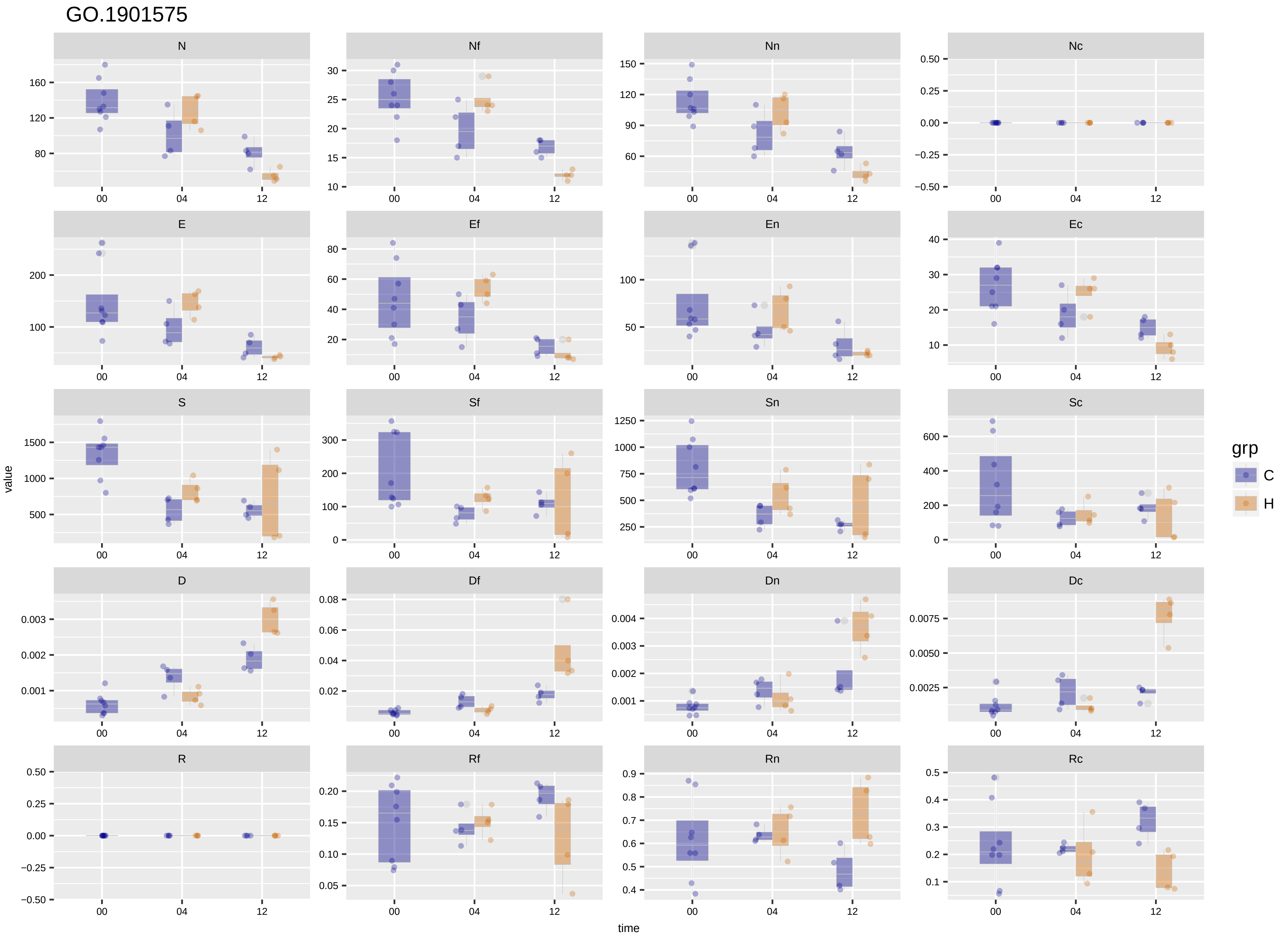

GO.1901575
N
Nf
Nn
Nc
E
Ef
En
Ec
S
Sf
Sn
Sc
D
Df
Dn
Dc
R
Rf
Rn
Rc
00
04
12
value
time
grp
C
H

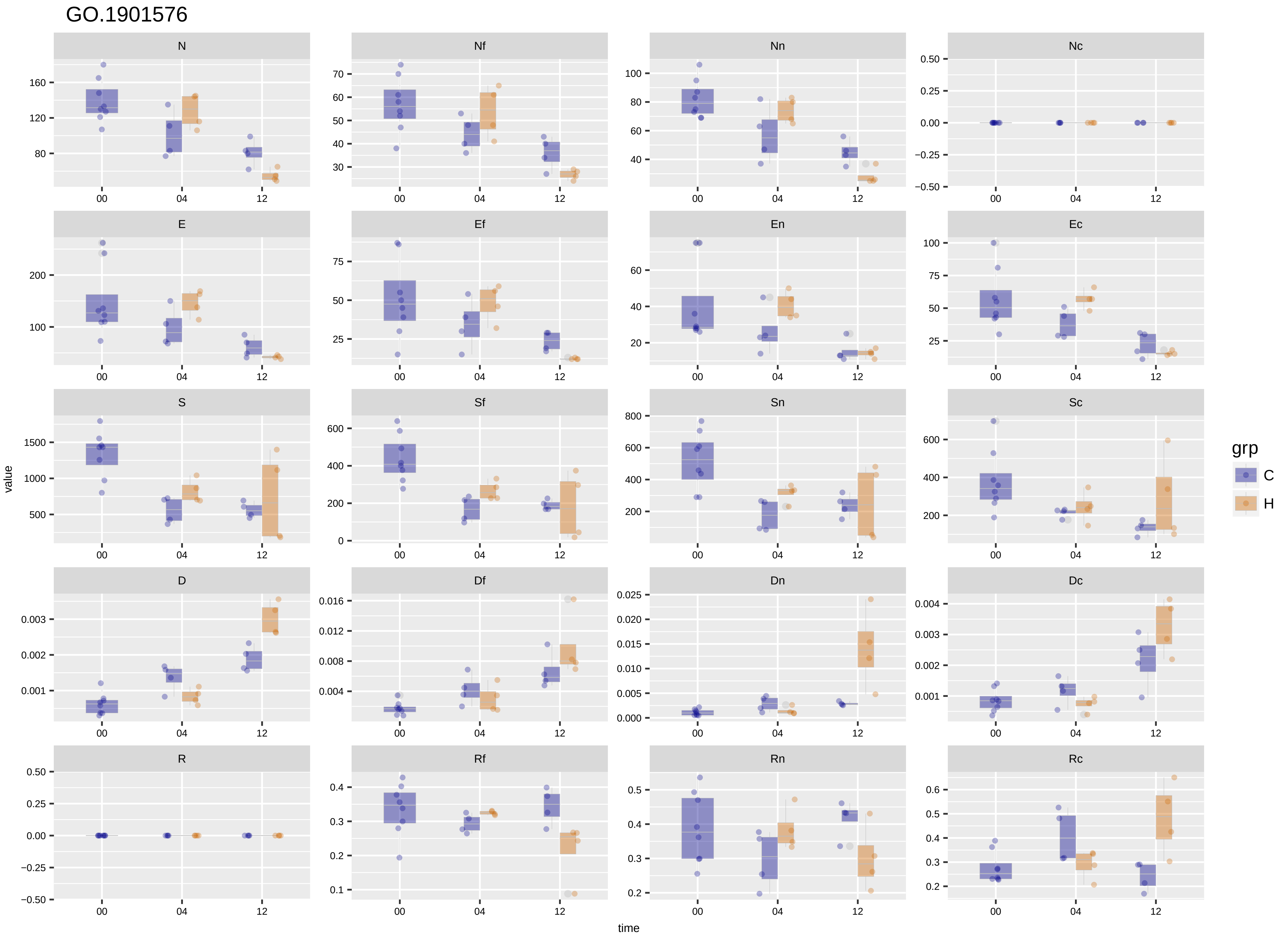
GO.1901576
N
Nf
Nn
Nc
E
Ef
En
Ec
S
Sf
Sn
Sc
D
Df
Dn
Dc
R
Rf
Rn
Rc
00
04
12
value
time
grp
C
H

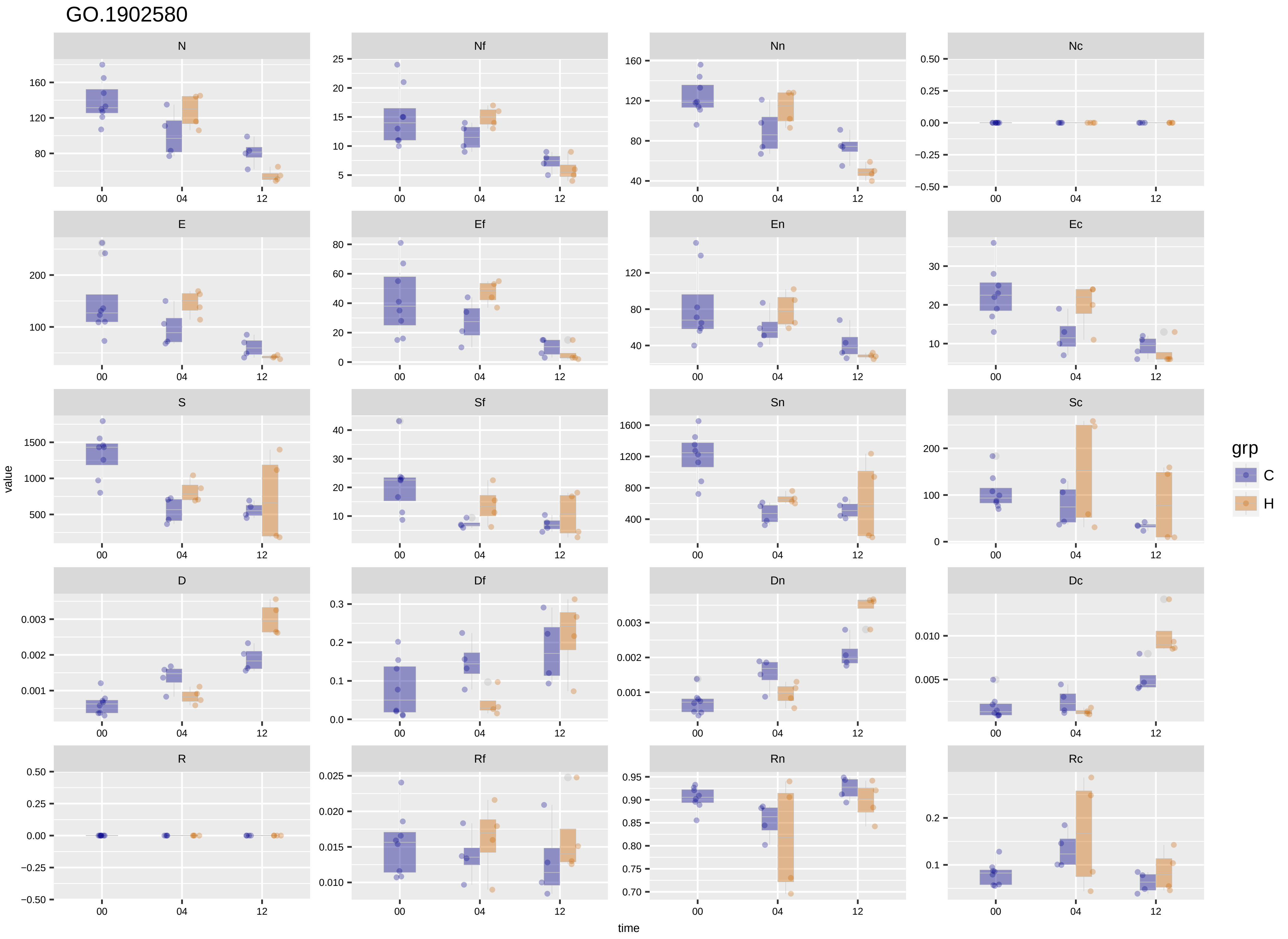

GO.1902580
N
Nf
Nn
Nc
E
Ef
En
Ec
S
Sf
Sn
Sc
D
Df
Dn
Dc
R
Rf
Rn
Rc
value
time
grp
C
H

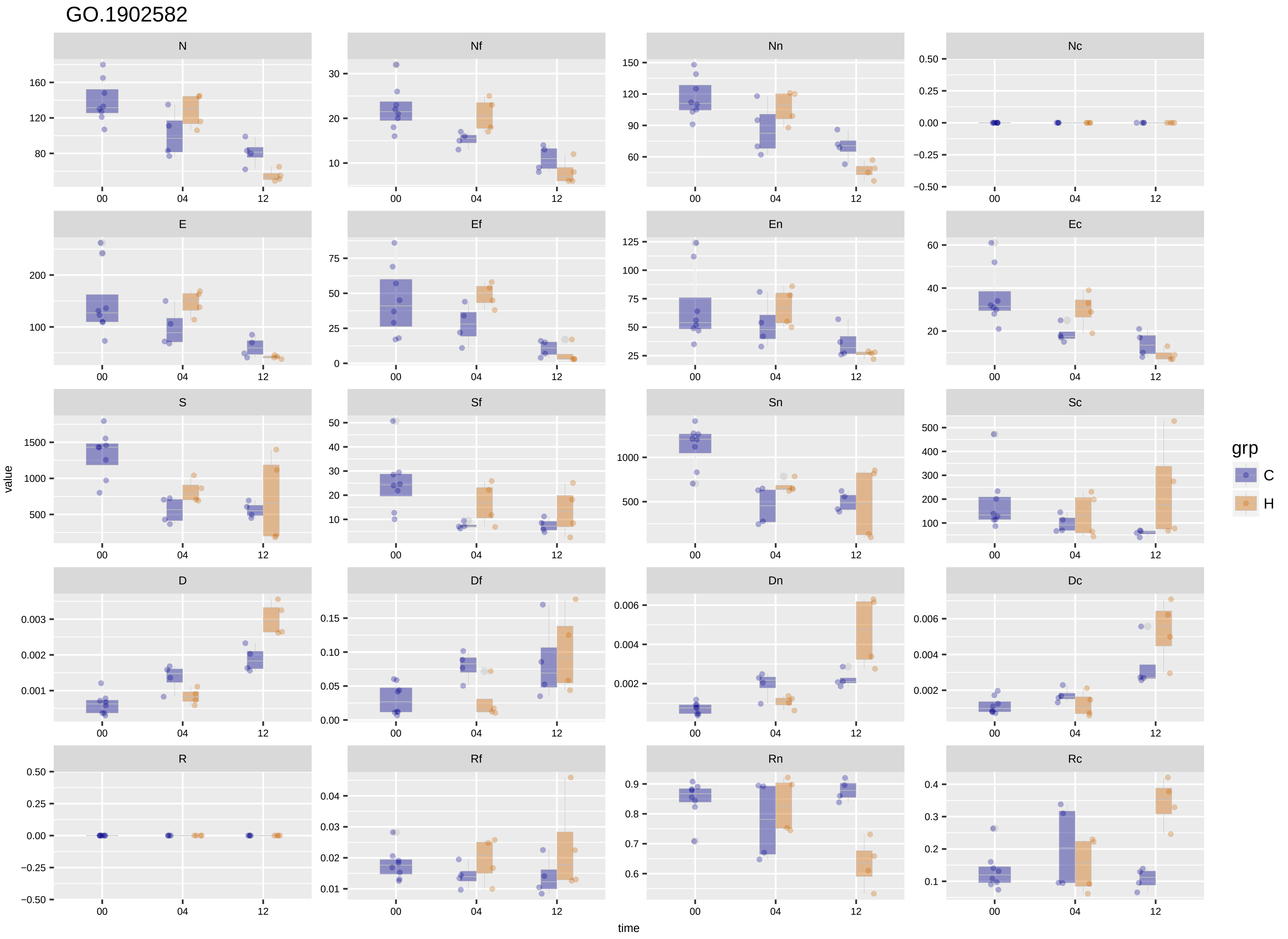
GO.1902582
N
Nf
Nn
Nc
E
Ef
En
Ec
S
Sf
Sn
Sc
D
Df
Dn
Dc
R
Rf
Rn
Rc
value
time
00
04
12
grp
C
H
